# Facile (*Z*)‐Selective Synthesis of β,γ‐Unsaturated Ketones by a Silicon‐based Olefination Strategy

**DOI:** 10.1002/anie.202517069

**Published:** 2025-10-23

**Authors:** Daniya Aynetdinova, Jakub Brześkiewicz, Nikolaos Skoulikas, Nuno Maulide

**Affiliations:** ^1^ Institute of Organic Chemistry University of Vienna Währinger Straße 38 Vienna Austria

**Keywords:** Charge relocation, Vinyl silanes, (*Z*)‐Selective olefination

## Abstract

A novel silicon‐based olefination strategy to access challenging β,γ‐(*Z*)‐unsaturated ketones with high diastereoselectivity in a single step from easily accessible, branched vinyl silanes is presented. This new disconnection resolves many longstanding challenges that have prevented general and efficient synthetic approaches to (*Z*)‐deconjugated enones, affording a broad scope of this type of alkenes with high functional group tolerance. The developed transformation has been applied as a key step in the synthesis of a range of natural products.

## Graphical abstract

A silicon‐based olefination strategy has been developed to access challenging (*Z*)‐configured β,γ‐unsaturated ketones from readily accessible branched vinyl silanes and acid chlorides. This method enables the straightforward synthesis of a broad variety of deconjugated ketones with high (*Z*)‐selectivity under mild conditions and provides a new retrosynthetic disconnection to streamline the synthesis of complex scaffolds and natural products.

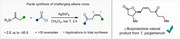

The stereoselective synthesis of (*Z*)‐alkenes is a fundamental, yet challenging part of the synthetic toolbox.^[^ ^1^, ^2^, ^3^, ^4^, ^5^, ^6^, ^7^, ^8^ ^]^ Although there are established methods to access (*Z*)‐alkenes bearing aliphatic,^[^ ^9^, ^10^, ^11^, ^12^ ^]^ aromatic,^[^ ^13^, ^14^, ^15^, ^16^, ^17^, ^18^ ^]^ and electron‐withdrawing substituents (e.g., α,β‐unsaturated carbonyls),^[^ ^19^, ^20^, ^21^, ^22^, ^23^, ^24^ ^]^ the stereoselective synthesis of alkenes bearing an adjacent nonconjugated electron‐withdrawing group (EWG) is considerably more challenging (Scheme 1a).^[^ ^1^, ^2^, ^3^, ^4^ ^]^ In particular, the synthesis of valuable β,γ‐(*Z*)‐unsaturated ketones in high (*Z*)‐selectivity is particularly rare and remains a largely unresolved challenge in olefination strategies.^[^ ^25^, ^26^, ^27^, ^28^ ^]^ One of the recent breakthroughs includes reductive isomerization to produce β,γ‐(*Z*)‐unsaturated carbonyl compounds; however, this approach often necessitates elaborate substrate synthesis, and ketone functional groups remain uncommon.^[^ ^25^ ^]^ This is a significant limitation, considering that a large number of natural products possess this motif (Scheme 1a).^[^ ^7^ ^]^ Furthermore, β,γ‐(*Z*)‐unsaturated ketones can serve as one‐step precursors to (*Z*‐)configured homoallylic alcohols, a common structural motif in hydroxy fatty acids.^[^ ^5^ ^]^ The development of an efficient (*Z*)‐selective synthesis of β,γ‐unsaturated ketones is, therefore, a highly desirable prospect.

**Scheme 1:**
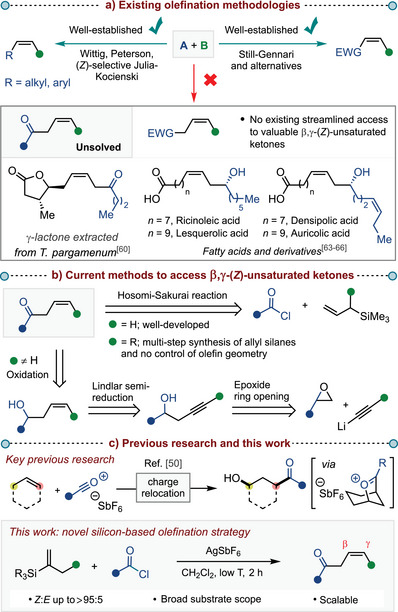
a) *Overview of (Z)‐selective olefination methodologies*. b) *Current methods to access β,γ‐(Z)‐unsaturated ketones*. c) *Previous research and this work*.

Conventional (*Z*)‐selective olefination approaches, such as the Wittig reaction with unstabilized ylides^[^ ^29^, ^30^ ^]^ or the Still–Gennari^[^ ^21^ ^]^ and Ando variants^[^ ^20^ ^]^ of the Horner–Wadsworth–Emmons reaction, typically require basic conditions to generate the necessary reactive intermediates, which are incompatible with substrate structures required for the synthesis of β,γ‐unsaturated ketones. This limitation was partly addressed in recent work by Harutyunyan with a modified Wittig salt which enabled the synthesis of (*Z)*‐alkenes carrying less *α*‐acidic groups, such as esters and amides.^[^ ^31^ ^]^ However, access to β,γ‐(*Z*)‐unsaturated ketones remained elusive.

In contrast to the carbanion‐based approaches, the Hosomi–Sakurai reaction^[^ ^32^, ^33^, ^34^ ^]^ of allyl silanes with acid chlorides allows access to β,γ‐unsaturated ketones under nonbasic conditions relying on the β‐silicon effect, the ability of silyl groups to stabilize carbocations at the β‐position by hyperconjugation with a C–Si bond (Scheme 1b).^[^ ^35^, ^36^, ^37^, ^38^, ^39^ ^]^ However, this is rarely achieved for substituted allyl silanes, especially intermolecularly, with most examples yielding only terminal olefins. In the few cases where internal alkenes result from (tediously synthesized)^[^ ^40^ ^]^ substituted allyl silanes, the stereoselectivity is not predictable and is highly dependent on the specific substrate combination.^[^ ^41^, ^42^ ^]^ As a result, the most commonly employed, “universal” strategy toward β,γ‐(*Z*)‐unsaturated ketones is a three‐step approach typically consisting of epoxide opening by an alkynyl anion, Lindlar semireduction and oxidation.^[^ ^43^, ^44^, ^45^, ^46^ ^]^ This approach, while reliable, is linear, redox‐inefficient and has intrinsic functional group limitations associated with each step.

To address this long‐standing challenge, we aimed to leverage our group's expertise in carbocation chemistry.^[^ ^47^, ^48^, ^49^ ^]^ In particular, we were interested in exploiting the β‐silicon effect in the context of charge relocation (Scheme 1c, *top*).^[^ ^50^ ^]^ Herein, we present a novel silicon‐based olefination strategy to access challenging β,γ‐(*Z*)‐unsaturated ketones with high diastereoselectivity and functional group tolerance under mild reaction conditions (Scheme 1c, *bottom*).

The electrophilic addition to linear vinyl silanes, for which *ipso*‐substitution is generally observed, is well‐explored.^[^ ^51^, ^52^, ^53^, ^54^, ^55^, ^56^ ^]^ The non‐*ipso* reactivity mode of ***branched*** vinyl silanes, on the other hand, has remained underexplored^[^ ^57^ ^]^ especially in the context of intermolecular C–C bond forming reactions, and our interest was piqued by the possibilities it might offer. The synthesis of the required branched vinyl silane starting materials **2** was achieved in a single step from commercially available or easily accessible alkynes **1** using Trost's hydrosilylation methodology (Scheme 2a).^[^ ^58^, ^59^ ^]^ This reaction proved to be robust and general in our hands, enabling access to a large scope of vinyl silane substrates (see the Supporting Information).

**Scheme 2:**
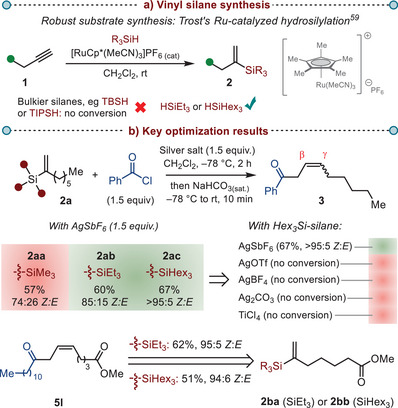
a) *Vinyl silane synthesis*. b) *Optimization of the key reaction parameters*. Isolated yields are reported**;** all *Z*:*E* ratios were determined before column chromatography based on ^1^H NMR analysis of the crude reaction mixture. TBSH—*tert‐*butyldimethylsilane; TIPSH–triisopropyl silane.

With the required substrates in hand, we explored the model reaction between trimethylvinyl silane **2aa** and benzoyl chloride (Scheme 2b). We opted to focus on silver salts to activate acid chlorides by halogen abstraction and, in particular, we were interested in application of AgSbF_6_, leveraging our group's recent expertise.^[^ ^50^ ^]^ Our initial attempt, deploying AgSbF_6_ at –78 °C, yielded 57% of the desired β,γ‐unsaturated ketone **3** in a 74:26 ratio of *Z*:*E* isomers. Encouraged by the initial results, we set out to improve the (*Z*)‐selectivity of this process. A key parameter for this system turned out to be the nature of the silane moiety (cf. Scheme 2b, *left*). Indeed, moving from trimethyl to triethyl and later trihexyl substitution resulted in improved *Z*:*E* ratios, with the trihexyl silyl group (**2ac**) affording the (*Z*)‐alkene product **3** as the only detectable isomer by NMR analysis. Although trihexylsilyl substitution provides the best results for this particular model system, we later found that a triethylsilyl group provides equally high *Z*:*E* ratios for a large range of other substrate combinations (e.g., product **5l** from silanes **2ba** and **2bb**, Scheme 2b, *bottom*). Interestingly, other silver salts and Lewis acids did not lead to any conversion due to solubility issues in dichloromethane at low reaction temperatures.

With optimized conditions in hand, we investigated the scope of this transformation (Scheme 3). At the outset, we tested various chlorides using triethylvinyl silane **2c** (Scheme 3, *top*). Various aromatic (**4a**–**4f**) and aliphatic acid chlorides (**4k**–**4v**) afforded the desired β,γ‐unsaturated ketones in good yields and with excellent *Z*:*E* ratios. This transformation proved to be scalable, as demonstrated by the formation of product **4a** (0.956 g of product, 74%, >95:5 *Z*:*E*) with no significant reduction in either the *Z*:*E* ratio or overall yield.

**Scheme 3:**
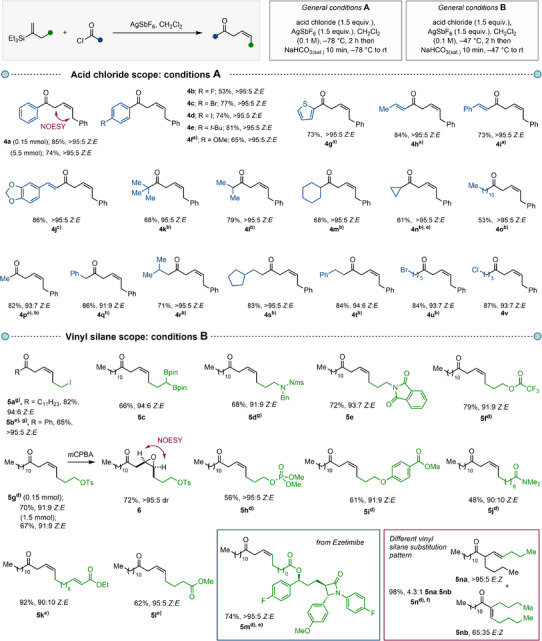
*Substrate scope*. All *Z*:*E* ratios were determined before column chromatography based on ^1^H NMR analysis of the crude reaction mixtures, unless otherwise stated. ^a)^ Reaction performed at –47 °C; ^b)^ 1.05 equiv. of acid chloride and 1.1 equiv. of AgSbF_6_ were used; ^c)^ reaction performed at 0 °C. ^d)^ a trihexylvinyl silane substrate was used; ^e)^ reaction performed at –61 °C; ^f)^ reaction performed at –78 °C; ^g)^ the *Z*:*E* ratio was determined after column chromatography—no partial isomer separation was detected; ^h)^ reaction performed with 2.0 equiv. of AgSbF_6_. Conditions for the epoxidation: *m*CPBA (2.0 equiv.), CH_2_Cl_2_, 0 °C to rt, 16 h.

In addition to benzoyl‐derived acid chlorides, 2‐thiophenecarbonyl chloride (product **4g**) proved to be a competent coupling partner in this olefination reaction. α,β‐Unsaturated acid chlorides, including cinnamoyl‐derived substrates, afforded the desired deconjugated enones (**4h**–**4j**) in excellent yields and with high (*Z*)‐selectivity. Likewise, our survey of aliphatic acid chlorides also resulted in consistently high yields and *Z*:*E* ratios for a variety of products (**4k**–**4v**), including those incorporating primary halides (**4u** and **4v**). In general, substrates carrying heteroatoms required only minor adjustments to the reaction conditions, typically involving elevated temperatures.

The scope of the vinyl silane coupling partner also proved to be broad, tolerating various functional groups at different positions relative to the olefin, with all products obtained in good yields and in ≥90:10 *Z*:*E* ratios (Scheme 3, *bottom*). β,γ‐Unsaturated (*Z*)‐enones possessing a primary halide (**5a** and **5b**), pinacol boronic esters (**5c**) and nitrogen‐containing functional groups, such as sulfonamide (**5d**) and phthalimide (**5e**), were prepared in good yields and with high (*Z*)‐selectivity. Remarkably, this methodology is tolerant of sensitive electrophiles such as a trifluoroacetate (**5f**), a primary tosylate (**5g**) and a phosphonate (**5** **h**) without decrease in (*Z*)‐selectivity. This is notable as these functional groups would likely pose challenges in the three‐step standard approach reliant on alkynyl anion intermediates, as described in Scheme 1b. In addition, deconjugated (*Z*)‐enones of various chain lengths possessing aryl ether (**5i**), amide (**5j**), α,β‐unsaturated ester (**5k**), and ester (**5l**) moieties were synthesized in good yields and selectivity from the corresponding vinyl silanes. While exploring the reaction scope, we observed that substrates with polar functionalities in proximity of the vinyl silane part typically afforded higher *Z*:*E* ratios, as demonstrated by products **5j** and **5l**. To demonstrate the utility of the β,γ‐unsaturated (*Z*)‐enones as valuable synthetic intermediates, we converted enone **5g** to *cis*‐epoxide **6**, isolated as a single diastereomer. The developed transformation was further validated using a derivative of the drug Ezetimibe, affording the corresponding product **5m** in excellent yield and *Z*:*E* ratio despite the complex array of functional groups present. Finally, we applied the developed method to access β,γ‐unsaturated ketones with α‐substitution, derived from internal alkenes. Interestingly, the reaction proceeded with the formation of an (*E*)‐configured deconjugated enone with excellent selectivity (**5n**), although we observed a decrease in regioselectivity in this case.

Encouraged by the broad substrate scope, we set out to explore contexts wherein this silicon‐based olefination strategy might considerably streamline overall synthetic pathways. In a first case study, we applied the developed method to the synthesis of the γ‐butyrolactone natural product **8** (Scheme 4a), first isolated from the cultures of the mushroom *T. pargamenum*.^[^ ^60^ ^]^ In the first synthesis, reported in 2014,^[^ ^61^ ^]^ this target was prepared using a lengthy 14‐step route featuring the classical epoxide/alkyne/Lindlar approach to install the required (*Z*)‐alkene (Scheme 4a, *top*). Our retrosynthetic analysis, in contrast, led us to vinyl silane **7**, which we prepared in four steps and 40% overall yield from pent‐4‐yn‐1‐ol, using the previously discussed ruthenium‐catalyzed hydrosilylation, followed by Krische's elegant stereoselective alkyne‐alcohol coupling^[^ ^62^ ^]^ (Scheme 4a, *middle*. See Supporting Information for details). The application of our conditions as a final step then afforded the required γ‐butyrolactone **8** in 57% yield with an excellent *Z*:*E* ratio and in 94:6 *er* (Scheme 4a, *bottom*). With this approach, we synthesized both enantiomers of this natural product in 23% and 33% yield over five steps. Furthermore, our route is amenable to rapid generation of analogs by changing the acid chloride coupling partner without a need for de novo synthesis. As a showcase, a reaction with benzoyl chloride proceeded with excellent *Z*:*E* ratio to generate the analog **9** in 47% yield.

**Scheme 4:**
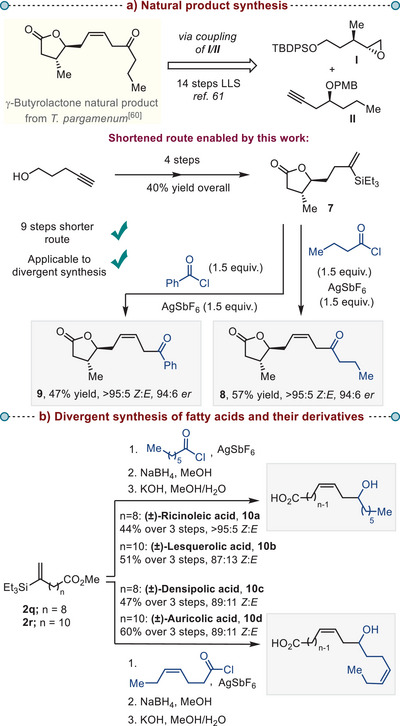
a) *Application of the developed method in the synthesis of a γ‐butyrolactone natural product*. b) *Application of the developed method in the divergent synthesis of fatty acids and their derivatives*. TBDPS—*tert*‐butyldiphenylsilyl; PMB—*p*‐methoxybenzyl.

In addition, we applied our olefination method to the synthesis of (±)‐Ricinoleic,^[^ ^63^ ^]^ (±)‐Lesquerolic,^[^ ^64^ ^]^ (±)‐Densipolic,^[^ ^65^ ^]^ and (±)‐Auricolic^[^ ^66^ ^]^ acids (**10a**–**10d**), valuable products carrying (*Z*)‐alkenes (Scheme 4b).^[^ ^67^ ^]^ The modular nature of our reaction allowed us to quickly access each of these compounds in only three steps by using suitable combinations of vinyl silanes an acid chlorides.

We then focused on gaining insight into the reaction mechanism and the origin of the observed (*Z*)‐selectivity. We hypothesized that the addition of Grignard reagents to the reaction mixture prior to work up could result in the capture of intermediate cationic species (Scheme 5a). Indeed, in the case of aromatic acid chlorides, we could isolate the tetrahydrofuran adducts, such as **11**, as single diastereomers (see the Supporting Information for additional examples of isolated tetrahydrofurans). Most importantly, these adducts were formed with *cis*‐configuration with respect to silyl, alkyl, and aryl groups, as determined by an X‐ray crystal structure of the tetrahydrofuran salt **12** (CCDC 2453341).^[^ ^68^ ^]^

**Scheme 5:**
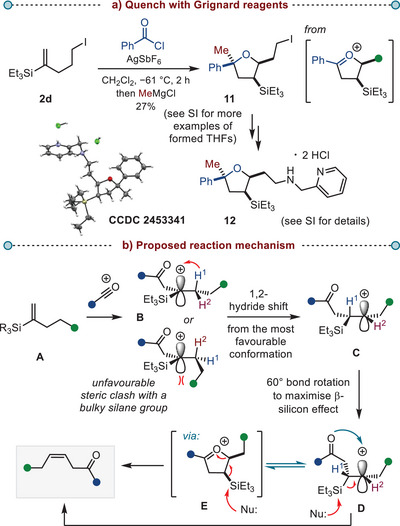
a) *Isolation of the tetrahydrofuran adduct as proof of the oxocarbenium intermediate*. b) *Proposed reaction mechanism*. THFs—tetrahydrofurans.

This observation, together with previous mechanistic studies of related charge relocation processes,^[^ ^69^ ^]^ led us to propose the following reaction pathway (Scheme 5b). The in situ formed acylium electrophile reacts with vinyl silane **A** at the terminal sp^2^‐hybridized carbon,^[^ ^52^ ^]^ forming carbocation **B**. A subsequent hydride shift via the conformation which avoids steric clash between the bulky silane group and the alkyl substituent generates carbocation **C**. While intermediate **C** is formally a β‐silyl carbocation, the relative orientations of silicon and the empty p‐orbital of the carbenium ion do not allow for optimal stabilization through hyperconjugation.^[^ ^35^ ^]^ Hence, a 60° bond rotation, following the principle of least motion, is necessary to bring the σ(C–Si) bond into coplanarity with the empty p‐orbital,^[^ ^53^ ^]^ thus allowing maximum stabilization through the β‐silicon effect in the conformer **D**. Depending on the reaction system, this intermediate can exist in equilibrium with a five‐membered oxocarbenium ion **E** with a *cis*‐arrangement of substituents. Finally, nucleophilic desilylation on either species leads to the (*Z*)‐alkene as the major product isomer.

In summary, we have developed a novel silicon‐based olefination to access highly valuable, yet challenging, β,γ‐(*Z*)‐unsaturated ketones. This reaction presents high diastereoselectivity, broad functional group tolerance and allows for a new type of a retrosynthetic disconnection, which can significantly shorten the synthesis of this type of alkenes. The synthetic utility of the method has been demonstrated by synthesizing five different natural products in a short and expeditious manner.

## Supporting Information

The authors have cited additional references within the Supporting Information.^[^ ^70^, ^71^, ^72^, ^73^, ^74^, ^75^, ^76^, ^77^, ^78^, ^79^, ^80^, ^81^ ^]^

## Conflict of Interests

The authors declare no conflict of interest.

## Supporting information



Supporting information

## Data Availability

The data that support the findings of this study are available in the Supporting Information of this article.
