# Supplementary material for: Facile (Z)‐Selective Synthesis of β,γ‐Unsaturated Ketones by a Silicon‐based Olefination Strategy
Source: Angew Chem Int Ed Engl. 2025 Oct 23;64(49):e202517069. doi: 10.1002/anie.202517069 (PMC12668309; doi:10.1002/anie.202517069)
Supplement: Supplementary file 1 — Supporting information [file ANIE-64-e202517069-s001.pdf]

Facile (*Z*)-Selective Synthesis of β,γ-Unsaturated Ketones by a Silicon-based Olefination Strategy

Daniya Aynetdinova‡ [a], Jakub Brześkiewicz‡[a], Nikolaos Skoulikas‡[a] and Nuno Maulide*[a]

[a] Institute of Organic Chemistry, University of Vienna, Währinger Straße 38, 1090 Vienna (Austria)
E-Mail: nuno.maulide@univie.ac.at, Homepage: http://maulide.univie.ac.at

Table of Contents

1. General Information 2
2. Optimization 3
3. Experimental Procedures and Characterization Data........ 7
3.1 Synthesis of Acyl Chlorides 7
3.2 Synthesis of Alkyne Substrates 7
3.3 Synthesis of Vinyl silanes........ 12
3.4 Synthesis of β,γ-Unsaturated Ketones 25
3.5 Unsuccessful and low-yielding substrates 49
4. Natural Products Synthesis 50
4.1 Fatty Acid Synthesis 50
4.2 Total Synthesis of the γ-butyrolactone natural product **8** 59
5. Mechanistic studies 69
5.1 Synthesis of THF derivative **12** for confirmation of formation of an oxocarbenium intermediate 69
5.2 Synthesis of additional THF derivatives 71
5.3 Comment on the desilylation step 73
6. Flow chart to help determining the optimum reaction temperature........ 74
7. NMR spectra 75
8. X-Ray Analysis 190
9. References 191

1. General Information

Unless otherwise stated, all glassware was flame-dried before use and all reactions were performed under an atmosphere of argon. All solvents were distilled from appropriate drying agents prior to use or directly taken from commercial sealed bottles under an atmosphere of argon. All reagents were used as received from commercial suppliers unless otherwise stated. Silver hexafluoroantimonate ($AgSbF_6$) was purchased from Sigma-Aldrich and stored in a glovebox under an atmosphere of argon. Reaction progress was monitored by thin layer chromatography (TLC) performed on aluminum plates coated with silica gel F254 with 0.2 mm thickness. Chromatograms were visualized by fluorescence quenching with UV light at 254 nm or by staining using potassium permanganate or phosphomolybdic acid, followed by heating. Flash column chromatography was performed using silica gel 60 (230-400 mesh, 0.040-0.063 mm Merck and co.) and silica gel RS SiOH (15-40 µm). Neat infrared spectra were recorded using a Perkin-Elmer Spectrum 100 FT-IR spectrometer. Wavenumbers (ν_{max}) are reported in cm^{-1}. Mass spectra were obtained on a Bruker maXis UHR-TOF (QQ-TOF) spectrometer, using electrospray ionization (ESI) or an Agilent 7200B GC/Q-TOF spectrometer, using electron ionization (EI). Optical rotations were measured on a Perkin Elmer 341 polarimeter using a 100 mm path-length cell at 589 nm (c given in g/100 mL). Chiral HPLC was performed using AGILENT Infinity 1260 with Chiralpak IH-3 column. Details on chromatographic conditions are indicated under each compound. All ^{1}H NMR, ^{13}C NMR, ^{31}P NMR, ^{29}Si NMR and ^{19}F NMR spectra were recorded using Bruker AV-400, AV-500, AV-600 or AV-700 spectrometers at 298 K, unless stated otherwise. Chemical shifts (δ) are given in parts per million (ppm) and referenced to the given solvent peak as described in literature.[70] Coupling constants are quoted in Hz (*J*). ^{1}H NMR splitting patterns are designated as singlet (s), doublet (d), triplet (t), quartet (q), pentet (p), nonet (n). ^{1}H NMR splitting patterns are reported as they appeared in the spectrum. Splitting patterns that could not be interpreted or easily visualized are designated as multiplet (m) or broad (br). The *Z*:*E* isomer ratio between each non-separable isomer mixture was determined based on the analysis of ^{1}H NMR spectra by integration of characteristic peaks.

2. Optimization

General optimization of the reaction conditions

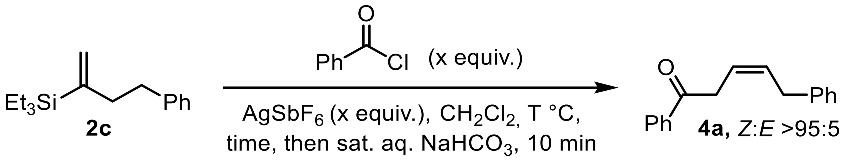

Entry	T	Equivalents	Time	Outcome
1[a]	35 °C	benzoyl chloride (1.05 equiv.), $AgSbF_6$ (1.1 equiv.)	30 min	complex RM, no SM
2[a]	0 °C	benzoyl chloride (1.05 equiv.), $AgSbF_6$ (1.1 equiv.)	30 min	complex RM, no SM
3[a]	–78 °C	benzoyl chloride (1.05 equiv.), $AgSbF_6$ (1.1 equiv.)	30 min	27% yield, >95:5 Z:E unreacted SM: 60%
4[a]	–78 °C	benzoyl chloride (1.05 equiv.), $AgSbF_6$ (1.1 equiv.)	2 h	47% yield, >95:5 Z:E unreacted SM: 43%
5[a]	–78 °C	benzoyl chloride (1.5 equiv.), $AgSbF_6$ (1.5 equiv.)	2 h	61% yield (67% NMR), >95:5 Z:E unreacted SM: 11%
**6[b]**	**–78 °C**	**benzoyl chloride (1.5 equiv.), $AgSbF_6$ (1.5 equiv.)**	**2 h**	**85% yield, >95:5 *Z:E***

Table **S1**. Isolated yields are reported, unless otherwise stated. All *Z:E* ratios were determined before column chromatography. [a)] Silver hexafluoroantimonate ($AgSbF_6$) was purchased from BLD Pharm, ABCR and Fluorochem. [b)] Silver hexafluoroantimonate ($AgSbF_6$) was purchased from Sigma-Aldrich. RM – reaction mixture; SM – starting material; NMR yields were determined using 1,1,2,2-tetrachloroethane as an internal standard.

Temperature variation

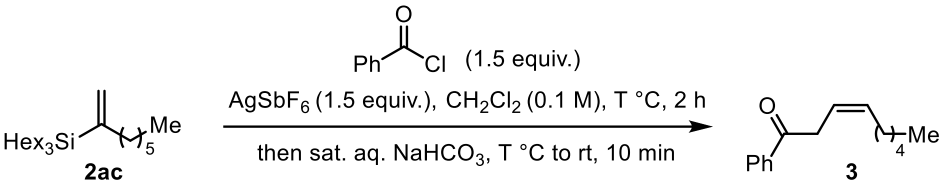

Entry	T, °C	Reaction yield, %	*Z:E*
**1**	**–78**	**75**	**>95:5**
2	–41	44 (NMR)	89:11
3	–20	28	55:45[a]

Table **S2**. Temperature dependance. Isolated yields are reported, unless otherwise stated. All *Z:E* ratios were determined before column chromatography, unless otherwise stated. [a] *Z:E* ratio was determined after purification by column chromatography.

Vinyl silane variation

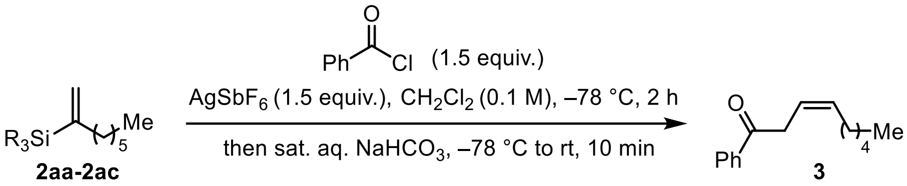

Entry	R_3Si	Reaction yield, %	*Z:E*
1	$SiMe_3$ (**2aa**)	57	74:26
2	$SiEt_3$ **(2ab)**	6	85:15
**3**	**$SiHex_3$ (2ac)**	**75**	**>95:5**

Table **S3**. Dependance on silicon substitution. Isolated yields are reported, unless otherwise stated. All *Z:E* ratios were determined before column chromatography, unless otherwise stated.

Silver salt variation

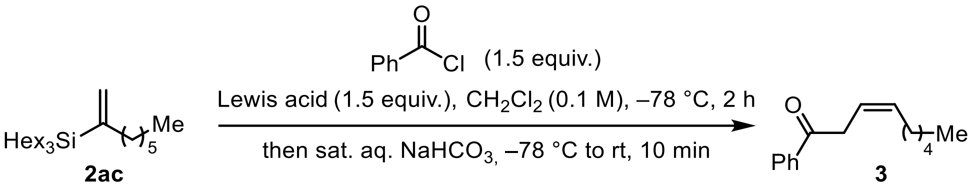

Entry	Lewis acid	Reaction yield, %	*Z:E*
**1**	**$AgSbF_6$**	**75**	**>95:5**
2	$AgBF_4$	0[a]	-
3	AgOTf	0[a]	-
4	Ag_2CO_3	0[a]	-
5	$AgNO_3$	0[a]	-
6	AgOTs	0[a]	-
7	$TiCl_4$	0[a]	-

Table **S4**. Lewis acid optimization. Isolated yields are reported, unless otherwise stated. All *Z:E* ratios were determined before column chromatography, unless otherwise stated.

Solvent variation

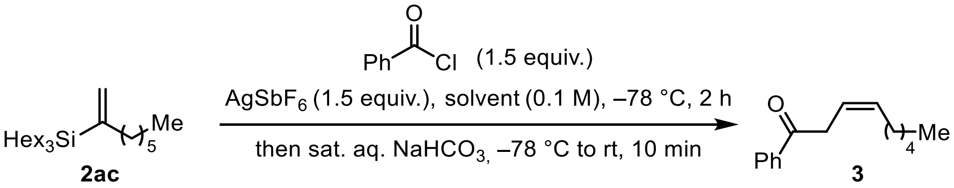

Entry	Solvent	Reaction yield, %	*Z:E*
**1**	**CH_2Cl_2**	**75**	**>95:5**
2	THF	0	-
3	Pentane	0	-
4	Acetone	0	-
5	AcOEt	0	-

Table **S5**. Solvent optimization. Isolated yields are reported, unless otherwise stated. All *Z:E* ratios were determined before column chromatography, unless otherwise stated.

Substrates with heteroatoms

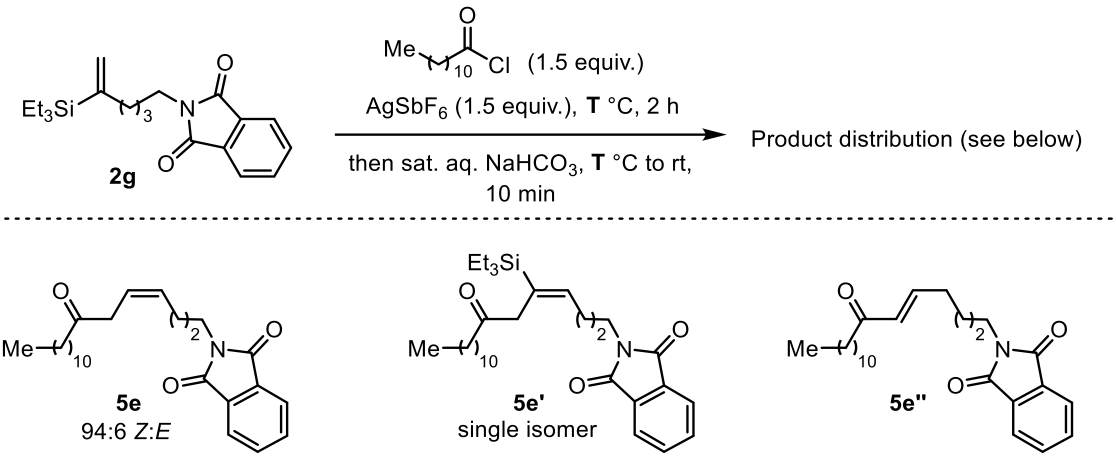

Entry	T, °C	Product 5e	*Z*:*E*	Product 5e' yield, %	Comment
**1**	–63 to –57	51% (NMR yield)	94:6	24% (NMR)	SM: <10% (NMR)
2	**–47**	**72%**	**93:7** (for **5e**)	**traces**	**5e'': <10% (NMR)**
3	–47 to –30	**5e** : **5e''** = 1 : 1	87:13 (for **5e**)	traces	formation of alkene isomers, e.g. **5e''**

Table **S6**. Example of the temperature optimization for a heteroatom substrate. All *Z:E* ratios were determined before column chromatography, unless otherwise stated. NMR yields were determined using 1,1,2,2-tetrachloroethane as an internal standard.

3. Experimental Procedures and Characterization Data

3.1 Synthesis of Acyl Chlorides

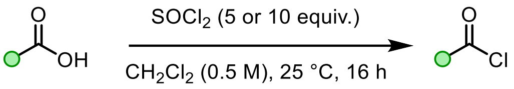

General procedure GP1: Synthesis of acid chlorides:

To a stirred solution of the carboxylic acid (1.0 equiv.) in anhydrous CH_2Cl_2 (0.5 M), thionyl chloride (5.0 or 10.0 equiv.) was added and the resulting mixture was stirred at ambient temperature (25 °C) for 14 h. Subsequently, the mixture was concentrated *in vacuo* affording the corresponding acyl chloride. The compound was used in the next step without further purification.

(*E*)-3-(Benzo[*d*][1,3]dioxol-5-yl)acryloyl chloride (S1)

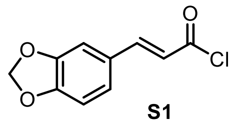
S1

Synthesized following general procedure **GP1**, using 3,4-methylenedioxycinnamic acid (192 mg, 1.00 mmol, 1.00 equiv.), thionyl chloride (360 µL, 5.00 mmol, 5.00 equiv.) and CH_2Cl_2 (2 mL). No analytical data were obtained for this compound, which was used without further purification.

(*Z*)-Hept-4-enoyl chloride (S2)

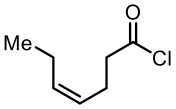

Synthesized following general procedure **GP1**, using (*Z*)-hept-4-enoic acid[71] (884 mg, 6.90 mmol, 1.00 equiv.), thionyl chloride (5.08 mL, 69.0 mmol, 10.0 equiv.) and CH_2Cl_2 (13.8 mL). No analytical data were obtained for this compound, which was used without further purification.

3.2 Synthesis of Alkyne Substrates

2,2'-(Hex-5-yne-1,1-diyl)bis(4,4,5,5-tetramethyl-1,3,2-dioxaborolane) (1a)

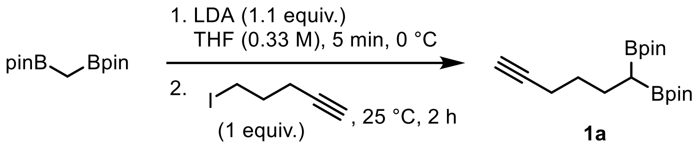

1a

To a flame-dried Schlenk flask equipped with a stir bar, bis[(pinacolato)boryl]methane (705 mg, 2.50 mmol, 1.00 equiv.) and anhydrous THF (7.5 mL) were added under an argon atmosphere. LDA (1 M in THF, 2.75 mL, 2.75 mmol, 1.10 equiv.) was added *via* syringe at 0 °C. The mixture was stirred for 5 min, then 5-iodopent-1-yne[72] (485 mg, 2.50 mmol, 1.00 equiv.) in anhydrous THF (2.5 mL) was added. The reaction mixture was allowed to reach room temperature (25 °C) while stirring for 2 h, then diluted with EtOAc (20 mL) and washed with a sat. aq. NH_4Cl (20 mL). The organic layer was dried over anhydrous Na_2SO_4, filtered and concentrated *in vacuo*. The crude residue was purified by flash column chromatography (silica gel, 5% EtOAc in heptane) to afford the desired compound as colorless oil (473 mg, 1.42 mmol, 57% yield).

^{1}H NMR (400 MHz, CDCl$_3$): δ 2.15 (td, *J* = 7.2, 2.7 Hz, 2H), 1.91 (t, *J* = 2.6 Hz, 1H), 1.67 – 1.59 (m, 2H), 1.58 – 1.48 (m, 2H), 1.24 – 1.20 (m, 24H), 0.72 (t, *J* = 7.6 Hz, 1H) ppm.

^{13}C NMR (101 MHz, CDCl$_3$): δ 85.1, 83.1 (4C), 68.0, 31.5, 25.3, 25.0 (4C), 24.9, 24.7 (4C), 18.7 ppm.

IR (neat) ν_{max}: 2977, 2933, 1361, 1309, 1267, 1137, 968, 849, 626 cm^{-1}.

HRMS (ESI$^+$): exact mass calculated for $[M+Na]^+$ $(C_{18}H_{32}{}^{11}B_2O_4{}^{23}Na)^+$ requires *m/z* 357.2380, found *m/z* 357.2379.

***N*-Benzyl-*N*-(hex-5-yn-1-yl)-2,4,6-tris(trifluoromethyl)benzenesulfonamide (1b)**

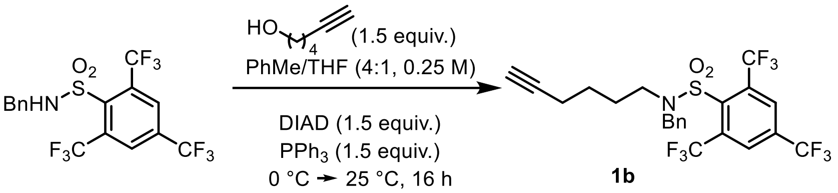

Under argon atmosphere, a flame dried Schlenk flask was charged with anhydrous solvents (toluene/THF 4:1, 3.5 mL) and cooled to 0 °C before adding the triphenylphosphine (489 mg, 1.86 mmol, 1.5 equiv.) and diethyl azodicarboxylate (DIAD, 0.367 mL, 1.86 mmol, 1.50 equiv.). After 15 min at 0 °C, the reaction mixture solidified and the mixture of 5-hexyn-1-ol (183 mg, 0.206 mL, 1.86 mmol, 1.50 equiv.) and *N*-benzyl-2,4,6-tris(trifluoromethyl)benzenesulfonamide[73] (561 mg, 1.24 mmol, 1.00 equiv.) in toluene/THF (4:1, 1.5 mL) was added via syringe. The resulting mixture became a clear solution and was stirred at room temperature (25 °C) for 16 h. After this time, the crude reaction mixture was concentrated *in vacuo* and the residue was purified by flash column chromatography (silica gel, 5% EtOAc in heptane) to afford the title product as a colorless oil (547 mg, 1.03 mmol, 83% yield).

^{1}H NMR (400 MHz, CDCl$_3$): δ 8.23 (s, 2H), 7.33 – 7.21 (m, 5H), 4.53 (s, 2H), 3.23 – 3.16 (m, 2H), 2.05 (td, *J* = 6.9, 2.6 Hz, 2H), 1.85 (t, *J* = 2.6 Hz, 1H), 1.53 – 1.45 (m, 2H), 1.37 – 1.24 (m, 2H) ppm.

^{13}C NMR (101 MHz, CDCl$_3$): δ 146.0, 135.1, 134.2 (q, *J* = 34.8 Hz, 2C), 133.2 (q, *J* = 33.8 Hz, 2C), 129.4 (m), 129.1 (2C), 128.8 (2C), 128.3, 122.2 (q, *J* = 275.7 Hz, 2C), 122.0 (q, *J* = 273.6 Hz), 83.5, 68.9, 53.1, 48.8, 26.6, 25.6, 18.0 ppm.

^{19}F NMR (376 MHz, CDCl$_3$): δ -54.97 (s, 6F), -63.60 (s, 3F) ppm.

IR (neat) ν_{max}: 1627, 1362, 1283, 1178, 1139, 1116, 1016, 914, 703 cm^{-1}.

HRMS (ESI$^+$): exact mass calculated for $[M+Na]^+$ $(C_{22}H_{18}F_9NO_2{}^{32}S^{23}Na)^+$ requires *m/z* 554.0807, found *m/z* 554.0810.

Ethyl (*E*)-tridec-2-en-12-ynoate (1c)

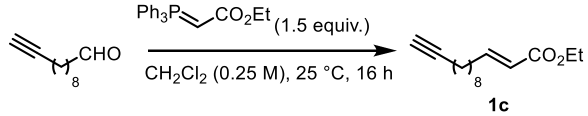

A 50 mL round-bottom flask was charged with a mixture of 10-undecyn-1-al[74] (482 mg, 2.90 mmol, 1.00 equiv.) and ethyl (triphenylphosphoranylidene)acetate (1.52 g, 4.35 mmol, 1.50 equiv.) in CH_2Cl_2 (11.6 mL) and was stirred at room temperature (25 °C) for 16 h. Then, the reaction mixture was concentrated *in vacuo* and was purified by flash column chromatography (silica gel, 2%-5% EtOAc in heptane) to afford the desired compound as colorless oil (680 mg, 2.88 mmol, 99% yield).

^{1}H NMR (400 MHz, $CDCl_3$): δ 6.94 (dt, *J* = 15.6, 7.0 Hz, 1H), 5.78 (dt, *J* = 15.6, 1.6 Hz, 1H), 4.16 (q, *J* = 7.1 Hz, 2H), 2.20 – 2.11 (m, 4H), 1.92 (t, *J* = 2.6 Hz, 1H), 1.55 – 1.22 (m, 15H) ppm.

^{13}C NMR (101 MHz, $CDCl_3$): δ 166.8, 149.5, 121.4, 84.8, 68.2, 60.2, 32.3, 29.3, 29.2, 29.1, 28.8, 28.5, 28.1, 18.5, 14.4 ppm.

IR (neat) ν_{max}: 2928, 1717, 1654, 1367, 1265, 1179, 1042, 979, 627 cm^{-1}.

HRMS (ESI$^+$): exact mass calculated for $[M+H]^+$ $(C_{16}H_{25}O_2)^+$ requires *m/z* 237.1849, found *m/z* 237.1847.

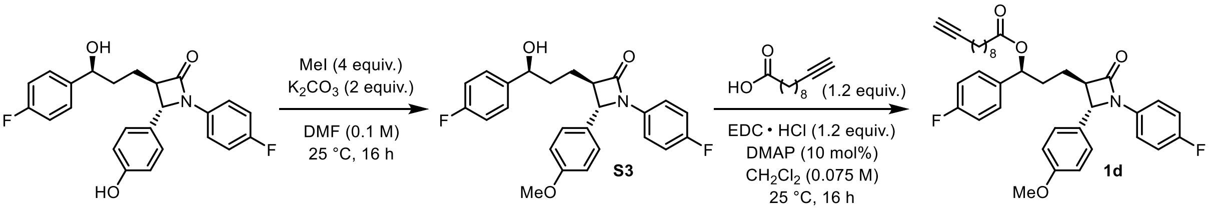

(3*R*,4*S*)-1-(4-Fluorophenyl)-3-((*S*)-3-(4-fluorophenyl)-3-hydroxypropyl)-4-(4-methoxyphenyl)azetidin-2-one (S3)

To ezetimibe (409 mg, 1.00 mmol, 1.00 equiv.) and K_2CO_3 (276 mg, 2.00 mmol, 2.00 equiv.) in anhydrous DMF (10 mL) was added methyl iodide (0.249 mL, 4.00 mmol, 4.00 equiv.). The reaction mixture was stirred at room temperature (25 °C) under an argon atmosphere. After 16 h, the reaction mixture was extracted with EtOAc (3 × 15 mL). The organic phase was washed with water (10 mL) andbrine (10 mL), dried over anhydrous Na_2SO_4, filtered and then concentrated *in vacuo*. The residue was purified by flash column chromatography (silica gel, 50% EtOAc in heptane) to afford the title product **S3** as a colorless oil (287 mg, 0.678 mmol, 68% yield).

^{1}H NMR (400 MHz, $CDCl_3$): δ 7.32 – 7.20 (m, 6H), 7.04 – 6.98 (m, 2H), 6.95 – 6.87 (m, 4H), 4.75 – 4.68 (m, 1H), 4.57 (d, *J* = 2.3 Hz, 1H), 3.80 (s, 3H), 3.11 – 3.04 (m, 1H), 2.28 (d, *J* = 3.4 Hz, 1H), 2.04 – 1.85 (m, 4H) ppm.

^{13}C NMR (101 MHz, CDCl$_3$): δ 167.8, 162.3 (d, *J* = 245.6 Hz), 160.0, 159.1 (d, *J* = 243.4 Hz), 140.2 (d, *J* = 3.1 Hz), 134.0 (d, *J* = 2.8 Hz), 129.5, 127.5 (d, *J* = 8.1 Hz, 2C), 127.3 (2C), 118.5 (d, *J* = 7.8 Hz, 2C), 116.0 (d, *J* = 22.6 Hz, 2C), 115.5 (d, *J* = 21.3 Hz, 2C), 114.8 (2C), 73.2, 61.3, 60.5, 55.5, 36.8, 25.2 ppm.

^{19}F NMR (376 MHz, CDCl$_3$): δ -114.83 – -114.97 (m, 1F), -118.04 – -118.17 (m, 1F) ppm.

IR (neat) ν_{max}: 3435, 2934, 1729, 1609, 1506, 1386, 1218, 831, 729 cm^{-1}.

HRMS (ESI$^+$): exact mass calculated for [M+Na]$^+$ ($C_{25}H_{23}F_2NO_3{}^{23}Na$)$^+$ requires *m/z* 446.1538, found *m/z* 446.1531.

$[\alpha]_D^{24}$ = -51.2 (*c* = 1.00, CHCl$_3$).

(*S*)-1-(4-Fluorophenyl)-3-((2*S*,3*R*)-1-(4-fluorophenyl)-2-(4-methoxyphenyl)-4-oxoazetidin-3-yl)propyl undec-10-ynoate (1d)

A round-bottomed flask was charged with the alcohol **S3** (255 mg, 0.602 mmol, 1.00 equiv.), EDC · HCl (139 mg, 0.723 mmol, 1.20 equiv.) and 4-dimethylaminopyridine (DMAP, 7.4 mg, 0.061 mmol, 10 mol%). Then, the combined solids were dissolved in anhydrous CH_2Cl_2 (3 mL) and stirred at room temperature (25 °C) for 15 min, before 10-undecynoic acid (132 mg, 0.724 mmol, 1.20 equiv.) dissolved in anhydrous CH_2Cl_2 (5 mL) was added via syringe. The reaction mixture was stirred at room temperature for 16 h. After this time, the reaction was quenched by the addition of sat. aq. $NaHCO_3$. The organic layers were washed with brine (10 mL), dried over anhydrous Na_2SO_4, filtered and then concentrated *in vacuo* to give the crude product, which was purified by flash column chromatography (silica gel, 20% EtOAc in heptane) to afford the desired product as a colorless oil (342 mg, 0.582 mmol, 97% yield).

^{1}H NMR (400 MHz, CDCl$_3$): δ 7.34 – 7.21 (m, 6H), 7.04 (t, *J* = 8.7 Hz, 2H), 6.99 – 6.90 (m, 4H), 5.74 (t, *J* = 6.7 Hz, 1H), 4.58 (d, *J* = 2.2 Hz, 1H), 3.84 (s, 3H), 3.10 (td, *J* = 7.7, 2.4 Hz, 1H), 2.33 (t, *J* = 7.2 Hz, 2H), 2.20 (td, *J* = 7.1, 2.7 Hz, 2H), 2.11 – 2.02 (m, 2H), 1.97 (t, *J* = 2.6 Hz, 1H), 1.93 – 1.84 (m, 2H), 1.67 – 1.49 (m, 4H), 1.44 – 1.25 (m, 8H) ppm.

^{13}C NMR (101 MHz, CDCl$_3$): δ 173.1, 167.2, 162.5 (d, *J* = 246.6 Hz), 160.0, 159.1 (d, *J* = 243.4 Hz), 136.1 (d, *J* = 3.3 Hz), 134.0 (d, *J* = 2.7 Hz), 129.4, 128.3 (d, *J* = 8.2 Hz, 2C), 127.3 (2C), 118.5 (d, *J* = 7.9 Hz, 2C), 115.9 (d, *J* = 22.6 Hz, 2C), 115.6 (d, *J* = 21.5 Hz, 2C), 114.8 (2C), 84.8, 74.7, 68.3, 61.1, 60.2, 55.5, 34.5, 33.8, 29.2, 29.1, 29.0, 28.8, 28.5, 25.01, 24.99, 18.5 ppm.

^{19}F NMR (376 MHz, CDCl$_3$): δ -113.88 – -113.98 (m, 1F), -118.03 – -118.19 (m, 1F) ppm.

IR (neat) ν_{max}: 2931, 2857, 1739, 1610, 1508, 1385, 1249, 832, 731 cm^{-1}.

HRMS (ESI$^+$): exact mass calculated for [M+Na]$^+$ ($C_{36}H_{39}F_2NO_4{}^{23}Na$)$^+$ requires *m/z* 610.2739, found *m/z* 610.2744.

$[\alpha]_D^{24}$ = -26.0 (*c* = 0.75, CHCl$_3$).

Methyl tridec-12-ynoate (1e)

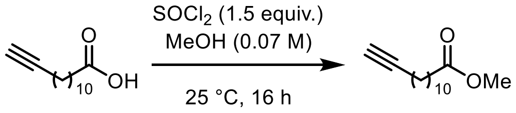

1e

Tridec-12-ynoic acid (600 mg, 2.85 mmol, 1.00 equiv.) was dissolved in MeOH (41 mL) and cooled to 0 °C. To the obtained solution, thionyl chloride (0.32 mL, 4.30 mmol, 1.50 equiv.) was added dropwise and the reaction mixture was allowed to slowly warm to room temperature (25 °C) over 16 h. The mixture was partitioned between CH_2Cl_2 (40 mL) and water (40 mL). The organic layer was separated, and the aqueous layer was extracted with CH_2Cl_2 (2 × 40 mL). The organic layers were combined, washed with brine (100 mL) and dried over anhydrous Na_2SO_4. After filtration, volatiles were removed *in vacuo* and the crude mixture was purified by flash column chromatography (silica gel, 0%-15% EtOAc in heptane) to obtain the desired product as a colorless oil (0.575 g, 2.56 mmol, 90% yield).

^{1}H NMR (400 MHz, $CDCl_3$): δ 3.67 (s, 3H), 2.30 (t, *J* = 7.5 Hz, 2H), 2.18 (td, *J* = 7.1, 2.6 Hz, 2H), 1.94 (t, *J* = 2.7 Hz, 1H), 1.67 – 1.57 (m, 2H), 1.53 – 1.47 (m, 2H), 1.44 – 1.23 (m, 12H) ppm.

^{13}C NMR (101 MHz, $CDCl_3$): δ 174.5, 84.9, 68.2, 51.6, 34.3, 29.6, 29.5, 29.4, 29.3, 29.2, 28.9, 28.6, 25.1, 18.5 ppm.

IR (neat) ν_{max}: 3309, 2927, 2855, 1739, 1436, 1362, 1197, 1172, 912, 734, 635 cm^{-1}.

HRMS (ESI$^+$): exact mass calculated for $[M+H]^+$ $(C_{14}H_{25}O_2)^+$ requires *m/z* 225.1849, found *m/z* 225.1854.

3.3 Synthesis of Vinyl silanes

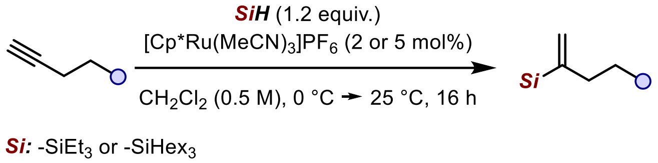

General procedure GP2: Synthesis of Vinyl Silanes Using [RuCp*(MeCN)$_3$]PF$_6$ Catalyst According to a Modified Method Reported by B. Trost *et al*:[59]

The corresponding alkyne (1.0 equiv.) was taken up in CH_2Cl_2 (0.5 M) at 0 °C under argon atmosphere, followed by slow addition of triethyl silane (1.2 equiv.) or trihexyl silane (1.2 equiv.). The solution was purged with argon for 10 min at 0 °C and then treated with [Cp*Ru(MeCN)$_3$]PF$_6$ (2 mol% or 5 mol%) at the same temperature. The reaction vessel was allowed to warm to room temperature (25 °C) and stirred for 16 h, after which time the alkyne was consumed, as determined by TLC analysis. The crude reaction mixture was concentrated *in vacuo*. The crude product was subjected to flash column chromatography (silica gel) using the appropriate mixture of eluents.

Trimethyl(oct-1-en-2-yl)silane (2aa)

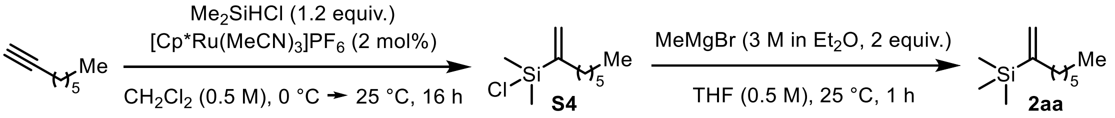

General procedure **GP2** was followed using 1-octyne (627 µL, 4.16 mmol, 1.00 equiv.), chlorodimethylsilane (555 µL, 5.00 mmol, 1.20 equiv.) and [Cp*Ru(MeCN)$_3$]PF$_6$ (42.1 mg, 0.080 mmol, 2 mol%). The resulting crude material was distilled under vacuum (85 °C, 14 mbar) to yield chlorodimethyl vinyl silane **S4** as a colorless oil (362 mg, 1.77 mmol, 43%). Due to its high reactivity, the product was not characterized and was directly used in the subsequent step.

To a solution of chlorodimethyl vinyl silane **S4** (100 mg, 0.490 mmol, 1.00 equiv.) in dry THF (1 mL), methylmagnesium bromide (3 M solution in Et_2O, 325 µL, 0.980 mmol, 2.00 equiv.) was added dropwise at room temperature (25 °C). The reaction mixture was stirred at the same temperature for 1 h. Afterwards, a sat. aq. NH_4Cl (2 mL) was added at 0 °C, and the mixture was transferred to a separatory funnel. The organic layer was diluted with diethyl ether (10 mL) and washed with sat. aq. NH_4Cl (2 × 10 mL) and with brine (10 mL). The organic phases were combined and dried over anhydrous Na_2SO_4. After filtration, the solvent was concentrated *in vacuo* to yield the title compound as a colorless oil (69.2 mg, 0.381 mmol, 77% yield).

^{1}H NMR (600 MHz, CDCl$_3$): δ 5.55 (dt, *J* = 3.1, 1.5 Hz, 1H), 5.30 (dt, *J* = 3.2, 1.0 Hz, 1H), 2.16 – 2.08 (m, 2H), 1.43 – 1.36 (m, 2H), 1.34 – 1.21 (m, 6H), 0.92 – 0.86 (m, 3H), 0.08 (s, 9H) ppm.

^{13}C NMR (151 MHz, CDCl$_3$): δ 152.8, 123.8, 36.4, 32.0, 29.24, 29.19, 22.8, 14.3, -1.3 (3C) ppm.

IR (neat) ν_{max}: 2956, 2926, 2856, 1459, 1248, 1058, 922, 834, 786, 756, 690 cm^{-1}.

HRMS: We were unable to obtain HRMS data for this compound, despite several attempts.

Triethyl(oct-1-en-2-yl)silane (2ab)

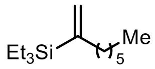

2ab

General procedure **GP2** was followed using 1-octyne (400 µL, 2.67 mmol, 1.00 equiv.), triethyl silane (509 µL, 1.20 mmol, 1.20 equiv.) and [Cp*Ru(MeCN)$_3$]PF$_6$ (27.4 mg, 0.050 mmol, 2 mol%). The resulting crude material was subjected to flash column chromatography (silica gel, 100% heptane) to obtain the title compound as a colorless oil (527 mg, 2.33 mmol, 88% yield).

^{1}H NMR (400 MHz, CDCl$_3$): δ 5.64 (dt, J = 3.13, 1.58 Hz, 1H), 5.34 – 5.25 (m, 1H), 2.14 – 2.00 (m, 2H), 1.47 – 1.37 (m, 2H), 1.36 – 1.24 (m, 6H), 1.01 – 0.85 (m, 12H), 0.69 – 0.48 (m, 6H) ppm.

^{13}C NMR (101 MHz, CDCl$_3$): δ 149.4, 125.1, 36.6, 32.1, 29.5, 29.1, 22.9, 14.3, 7.5 (3C), 3.2 (3C) ppm.

The analytical data is consistent with those previously reported in the literature.[75]

Trihexyl(oct-1-en-2-yl)silane (2ac)

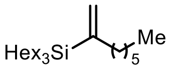

2ac

General procedure **GP2** was followed using 1-octyne (200 µL, 1.33 mmol, 1.00 equiv.), trihexyl silane (380 mg, 1.33 mmol, 1.00 equiv.) and [Cp*Ru(MeCN)$_3$]PF$_6$ (33.6 mg, 0.067 mmol, 5 mol%). The resulting crude material was subjected to flash column chromatography (silica gel, 100% heptane) to obtain the title compound as a colorless oil (466 mg, 1.22 mmol, 92% yield).

^{1}H NMR (600 MHz, CDCl$_3$): δ 5.60 (dt, J = 3.2, 1.6 Hz, 1H), 5.27 (d, J = 3.1 Hz, 1H), 2.10 – 2.03 (m, 2H), 1.39 (p, J = 7.7 Hz, 2H), 1.34 – 1.21 (m, 30H), 0.92 – 0.85 (m, 12H), 0.62 – 0.54 (m, 6H) ppm.

^{13}C NMR (151 MHz, CDCl$_3$): δ 150.2, 124.8, 36.4, 33.7 (3C), 32.0, 31.7 (3C), 29.4, 29.0, 23.9 (3C), 22.84, 22.80 (3C), 14.29 (3C), 14.27, 12.2 (3C) ppm.

IR (neat) ν_{max}: 2956, 2919, 2853, 2856, 1465, 1411, 1378, 1181, 1100, 960, 846, 719 cm^{-1}.

HRMS: We were unable to obtain HRMS data for this compound, despite several attempts.

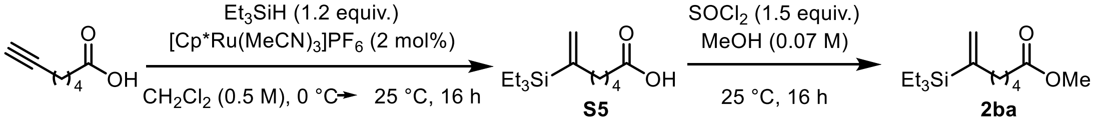

6-(Triethylsilyl)hept-6-enoic acid (S5)

General procedure **GP2** was followed using 6-heptynoic acid (242 mg, 1.92 mmol, 1.00 equiv.), triethyl silane (0.37 mL, 2.3 mmol, 1.20 equiv.) and [Cp*Ru(MeCN)$_3$]PF$_6$ (19.4 mg, 38.4 µmol, 2.0 mol%). The resulting crude

material was subjected to flash column chromatography (silica gel, 0%-30% EtOAc in heptane) to obtain the title compound as a colorless oil (390 mg, 1.61 mmol, 84% yield).

^{1}H NMR (400 MHz, CDCl$_3$): δ 10.60 – 9.28 (br, 1H), 5.63 (dt, J = 3.1, 1.6 Hz, 1H), 5.31 (dt, J = 2.6, 0.9 Hz, 1H), 2.37 (t, J = 7.5 Hz, 2H), 2.15 – 2.06 (m, 2H), 1.71 – 1.61 (m, 2H), 1.53 – 1.41 (m, 2H), 0.92 (t, J = 7.9 Hz, 9H), 0.64 – 0.55 (m, 6H) ppm.

^{13}C NMR (101 MHz, CDCl$_3$): δ 148.7, 125.5, 35.9, 33.8, 28.3, 24.7, 7.5 (3C), 3.1 (3C) ppm. *(One carbon peak could not be observed due to low intensity)*

IR (neat) ν$_{max}$: 2952, 2911, 2875, 1710, 1459, 1415, 1289, 1235, 1010, 922, 734 cm^{-1}.

HRMS (ESI$^-$): exact mass calculated for [M-H]$^-$ ($C_{13}H_{25}O_2{}^{28}Si$)$^-$ requires m/z 241.1629, found m/z 241.1624.

Methyl 6-(triethylsilyl)hept-6-enoate (2ba)

Vinyl silane **S5** (390 mg, 1.61 mmol, 1.00 equiv.) was dissolved in MeOH (23 mL) and cooled to 0 °C. To the obtained solution, thionyl chloride (0.18 mL, 2.40 mmol, 1.50 equiv.) was added dropwise and the reaction mixture was allowed to slowly warm to room temperature (25 °C) over 16 h. The mixture was partitioned between CH_2Cl_2 (20 mL) and water (20 mL). The organic layer was separated, and the aqueous layer was extracted with CH_2Cl_2 (2 × 20 mL). The organic layers were combined, washed with brine (50 mL) and dried over Na_2SO_4. After filtration, volatiles were removed *in vacuo* and the crude mixture was purified by flash column chromatography (silica gel, 0%-7% EtOAc in heptane) to obtain the desired product as a colorless oil (0.374 g, 1.46 mmol, 91%).

^{1}H NMR (400 MHz, CDCl$_3$): δ 5.62 (dt, J = 3.1, 1.6 Hz, 1H), 5.30 (dt, J = 2.9, 1.0 Hz, 1H), 3.67 (s, 3H), 2.32 (t, J = 7.5 Hz, 2H), 2.15 – 2.04 (m, 2H), 1.70 – 1.58 (m, 2H), 1.49 – 1.39 (m, 2H), 0.92 (t, J = 7.9 Hz, 9H), 0.66 – 0.53 (m, 6H) ppm.

^{13}C NMR (101 MHz, CDCl$_3$): δ 174.3, 148.7, 125.4, 51.6, 35.9, 34.2, 28.5, 25.0, 7.5 (3C), 3.1 (3C) ppm.

IR (neat) ν$_{max}$: 2951, 2875, 1741, 1436, 1366, 1230, 1217, 1172, 1007, 923, 720 cm^{-1}.

HRMS (ESI$^+$): exact mass calculated for [M+H]$^+$ ($C_{14}H_{29}O_2{}^{28}Si$)$^+$ requires m/z 257.1931, found m/z 257.1929.

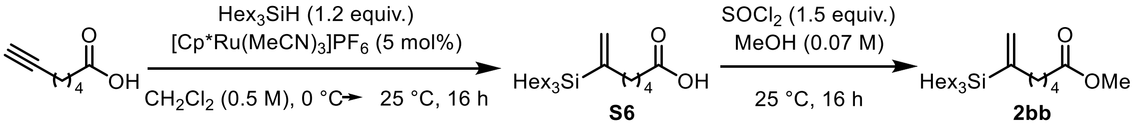

6-(Trihexylsilyl)hept-6-enoic acid (S6)

General procedure **GP2** was followed using 6-heptynoic acid (109 mg, 0.864 mmol, 1.00 equiv.), trihexyl silane (0.41 mL, 1.0 mmol, 1.20 equiv.) and [Cp*Ru(MeCN)$_3$]PF$_6$ (21.8 mg, 43.2 μmol, 5.0 mol%). The resulting crude material was subjected to flash column chromatography (silica gel, 0%-40% EtOAc in heptane) to obtain the title compound as a colorless oil (115 mg, 0.281 mmol, 33% yield).

^{1}H NMR (400 MHz, CDCl$_3$): δ 10.47 – 8.93 (br, 1H), 5.60 (dt, *J* = 3.0, 1.6 Hz, 1H), 5.33 – 5.25 (m, 1H), 2.37 (t, *J* = 7.5 Hz, 2H), 2.09 (t, *J* = 7.7 Hz, 2H), 1.71 – 1.59 (m, 2H), 1.52 – 1.41 (m, 2H), 1.35 – 1.20 (m, 24H), 0.93 – 0.84 (m, 9H), 0.63 – 0.52 (m, 6H) ppm.

^{13}C NMR (101 MHz, CDCl$_3$): δ 149.4, 125.1, 35.8, 33.7 (4C), 31.7 (3C), 28.3, 24.7, 23.9 (3C), 22.8 (3C), 14.3 (3C), 12.2 (3C) ppm. *(One carbon peak could not be observed due to low intensity)*

IR (neat) ν_{max}: 2956, 2920, 2854, 1711, 1458, 1413, 1378, 1288, 1239, 1183, 1101, 923, 742, 479 cm^{-1}.

HRMS (ESI$^-$): exact mass calculated for $[M-H]^-$ $(C_{25}H_{49}O_2{}^{28}Si)^-$ requires *m*/*z* 409.3507, found *m*/*z* 409.3515.

Methyl 6-(trihexylsilyl)hept-6-enoate (2bb)

Vinyl silane **S6** (115 mg, 0.280 mmol, 1.00 equiv.) was dissolved in MeOH (4 mL) and cooled to 0 °C. To the obtained solution, thionyl chloride (30 µL, 0.40 mmol, 1.50 equiv.) was added dropwise and the reaction mixture was allowed to slowly warm to room temperature (25 °C) over 16 h. The mixture was partitioned between CH_2Cl_2 (5 mL) and water (5 mL). The organic layer was separated, and the aqueous layer was extracted with CH_2Cl_2 (2 × 5 mL). The organic layers were combined, washed with brine (15 mL) and dried over Na_2SO_4. After filtration, volatiles were removed *in vacuo* and the crude mixture was purified by flash column chromatography (silica gel, 0%-7% EtOAc in heptane) to obtain the desired product as a colorless oil (124 mg, 0.292 mmol, quant).

^{1}H NMR (400 MHz, CDCl$_3$): δ 5.59 (dt, *J* = 3.1, 1.6 Hz, 1H), 5.33 – 5.23 (m, 1H), 3.67 (s, 3H), 2.32 (t, *J* = 7.5 Hz, 2H), 2.14 – 2.01 (m, 2H), 1.70 – 1.59 (m, 2H), 1.48 – 1.39 (m, 2H), 1.36 – 1.19 (m, 24H), 0.95 – 0.81 (m, 9H), 0.61 – 0.51 (m, 6H) ppm.

^{13}C NMR (101 MHz, CDCl$_3$): δ 174.3, 149.5, 125.1, 51.6, 35.8, 34.2, 33.7 (3C), 31.7 (3C), 28.4, 25.0, 23.9 (3C), 22.8 (3C), 14.3 (3C), 12.2 (3C) ppm.

IR (neat) ν_{max}: 2955, 2920, 2854, 1743, 1458, 1436, 1377, 1172, 1100, 996, 962, 922, 847, 742, 719 cm^{-1}.

HRMS (ESI$^+$): exact mass calculated for $[M+H]^+$ $(C_{26}H_{53}O_2{}^{28}Si)^+$ requires *m*/*z* 425.3809, found *m*/*z* 425.3816.

Triethyl(4-phenylbut-1-en-2-yl)silane (2c)

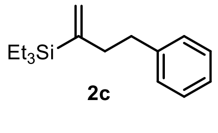

General procedure **GP2** was followed using but-3-yn-1-ylbenzene (0.14 mL, 1.00 mmol, 1.00 equiv.), triethyl silane (0.19 mL, 1.20 mmol, 1.20 equiv.) and $[Cp^*Ru(MeCN)_3]PF_6$ (10.2 mg, 0.020 mmol, 2 mol%). The resulting crude material was subjected to flash column chromatography (silica gel, 100% heptane) to obtain the title compound as a colorless oil (100 mg, 0.412 mmol, 41% yield).

^{1}H NMR (400 MHz, CDCl3): δ 7.32 – 7.11 (m, 13H), 5.72 (dt, *J* = 3.0, 1.6 Hz, 2H), 5.39 – 5.32 (m, 2H), 2.78 – 2.62 (m, 4H), 2.47 – 2.29 (m, 4H), 1.01 – 0.86 (m, 18H), 0.62 (dt, *J* = 18.8, 7.2 Hz, 12H) ppm.

^{13}C NMR (101 MHz, CDCl3): δ 148.8, 142.7, 128.51 (2C), 128.46 (2C), 125.9, 125.5, 38.1, 35.5, 7.5 (3C), 3.1 (3C) ppm.

The analytical data is consistent with those previously reported in the literature.[76]

Triethyl(5-iodopent-1-en-2-yl)silane (2d)

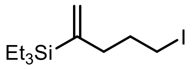

2d

General procedure **GP2** was followed using 5-iodopent-1-yne[72] (202 mg, 1.00 mmol, 1.00 equiv.), triethyl silane (0.19 mL, 1.2 mmol, 1.2 equiv.) and [Cp*Ru(MeCN)$_3$]PF$_6$ (10.1 mg, 20.0 μmol, 2.0 mol%). The resulting crude material was subjected to flash column chromatography (silica gel, 100% heptane) to obtain the title compound as a colorless oil (304 mg, 0.980 mmol, 98% yield).

^{1}H NMR (400 MHz, CDCl$_3$): δ 5.69 – 5.64 (m, 1H), 5.37 – 5.31 (m, 1H), 3.19 (t, *J* = 6.9 Hz, 2H), 2.24 – 2.14 (m, 2H), 1.94 (dd, *J* = 8.3, 6.3 Hz, 2H), 0.93 (t, *J* = 7.9 Hz, 9H), 0.61 (q, *J* = 8.1 Hz, 6H) ppm.

^{13}C NMR (101 MHz, CDCl$_3$): δ 147.3, 126.3, 36.9, 32.7, 7.5 (3C), 6.9, 3.0 (3C) ppm.

IR (neat) ν_{max}: 2952, 2908, 2874, 1456, 1417, 1233, 1004, 924, 717 cm^{-1}.

HRMS (ESI$^+$): exact mass calculated for [M+H]$^+$ ($C_{11}H_{24}{}^{127}I^{28}Si$)$^+$ requires *m/z* 311.0683, found *m/z* 311.0686.

(6,6-Bis(4,4,5,5-tetramethyl-1,3,2-dioxaborolan-2-yl)hex-1-en-2-yl)triethylsilane (2e)

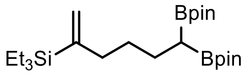

2e

General procedure **GP2** was followed using alkyne **1a** (244 mg, 0.70 mmol, 1.00 equiv.), triethyl silane (0.13 mL, 0.84 mmol, 1.20 equiv.) and [Cp*Ru(MeCN)$_3$]PF$_6$ (7.1 mg, 0.014 mmol, 2.0 mol%). The resulting crude material was subjected to flash column chromatography (silica gel, 0%-5% EtOAc in heptane) to obtain the title compound as a colorless oil (271 mg, 0.603 mmol, 86% yield).

^{1}H NMR (400 MHz, CDCl$_3$): δ 5.63 – 5.59 (m, 1H), 5.24 (d, *J* = 3.1 Hz, 1H), 2.09 – 2.02 (m, 2H), 1.56 (q, *J* = 7.7 Hz, 2H), 1.44 – 1.34 (m, 2H), 1.28 – 1.18 (m, 24H), 0.90 (t, *J* = 7.9 Hz, 9H), 0.74 (t, *J* = 7.8 Hz, 1H), 0.58 (q, *J* = 7.8 Hz, 6H) ppm.

^{13}C NMR (101 MHz, CDCl$_3$): δ 149.5, 124.9, 83.0 (4C), 36.6, 32.2, 26.1, 25.0 (4C), 24.7 (4C), 7.5 (3C), 3.1 (3C) ppm. (*The carbon attached to boron could not be observed due to quadrupolar relaxation)*

IR (neat) ν_{max}: 2977, 2952, 2875, 1461, 1308, 1267, 1138, 1005, 718 cm^{-1}.

HRMS (ESI$^+$): exact mass calculated for [M+Na]$^+$ ($C_{24}H_{48}{}^{11}B_2O_4{}^{28}Si^{23}Na$)$^+$ requires *m/z* 473.3405, found *m/z* 473.3410.

N-Benzyl-N-(5-(triethylsilyl)hex-5-en-1-yl)-2,4,6-tris(trifluoromethyl)benzenesulfonamide (2f)

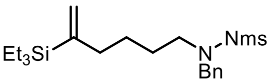

2f

General procedure **GP2** was followed using alkyne **1b** (521 mg, 0.98 mmol, 1.00 equiv.), triethyl silane (0.19 mL, 1.20 mmol, 1.20 equiv.) and [Cp*Ru(MeCN)$_3$]PF$_6$ (9.9 mg, 20 µmol, 2.0 mol%). The resulting crude material was subjected to flash column chromatography (silica gel, 0%-2% EtOAc in heptane) to obtain the title compound as a colorless oil (624 mg, 0.961 mmol, 98% yield).

^{1}H NMR (400 MHz, CDCl$_3$): δ 8.23 (s, 2H), 7.33 – 7.22 (m, 5H), 5.45 – 5.41 (m, 1H), 5.22 – 5.19 (m, 1H), 4.53 (s, 2H), 3.18 – 3.12 (m, 2H), 1.91 (tt, J = 7.7, 1.3 Hz, 2H), 1.41 – 1.31 (m, 2H), 1.24 – 1.13 (m, 2H), 0.87 (t, J = 7.9 Hz, 9H), 0.53 (q, J = 7.9 Hz, 6H) ppm.

^{13}C NMR (101 MHz, CDCl$_3$): δ 148.3, 146.1, 135.3, 134.1 (q, J = 35.3 Hz, 2C), 133.2 (q, J = 33.8 Hz, 2C), 129.4 (m), 129.0 (2C), 128.8 (2C), 128.3, 125.5, 122.2 (q, J = 275.7 Hz, 2C), 122.0 (q, J = 273.4 Hz), 53.1, 49.2, 35.6, 27.4, 25.8, 7.4 (3C), 3.0 (3C) ppm.

^{19}F NMR (376 MHz, CDCl$_3$): δ -54.97 (s, 6F), -63.61 (s, 3F) ppm.

IR (neat) ν$_{max}$: 2954, 2876, 1368, 1272, 1194, 1150, 909, 730, 702 cm^{-1}.

HRMS (ESI$^+$): exact mass calculated for [M+Na]$^+$ ($C_{28}H_{34}F_9NO_2{}^{32}S^{28}Si^{23}Na$)$^+$ requires m/z 670.1828, found m/z 670.1837.

2-(5-(Triethylsilyl)hex-5-en-1-yl)isoindoline-1,3-dione (2g)

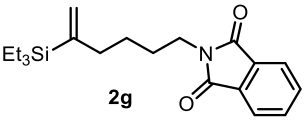

2g

General procedure **GP2** was followed using N-(5-hexynyl)phthalimide (225 mg, 0.991 mmol, 1.00 equiv.), triethyl silane (0.19 mL, 1.20 mmol, 1.20 equiv.) and [Cp*Ru(MeCN)$_3$]PF$_6$ (10.1 mg, 0.020 mmol, 2 mol%). The resulting crude material was subjected to flash column chromatography (silica gel, 0%-35% EtOAc in heptane) to obtain the title compound as a pale-yellow oil (274 mg, 0.798 mmol, 80% yield).

^{1}H NMR (400 MHz, CDCl$_3$): δ 7.89 – 7.80 (m, 2H), 7.76 – 7.65 (m, 2H), 5.62 (dt, J = 2.9, 1.5 Hz, 1H), 5.34 – 5.23 (m, 1H), 3.69 (t, J = 7.3 Hz, 2H), 2.14 – 2.07 (m, 2H), 1.77 – 1.62 (m, 2H), 1.51 – 1.42 (m, 2H), 0.90 (t, J = 8.0 Hz, 9H), 0.57 (q J = 7.7 Hz, 6H) ppm.

^{13}C NMR (101 MHz, CDCl$_3$): δ 168.6 (2C), 148.6 (2C), 134.0 (2C), 132.3, 125.6, 123.3 (2C), 38.1, 35.8, 28.6, 26.1, 7.5 (3C), 3.0 (3C) ppm.

IR (neat) ν$_{max}$: 2950, 2874, 1772, 1711, 1466, 1437, 1395, 1369, 1036, 924, 718 cm^{-1}.

HRMS (ESI$^+$): exact mass calculated for $[M+H]^+$ $(C_{20}H_{30}NO_2{}^{28}Si)^+$ requires *m/z* 344.2040, found *m/z* 344.2037.

5-(Trihexylsilyl)hex-5-en-1-ol (2h)

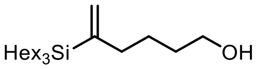

2h

General procedure **GP2** was followed using hex-5-yn-1-ol (117 mg, 1.19 mmol, 1.00 equiv.), trihexyl silane (0.51 mL, 1.40 mmol, 1.18 equiv.) and $[Cp^*Ru(MeCN)_3]PF_6$ (30.3 mg, 0.060 mmol, 5.0 mol%). The resulting crude material was subjected to flash column chromatography (silica gel, 0%-40% EtOAc in heptane) to obtain the title compound as a pale-yellow oil (238 mg, 0.622 mmol, 52% yield).

^{1}H NMR (400 MHz, CDCl$_3$): δ 5.61 (dt, *J* = 3.0, 1.5 Hz, 1H), 5.34 – 5.24 (m, 1H), 3.73 – 3.58 (m, 2H), 2.18 – 2.03 (m, 2H), 1.64 – 1.43 (m, 4H), 1.35 – 1.17 (m, 24H), 0.88 (t, *J* = 6.9 Hz, 9H), 0.67 – 0.50 (m, 6H) ppm. *(The signal for OH group could not be detected)*

^{13}C NMR (101 MHz, CDCl$_3$): δ 149.7, 125.0, 63.2, 35.9, 33.7 (3C), 32.8, 31.7 (3C), 25.0, 23.9 (3C), 22.8 (3C), 14.3 (3C), 12.2 (3C) ppm.

IR (neat) ν_{max}: 3329, 2956, 2920, 2855, 1459, 1378, 1180, 1062, 994, 922, 741, 531, 450 cm^{-1}.

HRMS (ESI$^+$): exact mass calculated for $[M+H]^+$ $(C_{24}H_{51}O^{28}Si)^+$ requires *m/z* 383.3704, found *m/z* 383.3708.

5-(Trihexylsilyl)hex-5-en-1-yl 2,2,2-trifluoroacetate (2i)

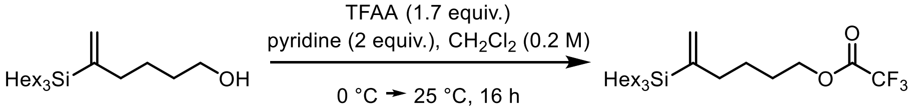

2h **2i**

5-(Trihexylsilyl)hex-5-en-1-ol **2h** (160 mg, 0.418 mmol, 1.00 equiv.) was dissolved in CH_2Cl_2 (2 mL) and cooled to 0 °C. To the obtained solution, pyridine (68 µL, 0.836 mmol, 2.00 equiv.) was added in one portion, followed by the dropwise addition of trifluoroacetic anhydride (0.10 mL, 0.711 mmol, 1.70 equiv.). The reaction mixture was allowed to slowly warm to room temperature (25 °C) and stirred for 16 h. The mixture was diluted with water (5 mL) and extracted with EtOAc (3 × 5 mL). The organic layers were combined, washed with 1 M HCl_{aq} (10 mL), sat. aq. $NaHCO_3$ (10 mL) and brine (15 mL). After filtration, volatiles were removed *in vacuo* and the crude mixture was purified by flash column chromatography (silica gel, 0%-5% EtOAc in heptane) to obtain the desired product as a colorless oil (173 mg, 0.361 mmol, 86% yield).

^{1}H NMR (400 MHz, CDCl$_3$): δ 5.60 (dt, *J* = 2.8, 1.4 Hz, 1H), 5.38 – 5.27 (m, 1H), 4.36 (t, *J* = 6.6 Hz, 2H), 2.12 (t, *J* = 7.7 Hz, 2H), 1.83 – 1.71 (m, 2H), 1.58 – 1.47 (m, 2H), 1.40 – 1.17 (m, 24H), 0.88 (t, *J* = 6.9 Hz, 9H), 0.67 – 0.51 (m, 6H).

^{13}C NMR (101 MHz, $CDCl_3$): δ 149.1, 125.5, 68.3, 35.6, 33.6 (3C), 31.7 (3C), 28.1, 24.8, 23.9 (3C), 22.8 (3C), 14.3 (3C), 12.1 (3C). *(Two carbon peaks could not be observed due to low intensity resulting from ^{13}C-^{19}F coupling)*

^{19}F NMR (376 MHz, $CDCl_3$): δ -75.11 (s, 3F).

IR (neat) ν_{max}: 2957, 2922, 2856, 2360, 2013, 1788, 1738, 1466, 1352, 1267, 1170, 1102, 742 cm^{-1}.

HRMS: We were unable to obtain HRMS data for this compound, despite several attempts.

5-(Trihexylsilyl)hex-5-en-1-yl 4-methylbenzenesulfonate (2j)

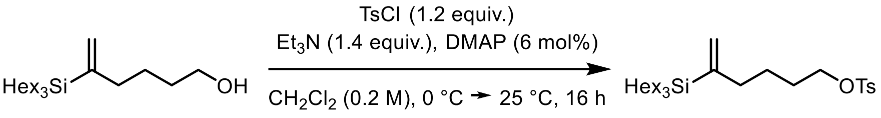

2h **2j**

To a stirred solution of 5-(trihexylsilyl)hex-5-en-1-ol **2h** (200 mg, 0.523 mmol, 1.00 equiv.), triethylamine (0.10 mL, 0.732 mmol, 1.40 equiv.) and 4-dimethylaminopyridine (3.8 mg, 31 µmol, 6.0 mol%) in CH_2Cl_2 (2.5 mL), *p*-toluenesulfonyl chloride (120 mg, 0.629 mmol, 1.20 equiv.) was added at 0 °C. Then, the cooling bath was removed and the mixture was allowed to warm to room temperature (25 °C) over 16 h. The reaction was quenched with sat. aq. NH_4Cl (1 mL) and water (5 mL). The mixture was extracted with CH_2Cl_2 (3 × 4 mL), the organic layers were combined, washed with brine (12 mL) and dried over anhydrous Na_2SO_4. After filtration, volatiles were removed *in vacuo* and the crude mixture was purified by flash column chromatography (silica gel, 0%-20% EtOAc in heptane) to obtain the desired product as a colorless oil (261 mg, 0.486 mmol, 93% yield).

^{1}H NMR (400 MHz, $CDCl_3$): δ 7.87 – 7.72 (m, 2H), 7.41 – 7.30 (m, 2H), 5.56 – 5.48 (m, 1H), 5.31 – 5.23 (m, 1H), 4.03 (t, *J* = 6.5 Hz, 2H), 2.45 (s, 3H), 2.02 (t, *J* = 7.6 Hz, 2H), 1.73 – 1.58 (m, 2H), 1.47 – 1.37 (m, 2H), 1.35 – 1.16 (m, 24H), 0.88 (t, *J* = 6.8 Hz, 9H), 0.62 – 0.46 (m, 6H) ppm.

^{13}C NMR (101 MHz, $CDCl_3$): δ 149.1, 144.7, 133.4, 129.9 (2C), 128.0 (2C), 125.2, 70.7, 35.4, 33.6 (3C), 31.7 (3C), 28.8, 24.5, 23.9 (3C), 22.8 (3C), 21.8, 14.3 (3C), 12.1 (3C) ppm.

IR (neat) ν_{max}: 2955, 2920, 2853, 1599, 1465, 1365, 1266, 1188, 1177, 1098, 933, 813, 743, 663, 554, 493 cm^{-1}.

HRMS (ESI$^+$): exact mass calculated for $[M+H]^+$ $(C_{31}H_{57}O_3{}^{32}S^{28}Si)^+$ requires *m/z* 537.3792, found *m/z* 537.3795.

Dimethyl (5-(trihexylsilyl)hex-5-en-1-yl) phosphate (2k)

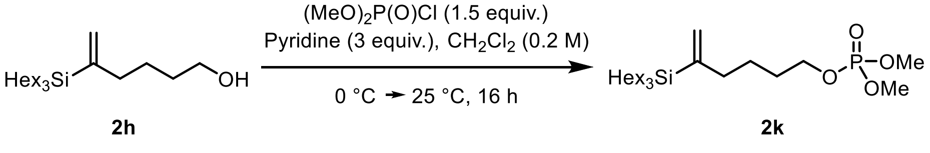

2h **2k**

Pyridine (45 µL, 0.56 mmol, 3.0 equiv.) was added to a solution of 5-(trihexylsilyl)hex-5-en-1-ol **2h** (70.8 mg, 0.185 mmol, 1.00 equiv.) and dimethyl chlorophosphate (30 µL, 0.28 mmol, 1.50 equiv.) in CH_2Cl_2 (2.5 mL) at 0 °C. The reaction mixture was allowed to slowly warm to room temperature (25 °C) over 16 h and then quenched

with sat. aq. NH_4Cl (1 mL). The mixture was partitioned between CH_2Cl_2 (5 mL) and water (5 mL) and the organic layer was separated. The aqueous layer was extracted with CH_2Cl_2 (2 × 5 mL), the organic layers were combined, washed with brine (15 mL) and dried over Na_2SO_4. After filtration, volatiles were removed *in vacuo* and the crude mixture was purified by flash column chromatography (silica gel, 50%-60% EtOAc in heptane) to obtain the desired product as a colorless oil (69.7 mg, 0.142 mmol, 77% yield).

^{1}H NMR (400 MHz, CDCl$_3$): δ 5.66 – 5.55 (m, 1H), 5.30 (d, *J* = 2.9 Hz, 1H), 4.06 (q, *J* = 6.7 Hz, 2H), 3.77 (d, *J* = 11.1 Hz, 6H), 2.15 – 2.00 (m, 2H), 1.78 – 1.61 (m, 2H), 1.55 – 1.45 (m, 2H), 1.37 – 1.14 (m, 24H), 0.88 (t, *J* = 6.9 Hz, 9H), 0.65 – 0.50 (m, 6H) ppm.

^{13}C NMR (101 MHz, CDCl$_3$): δ 149.3, 125.2, 68.03 (d, *J* = 5.9 Hz), 54.35 (d, *J* = 5.9 Hz, 2C), 35.6, 33.7 (3C), 31.7 (3C), 30.29 (d, *J* = 6.7 Hz), 24.6, 23.9 (3C), 22.8 (3C), 14.3 (3C), 12.1 (3C) ppm.

^{31}P NMR (162 MHz, CDCl$_3$): δ 1.40 (s).

IR (neat) ν_{max}: 2955, 2920, 2854, 1460, 1286, 1186, 1041, 912, 848, 737, 508 cm^{-1}.

HRMS (ESI$^+$): exact mass calculated for $[M+Na]^+$ ($C_{26}H_{55}O_4P^{28}Si^{23}Na)^+$ requires *m*/*z* 513.3499, found *m*/*z* 513.3498.

Methyl 4-((5-(trihexylsilyl)hex-5-en-1-yl)oxy)benzoate (2l)

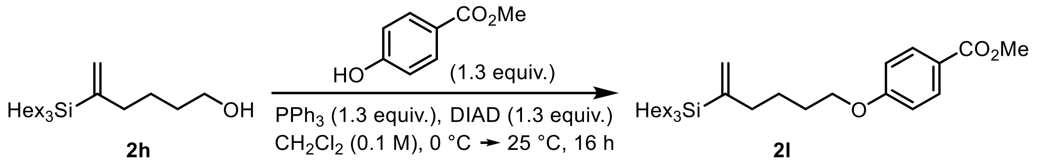

Triphenylphosphine (160 mg, 0.611 mmol, 1.30 equiv.) was added to a cooled (0 °C) solution of 5-(trihexylsilyl)hex-5-en-1-ol **2h** (180 mg, 0.470 mmol, 1.00 equiv.) and methyl 4-hydroxybenzoate (93 mg, 0.61 mmol, 1.3 equiv.) in CH_2Cl_2 (5 mL). Then diisopropyl azodicarboxylate (DIAD, 0.12 mL, 0.61 mmol, 1.30 equiv.) was added dropwise and the reaction mixture was slowly warmed to room temperature (25 °C) over 16 h and then diluted with CH_2Cl_2 (10 mL). The mixture was washed with water (10 mL), the organic phase was separated, and the aqueous phase was extracted with CH_2Cl_2 (2 × 10 mL). The organic layers were combined, washed with brine (30 mL) and dried over anhydrous Na_2SO_4. After filtration, volatiles were removed *in vacuo* and the crude mixture was purified by flash column chromatography (silica gel, 0%-10% EtOAc in heptane) to obtain the desired product as a colorless oil (170 mg, 0.329 mmol, 70% yield).

^{1}H NMR (400 MHz, CDCl$_3$): δ 8.04 – 7.91 (m, 2H), 6.95 – 6.84 (m, 2H), 5.67 – 5.57 (m, 1H), 5.31 (d, *J* = 2.9 Hz, 1H), 4.02 (t, *J* = 6.4 Hz, 2H), 3.88 (s, 3H), 2.23 – 2.08 (m, 2H), 1.87 – 1.75 (m, 2H), 1.64 – 1.56 (m, 2H), 1.35 – 1.18 (m, 24H), 0.87 (t, *J* = 6.8 Hz, 9H), 0.66 – 0.48 (m, 6H) ppm.

^{13}C NMR (101 MHz, CDCl$_3$): δ 167.1, 163.1, 149.5, 131.7 (2C), 125.2, 122.5, 114.2 (2C), 68.2, 52.0, 35.9, 33.7 (3C), 31.7 (3C), 29.1, 25.3, 23.9 (3C), 22.8 (3C), 14.3 (3C), 12.2 (3C) ppm.

IR (neat) ν_{max}: 2954, 2919, 2854, 1721, 1606, 1579, 1466, 1434, 1277, 1250, 1103, 921, 846, 769, 696, cm^{-1}.

HRMS (ESI$^+$): exact mass calculated for $[M+H]^+$ $(C_{32}H_{57}O_3{}^{28}Si)^+$ requires *m/z* 517.4072, found *m/z* 517.4077.

***N,N*-Dimethyl-10-(trihexylsilyl)undec-10-enamide (2m)**

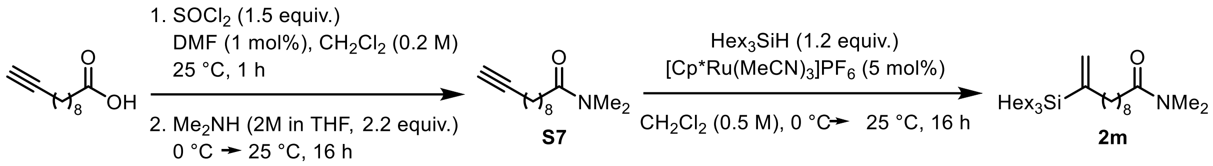

Undec-10-ynoic acid (3.21 g, 17.6 mmol, 1.00 equiv.) was dissolved in CH_2Cl_2 (88 mL). To the obtained solution, thionyl chloride (1.94 mL, 26.4 mmol, 1.50 equiv.) and dimethylformamide (14 µL, 0.18 mmol, 1.0 mol%) was added dropwise and the reaction mixture was stirred for 1 h at room temperature (25 °C). Subsequently, dimethylamine (2 M solution in THF, 19.3 mL, 38.7 mmol, 2.20 equiv.) was added at 0 °C and allowed to slowly warm to room temperature for 16 h. The mixture was partitioned between CH_2Cl_2 (100 mL) and water (100 mL). The organic layer was separated, and the aqueous layer was extracted with CH_2Cl_2 (2 × 50 mL). The organic layers were combined, washed with brine (100 mL) and dried over Na_2SO_4. After filtration, volatiles were removed *in vacuo* affording the crude product **S7** as a pale-yellow oil (3.545 g, 16.9 mmol, 96% yield) which was used directly in the next step without further purification.

General procedure **GP2** was followed using crude *N,N*-dimethylundec-10-ynamide **S7** (250 mg, 1.19 mmol, 1.00 equiv.), trihexyl silane (0.51 mL, 1.4 mmol, 1.2 equiv.) and $[Cp^*Ru(MeCN)_3]PF_6$ (30.3 mg, 0.060 mmol, 5.0 mol%). The resulting crude material was subjected to flash column chromatography (silica gel, 0%-50% EtOAc in heptane) to obtain the title compound as a colorless oil (151 mg, 0.31 mmol, 26% yield after two steps).

^{1}H NMR (400 MHz, CDCl$_3$): δ 5.66 – 5.51 (m, 1H), 5.30 – 5.22 (m, 1H), 3.00 (s, 3H), 2.94 (s, 3H), 2.35 – 2.25 (m, 2H), 2.11 – 1.99 (m, 2H), 1.69 – 1.58 (m, 2H), 1.42 – 1.19 (m, 34H), 0.88 (t, *J* = 6.9 Hz, 9H), 0.64 – 0.48 (m, 6H) ppm.

^{13}C NMR (101 MHz, CDCl$_3$): δ 173.4, 150.2, 124.7, 37.4, 36.4, 35.5, 33.7 (3C), 33.6, 31.7 (3C), 29.74, 29.71, 29.65 (2C), 29.0, 25.4, 23.9 (3C), 22.8 (3C), 14.3 (3C), 12.2 (3C) ppm.

IR (neat) ν_{max}: 2920, 2853, 1656, 1465, 1396, 1267, 1181, 1099, 960, 920, 742, 419 cm^{-1}.

HRMS (ESI$^+$): exact mass calculated for $[M+K]^+$ $(C_{31}H_{63}NO^{28}Si^{39}K)^+$ requires *m/z* 532.4311, found *m/z* 532.4308.

Ethyl (*E*)-12-(triethylsilyl)trideca-2,12-dienoate (2n)

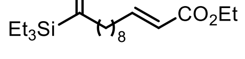

2n

General procedure **GP2** was followed using alkyne **1c** (246 mg, 1.00 mmol, 1.00 equiv.), triethyl silane (0.192 mL, 1.20 mmol, 1.20 equiv.) and $[Cp^*Ru(MeCN)_3]PF_6$ (10.1 mg, 0.020 mmol, 2.0 mol%). The resulting crude material was subjected to flash column chromatography (silica gel, 0%-2% EtOAc in heptane) to obtain the title compound as a colorless oil (339 mg, 0.961 mmol, 96% yield).

^{1}H NMR (400 MHz, CDCl$_3$): δ 6.96 (dt, *J* = 15.5, 7.0 Hz, 1H), 5.80 (dt, *J* = 15.6, 1.5 Hz, 1H), 5.62 (dt, *J* = 2.8, 1.3 Hz, 1H), 5.30 – 5.25 (m, 1H), 4.18 (q, *J* = 7.1 Hz, 2H), 2.23 – 2.14 (m, 2H), 2.11 – 2.03 (m, 2H), 1.49 – 1.23 (m, 15H), 0.92 (t, *J* = 7.9 Hz, 9H), 0.59 (q, *J* = 7.9 Hz, 6H) ppm.

^{13}C NMR (101 MHz, CDCl$_3$): δ 166.9, 149.6, 149.4, 125.1, 121.4, 60.2, 36.5, 32.3, 29.7, 29.6, 29.5, 29.3, 29.0, 28.2, 14.4, 7.5 (3C), 3.1 (3C) ppm.

IR (neat) ν_{max}: 2927, 2855, 1722, 1655, 1462, 1177, 1046, 921, 718 cm^{-1}.

HRMS (ESI$^+$): exact mass calculated for $[M+Na]^+$ $(C_{21}H_{40}O_2{}^{28}Si^{23}Na)^+$ requires *m/z* 375.2690, found *m/z* 375.2692.

(*S*)-1-(4-Fluorophenyl)-3-((2*S*,3*R*)-1-(4-fluorophenyl)-2-(4-methoxyphenyl)-4-oxoazetidin-3-yl)propyl 10-(trihexylsilyl)undec-10-enoate (2o)

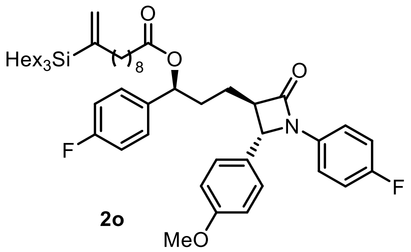

2o

General procedure **GP2** was followed using alkyne **1d** (140 mg, 0.24 mmol, 1.00 equiv.), trihexyl silane (0.10 mL, 0.29 mmol, 1.20 equiv.) and [Cp*Ru(MeCN)$_3$]PF$_6$ (2.4 mg, 4.8 µmol, 2.0 mol%). The resulting crude material was subjected to flash column chromatography (silica gel, 0%-40% EtOAc in heptane) to obtain the title compound as a colorless oil (205 mg, 0.24 mmol, 98% yield).

^{1}H NMR (400 MHz, CDCl$_3$): δ 7.30 – 7.18 (m, 6H), 7.01 (t, *J* = 8.6 Hz, 2H), 6.96 – 6.87 (m, 4H), 5.70 (t, *J* = 6.7 Hz, 1H), 5.61 – 5.56 (m, 1H), 5.27 (d, *J* = 3.1 Hz, 1H), 4.54 (d, *J* = 2.3 Hz, 1H), 3.80 (s, 3H), 3.06 (td, *J* = 7.7, 2.4 Hz, 1H), 2.29 (t, *J* = 7.4 Hz, 2H), 2.08 – 1.99 (m, 4H), 1.89 – 1.81 (m, 2H), 1.62 – 1.55 (m, 2H), 1.42 – 1.19 (m, 34H), 0.88 (t, *J* = 6.8 Hz, 9H), 0.61 – 0.53 (m, 6H) ppm.

^{13}C NMR (101 MHz, CDCl$_3$): δ 173.1, 167.2, 162.5 (d, *J* = 246.6 Hz), 160.0, 159.1 (d, *J* = 243.4 Hz), 150.1, 136.1 (d, *J* = 3.2 Hz), 134.0 (d, *J* = 2.8 Hz), 129.4, 128.3 (d, *J* = 8.2 Hz, 2C), 127.3 (2C), 124.8, 118.5 (d, *J* = 7.9 Hz, 2C), 115.9 (d, *J* = 22.6 Hz, 2C), 115.6 (d, *J* = 21.5 Hz, 2C), 114.8 (2C), 74.7, 61.1, 60.2, 55.5, 36.3, 34.6, 33.8, 33.7 (3C), 31.7 (3C), 29.7, 29.6, 29.4, 29.3, 29.0, 25.1, 25.0, 23.9 (3C), 22.8 (3C), 14.3 (3C), 12.2 (3C) ppm.

^{19}F NMR (376 MHz, CDCl$_3$): δ -113.84 – -113.99 (m, 1F), -118.04 – -118.16 (m, 1F) ppm.

IR (neat) ν_{max}: 2921, 2853, 1745, 1609, 1509, 1385, 1250, 832, 720 cm^{-1}.

HRMS (ESI$^+$): exact mass calculated for $[M+Na]^+$ $(C_{54}H_{79}F_2NO_4{}^{28}Si^{23}Na)^+$ requires *m/z* 894.5639, found *m/z* 894.5624.

$[\alpha]_D^{24}$ = -16.6 (*c* = 0.70, CHCl$_3$).

(*E*)-Dec-5-en-5-yltrihexylsilane (2p)

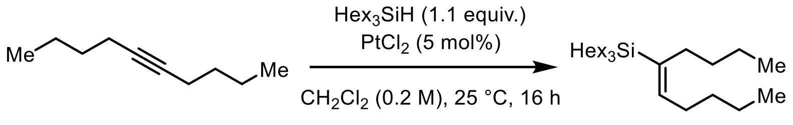

2p

To a solution of 5-decyne (0.36 mL, 2.00 mmol, 1.0 equiv.) and trihexylsilane (626 mg, 2.20 mmol, 1.10 equiv.) in CH_2Cl_2 (10 mL) was added $PtCl_2$ (26.6 mg, 0.100 mmol, 5.0 mol%) under an argon atmosphere. The reaction mixture was allowed to stir at room temperature (25 °C) for 16 h, and after completion of the reaction, was filtered through a small plug of silica gel and washed with Et_2O (30 mL). The solvent was removed *in vacuo*, and the resulting residue was purified by flash column chromatography (silica gel, 100% heptane) to afford the desired compound as colorless oil (768 mg, 1.82 mmol, 91% yield).

^{1}H NMR (600 MHz, $CDCl_3$): δ 5.65 (t, *J* = 6.9 Hz, 1H), 2.10 (q, *J* = 7.0 Hz, 2H), 2.07 – 2.03 (m, 2H), 1.38 – 1.21 (m, 32H), 0.94 – 0.86 (m, 15H), 0.57 – 0.52 (m, 6H) ppm.

^{13}C NMR (101 MHz, $CDCl_3$): δ 141.8, 138.2, 33.7 (3C), 32.7, 32.1, 31.7 (3C), 29.9, 28.3, 24.0 (3C), 23.4, 22.8 (3C), 22.6, 14.3 (3C), 14.22, 14.19, 12.5 (3C) ppm.

IR (neat) ν_{max}: 2956, 2919, 2855, 1465, 1378, 1181, 1100, 960, 713 cm^{-1}.

HRMS: We were unable to obtain HRMS data for this compound, despite several attempts.

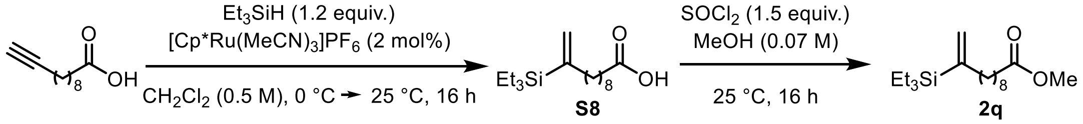

S8 **2q**

10-(Triethylsilyl)undec-10-enoic acid (S8)

General procedure **GP2** was followed using 10-undecynoic acid (350 mg, 1.92 mmol, 1.00 equiv.), triethyl silane (0.37 mL, 2.3 mmol, 1.20 equiv.) and [Cp*Ru(MeCN)$_3$]PF$_6$ (19.4 mg, 38.4 μmol, 2.0 mol%). The resulting crude material was subjected to flash column chromatography (silica gel, 0%-20% EtOAc in heptane) to obtain the title compound as a colorless oil (432 mg, 1.45 mmol, 75% yield).

^{1}H NMR (400 MHz, $CDCl_3$): δ 11.51 – 9.35 (br, 1H), 5.62 (dt, *J* = 3.1, 1.6 Hz, 1H), 5.28 (dt, *J* = 3.1, 1.0 Hz, 1H), 2.35 (t, *J* = 7.5 Hz, 2H), 2.13 – 1.99 (m, 2H), 1.71 – 1.58 (m, 2H), 1.46 – 1.24 (m, 10H), 0.92 (t, *J* = 7.9 Hz, 9H), 0.66 – 0.60 (q, *J* = 7.9 Hz, 6H) ppm.

^{13}C NMR (101 MHz, $CDCl_3$): δ 149.4, 125.1, 36.5, 33.9, 29.7, 29.5, 29.4, 29.2, 29.0, 24.8, 7.5 (3C), 3.1 (3C) ppm. *(One carbon peak could not be observed due to low intensity)*

IR (neat) ν_{max}: 3016, 2970, 2929, 1739, 1436, 1366, 1229, 1217, 722, 527 cm^{-1}.

HRMS (ESI$^-$): exact mass calculated for [M-H]$^-$ ($C_{17}H_{33}O_2{}^{28}Si$)$^-$ requires *m/z* 297.2255, found *m/z* 297.2254.

Methyl 10-(triethylsilyl)undec-10-enoate (2q)

Vinyl silane **S8** (173 mg, 0.579 mmol, 1.00 equiv.) was dissolved in MeOH (8.3 mL) and cooled to 0 °C. To the obtained solution, thionyl chloride (64 µL, 0.87 mmol, 1.50 equiv.) was added dropwise and the reaction mixture was allowed to slowly warm to room temperature (25 °C) over 16 h. The mixture was partitioned between CH_2Cl_2 (10 mL) and water (10 mL). The organic layer was separated, and the aqueous layer was extracted with CH_2Cl_2 (2 × 10 mL). The organic layers were combined, washed with brine (30 mL) and dried over Na_2SO_4. After filtration, volatiles were removed *in vacuo* and the crude mixture was purified by flash column chromatography (silica gel, 0%-10% EtOAc in heptane) to obtain the desired product as a colorless oil (151 mg, 0.482 mmol, 83%).

^{1}H NMR (400 MHz, $CDCl_3$): δ 5.62 (dt, *J* = 3.1, 1.6 Hz, 1H), 5.29 – 5.25 (m, 1H), 3.67 (s, 3H), 2.30 (t, *J* = 7.6 Hz, 2H), 2.11 – 2.01 (m, 2H), 1.68 – 1.56 (m, 2H), 1.43 – 1.20 (m, 10H), 0.92 (t, *J* = 7.9 Hz, 9H), 0.65 – 0.54 (m, 6H) ppm.

^{13}C NMR (101 MHz, $CDCl_3$): δ 174.5, 149.4, 125.1, 51.6, 36.5, 34.3, 29.7, 29.5, 29.4, 29.3, 29.0, 25.1, 7.5 (3C), 3.1 (3C) ppm.

IR (neat) ν_{max}: 2928, 2874, 2855, 1743, 1436, 1366, 1230, 1217, 1171, 1123, 1009, 921, 721, 670 cm^{-1}.

HRMS (ESI$^+$): exact mass calculated for $[M+H]^+$ $(C_{18}H_{37}O_2{}^{28}Si)^+$ requires *m/z* 313.2557, found *m/z* 313.2559.

Methyl 12-(triethylsilyl)tridec-12-enoate (2r)

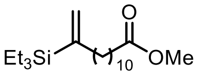

2r

General procedure **GP2** was followed using alkyne **1e** (573 mg, 2.55 mmol, 1.00 equiv.), triethyl silane (0.49 mL, 3.1 mmol, 1.20 equiv.) and $[Cp^*Ru(MeCN)_3]PF_6$ (25.8 mg, 51.1 µmol, 2.0 mol%). The resulting crude material was subjected to flash column chromatography (silica gel, 0%-7% EtOAc in heptane) to obtain the title compound as a colorless oil (819 mg, 2.40 mmol, 94% yield).

^{1}H NMR (400 MHz, $CDCl_3$): δ 5.62 (dt, *J* = 3.1, 1.6 Hz, 1H), 5.28 (dt, *J* = 3.1, 1.0 Hz, 1H), 3.67 (s, 3H), 2.30 (t, *J* = 7.5 Hz, 2H), 2.11 – 2.02 (m, 2H), 1.68 – 1.56 (m, 2H), 1.45 – 1.22 (m, 14H), 0.92 (t, *J* = 7.9 Hz, 9H), 0.65 – 0.55 (m, 6H) ppm.

^{13}C NMR (101 MHz, $CDCl_3$): δ 174.5, 149.4, 125.1, 51.6, 36.5, 34.3, 29.74, 29.72, 29.69, 29.6, 29.4, 29.3, 29.1, 25.1, 7.5 (3C), 3.1 (3C) ppm.

IR (neat) ν_{max}: 2925, 2874, 2854, 1742, 1459, 1435, 1362, 1196, 1170, 1007, 921, 719 cm^{-1}.

HRMS (ESI$^+$): exact mass calculated for $[M+H]^+$ $(C_{20}H_{41}O_2Si)^+$ requires *m/z* 341.2870, found *m/z* 341.2877.

3.4 Synthesis of β,γ-Unsaturated Ketones

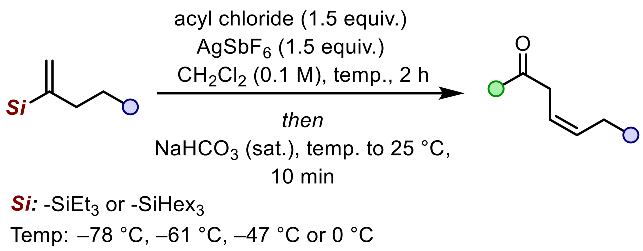

General procedure GP3: Synthesis of β,γ-Unsaturated Enones *via* Charge Relocation:

A flame-dried Schlenk tube or oven-dried vial was charged with a solution of vinyl silane (1.0 equiv.) in anhydrous CH_2Cl_2 (0.2 M) at room temperature (25 °C). To the solution, the required acid chloride (1.0–1.5 equiv.) was added in one portion at the same temperature. The solution was placed in an appropriate cooling bath at –78 °C (acetone/dry ice cooling-bath), –61 °C (chloroform/dry ice cooling bath), –47 °C (acetonitrile/dry ice cooling bath) or 0 °C (ice/water cooling bath) and allowed to stir for 1 min. In a separate vial, silver hexafluoroantimonate ($AgSbF_6$, 1.0–2.0 equiv.) was dissolved in CH_2Cl_2 (equal volume to the vinyl silane solution) at room temperature, and the obtained solution was then added dropwise to the pre-cooled vinyl silane and acyl chloride mixture to obtain an overall 0.1 M solution of the vinyl silane. The reaction mixture was stirred at the corresponding temperature for 2 h and then quenched with sat. aq. $NaHCO_3$ (equal volume to CH_2Cl_2) at the same temperature (at –78 °C, –61 °C, –47 °C or 0 °C respectively, please see the specific experimental procedures). The vial was immediately removed from the cooling bath and allowed to warm to room temperature over 5-10 min with vigorous stirring. The reaction mixture was partitioned between CH_2Cl_2 and water, and the organic layer was separated. The aqueous layer was extracted with CH_2Cl_2 (2 × 20 mL), the organic layers were combined, washed with brine and dried over anhydrous Na_2SO_4. After filtration, volatiles were removed *in vacuo*, and the crude mixture was purified by flash column chromatography (silica gel) using the appropriate mixture of eluents.

Note: For scale-up experiments (5.5 mmol scale for **4a** and 1.5 mmol scale for **5g**), after the addition of sat. aq. $NaHCO_3$ and warming to room temperature with vigorous stirring, the suspension was filtered through celite (washing with CH_2Cl_2).[1] The filtrate was collected and subjected to the aqueous work-up procedure as explained in General procedure **GP3**.

[1] Generally, upon the addition of a solution of $AgSbF_6$ in CH_2Cl_2 to the pre-cooled solution of a vinyl silane and an acid chloride in CH_2Cl_2 at the corresponding reaction temperature, a precipitate was observed which was attributed to AgCl formation. Therefore, on larger scales, following quenching with saturated aqueous $NaHCO_3$ and warming to room temperature, the mixture was filtered through celite to remove solids, thereby facilitating subsequent work-up.

Representative experimental set-up for the synthesis of 4a (0.2 mmol scale).

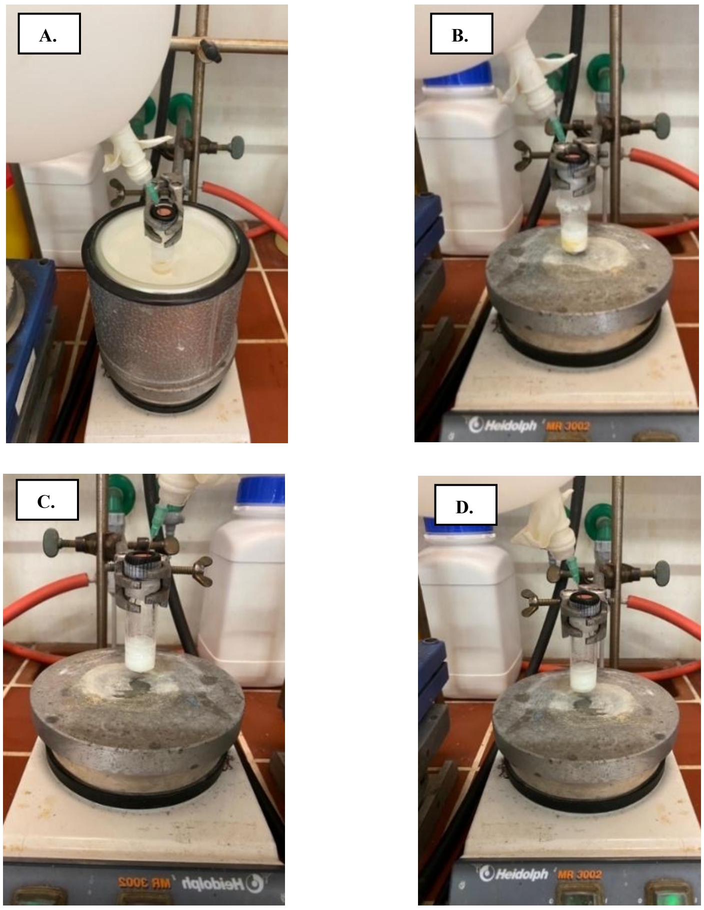

A. Reaction set-up (synthesis of **4a**) at –78 °C (acetone, dry ice).
B. Reaction appearance after quenching at –78 °C followed by immediate removal from the cooling bath.
C. Reaction appearance 5 min after quench with vigorous stirring while warming to room temperature.
D. Reaction appearance 7 min after quench with vigorous stirring while warming to room temperature.

Note:

Upon the addition of a saturated aqueous solution of $NaHCO_3$ (equal volume to CH_2Cl_2) at –78 °C (or –61 °C, or –47 °C depending on the specific reaction) and the subsequent removal from the cooling bath, the aqueous layer appears frozen (Photo **B**). Upon vigorous stirring and gradual warming to room temperature over 10 min, the aqueous layer melts (Photos **C** and **D**).

In the case of **4a**, transferring the reaction mixture from the –78 °C cooling bath to a 0 °C cooling bath (ice/water) and stirring at this temperature for 15 min prior to the addition of $NaHCO_3$ at 0 °C led to nonspecific decomposition. Consequently, all reactions were quenched at their respective reaction temperatures.

Furthermore, we found that quenching the reactions with $NaHCO_3$ provided the highest yields; quenching with 1M HCl was found to lead to irreproducible results.

(*Z*)-1-Phenylnon-3-en-1-one (3)

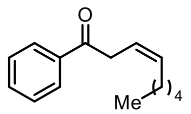

3

General procedure **GP3** was followed using vinyl silane **2ac** (57 mg, 0.15 mmol, 1.0 equiv.), benzoyl chloride (26 µL, 0.23 mmol, 1.5 equiv.) and silver hexafluoroantimonate (77.3 mg, 0.225 mmol, 1.50 equiv.) at –78 °C. The resulting crude material was subjected to flash column chromatography (silica gel, 0%-10% Et_2O in heptane) to obtain the title compound as a colorless oil (21.8 mg, 0.101 mmol, 67% yield). The product was obtained as a mixture of *Z*/*E* isomers in a ratio of >95:5 (determined based on the ^{1}H NMR of the crude reaction mixture).

^{1}H NMR (600 MHz, $CDCl_3$): δ 7.99 – 7.96 (m, 2H), 7.58 – 7.54 (m, 1H), 7.49 – 7.43 (m, 2H), 5.72 – 5.60 (m, 2H), 3.76 – 3.73 (m, 2H), 2.10 (q, *J* = 7.3 Hz, 2H), 1.39 (p, *J* = 7.3 Hz, 2H), 1.35 – 1.24 (m, 4H), 0.89 (t, *J* = 7.0 Hz, 3H) ppm.

^{13}C NMR (151 MHz, $CDCl_3$): δ 198.4, 136.9, 133.8, 133.2, 128.7 (2C), 128.4 (2C), 121.3, 37.6, 31.7, 29.2, 27.8, 22.7, 14.2 ppm.

IR (neat) ν_{max}: 2955, 2928, 2859, 1685, 1622, 1449, 1273, 1212, 1178, 1002, 974, 755, 689, 662 cm^{-1}.

HRMS (ESI$^+$): exact mass calculated for $[M+H]^+$ $(C_{15}H_{21}O)^+$ requires *m*/*z* 217.1592, found *m*/*z* 217.1587.

(*Z*)-1,5-Diphenylpent-3-en-1-one (4a)

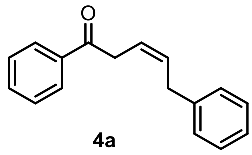

4a

General procedure **GP3** was followed using vinyl silane **2c** (37.0 mg, 0.150 mmol, 1.00 equiv.), benzoyl chloride (26 µL, 0.230 mmol, 1.50 equiv.) and silver hexafluoroantimonate (77.3 mg, 0.225 mmol, 1.50 equiv.) at –78 °C. The resulting crude material was subjected to flash column chromatography (silica gel, 0%-15% EtOAc in heptane) to obtain the title compound as a pale-yellow oil (30.1 mg, 0.127 mmol, 85% yield). The product was obtained as a mixture of *Z*/*E* isomers in a ratio of >95:5 (determined based on the ^{1}H NMR of the crude reaction mixture).

^{1}H NMR (400 MHz, $CDCl_3$): δ 8.03 – 7.94 (m, 2H), 7.62 – 7.54 (m, 1H), 7.50 – 7.43 (m, 2H), 7.33 – 7.16 (m, 5H), 5.92 – 5.80 (m, 2H), 3.86 (d, *J* = 5.5 Hz, 2H), 3.49 (d, *J* = 5.8 Hz, 2H) ppm.

^{13}C NMR (101 MHz, $CDCl_3$): δ 198.0, 140.3, 136.8, 133.3, 131.7, 128.8 (2C), 128.7 (2C), 128.5 (2C), 128.4 (2C), 126.3, 122.6, 37.5, 34.0 ppm.

HRMS (ESI$^+$): exact mass calculated for $[M+Na]^+$ $(C_{17}H_{16}O^{23}Na)^+$ requires *m*/*z* 259.1093, found *m*/*z* 259.1093.

IR (neat) ν_{max}: 3061, 3027, 1686, 1598, 1581, 1494, 1449, 1391, 1275, 1209, 1180, 1008, 911, 738, 692 cm^{-1}.

Scale-up procedure for (*Z*)-1,5-Diphenylpent-3-en-1-one (4a)

A flame-dried Schlenk tube was charged with a solution of vinyl silane (1.36 g, 5.50 mmol, 1.00 equiv.) in anhydrous CH_2Cl_2 (27.5 mL) at room temperature (25 °C). To the solution, benzoyl chloride (0.96 mL, 8.3 mmol, 1.5 equiv.) was added in one portion at the same temperature. The reaction vessel was placed in a cooling bath at –78 °C (acetone/dry ice cooling-bath) and allowed to stir for 1 min. In a separate flask, silver hexafluoroantimonate (2.84 g, 8.25 mmol, 1.50 equiv.) was dissolved in CH_2Cl_2 (27.5 mL) at room temperature under argon, and the obtained solution was then added dropwise to the pre-cooled vinyl silane and acyl chloride mixture. The reaction mixture was stirred at –78 °C for 2 h and then quenched with sat. aq. $NaHCO_3$ (55 mL) at the same temperature. The flask was immediately removed from the cooling bath and allowed to warm to room temperature over 20 min with vigorous stirring. The reaction mixture was filtered through celite, washing with CH_2Cl_2 (30 mL). The filtrate was transferred to a separatory funnel and the organic layer was separated. The aqueous layer was further extracted with CH_2Cl_2 (2 × 30 mL), the organic layers were combined, washed with brine and dried over anhydrous Na_2SO_4. After filtration, volatiles were removed *in vacuo*, and the crude mixture was purified by flash column chromatography (silica gel, 0%-10% EtOAc in heptane) to obtain the title compound as a pale-yellow oil (0.956 g, 4.05 mmol, 74% yield). The product was obtained as a mixture of *Z*/*E* isomers in a ratio of >95:5 (determined based on the ^{1}H NMR of the crude reaction mixture).

The analytical data is consistent with those obtained for this compound on 0.15 mmol scale (see page S27).

(*Z*)-1-(4-Fluorophenyl)-5-phenylpent-3-en-1-one (4b)

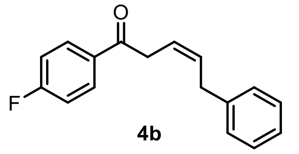

4b

General procedure **GP3** was followed using vinyl silane **2c** (37.0 mg, 0.150 mmol, 1.00 equiv.), 4-fluorobenzoyl chloride (26.6 µL, 0.225 mmol, 1.50 equiv.) and silver hexafluoroantimonate (77.3 mg, 0.225 mmol, 1.50 equiv.) at –78 °C. The resulting crude material was subjected to flash column chromatography (silica gel, 0%-10% Et_2O in heptane) to obtain the title compound as a colorless oil (20.3 mg, 0.080 mmol, 53% yield). The product was obtained as a mixture of *Z*/*E* isomers in a ratio of >95:5 (determined based on the ^{1}H NMR of the crude reaction mixture).

^{1}H NMR (400 MHz, $CDCl_3$): δ 8.06 – 7.92 (m, 2H), 7.33 – 7.28 (m, 2H), 7.24 – 7.06 (m, 5H), 5.91 – 5.81 (m, 2H), 3.87 – 3.79 (m, 2H), 3.53 – 3.45 (m, 2H) ppm.

^{13}C NMR (151 MHz, $CDCl_3$): δ 196.4, 165.9 (d, *J* = 255.0 Hz), 140.1, 133.2 (d, *J* = 3.2 Hz), 131.8, 131.1 (d, *J* = 9.3 Hz, 2C), 128.7 (2C), 128.5 (2C), 126.3, 122.4, 115.9 (d, *J* = 21.8 Hz, 2C), 37.5, 33.9 ppm.

^{19}F NMR (376 MHz, $CDCl_3$): δ -105.05 (s, 1F) ppm.

IR (neat) ν_{max}: 3062, 3027, 2916, 1684, 1596, 1495, 1453, 1298, 1277, 1229, 1155, 1009, 738, 699, 595 cm^{-1}.

HRMS (ESI$^+$): exact mass calculated for $[M+Na]^+$ $(C_{17}H_{15}FO^{23}Na)^+$ requires *m*/*z* 277.1005, found *m*/*z* 277.0999.

(*Z*)-1-(4-Bromophenyl)-5-phenylpent-3-en-1-one (4c)

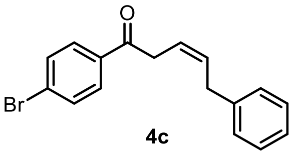

4c

General procedure **GP3** was followed using vinyl silane **2c** (37.0 mg, 0.150 mmol, 1.00 equiv.), 4-bromobenzoyl chloride (49.4 mg, 0.225 mmol, 1.50 equiv.) and silver hexafluoroantimonate (77.3 mg, 0.225 mmol, 1.50 equiv.) at –78 °C. The resulting crude material was subjected to flash column chromatography (silica gel, 0%-10% Et_2O in heptane) to obtain the title compound as a colorless oil (36.4 mg, 0.115 mmol, 77% yield). The product was obtained as a mixture of *Z*/*E* isomers in a ratio of >95:5 (determined based on the ^{1}H NMR of the crude reaction mixture).

^{1}H NMR (600 MHz, $CDCl_3$): δ 7.87 – 7.80 (m, 2H), 7.65 – 7.57 (m, 2H), 7.30 (dd, *J* = 8.6, 6.6 Hz, 2H), 7.24 – 7.17 (m, 3H), 5.92 – 5.80 (m, 2H), 3.85 – 3.79 (m, 2H), 3.48 (d, *J* = 6.1 Hz, 2H) ppm.

^{13}C NMR (151 MHz, $CDCl_3$): δ 196.9, 140.1, 135.5, 132.1 (2C), 132.0, 129.9 (2C), 128.7 (2C), 128.5 (3C), 126.3, 122.2, 37.5, 34.0 ppm.

IR (neat) ν_{max}: 3026, 1684, 1583, 1494, 1452, 1395, 1324, 1272. 1204, 1176, 1069, 1005, 735, 696, 625 cm^{-1}.

HRMS (ESI$^+$): exact mass calculated for $[M+Na]^+$ $(C_{17}H_{15}{}^{79}BrO^{23}Na)^+$ requires *m*/*z* 337.0204, found *m*/*z* 337.0198.

(*Z*)-1-(4-Iodophenyl)-5-phenylpent-3-en-1-one (4d)

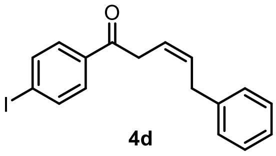

4d

General procedure **GP3** was followed using vinyl silane **2c** (37.0 mg, 0.150 mmol, 1.00 equiv.), 4-iodobenzoyl chloride (60.0 mg, 0.225 mmol, 1.50 equiv.) and silver hexafluoroantimonate (77.3 mg, 0.225 mmol, 1.5 equiv.) at –78 °C. The resulting crude material was subjected to flash column chromatography (silica gel, 0%-10% Et_2O in heptane) to obtain the title compound as a colorless solid (40.2 mg, 0.112 mmol, 74% yield). The product was obtained as a mixture of *Z*/*E* isomers in a ratio of >95:5 (determined based on the ^{1}H NMR of the crude reaction mixture).

^{1}H NMR (700 MHz, $CDCl_3$): δ 7.85 – 7.79 (m, 2H), 7.70 – 7.65 (m, 2H), 7.30 (tt, *J* = 7.8, 1.8 Hz, 2H), 7.24 – 7.17 (m, 3H), 5.90 – 5.81 (m, 2H), 3.81 (dt, *J* = 6.3, 1.1 Hz, 2H), 3.48 (d, *J* = 6.4 Hz, 2H) ppm.

^{13}C NMR (176 MHz, $CDCl_3$): δ 197.2, 140.1, 138.1 (2C), 136.0, 132.0, 129.8 (2C), 128.7 (2C), 128.5 (2C), 126.3, 122.2, 101.3, 37.4, 33.9 ppm.

IR (neat) ν_{max}: 3025, 1725, 1683, 1579, 1494, 1452, 1390, 1323, 1324, 1274, 1207, 1178, 1057, 1000, 737 cm^{-1}.

HRMS (ESI$^+$): exact mass calculated for $[M+Na]^+$ $(C_{17}H_{15}{}^{127}IO^{23}Na)^+$ requires *m*/*z* 385.0065, found *m*/*z* 385.0060.

(*Z*)-1-(4-(*tert*-Butyl)phenyl)-5-phenylpent-3-en-1-one (4e)

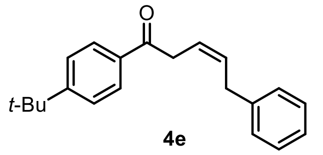

4e

General procedure **GP3** was followed using vinyl silane **2c** (37.0 mg, 0.150 mmol, 1.00 equiv.), 4-*tert*-butylbenzoyl chloride (43.9 µL, 0.225 mmol, 1.50 equiv.) and silver hexafluoroantimonate (77.3 mg, 0.225 mmol, 1.50 equiv.) at –78 °C. The resulting crude material was subjected to flash column chromatography (silica gel, 0%-10% Et_2O in heptane) to obtain the title compound as a colorless oil (35.6 mg, 0.122 mmol, 81% yield). The product was obtained as a mixture of *Z*/*E* isomers in a ratio of >95:5 (determined based on the ^{1}H NMR of the crude reaction mixture).

^{1}H NMR (700 MHz, $CDCl_3$): δ 8.09 – 7.90 (m, 2H), 7.55 – 7.46 (m, 2H), 7.32 – 7.26 (m, 2H), 7.24 – 7.16 (m, 3H), 5.92 – 5.78 (m, 2H), 3.88 – 3.78 (m, 2H), 3.49 (d, *J* = 6.8 Hz, 2H), 1.35 (s, 9H) ppm.

^{13}C NMR (176 MHz, $CDCl_3$): δ 197.7, 157.1, 140.3, 134.3, 131.6, 128.7 (2C), 128.5 (2C), 128.4 (2C), 126.2, 125.7 (2C), 122.9, 37.5, 35.3, 34.0, 31.2 (3C) ppm.

IR (neat) ν_{max}: 3028, 2969, 1738, 1682, 1605, 1494, 1366, 1109, 1004, 764, 745 cm^{-1}.

HRMS (ESI$^+$): exact mass calculated for $[M+Na]^+$ $(C_{21}H_{24}O^{23}Na)^+$ requires *m*/*z* 315.1725, found *m*/*z* 315.1719.

(*Z*)-1-(4-Methoxyphenyl)-5-phenylpent-3-en-1-one (4f)

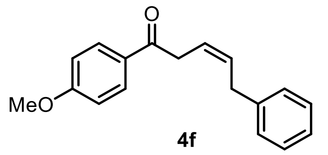

4f

General procedure **GP3** was followed using vinyl silane **2c** (37.0 mg, 0.150 mmol, 1.00 equiv.), 4-methoxybenzoyl chloride (30.5 µL, 0.225 mmol, 1.50 equiv.) and silver hexafluoroantimonate (77.3 mg, 0.225 mmol, 1.5 equiv.) at –47 °C. The resulting crude material was subjected to flash column chromatography (silica gel, 0%-10% Et_2O in heptane) to obtain the title compound as a colorless oil (26.1 mg, 0.098 mmol, 65% yield). The product was obtained as a mixture of *Z*/*E* isomers in a ratio of >95:5 (determined based on the ^{1}H NMR of the crude reaction mixture).

^{1}H NMR (700 MHz, $CDCl_3$): δ 7.99 – 7.93 (m, 2H), 7.30 (td, *J* = 7.2, 1.5 Hz, 2H), 7.24 – 7.17 (m, 3H), 6.97 – 6.91 (m, 2H), 5.90 – 5.80 (m, 2H), 3.87 (s, 3H), 3.80 (dq, *J* = 6.8, 1.0 Hz, 2H), 3.49 (d, *J* = 6.7 Hz, 2H) ppm.

^{13}C NMR (176 MHz, $CDCl_3$): δ 196.6, 163.7, 140.4, 131.5, 130.73 (2C), 129.9, 128.7 (2C), 128.5 (2C), 126.2, 123.0, 113.9 (2C), 55.6, 37.3, 34.0 ppm.

IR (neat) ν_{max}: 2970, 1738, 1677, 1600, 1510, 1454, 1366, 1260, 1216, 1170, 1030, 834, 743, 701 cm^{-1}

HRMS (ESI$^+$): exact mass calculated for $[M+Na]^+$ $(C_{18}H_{18}O_2{}^{23}Na)^+$ requires *m*/*z* 289.1204, found *m*/*z* 289.1199.

(*Z*)-5-Phenyl-1-(thiophen-2-yl)pent-3-en-1-one (4g)

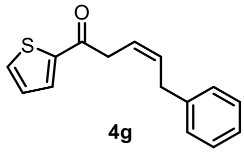

4g

General procedure **GP3** was followed using vinyl silane **2c** (37.0 mg, 0.150 mmol, 1.00 equiv.), 2-thenoyl chloride (24.3 µL, 0.225 mmol, 1.50 equiv.) and silver hexafluoroantimonate (77.3 mg, 0.225 mmol, 1.50 equiv.) at –47 °C. The resulting crude material was subjected to flash column chromatography (silica gel, 0%-10% Et_2O in heptane) to obtain the title compound as a colorless solid (26.5 mg, 0.110 mmol, 73% yield). The product was obtained as a mixture of *Z*/*E* isomers in a ratio of >95:5 (determined based on the ^{1}H NMR of the crude reaction mixture).

^{1}H NMR (400 MHz, $CDCl_3$): δ 7.74 (dd, *J* = 3.8, 1.2 Hz, 1H), 7.65 (dd, *J* = 4.9, 1.2 Hz, 1H), 7.35 – 7.26 (m, 2H), 7.22 (dd, *J* = 7.4, 1.5 Hz, 3H), 7.14 (dd, *J* = 5.0, 3.8 Hz, 1H), 5.92 – 5.78 (m, 2H), 3.79 (d, *J* = 4.7 Hz, 2H), 3.50 (d, *J* = 5.0 Hz, 2H) ppm.

^{13}C NMR (176 MHz, $CDCl_3$): δ 190.8, 144.0, 140.2, 133.9, 132.3, 131.9, 128.7 (2C), 128.5 (2C), 128.3, 126.3, 122.4, 38.3, 34.0 ppm.

IR (neat) ν_{max}: 3084, 3025, 2910, 1660, 1601, 1517, 1494, 1415, 1391, 1234, 1214, 858, 827, 770, 727 cm^{-1}.

HRMS (ESI$^+$): exact mass calculated for $[M+Na]^+$ $(C_{15}H_{14}O^{32}S^{23}Na)^+$ requires *m/z* 265.0663, found *m/z* 265.0658.

(2*E*,6*Z*)-8-Phenylocta-2,6-dien-4-one (4h)

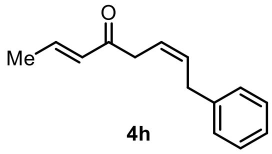

4h

General procedure **GP3** was followed using vinyl silane **2c** (37.0 mg, 0.150 mmol, 1.00 equiv.), (*E*)-2-butenoyl chloride (21.6 µL, 0.225 mmol, 1.50 equiv.) and silver hexafluoroantimonate (77.3 mg, 0.225 mmol, 1.50 equiv.) at –47 °C. The resulting crude material was subjected to flash column chromatography (silica gel, 0%-10% Et_2O in heptane) to obtain the title compound as a colorless oil (25.2 mg, 0.126 mmol, 84% yield). The product was obtained as a mixture of *Z*/*E* isomers in a ratio of >95:5 (determined based on the ^{1}H NMR of the crude reaction mixture).

^{1}H NMR (400 MHz, $CDCl_3$): δ 7.34 – 7.25 (m, 2H), 7.24 – 7.16 (m, 3H), 6.89 (dq, *J* = 15.7, 6.8 Hz, 1H), 6.16 (dq, *J* = 15.7, 1.7 Hz, 1H), 5.87 – 5.67 (m, 2H), 3.46 – 3.37 (m, 4H), 1.90 (dd, *J* = 6.9, 1.7 Hz, 3H) ppm.

^{13}C NMR (151 MHz, $CDCl_3$): δ 197.8, 143.4, 140.3, 131.6, 131.5, 128.6 (2C), 128.5 (2C), 126.2, 122.6, 39.1, 33.9, 18.4 ppm.

IR (neat) ν_{max}: 3027, 1670, 1629, 1494, 1441, 1390, 1292, 1185, 1127, 1075, 968, 737, 697 cm^{-1}.

HRMS (ESI$^+$): exact mass calculated for $[M+Na]^+$ $(C_{14}H_{16}O^{23}Na)^+$ requires *m/z* 223.1099, found *m/z* 223.1093.

(1*E*,5*Z*)-1,7-Diphenylhepta-1,5-dien-3-one (4i)

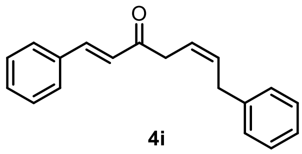

4i

General procedure **GP3** was followed using vinyl silane **2c** (37.0 mg, 0.150 mmol, 1.00 equiv.), cinnamoyl chloride (37.5 mg, 0.225 mmol, 1.50 equiv.) and silver hexafluoroantimonate (77.3 mg, 0.225 mmol, 1.50 equiv.) at –47 °C. The resulting crude material was subjected to flash column chromatography (silica gel, 0%-10% Et_2O in heptane) to obtain the title compound as a colorless oil (28.8 mg, 0.110 mmol, 73% yield). The product was obtained as a mixture of *Z*/*E* isomers in a ratio of >95:5 (determined based on the ^{1}H NMR of the crude reaction mixture).

^{1}H NMR (400 MHz, $CDCl_3$): δ 7.60 (d, *J* = 16.1 Hz, 1H), 7.56 – 7.50 (m, 2H), 7.41 – 7.37 (m, 3H), 7.35 – 7.28 (m, 2H), 7.22 (d, *J* = 7.4 Hz, 3H), 6.78 (d, *J* = 16.1 Hz, 1H), 5.93 – 5.75 (m, 2H), 3.54 (d, *J* = 6.3 Hz, 2H), 3.49 (d, *J* = 6.9 Hz, 2H) ppm.

^{13}C NMR (176 MHz, $CDCl_3$): δ 197.8, 143.2, 140.3, 134.6, 132.0, 130.7, 129.1 (2C), 128.7 (2C), 128.5 (4C), 126.3, 125.6, 122.5, 40.1, 33.9 ppm.

IR (neat) ν_{max}: 3026, 1689, 1661, 1608, 1576, 1494, 1450, 1330, 1202, 1170, 1096, 1075, 1030, 741, 690 cm^{-1}.

HRMS (ESI$^+$): exact mass calculated for $[M+Na]^+$ $(C_{19}H_{18}O^{23}Na)^+$ requires *m*/*z* 285.1255, found *m*/*z* 285.1250.

(1*E*,5*Z*)-1-(Benzo[*d*][1,3]dioxol-5-yl)-7-phenylhepta-1,5-dien-3-one (4j)

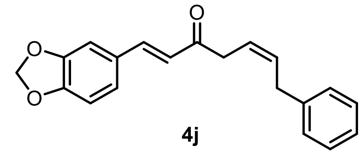

4j

General procedure **GP3** was followed using vinyl silane **2c** (37.0 mg, 0.150 mmol, 1.00 equiv.), acyl chloride **S1** (47.4 mg, 0.225 mmol, 1.50 equiv.) and silver hexafluoroantimonate (77.3 mg, 0.225 mmol, 1.50 equiv.) at 0 °C. The resulting crude material was subjected to flash column chromatography (silica gel, 0%-20% Et_2O in heptane) to obtain the title compound as a colorless oil (39.4 mg, 0.129 mmol, 86% yield). The product was obtained as a mixture of *Z*/*E* isomers in a ratio of >95:5 (determined based on the ^{1}H NMR of the crude reaction mixture).

^{1}H NMR (400 MHz, $CDCl_3$): δ 7.50 (d, *J* = 16.0 Hz, 1H), 7.33 – 7.28 (m, 2H), 7.24 – 7.17 (m, 3H), 7.05 – 6.97 (m, 2H), 6.82 (d, *J* = 7.9 Hz, 1H), 6.60 (d, *J* = 16.0 Hz, 1H), 6.02 (s, 2H), 5.91 – 5.74 (m, 2H), 3.50 (d, *J* = 6.5 Hz, 2H), 3.48 (d, *J* = 7.0 Hz, 2H) ppm.

^{13}C NMR (151 MHz, $CDCl_3$): δ 197.6, 150.1, 148.6, 143.0, 140.3, 131.8, 129.0, 128.7 (2C), 128.5 (2C), 126.3, 125.2, 123.7, 122.7, 108.8, 106.7, 101.8, 40.2, 33.9 ppm.

IR (neat) ν_{max}: 3025, 2901, 1685, 1657, 1593, 1502, 1488, 1446, 1390, 1241, 1170, 1036, 976, 740. 699 cm^{-1}.

HRMS (ESI$^+$): exact mass calculated for $[M+H]^+$ $(C_{20}H_{19}O_3)^+$ requires *m*/*z* 307.1334, found *m*/*z* 307.1329.

(*Z*)-2,2-Dimethyl-7-phenylhept-5-en-3-one (4k)

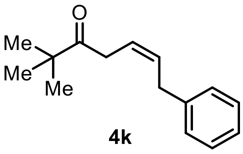

4k

General procedure **GP3** was followed using vinyl silane **2c** (49.3 mg, 0.200 mmol, 1.00 equiv.), pivaloyl chloride (25.9 µL, 0.210 mmol, 1.05 equiv.) and silver hexafluoroantimonate (75.6 mg, 0.220 mmol, 1.10 equiv.) at –78 °C. The resulting crude material was subjected to flash column chromatography (silica gel, 0%-7% EtOAc in heptane) to obtain the title compound as a colorless oil (29.5 mg, 0.136 mmol, 68% yield). The product was obtained as a mixture of *Z*/*E* isomers in a ratio of >95:5 (determined based on the ^{1}H NMR of the crude reaction mixture).

^{1}H NMR (400 MHz, $CDCl_3$): δ 7.34 – 7.26 (m, 2H), 7.23 – 7.15 (m, 3H), 5.83 – 5.67 (m, 2H), 3.47 – 3.31 (m, 4H), 1.17 (s, 9H) ppm.

^{13}C NMR (101 MHz, $CDCl_3$): δ 213.6, 140.4, 130.9, 128.6 (2C) 128.5 (2C), 126.2, 123.3, 44.5, 35.2, 33.9, 26.6 (3C) ppm.

IR (neat) ν_{max}: 3028, 2969, 1708, 1602, 1495, 1454, 1365, 1089, 1072, 1011, 945, 783, 740, 699, 530, 413 cm^{-1}.

HRMS (ESI$^+$): exact mass calculated for $[M+Na]^+$ $(C_{15}H_{20}O^{23}Na)^+$ requires *m*/*z* 239.1406, found *m*/*z* 239.1403.

(*Z*)-2-Methyl-7-phenylhept-5-en-3-one (4l)

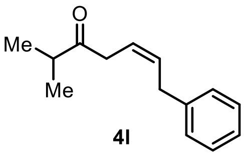

4l

General procedure **GP3** was followed using vinyl silane **2c** (37.0 mg, 0.150 mmol, 1.00 equiv.), isobutyryl chloride (16.5 µL, 0.160 mmol, 1.05 equiv.) and silver hexafluoroantimonate (56.7 mg, 0.165 mmol, 1.10 equiv.) at –78 °C. The resulting crude material was subjected to flash column chromatography (silica gel, 0%-10% Et_2O in heptane) to obtain the title compound as a colorless oil (23.9 mg, 0.119 mmol, 79% yield). The product was obtained as a mixture of *Z*/*E* isomers in a ratio of >95:5 (determined based on the ^{1}H NMR of the crude reaction mixture).

^{1}H NMR (700 MHz, $CDCl_3$): δ 7.31 – 7.24 (m, 2H), 7.23 – 7.16 (m, 3H), 5.79 (dtt, *J* = 10.4, 7.2, 1.6 Hz, 1H), 5.73 (dtt, *J* = 10.8, 7.1, 1.5 Hz, 1H), 3.41 (d, *J* = 7.3 Hz, 2H), 3.35 – 3.31 (m, 2H), 2.67 (hept, *J* = 6.9 Hz, 1H), 1.12 (d, *J* = 6.9 Hz, 6H) ppm.

^{13}C NMR (176 MHz, $CDCl_3$): δ 212.4, 140.3, 131.5, 128.7 (2C), 128.5 (2C), 126.2, 122.5, 40.8, 39.2, 33.9, 18.4 (2C) ppm.

IR (neat) ν_{max}: 3027, 2970, 2932, 1711, 1494, 1465, 1453, 1383, 1069, 1031, 828, 739, 697 cm^{-1}.

HRMS (ESI$^+$): exact mass calculated for $[M+Na]^+$ $(C_{14}H_{18}O^{23}Na)^+$ requires *m/z* 225.1255, found *m/z* 225.1250.

(*Z*)-1-Cyclohexyl-5-phenylpent-3-en-1-one (4m)

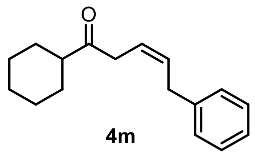

4m

General procedure **GP3** was followed using vinyl silane **2c** (37.0 mg, 0.150 mmol, 1.00 equiv.), cyclohexanecarbonyl chloride (21.2 μL, 0.160 mmol, 1.05 equiv.) and silver hexafluoroantimonate (56.7 mg, 0.165 mmol, 1.10 equiv.) at –78 °C. The resulting crude material was subjected to flash column chromatography (silica gel, 0%-10% Et_2O in heptane) to obtain the title compound as a colorless oil (24.6 mg, 0.102 mmol, 68% yield). The product was obtained as a mixture of *Z*/*E* isomers in a ratio of >95:5 (determined based on the ^{1}H NMR of the crude reaction mixture).

^{1}H NMR (400 MHz, CDCl$_3$): δ 7.29 (t, *J* = 7.4 Hz, 2H), 7.19 (t, *J* = 9.1 Hz, 3H), 5.76 (ddt, *J* = 22.5, 11.1, 5.8 Hz, 2H), 3.40 (d, *J* = 7.1 Hz, 2H), 3.31 (d, *J* = 6.7 Hz, 2H), 2.40 (tt, *J* = 11.3, 3.5 Hz, 1H), 1.90 – 1.72 (m, 4H), 1.71 – 1.63 (m, 1H), 1.41 – 1.21 (m, 5H) ppm.

^{13}C NMR (176 MHz, CDCl$_3$): δ 211.8, 140.3, 131.5, 128.6 (2C), 128.5 (2C), 126.2, 122.6, 50.7, 39.5, 33.9, 28.7 (2C), 26.0, 25.8 (2C) ppm.

IR (neat) ν_{max}: 3027, 2929, 2854, 1709, 1494, 1450, 1369, 1316, 1217, 1143, 1006, 738, 698 cm^{-1}.

HRMS (ESI$^+$): exact mass calculated for $[M+Na]^+$ $(C_{17}H_{22}O^{23}Na)^+$ requires *m/z* 265.1568, found *m/z* 265.1563.

(*Z*)-1-Cyclopropyl-5-phenylpent-3-en-1-one (4n)

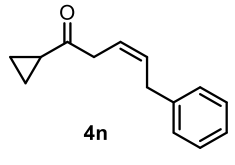

4n

General procedure **GP3** was followed using vinyl silane **2c** (49.3 mg, 0.200 mmol, 1.00 equiv.), cyclopropanecarbonyl chloride (19.1 μL, 0.210 mmol, 1.05 equiv.) and silver hexafluoroantimonate (75.6 mg, 0.220 mmol, 1.10 equiv.) at –61 °C. The resulting crude material was subjected to flash column chromatography (silica gel, 0%-10% EtOAc in heptane) to obtain the title compound as a pale-yellow oil (24.4 mg, 0.122 mmol, 61% yield). The product was obtained as a mixture of *Z*/*E* isomers in a ratio of >95:5 (determined based on the ^{1}H NMR of the crude reaction mixture).

^{1}H NMR (400 MHz, CDCl$_3$): δ 7.33 – 7.27 (m, 2H), 7.23 – 7.16 (m, 3H), 5.87 – 5.71 (m, 2H), 3.49 – 3.38 (m, 4H), 1.97 (tt, *J* = 7.8, 4.6 Hz, 1H), 1.09 – 1.02 (m, 2H), 0.92 – 0.85 (m, 2H) ppm.

^{13}C NMR (101 MHz, CDCl$_3$): δ 208.6, 140.3, 131.7, 128.7 (2C), 128.5 (2C) 126.2, 122.3, 42.3, 33.9, 20.4, 11.2 (2C) ppm.

IR (neat) ν_{max}: 3026, 1699, 1494, 1453, 1383, 1195, 1076, 1028, 902, 816, 742, 699 cm^{-1}.

HRMS (ESI$^+$): exact mass calculated for $[M+Na]^+$ $(C_{14}H_{16}O^{23}Na)^+$ requires *m/z* 223.1093, found *m/z* 223.1093.

(*Z*)-1-Phenylhexadec-2-en-5-one (4o)

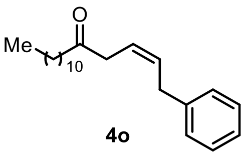

4o

General procedure **GP3** was followed using vinyl silane **2c** (40.0 mg, 0.160 mmol, 1.00 equiv.), lauroyl chloride (39.4 µL, 0.170 mmol, 1.05 equiv.) and silver hexafluoroantimonate (61.3 mg, 0.179 mmol, 1.10 equiv.) at –78 °C. The resulting crude material was subjected to flash column chromatography (silica gel, 0%-10% EtOAc in heptane) to obtain the title compound as a colorless oil (27.2 mg, 0.087 mmol, 53% yield). The product was obtained as a mixture of *Z*/*E* isomers in a ratio of >95:5 (determined based on the ^{1}H NMR of the crude reaction mixture).

^{1}H NMR (400 MHz, $CDCl_3$): δ 7.37 – 7.04 (m, 5H), 5.85 – 5.63 (m, 2H), 3.41 (d, *J* = 7.1 Hz, 2H), 3.27 (d, *J* = 7.0 Hz, 2H), 2.44 (t, *J* = 7.5 Hz, 2H), 1.65 – 1.51 (m, 2H), 1.33 – 1.23 (m, 16H), 0.88 (t, *J* = 6.9 Hz, 3H) ppm.

^{13}C NMR (101 MHz, $CDCl_3$): 209.0, 140.3, 131.7, 128.7 (2C), 128.5 (2C), 126.2, 122.4, 42.7, 41.7, 33.8, 32.1, 29.8 (2C), 29.62, 29.55, 29.48, 29.4, 24.0, 22.8, 14.3 ppm.

IR (neat) ν_{max}: 3027, 2924, 2853, 2360, 2342, 1738, 1720, 1495, 1455, 1366, 1228, 1217, 741, 697, 669 cm^{-1}.

HRMS (ESI$^+$): exact mass calculated for $[M+Na]^+$ $(C_{22}H_{34}O^{23}Na)^+$ requires *m/z* 337.2502, found *m/z* 337.2500.

(*Z*)-6-Phenylhex-4-en-2-one (4p)

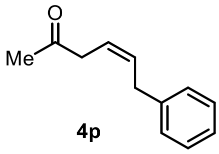

4p

General procedure **GP3** was followed using vinyl silane **2c** (37.0 mg, 0.150 mmol, 1.00 equiv.), acetyl chloride (10.6 µL, 0.160 mmol, 1.05 equiv.) and silver hexafluoroantimonate (56.7 mg, 0.165 mmol, 1.10 equiv.) at –47 °C. The resulting crude material was subjected to flash column chromatography (silica gel, 0%-10% Et_2O in heptane) to obtain the title compound as a colorless oil (21.4 mg, 0.123 mmol, 82% yield). The product was obtained as a mixture of *Z*/*E* isomers in a ratio of 93:7 (determined based on the ^{1}H NMR of the crude reaction mixture). NMR peaks in ^{1}H NMR corresponding exclusively to the minor isomer are indicated with an asterisk.

^{1}H NMR (600 MHz, $CDCl_3$): δ 7.30 (t, *J* = 7.5 Hz, 2H), 7.25 – 7.14 (m, 3H), 5.85 – 5.78 (m, 1H), 5.75 – 5.68 (m, 1H), 3.41 (d, *J* = 7.5 Hz, 2H), 3.30 (d, *J* = 7.4 Hz, 1.86H), 3.16 (d, *J* = 6.7 Hz, 0.14H*), 2.18 (s, 3H) ppm.

^{13}C NMR (151 MHz, $CDCl_3$): δ 206.6, 140.2, 132.0, 128.7 (2C), 128.4 (2C), 126.2, 122.1, 42.5, 33.8, 29.8 ppm.

IR (neat) ν_{max}: 3026, 1714, 1602, 1494, 1453, 1422, 1390, 1356, 1316, 1229, 1159, 1030, 697, 547, 560 cm^{-1}.

HRMS (ESI$^+$): exact mass calculated for $[M+Na]^+$ $(C_{12}H_{14}O^{23}Na)^+$ requires *m*/*z* 197.0942, found *m*/*z* 197.0937.

(*Z*)-1,6-Diphenylhex-4-en-2-one (4q)

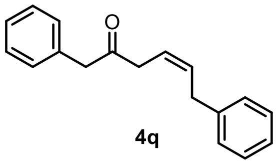

4q

General procedure **GP3** was followed using vinyl silane **2c** (37.0 mg, 0.150 mmol, 1.00 equiv.), phenylacetyl chloride (10.6 µL, 0.225 mmol, 1.50 equiv.) and silver hexafluoroantimonate (103.0 mg, 0.300 mmol, 2.00 equiv.) at –78 °C. The resulting crude material was subjected to flash column chromatography (silica gel, 0%-10% Et_2O in heptane) to obtain the title compound as a colorless oil (32.4 mg, 0.129 mmol, 86% yield). The product was obtained as a mixture of *Z*/*E* isomers in a ratio of 91:9 (determined based on the ^{1}H NMR of the crude reaction mixture). NMR peaks in ^{1}H NMR corresponding exclusively to the minor isomer are indicated with an asterisk.

^{1}H NMR (700 MHz, $CDCl_3$): δ 7.36 – 7.30 (m, 2H), 7.30 – 7.25 (m, 3H), 7.24 – 7.18 (m, 3H), 7.18 – 7.12 (m, 2H), 5.84 – 5.76 (m, 0.91H), 5.72 – 5.64 (m, 1H), 5.63 – 5.57 (m, 0.09H*), 3.73 (s, 1.82H), 3.71 (s, 0.18H*), 3.38 (d, *J* = 6.6 Hz, 0.18H*), 3.32 (t, *J* = 7.1 Hz, 3.64H), 3.19 (d, *J* = 7.5 Hz, 0.18H*) ppm.

^{13}C NMR (176 MHz, $CDCl_3$): δ 206.1, 140.2, 134.2, 132.1, 129.6 (2C), 128.9 (2C), 128.7 (2C), 128.4 (2C), 127.3, 126.2, 122.0, 49.9, 40.8, 33.8 ppm.

IR (neat) ν_{max}: 3061, 3027, 2917, 1714, 1602, 1495, 1453, 1391, 1327, 1072, 1030, 969, 740, 699 cm^{-1}.

HRMS (ESI$^+$): exact mass calculated for $[M+Na]^+$ $(C_{18}H_{18}O^{23}Na)^+$ requires *m*/*z* 273.1255, found *m*/*z* 273.1250.

(*Z*)-2-Methyl-8-phenyloct-6-en-4-one (4r)

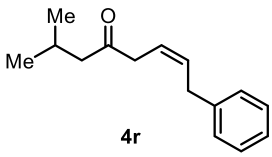

4r

General procedure **GP3** was followed using vinyl silane **2c** (37.0 mg, 0.150 mmol, 1.00 equiv.), isovaleryl chloride (19.2 µL, 0.160 mmol, 1.05 equiv.) and silver hexafluoroantimonate (56.7 mg, 0.165 mmol, 1.10 equiv.) at –78 °C. The resulting crude material was subjected to flash column chromatography (silica gel, 0%-10% Et_2O in heptane) to obtain the title compound as a colorless oil (23.1 mg, 0.107 mmol, 71% yield). The product was obtained as a mixture of *Z*/*E* isomers in a ratio of >95:5 (determined based on the ^{1}H NMR of the crude reaction mixture).

^{1}H NMR (500 MHz, $CDCl_3$): δ 7.32 – 7.26 (m, 2H), 7.24 – 7.15 (m, 3H), 5.85 – 5.76 (m, 1H), 5.76 – 5.66 (m, 1H), 3.41 (d, *J* = 7.8 Hz, 2H), 3.25 (d, *J* = 6.6 Hz, 2H), 2.33 (d, *J* = 6.7 Hz, 2H), 2.15 (n, *J* = 6.7 Hz, 1H), 0.92 (d, *J* = 6.6 Hz, 6H) ppm.

^{13}C NMR (126 MHz, CDCl$_3$): δ 208.5, 140.3, 131.7, 128.7 (2C), 128.5 (2C), 126.2, 122.3, 51.6, 42.2, 33.8, 24.7, 22.7 (2C) ppm.

IR (neat) ν$_{max}$: 3026, 2965, 2870, 1738, 1716, 1451, 1366, 1266, 1229, 1217, 739, 703 cm^{-1}.

HRMS (ESI$^+$): exact mass calculated for $[M+Na]^+$ $(C_{15}H_{20}O^{23}Na)^+$ requires *m/z* 239.1412, found *m/z* 239.1406.

(*Z*)-1-Cyclopentyl-7-phenylhept-5-en-3-one (4s)

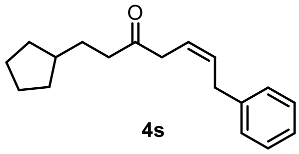

4s

General procedure **GP3** was followed using vinyl silane **2c** (37.0 mg, 0.150 mmol, 1.00 equiv.), cyclopentanepropionyl chloride (24.1 µL, 0.160 mmol, 1.05 equiv.) and silver hexafluoroantimonate (56.7 mg, 0.165 mmol, 1.10 equiv.) at –78 °C. The resulting crude material was subjected to flash column chromatography (silica gel, 0%-10% Et_2O in heptane) to obtain the title compound as a colorless oil (32.0 mg, 0.125 mmol, 83% yield). The product was obtained as a mixture of *Z*/*E* isomers in a ratio of >95:5 (determined based on the ^{1}H NMR of the crude reaction mixture).

^{1}H NMR (400 MHz, CDCl$_3$): δ 7.29 (t, *J* = 7.4 Hz, 2H), 7.25 – 7.13 (m, 3H), 5.87 – 5.64 (m, 2H), 3.41 (d, *J* = 7.2 Hz, 2H), 3.28 (d, *J* = 6.9 Hz, 2H), 2.46 (t, *J* = 7.7 Hz, 2H), 1.80 – 1.44 (m, 9H), 1.14 – 0.99 (m, 2H) ppm.

^{13}C NMR (126 MHz, CDCl$_3$): δ 209.1, 140.3, 131.7, 128.7 (2C), 128.5 (2C), 126.2, 122.4, 42.0, 41.6, 39.8, 33.8, 32.6 (2C), 30.1, 25.3 (2C) ppm.

IR (neat) ν$_{max}$: 3026, 2945, 2864, 1714, 1494, 1452, 1390, 1324, 1267, 1072, 1030, 738, 698 cm^{-1}.

HRMS (ESI$^+$): exact mass calculated for $[M+Na]^+$ $(C_{18}H_{24}O^{23}Na)^+$ requires *m/z* 279.1725, found *m/z* 279.1719.

(*Z*)-1,7-Diphenylhept-5-en-3-one (4t)

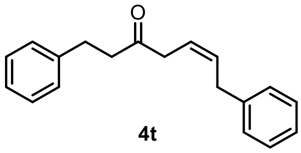

4t

General procedure **GP3** was followed using vinyl silane **2c** (37.0 mg, 0.150 mmol, 1.00 equiv.), hydrocinnamyl chloride (23.8 µL, 0.160 mmol, 1.05 equiv.) and silver hexafluoroantimonate (56.7 mg, 0.165 mmol, 1.10 equiv.) at –78 °C. The resulting crude material was subjected to flash column chromatography (silica gel, 0%-10% Et_2O in heptane) to obtain the title compound as a colorless oil (33.4 mg, 0.126 mmol, 84% yield). The product was obtained as a mixture of *Z/E* isomers in a ratio of 94:6 (determined based on the ^{1}H NMR of the crude reaction mixture). NMR peaks in ^{1}H NMR corresponding exclusively to the minor isomer are indicated with an asterisk.

^{1}H NMR (600 MHz, CDCl$_3$): δ 7.32 – 7.26 (m, 4H), 7.23 – 7.14 (m, 6H), 5.84 – 5.77 (m, 0.93H), 5.73 – 5.66 (m, 1H), 5.64 – 5.57 (m, 0.07H*), 3.38 (d, *J* = 7.4 Hz, 2H), 3.26 (d, *J* = 7.2 Hz, 1.88H), 3.13 (d, *J* = 6.7 Hz, 0.12H*), 2.92 (t, *J* = 7.6 Hz, 2H), 2.78 (t, *J* = 7.6 Hz, 2H) ppm.

^{13}C NMR (151 MHz, CDCl$_3$): δ 207.7, 141.1, 140.2, 131.9, 128.66 (2C), 128.65 (2C), 128.46 (2C), 128.44 (2C), 126.3, 126.2, 122.0, 44.1, 41.9, 33.8, 29.9 ppm.

IR (neat) ν_{max}: 3026, 1714, 1602, 1495, 1453, 1365, 1217, 1092, 1030, 741, 698, 542 cm^{-1}.

HRMS (ESI$^+$): exact mass calculated for $[M+Na]^+$ ($C_{19}H_{20}O^{23}Na$)$^+$ requires *m*/*z* 287.1412, found *m*/*z* 287.1406.

(*Z*)-10-Bromo-1-phenyldec-2-en-5-one (4u)

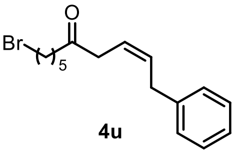

4u

General procedure **GP3** was followed using vinyl silane **2c** (37.0 mg, 0.150 mmol, 1.00 equiv.), 6-bromohexanoyl chloride (24.1 µL, 0.160 mmol, 1.05 equiv.) and silver hexafluoroantimonate (56.7 mg, 0.165 mmol, 1.10 equiv.) at –78 °C. The resulting crude material was subjected to flash column chromatography (silica gel, 0%-10% Et_2O in heptane) to obtain the title compound as a colorless oil (38.9 mg, 0.126 mmol, 84% yield). The product was obtained as a mixture of *Z*/*E* isomers in a ratio of 93:7 (determined based on the ^{1}H NMR of the crude reaction mixture). NMR peaks in ^{1}H NMR corresponding exclusively to the minor isomer are indicated with an asterisk.

^{1}H NMR (600 MHz, CDCl$_3$): δ 7.33 – 7.24 (m, 2H), 7.24 – 7.15 (m, 3H), 5.85 – 5.78 (m, 0.93H), 5.74 – 5.68 (m, 1H), 5.66 – 5.59 (m, 0.07H*), 3.44 – 3.37 (m, 4H), 3.27 (d, *J* = 7.2 Hz, 1.86H), 3.14 (d, *J* = 6.8 Hz, 0.14H*), 2.47 (t, *J* = 7.4 Hz, 2H), 1.86 (p, *J* = 7.0 Hz, 2H), 1.61 (p, *J* = 7.4 Hz, 2H), 1.48 – 1.38 (m, 2H) ppm.

^{13}C NMR (151 MHz, CDCl$_3$): δ 208.4, 140.2, 131.8, 128.6 (2C), 128.4 (2C), 126.2, 122.2, 42.2, 41.7, 33.8, 33.7, 32.6, 27.8, 22.9 ppm.

IR (neat) ν_{max}: 3026, 2934, 1713, 1602, 1494, 1453, 1395, 1366, 1252, 738, 699 cm^{-1}.

HRMS (ESI$^+$): exact mass calculated for $[M+Na]^+$ ($C_{16}H_{21}{}^{79}BrO^{23}Na$)$^+$ requires *m*/*z* 331.0673, found *m*/*z* 331.0668.

(*Z*)-1-Chloro-8-phenyloct-6-en-4-one (4v)

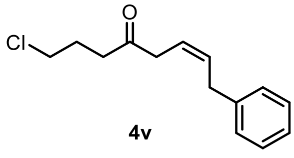

4v

General procedure **GP3** was followed using vinyl silane **2c** (49.3 mg, 0.200 mmol, 1.00 equiv.), 4-chlorobutyryl chloride (34 µL, 0.300 mmol, 1.5 equiv.) and silver hexafluoroantimonate (103 mg, 0.300 mmol, 1.50 equiv.) at

–78 °C. The resulting crude material was subjected to flash column chromatography (silica gel, 0%-15% EtOAc in heptane) to obtain the title compound as a pale-yellow oil (41.3 mg, 0.174 mmol, 87% yield). The product was obtained as a mixture of *Z*/*E* isomers in a ratio of 93:7 (determined based on the ^{1}H NMR of the crude reaction mixture). NMR peaks in ^{1}H NMR corresponding exclusively to the minor isomer are indicated with an asterisk.

^{1}H NMR (400 MHz, CDCl$_3$): δ 7.34 – 7.27 (m, 2H), 7.24 – 7.13 (m, 3H), 5.88 – 5.78 (m, 1H), 5.77 – 5.66 (m, 1H), 3.57 (t, *J* = 6.3 Hz, 2H), 3.46 – 3.37 (m, 2H), 3.30 (d, *J* = 7.1 Hz, 1.87H), 3.16 (d, *J* = 6.9 Hz, 0.13H*), 2.66 (t, *J* = 6.9 Hz, 2H), 2.11 – 2.00 (m, 2H) ppm.

^{13}C NMR (101 MHz, CDCl$_3$): δ 207.5, 140.2, 132.1, 128.7 (2C), 128.5 (2C), 126.3, 121.9, 44.6, 41.9, 39.1, 33.8, 26.4 ppm.

IR (neat) ν$_{max}$: 3027, 2919, 1715, 1601, 1495, 1453, 1309, 1094, 1030, 911, 828, 738, 699, 648 cm^{-1}.

HRMS (ESI$^+$): exact mass calculated for [M+Na]$^+$ ($C_{14}H_{17}{}^{35}ClO^{23}Na$)$^+$ requires *m*/*z* 259.0860, found *m*/*z* 259.0857.

(*Z*)-1-Iodoheptadec-3-en-6-one (5a)

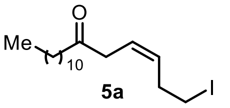

General procedure **GP3** was followed using vinyl silane **2d** (46.5 mg, 0.150 mmol, 1.00 equiv.), lauroyl chloride (49 mg, 52 µL, 0.230 mmol, 1.50 equiv.) and silver hexafluoroantimonate (77.3 mg, 0.225 mmol, 1.50 equiv.) at –47 °C. The resulting crude material was subjected to flash column chromatography (silica gel, 0%-5% EtOAc in heptane) to obtain the title compound as a colorless solid (46.3 mg, 0.122 mmol, 82% yield). The product was obtained as a mixture of *Z*/*E* isomers in a ratio of 94:6 (determined based on the ^{1}H NMR of the purified product). NMR peaks in ^{1}H NMR corresponding exclusively to the minor isomer are indicated with an asterisk.

^{1}H NMR (700 MHz, C$_6$D$_6$): δ 5.71 – 5.66 (m, 0.94H), 5.49 – 5.43 (m, 0.06H*), 5.22 – 5.17 (m, 0.94H), 5.14 – 5.09 (m, 0.06H*), 2.70 (dd, *J* = 7.2, 1.6 Hz, 2H), 2.65 (t, *J* = 7.1 Hz, 2H), 2.21 (q, *J* = 7.2 Hz, 2H), 2.01 (t, *J* = 7.3 Hz, 2H), 1.52 (dt, *J* = 14.9, 7.4 Hz, 2H), 1.34 – 1.22 (m, 16H), 0.92 (t, *J* = 7.1 Hz, 3H) ppm.

^{13}C NMR (101 MHz, C$_6$D$_6$): δ 205.9, 131.0, 124.3, 42.4, 41.4, 32.4, 31.9, 30.1 (2C), 30.0, 29.9, 29.8, 29.6, 24.0, 23.1, 14.4, 4.5 ppm.

IR (neat) ν$_{max}$: 2923, 2853, 1713, 1457, 1360, 1219, 1089, 721, 529 cm^{-1}.

HRMS (ESI$^+$): exact mass calculated for [M+Na]$^+$ ($C_{17}H_{31}{}^{127}IO^{23}Na$)$^+$ requires *m*/*z* 401.1312, found *m*/*z* 401.1315.

(*Z*)-6-Iodo-1-phenylhex-3-en-1-one (5b)

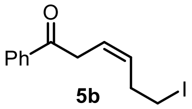

5b

General procedure **GP3** was followed using vinyl silane **2d** (46.5 mg, 0.150 mmol, 1.00 equiv.), benzoyl chloride (31.6 mg, 26.1 µL, 0.225 mmol, 1.50 equiv.) and silver hexafluoroantimonate (77.3 mg, 0.225 mmol, 1.50 equiv.) at –61 °C. The resulting crude material was subjected to flash column chromatography (silica gel, 0%-20% EtOAc in heptane) to obtain the title compound as a colorless oil (29.1 mg, 0.097 mmol, 65% yield). The product was obtained as a mixture of *Z*/*E* isomers in a ratio of >95:5 (determined based on the ^{1}H NMR of the purified product).

^{1}H NMR (400 MHz, C_6D_6): δ 7.80 – 7.74 (m, 2H), 7.19 – 7.10 (m, 1H), 7.08 – 7.02 (m, 2H), 5.87 – 5.78 (m, 1H), 5.29 – 5.16 (m, 1H), 3.26 (d, *J* = 8.6 Hz, 2H), 2.65 (t, *J* = 7.2 Hz, 2H), 2.25 (q, *J* = 7.2 Hz, 2H) ppm.

^{13}C NMR (101 MHz, C_6D_6): δ 196.2, 137.2, 132.9, 131.1, 128.7 (2C), 128.5 (2C), 124.4, 37.3, 32.0, 4.6 ppm.

IR (neat) ν_{max}: 3025, 2957, 1682, 1597, 1447, 1330, 1208, 1001, 688 cm^{-1}.

HRMS (ESI^{+}): exact mass calculated for $[M+H]^{+}$ $(C_{12}H_{14}{}^{127}IO)^{+}$ requires *m/z* 301.0084, found *m/z* 301.0081.

(*Z*)-1,1-Bis(4,4,5,5-tetramethyl-1,3,2-dioxaborolan-2-yl)octadec-4-en-7-one (5c)

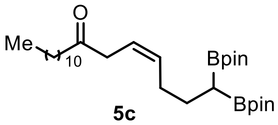

5c

General procedure **GP3** was followed using vinyl silane **2e** (67.6 mg, 0.150 mmol, 1.00 equiv.), lauroyl chloride (49 mg, 52 µL, 0.230 mmol, 1.50 equiv.) and silver hexafluoroantimonate (77.3 mg, 0.225 mmol, 1.50 equiv.) at –47 °C. The resulting crude material was subjected to flash column chromatography (silica gel, 0%-30% EtOAc in heptane) to obtain the title compound as a colorless oil (49.9 mg, 0.099 mmol, 66% yield). The product was obtained as a mixture of *Z*/*E* isomers in a ratio of 94:6 (determined based on the ^{1}H NMR of the crude reaction mixture). NMR peaks in ^{1}H NMR corresponding exclusively to the minor isomer are indicated with an asterisk.

^{1}H NMR (400 MHz, $CDCl_3$): δ 5.63 – 5.45 (m, 2H), 3.14 (d, *J* = 6.5 Hz, 1.88H), 3.04 (d, *J* = 5.3 Hz, 0.12H*), 2.40 (t, *J* = 7.4 Hz, 2H), 2.01 (q, *J* = 7.3 Hz, 2H), 1.68 – 1.49 (m, 4H), 1.40 – 1.12 (m, 40H), 0.87 (t, *J* = 6.8 Hz, 3H), 0.72 (t, *J* = 7.7 Hz, 1H) ppm.

^{13}C NMR (101 MHz, $CDCl_3$): δ 209.6, 133.5, 121.3, 83.1 (4C), 42.5, 41.8, 32.0, 30.4, 29.7 (2C), 29.6 (2C), 29.5, 29.4, 25.6, 25.0 (4C), 24.7 (4C), 23.9, 22.8, 14.2 ppm. *(The carbon attached to boron could not be observed due to quadrupolar relaxation)*

IR (neat) ν_{max}: 2924, 2854, 1716, 1459, 1360, 1310, 1138, 969, 849 cm^{-1}.

HRMS (ESI^{+}): exact mass calculated for $[M+Na]^{+}$ $(C_{30}H_{56}{}^{11}B_2O_5{}^{23}Na)^{+}$ requires *m/z* 541.4206, found *m/z* 541.4218.

(*Z*)-*N*-Benzyl-*N*-(7-oxooctadec-4-en-1-yl)-2,4,6-tris(trifluoromethyl)benzenesulfonamide (5d)

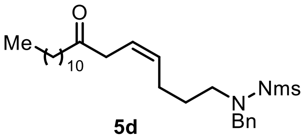

5d

General procedure **GP3** was followed using vinyl silane **2f** (64.8 mg, 0.100 mmol, 1.00 equiv.), lauroyl chloride (32.8 mg, 34.7 µL, 0.150 mmol, 1.50 equiv.) and silver hexafluoroantimonate (51.5 mg, 0.150 mmol, 1.50 equiv.) at –47 °C. The resulting crude material was subjected to flash column chromatography (silica gel, 0%-20% EtOAc in heptane) to obtain the title compound as a colorless solid (48.5 mg, 0.068 mmol, 68% yield). The product was obtained as a mixture of *Z*/*E* isomers in a ratio of 91:9 (determined based on the ^{1}H NMR in C_6D_6 of purified product due to better separation of characteristic peaks).

^{1}H NMR (400 MHz, CDCl$_3$): δ 8.22 (s, 2H), 7.31 – 7.23 (m, 5H), 5.54 – 5.45 (m, 1H), 5.36 – 5.28 (m, 1H), 4.50 (s, 2H), 3.20 – 3.12 (m, 2H), 3.02 (d, *J* = 6.9 Hz, 2H), 2.36 (t, *J* = 7.5 Hz, 2H), 1.84 (q, *J* = 7.1 Hz, 2H), 1.59 – 1.39 (m, 4H), 1.31 – 1.21 (m, 16H), 0.88 (t, *J* = 6.8 Hz, 3H) ppm.

^{13}C NMR (101 MHz, CDCl$_3$): δ 208.7, 145.9, 135.2, 134.2 (q, *J* = 34.9 Hz, 2C), 133.2 (q, *J* = 33.8 Hz, 2C), 131.5, 129.4 (m), 129.2 (2C), 128.8 (2C), 128.4, 122.5, 122.2 (q, *J* = 275.7 Hz, 2C), 122.0 (q, *J* = 273.5 Hz), 53.3, 49.1, 42.6, 41.5, 32.1, 29.8 (2C), 29.61, 29.55, 29.51, 29.3, 27.4, 24.9, 23.9, 22.8, 14.3 ppm.

^{19}F NMR (376 MHz, CDCl$_3$): δ -55.01 (s, 6F), -63.61 (s, 3F) ppm.

IR (neat) ν_{max}: 2925, 2855, 1715, 1365, 1273, 1194, 1149, 914, 702 cm^{-1}.

HRMS (ESI$^+$): exact mass calculated for [M+Na]$^+$ ($C_{34}H_{42}F_9NO_3{}^{32}S^{23}Na)^+$ requires *m/z* 738.2634, found *m/z* 738.2632.

(*Z*)-2-(7-Oxooctadec-4-en-1-yl)isoindoline-1,3-dione (5e)

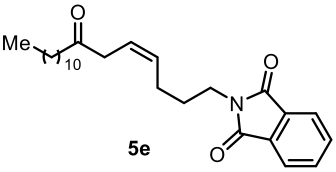

5e

General procedure **GP3** was followed using vinyl silane **2g** (55.8 mg, 0.162 mmol, 1.00 equiv.), lauroyl chloride (56 µL, 0.240 mmol, 1.50 equiv.) and silver hexafluoroantimonate (83.6 mg, 0.243 mmol, 1.50 equiv.) at –47 °C. The resulting crude material was subjected to flash column chromatography (silica gel, 0%-30% EtOAc in heptane) to obtain the title compound as a colorless oil (48.3 mg, 0.117 mmol, 72% yield). The product was obtained as a mixture of *Z*/*E* isomers in a ratio of 93:7 (determined based on the ^{1}H NMR of the crude reaction mixture). mixture). NMR peaks in ^{1}H NMR corresponding exclusively to the minor isomer are indicated with an asterisk.

^{1}H NMR (400 MHz, CDCl$_3$): δ 7.87 – 7.80 (m, 2H), 7.75 – 7.67 (m, 2H), 5.66 – 5.49 (m, 2H), 3.69 (t, *J* = 7.2 Hz, 2H), 3.21 – 3.10 (m, 1.88H), 3.07 (d, *J* = 5.7 Hz, 0.12H*), 2.41 (t, *J* = 7.4 Hz, 2H), 2.17 – 2.03 (m, 2H), 1.83 – 1.71 (m, 2H), 1.61 – 1.49 (m, 2H), 1.34 – 1.20 (m, 16H), 0.87 (t, *J* = 6.8 Hz, 3H) ppm.

^{13}C NMR (101 MHz, CDCl$_3$): δ 209.1, 168.5 (2C), 134.1 (2C), 132.3 (2C), 131.9, 123.3 (2C), 122.3, 42.6, 41.6, 37.8, 32.0, 29.7 (2C), 29.61, 29.55, 29.47, 29.36, 28.3, 25.1, 23.9, 22.8, 14.3 ppm.

IR (neat) ν_{max}: 2924, 2853, 1772, 1713, 1467, 1438, 1395, 1369, 1020, 910, 721, 530 cm^{-1}.

HRMS (ESI$^+$): exact mass calculated for $[M+Na]^+$ $(C_{26}H_{37}NO_3{}^{23}Na)^+$ requires *m/z* 434.2666, found *m/z* 434.2664.

(*Z*)-7-Oxooctadec-4-en-1-yl 2,2,2-trifluoroacetate (5f)

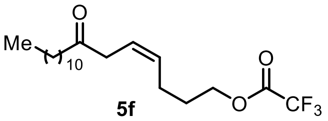

5f

General procedure **GP3** was followed using vinyl silane **2i** (50.0 mg, 0.104 mmol, 1.00 equiv.), lauroyl chloride (36 µL, 0.160 mmol, 1.50 equiv.) and silver hexafluoroantimonate (53.8 mg, 0.157 mmol, 1.50 equiv.) at –47 °C. The resulting crude material was subjected to flash column chromatography (silica gel, 0%-25% EtOAc in heptane) to obtain the title compound as a colorless oil (31.4 mg, 0.0830 mmol, 79% yield). The product was obtained as a mixture of *Z*/*E* isomers in a ratio of 91:9 (determined based on the ^{1}H NMR of the crude reaction mixture). NMR peaks in ^{1}H NMR corresponding exclusively to the minor isomer are indicated with an asterisk.

^{1}H NMR (400 MHz, CDCl$_3$): δ 5.71 – 5.60 (m, 1H), 5.60 – 5.50 (m, 1H), 4.35 (t, *J* = 6.4 Hz, 2H), 3.16 (d, *J* = 7.1 Hz, 1.82H), 3.11 (d, *J* = 6.2 Hz, 0.18H*), 2.41 (t, *J* = 7.4, 2H), 2.22 – 2.09 (m, 2H), 1.89 – 1.77 (m, 2H), 1.65 – 1.50 (m, 2H), 1.34 – 1.21 (m, 16H), 0.88 (t, *J* = 7.0 Hz, 3H) ppm.

^{13}C NMR (101 MHz, CDCl$_3$): δ 208.7, 157.6 (q, *J* = 42.1 Hz), 131.0, 123.2, 114.7 (q, *J* = 285.6 Hz), 67.4, 42.7, 41.3, 32.0, 29.7 (2C), 29.6, 29.52, 29.45, 29.3, 27.8, 23.9, 23.5, 22.8, 14.2 ppm.

^{19}F NMR (376 MHz, CDCl$_3$): δ -75.12 (s, 3F).

IR (neat) ν_{max}: 2925, 2855, 2359, 1786, 1717, 1466, 1402, 1346, 1221, 1154, 777, 731, 523 cm^{-1}

HRMS (ESI$^+$): exact mass calculated for $[M+Na]^+$ $(C_{20}H_{33}F_3O_3{}^{23}Na)^+$ requires *m/z* 401.2274, found *m/z* 401.2272.

(*Z*)-7-Oxooctadec-4-en-1-yl 4-methylbenzenesulfonate (5g)

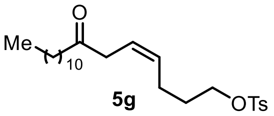

5g

General procedure **GP3** was followed using vinyl silane **2j** (80.5 mg, 0.150 mmol, 1.00 equiv.), lauroyl chloride (52 µL, 0.230 mmol, 1.50 equiv.) and silver hexafluoroantimonate (77.3 mg, 0.225 mmol, 1.50 equiv.) at –47 °C. The resulting crude material was subjected to flash column chromatography (silica gel, 0%-20% EtOAc in heptane) to obtain the title compound as a colorless oil (46.1 mg, 0.106 mmol, 70% yield). The product was obtained as a mixture of *Z*/*E* isomers in a ratio of 91:9 (determined based on the ^{1}H NMR of the crude reaction mixture). NMR peaks in ^{1}H NMR corresponding exclusively to the minor isomer are indicated with an asterisk.

^{1}H NMR (400 MHz, CDCl$_3$): δ 7.78 (d, J = 8.3 Hz, 2H), 7.35 (d, J = 8.0 Hz, 2H), 5.66 – 5.54 (m, 1H), 5.52 – 5.42 (m, 1H), 4.02 (t, J = 6.2 Hz, 2H), 3.13 (d, J = 7.1 Hz, 1.84H), 3.05 (d, J = 6.5 Hz, 0.16H*), 2.50 – 2.36 (m, 5H), 2.09 (q, J = 7.2 Hz, 2H), 1.78 – 1.67 (m, 2H), 1.61 – 1.49 (m, 2H), 1.33 – 1.20 (m, 16H), 0.88 (t, J = 6.8 Hz, 3H) ppm.

^{13}C NMR (101 MHz, CDCl$_3$): δ 208.9, 144.9, 133.3, 131.0, 130.0 (2C), 128.0 (2C), 123.0, 69.9, 42.7, 41.4, 32.1, 29.8 (2C), 29.63, 29.56, 29.49, 29.38, 28.6, 23.9, 23.5, 22.8, 21.8, 14.3 ppm.

IR (neat) ν$_{max}$: 2923, 2853, 1738, 1716, 1598, 1456, 1363, 1216, 1189, 1175, 969, 927, 741, 689, 664, 575 cm^{-1}.

HRMS (ESI$^+$): exact mass calculated for $[M+Na]^+$ ($C_{25}H_{40}{}^{32}SO_4{}^{23}Na)^+$ requires *m*/*z* 459.2540, found *m*/*z* 459.2541.

Scale-up procedure for synthesis of (*Z*)-7-Oxooctadec-4-en-1-yl 4-methylbenzenesulfonate (5g)

A flame-dried Schlenk tube was charged with a solution of vinyl silane **2j** (0.805 g, 1.50 mmol, 1.00 equiv.) in anhydrous CH_2Cl_2 (7.5 mL) at room temperature (25 °C). To the solution, lauroyl chloride (0.52 mL, 2.3 mmol, 1.5 equiv.) was added in one portion at the same temperature. The reaction vessel was placed in a cooling bath at –47 °C (acetonitrile/dry ice cooling-bath) and allowed to stir for 1 min. In a separate flask, silver hexafluoroantimonate (0.773 g, 2.25 mmol, 1.50 equiv.) was dissolved in CH_2Cl_2 (7.5 mL) at room temperature under argon, and the obtained solution was then added dropwise to the pre-cooled vinyl silane and acyl chloride mixture. The reaction mixture was stirred at –47 °C for 2 h and then quenched with sat. aq. $NaHCO_3$ (7.5 mL) at the same temperature. The flask was immediately removed from the cooling bath and allowed to warm to room temperature over 15 min with vigorous stirring. The reaction mixture was filtered through celite, washing with CH_2Cl_2 (30 mL). The filtrate was transferred to a separatory funnel and the organic layer was separated. The aqueous layer was further extracted with CH_2Cl_2 (2 × 10 mL), the organic layers were combined, washed with brine and dried over anhydrous Na_2SO_4. After filtration, volatiles were removed *in vacuo*, and the crude mixture was purified by flash column chromatography (silica gel, 0%-20% EtOAc in heptane) to obtain the title compound as a pale-yellow oil (0.438 g, 1.00 mmol, 67% yield). The product was obtained as a mixture of *Z*/*E* isomers in a ratio of 91:9 (determined based on the ^{1}H NMR of the crude reaction mixture).

The analytical data is consistent with those obtained for this compound on 0.15 mmol scale (see page S42).

(±)-3-((2*S*,3*R*)-3-(2-Oxotridecyl)oxiran-2-yl)propyl 4-methylbenzenesulfonate (6)

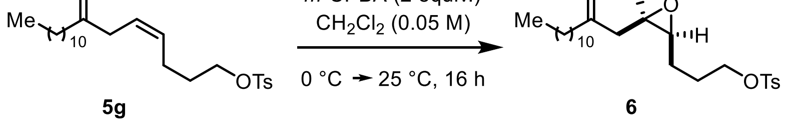

Deconjugated (*Z*)-enone **5g** (37.3 mg, 85.4 µmol, 1.00 equiv., 92:8 *Z*:*E*) was dissolved in CH_2Cl_2 (1.7 mL) and the solution was cooled to 0 °C. 3-Chloroperoxybenzoic acid (*m*-CPBA, 77% purity, 38 mg, 0.170 mmol, 2.00 equiv.) was then added in one portion and the obtained mixture was allowed to warm to room temperature (25 °C) and

then stirred for 16 h before being quenched with sat. aq. $Na_2S_2O_3$ (1 mL). The mixture was diluted with water (3 mL) and CH_2Cl_2 (2 mL). The organic layer was separated and the aqueous layer was extracted with CH_2Cl_2 (2 × 4 mL). The organic layers were combined, washed with brine (10 mL) and dried over Na_2SO_4. After filtration, volatiles were removed *in vacuo*, and the crude mixture was purified by flash column chromatography (silica gel, 0%-35% EtOAc in heptane) to obtain the desired product as a colorless oil (27.7 mg, 0.061 mmol, 72% yield). The product was isolated as a single diastereoisomer (d.r. >20:1).

^{1}H NMR (400 MHz, CDCl$_3$): δ 7.82 – 7.75 (m, 2H), 7.38 – 7.32 (m, 2H), 4.14 – 4.02 (m, 2H), 3.32 – 3.35 (m, 1H), 2.96 – 2.89 (m, 1H), 2.67 (dd, J = 17.3, 6.5 Hz, 1H), 2.55 (dd, J = 17.4, 5.5 Hz, 1H), 2.49 – 2.42 (m, 5H), 1.91 – 1.76 (m, 2H), 1.69 – 1.53 (m, 3H), 1.51 – 1.39 (m, 1H), 1.33 – 1.22 (m, 16H), 0.88 (t, J = 6.5 Hz, 3H) ppm.

^{13}C NMR (101 MHz, CDCl$_3$): δ 208.3, 145.0, 133.2, 130.0 (2C), 128.0 (2C), 69.9, 55.7, 52.5, 43.5, 41.5, 32.1, 29.8 (2C), 29.6, 29.54, 29.48, 29.3, 26.2, 24.3, 23.7, 22.8, 21.8, 14.3 ppm.

IR (neat) ν_{max}: 2924, 2853, 1715, 1464, 1359, 1188, 1176, 1098, 964, 929, 815, 664, 577, 555 cm^{-1}.

HRMS (ESI$^+$): exact mass calculated for $[M+Na]^+$ $(C_{25}H_{40}O_5{}^{32}S^{23}Na)^+$ requires *m*/*z* 475.2489, found *m*/*z* 475.2499.

(*Z*)-Dimethyl (7-oxooctadec-4-en-1-yl) phosphate (5h)

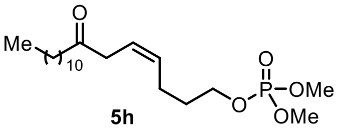

5h

General procedure **GP3** was followed using vinyl silane **2k** (65.3 mg, 0.133 mmol, 1.00 equiv.), lauroyl chloride (46 µL, 0.200 mmol, 1.50 equiv.) and silver hexafluoroantimonate (68.6 mg, 0.200 mmol, 1.50 equiv.) at –47 °C. The resulting crude material was subjected to flash column chromatography (silica gel, 40%-75% EtOAc in heptane) to obtain the title compound as a pale-yellow oil (29.1 mg, 0.075 mmol, 56% yield). The product was obtained as a mixture of *Z*/*E* isomers in a ratio of >95:5 (determined based on the ^{1}H NMR of the crude reaction mixture).

^{1}H NMR (400 MHz, CDCl$_3$): δ 5.69 – 5.48 (m, 2H), 4.05 (q, J = 6.5 Hz, 2H), 3.77 (d, J = 11.1 Hz, 6H), 3.17 (d, J = 6.5 Hz, 2H), 2.42 (t, J = 7.4 Hz, 2H), 2.22 – 2.08 (m, 2H), 1.82 – 1.70 (m, 2H), 1.65 – 1.49 (m, 2H), 1.35 – 1.16 (m, 16H), 0.87 (t, J = 6.8 Hz, 3H) ppm.

^{13}C NMR (101 MHz, CDCl$_3$): δ 209.0, 131.6, 122.6, 67.3 (d, J = 5.9 Hz), 54.4 (d, J = 6.1 Hz, 2C), 42.7, 41.5, 32.0, 30.0 (d, J = 6.7 Hz), 29.8 (2C), 29.62, 29.56, 29.5, 29.4, 23.9, 23.6, 22.8, 14.3 ppm.

^{31}P NMR (162 MHz, CDCl$_3$): δ 1.42 (s, 1P).

IR (neat) ν_{max}: 2923, 2853, 1715, 1464, 1279, 1187, 1034, 910, 847, 734, 506 cm^{-1}.

HRMS (ESI$^+$): exact mass calculated for $[M+Na]^+$ $(C_{20}H_{39}O_5P^{23}Na)^+$ requires *m*/*z* 413.2427, found *m*/*z* 413.2419.

Methyl (*Z*)-4-((7-oxooctadec-4-en-1-yl)oxy)benzoate (5i)

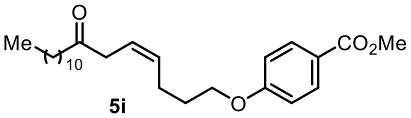

5i

General procedure **GP3** was followed using vinyl silane **2l** (77.5 mg, 0.150 mmol, 1.00 equiv.), lauroyl chloride (52 µL, 0.230 mmol, 1.50 equiv.) and silver hexafluoroantimonate (77.3 mg, 0.225 mmol, 1.50 equiv.) at –47 °C. The resulting crude material was subjected to flash column chromatography (silica gel, 0%-15% EtOAc in heptane) to obtain the title compound as a colorless oil (38.0 mg, 0.091 mmol, 61% yield). The product was obtained as a mixture of *Z*/*E* isomers in a ratio of 91:9 (determined based on the ^{1}H NMR of the crude reaction mixture). NMR peaks in ^{1}H NMR corresponding exclusively to the minor isomer are indicated with an asterisk.

^{1}H NMR (400 MHz, CDCl$_3$): δ 8.05 – 7.91 (m, 2H), 6.93 – 6.85 (m, 2H), 5.70 – 5.53 (m, 2H), 3.99 (t, *J* = 6.2 Hz, 2H), 3.88 (s, 3H), 3.20 – 3.12 (m, 1.81H), 3.10 (d, *J* = 5.4 Hz, 0.19H*), 2.34 (t, *J* = 7.5 Hz, 2H), 2.29 – 2.20 (m, 2H), 1.95 – 1.82 (m, 2H), 1.56 – 1.44 (m, 2H), 1.34 – 1.16 (m, 16H), 0.88 (t, *J* = 6.8 Hz, 3H) ppm.

^{13}C NMR (101 MHz, CDCl$_3$): δ 209.1, 167.0, 162.9, 131.9, 131.8 (2C), 122.7 (2C), 114.2 (2C), 67.1, 52.0, 42.6, 41.5, 32.1, 29.8 (2C), 29.62, 29.56, 29.5, 29.4, 28.7, 23.91, 23.90, 22.8, 14.3 ppm.

IR (neat) ν_{max}: 2925, 2853, 1718, 1606, 1511, 1435, 1279, 1254, 1168, 1105, 771, 735 cm^{-1}.

HRMS (ESI$^+$): exact mass calculated for $[M+Na]^+$ $(C_{26}H_{40}O_4{}^{23}Na)^+$ requires *m*/*z* 439.2819, found *m*/*z* 439.2819.

(*Z*)-*N*,*N*-Dimethyl-12-oxotricos-9-enamide (5j)

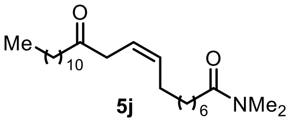

5j

General procedure **GP3** was followed using vinyl silane **2m** (62.8 mg, 0.127 mmol, 1.00 equiv.), lauroyl chloride (44 µL, 0.190 mmol, 1.50 equiv.) and silver hexafluoroantimonate (65.5 mg, 0.191 mmol, 1.50 equiv.) at –47 °C. The resulting crude material was subjected to flash column chromatography (silica gel, 0%-50% EtOAc in heptane) to obtain the title compound as a colorless oil (23.8 mg, 0.061 mmol, 48% yield). The product was obtained as a mixture of *Z*/*E* isomers in a ratio of 90:10 (determined based on the ^{1}H NMR of the crude reaction mixture). NMR peaks in ^{1}H NMR corresponding exclusively to the minor isomer are indicated with an asterisk.

^{1}H NMR (400 MHz, CDCl$_3$): δ 5.63 – 5.49 (m, 2H), 3.14 (d, *J* = 6.3 Hz, 1.80H), 3.08 (d, *J* = 5.2 Hz, 0.20H*), 3.00 (s, 3H), 2.94 (s, 3H), 2.46 – 2.37 (m, 2H), 2.33 – 2.26 (m, 2H), 2.07 – 1.95 (m, 2H), 1.67 – 1.51 (m, 4H), 1.38 – 1.21 (m, 24H), 0.87 (t, *J* = 6.8 Hz, 3H).

^{13}C NMR (101 MHz, CDCl$_3$): δ 209.5, 173.3, 133.7, 121.1, 42.5, 41.8, 37.4, 35.5, 33.5, 32.1, 29.8 (2C), 29.62, 29.59, 29.56, 29.5 (2C), 29.45, 29.38, 29.3, 27.6, 25.3, 24.0, 22.8, 14.3 ppm.

IR (neat) ν_{max}: 2924, 2853, 2359, 1715, 1651, 1465, 1396, 1267, 1150, 738 cm^{-1}.

HRMS (ESI$^+$): exact mass calculated for $[M+Na]^+$ $(C_{25}H_{47}NO_2{}^{23}Na)^+$ requires *m/z* 416.3499, found *m/z* 416.3498.

Ethyl (2*E*,11*Z*)-14-oxopentacosa-2,11-dienoate (5k)

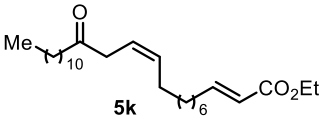

5k

General procedure **GP3** was followed using vinyl silane **2n** (52.9 mg, 0.150 mmol, 1.00 equiv.), lauroyl chloride (49.2 mg, 52 μL, 0.225 mmol, 1.50 equiv.) and silver hexafluoroantimonate (77.3 mg, 0.225 mmol, 1.50 equiv.) at –61 °C. The resulting crude material was subjected to flash column chromatography (silica gel, 0%-20% EtOAc in heptane) to obtain the title compound as a colorless oil (58.0 mg, 0.138 mmol, 92% yield). The product was obtained as a mixture of *Z*/*E* isomers in a ratio of 90:10 (determined based on the ^{1}H NMR of the crude reaction mixture). NMR peaks in ^{1}H NMR corresponding exclusively to the minor isomer are indicated with an asterisk.

^{1}H NMR (400 MHz, CDCl$_3$): δ 6.94 (dt, *J* = 15.6, 7.0 Hz, 1H), 5.79 (dt, *J* = 15.6, 1.5 Hz, 1H), 5.61 – 5.48 (m, 2H), 4.17 (q, *J* = 7.1 Hz, 2H), 3.13 (d, *J* = 6.1 Hz, 1.80H), 3.07 (d, *J* = 5.1 Hz, 0.20H*), 2.43 – 2.37 (m, 2H), 2.22 – 2.14 (m, 2H), 2.01 (q, *J* = 6.6 Hz, 2H), 1.60 – 1.50 (m, 2H), 1.47 – 1.39 (m, 2H), 1.37 – 1.20 (m, 27H), 0.86 (t, *J* = 6.9 Hz, 3H) ppm.

^{13}C NMR (101 MHz, CDCl$_3$): δ 209.3, 166.9, 149.5, 133.6, 121.4, 121.1, 60.2, 42.5, 41.8, 32.3, 32.0, 29.7 (2C), 29.6, 29.5 (2C), 29.4 (2C), 29.3 (2C), 29.2, 28.1, 27.6, 23.9, 22.8, 14.4, 14.2 ppm.

IR (neat) ν_{max}: 2923, 2853, 1718, 1654, 1464, 1264, 1179, 1044, 721 cm^{-1}.

HRMS (ESI$^+$): exact mass calculated for $[M+H]^+$ $(C_{27}H_{49}O_3)^+$ requires *m/z* 421.3676, found *m/z* 421.3673.

Methyl (*Z*)-8-oxononadec-5-enoate (5l)

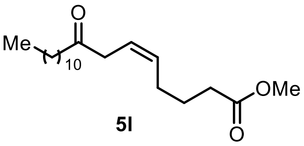

5l

General procedure **GP3** was followed using vinyl silane **2ba** (51.3 mg, 0.200 mmol, 1.00 equiv.), lauroyl chloride (66 mg, 69 μL, 0.300 mmol, 1.50 equiv.) and silver hexafluoroantimonate (103 mg, 0.300 mmol, 1.50 equiv.) at –61 °C. The resulting crude material was subjected to flash column chromatography (silica gel, 0%-12% EtOAc in heptane) to obtain the title compound as a pale-yellow oil (40.4 mg, 0.124 mmol, 62% yield). The product was obtained as a mixture of *Z*/*E* isomers in a ratio of 95:5 (determined based on the ^{1}H NMR of the crude reaction mixture). NMR peaks in ^{1}H NMR corresponding exclusively to the minor isomer are indicated with an asterisk.

^{1}H NMR (400 MHz, CDCl$_3$): δ 5.66 – 5.47 (m, 2H), 3.67 (s, 3H), 3.15 (d, *J* = 6.3 Hz, 1.90H), 3.09 (d, *J* = 6.3 Hz, 0.10H*), 2.42 (t, *J* = 7.5 Hz, 2H), 2.32 (t, *J* = 7.4 Hz, 2H), 2.14 – 2.03 (m, 2H), 1.77 – 1.66 (m, 2H), 1.61 – 1.51 (m, 2H), 1.33 – 1.20 (m, 16H), 0.88 (t, *J* = 6.6 Hz, 3H) ppm.

^{13}C NMR (101 MHz, CDCl$_3$): δ 209.1, 174.1, 132.2, 122.4, 51.7, 42.6, 41.6, 33.5, 32.1, 29.8 (2C), 29.62, 29.56, 29.5, 29.4, 26.9, 24.6, 24.0, 22.8, 14.3 ppm.

IR (neat) ν$_{max}$: 2925, 2854, 1739, 1717, 1437, 1366, 1203, 1160, 910, 733, 650, 441, 429, 408 cm^{-1}.

HRMS (ESI$^+$): exact mass calculated for $[M+Na]^+$ $(C_{20}H_{36}O_3Na)^+$ requires *m*/*z* 347.2557, found *m*/*z* 347.2547.

Methyl (*Z*)-8-oxononadec-5-enoate (5l) from trihexylvinyl silane 2bb

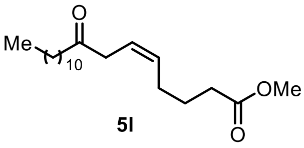

5l

General procedure **GP3** was followed using vinyl silane **2bb** (85.0 mg, 0.200 mmol, 1.00 equiv.), lauroyl chloride (65.6 mg, 69.4 µL, 0.300 mmol, 1.50 equiv.) and silver hexafluoroantimonate (103 mg, 0.300 mmol, 1.50 equiv.) at –61 °C. The resulting crude material was subjected to flash column chromatography (silica gel, 0%-12% EtOAc in heptane) to obtain the title compound as a pale-yellow oil (33.2 mg, 0.102 mmol, 51% yield). The product was obtained as a mixture of *Z*/*E* isomers in a ratio of 94:6 (determined based on the ^{1}H NMR of the crude reaction mixture).

The analytical data is consistent with those obtained for this compound from vinyl silane **2ba** (see page S46).

(*S*)-1-(4-Fluorophenyl)-3-((2*S*,3*R*)-1-(4-fluorophenyl)-2-(4-methoxyphenyl)-4-oxoazetidin-3-yl)propyl (*Z*)-12-oxotricos-9-enoate (5m)

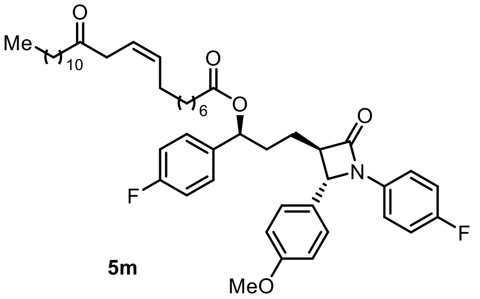

5m

General procedure **GP3** was followed using vinyl silane **2o** (87.2 mg, 0.100 mmol, 1.00 equiv.), lauroyl chloride (32.8 mg, 34.7 µL, 0.150 mmol, 1.50 equiv.) and silver hexafluoroantimonate (51.5 mg, 0.150 mmol, 1.50 equiv.) at –61 °C. The resulting crude material was subjected to flash column chromatography (silica gel, 0%-40% EtOAc in heptane) to obtain the title compound as a colorless oil (56.9 mg, 0.074 mmol, 74% yield). The product was obtained as a mixture of *Z*/*E* isomers in a ratio of >95:5 (determined based on the ^{1}H NMR of the crude reaction mixture).

^{1}H NMR (400 MHz, CDCl$_3$): δ 7.28 – 7.19 (m, 6H), 7.00 (t, *J* = 8.7 Hz, 2H), 6.94 – 6.88 (m, 4H), 5.70 (t, *J* = 6.7 Hz, 1H), 5.60 – 5.48 (m, 2H), 4.54 (d, *J* = 2.3 Hz, 1H), 3.80 (s, 3H), 3.14 (d, *J* = 5.6 Hz, 2H), 3.06 (td, *J* = 7.7, 2.3 Hz, 1H), 2.41 (t, *J* = 7.4 Hz, 2H), 2.29 (t, *J* = 7.7 Hz, 2H), 2.06 – 1.96 (m, 4H), 1.89 – 1.81 (m, 2H), 1.64 – 1.51 (m, 4H), 1.34 – 1.22 (m, 24H), 0.87 (t, *J* = 6.9 Hz, 3H) ppm.

^{13}C NMR (101 MHz, CDCl$_3$): δ 209.4, 173.1, 167.2, 162.5 (d, J = 246.6 Hz), 160.0, 159.1 (d, J = 243.4 Hz), 136.1 (d, J = 3.2 Hz), 134.0 (d, J = 2.6 Hz), 133.6, 129.4, 128.3 (d, J = 8.2 Hz, 2C), 127.3 (2C), 121.2, 118.5 (d, J = 7.7 Hz, 2C), 115.9 (d, J = 22.7 Hz, 2C), 115.6 (d, J = 21.5 Hz, 2C), 114.8 (2C), 74.7, 61.1, 60.2, 55.5, 42.5, 41.8, 34.5, 33.8 (2C), 32.0, 29.7, 29.6, 29.5 (2C), 29.4 (2C) 29.2 (2C), 29.1, 27.6, 25.0 (2C) 24.0, 22.8, 14.3 ppm.

^{19}F NMR (376 MHz, CDCl$_3$): δ -113.85 – -113.95 (m, 1F), -118.00 – -118.11 (m, 1F) ppm.

IR (neat) ν_{max}: 2925, 2854, 1740, 1509, 1249, 1225, 1033, 832, 730 cm^{-1}.

HRMS (ESI$^+$): exact mass calculated for [M+Na]$^+$ ($C_{48}H_{63}F_2NO_5{}^{23}Na$)$^+$ requires *m/z* 794.4567, found *m/z* 794.4559.

[α]$_D^{24}$ = -18.6 (c = 0.73, CHCl$_3$).

(*Z*)-6-Butyloctadec-4-en-7-one (5n)

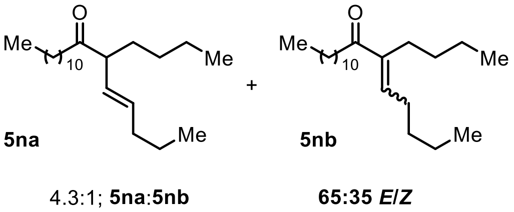

General procedure **GP3** was followed using vinyl silane **2p** (63.4 mg, 0.150 mmol, 1.00 equiv.), lauroyl chloride (49.2 mg, 52.0 μL, 0.225 mmol, 1.50 equiv.) and silver hexafluoroantimonate (77.3 mg, 0.225 mmol, 1.50 equiv.) at –78 °C. The resulting crude material was subjected to flash column chromatography (silica gel, 0%-2% EtOAc in heptane) to obtain the title compound as a colorless oil (47.3 mg, 0.147 mmol, 98% yield). The product was obtained as an inseparable mixture of two regioisomers in a ratio of 4.3:1. Major regioisomer was formed as a *E*-isomer (*E*/*Z* ratio >95:5). Minor regioisomer was formed as a mixture of *E*/*Z* isomers in a ratio of 65:35 (determined based on the ^{1}H NMR of the crude reaction mixture).

Major isomer (from the mixture): **^{1}H NMR (400 MHz, CDCl$_3$):** δ 5.60 – 5.45 (m, 1H), 5.35 – 5.19 (m, 1H), 3.05 – 2.93 (m, 1H), 2.50 – 2.31 (m, 2H), 1.99 (q, J = 7.2 Hz, 2H), 1.74 – 1.47 (m, 4H), 1.42 – 1.13 (m, 22H), 0.94 – 0.81 (m, 9H).

Major isomer (from the mixture): **^{13}C NMR (176 MHz, CDCl$_3$):** δ 212.4, 134.0, 128.6, 56.9, 41.5, 34.8, 32.1, 31.0, 29.8 (2C), 29.6 (3C), 29.5, 29.4, 23.8, 22.8, 22.7, 22.6, 14.2, 14.1, 13.7 ppm.

IR (neat) ν_{max}: 2956, 2923, 2854, 1714, 1464, 1378, 1096, 970, 722 cm^{-1}.

HRMS (ESI$^+$): exact mass calculated for [M+Na]$^+$ ($C_{22}H_{42}O^{23}Na$)$^+$ requires *m/z* 345.3128, found *m/z* 345.3125.

3.5 Unsuccessful and low-yielding substrates

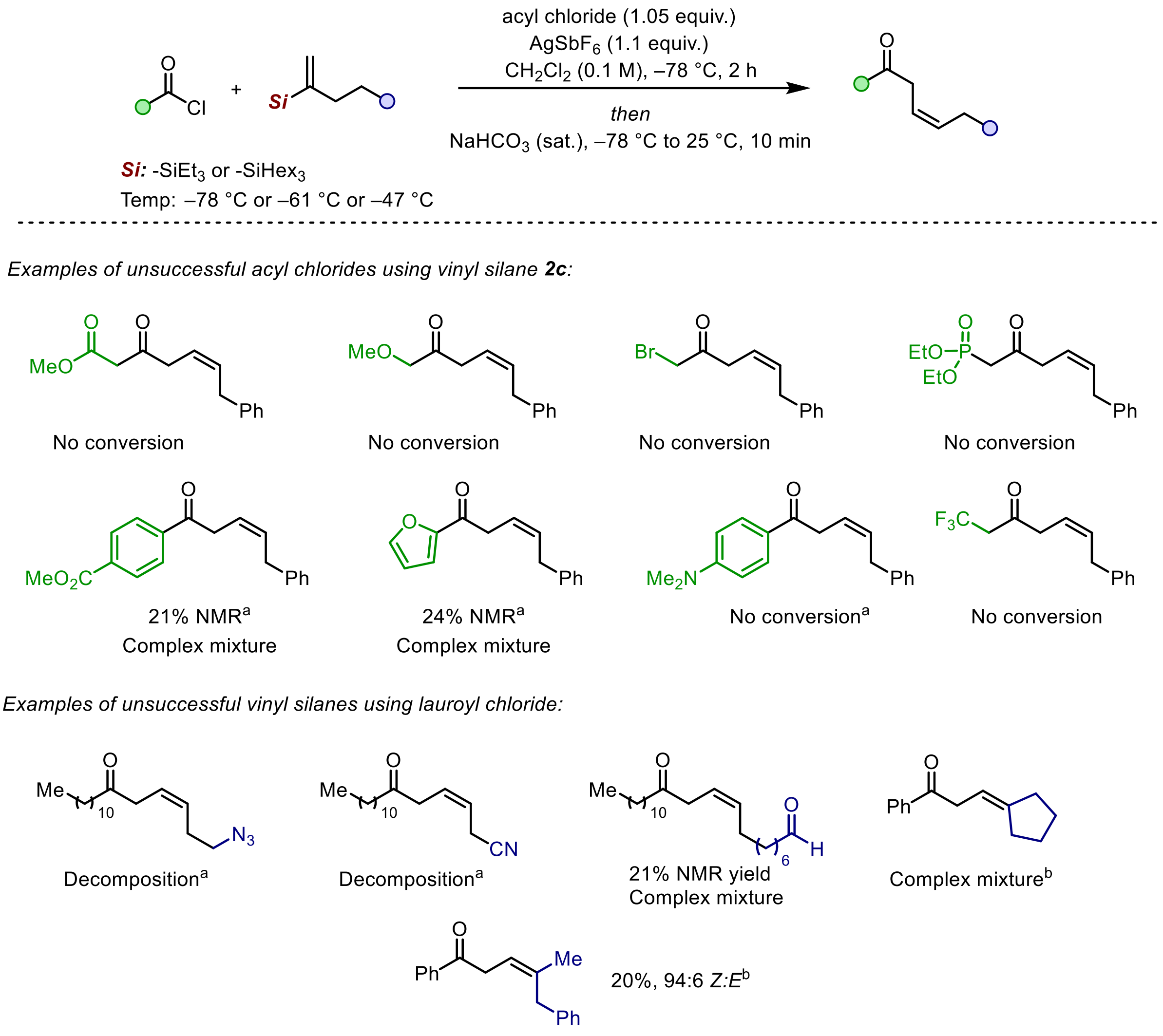

Scheme **S1**. Example of the unsuccessful substrates. a) Reaction performed at –47 °C with 1.5 equivalents of acid chloride and 1.5 equivalents of $AgSbF_6$, b) Reaction performed at –78 °C with 1.5 equivalents of benzoyl chloride and 1.5 equivalents of $AgSbF_6$,

4. Natural Products Synthesis

4.1 Fatty Acid Synthesis

Scheme S2: General overview of fatty acid synthesis

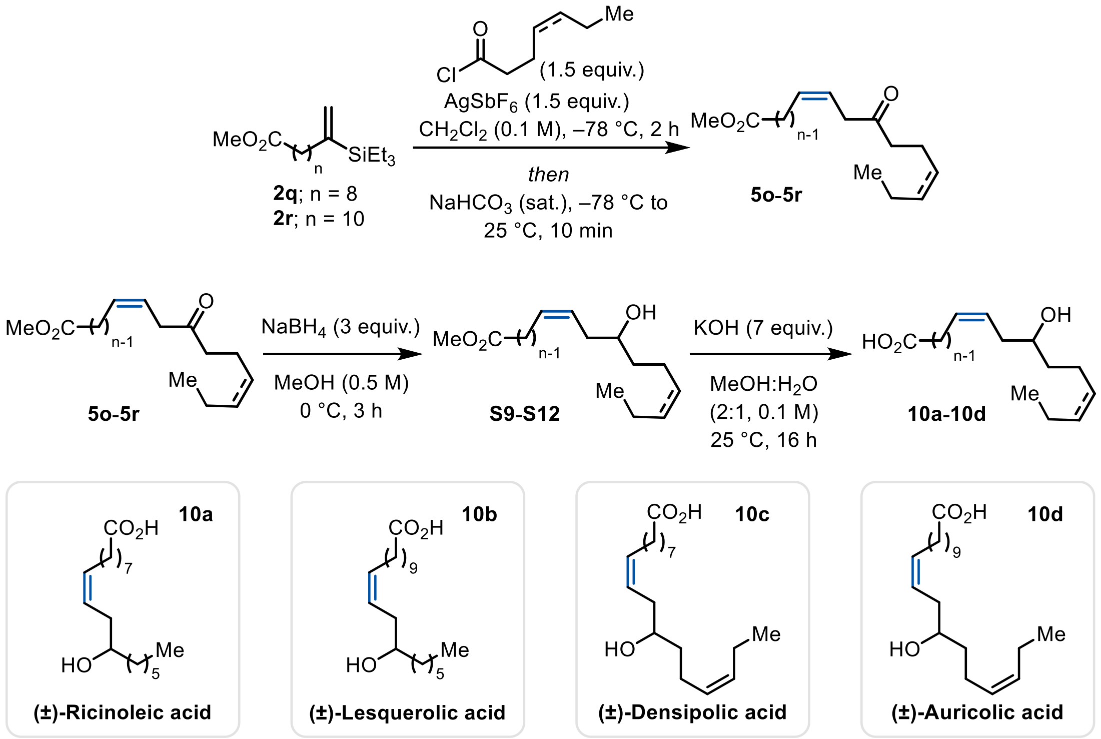

General procedure GP4: Synthesis of β,γ-Unsaturated Enones *via* Charge Relocation:

A flame-dried Schlenk tube or oven-dried vial was charged with a solution of vinyl silane (1.0 equiv.) in anhydrous CH_2Cl_2 (0.2 M) at room temperature. To the solution, heptanoyl chloride (1.5 equiv.) was added in one portion at the same temperature. The reaction vessel was placed in a –78 °C cooling bath (acetone/dry ice cooling-bath) and allowed to stir for 1 min. In a separate vial, silver hexafluoroantimonate ($AgSbF_6$, 1.5 equiv.) was dissolved in CH_2Cl_2 (equal volume to the vinyl silane solution) at room temperature, and the obtained solution was then added dropwise to the pre-cooled vinyl silane and heptanoyl chloride mixture to obtain overall 0.1 M solution of the vinyl silane. The reaction mixture was stirred at –78 °C for 2 h and then quenched with sat. aq. $NaHCO_3$ (equal volume to CH_2Cl_2) at the same temperature. The vial was immediately removed from the cooling bath and allowed to warm to room temperature over 10 min with vigorous stirring. The reaction mixture was partitioned between CH_2Cl_2 and water, and the organic layer was separated. The aqueous layer was extracted with CH_2Cl_2 (2 × 20 mL), the organic layers were combined, washed with brine and dried over anhydrous Na_2SO_4. After filtration, volatiles were removed *in vacuo*, and the crude mixture was purified by flash column chromatography (silica gel) using the appropriate mixture of eluents.

General procedure GP5: Reduction of β,γ-Unsaturated Enones with Sodium Borohydride:

To a stirred solution of ketone (1.0 equiv.) in MeOH (0.5 M), sodium borohydride ($NaBH_4$, 3.0 equiv.) was added at 0 °C in one portion. The obtained mixture was stirred at 0 °C for 3 h and then quenched by the addition of 1 M HCl_{aq} (equal volume to MeOH) followed by water (equal volume to MeOH). The mixture was extracted with EtOAc (3 × 5 mL), the organic layers were combined, washed with brine (15 mL), dried over anhydrous Na_2SO_4, filtered and concentrated *in vacuo*. The residue was purified by flash column chromatography (silica gel) using the appropriate mixture of eluents.

General procedure GP6: Saponification of Methyl Esters to Fatty Acids:

To a 10 mL round-bottom flask charged with solution of methyl ester (1.0 equiv.) in the mixture of MeOH and H_2O (2:1, 0.1 M), potassium hydroxide (KOH, 7 equiv.) was added at room temperature (25 °C) and stirred for 16 h. After completion of the reaction, the mixture was acidified with 1M HCl_{aq} to pH 1–2, transferred to a separatory funnel and extracted with EtOAc (2 × 5 mL). Combined organic phases were dried over anhydrous Na_2SO_4, filtered and concentrated *in vacuo*. The crude mixture was purified by flash column chromatography (silica gel) using the appropriate mixture of eluents.

Methyl (*Z*)-12-oxooctadec-9-enoate (5o)

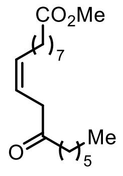

5o

General procedure **GP4** was followed using vinyl silane **2q** (62.5 mg, 0.200 mmol, 1.00 equiv.), heptanoyl chloride (46 µL, 0.300 mmol, 1.50 equiv.) and silver hexafluoroantimonate (103 mg, 0.300 mmol, 1.50 equiv.) at –78 °C. The resulting crude material was subjected to flash column chromatography (silica gel, 0%-10% EtOAc in heptane) to obtain the title compound as a pale-yellow oil (40.5 mg, 0.130 mmol, 65% yield). The product was obtained as a mixture of *Z*/*E* isomers in a ratio of 88:12 (determined based on the ^{1}H NMR of the crude reaction mixture). NMR peaks in ^{1}H NMR corresponding exclusively to the minor isomer are indicated with an asterisk.

^{1}H NMR (400 MHz, $CDCl_3$): δ 5.63 – 5.47 (m, 2H), 3.66 (s, 3H), 3.14 (d, *J* = 6.1 Hz, 1.75H), 3.08 (d, *J* = 5.2 Hz, 0.25H*), 2.47 – 2.38 (m, 2H), 2.30 (t, *J* = 7.5 Hz, 2H), 2.02 (q, *J* = 6.7 Hz, 2H), 1.66 – 1.52 (m, 4H), 1.40 – 1.22 (m, 14H), 0.93 – 0.82 (m, 3H) ppm.

^{13}C NMR (101 MHz, $CDCl_3$): δ 209.4, 174.4, 133.7, 121.2, 51.6, 42.5, 41.8, 34.2, 31.7, 29.4, 29.3, 29.2 (2C), 29.0, 27.6, 25.1, 23.9, 22.7, 14.2 ppm.

IR (neat) ν_{max}: 2927, 2855, 1738, 1715, 1459, 1436, 1362, 1196, 1170, 1072, 1015, 844, 725 cm^{-1}.

HRMS (ESI$^+$): exact mass calculated for $[M+Na]^+$ $(C_{19}H_{34}O_3Na)^+$ requires *m*/*z* 333.2400, found *m*/*z* 333.2397.

Methyl (*Z*)-14-oxoicos-11-enoate (5p)

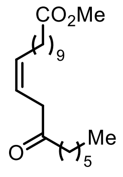

5p

General procedure **GP4** was followed using vinyl silane **2r** (68.1 mg, 0.200 mmol, 1.00 equiv.), heptanoyl chloride (46 µL, 0.300 mmol, 1.50 equiv.) and silver hexafluoroantimonate (103 mg, 0.300 mmol, 1.50 equiv.) at –78 °C. The resulting crude material was subjected to flash column chromatography (silica gel, 0%-10% EtOAc in heptane) to obtain the title compound as a pale-yellow oil (43.7 mg, 0.129 mmol, 65% yield). The product was obtained as a mixture of *Z*/*E* isomers in a ratio of 88:12 (determined based on the ^{1}H NMR of the crude reaction mixture). NMR peaks in ^{1}H NMR corresponding exclusively to the minor isomer are indicated with an asterisk.

^{1}H NMR (400 MHz, CDCl$_3$): δ 5.66 – 5.44 (m, 2H), 3.66 (s, 3H), 3.15 (d, *J* = 6.4 Hz, 1.77H*), 3.08 (d, *J* = 5.2 Hz, 0.23H*), 2.47 – 2.37 (m, 2H), 2.30 (t, *J* = 7.5 Hz, 2H), 2.02 (q, *J* = 6.9 Hz, 2H), 1.68 – 1.51 (m, 4H), 1.39 – 1.22 (m, 18H), 0.92 – 0.82 (m, 3H) ppm.

^{13}C NMR (101 MHz, CDCl$_3$): δ 209.5, 174.5, 133.8, 121.1, 51.6, 42.5, 41.8, 34.3, 31.8, 29.6, 29.53, 29.48, 29.40, 29.38, 29.3, 29.1, 27.7, 25.1, 23.9, 22.6, 14.2 ppm.

IR (neat) ν_{max}: 2926, 2855, 1740, 1717, 1461, 1436, 1364, 1196, 1172, 911, 733 cm^{-1}.

HRMS (ESI$^+$): exact mass calculated for $[M+Na]^+$ $(C_{21}H_{38}O_3{}^{23}Na)^+$ requires *m*/*z* 361.2713, found *m*/*z* 361.2704.

Methyl (9*Z*,15*Z*)-12-oxooctadeca-9,15-dienoate (5q)

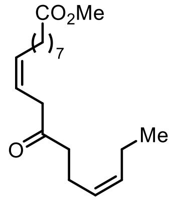

5q

General procedure **GP4** was followed using vinyl silane **2q** (46.9 mg, 0.150 mmol, 1.00 equiv.), acyl chloride **S2** (33.0 mg, 0.225 mmol, 1.50 equiv.) and silver hexafluoroantimonate (77.3 mg, 0.225 mmol, 1.50 equiv.) at –78 °C. The resulting crude material was subjected to flash column chromatography (silica gel, 0%-10% EtOAc in heptane) to obtain the title compound as a colorless oil (29.6 mg, 0.096 mmol, 64% yield). The product was obtained as a mixture of *Z*/*E* isomers in a ratio of 89:11 (determined based on the ^{1}H NMR of the crude reaction mixture). NMR peaks in ^{1}H NMR corresponding exclusively to the minor isomer are indicated with an asterisk.

^{1}H NMR (400 MHz, CDCl$_3$): δ 5.63 – 5.47 (m, 2H), 5.42 – 5.34 (m, 1H), 5.31 – 5.21 (m, 1H), 3.65 (s, 3H), 3.14 (d, *J* = 6.4 Hz, 1.77H), 3.08 (d, *J* = 5.3 Hz, 0.23H*), 2.47 (t, *J* = 7.4 Hz, 2H), 2.29 (t, *J* = 7.5 Hz, 4H), 2.08 – 1.97 (m, 4H), 1.65 – 1.56 (m, 2H), 1.38 – 1.24 (m, 8H), 0.94 (t, *J* = 7.5 Hz, 3H) ppm.

^{13}C NMR (101 MHz, CDCl$_3$): δ 208.6, 174.4, 133.8, 133.0, 127.3, 121.0, 51.6, 42.4, 41.9, 34.2, 29.4, 29.24, 29.20, 29.18, 27.6, 25.0, 21.7, 20.6, 14.4 ppm.

IR (neat) ν_{max}: 2928, 2855, 1738, 1716, 1436, 1359, 1197, 1081, 723 cm^{-1}.

HRMS (ESI$^+$): exact mass calculated for $[M+Na]^+$ $(C_{19}H_{32}O_3{}^{23}Na)^+$ requires *m/z* 331.2244, found *m/z* 331.2234.

Methyl (11*Z*,17*Z*)-14-oxoicosa-11,17-dienoate (5r)

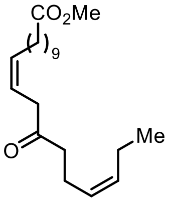

5r

General procedure **GP4** was followed using vinyl silane **2r** (68.1 mg, 0.200 mmol, 1.00 equiv.), acyl chloride **S2** (44.0 mg, 0.300 mmol, 1.50 equiv.) and silver hexafluoroantimonate (103.0 mg, 0.300 mmol, 1.50 equiv.) at –78 °C. The resulting crude material was subjected to flash column chromatography (silica gel, 0%-10% EtOAc in heptane) to obtain the title compound as a colorless oil (54.3 mg, 0.161 mmol, 81% yield). The product was obtained as a mixture of *Z*/*E* isomers in a ratio of 89:11 (determined based on the analysis of ^{1}H NMR after purification). NMR peaks in ^{1}H NMR corresponding exclusively to the minor isomer are indicated with an asterisk.

^{1}H NMR (400 MHz, CDCl$_3$): δ 5.62 – 5.48 (m, 2H), 5.42 – 5.34 (m, 1H), 5.31 – 5.21 (m, 1H), 3.65 (s, 3H), 3.14 (d, *J* = 6.5 Hz, 1.77H), 3.07 (d, *J* = 5.5 Hz, 0.23H*), 2.47 (t, *J* = 7.4 Hz, 2H), 2.29 (t, *J* = 7.5 Hz, 4H), 2.08 – 1.96 (m, 4H), 1.64 – 1.55 (m, 2H), 1.37 – 1.23 (m, 12H), 0.94 (t, *J* = 7.5 Hz, 3H) ppm.

^{13}C NMR (101 MHz, CDCl$_3$): δ 208.6, 174.4, 133.9, 133.0, 127.3, 120.9, 51.6, 42.3, 41.9, 34.2, 29.54, 29.49, 29.44, 29.36, 29.34, 29.25, 27.6, 25.1, 21.7, 20.6, 14.4 ppm.

IR (neat) ν_{max}: 2926, 2854, 1738, 1716, 1436, 1358, 1196, 1082, 722 cm^{-1}.

HRMS (ESI$^+$): exact mass calculated for $[M+Na]^+$ $(C_{21}H_{36}O_3{}^{23}Na)^+$ requires *m/z* 359.2557, found *m/z* 359.2555.

(±)-Methyl (*Z*)-12-hydroxyoctadec-9-enoate (S9)

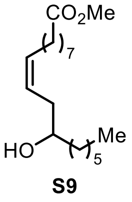

S9

General procedure **GP5** was followed using ketone **5o** (59.5 mg, 0.192 mmol, 1.00 equiv.) and sodium borohydride (21.7 mg, 0.575 mmol, 3.00 equiv.). The resulting crude material was subjected to flash column chromatography (silica gel, 0%-30% EtOAc in heptane) to obtain the title compound as a colorless oil (42.1 mg, 0.135 mmol, 70% yield). The product was isolated as a single *Z* isomer (>95:5 *Z*/*E*).

^{1}H NMR (400 MHz, CDCl$_3$): δ 5.61 – 5.48 (m, 1H), 5.46 – 5.35 (m, 1H), 3.75 – 3.53 (m, 4H), 2.30 (t, *J* = 7.5 Hz, 2H), 2.25 – 2.17 (m, 2H), 2.11 – 1.97 (m, 2H), 1.67 – 1.57 (m, 2H), 1.53 (s, 1H), 1.50 – 1.40 (m, 3H), 1.39 – 1.22 (m, 15H), 0.94 – 0.82 (m, 3H) ppm.

^{13}C NMR (101 MHz, CDCl$_3$): δ 174.5, 133.6, 125.4, 71.7, 51.6, 37.0, 35.5, 34.2, 32.0, 29.7, 29.5, 29.3, 29.2 (2C), 27.5, 25.9, 25.1, 22.8, 14.2 ppm.

IR (neat) ν_{max}: 3454, 2927, 2855, 1742, 1437, 1363, 1198, 722 cm^{-1}.

HRMS (ESI^{+}): exact mass calculated for [M+Na]$^{+}$ ($C_{19}H_{36}O_3Na$)$^{+}$ requires *m*/*z* 335.2557, found *m*/*z* 335.2563.

(±)-Methyl (*Z*)-14-hydroxyicos-11-enoate (S10)

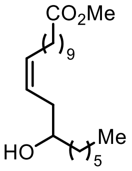

S10

General procedure **GP5** was followed using ketone **5p** (40.8 mg, 0.121 mmol, 1.00 equiv.) and sodium borohydride (13.7 mg, 0.362 mmol, 3.00 equiv.). The resulting crude material was subjected to flash column chromatography (silica gel, 0%-25% EtOAc in heptane) to obtain the title compound as a colorless oil (33.0 mg, 0.097 mmol, 80% yield). The product was obtained as a mixture of *Z*/*E* isomers in a ratio of 87:13 (determined based on the analysis of ^{1}H NMR after purification). NMR peaks in ^{1}H NMR corresponding exclusively to the minor isomer are indicated with an asterisk.

^{1}H NMR (600 MHz, CDCl$_3$): δ 5.61 – 5.49 (m, 1H), 5.44 – 5.36 (m, 1H), 3.69 – 3.55 (m, 4H), 2.30 (t, *J* = 7.6 Hz, 2H), 2.26 – 2.18 (m, 2H), 2.08 – 1.99 (m, 2H), 1.65 – 1.58 (m, 2H), 1.57 (d, *J* = 3.9 Hz, 0.13H*), 1.51 (d, *J* = 4.1 Hz, 0.87H), 1.50 – 1.40 (m, 3H), 1.38 – 1.22 (m, 19H), 0.92 – 0.85 (m, 3H) ppm.

^{13}C NMR (151 MHz, CDCl$_3$): δ 174.5, 133.7, 125.3, 71.7, 51.6, 37.0, 35.5, 34.3, 32.0, 29.8, 29.6, 29.53, 29.51, 29.42, 29.38, 29.3, 27.6, 25.9, 25.1, 22.8, 14.2 ppm.

IR (neat) ν_{max}: 3433, 2925, 2854, 1742, 1437, 1365, 1199, 1172, 1044, 911, 734 cm^{-1}.

HRMS (ESI^{+}): exact mass calculated for [M+Na]$^{+}$ ($C_{21}H_{40}O_3{}^{23}Na$)$^{+}$ requires *m*/*z* 363.2870, found *m*/*z* 363.2868.

(±)-Methyl (9*Z*,15*Z*)-12-hydroxyoctadeca-9,15-dienoate (S11)

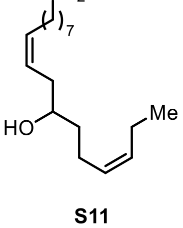

S11

General procedure **GP5** was followed using ketone **5q** (42.1 mg, 0.136 mmol, 1.00 equiv.) and sodium borohydride (15.5 mg, 0.409 mmol, 3.01 equiv.). The resulting crude material was subjected to flash column chromatography (silica gel, 0%-30% EtOAc in heptane) to obtain the title compound as a colorless oil (32.3 mg, 0.104 mmol, 77% yield). The product was obtained as a mixture of *Z*/*E* isomers in a ratio of 89:11 (determined based on the analysis of ^{13}C NMR after purification).

^{1}H NMR (400 MHz, CDCl$_3$): δ 5.60 – 5.49 (m, 1H), 5.44 – 5.29 (m, 3H), 3.70 – 3.57 (m, 4H), 2.29 (t, *J* = 7.5 Hz, 2H), 2.24 – 1.98 (m, 8H), 1.66 – 1.48 (m, 5H), 1.38 – 1.22 (m, 8H), 0.96 (t, *J* = 7.5 Hz, 3H) ppm.

^{13}C NMR (101 MHz, CDCl$_3$): δ 174.5, 133.6, 132.4, 128.7, 125.2, 71.3, 51.6, 36.8, 35.5, 34.2, 29.7, 29.25, 29.21 (2C), 27.5, 25.1, 23.7, 20.7, 14.5 ppm.

IR (neat) ν_{max}: 3458, 2926, 2854, 1740, 1436, 1197, 1171, 1066, 723 cm^{-1}.

HRMS (ESI$^+$): exact mass calculated for [M+Na]$^+$ ($C_{19}H_{34}O_3{}^{23}Na$)$^+$ requires *m/z* 333.2400, found *m/z* 333.2405.

(±)-Methyl (11*Z*,17*Z*)-14-hydroxyicosa-11,17-dienoate (S12)

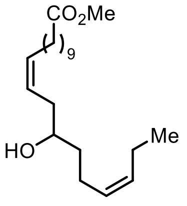

S12

General procedure **GP5** was followed using ketone **5r** (59.3 mg, 0.176 mmol, 1.00 equiv.) and sodium borohydride (20.0 mg, 0.529 mmol, 3.01 equiv.). The resulting crude material was subjected to flash column chromatography (silica gel, 0%-30% EtOAc in heptane) to obtain the title compound as a colorless oil (44.9 mg, 0.133 mmol, 75% yield). The product was obtained as a mixture of *Z*/*E* isomers in a ratio of 89:11 (determined based on the analysis of ^{13}C NMR after purification).

^{1}H NMR (400 MHz, CDCl$_3$): δ 5.60 – 5.48 (m, 1H), 5.43 – 5.28 (m, 3H), 3.69 – 3.57 (m, 4H), 2.29 (t, *J* = 7.6 Hz, 2H), 2.24 – 1.98 (m, 8H), 1.70 – 1.47 (m, 5H), 1.38 – 1.21 (m, 12H), 0.95 (t, *J* = 7.5 Hz, 3H) ppm.

^{13}C NMR (101 MHz, CDCl$_3$): δ 174.5, 133.6, 132.4, 128.7, 125.1, 71.3, 51.6, 36.8, 35.5, 34.2, 29.8, 29.6, 29.5, 29.39, 29.35, 29.3, 27.6, 25.1, 23.7, 20.6, 14.5 ppm.

IR (neat) ν_{max}: 3448, 2924, 2854, 1740, 1436, 1197, 1172, 1066, 721 cm^{-1}.

HRMS (ESI$^+$): exact mass calculated for [M+Na]$^+$ ($C_{21}H_{38}O_3{}^{23}Na$)$^+$ requires *m/z* 361.2713, found *m/z* 361.2710.

(±)-Ricinoleic acid (10a)

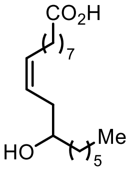

10a

General procedure **GP6** was followed using methyl ester **S9** (single *Z* isomer, 40.2 mg, 0.129 mmol, 1.00 equiv.) and potassium hydroxide (50 mg, 0.890 mmol, 7.00 equiv.). The resulting crude material was redissolved in Et_2O (5 mL) and potassium hydroxide (KOH, 2 M aq., 5 mL) was added to obtain pH > 12. The aqueous layer was separated, and the organic layer was washed with water (2 × 5 mL). The aqueous layers were combined, acidified with 1 M HCl_{aq} until pH < 2, and then extracted with EtOAc (3 × 10 mL). The organic layers were combined, washed with brine and dried over Na_2SO_4. After filtration, volatiles were removed *in vacuo* to obtain the product as a pale-yellow oil which did not require any additional purification (37.3 mg, 0.125 mmol, 97% yield, >95:5 *Z*:*E*).

^{1}H NMR (400 MHz, CDCl$_3$): δ 5.62 – 5.49 (m, 1H), 5.46 – 5.34 (m, 1H), 3.69 – 3.55 (m, 1H), 2.35 (t, *J* = 7.4 Hz, 2H), 2.27 – 2.16 (m, 2H), 2.11 – 1.98 (m, 2H), 1.70 – 1.57 (m, 2H), 1.51 – 1.21 (m, 18H), 0.92 – 0.84 (m, 3H) ppm. *(Two ^{1}H NMR signals for the carboxylic acid group and alcohol group were not observed)*

^{13}C NMR (176 MHz, CDCl$_3$): δ 178.5, 133.6, 125.3, 71.7, 36.9, 35.5, 34.0, 32.0, 29.6, 29.5, 29.1 (2C), 29.0, 27.5, 25.9, 24.8, 22.8, 14.3 ppm. *(^{13}C NMR resonance at 178.5 ppm was determined by HMBC analysis due to low intensity)*

IR (neat) ν_{max}: 3431, 2925, 2854, 2361, 1738, 1715, 1460, 1366, 1229, 1217, 1123, 966, 724, 527 cm^{-1}.

HRMS (ESI$^+$): exact mass calculated for $[M+Na]^+$ $(C_{18}H_{34}O_3{}^{23}Na)^+$ requires *m/z* 321.2400, found *m/z* 321.2403.

(±)-Lesquerolic acid (10b)

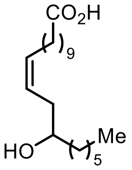

10b

General procedure **GP6** was followed using methyl ester **S10** (87:13 *Z*:*E*, 31.5 mg, 92.5 µmol, 1.00 equiv.) and potassium hydroxide (36 mg, 0.650 mmol, 7.00 equiv.). The resulting crude material was redissolved in Et_2O (5 mL) and potassium hydroxide (KOH, 2 M aq. 5 mL) was added to obtain pH > 12. The aqueous layer was separated, and the organic layer was washed with water (2 × 5 mL). The aqueous layers were combined, acidified with 1 M HCl_{aq} until pH < 2, and then extracted with EtOAc (3 × 10 mL). The organic layers were combined, washed with brine and dried over Na_2SO_4. After filtration, volatiles were removed *in vacuo* to obtain the product as a pale-yellow oil which did not require any additional purification (29.7 mg, 91.2 µmol, 98% yield) and a single isomer (87:13 *Z*/*E* based on the starting material **S10**).

^{1}H NMR (400 MHz, CDCl$_3$): δ 5.63 – 5.49 (m, 1H), 5.47 – 5.32 (m, 1H), 3.70 – 3.55 (m, 1H), 2.41 – 2.29 (m, 2H), 2.28 – 2.15 (m, 2H), 2.11 – 1.97 (m, 2H), 1.69 – 1.58 (m, 2H), 1.51 – 1.40 (m, 3H), 1.39 – 1.22 (m, 19H), 0.96 – 0.79 (m, 3H) ppm. *(Two ^{1}H NMR signals for the carboxylic acid group and alcohol group were not observed)*

^{13}C NMR (151 MHz, CDCl$_3$): δ 178.4, 133.7, 125.2, 71.8, 36.9, 35.5, 34.0, 32.0, 29.7, 29.5, 29.44, 29.39, 29.3, 29.2, 29.1, 27.5, 25.9, 24.8, 22.8, 14.2 ppm. *(^{13}C NMR resonance at 178.4 ppm was determined by HMBC analysis due to low intensity)*

IR (neat) ν_{max}: 3454, 2923, 2853, 1709, 1462, 1410, 1229, 1123, 1041, 911, 728, 649, 515, 479 cm^{-1}.

HRMS (ESI$^+$): exact mass calculated for [M+Na]$^+$ ($C_{20}H_{38}O_3{}^{23}Na$)$^+$ requires *m/z* 349.2713, found *m/z* 349.2714.

(±)-Densipolic acid (10c)

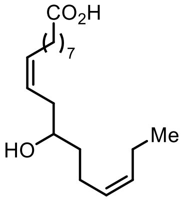

10c

General procedure **GP6** was followed using methyl ester **S11** (22.0 mg, 0.071 mmol, 1.00 equiv.) and potassium hydroxide (27.8 mg, 0.496 mmol, 7.00 equiv.). The resulting crude material was subjected to flash column chromatography (silica gel, 0%-50% EtOAc in heptane) to obtain the title compound as a colorless oil (19.9 mg, 0.067 mmol, 95% yield). The product was obtained as a mixture of *Z*/*E* isomers in a ratio of 89:11 (determined based on the analysis of ^{13}C NMR after purification).

^{1}H NMR (400 MHz, CDCl$_3$): δ 5.60 – 5.50 (m, 1H), 5.44 – 5.30 (m, 3H), 3.69 – 3.59 (m, 1H), 2.34 (t, *J* = 7.5 Hz, 2H), 2.26 – 1.99 (m, 8H), 1.67 – 1.48 (m, 4H), 1.39 – 1.24 (m, 9H), 0.96 (t, *J* = 7.5 Hz, 3H) ppm. *(One ^{1}H NMR signal for the carboxylic acid group was not observed)*

^{13}C NMR (101 MHz, CDCl$_3$): δ 179.5, 133.6, 132.4, 128.7, 125.2, 71.4, 36.8, 35.5, 34.1, 29.6, 29.15, 29.13, 29.05, 27.5, 24.8, 23.7, 20.7, 14.5 ppm.

IR (neat) ν_{max}: 3368, 2926, 2854, 1708, 1456, 1245, 1062, 910, 732 cm^{-1}.

HRMS (ESI$^+$): exact mass calculated for [M+Na]$^+$ ($C_{18}H_{32}O_3{}^{23}Na$)$^+$ requires *m/z* 319.2244, found *m/z* 319.2242.

(±)-Auricolic acid (10d)

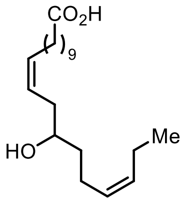

10d

General procedure **GP6** was followed using methyl ester **S12** (35.3 mg, 0.104 mmol, 1.00 equiv.) and potassium hydroxide (41 mg, 0.73 mmol, 7.0 equiv.). The resulting crude material was subjected to flash column chromatography (silica gel, 0%-50% EtOAc in heptane) to obtain the title compound as a colorless oil (33.5 mg, 0.103 mmol, 99% yield). The product was obtained as a mixture of *Z*/*E* isomers in a ratio of 89:11 (determined based on the analysis of ^{13}C NMR after purification).

^{1}H NMR (400 MHz, CDCl$_3$): δ 5.60 – 5.48 (m, 1H), 5.44 – 5.29 (m, 3H), 3.70 – 3.58 (m, 1H), 2.33 (t, J = 7.5 Hz, 2H), 2.26 – 1.99 (m, 8H), 1.67 – 1.48 (m, 4H), 1.39 – 1.20 (m, 13H), 0.96 (t, J = 7.5 Hz, 3H) ppm. *(One ^{1}H NMR signal for the carboxylic acid group was not observed)*

^{13}C NMR (101 MHz, CDCl$_3$): δ 179.6, 133.7, 132.4, 128.7, 125.1, 71.4, 36.8, 35.5, 34.1, 29.7, 29.5, 29.4, 29.34, 29.27, 29.1, 27.5, 24.8, 23.7, 20.7, 14.5 ppm.

IR (neat) ν_{max}: 3375, 2923, 2853, 1709, 1457, 1262, 1059, 933, 721 cm^{-1}.

HRMS (ESI^{+}): exact mass calculated for $[M+Na]^{+}$ $(C_{20}H_{36}O_{3}{}^{23}Na)^{+}$ requires *m/z* 347.2557, found *m/z* 347.2562.

4.2 Total Synthesis of the γ-butyrolactone natural product 8

In this section, the absolute configuration of (6*Z*)-3,4-*Trans*-9-oxo-3-methyl dodec-*cis*-6-en-4-olide drawn is the one which agrees with the Krische's method[62] (see page S65 for explanation).

Scheme S3: Synthetic route towards (6*Z*)-3,4-*trans*-9-oxo-3-methyl dodec-*cis*-6-en-4-olide.

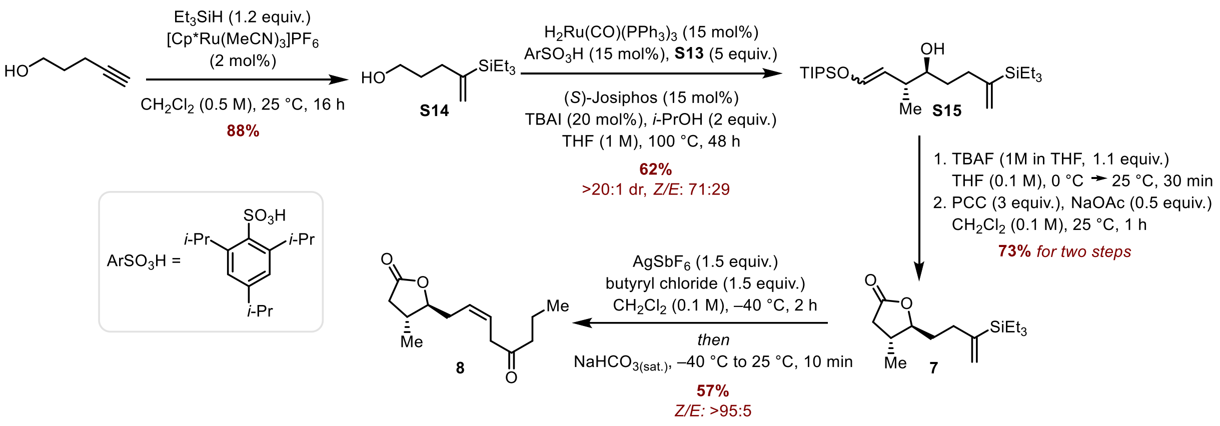

(But-2-yn-1-yloxy)triisopropylsilane (S13)

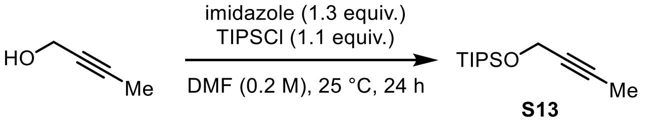

To a 250 mL round-bottom flask equipped with a magnetic stirring bar were added imidazole (1.77 g, 26.0 mmol, 1.3 equiv.) and a solution of 2-butyn-1-ol (1.40 g, 20.0 mmol, 1.0 equiv.) in DMF (100 mL). Subsequently, TIPSCl (4.7 mL, 22 mmol, 1.1 equiv.) was added dropwise at room temperature (25 °C) and the reaction was stirred for 24 h. After this time, the crude mixture was partitioned between Et_2O and water, and the organic layer was separated. The aqueous layer was extracted with Et_2O (2 × 100 mL), the organic layers were combined, washed with brine (2 × 100 mL) and dried over anhydrous Na_2SO_4. After filtration, volatiles were removed *in vacuo*, and the crude mixture was subjected to flash column chromatography (silica gel, 100% pentane) to obtain **S13** as a colorless oil (3.984 g, 17.6 mmol, 88% yield).

^{1}H NMR (400 MHz, $CDCl_3$): δ 4.33 (q, *J* = 2.3 Hz, 2H), 1.83 (t, *J* = 2.4 Hz, 3H), 1.17 – 1.03 (m, 21H) ppm.

^{13}C NMR (101 MHz, $CDCl_3$): δ 80.7, 78.0, 52.3, 18.1 (6C), 12.2 (3C), 3.7 ppm.

IR (neat) ν_{max}: 2943, 2866, 1463, 1370, 1146, 1084, 1065, 811, 679 cm^{-1}.

HRMS (ESI$^+$): exact mass calculated for $[M+Na]^+$ $(C_{13}H_{26}O^{28}Si^{23}Na)^+$ requires *m/z* 249.1645, found *m/z* 249.1640.

4-(Triethylsilyl)pent-4-en-1-ol (S14)

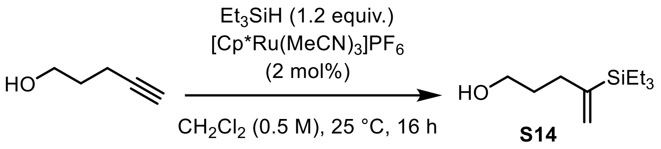

4-Pentyn-1-ol (252 mg, 3.00 mmol, 1.00 equiv.) was taken up in CH_2Cl_2 (6 mL) at 0 °C under argon atmosphere, followed by slow addition of triethylsilane (0.575 mL, 3.60 mmol, 1.20 equiv.). The solution was next treated with $[Cp^*Ru(MeCN)_3]PF_6$ (30.3 mg, 0.060 mmol, 2.00 mol%) and the reaction vessel was allowed to warm to room temperature (25 °C). After 16 h, full consumption of the alkyne was determined by TLC analysis, and the crude reaction mixture was concentrated *in vacuo.* The crude product was subjected to flash column chromatography (silica gel, 0%-30% EtOAc in heptane) to obtain **S14** as a colorless oil (528 mg, 2.63 mmol, 88% yield).

^{1}H NMR (400 MHz, CDCl$_3$): δ 5.66 (dt, *J* = 3.0, 1.6 Hz, 1H), 5.32 (dd, *J* = 2.8, 1.0 Hz, 1H), 3.66 (t, *J* = 6.3 Hz, 2H), 2.20 – 2.11 (m, 2H), 1.75 – 1.65 (m, 2H), 1.33 (s, 1H), 0.92 (t, *J* = 7.9 Hz, 9H), 0.61 (q, *J* = 7.9 Hz, 6H) ppm.

^{13}C NMR (101 MHz, CDCl$_3$): δ 148.6, 125.5, 63.0, 32.4, 32.0, 7.5 (3C), 3.0 (3C) ppm.

IR (neat) ν$_{max}$: 3322, 2951, 2875, 1457, 1416, 1237, 1008, 921, 716 cm^{-1}.

HRMS (ESI$^+$): exact mass calculated for $[M+H]^+$ $(C_{11}H_{25}O^{28}Si)^+$ requires *m/z* 201.1669, found *m/z* 201.1667.

(3*R*,4*S*)-3-Methyl-7-(triethylsilyl)-1-((triisopropylsilyl)oxy)octa-1,7-dien-4-ol (S15)

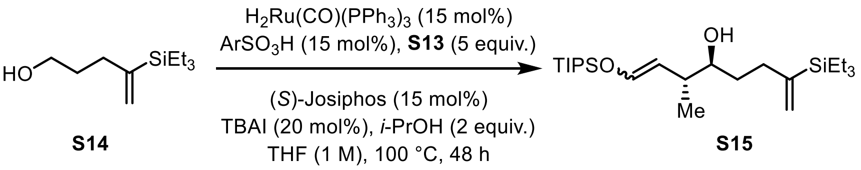

A modified procedure reported by M. Krische *et al.* was used.[62] To an oven-dried vial charged with a magnetic stirring bar, was added $H_2Ru(CO)(PPh_3)_3$ (138 mg, 0.150 mmol, 15.0 mol%), (*S*)-Josiphos (SL-J009-2, 83.2 mg, 0.150 mmol, 15.0 mol%), TBAI (73.9 mg, 0.200 mmol, 20.0 mol%) and 2,4,6-tri(2-propyl)phenylsulfonic acid (42.7 mg, 0.150 mmol, 15.0 mol%). Then, a THF solution (1.0 mL, 1 M concentration with respect to alcohol) of alkyne **S13** (1.13 g, 5.00 mmol, 5.00 equiv.) and alcohol **S14** (200 mg, 1.00 mmol, 1.00 equiv.) was added, followed by addition of 2-propylalcohol (153 µL, 2.00 mmol, 2.00 equiv.). The vial was tightly closed with a screw cap and placed in a metal heating block. The reaction mixture was heated at 100 °C for 48 h. After cooling to room temperature, the mixture was concentrated *in vacuo* and directly subjected to flash column chromatography (silica gel, 0%-5% EtOAc in heptane) to obtain **S15** as a pale-yellow oil (265 mg, 0.621 mmol, 62% yield). The product was obtained as a mixture of *Z*/*E* isomers in a ratio of 71:29 (determined based on the analysis of the crude ^{1}H NMR) and a single diastereoisomer (d.r. >20:1). ^{1}H NMR peaks corresponding exclusively to the minor isomer are indicated with an asterisk.

^{1}H NMR (400 MHz, C$_6$D$_6$): δ 6.43 (dd, *J* = 11.9, 0.5 Hz, 0.3H*), 6.31 (dd, *J* = 5.9, 0.8 Hz, 0.7H), 5.86 – 5.76 (m, 1H), 5.46 – 5.41 (m, 1H), 5.06 (dd, *J* = 11.9, 9.5 Hz, 0.3H*), 4.42 (dd, *J* = 9.4, 5.9 Hz, 0.7H), 3.46 (ddd,

J = 8.7, 5.3, 3.6 Hz, 0.7H), 3.26 (ddd, J = 8.9, 5.6, 3.4 Hz, 0.3H*), 3.04 – 2.92 (m, 0.7H), 2.60 – 2.44 (m, 1H), 2.39 – 2.22 (m, 1H), 2.07 – 1.95 (m, 0.3H*), 1.84 – 1.51 (m, 2H), 1.37 – 0.83 (m, 34H), 0.74 – 0.61 (m, 6H) ppm.

Major isomer: **^{13}C NMR (101 MHz, C_6D_6):** δ 147.5, 138.2, 123.6, 109.7, 73.5, 33.5, 32.7, 30.8, 16.0, 15.92 (3C), 15.91 (3C), 10.2 (3C), 5.7 (3C), 1.4 (3C) ppm.

Minor isomer: **^{13}C NMR (101 MHz, C_6D_6):** δ 147.4, 140.3, 123.6, 111.0, 73.0, 37.5, 32.2, 30.9, 16.1, 16.0 (3C), 15.8 (3C), 10.4 (3C), 5.7 (3C), 1.4 (3C) ppm.

IR (neat) ν_{max}: 2945, 2868, 1652, 1462, 1242, 1155, 1061, 1010, 719, 683 cm^{-1}.

HRMS (ESI$^-$): exact mass calculated for [M-H]$^-$ ($C_{24}H_{49}O_2{}^{28}Si_2$)$^-$ requires *m/z* 425.3277, found *m/z* 425.3272.

[α]$_D^{24}$ = –12.7 (c = 0.91 in $CDCl_3$).

(4*R*,5*S*)-4-Methyl-5-(3-(triethylsilyl)but-3-en-1-yl)dihydrofuran-2(3*H*)-one (7)

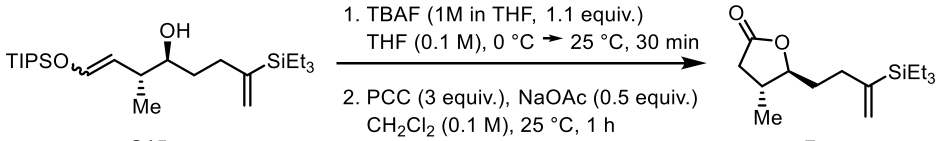

S15 **7**

The silyl enol ether **S15** (90.6 mg, 0.335 mmol, 1.00 equiv.) was dissolved in THF (3.4 mL) and TBAF (1.0 M in THF, 0.37 mL, 0.370 mmol, 1.10 equiv.) was added at 0 ºC. The mixture was stirred at room temperature (25 °C) for 30 min, followed by the addition of water (10 mL) and extraction with CH_2Cl_2 (3 × 5 mL). The organic layer was washed by brine (10 mL) and dried over anhydrous Na_2SO_4. the crude material was concentrated *in vacuo* and CH_2Cl_2 (3.4 mL), NaOAc (13.7 mg, 0.168 mmol, 0.50 equiv.) and pyridinium chlorochromate (PCC, 218 mg, 1.00 mmol, 3.00 equiv.) were added at room temperature (25 °C). The reaction mixture was stirred for 1 h, concentrated *in vacuo* and directly subjected to flash column chromatography (silica gel, 0%-20% EtOAc in heptane) to obtain **7** as a pale-yellow oil (65.2 mg, 0.243 mmol, 73% yield for two steps).

^{1}H NMR (400 MHz, $CDCl_3$): δ 5.66 (dt, J = 3.0, 1.6 Hz, 1H), 5.35 (d, J = 2.7 Hz, 1H), 4.02 (ddd, J = 8.7, 7.5, 3.6 Hz, 1H), 2.73 – 2.62 (m, 1H), 2.40 – 2.29 (m, 1H), 2.27 – 2.12 (m, 3H), 1.87 – 1.64 (m, 2H), 1.14 (d, J = 6.5 Hz, 3H), 0.92 (t, J = 7.9 Hz, 9H), 0.61 (q, J = 8.0 Hz, 6H) ppm.

^{13}C NMR (101 MHz, $CDCl_3$): δ 176.6, 147.8, 125.8, 87.0, 37.3, 36.3, 33.2, 32.0, 17.5, 7.5 (3C), 3.0 (3C) ppm.

IR (neat) ν_{max}: 2952, 2875, 1776, 1457, 1208, 1036, 1005, 931, 718 cm^{-1}.

HRMS (ESI$^+$): exact mass calculated for [M+Na]$^+$ ($C_{15}H_{28}O_2{}^{28}Si^{23}Na$)$^+$ requires *m/z* 291.1751, found *m/z* 291.1749.

[α]$_D^{24}$ = –50.7 (c = 0.98 in $CDCl_3$).

(4*R*,5*S*)-4-Methyl-5-((*Z*)-5-oxooct-2-en-1-yl)dihydrofuran-2(3*H*)-one (8)

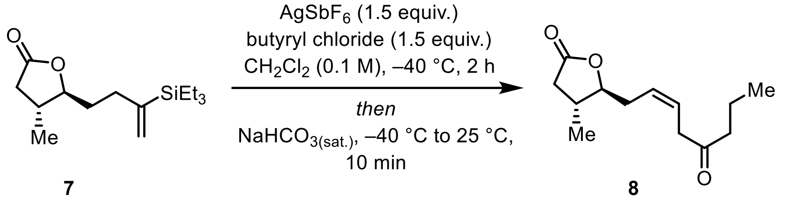

A flame-dried Schlenk tube was charged with a solution of vinyl silane **7** (40.3 mg, 0.150 mmol, 1.00 equiv.) and butyryl chloride (24.0 mg, 23.4 µL, 0.225 mmol, 1.50 equiv.) in anhydrous CH_2Cl_2 (1 mL) at room temperature. The reaction vessel was placed in a cooling bath at –40 °C (acetonitrile/dry ice cooling-bath) and allowed to stir for 1 min. In a separate vial, silver hexafluoroantimonate (77.3 mg, 0.225 mmol, 1.50 equiv.) was dissolved in CH_2Cl_2 (0.5 mL) at room temperature (25 °C), and the obtained solution was then added dropwise to the pre-cooled vinyl silane and acyl chloride mixture to obtain overall 0.1 M solution of the vinyl silane. The reaction mixture was stirred at –40 °C (with careful temperature control) for 2 h and then quenched with sat. aq. $NaHCO_3$ (equal volume to CH_2Cl_2) at the same temperature. The vial was immediately removed from the cooling bath and allowed to warm to room temperature over 10 min with vigorous stirring. The reaction mixture was partitioned between CH_2Cl_2 and water, and the organic layer was separated. The aqueous layer was extracted with CH_2Cl_2 (2 × 20 mL), the organic layers were combined, washed with brine and dried over anhydrous Na_2SO_4. After filtration, volatiles were removed *in vacuo*, and the crude mixture was purified by flash column chromatography (silica gel, 0%-40% EtOAc in heptane) to obtain **8** as a colorless oil (19.3 mg, 0.086 mmol, 57% yield). The product was obtained as a mixture of *Z*/*E* isomers in a ratio of >95:5 (determined based on the analysis of the crude ^1H NMR).

^1H NMR (400 MHz, $CDCl_3$): δ 5.82 – 5.71 (m, 1H), 5.69 – 5.59 (m, 1H), 4.07 (q, *J* = 6.9 Hz, 1H), 3.18 (d, *J* = 7.2 Hz, 2H), 2.68 (dd, *J* = 16.7, 7.6 Hz, 1H), 2.53 – 2.34 (m, 4H), 2.31 – 2.13 (m, 2H), 1.67 – 1.54 (m, 2H), 1.14 (d, *J* = 6.4 Hz, 3H), 0.91 (t, *J* = 7.4 Hz, 3H) ppm.

^{13}C NMR (101 MHz, $CDCl_3$): δ 208.4, 176.3, 126.9, 125.0, 86.4, 44.6, 41.7, 37.1, 35.4, 31.7, 17.7, 17.3, 13.8 ppm.

IR (neat) ν_{max}: 2962, 2876, 1773, 1711, 1421, 1207, 1152, 1005, 930 cm^{-1}.

HRMS (ESI$^+$): exact mass calculated for $[M+Na]^+$ $(C_{13}H_{20}O_3{}^{23}Na)^+$ requires *m/z* 247.1305, found *m/z* 247.1308.

$[\alpha]_D^{24}$ = –28.7 (c = 0.75 in MeOH).

Table S7: ^{1}H NMR ($CDCl_3$) Spectroscopic comparison of natural and synthetic γ-butyrolactone with the sample synthesized in this work

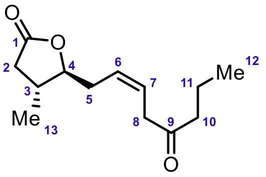

No.	Natural[60], 400 MHz<br>δ ^{1}H [ppm, mult, J(Hz)]	Synthetic[61], 300 MHz<br>δ ^{1}H [ppm, mult, J(Hz)]	This work, 400 MHz<br>δ ^{1}H [ppm, mult, J(Hz)]
2a	2.68, (dd, *J* = 17.0, 7.8 Hz, 1H)	2.68, (dd, *J* = 16.9, 7.7 Hz, 1H)	2.68 (dd, *J* = 16.7, 7.6 Hz, 1H)
2b	2.20, (overlap, 1H)	2.18, (m, 1H)	2.31 – 2.13 (m, 1H)
3	2.27, (m, 1H)	2.26, (m, 1H)	2.31 – 2.13 (m, 1H)
4	4.07, (m, 1H)	4.08, (m, 1H)	4.07 (q, *J* = 6.9 Hz, 1H)
5a	2.48, (m, 1H)	2.50 – 2.37, (m, 1H)	2.53 – 2.34 (m, 1H)
5b	2.41, (m, 1H)	2.50 – 2.37, (m, 1H)	2.53 – 2.34 (m, 1H)
6	5.65, (m, 1H)	5.65, (m, 1H)	5.69 – 5.59 (m, 1H)
7	5.77, (m, 1H)	5.77, (m, 1H)	5.82 – 5.71 (m, 1H)
8	3.19, (m, 2H)	3.19, (d, *J* = 7.1 Hz, 2H)	3.18 (d, *J* = 7.2 Hz, 2H)
10	2.43, (m, 2H)	2.50-2.37, (m, 2H)	2.53 – 2.34 (m, 2H)
11	1.60, (m, 2H)	1.70 – 1.52, (m, 2H)	1.67 – 1.54 (m, 2H),
12	0.92, (t, *J* = 7.2 Hz, 3H)	0.92, (t, *J* = 7.3, 3H)	0.91 (t, *J* = 7.4 Hz, 3H)
13	1.14, (d, *J* = 6.6 Hz, 3H)	1.15, (d, *J* = 6.4, 3H)	1.14 (d, *J* = 6.4 Hz, 3H),

Table S8: ^{13}C NMR ($CDCl_3$) Spectroscopic comparison of natural and synthetic γ-butyrolactone with the sample synthesized in this work

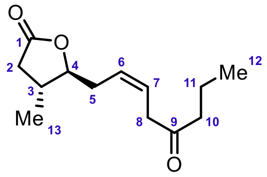

No.	**Natural[60], 101 MHz** δ ^{13}C (ppm)	**Synthetic[61], 125 MHz** δ ^{13}C (ppm)	**This work, 101 MHz** δ ^{13}C (ppm)
1	175.7	176.1	176.3
2	36.9	36.9	37.1
3	35.2	35.2	35.4
4	86.2	86.1	86.4
5	31.5	31.5	31.7
6	126.6	126.6	126.9
7	124.9	124.8	125.0
8	41.5	41.5	41.7
9	208.3	208.2	208.4
10	44.5	44.4	44.6
11	17.2	17.1	17.3
12	13.7	13.6	13.8
13	17.5	17.4	17.7

Stereoisomer analysis:

Based on the sign of [α]D values, the assignment of the absolute configuration of (4*R*,5*S*)-4-methyl-5-((*Z*)-5-oxooct-2-en-1-yl)dihydrofuran-2(3*H*)-one **8** in the isolation[60] and synthetic[61] papers is opposite to the absolute configuration expected following M. Krische's[62] stereoselective coupling of propargyl ethers with alcohols. We were unable to explain this discrepancy; hence we synthesized both enantiomers of this natural product using (*S*)-Josiphos (SL-J009-2) and (*R*)-Josiphos (SL-J009-1). Our methodology allows us to efficiently obtain both enantiomers of this natural product with similar overall yield and high stereoselectivity.

For convenience, the absolute configuration drawn is the one which agrees with Krische's method. [62]

Scheme S4: Synthesis of the opposite enantiomer of the γ-butyrolactone natural product.

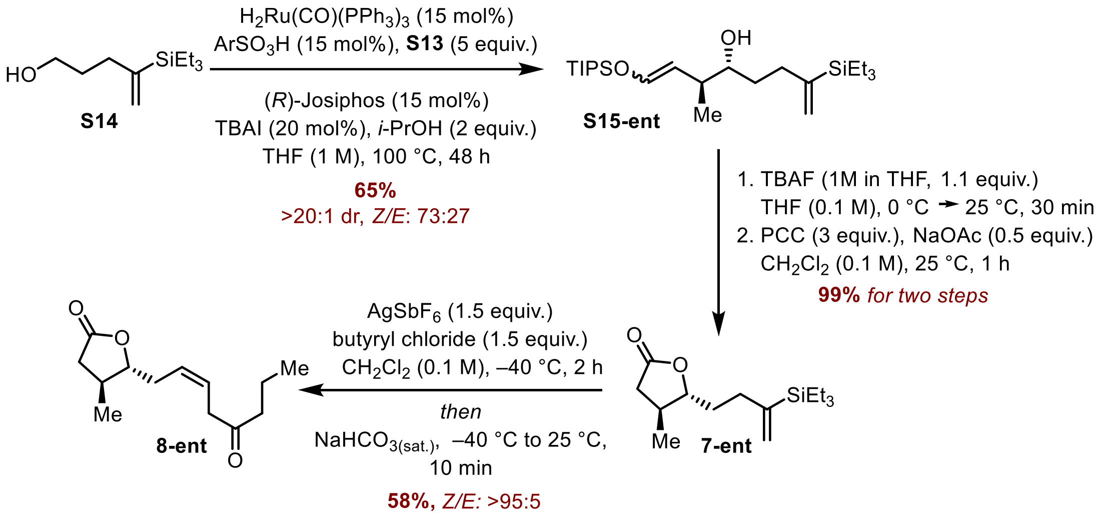

The analytical data for **S15-ent**, **7-ent** and **8-ent** is consistent with **S15**, **7** and **8** respectively (see pages S60-S62).

Specific rotation for **S15-ent**: $[\alpha]_D^{24}$ = +11.1 (c = 0.74 in $CHCl_3$).

Specific rotation for **7-ent**: $[\alpha]_D^{24}$ = +47.4 (c = 1.00 in $CHCl_3$).

Specific rotation for **8-ent**: $[\alpha]_D^{24}$ = +31.9 (c = 0.84 in MeOH).

Specific rotation for isolated[60] γ-butyrolactone: $[\alpha]_D^{25}$ = +68.4 (c = 0.07 in MeOH)

Specific rotation for synthetic[61] γ-butyrolactone: $[\alpha]_D^{25}$ = +60.8 (c = 0.20 in MeOH);

The ratio of enantiomers for **8** was determined based on **9** since both were synthesised from the same common vinyl silane **7**; The ratio of enantiomers for **8-ent** was determined based on **9-ent** since both were synthesised from the same common vinyl silane **7-ent**. The er measurement of **8**/**8-ent** was not possible due to the lack of UV activity and potential issues with diastereomer formation.

Despite the observed lower [α]D value for our samples of natural product, enantiopurity of the samples is not impaired (*see below section*).

Determination of enantiopurity:

Note: The absolute configuration drawn in this section is the one which agrees with Krische's method[62] (see page S65 for explanation).

Synthesis of the racemic sample:

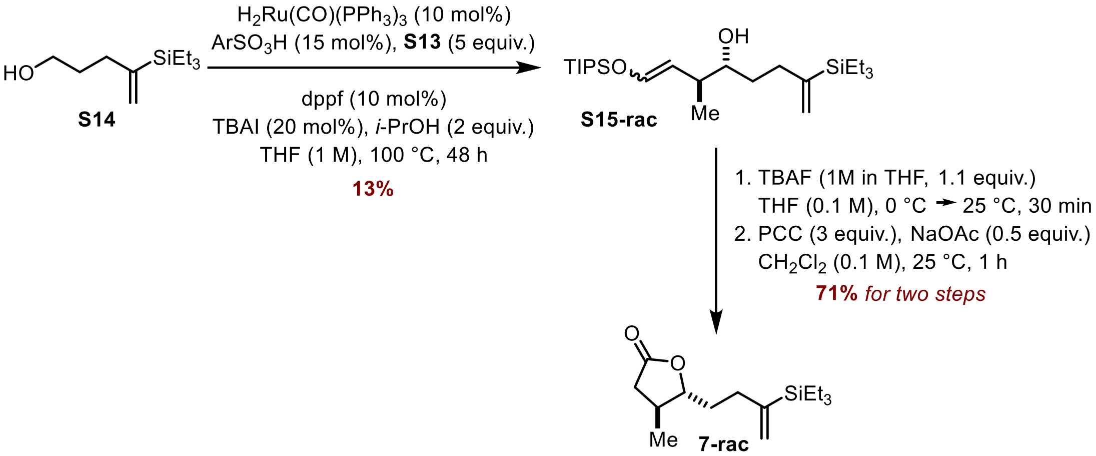

The analytical data for **S15-rac** and **7-rac** is consistent with **S15** and **7** and respectively (see pages S60-S61).

(±)- (4*S*,5*R*)-4-Methyl-5-((*Z*)-5-oxo-5-phenylpent-2-en-1-yl)dihydrofuran-2(3*H*)-one (9-rac)

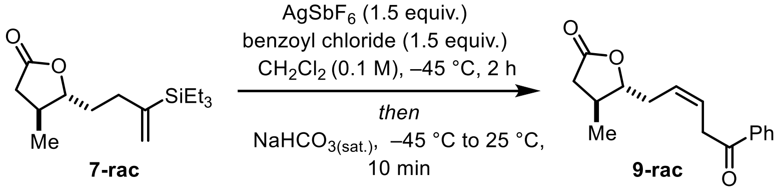

General procedure **GP3** was followed using vinyl silane **7-rac** (27.8 mg, 0.104 µmol, 1.00 equiv.), benzoyl chloride (18 µL, 0.16 mmol, 1.50 equiv.) and silver hexafluoroantimonate (53.4 mg, 0.155 mmol, 1.50 equiv.) at –45 °C (acetonitrile/dry ice; temperature was carefully controlled). The resulting crude material was subjected to flash column chromatography (silica gel, 0%-45% EtOAc in heptane) to obtain the title compound as orange oil (13.4 mg, 0.052 mmol, 50% yield). The product was obtained as a mixture of *Z*/*E* isomers in a ratio of >95:5 (determined based on the analysis of ^{1}H NMR after purification).

The analytical data for **9-rac** is consistent with enantioenriched sample **9** (see pages S67).

(4*R*,5*S*)-4-Methyl-5-((*Z*)-5-oxo-5-phenylpent-2-en-1-yl)dihydrofuran-2(3*H*)-one (9)

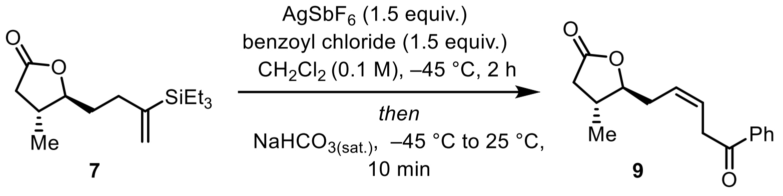

General procedure **GP3** was followed using vinyl silane **7** (13.0 mg, 48.4 μmol, 1.00 equiv.), benzoyl chloride (8.4 μL, 73 μmol, 1.50 equiv.) and silver hexafluoroantimonate (25 mg, 73 μmol, 1.50 equiv.) at –45 °C (acetonitrile/dry ice; temperature was carefully controlled). The resulting crude material was subjected to flash column chromatography (silica gel, 0%-45% EtOAc in heptane) to obtain the title compound as orange oil (5.9 mg, 0.023 mmol, 47% yield). The product was obtained as a mixture of *Z*/*E* isomers in a ratio of >95:5 (determined based on the analysis of ^{1}H NMR after purification).

^{1}H NMR (400 MHz, C_6D_6): δ 7.89 – 7.78 (m, 2H), 7.14 – 7.02 (m, 3H), 5.89 – 5.77 (m, 1H), 5.49 – 5.38 (m, 1H), 3.50 – 3.30 (m, 3H), 2.14 – 1.96 (m, 3H), 1.58 – 1.45 (m, 2H), 0.45 (d, *J* = 6.1 Hz, 3H) ppm.

^{13}C NMR (151 MHz, C_6D_6): δ 196.4, 174.7, 137.1, 133.0, 128.8 (2C), 128.6 (2C), 127.2, 125.5, 85.3, 37.6, 36.8, 35.1, 31.9, 17.0 ppm.

IR (neat) ν_{max}: 2924, 1775, 1684, 1597, 1581, 1449, 1422, 1330, 1209, 1153, 1015, 932, 757, 692 cm^{-1}.

HRMS (ESI$^+$): exact mass calculated for $[M+Na]^+$ $(C_{16}H_{18}O_3{}^{23}Na)^+$ requires *m*/*z* 281.1148, found *m*/*z* 281.1147.

Enantiomeric excess: 88% determined by chiral HPLC analysis: Chiralpak IH-3, *n*-heptane + 0,1%IPA/IPA 85:15, 1.0 mL/min, 25 °C, detection at 210 nm, retention time (min): 17.1 (major) and 20.8 (minor).

$[\alpha]_D^{23}$ = –27.4 (*c* = 0.23, $CHCl_3$).

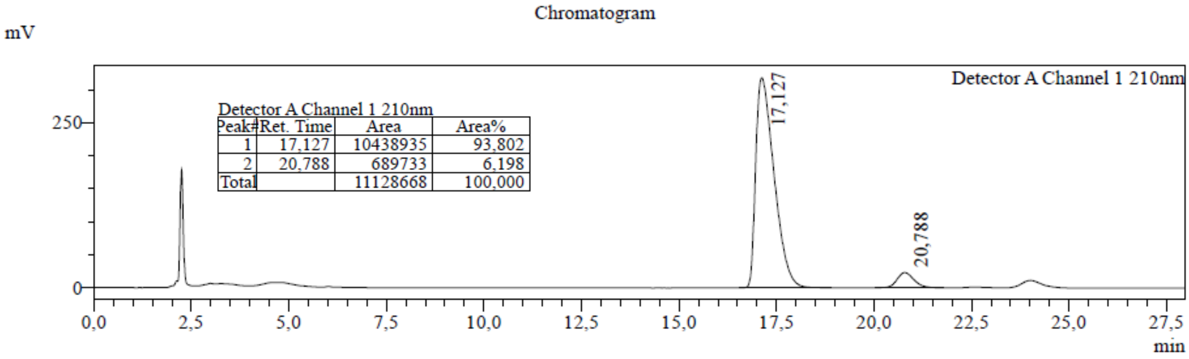

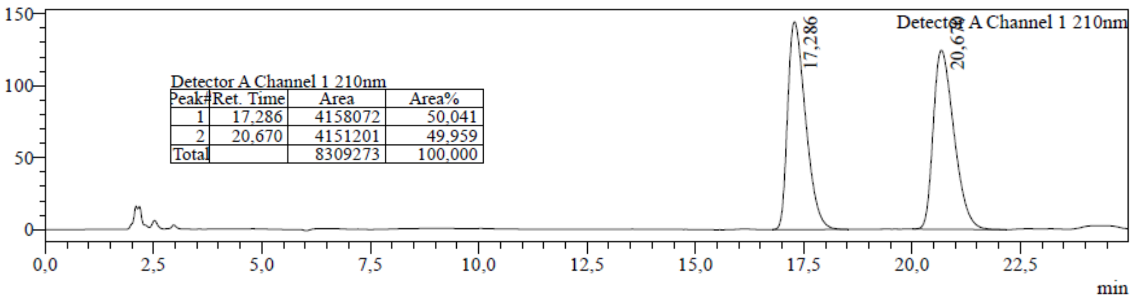

(4*S*,5*R*)-4-Methyl-5-((*Z*)-5-oxo-5-phenylpent-2-en-1-yl)dihydrofuran-2(3*H*)-one (9-ent)

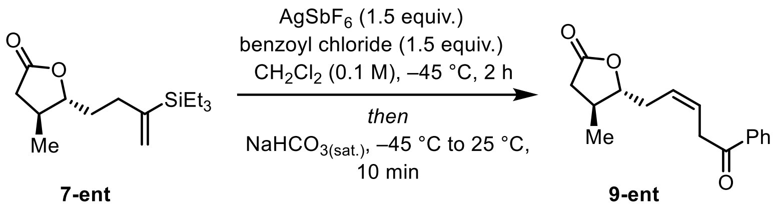

7-ent **9-ent**

General procedure **GP3** was followed using vinyl silane **7-ent** (12.5 mg, 46.6 µmol, 1.00 equiv.), benzoyl chloride (8.1 µL, 70 µmol, 1.5 equiv.) and silver hexafluoroantimonate (24.2 mg, 70.0 µmol, 1.50 equiv.) at –45 °C (acetonitrile/dry ice; temperature was carefully controlled). The resulting crude material was subjected to flash column chromatography (silica gel, 0%-45% EtOAc in heptane) to obtain the title compound as a pale-yellow oil (6.4 mg, 0.025 mmol, 53% yield). The product was obtained as a mixture of *Z*/*E* isomers in a ratio of >95:5 (determined based on the analysis of ^{1}H NMR after purification).

All analytical data is consistent with **9**.

Enantiomeric excess: 88% determined by chiral HPLC analysis: Chiralpak IH-3, *n*-heptane + 0,1%IPA/IPA 85:15, 1.0 mL/min, 25 °C, detection at 210 nm, retention time (min): 17.2 (minor) and 20.4 (major).

$[\alpha]_D^{24}$ = +23.3 (*c* = 0.30, $CHCl_3$).

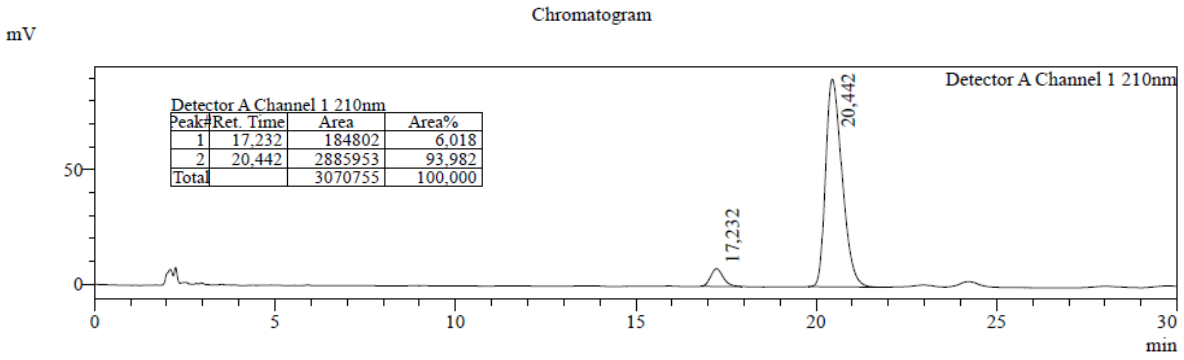

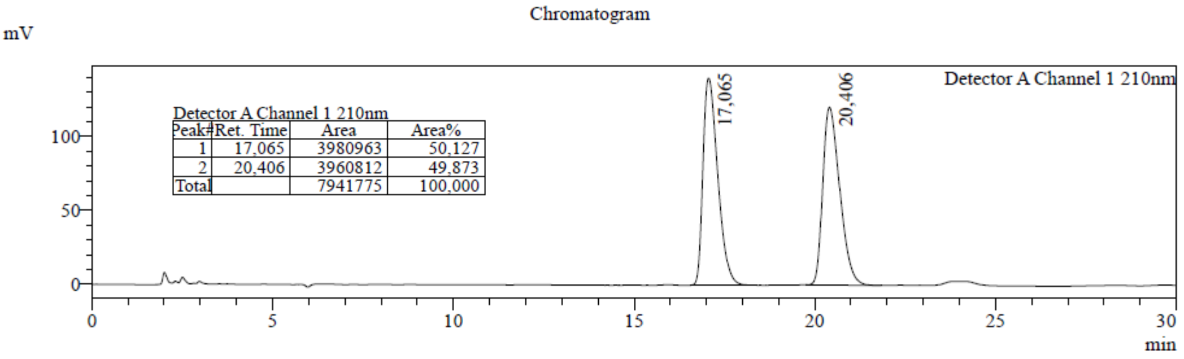

5. Mechanistic studies

5.1 Synthesis of THF derivative 12 for confirmation of formation of an oxocarbenium intermediate

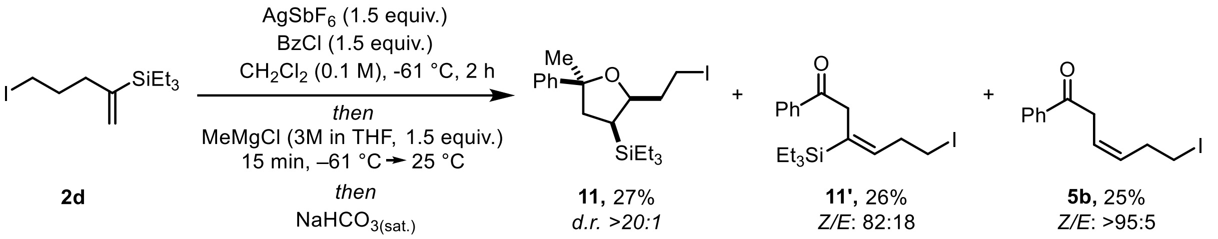

A flame-dried Schlenk tube was charged with a solution of **2d** (310 mg, 1.00 mmol, 1.00 equiv.) and benzoyl chloride (211 mg, 0.174 µL, 1.50 mmol, 1.50 equiv.) in anhydrous CH_2Cl_2 (7 mL) at room temperature. The solution was placed in a cooling bath at –61 °C (chloroform/dry ice cooling-bath) and allowed to stir for 1 min. In a separate vial, silver hexafluoroantimonate (515 mg, 1.50 mmol, 1.50 equiv.) was dissolved in CH_2Cl_2 (3 mL) at room temperature, and the obtained solution was then added dropwise to the pre-cooled vinyl silane and acyl chloride mixture to obtain overall 0.1 M solution of the vinyl silane. The reaction mixture was stirred at –61 °C for 2 h, after which a solution of methylmagnesium chloride (3 M in THF, 0.5 mL, 1.50 mmol, 1.50 equiv.) was added. The resulting solution was slowly warmed to room temperature (25 °C) over 15 min, followed by addition of sat. aq. $NaHCO_3$ (equal volume to CH_2Cl_2) at the same temperature. The reaction mixture was partitioned between CH_2Cl_2 and water, and the organic layer was separated. The aqueous layer was extracted with CH_2Cl_2 (2 × 50 mL), the organic layers were combined, washed with brine and dried over anhydrous Na_2SO_4. After filtration, volatiles were removed *in vacuo*, and the crude mixture was subjected to flash column chromatography (silica gel, 0%-20% Et_2O in heptane) to obtain **11** as a colorless oil (115.1 mg, 0.272 mmol, 27% yield) and a single diastereoisomer (d.r. >20:1), **11'** as a colorless oil (101.4 mg, 0.241 mmol, 24% yield) and a mixture of *Z*/*E* isomers in a ratio of 82:18 and **5b** as a colorless oil (76.2 mg, 0.254 mmol, 25% yield) and a single *Z* isomer (*Z*/*E* >95:5). All ratios were determined based on the analysis of ^{1}H NMR after purification.

(±)-Triethyl((2*S*,3*S*,5*S*)-2-(2-iodoethyl)-5-methyl-5-phenyltetrahydrofuran-3-yl)silane (11)

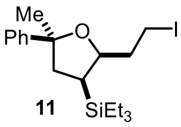

11

^{1}H NMR (400 MHz, CDCl$_3$): δ 7.39 – 7.36 (m, 2H), 7.34 – 7.29 (m, 2H), 7.21 (tt, *J* = 6.5, 1.3 Hz, 1H), 4.46 (dq, *J* = 9.7, 3.4 Hz, 1H), 3.41 – 3.32 (m, 2H), 2.28 – 2.23 (m, 1H), 2.04 – 1.98 (m, 2H), 1.77 – 1.68 (m, 2H), 1.43 (s, 3H), 0.99 (t, *J* = 7.9 Hz, 9H), 0.61 (q, *J* = 7.6 Hz, 6H) ppm.

^{13}C NMR (101 MHz, CDCl$_3$): δ 150.3, 128.3 (2C), 126.4, 124.6 (2C), 84.6, 82.0, 41.2, 39.1, 31.2, 29.0, 7.9 (3C), 4.8, 3.9 (3C) ppm.

IR (neat) ν_{max}: 2952, 2874, 1445, 1367, 1159, 1064, 1010, 818, 699 cm^{-1}.

HRMS (ESI$^+$): exact mass calculated for $[M+Na]^+$ $(C_{19}H_{31}{}^{127}IO^{28}Si^{23}Na)^+$ requires *m/z* 453.1081, found *m/z* 453.1078.

(*E*)-6-Iodo-1-phenyl-3-(triethylsilyl)hex-3-en-1-one (11')

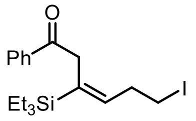

11'

Major isomer: **^{1}H NMR (400 MHz, $CDCl_3$):** δ 8.01 – 7.91 (m, 2H), 7.60 – 7.53 (m, 1H), 7.48 (t, *J* = 8.4 Hz, 2H), 5.95 (t, *J* = 6.8 Hz, 1H), 3.83 (s, 2H), 3.20 (t, *J* = 7.1 Hz, 2H), 2.65 (q, *J* = 7.0 Hz, 2H), 0.92 (t, *J* = 7.9 Hz, 9H), 0.58 (q, *J* = 7.9 Hz, 6H) ppm.

^{13}C NMR (101 MHz, $CDCl_3$): δ 197.3, 143.1, 137.3, 133.6, 133.2, 128.8 (2C), 128.2 (2C), 39.6, 33.4, 7.5 (3C), 5.1, 3.0 (3C) ppm.

Minor isomer: **^{1}H NMR (400 MHz, $CDCl_3$):** δ 7.99 – 7.92 (m, 2H), 7.60 – 7.52 (m, 1H), 7.52 – 7.41 (m, 2H), 5.95 (t, *J* = 6.8 Hz, 1H), 3.74 (s, 2H), 3.11 (t, *J* = 7.3 Hz, 2H), 2.76 (q, *J* = 7.3 Hz, 2H), 0.97 (t, *J* = 7.9 Hz, 9H), 0.69 (q, *J* = 8.4, 7.9 Hz, 6H) ppm.

^{13}C NMR (101 MHz, $CDCl_3$): δ 199.6, 145.5, 137.1, 134.5, 133.1, 128.7 (2C), 128.4 (2C), 48.2, 36.2, 7.7 (3C), 4.5, 4.3 (3C) ppm.

IR (neat) ν_{max}: 2951, 2873, 1687, 1448, 1240, 1211, 1170, 1001, 716 cm^{-1}.

HRMS (ESI$^+$): exact mass calculated for $[M+H]^+$ $(C_{18}H_{28}{}^{127}IO^{28}Si^{23})^+$ requires *m/z* 415.0949, found *m/z* 415.0941.

(±)-2-((2*S*,3*S*,5*S*)-5-Methyl-5-phenyl-3-(triethylsilyl)tetrahydrofuran-2-yl)-N-(pyridin-2-ylmethyl)ethan-1-amine (12·2HCl)

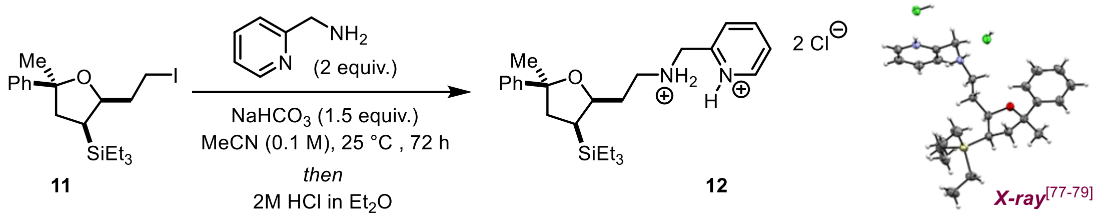

To a 10 mL round-bottom flask equipped with a magnetic stirring bar were added $NaHCO_3$ (25.2 mg, 0.300 mmol, 1.50 equiv.), 2-(aminomethyl)pyridine (41.2 µL, 0.400 mmol, 2.00 equiv.) and **11** (86.1 mg, 0.200 mmol, 1.00 equiv.) in acetonitrile (2 mL). The resulting mixture was stirred at room temperature (25 °C) for 72 h. After this time, the reaction mixture was diluted with distilled H_2O and the aqueous phase was extracted with CH_2Cl_2 (3 × 10 mL). The combined organic phases were dried over anhydrous Na_2SO_4, concentrated *in vacuo*, and the crude was purified by flash column chromatography (silica gel, 0%-10% MeOH in CH_2Cl_2) to afford the title compound as a pale-yellow oil (57.4 mg, 0.14 mmol, 69% yield). Subsequently, the free amine was dissolved in 2 mL of Et_2O and treated with 2M HCl in Et_2O (1 mL). The solution was concentrated *in vacuo* affording pale-yellow solid which was subjected to recrystallization from acetonitrile.

^{1}H NMR (400 MHz, CDCl$_3$): δ 10.26 (br s, 2H), 8.57 (d, *J* = 4.5 Hz, 1H), 8.07 (s, 2H), 7.52 (s, 1H), 7.35 (d, *J* = 7.4 Hz, 2H), 7.27 (t, *J* = 7.5 Hz, 2H), 7.17 (t, *J* = 7.2 Hz, 1H), 4.61 – 4.31 (m, 3H), 3.42 – 3.22 (m, 2H), 2.20 – 1.80 (m, 5H), 1.43 (s, 3H), 0.97 (t, *J* = 7.9 Hz, 9H), 0.63 (q, *J* = 7.9 Hz, 6H) ppm.

^{13}C NMR (101 MHz, CDCl$_3$): δ 148.6, 148.4, 145.4, 142.0, 128.6 (2C), 127.1, 126.9, 125.5, 124.9 (2C), 85.1, 80.3, 49.1, 47.5, 40.6, 31.6, 29.4, 28.7, 8.0 (3C), 3.9 (3C) ppm.

IR (neat) ν_{max}: 2954, 2874, 2609, 2363, 1620, 1465, 1093, 998, 700 cm^{-1}.

HRMS (ESI$^+$): exact mass calculated for $[M]^+$ $(C_{25}H_{39}N_2O^{28}Si)^+$ requires *m/z* 411.2826, found *m/z* 411.2814.

5.2 Synthesis of additional THF derivatives

(±)-(2-Benzyl-5-methyl-5-phenyltetrahydrofuran-3-yl)triethylsilane (S16)

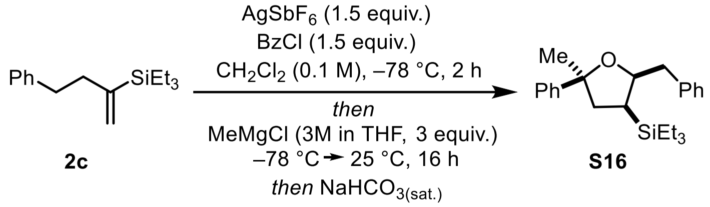

In a flame-dried vial, triethyl(4-phenylbut-1-en-2-yl)silane **2c** (37.0 mg, 0.150 mmol, 1.00 equiv.) was dissolved in CH_2Cl_2 (0.75 mL) and benzoyl chloride (26 μL, 0.230 mmol, 1.50 equiv.) was added in one portion at room temperature. The obtained solution was placed in a cooling bath at –78 °C and allowed to stir for 1 min. In a separate vial, silver hexafluoroantimonate (77.3 mg, 0.225 mmol, 1.50 equiv.) was dissolved in CH_2Cl_2 (0.75 mL) at room temperature (25 °C), and the obtained solution was added dropwise to the pre-cooled vinyl silane solution to obtain overall 0.1 M solution of the vinyl silane. The reaction mixture was stirred at –78 °C for 2 h, after which a solution of methylmagnesium chloride (3 M in THF, 0.15 mL, 0.450 mmol, 3.00 equiv.) was added dropwise at the same temperature. The obtained reaction mixture was allowed to slowly warm to room temperature over 16 h under vigorous stirring. Sat. aq. $NaHCO_3$ (1.5 mL) was then added and the obtained suspension was stirred for 10 min. The reaction mixture was partitioned between CH_2Cl_2 (5 mL) and water (5 mL), and the organic layer was separated. The aqueous layer was extracted with CH_2Cl_2 (2 × 5 mL), the organic layers were combined, washed with brine (15 mL) and dried over anhydrous Na_2SO_4. After filtration, volatiles were removed *in vacuo*, and the crude mixture was purified by flash column chromatography (silica gel, 0%-15% EtOAc in heptane) to obtain the desired product as a colorless oil (21.2 mg, 0.057 mmol, 39% yield, d.r. >20:1).

^{1}H NMR (400 MHz, CDCl$_3$): δ 7.43 – 7.13 (m, 10H), 4.74 (ddd, *J* = 11.3, 7.4, 2.0 Hz, 1H), 2.70 – 2.62 (m, 1H), 2.48 (dd, *J* = 13.7, 11.4 Hz, 1H), 2.28 (dd, *J* = 11.8, 6.6 Hz, 1H), 2.24 – 2.14 (m, 1H), 2.11 – 2.02 (m, 1H), 1.39 (s, 3H), 1.04 (t, *J* = 7.9 Hz, 9H), 0.69 (q, *J* = 7.7 Hz, 6H) ppm.

^{13}C NMR (101 MHz, CDCl$_3$): δ 150.3, 140.2, 129.4 (2C), 128.3 (2C), 128.0 (2C), 126.3, 126.1, 125.2 (2C), 84.4, 83.6, 41.9, 41.0, 31.1, 29.5, 8.0 (3C), 4.0 (3C) ppm.

IR (neat) ν_{max}: 3026, 2952, 2874, 1689, 1602, 1495, 1454, 1366, 1317, 1177, 1080, 1016, 732, 698, 562 cm^{-1}.

HRMS (ESI$^+$): exact mass calculated for $[M+Na]^+$ $(C_{24}H_{34}O^{28}SiNa)^+$ requires *m*/*z* 389.2271, found *m*/*z* 389.2269.

Relative stereochemistry was assigned based on compound **12**.

(±)-(2-Benzyl-5-(4-methoxyphenyl)-5-methyltetrahydrofuran-3-yl)triethylsilane (S17)

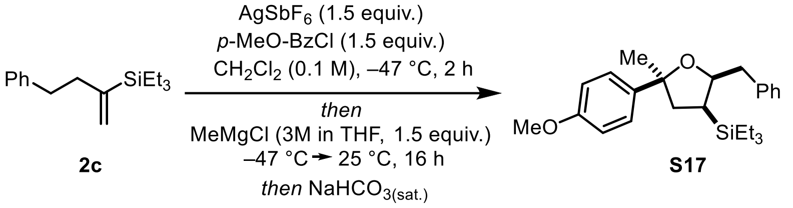

In a flame-dried vial, triethyl(4-phenylbut-1-en-2-yl)silane **2c** (49.3 mg, 0.200 mmol, 1.00 equiv.) was dissolved in CH_2Cl_2 (1 mL) and 4-methoxybenzoyl chloride (41 µL, 0.300 mmol, 1.50 equiv.) was added in one portion at room temperature (25 °C). The obtained solution was placed in a cooling bath at –47 °C and allowed to stir for 1 min. In a separate vial, silver hexafluoroantimonate (103 mg, 0.300 mmol, 1.50 equiv.) was dissolved in CH_2Cl_2 (1 mL) at room temperature, and the obtained solution was added dropwise to the pre-cooled vinyl silane solution to obtain overall 0.1 M solution of the vinyl silane. The reaction mixture was stirred at –47 °C for 2 h, after which a solution of methylmagnesium chloride (MeMgCl, 3 M in THF, 0.10 mL, 0.300 mmol, 1.50 equiv.) was added dropwise at the same temperature. The obtained reaction mixture was allowed to slowly warm to room temperature (25 °C) over 16 h under vigorous stirring. Sat. aq. $NaHCO_3$ (2 mL) was then added and the obtained suspension was stirred for 10 min. The reaction mixture was partitioned between CH_2Cl_2 (5 mL) and water (5 mL), and the organic layer was separated. The aqueous layer was extracted with CH_2Cl_2 (2 × 5 mL), the organic layers were combined, washed with brine (15 mL) and dried over anhydrous Na_2SO_4. After filtration, volatiles were removed *in vacuo*, and the crude mixture was purified by flash column chromatography (silica gel, 0%-15% EtOAc in heptane) to obtain the desired product as a colorless oil (24.7 mg, 0.062 mmol, 31% yield, d.r. >20:1).

^{1}H NMR (400 MHz, $CDCl_3$): δ 7.35 – 7.16 (m, 7H), 6.91 – 6.81 (m, 2H), 4.74 (ddd, *J* = 11.4, 7.4, 2.0 Hz, 1H), 3.83 (s, 3H), 2.68 (dd, *J* = 13.6, 1.4 Hz, 1H), 2.51 (dd, *J* = 13.7, 11.4 Hz, 1H), 2.31 – 2.14 (m, 2H), 2.12 – 2.02 (m, 1H), 1.39 (s, 3H), 1.05 (t, *J* = 7.9 Hz, 9H), 0.71 (q, *J* = 7.7 Hz, 6H) ppm.

^{13}C NMR (101 MHz, $CDCl_3$): δ 158.1, 142.7, 140.2, 129.4 (2C), 128.3 (2C), 126.3 (2C), 126.1, 113.3 (2C), 84.2, 83.5, 55.4, 41.9, 41.1, 31.2, 29.6, 8.0 (3C), 4.0 (3C) ppm.

IR (neat) ν_{max}: 2952, 2874, 1611, 1583, 1511, 1454, 1415, 1366, 1243, 1174, 946, 914, 830, 787, 560 cm^{-1}.

HRMS (ESI$^+$): exact mass calculated for $[M+Na]^+$ $(C_{25}H_{36}O_2{}^{28}Si^{23}Na)^+$ requires *m*/*z* 419.2377, found *m*/*z* 419.2371.

Relative stereochemistry was assigned based on compound **12**.

5.3 Comment on the desilylation step

Upon analyzing the crude NMR data (see pages S187–S189) for the synthesis of (*Z*)-1,5-diphenylpent-3-en-1-one (**4a**) from triethyl(4-phenylbut-1-en-2-yl)silane (**2c**), we believe the identity of the major silicon by-product species to be hexaethyldisiloxane ($(Et_3Si)_2O$, or $(Hex_3Si)_2O$ for trihexyl vinyl silanes),[80-81] suggesting that water or $NaHCO_3$ act as the required nucleophile in the desilylation step. However, the formation of triethylsilanol/trihexylsilanol (R_3SiOH), fluorotriethylsilane/fluorotrihexylsilane (R_3SiF) or other related silicon-containing species cannot be ruled out.

6. Flow chart to help determining the optimum reaction temperature

Below, we provide a flow chart to assist in determining the optimal reaction temperature.

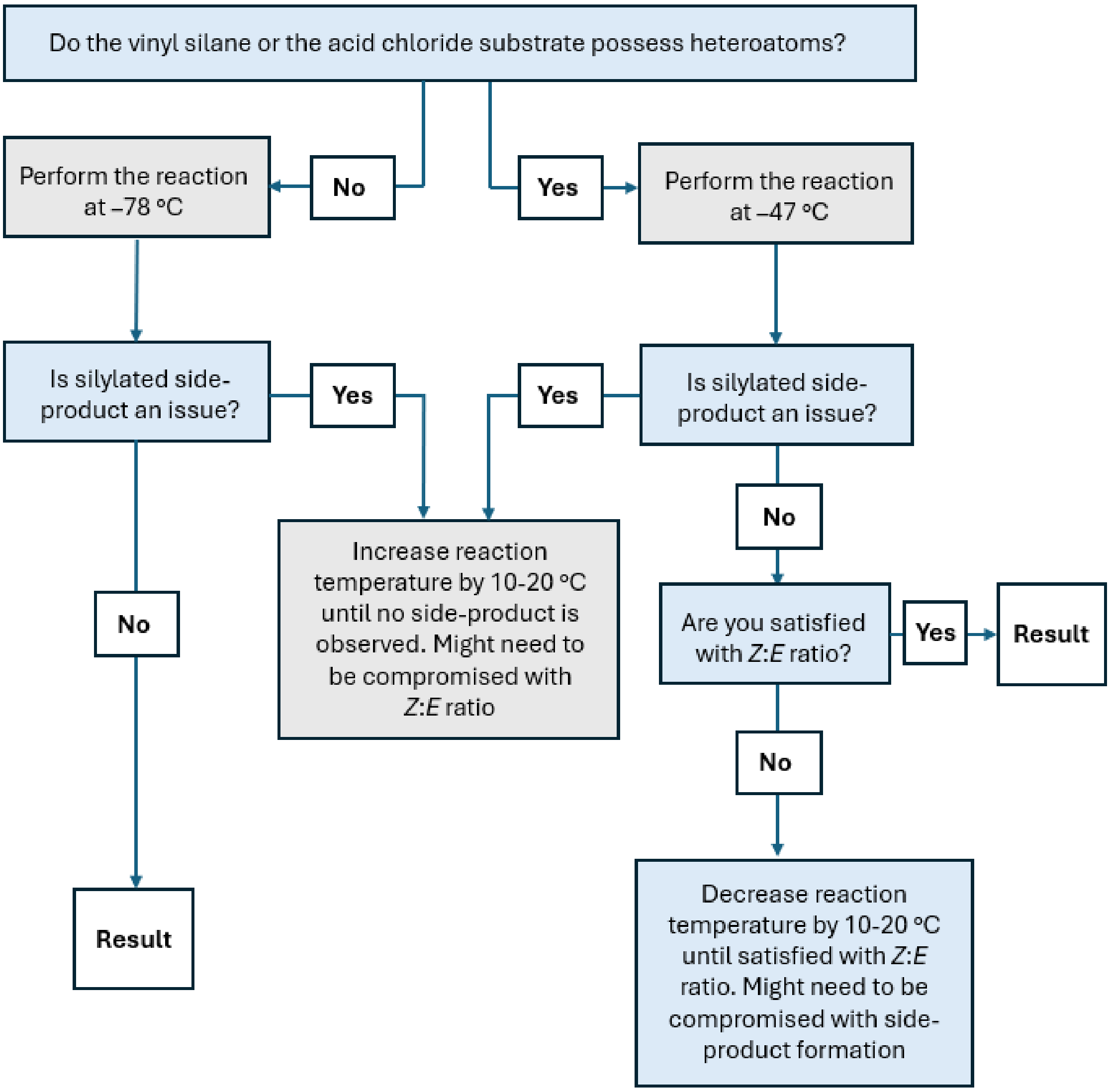

7. NMR spectra

2,2'-(Hex-5-yne-1,1-diyl)bis(4,4,5,5-tetramethyl-1,3,2-dioxaborolane) (1a)

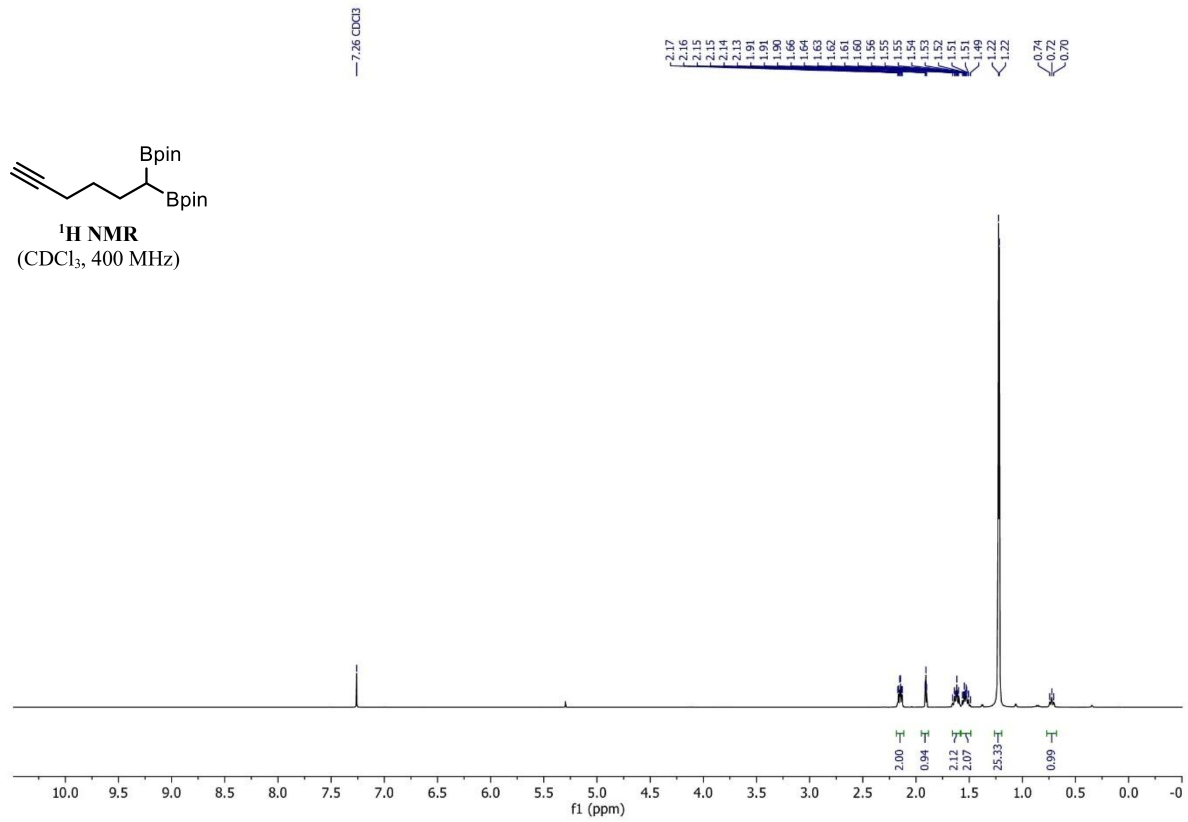

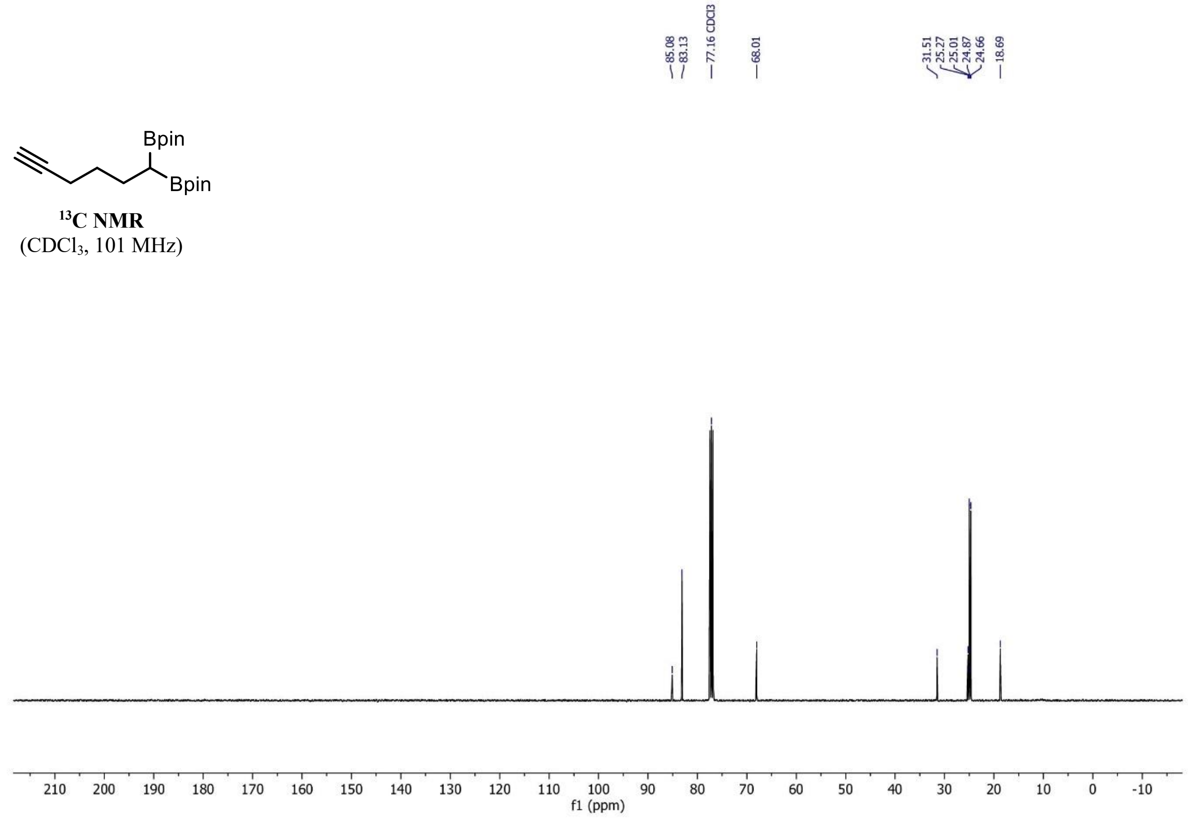

***N*-Benzyl-*N*-(hex-5-yn-1-yl)-2,4,6-tris(trifluoromethyl)benzenesulfonamide (1b)**

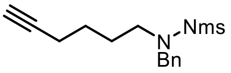

^{1}H NMR
($CDCl_3$, 400 MHz)

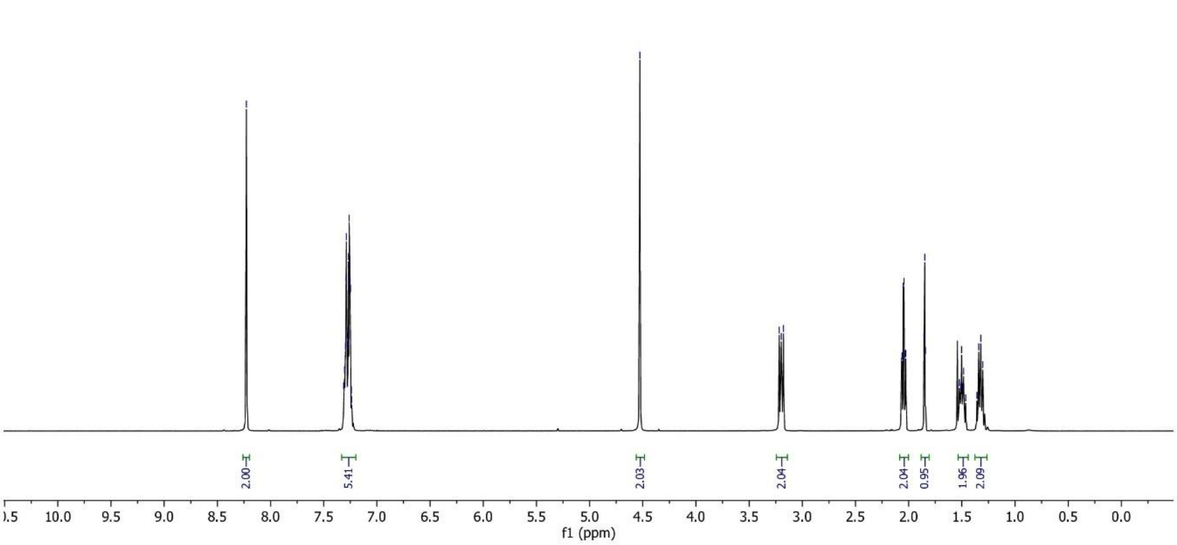

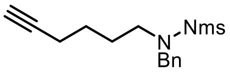

^{13}C NMR
($CDCl_3$, 101 MHz)

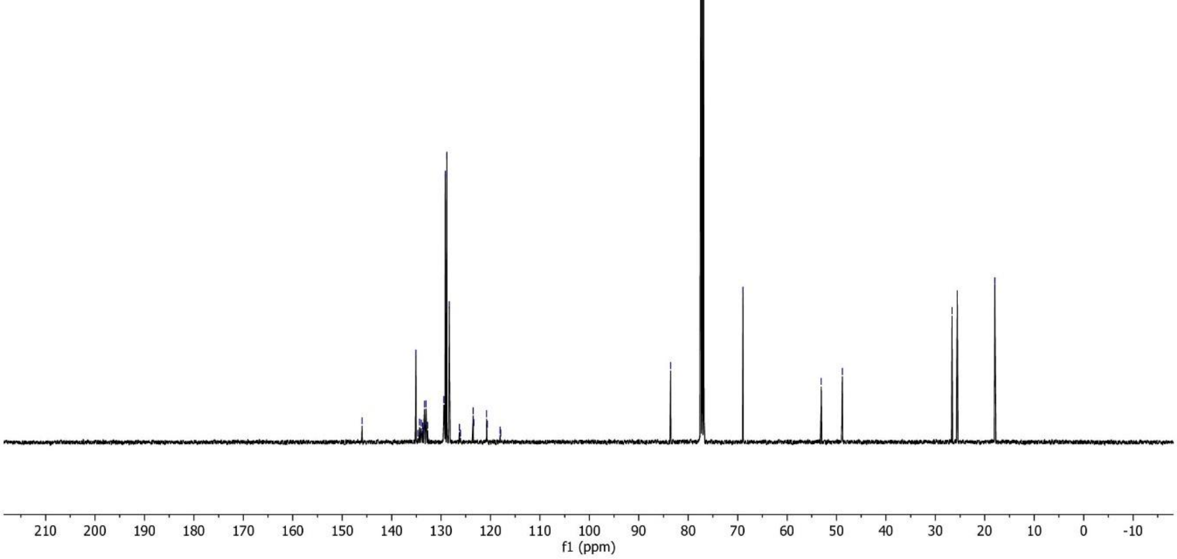

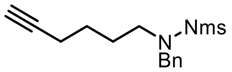

^{19}F NMR
($CDCl_3$, 376 MHz)

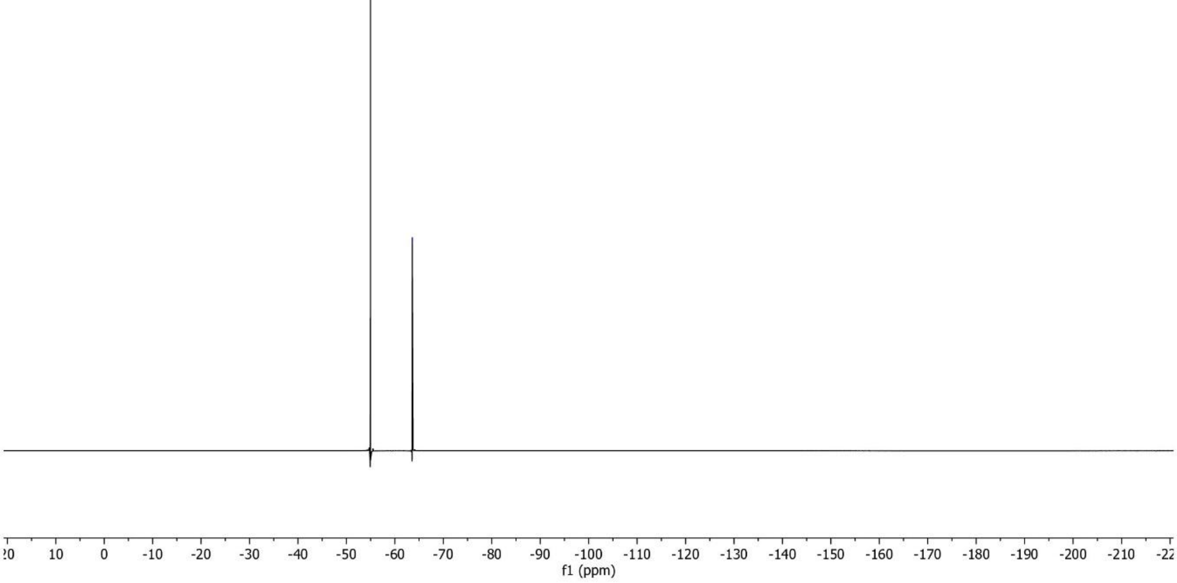

Ethyl (*E*)-tridec-2-en-12-ynoate (1c)

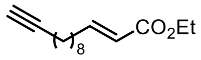

^{1}H NMR
($CDCl_3$, 400 MHz)

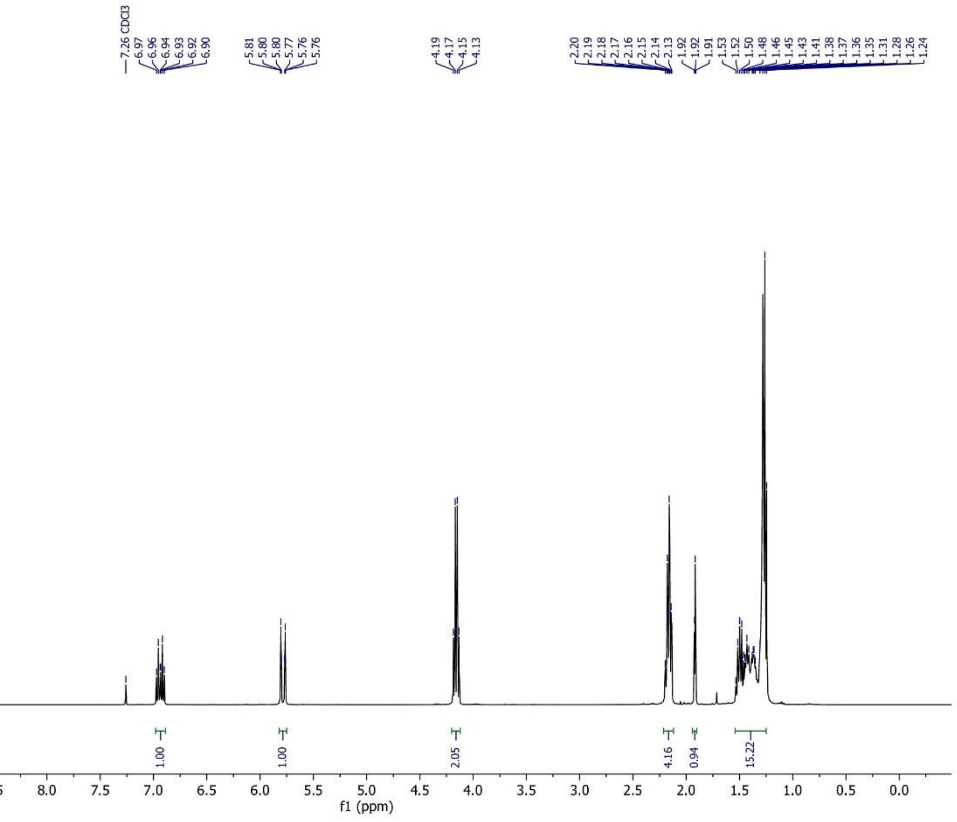

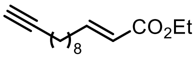

^{13}C NMR
($CDCl_3$, 101 MHz)

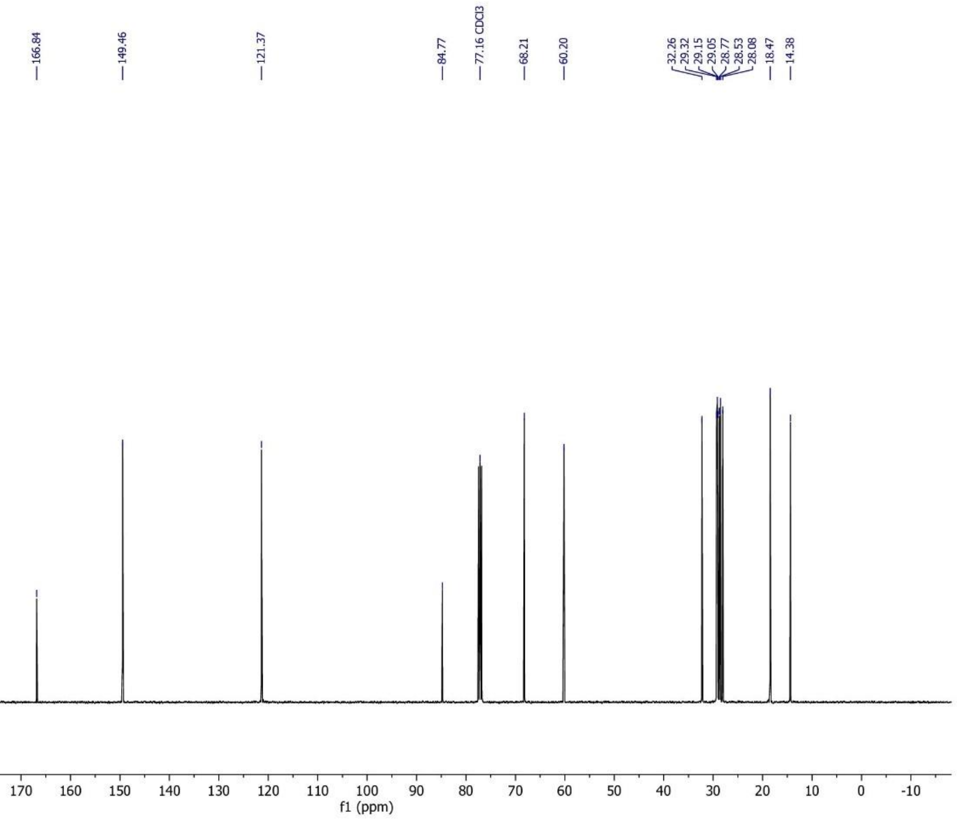

(3*R*,4*S*)-1-(4-Fluorophenyl)-3-((*S*)-3-(4-fluorophenyl)-3-hydroxypropyl)-4-(4-methoxyphenyl)azetidin-2-one (S3)

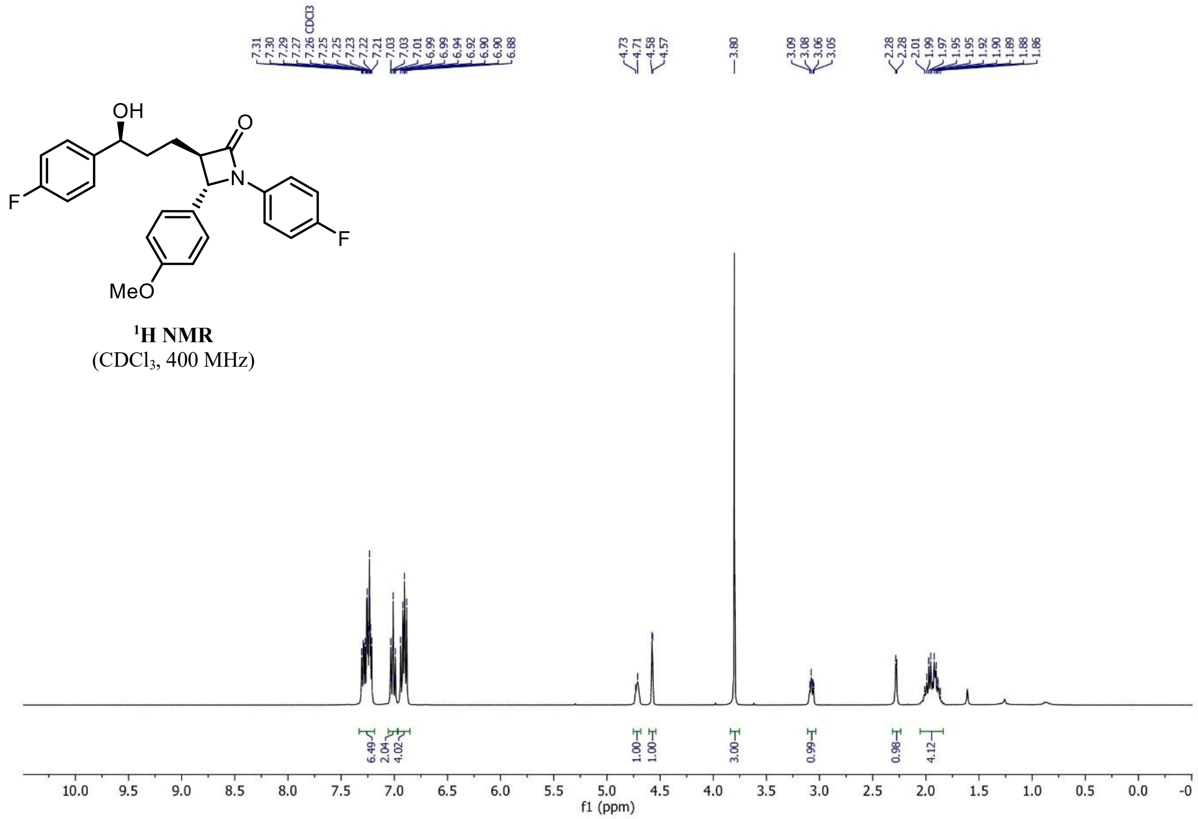

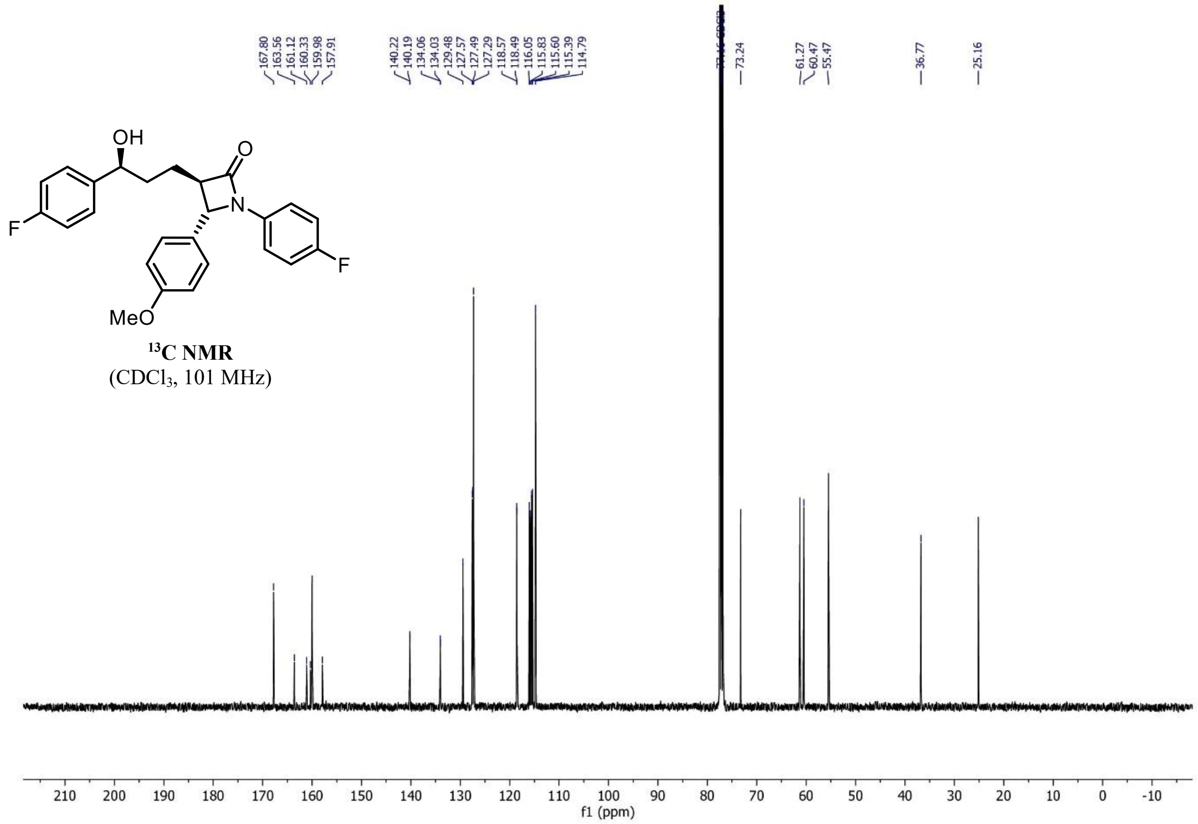

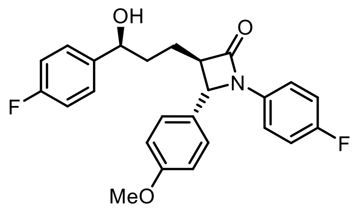

^{19}F NMR
($CDCl_3$, 376 MHz)

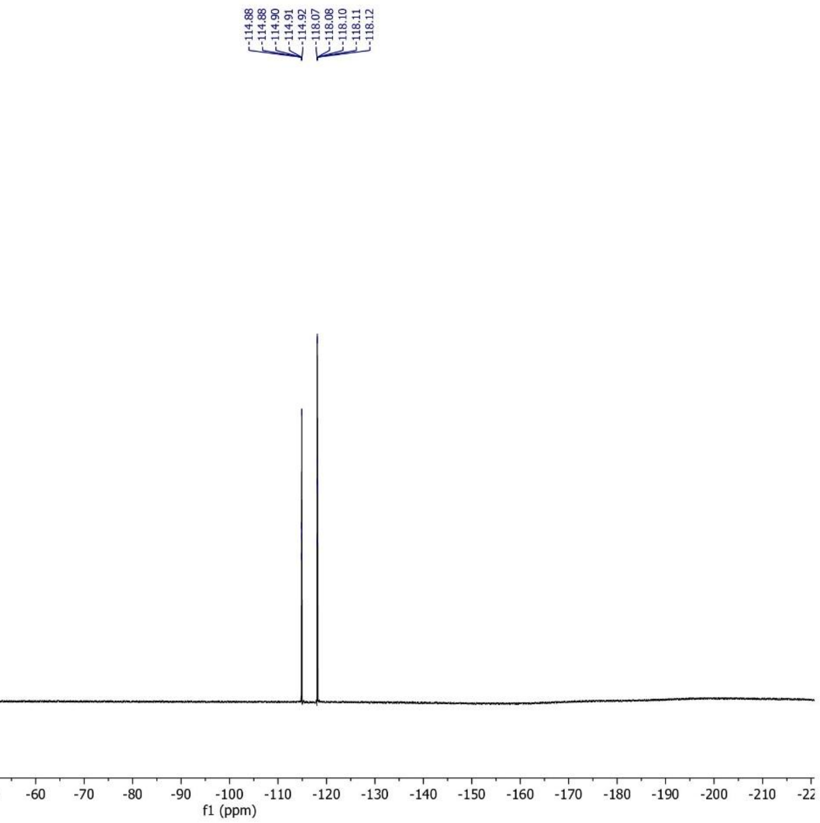

(*S*)-1-(4-Fluorophenyl)-3-((2*S*,3*R*)-1-(4-fluorophenyl)-2-(4-methoxyphenyl)-4-oxoazetidin-3-yl)propyl undec-10-ynoate (1d)

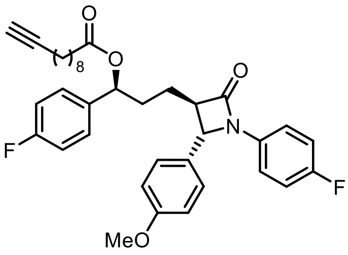

^{1}H NMR
($CDCl_3$, 400 MHz)

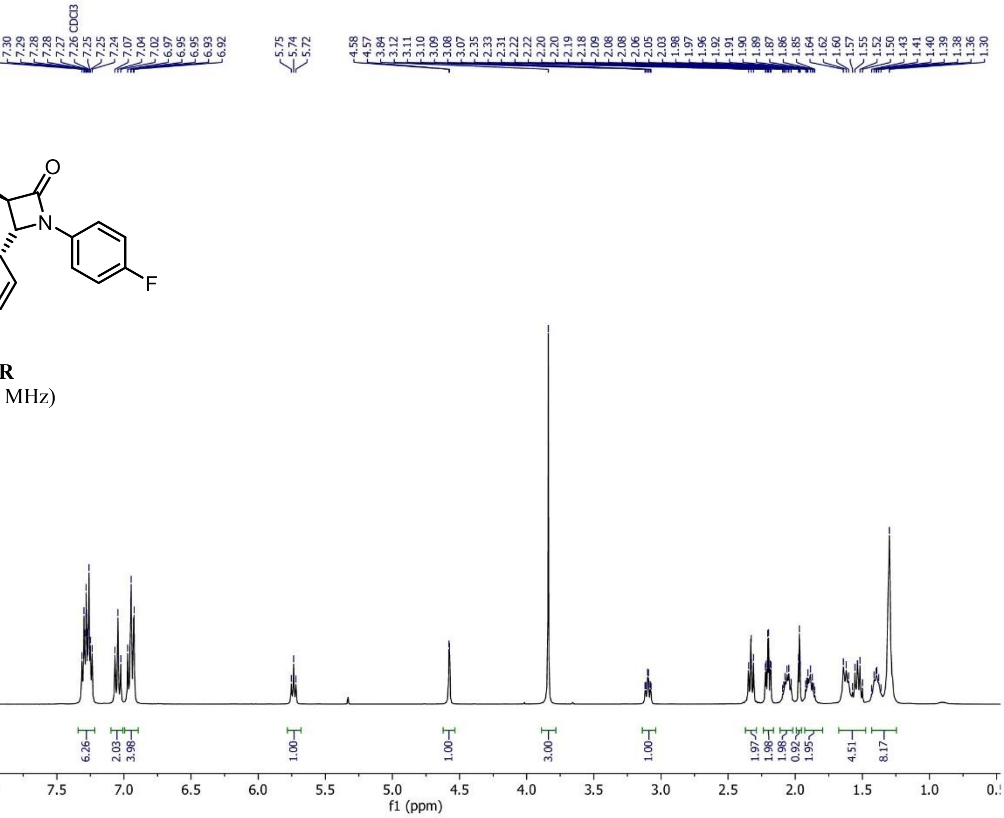

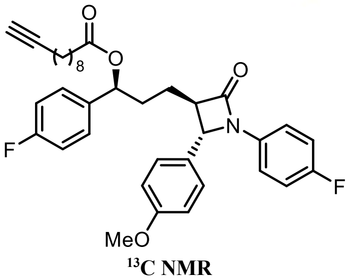

^{13}C NMR
($CDCl_3$, 101 MHz)

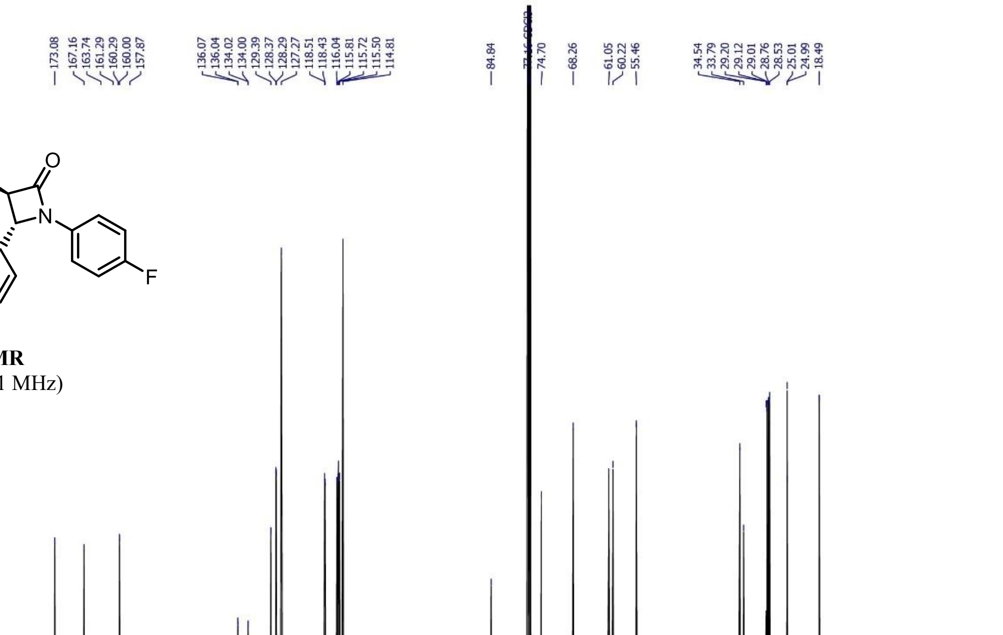

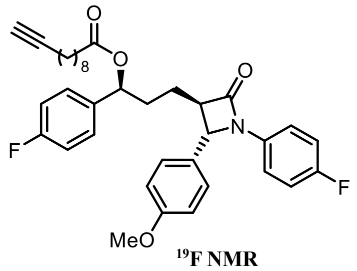

^{19}**F NMR**
($CDCl_3$, 376 MHz)

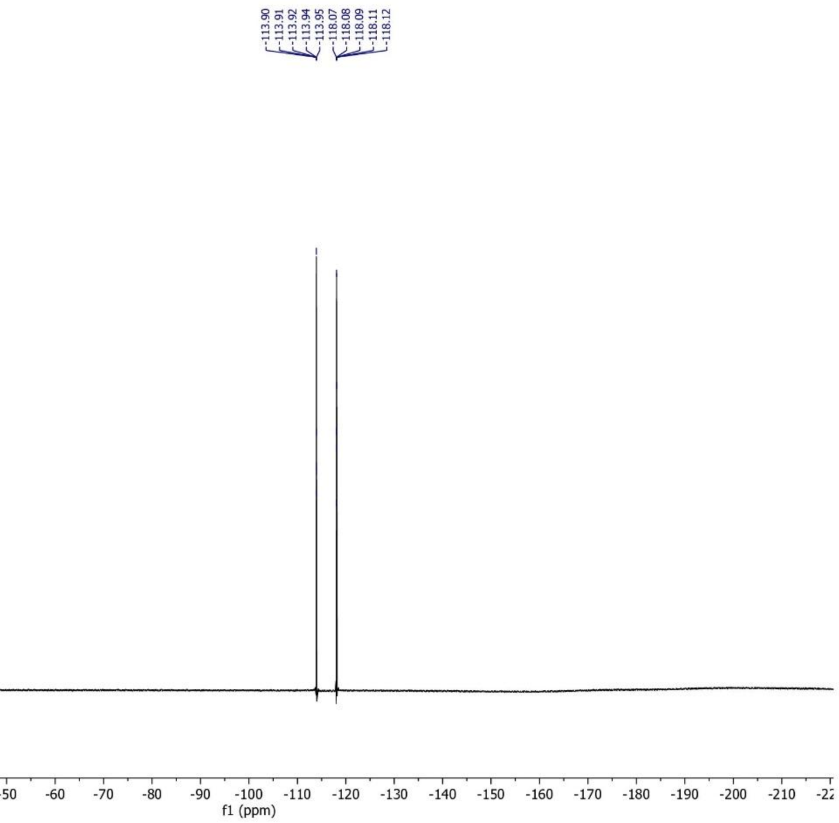

Methyl tridec-12-ynoate (1e)

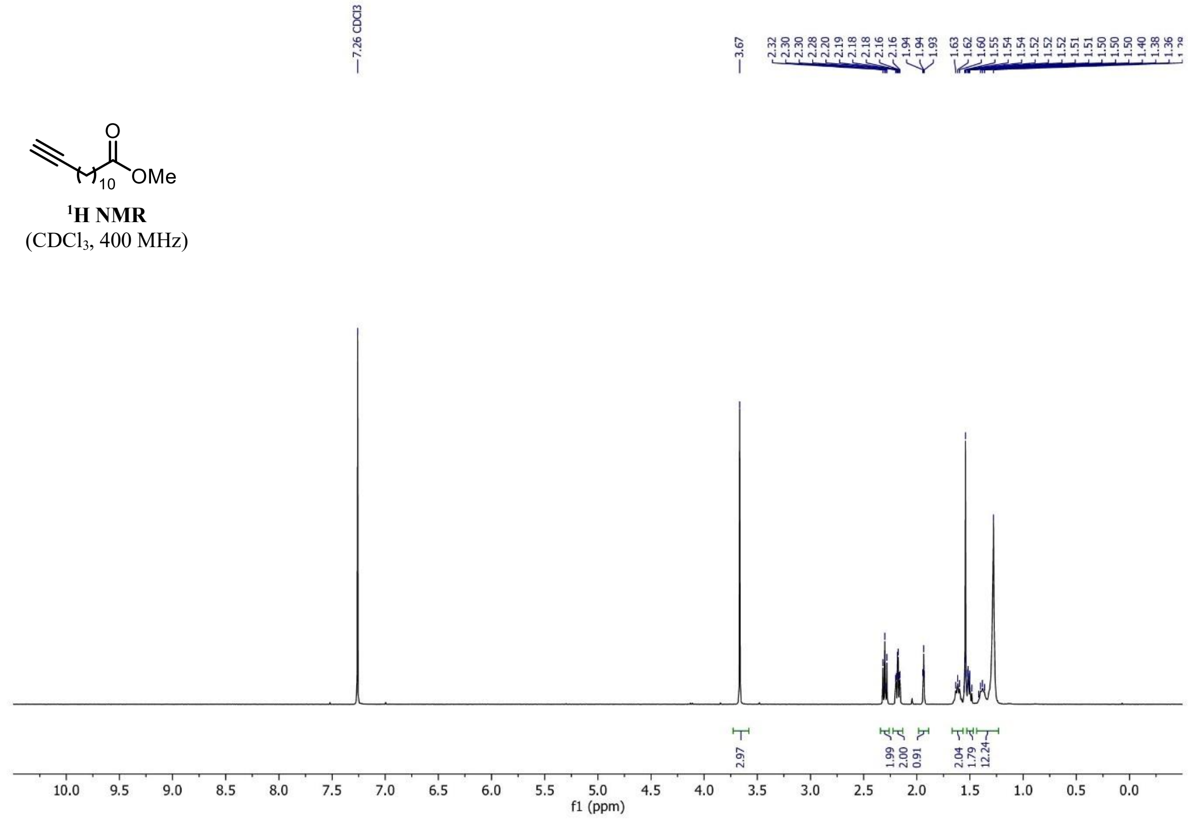

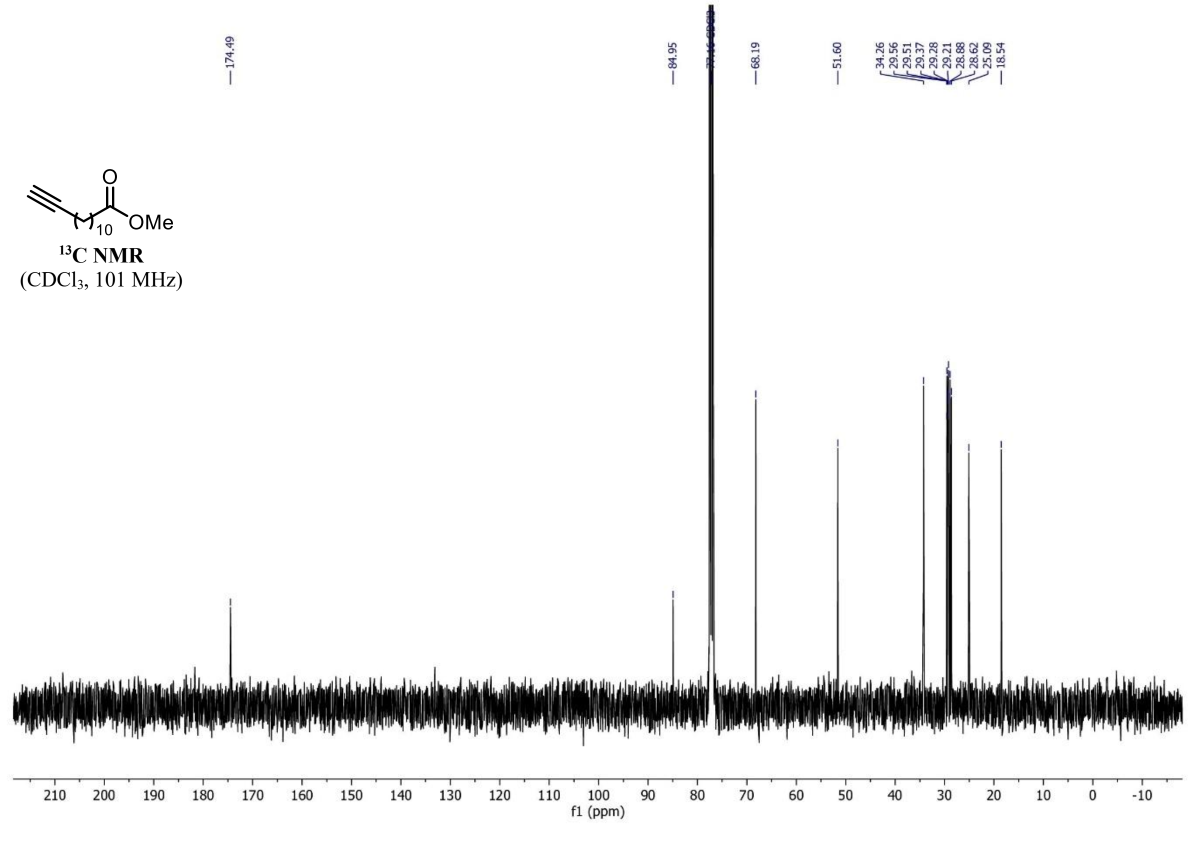

Trimethyl(oct-1-en-2-yl)silane (2aa)

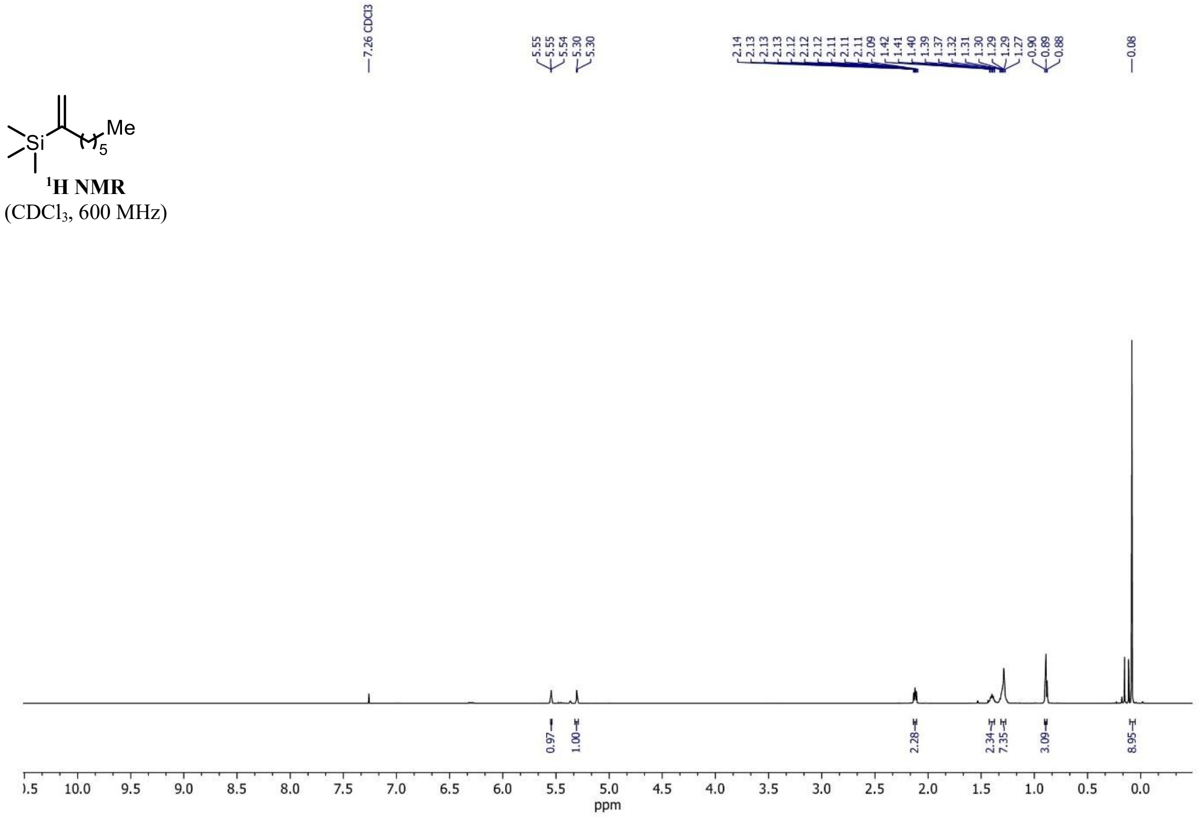

^{1}H NMR
($CDCl_3$, 600 MHz)

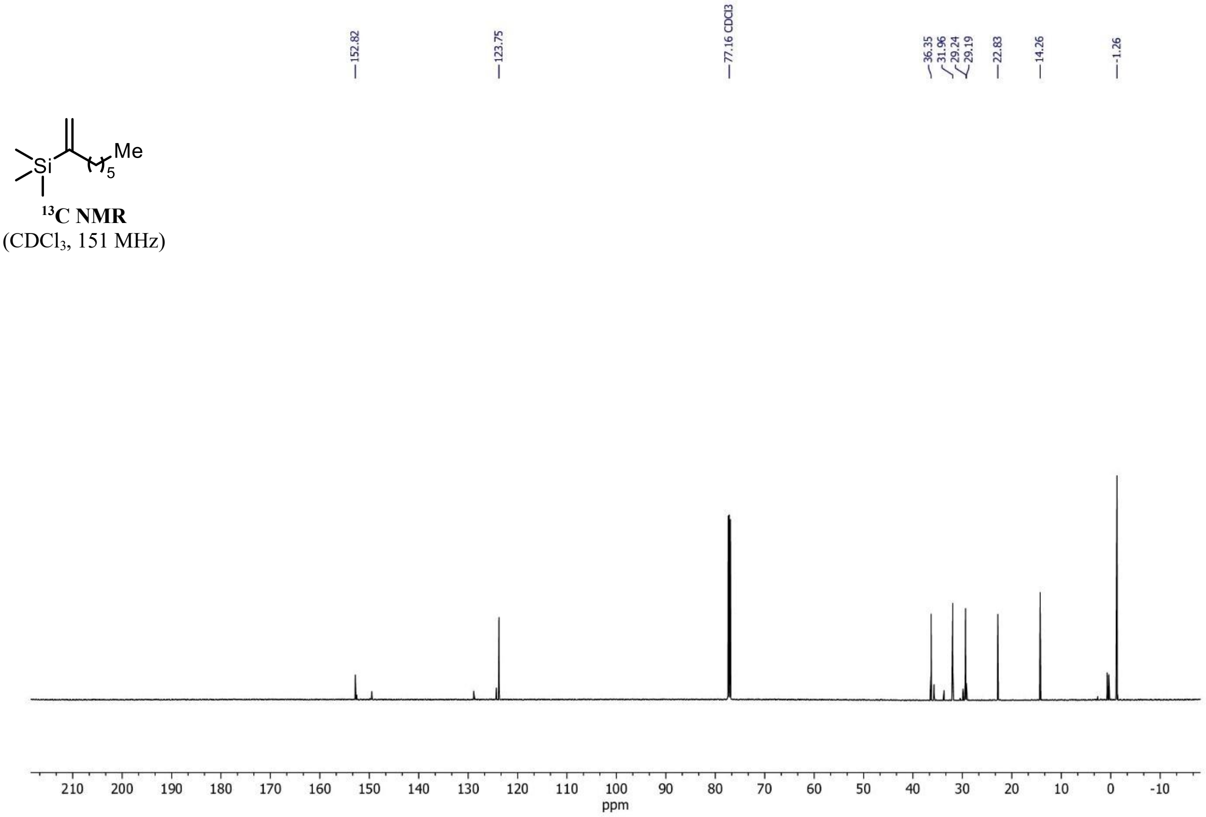

^{13}C NMR
($CDCl_3$, 151 MHz)

Trihexyl(oct-1-en-2-yl)silane (2ac)

^{1}**H NMR**
($CDCl_3$, 600 MHz)

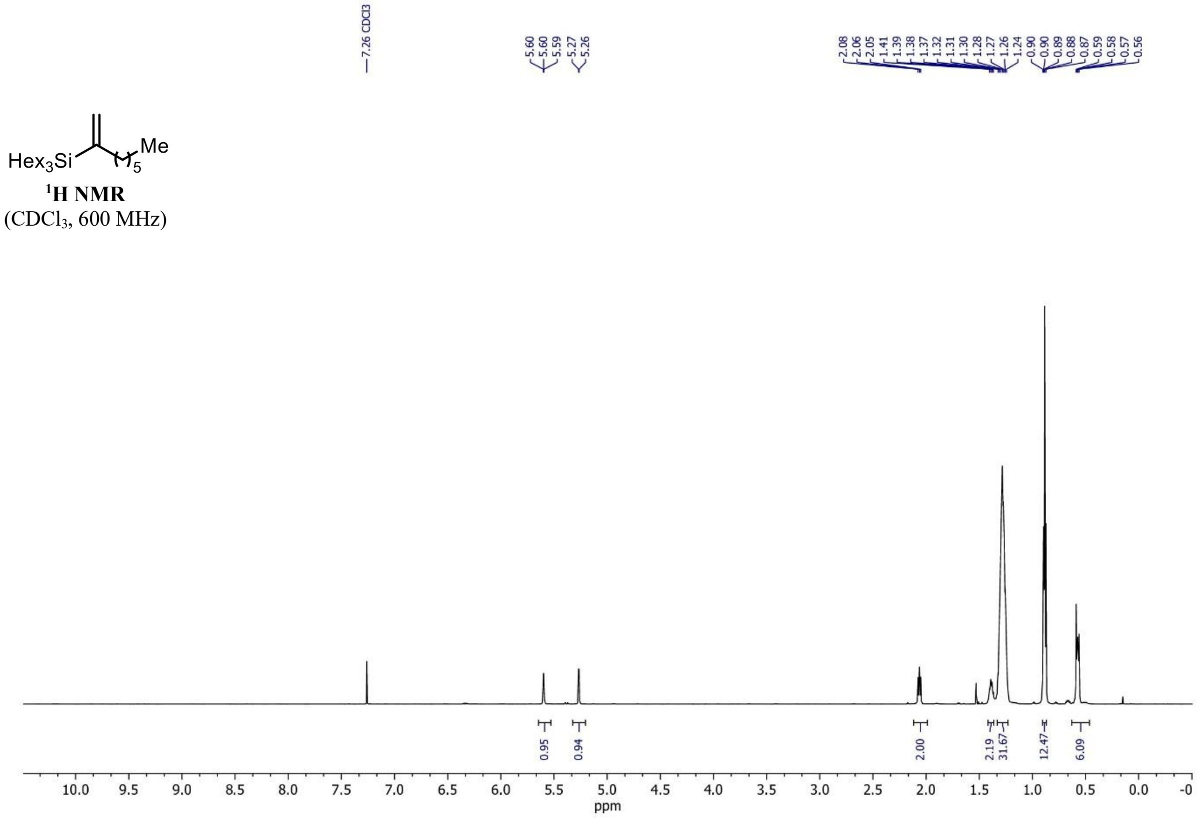

^{13}**C NMR**
($CDCl_3$, 151 MHz)

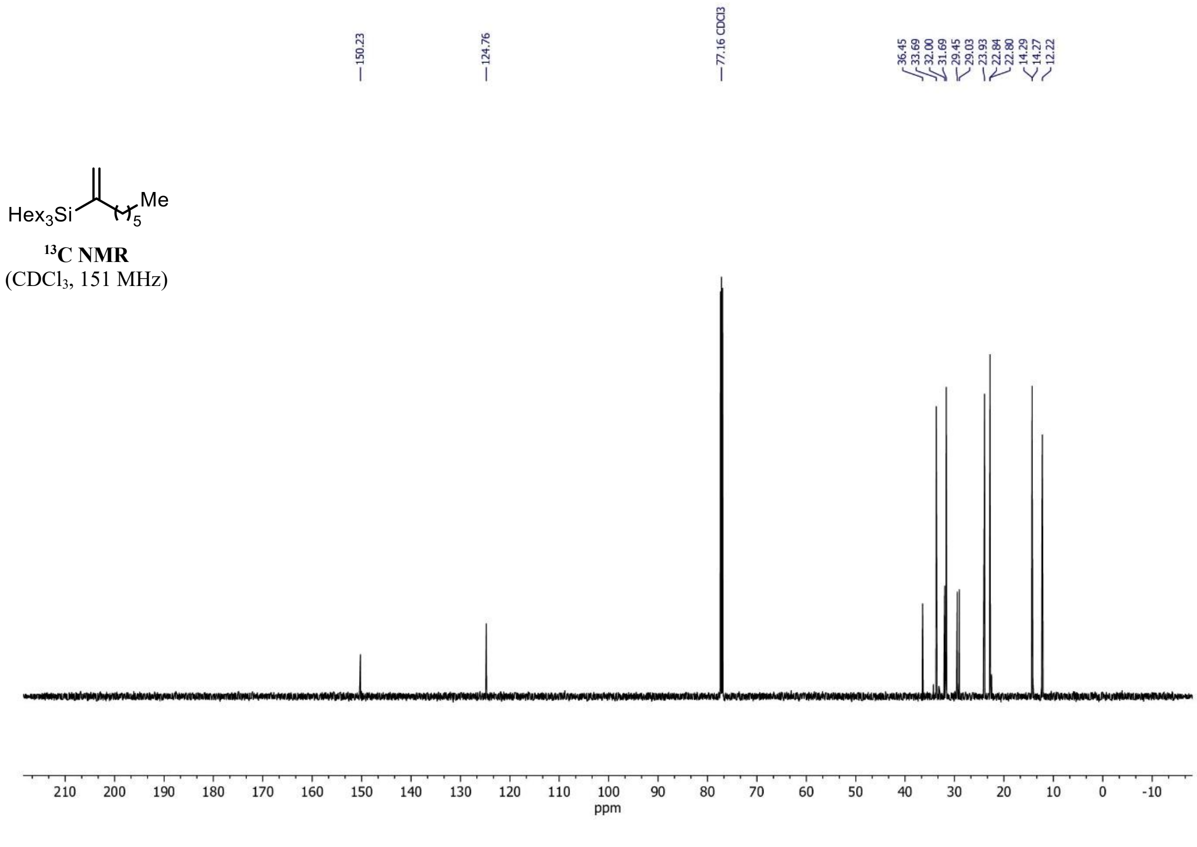

6-(Triethylsilyl)hept-6-enoic acid (S5)

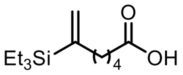

^{1}H NMR
($CDCl_3$, 400 MHz)

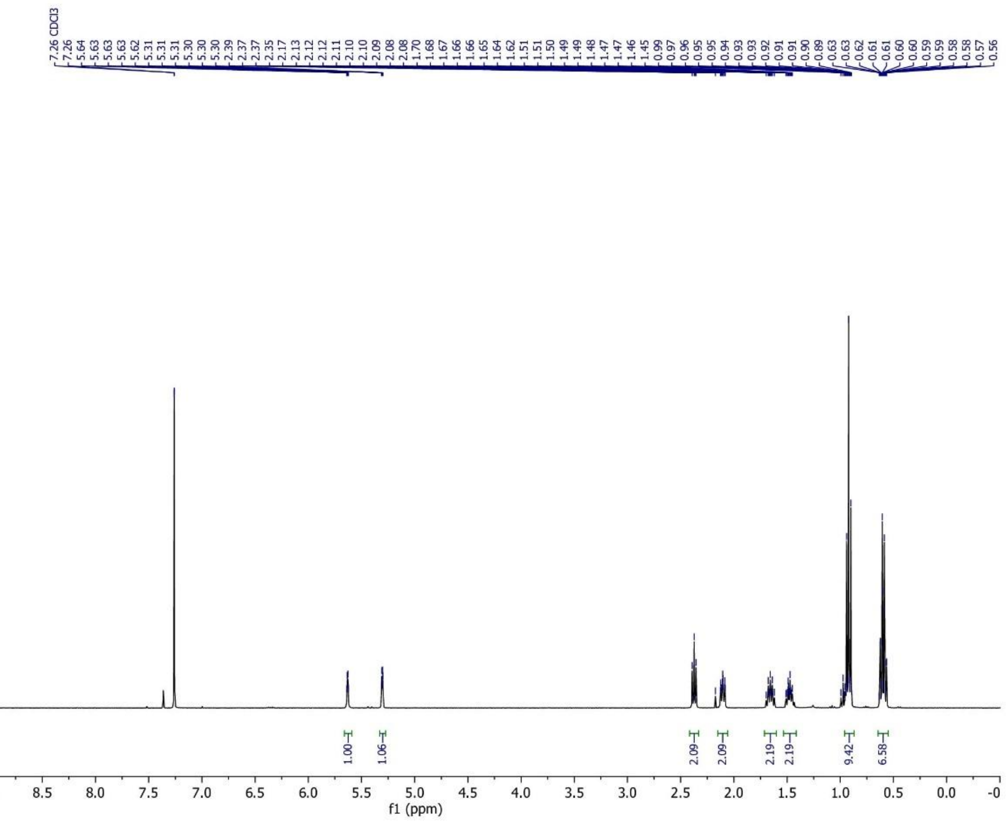

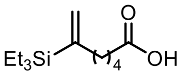

^{13}C NMR
($CDCl_3$, 101 MHz)

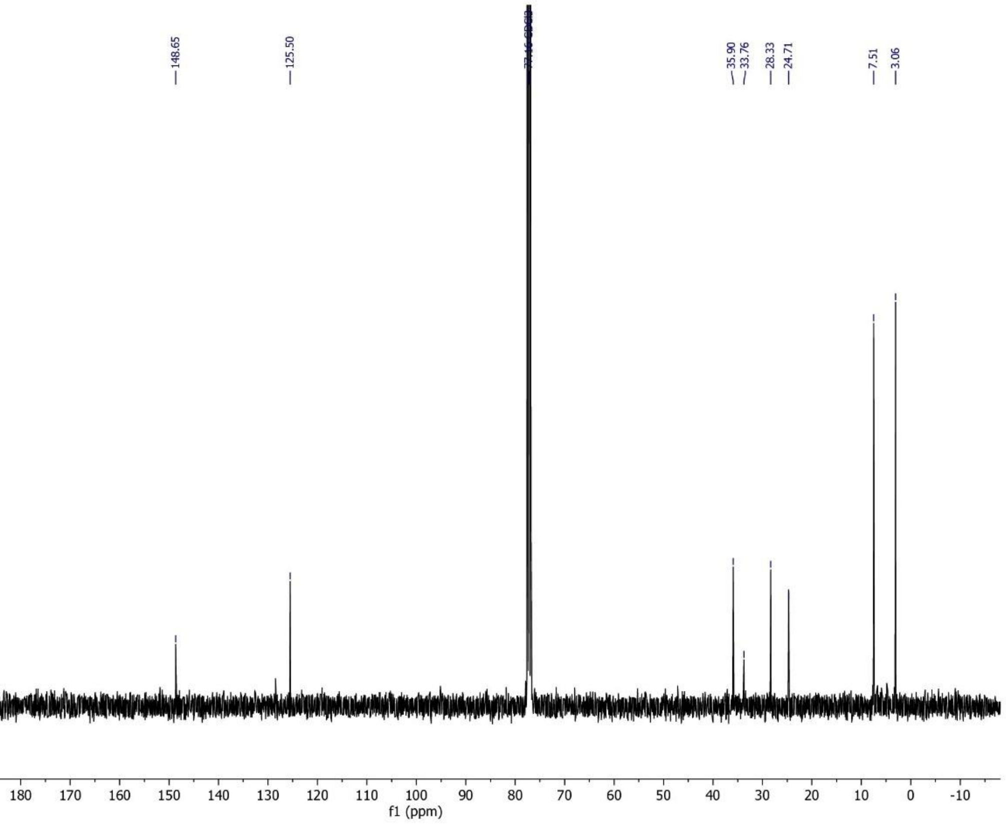

Methyl 6-(triethylsilyl)hept-6-enoate (2ba)

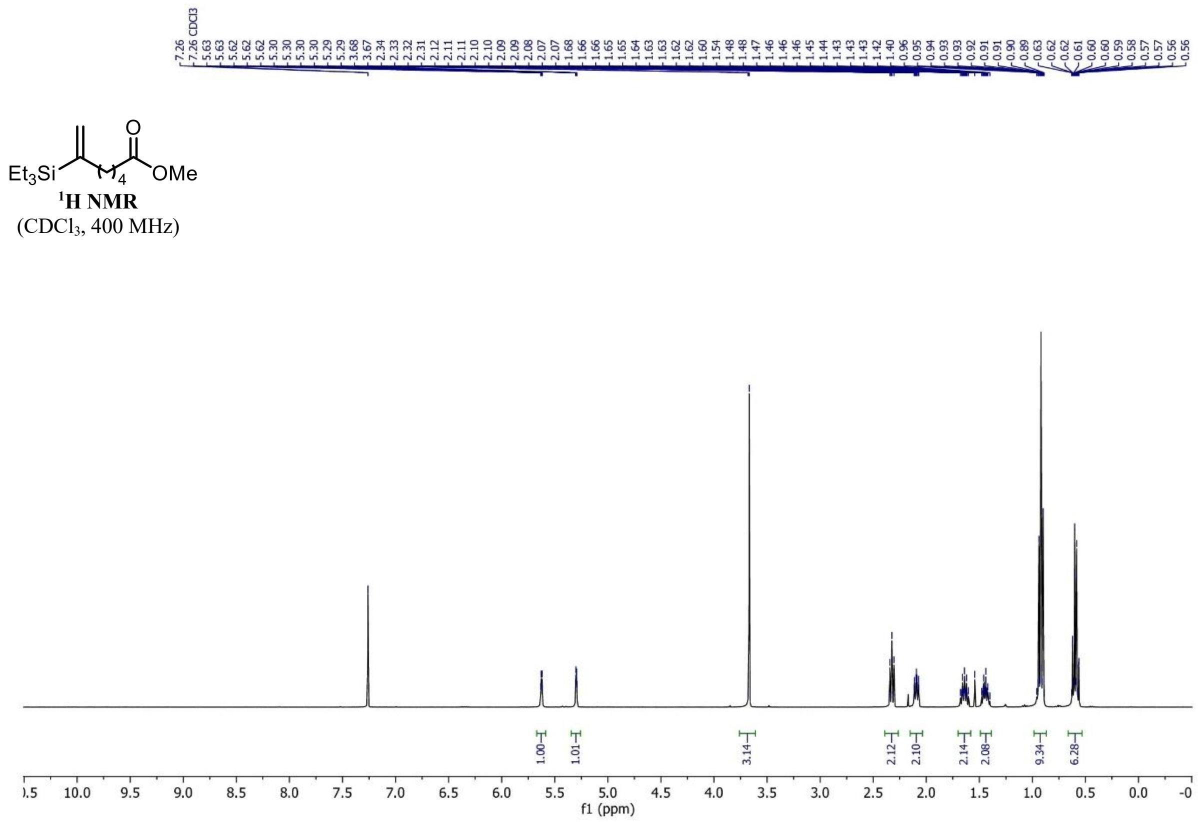

^{1}H NMR
($CDCl_3$, 400 MHz)

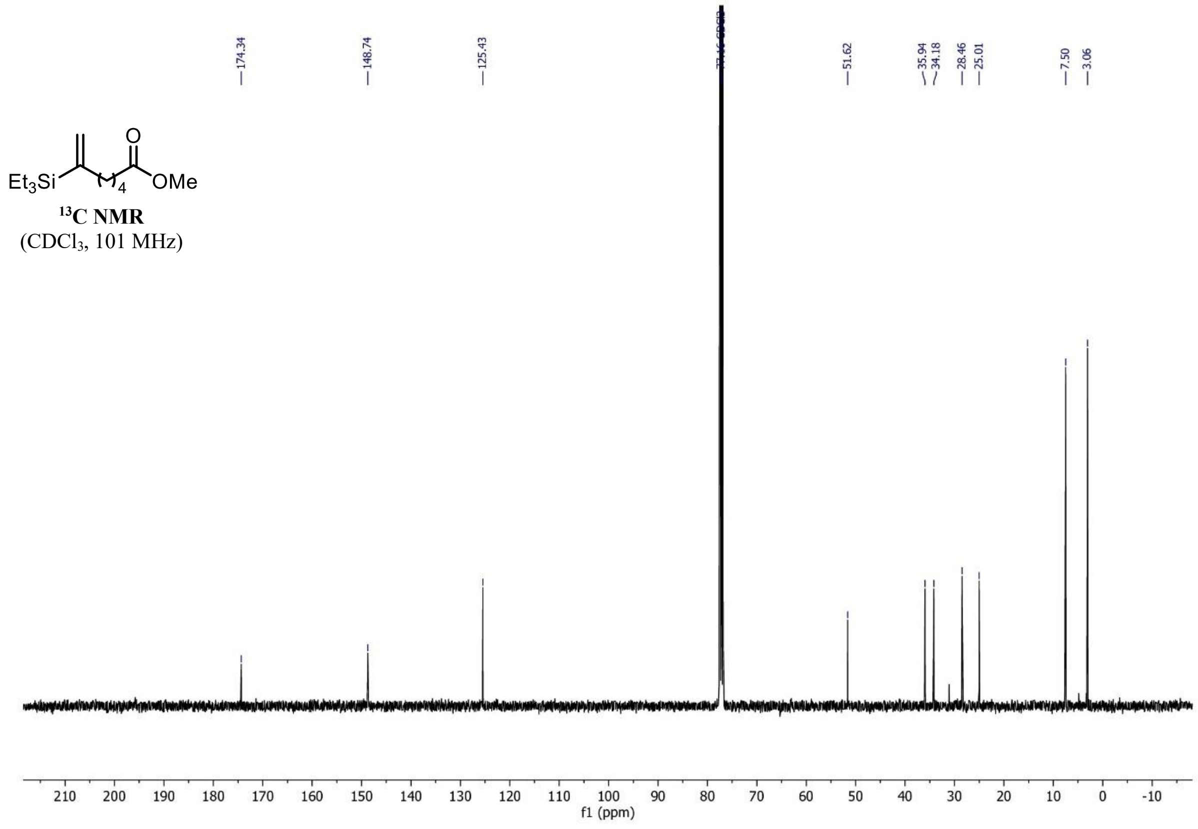

^{13}C NMR
($CDCl_3$, 101 MHz)

6-(Trihexylsilyl)hept-6-enoic acid (S6)

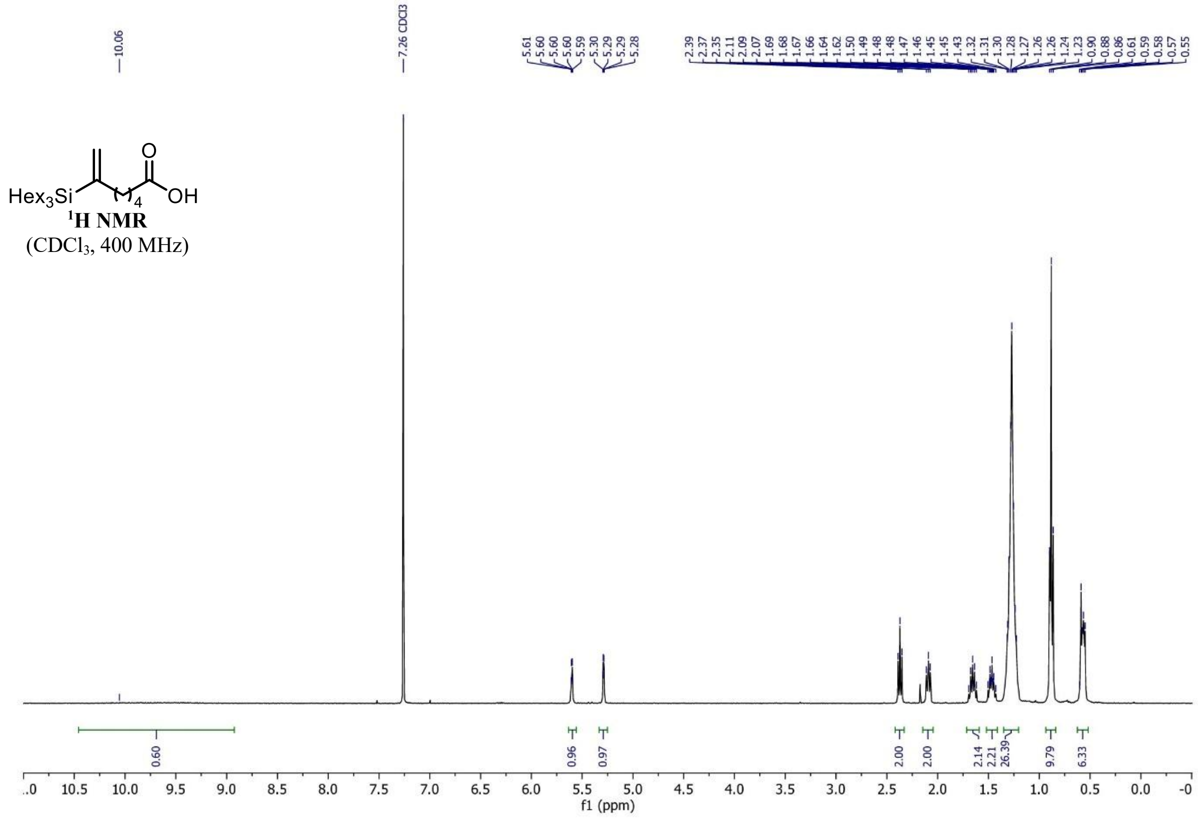

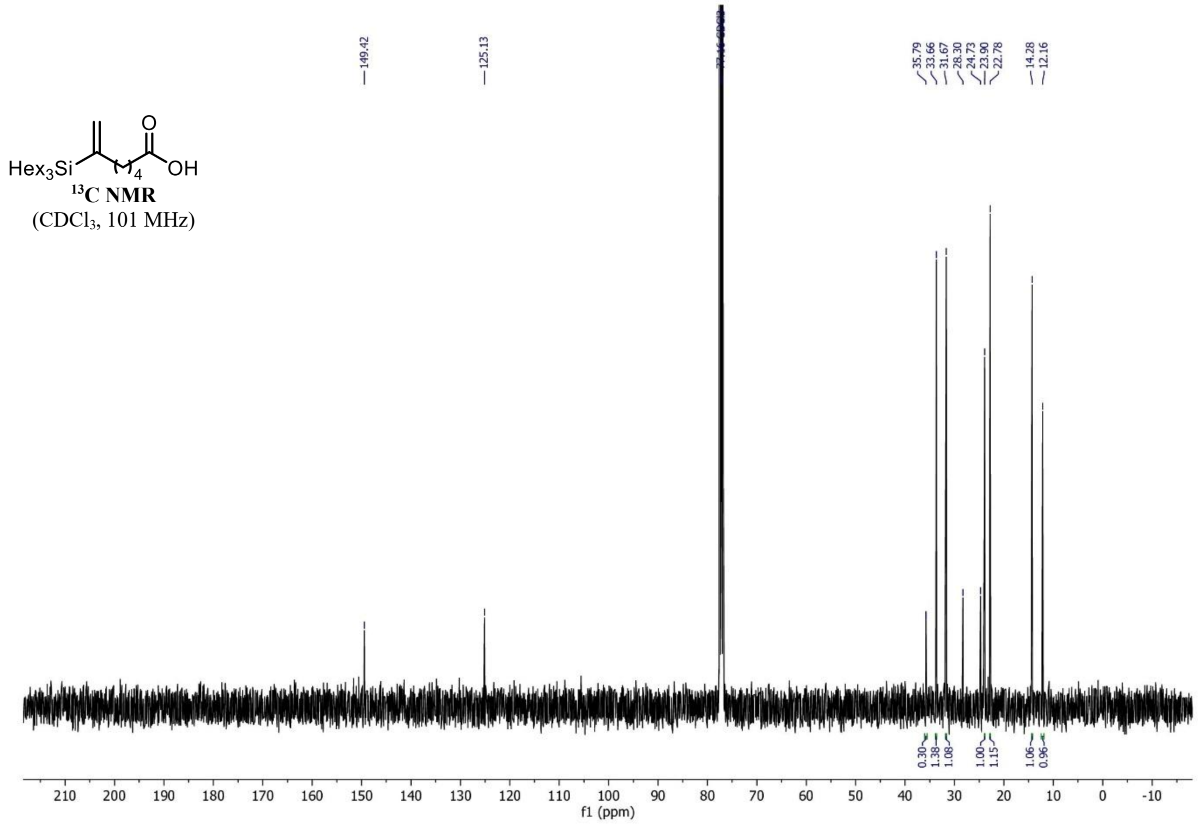

Methyl 6-(trihexylsilyl)hept-6-enoate (2bb)

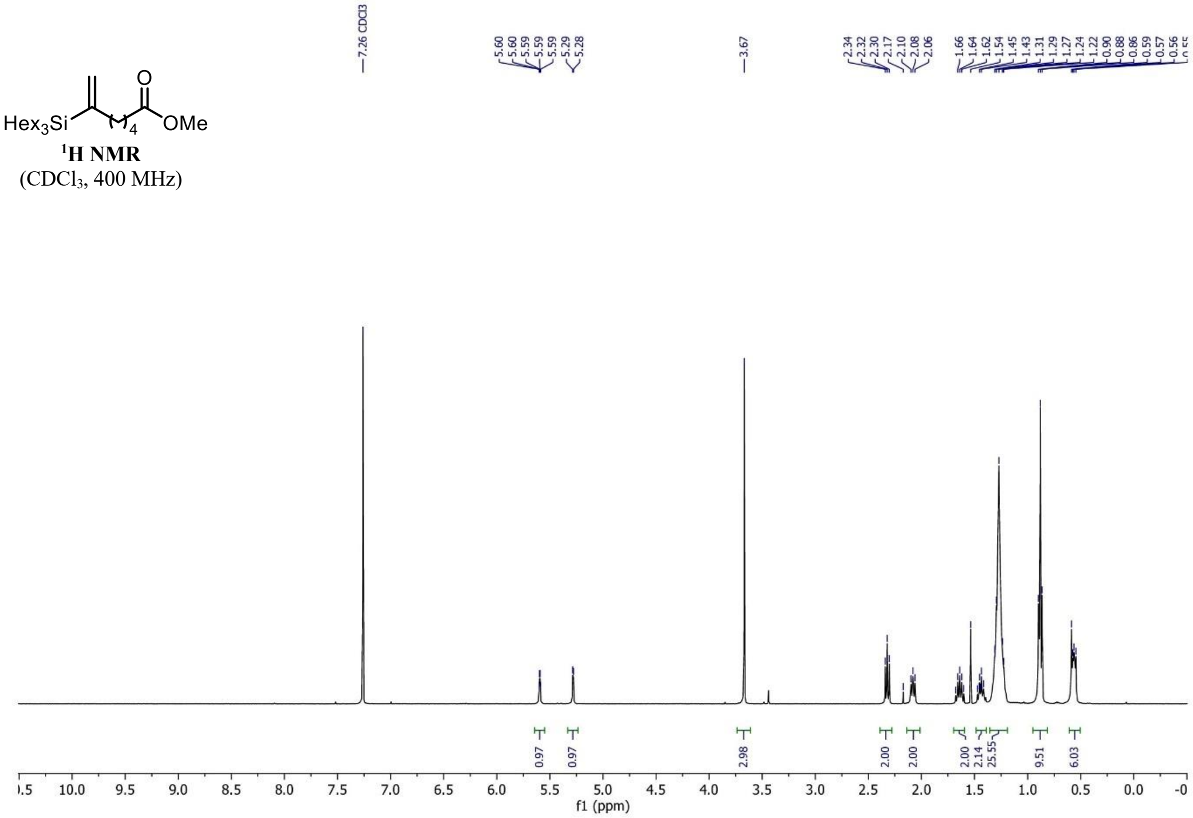

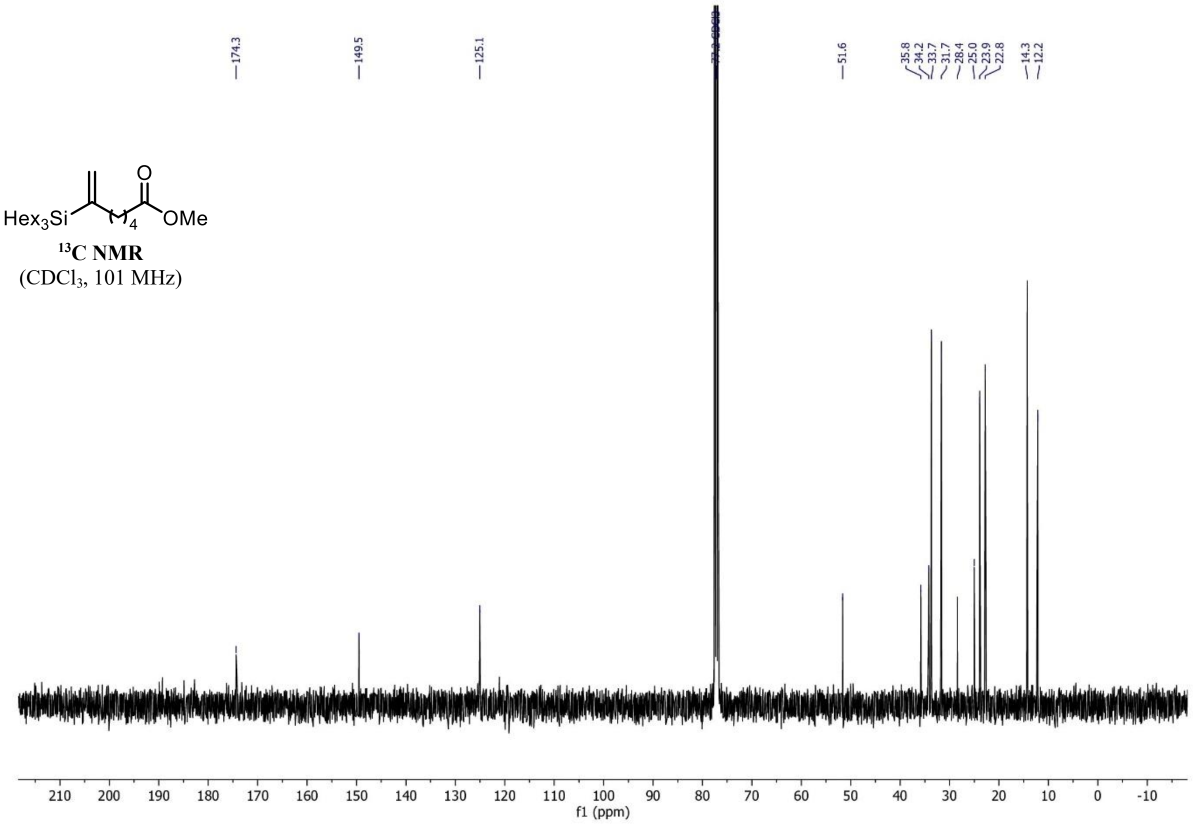

Triethyl(5-iodopent-1-en-2-yl)silane (2d)

^{1}H NMR
($CDCl_3$, 400 MHz)

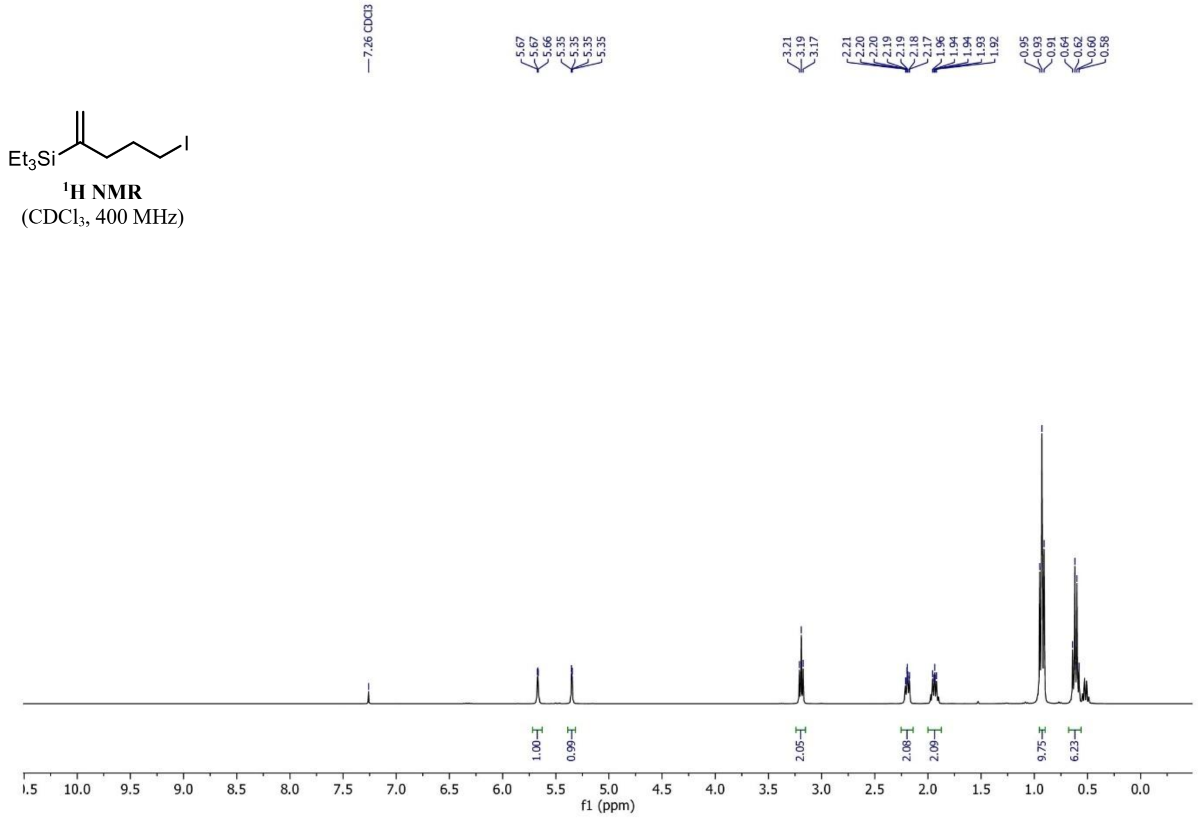

^{13}C NMR
($CDCl_3$, 101 MHz)

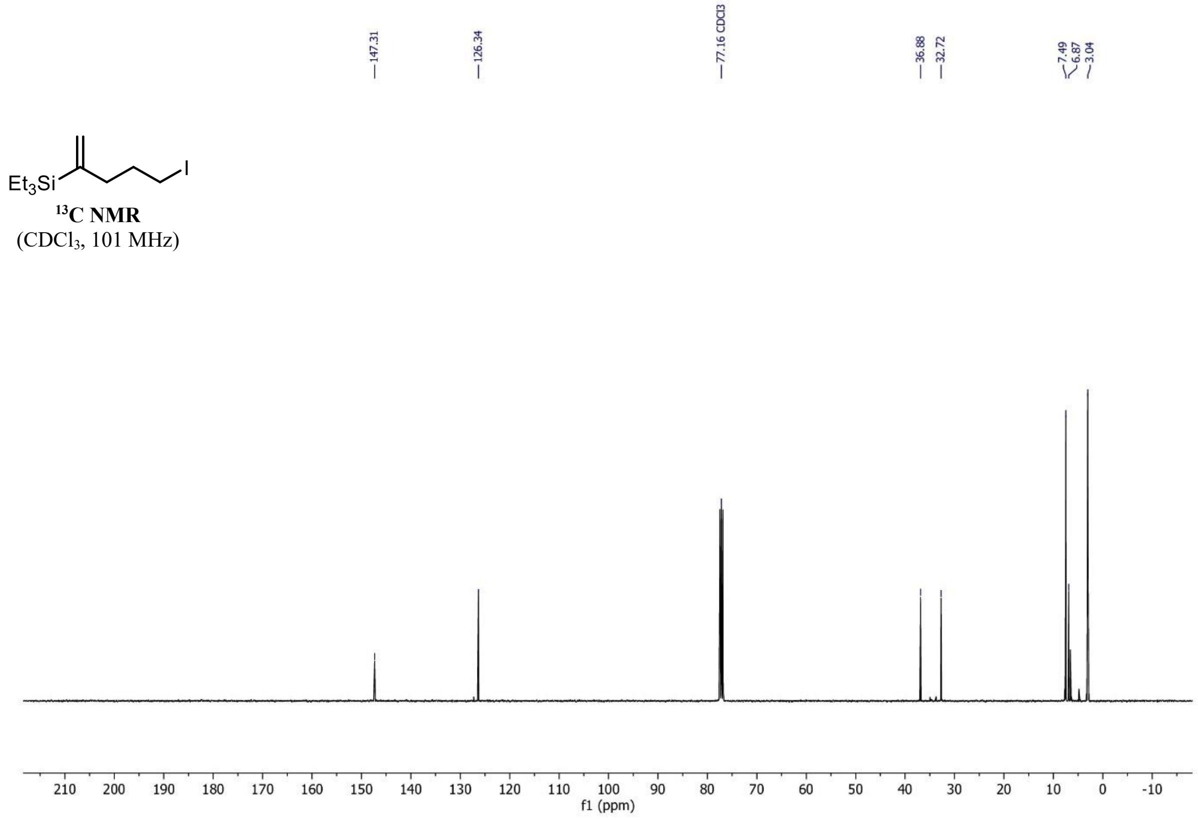

(6,6-Bis(4,4,5,5-tetramethyl-1,3,2-dioxaborolan-2-yl)hex-1-en-2-yl)triethylsilane (2e)

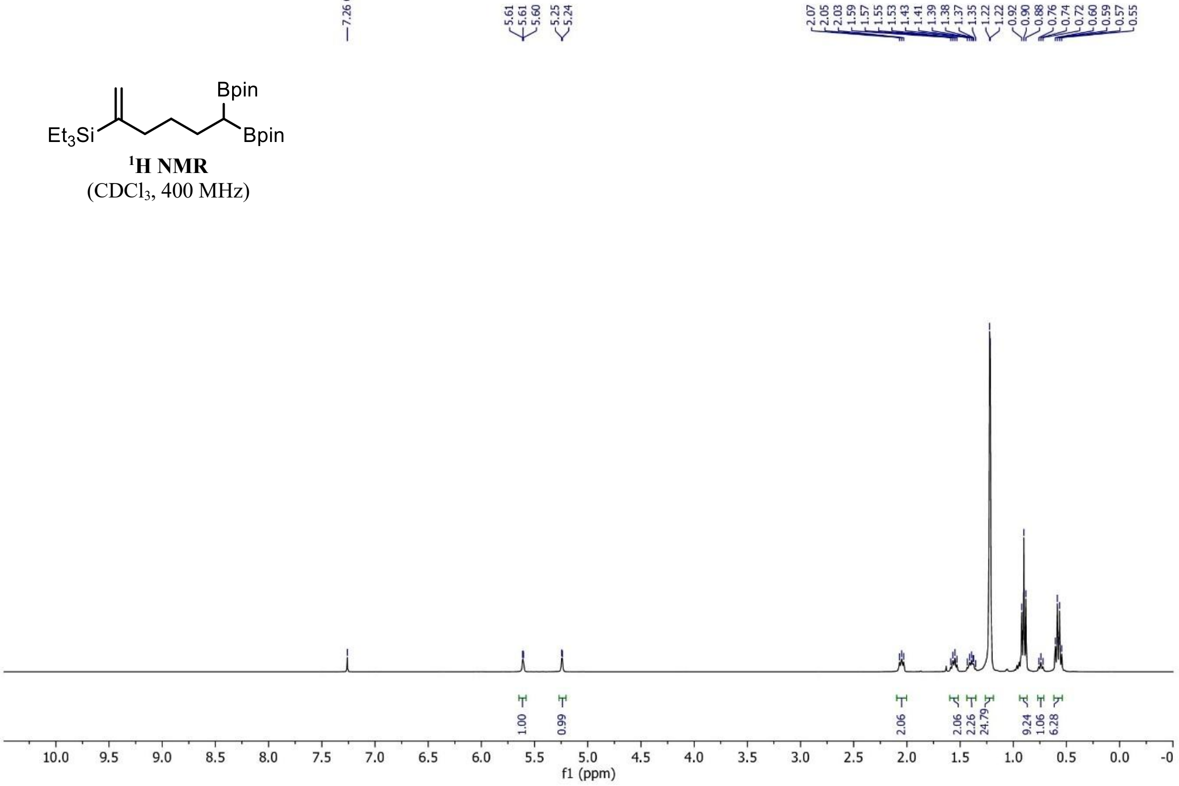

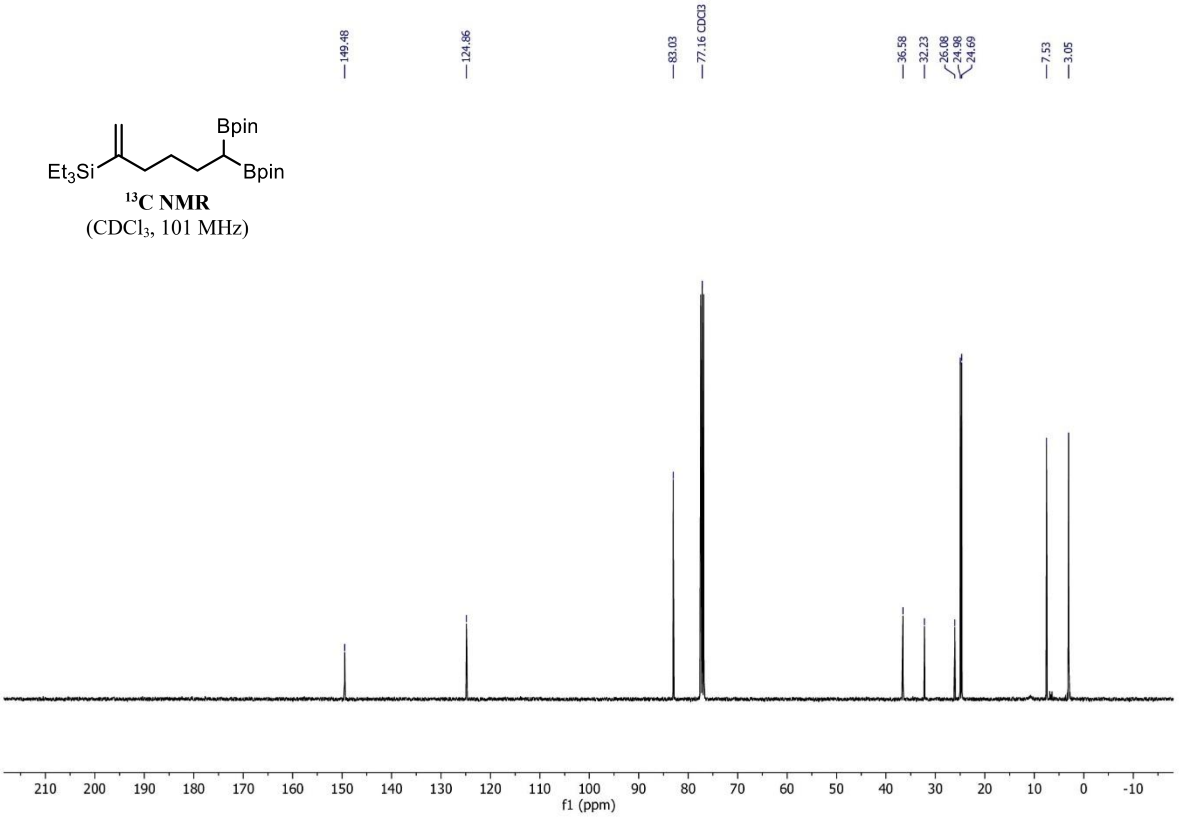

***N*-Benzyl-*N*-(5-(triethylsilyl)hex-5-en-1-yl)-2,4,6-tris(trifluoromethyl)benzenesulfonamide (2f)**

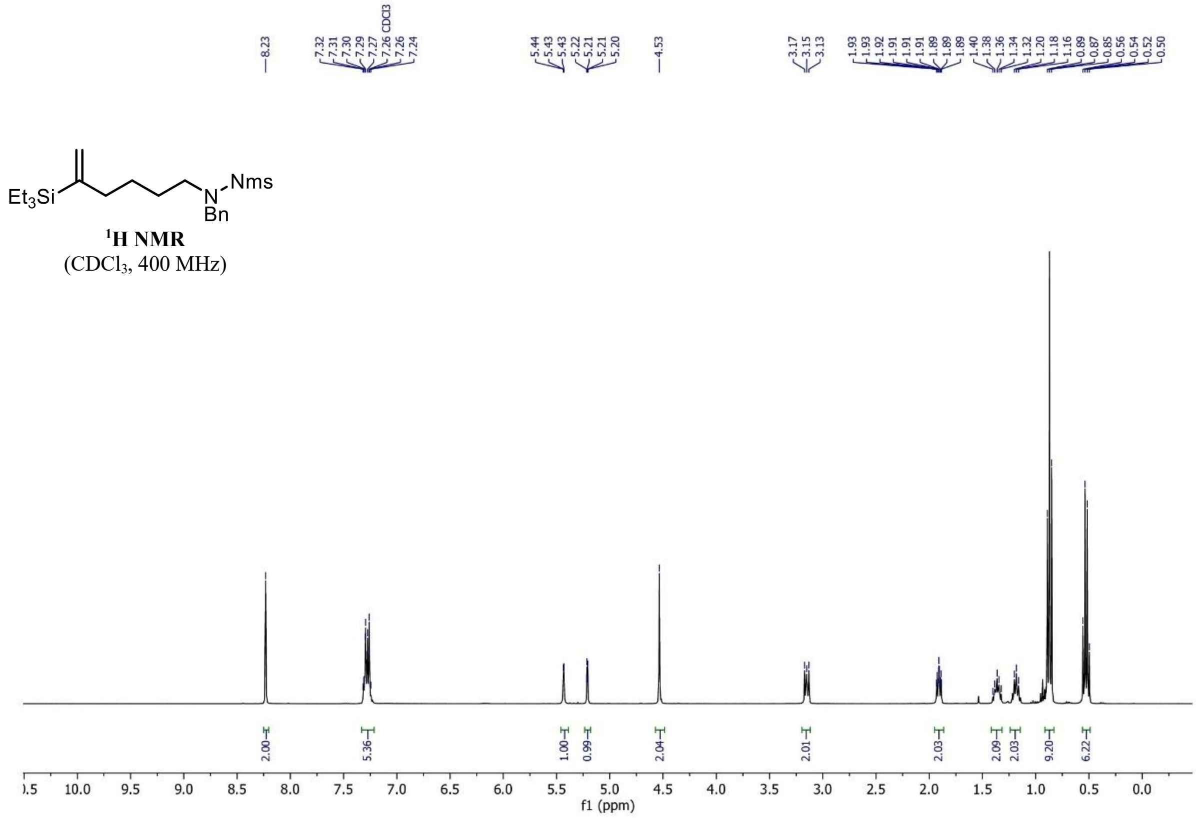

^{1}H NMR
($CDCl_3$, 400 MHz)

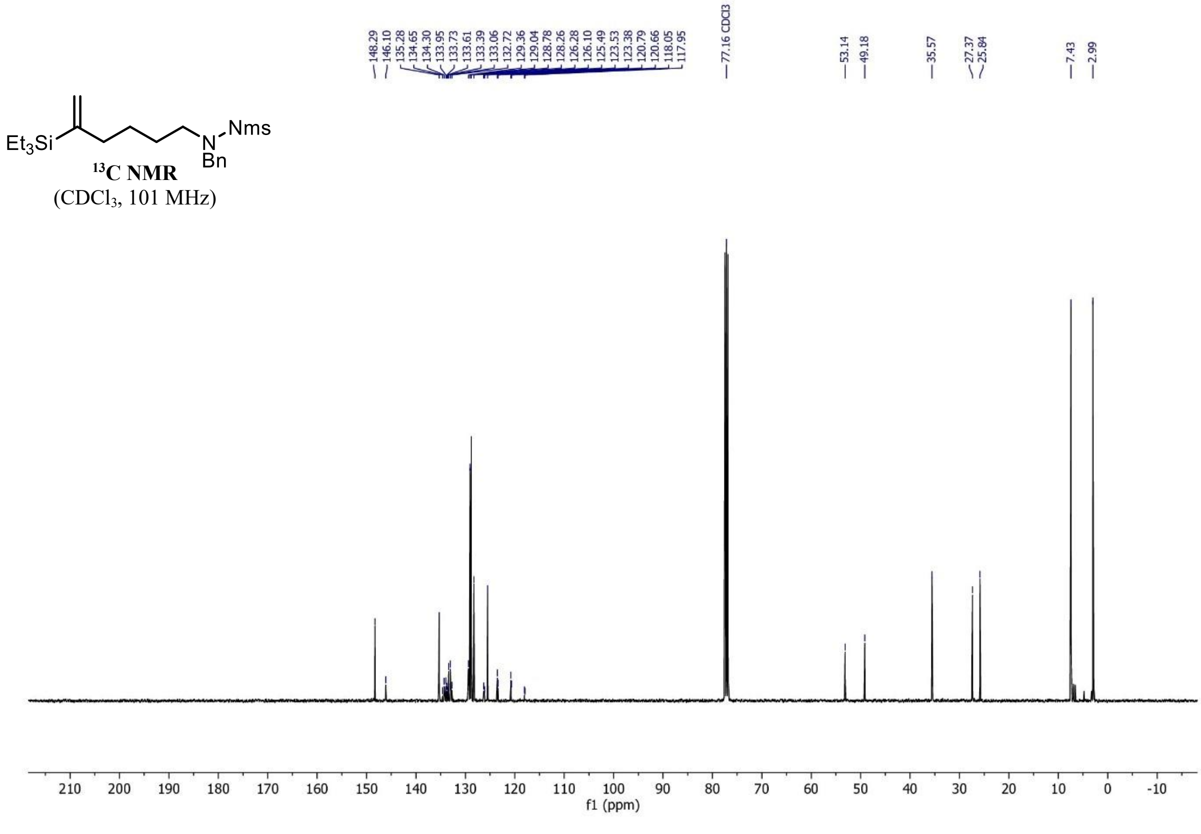

^{13}C NMR
($CDCl_3$, 101 MHz)

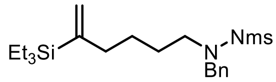

^{19}F NMR
($CDCl_3$, 376 MHz)

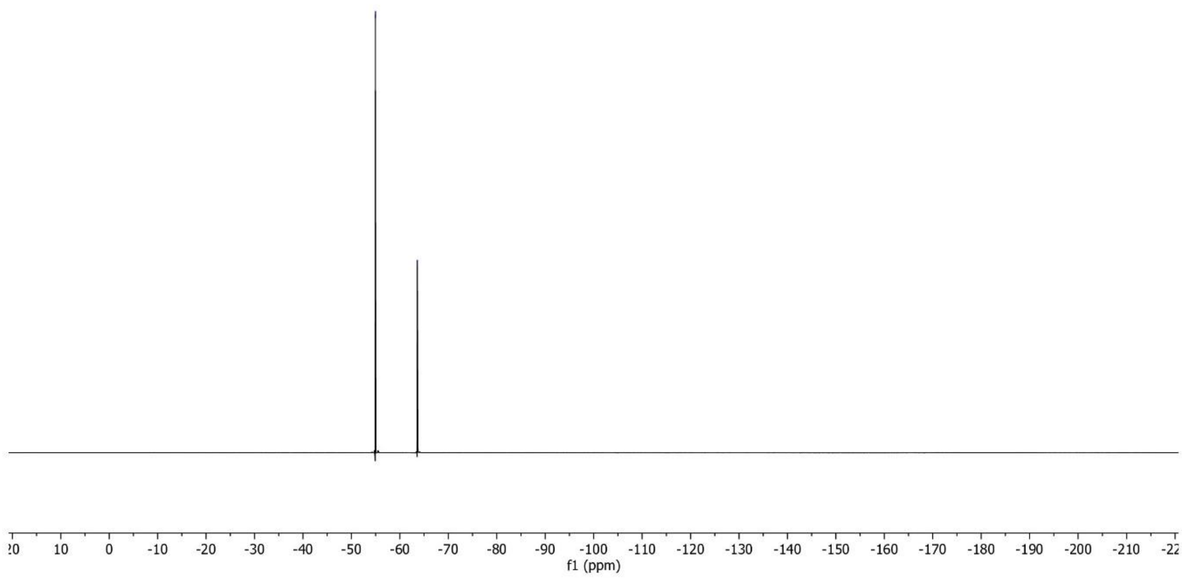

2-(5-(Triethylsilyl)hex-5-en-1-yl)isoindoline-1,3-dione (2g)

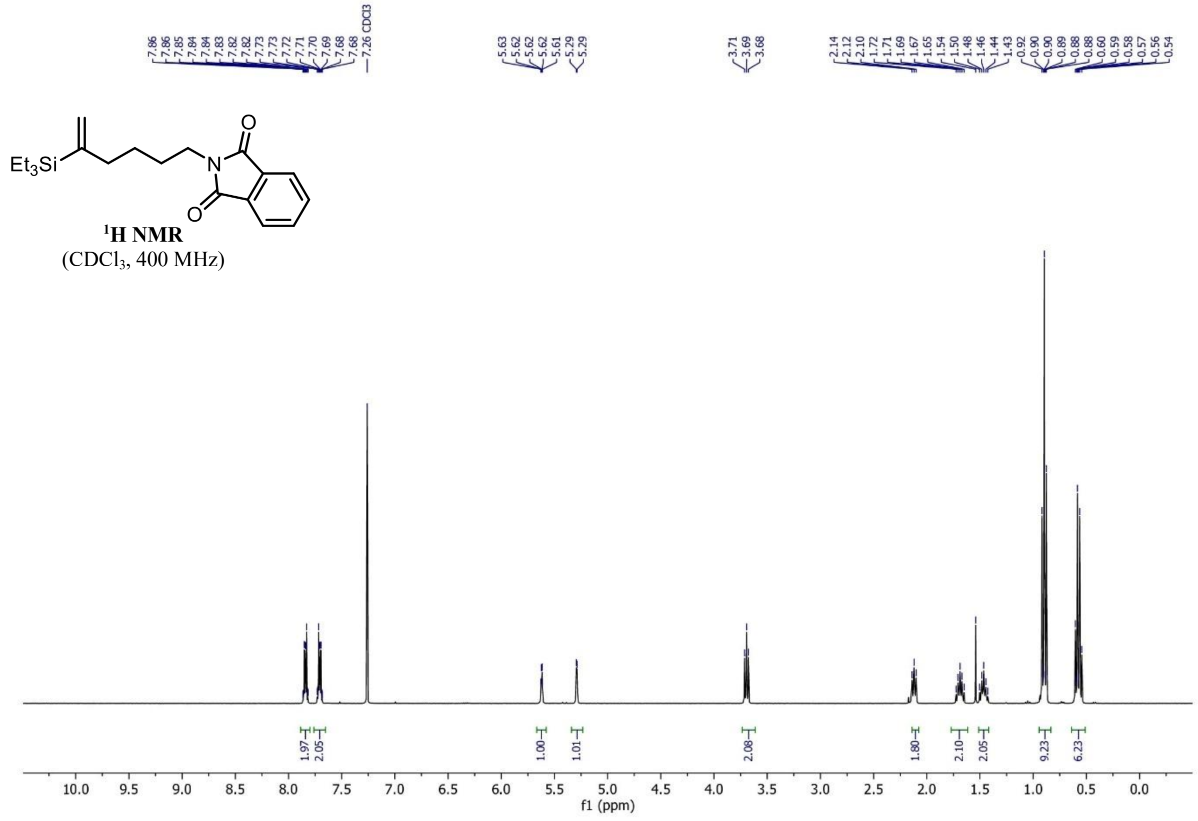

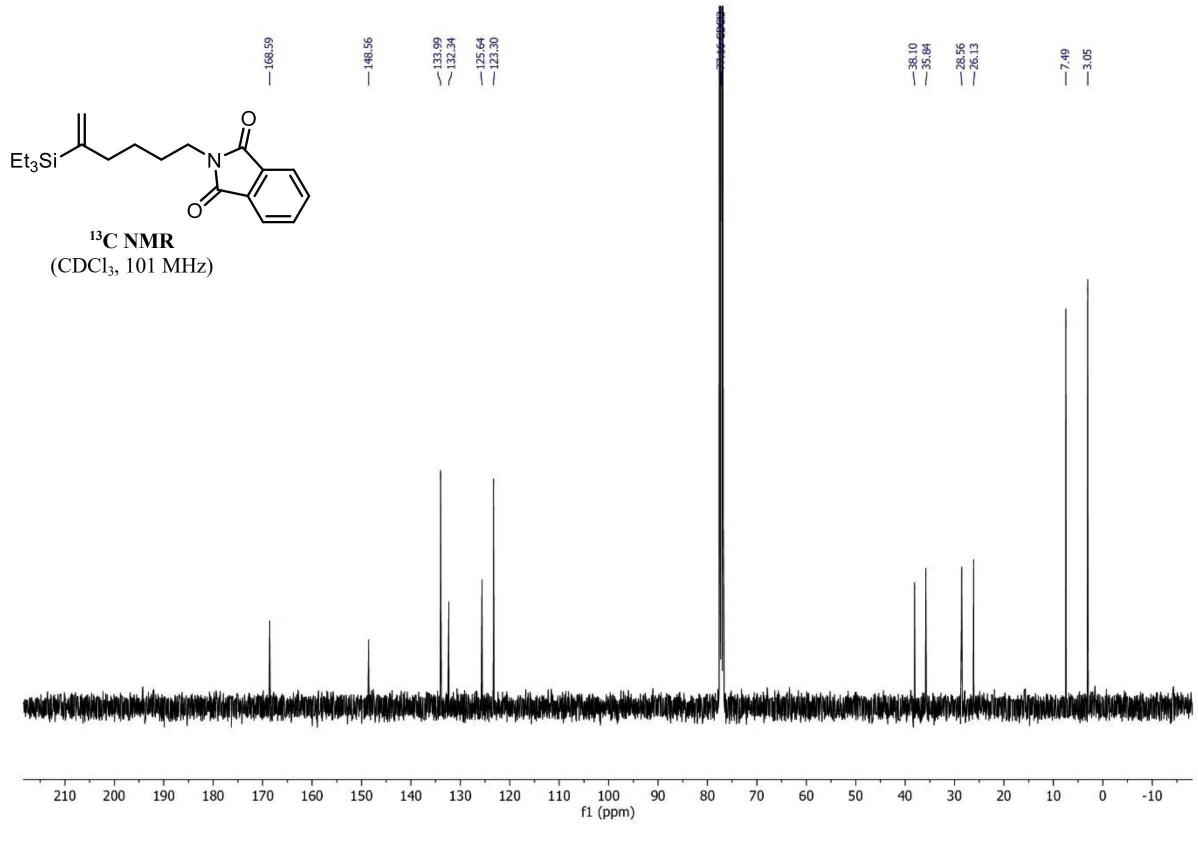

5-(Trihexylsilyl)hex-5-en-1-ol (2h)

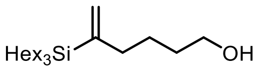

^{1}H NMR
($CDCl_3$, 400 MHz)

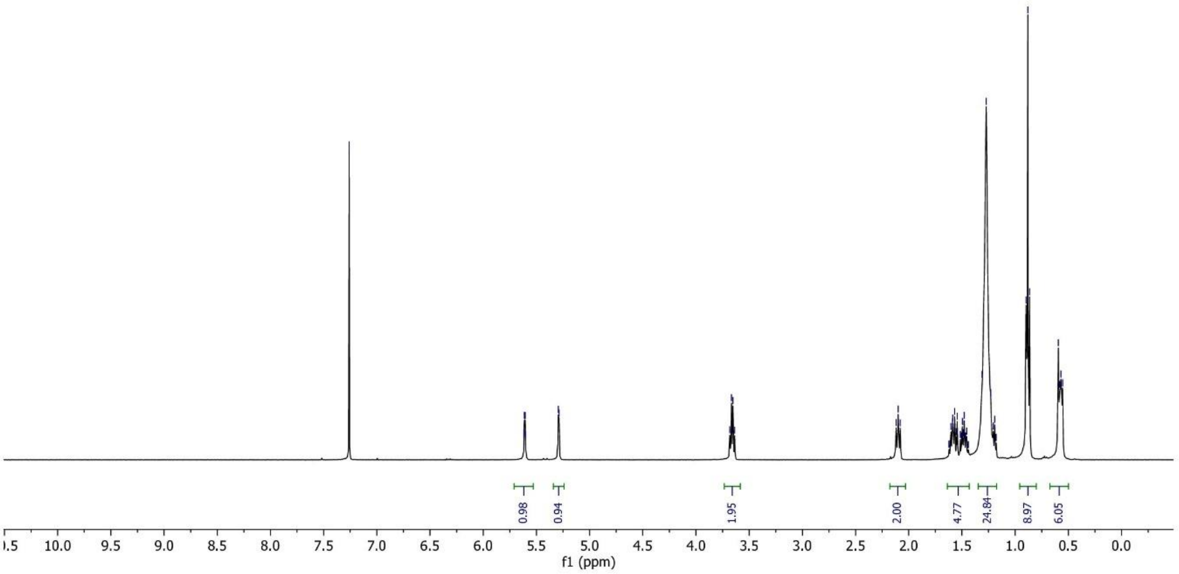

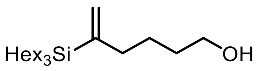

^{13}C NMR
($CDCl_3$, 101 MHz)

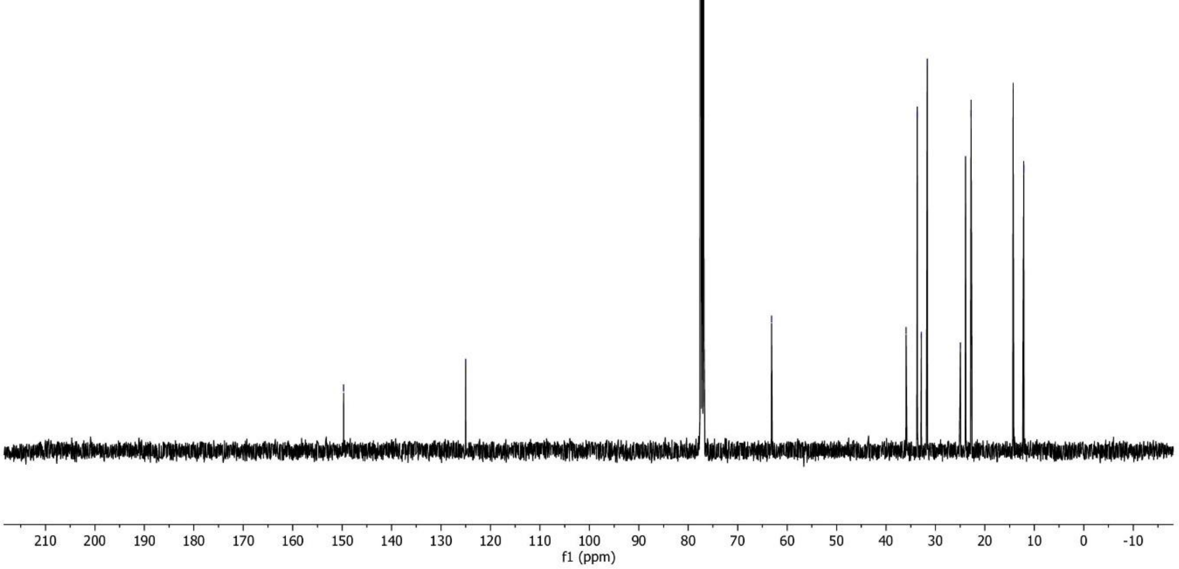

5-(Trihexylsilyl)hex-5-en-1-yl 2,2,2-trifluoroacetate (2i)

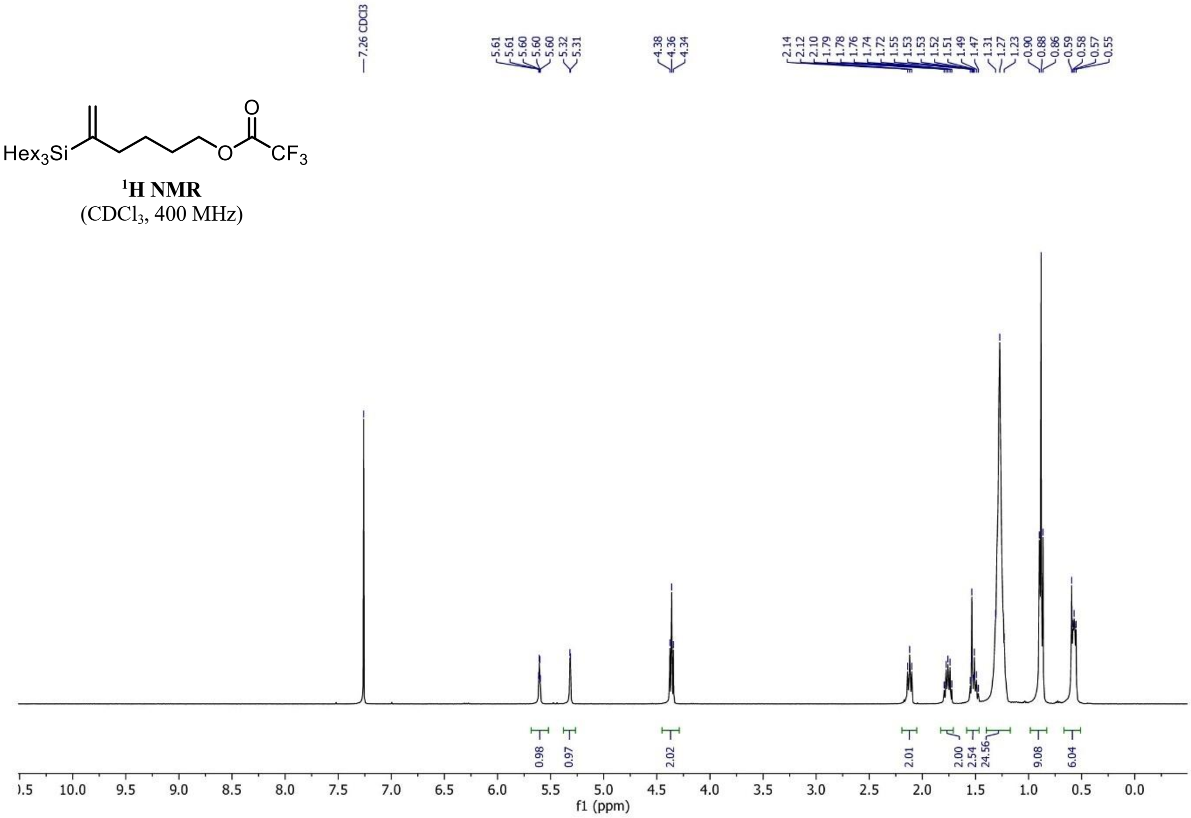

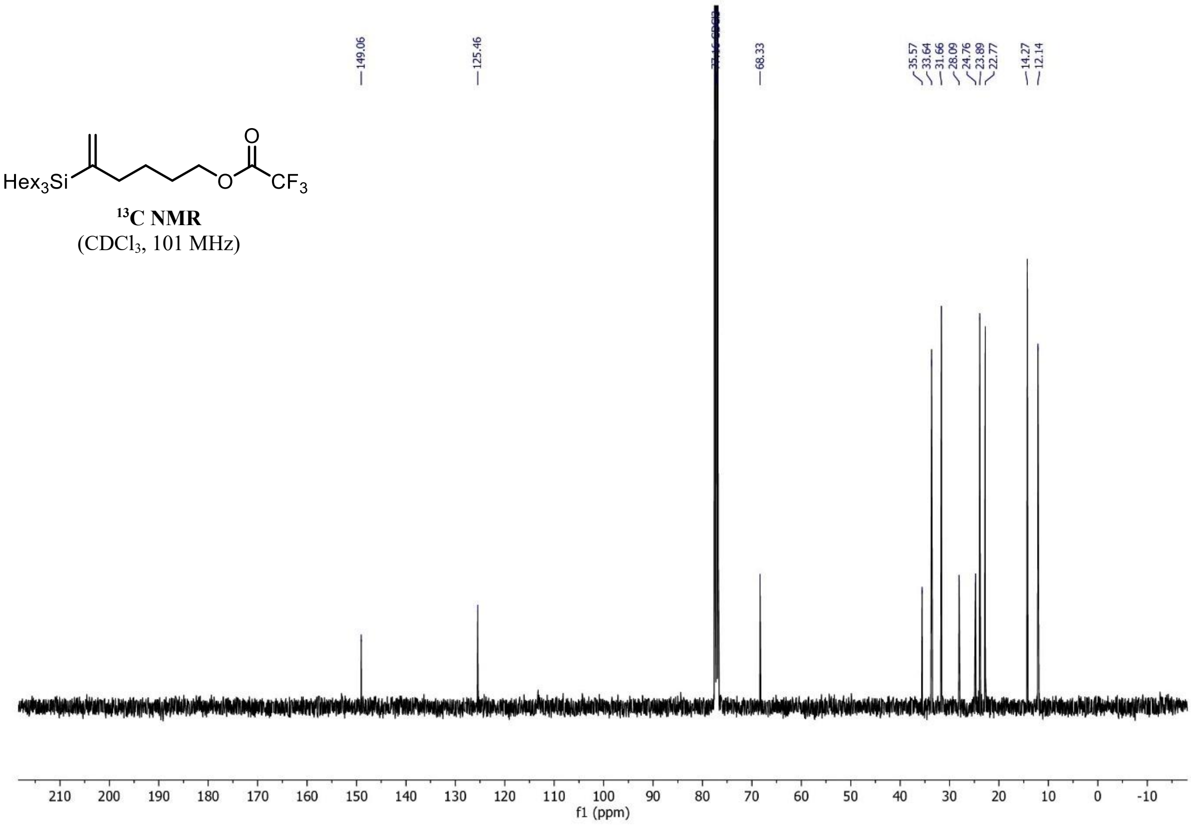

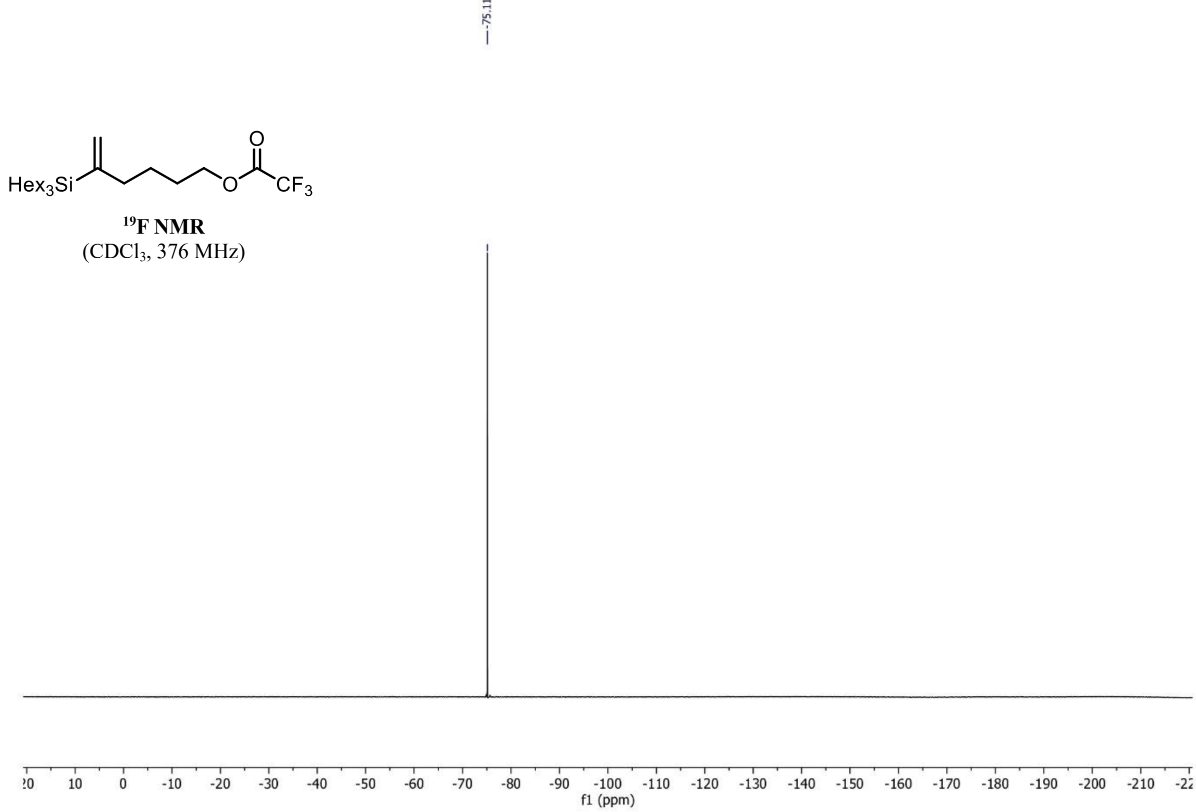

^{19}F NMR
($CDCl_3$, 376 MHz)

5-(Trihexylsilyl)hex-5-en-1-yl 4-methylbenzenesulfonate (2j)

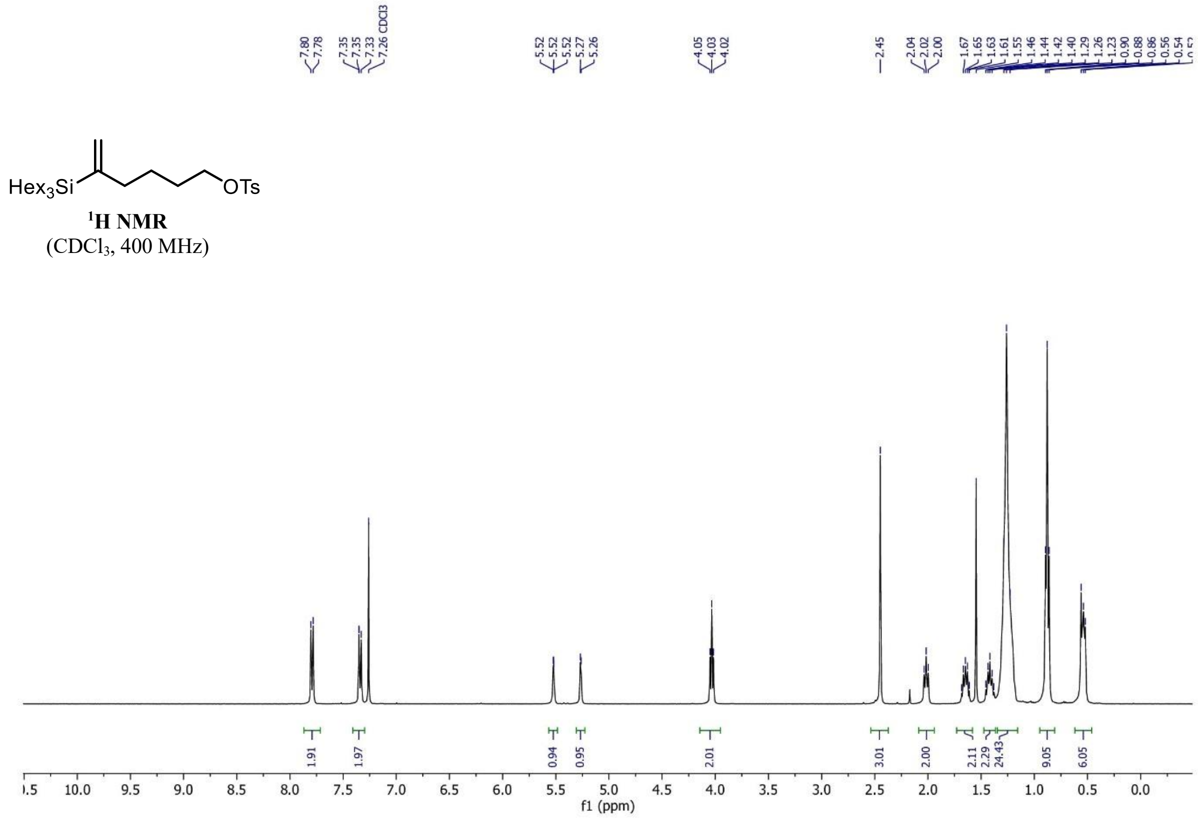

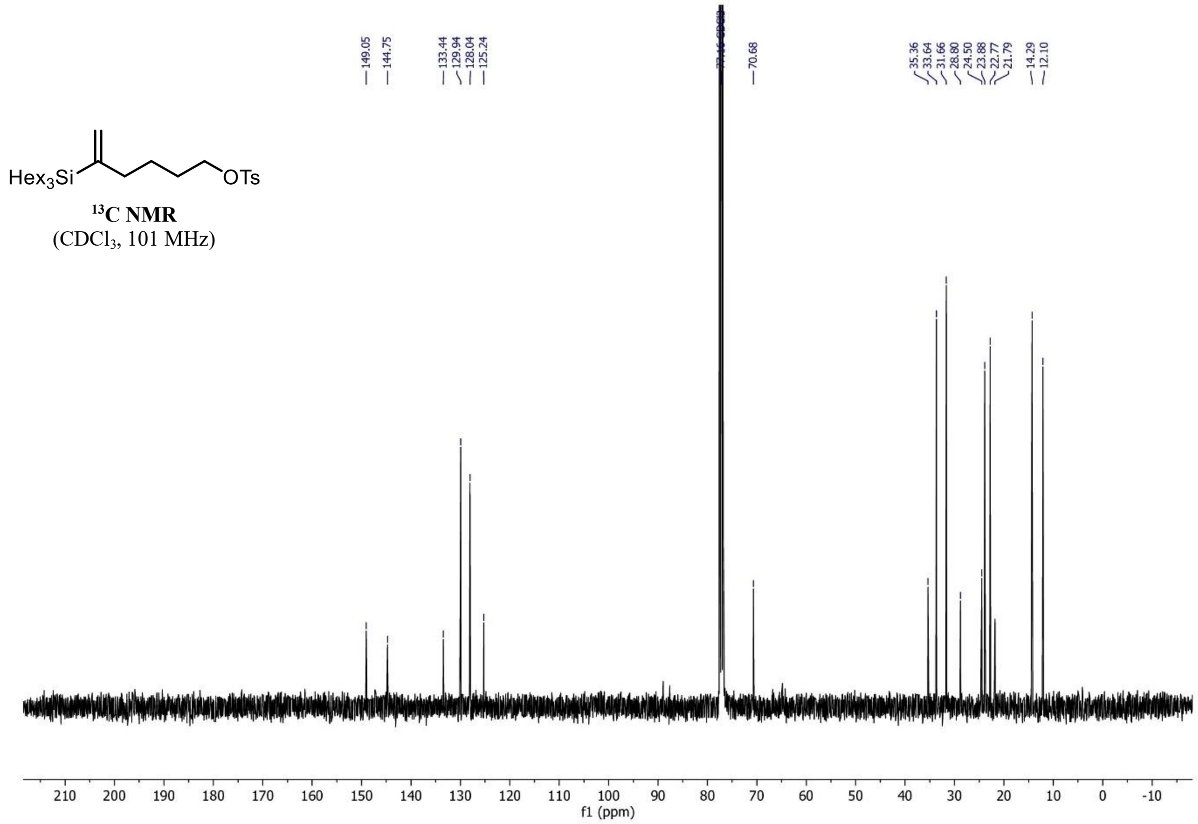

Dimethyl (5-(trihexylsilyl)hex-5-en-1-yl) phosphate (2k)

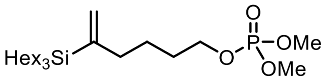

^{1}**H NMR**
($CDCl_3$, 400 MHz)

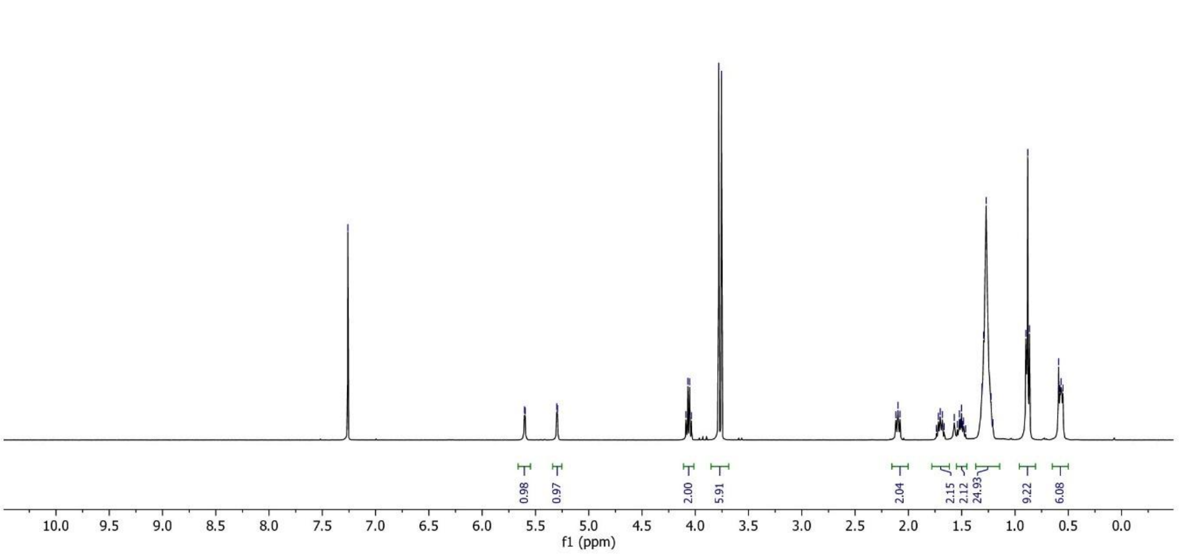

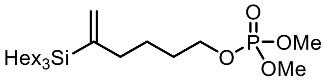

^{13}**C NMR**
($CDCl_3$, 101 MHz)

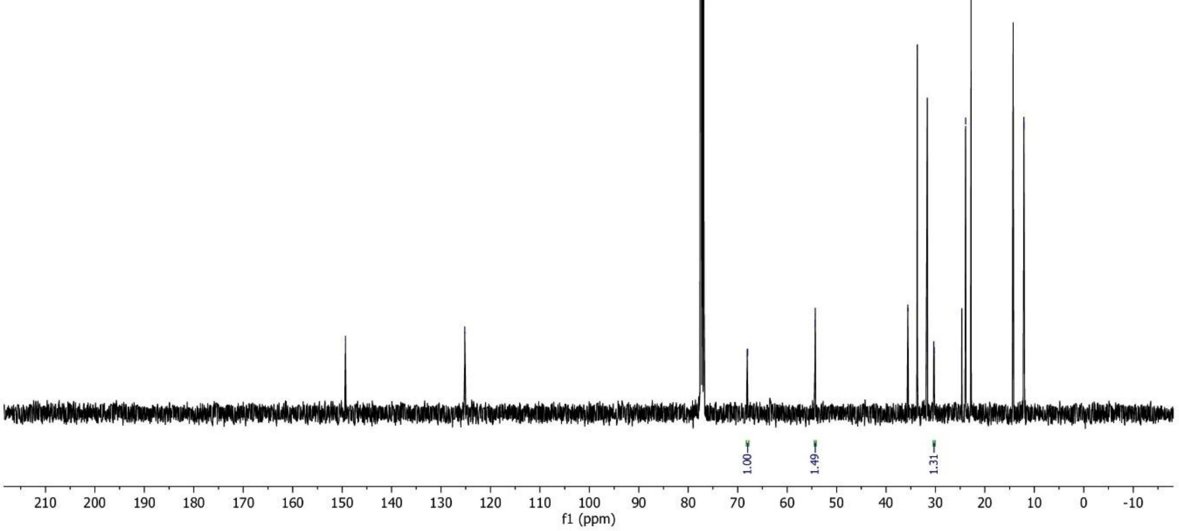

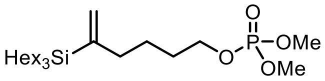

^{31}P NMR
($CDCl_3$, 162 MHz)

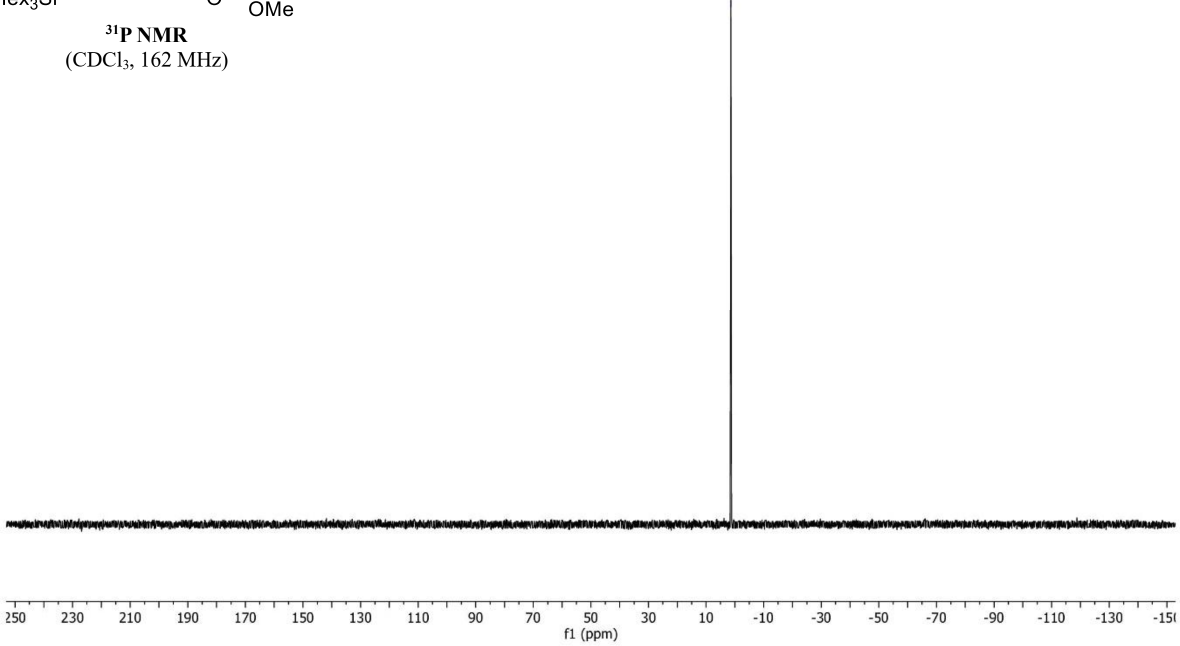

Methyl 4-((5-(trihexylsilyl)hex-5-en-1-yl)oxy)benzoate (2l)

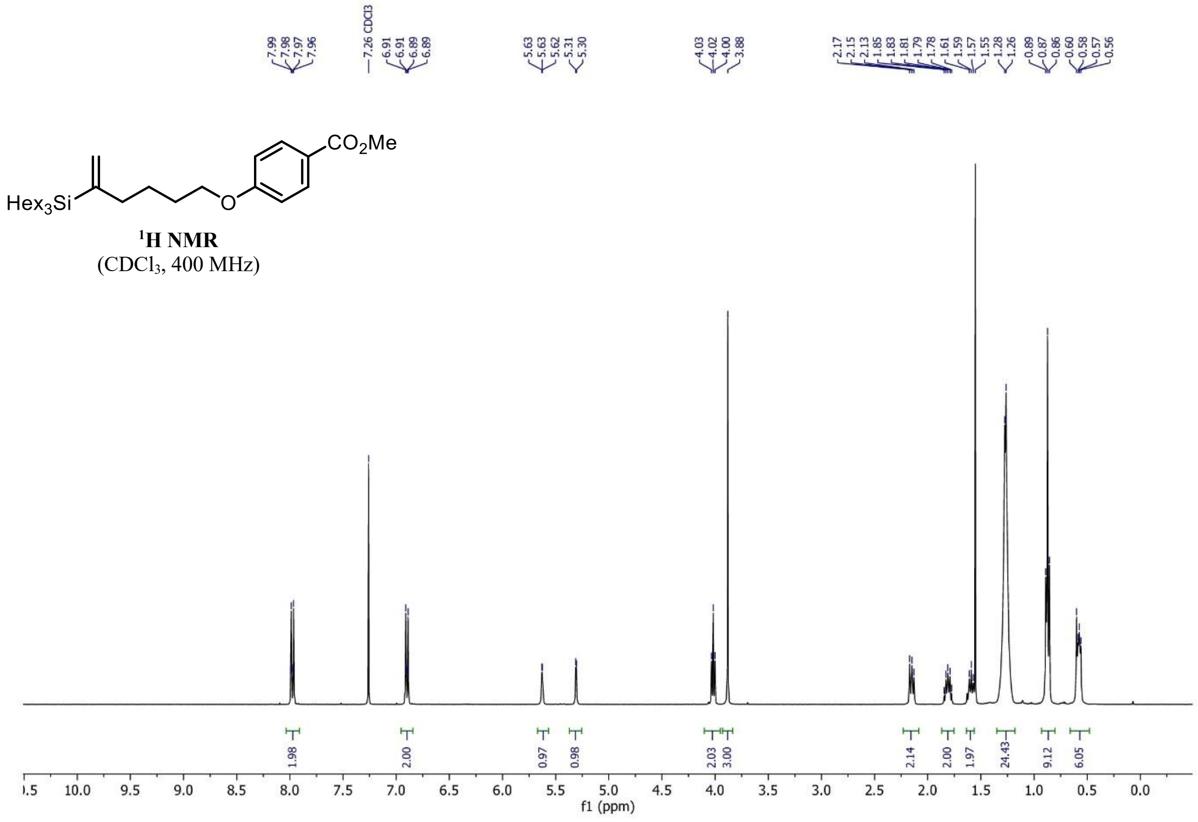

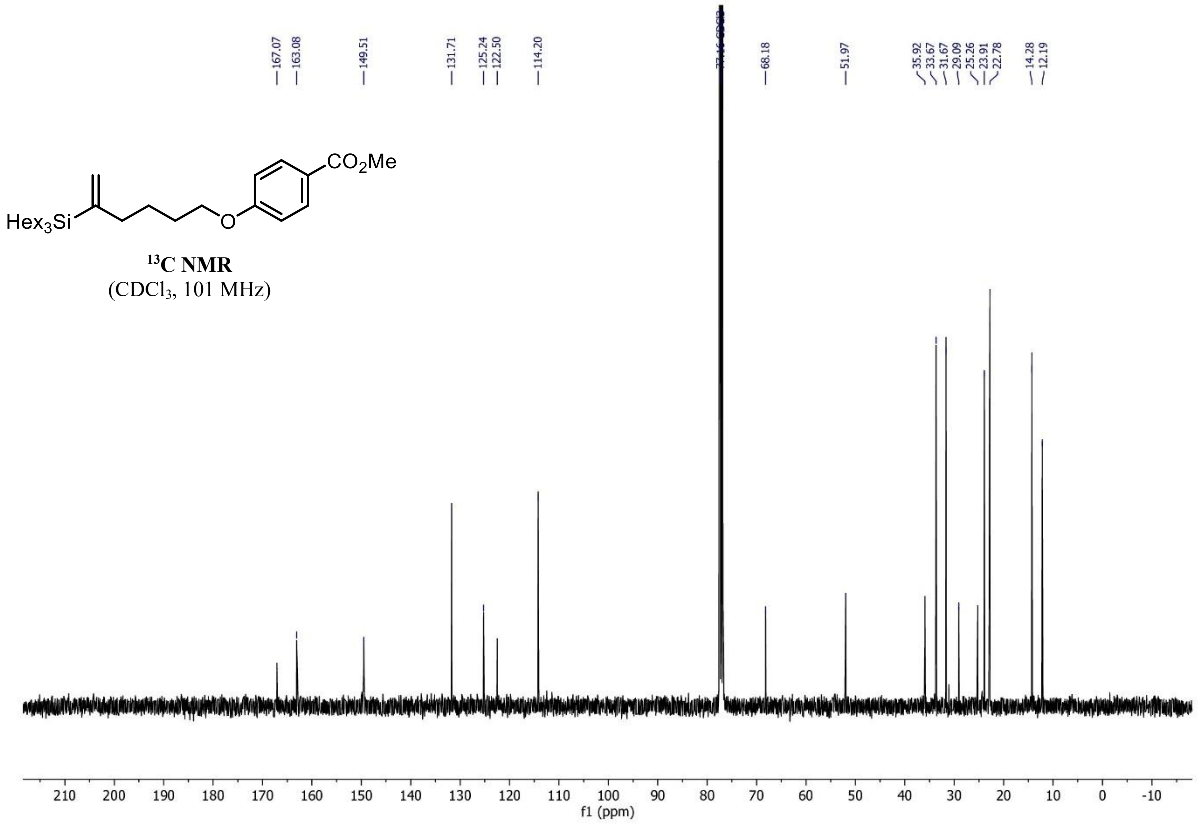

***N*,*N*-Dimethyl-10-(trihexylsilyl)undec-10-enamide (2m)**

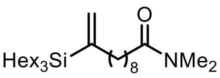

^{1}H NMR
($CDCl_3$, 400 MHz)

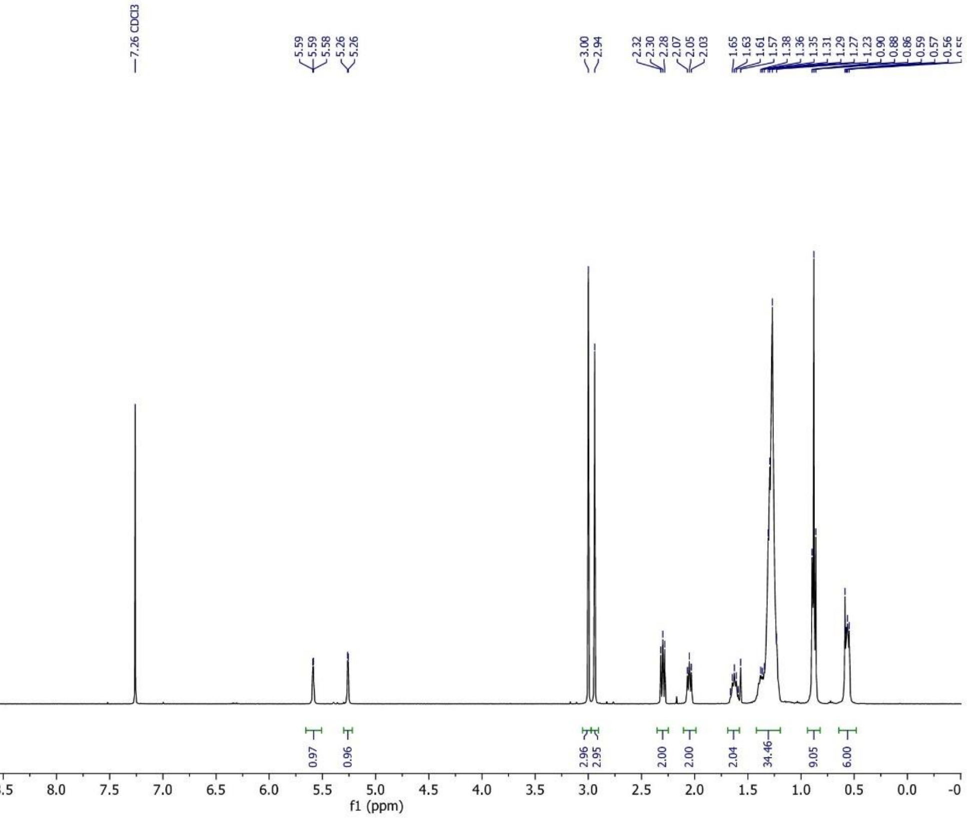

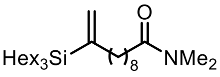

^{13}C NMR
($CDCl_3$, 101 MHz)

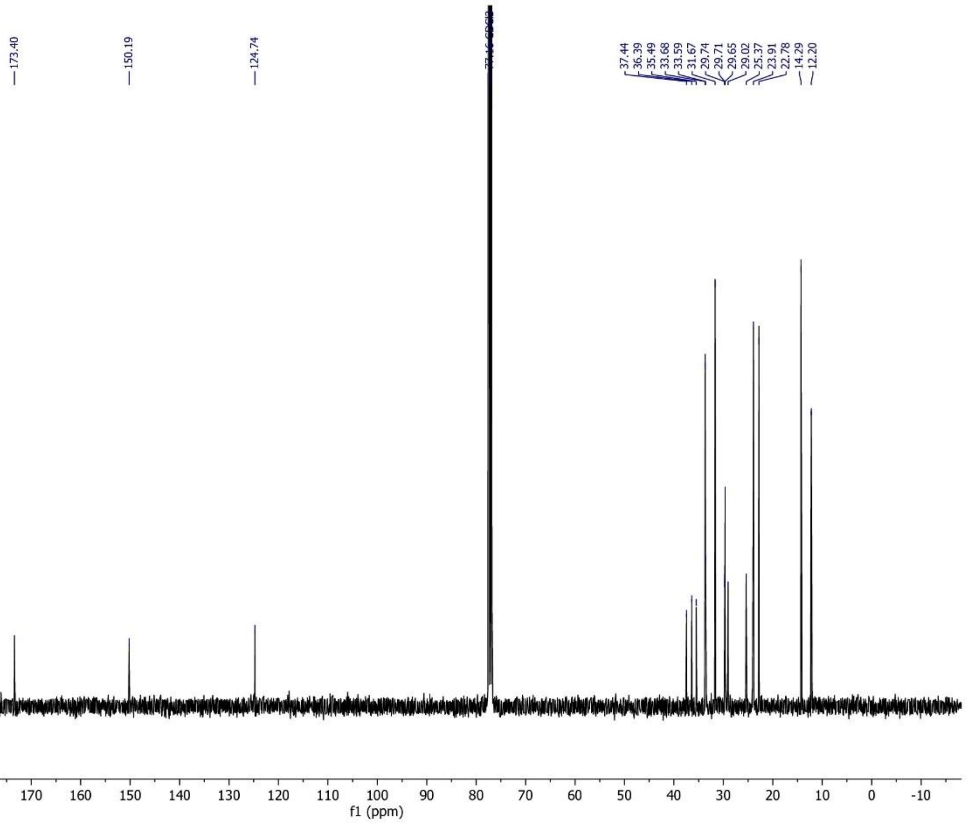

Ethyl (*E*)-12-(triethylsilyl)trideca-2,12-dienoate (2n)

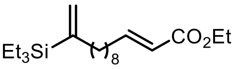

^{1}H NMR
($CDCl_3$, 400 MHz)

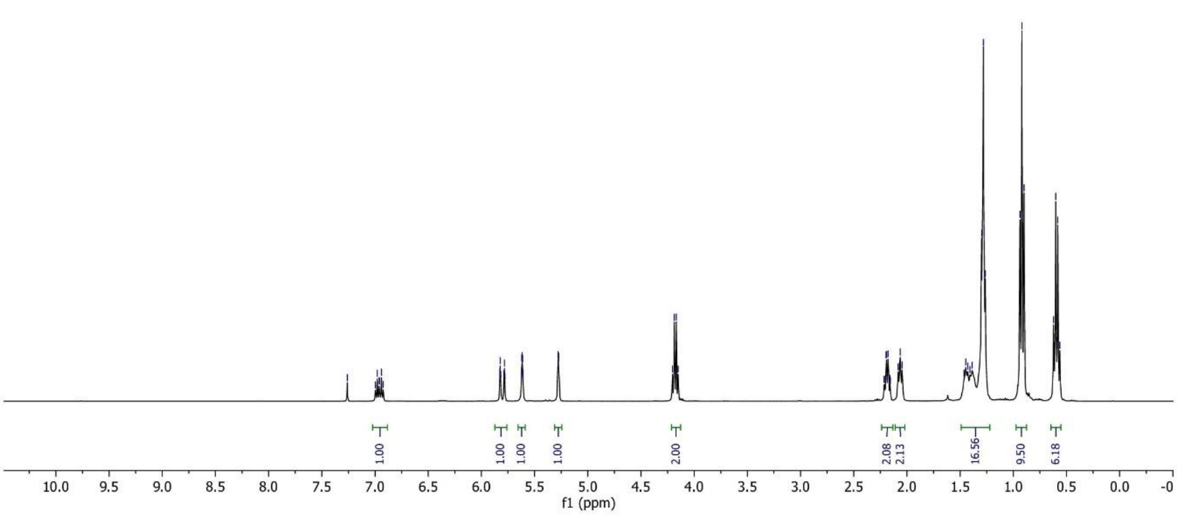

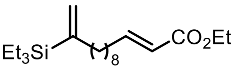

^{13}C NMR
($CDCl_3$, 101 MHz)

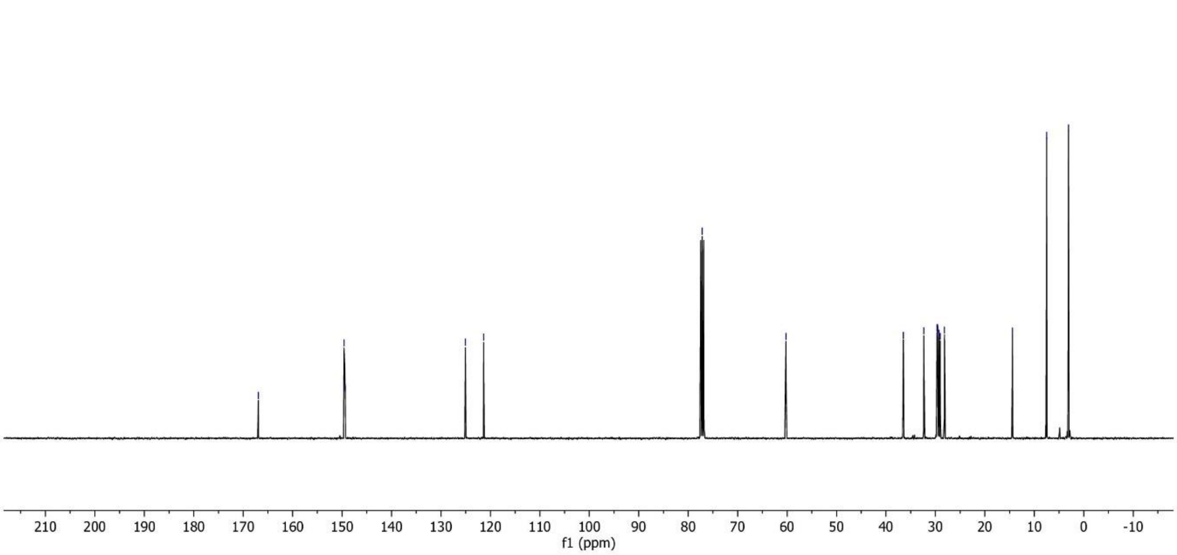

(*S*)-1-(4-Fluorophenyl)-3-((2*S*,3*R*)-1-(4-fluorophenyl)-2-(4-methoxyphenyl)-4-oxoazetidin-3-yl)propyl 10-(trihexylsilyl)undec-10-enoate (2o)

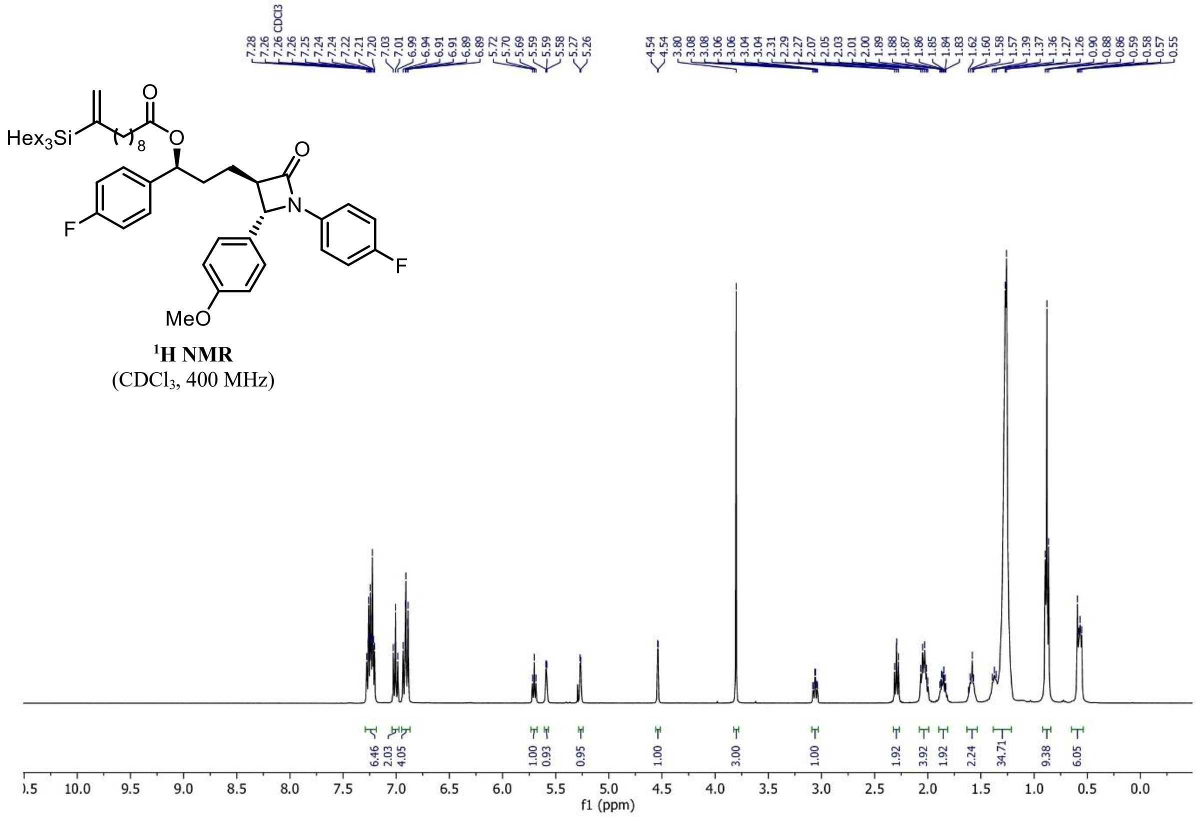

^{1}H NMR
($CDCl_3$, 400 MHz)

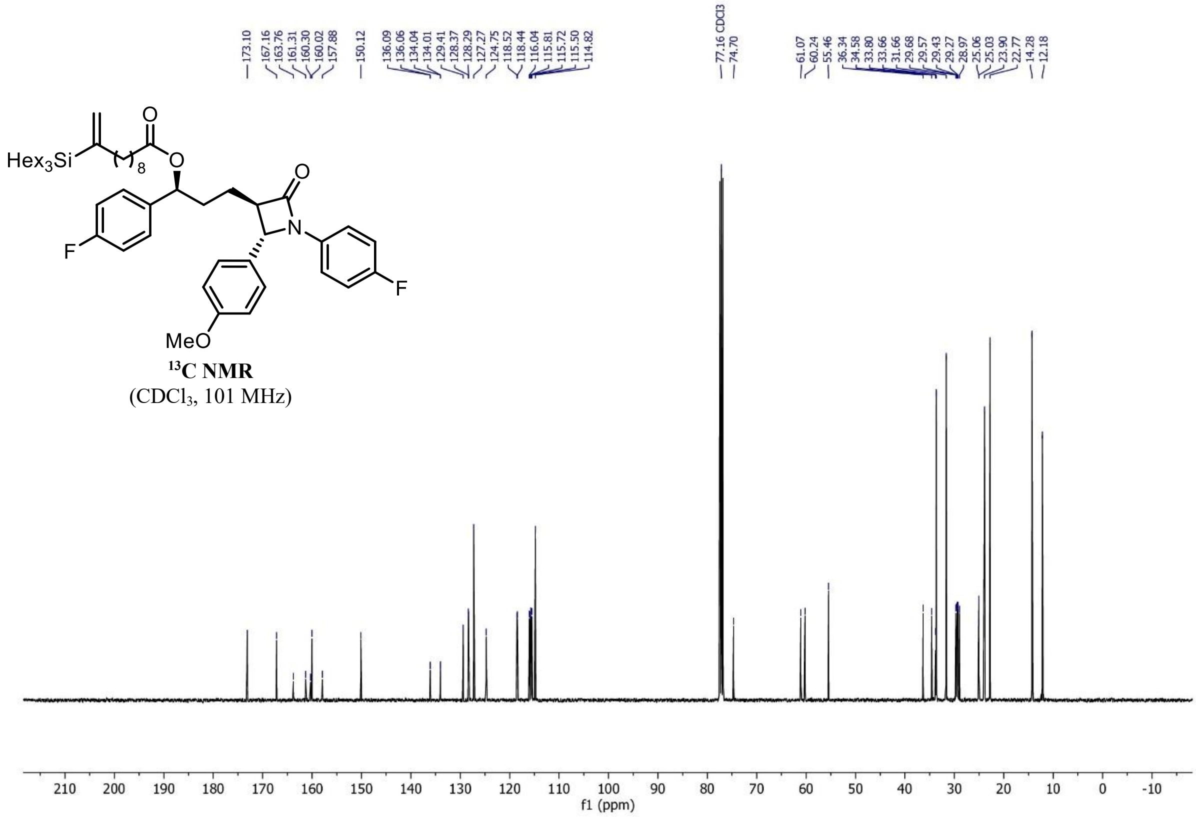

^{13}C NMR
($CDCl_3$, 101 MHz)

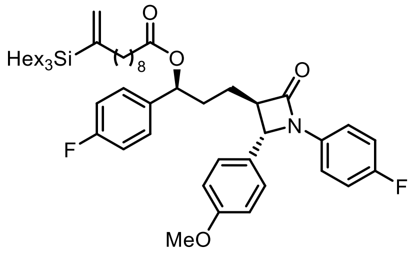

^{19}F NMR
($CDCl_3$, 376 MHz)

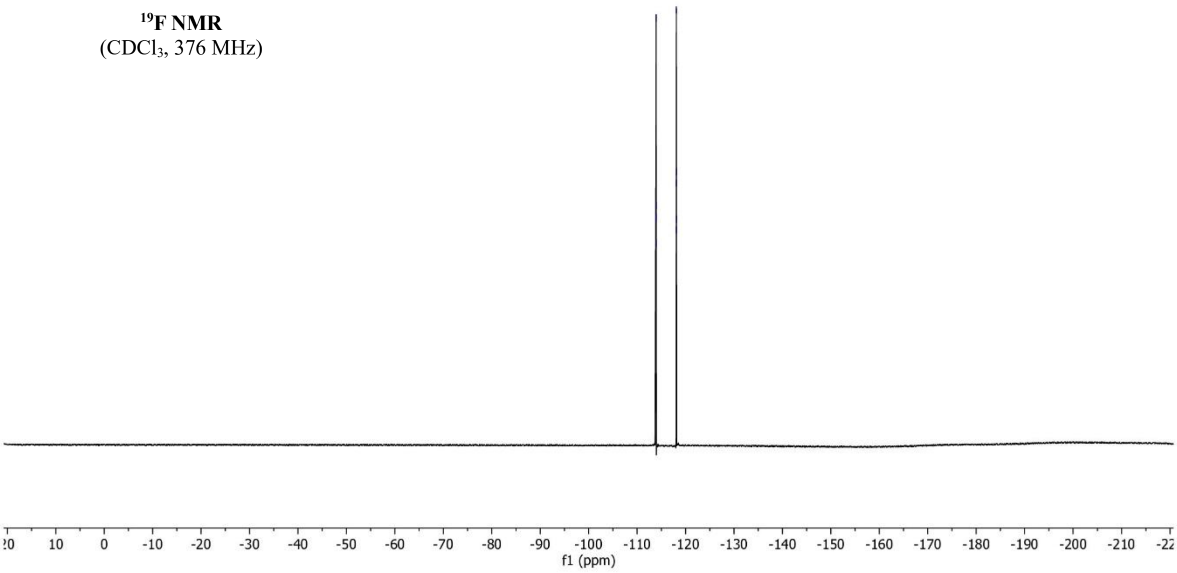

(*E*)-Dec-5-en-5-yltrihexylsilane (2p)

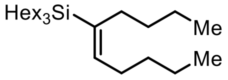

^{1}H NMR
($CDCl_3$, 600 MHz)

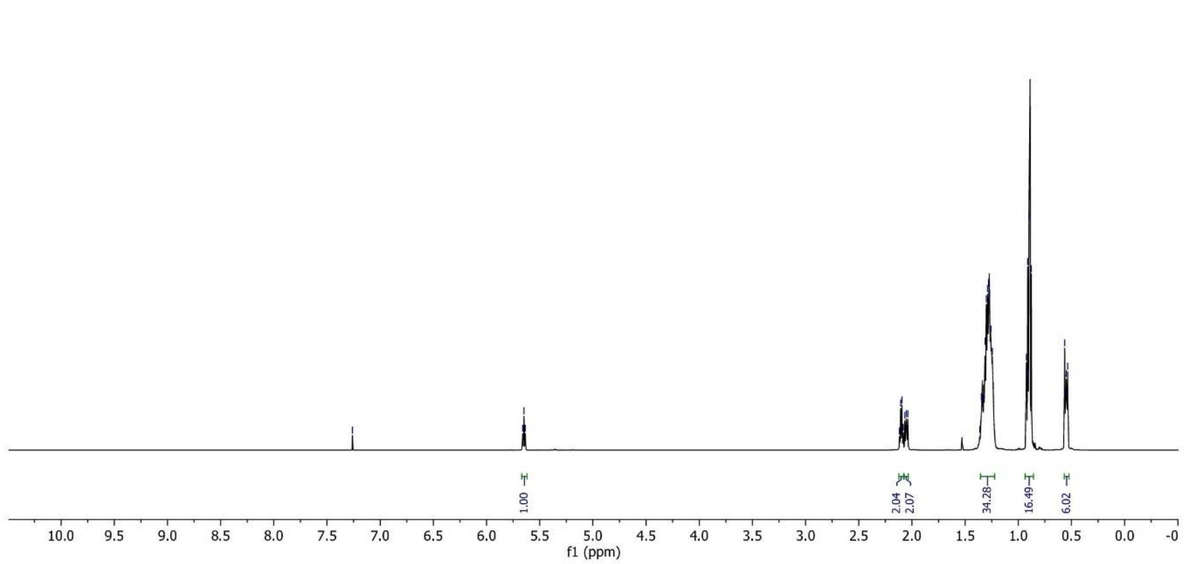

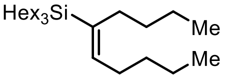

^{13}C NMR
($CDCl_3$, 101 MHz)

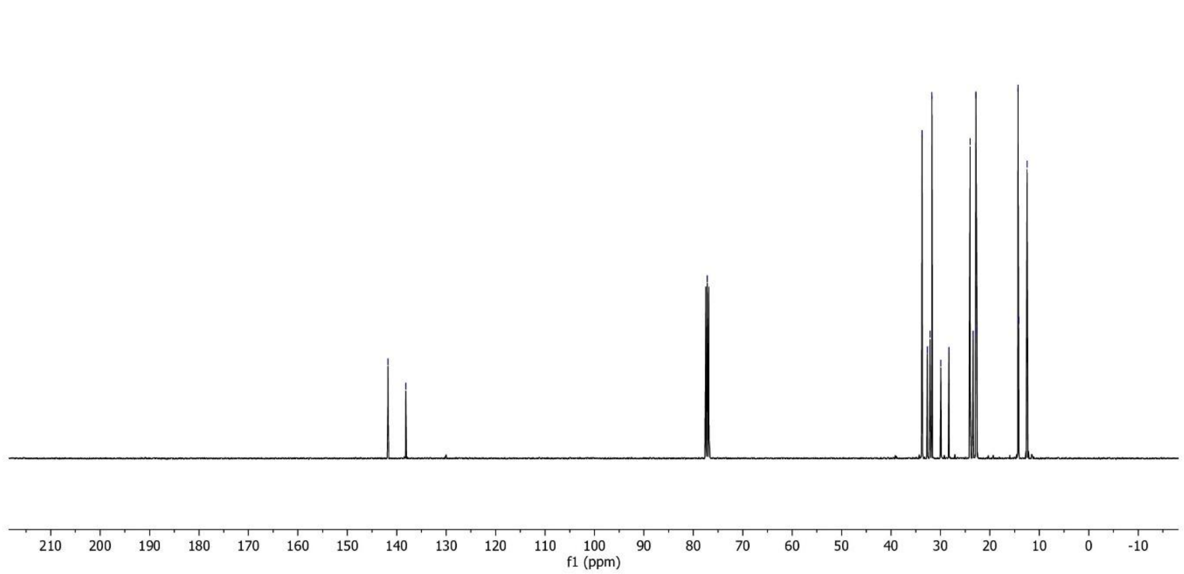

10-(Triethylsilyl)undec-10-enoic acid (S8)

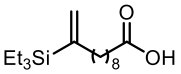

^{1}H NMR
($CDCl_3$, 400 MHz)

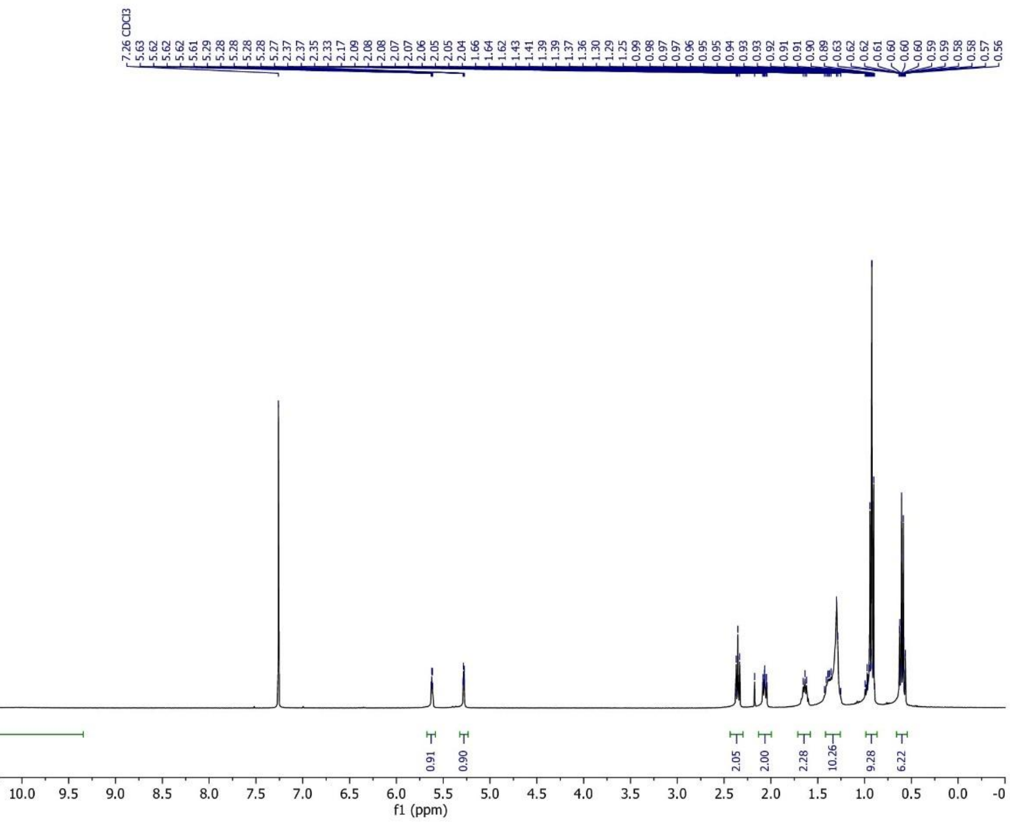

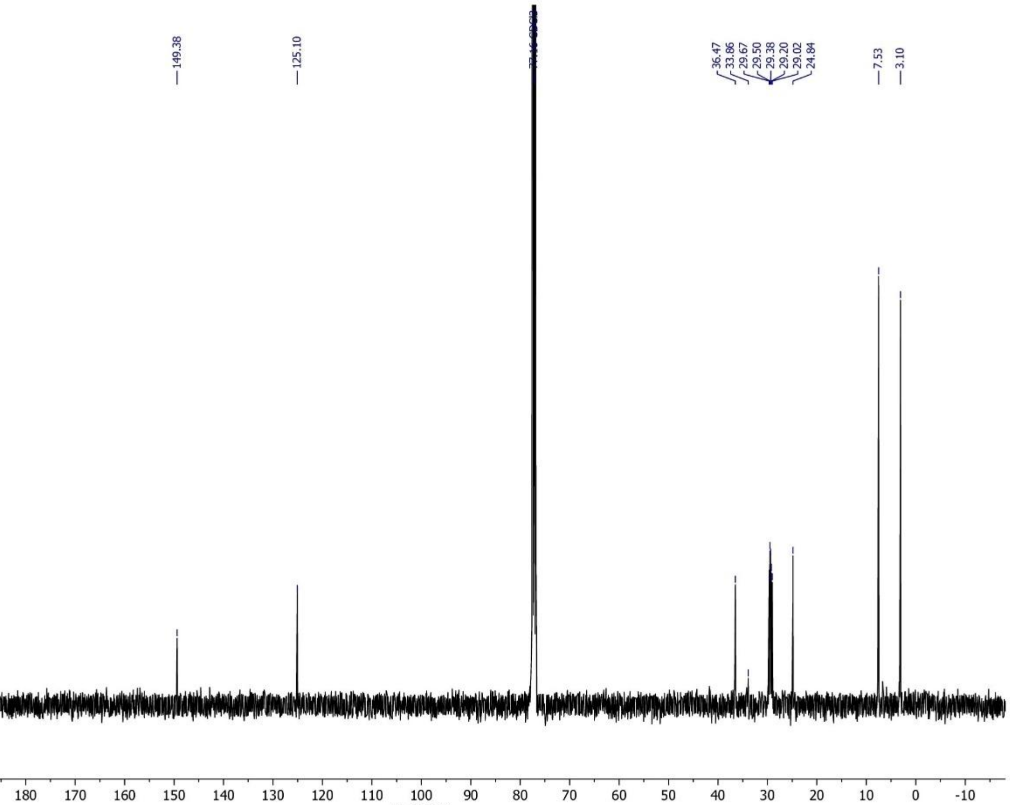

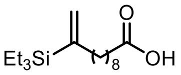

^{13}C NMR
($CDCl_3$, 101 MHz)

Methyl 10-(triethylsilyl)undec-10-enoate (2q)

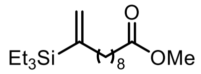

^{1}H NMR
($CDCl_3$, 400 MHz)

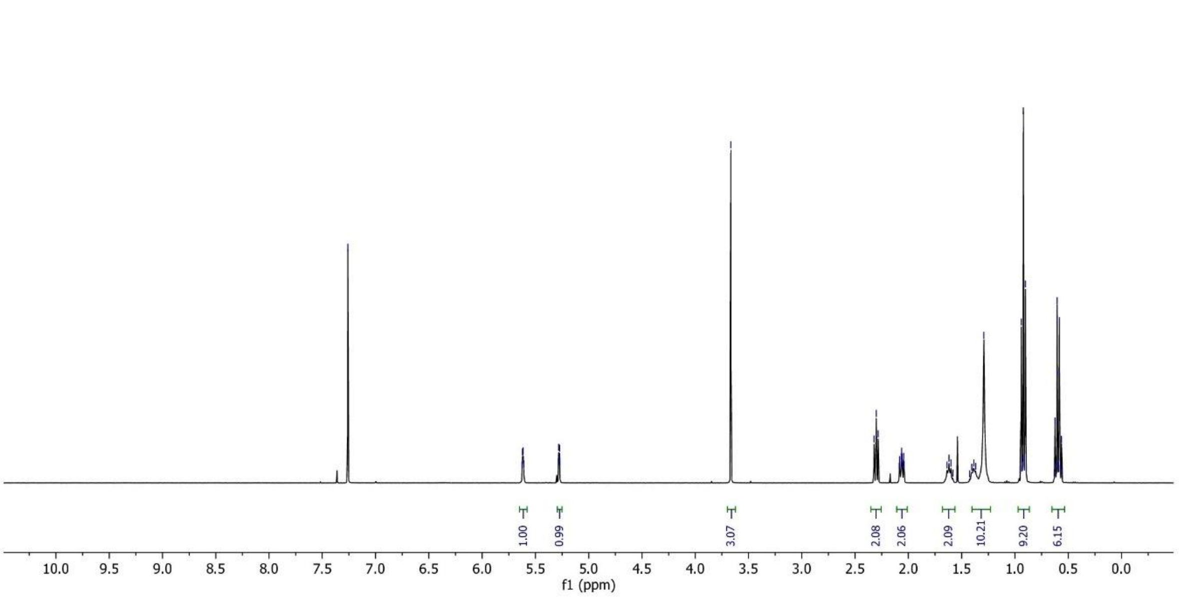

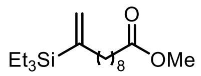

^{13}C NMR
($CDCl_3$, 101 MHz)

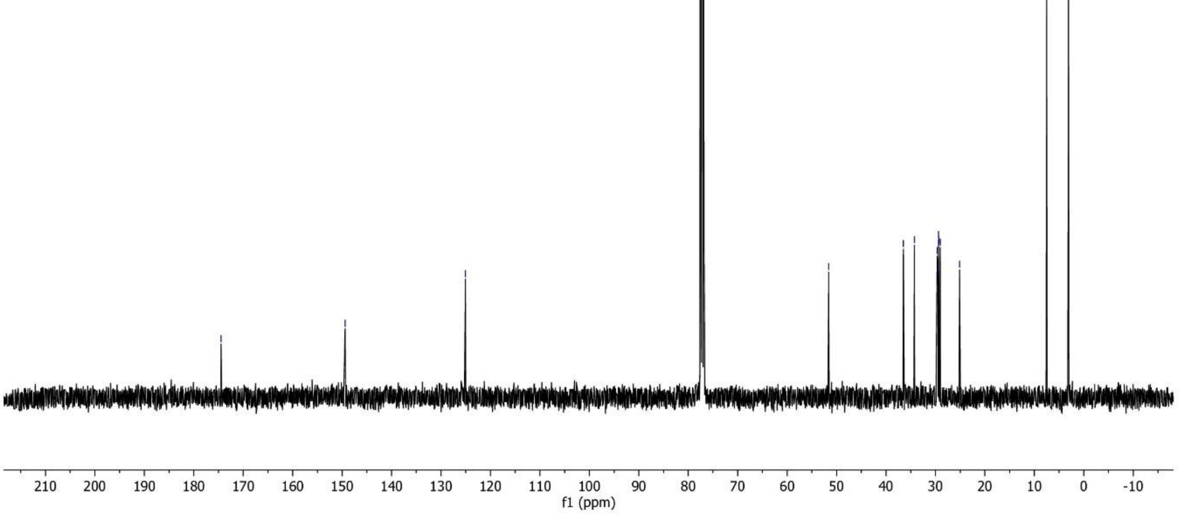

Methyl 12-(triethylsilyl)tridec-12-enoate (2r)

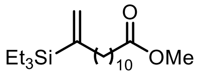

^{1}H NMR
($CDCl_3$, 400 MHz)

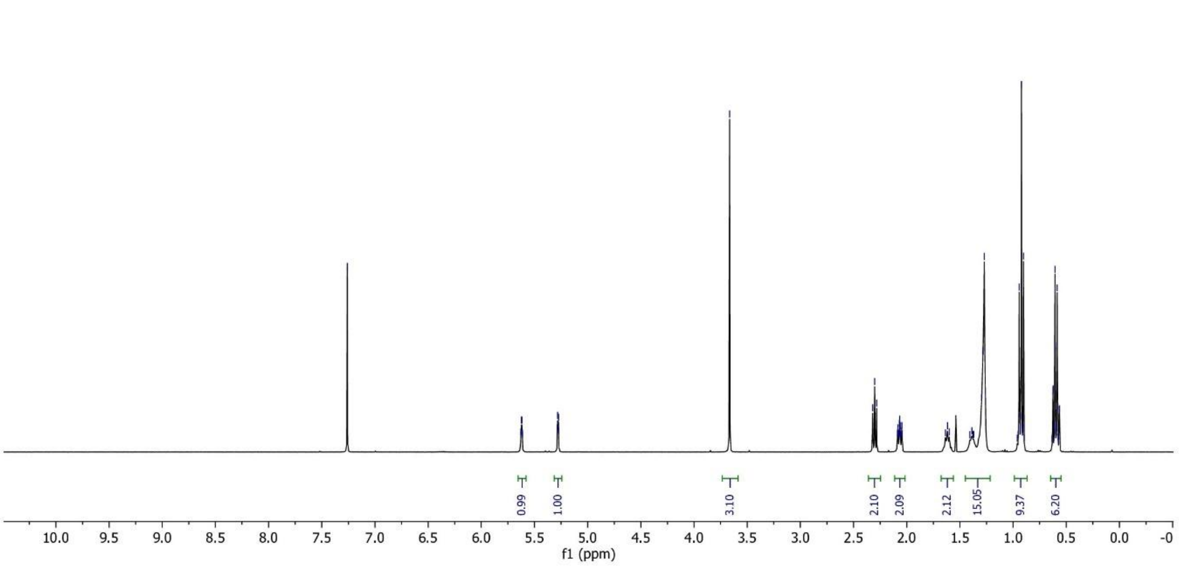

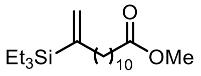

^{13}C NMR
($CDCl_3$, 101 MHz)

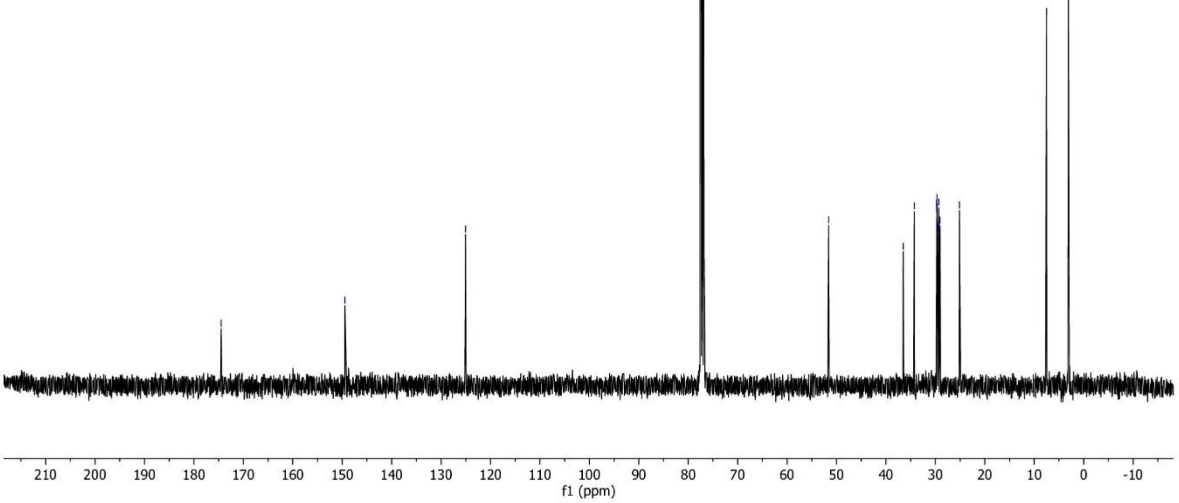

(*Z*)-1-Phenylnon-3-en-1-one (3)

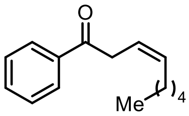

^{1}H NMR
($CDCl_3$, 600 MHz)

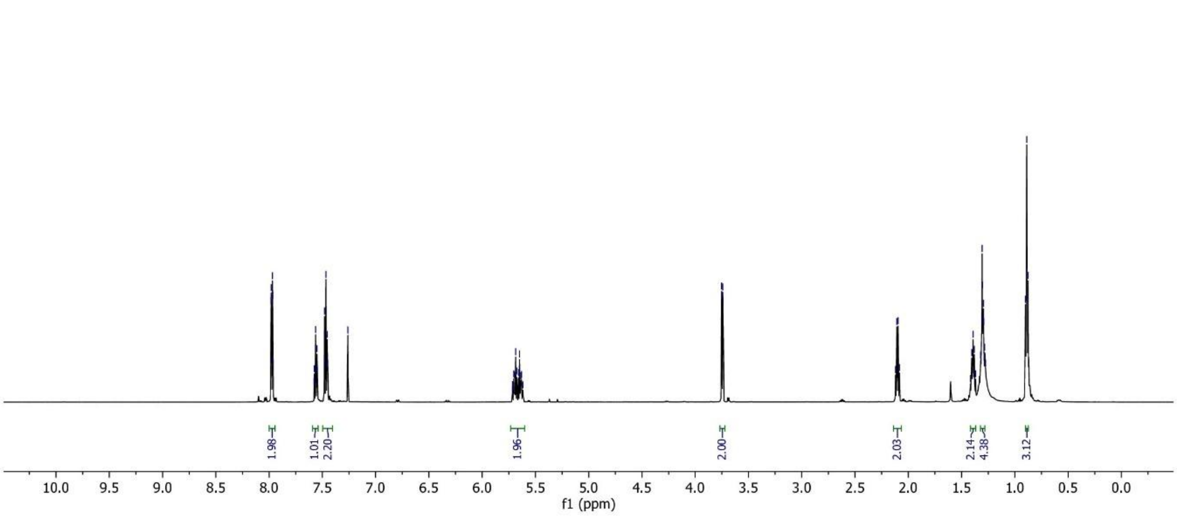

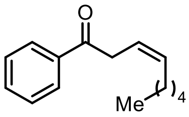

^{13}C NMR
($CDCl_3$, 151 MHz)

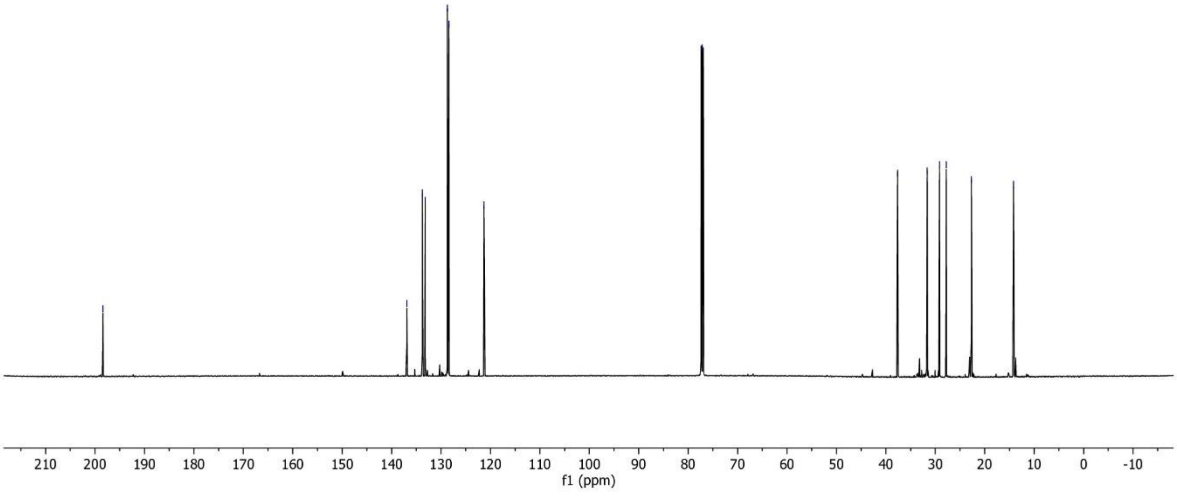

(*Z*)-1,5-Diphenylpent-3-en-1-one (4a)

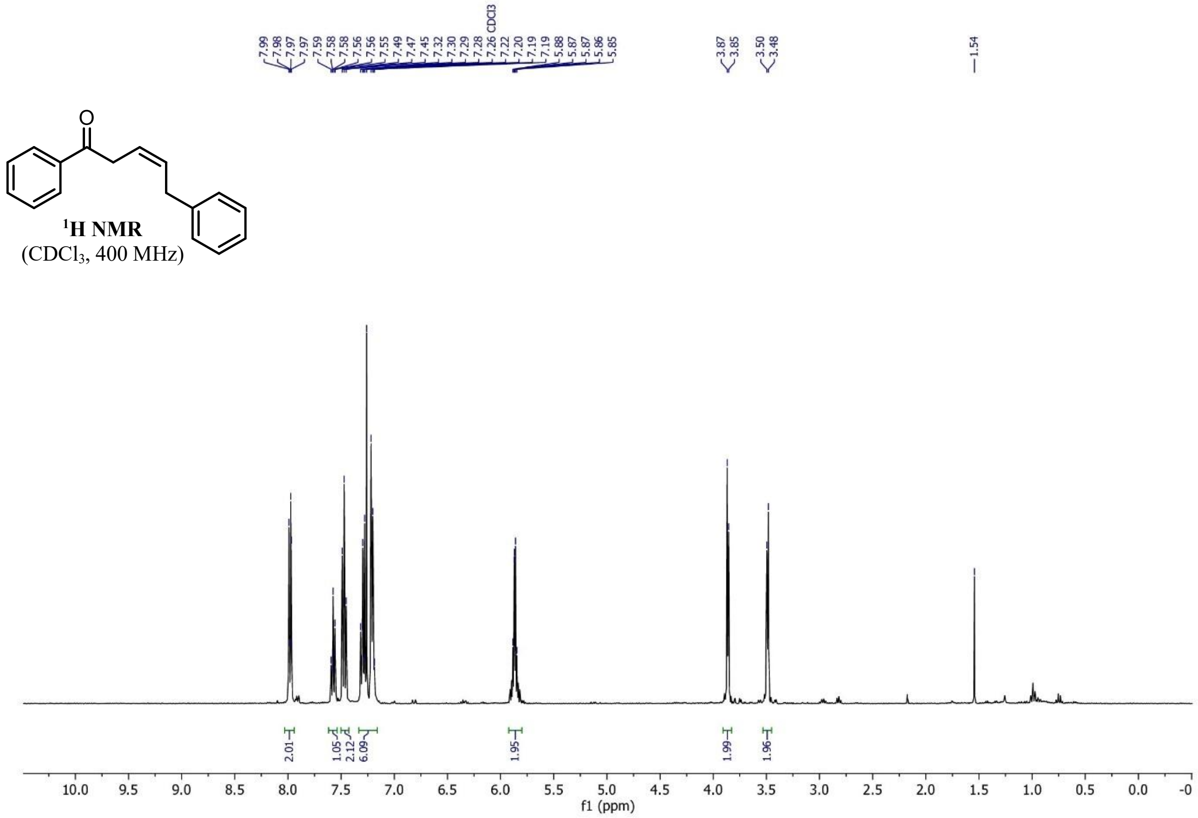

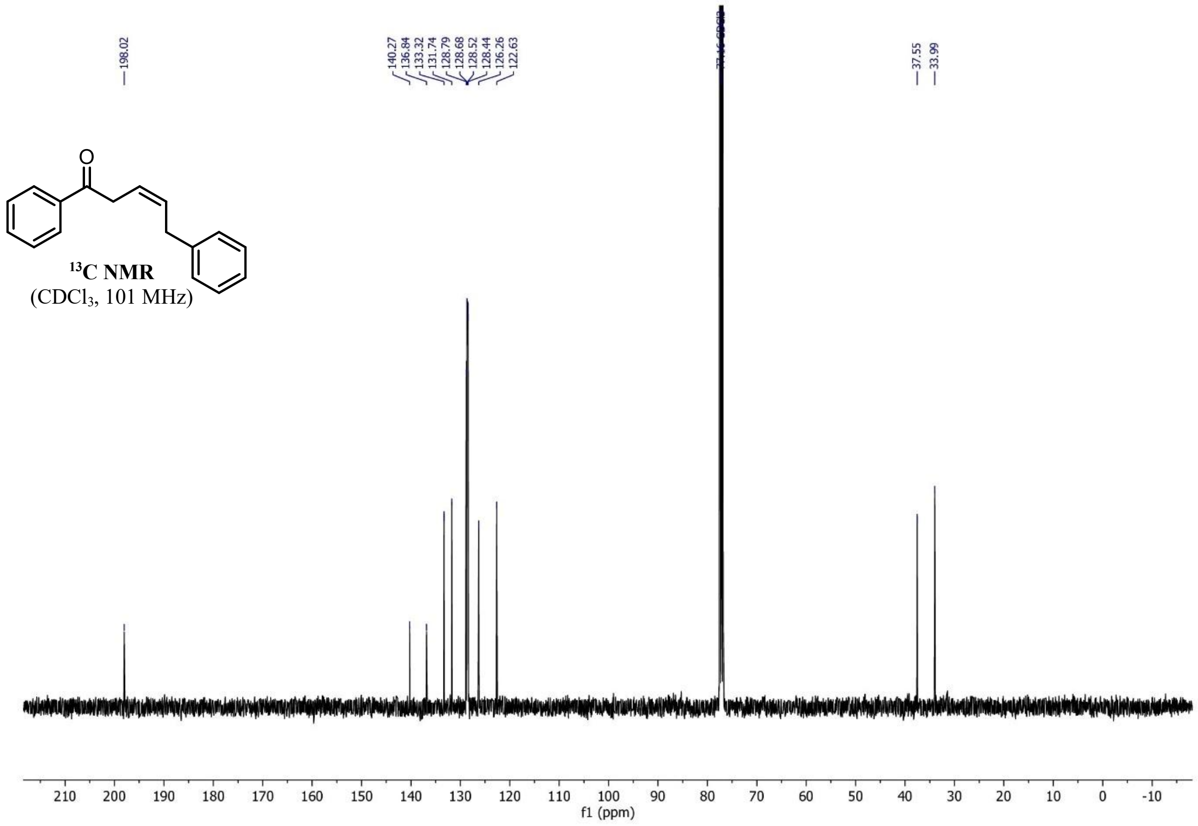

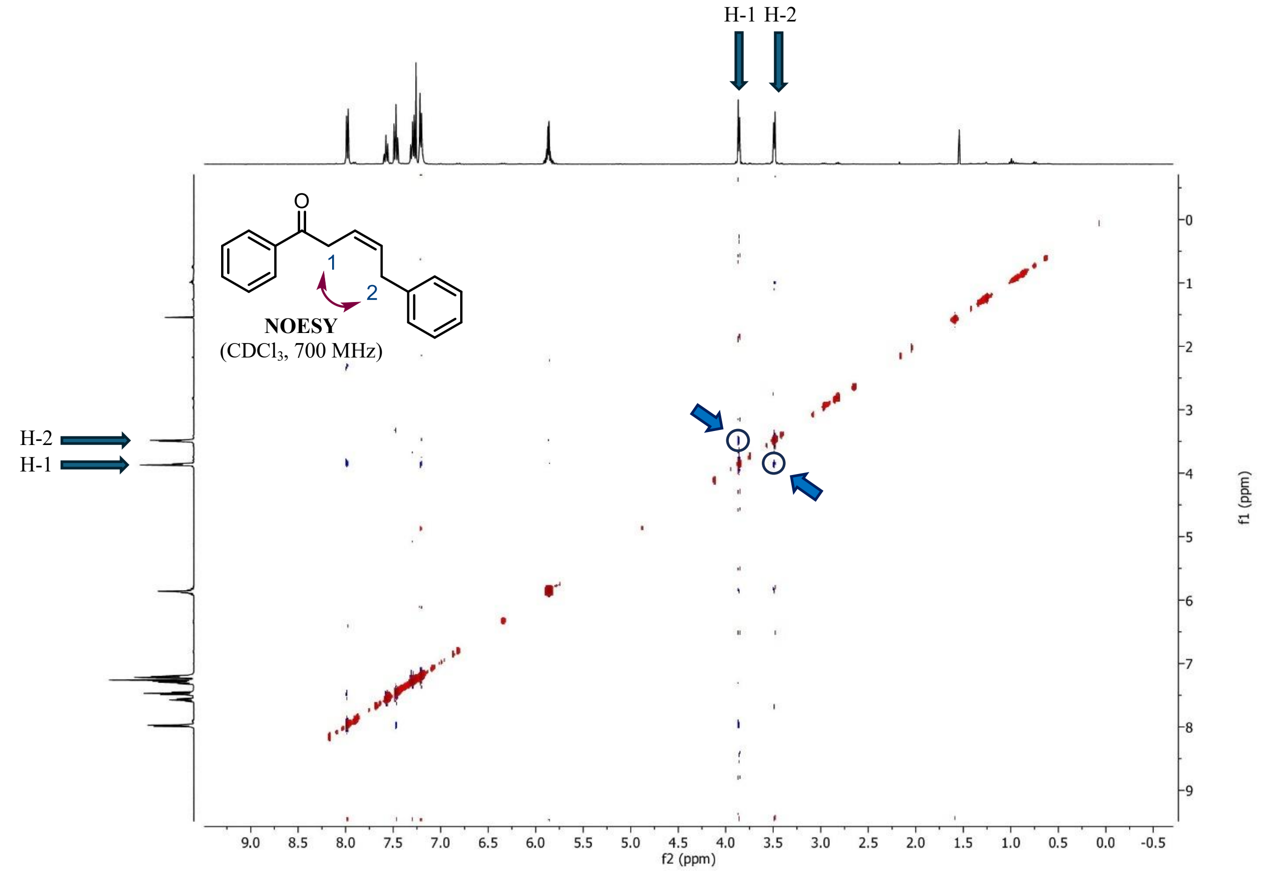
H-1 H-2
H-2
H-1
O
1
2
NOESY
(CDCl3, 700 MHz)
f1 (ppm)
f2 (ppm)

(*Z*)-1-(4-Fluorophenyl)-5-phenylpent-3-en-1-one (4b)

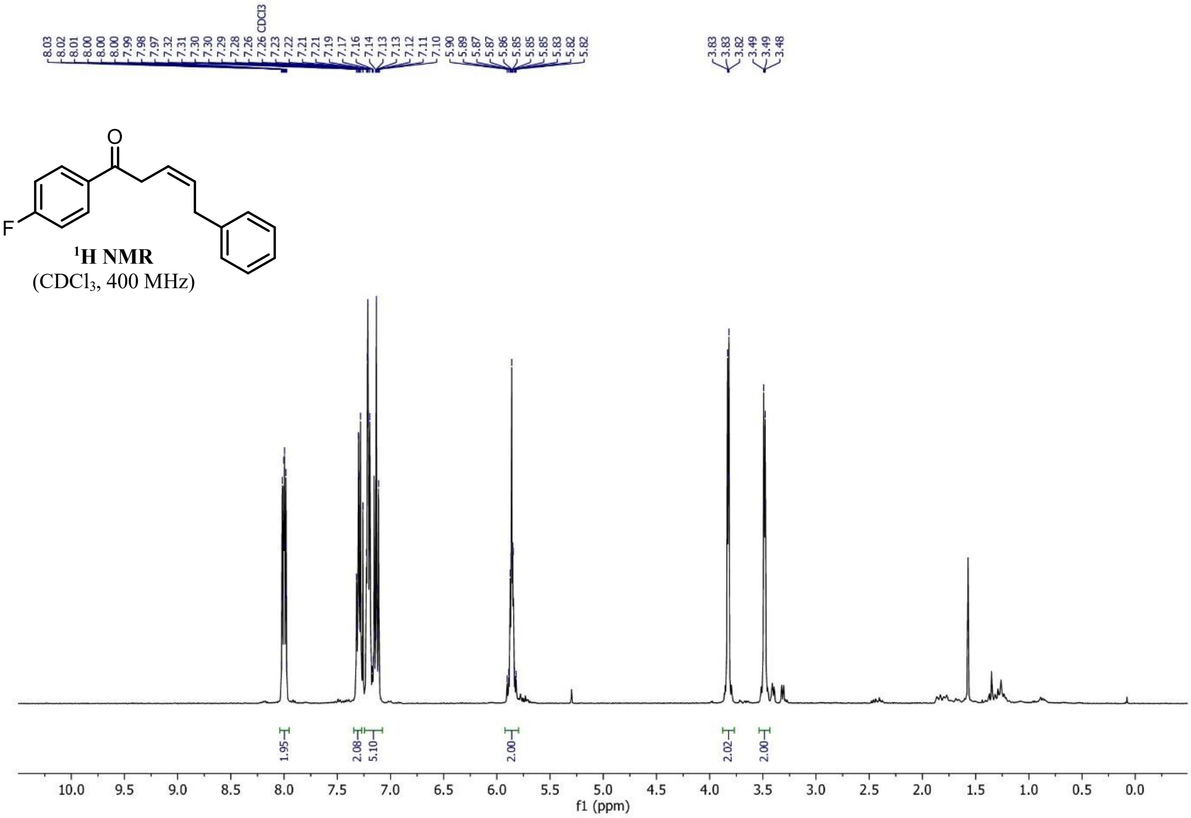

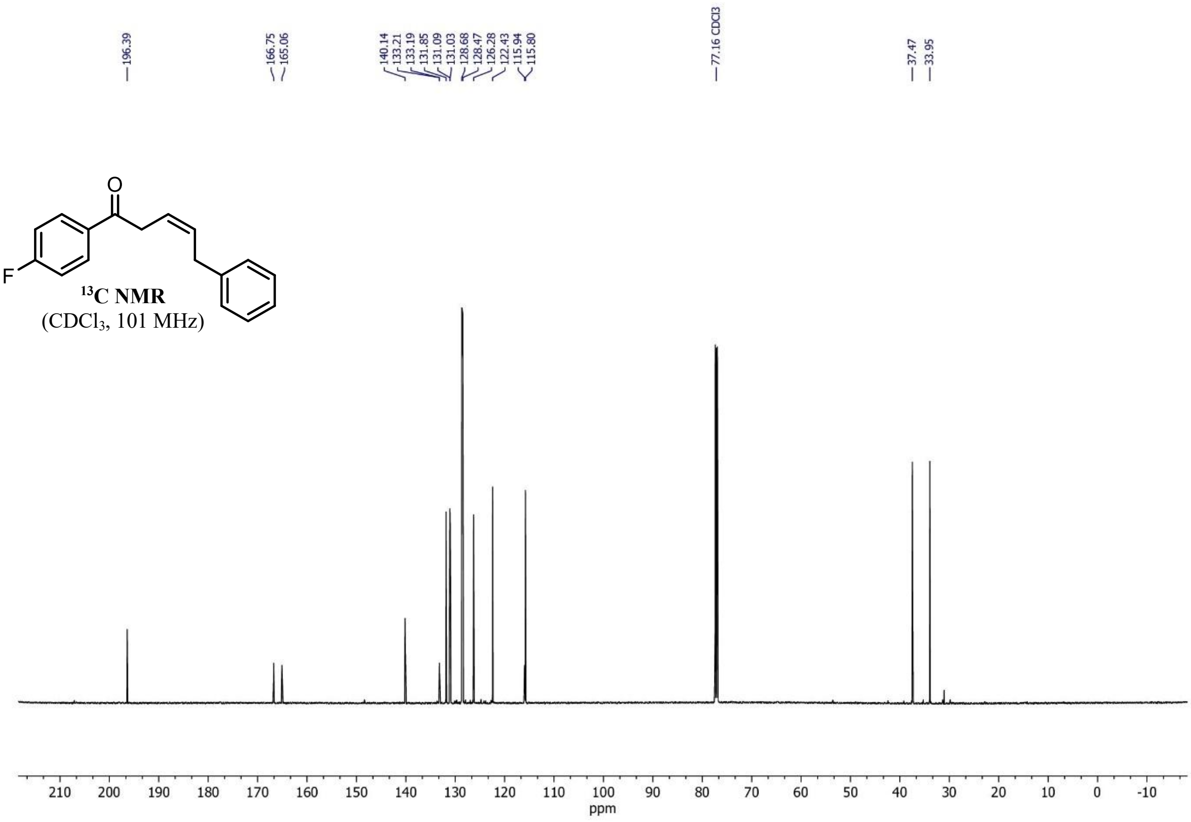

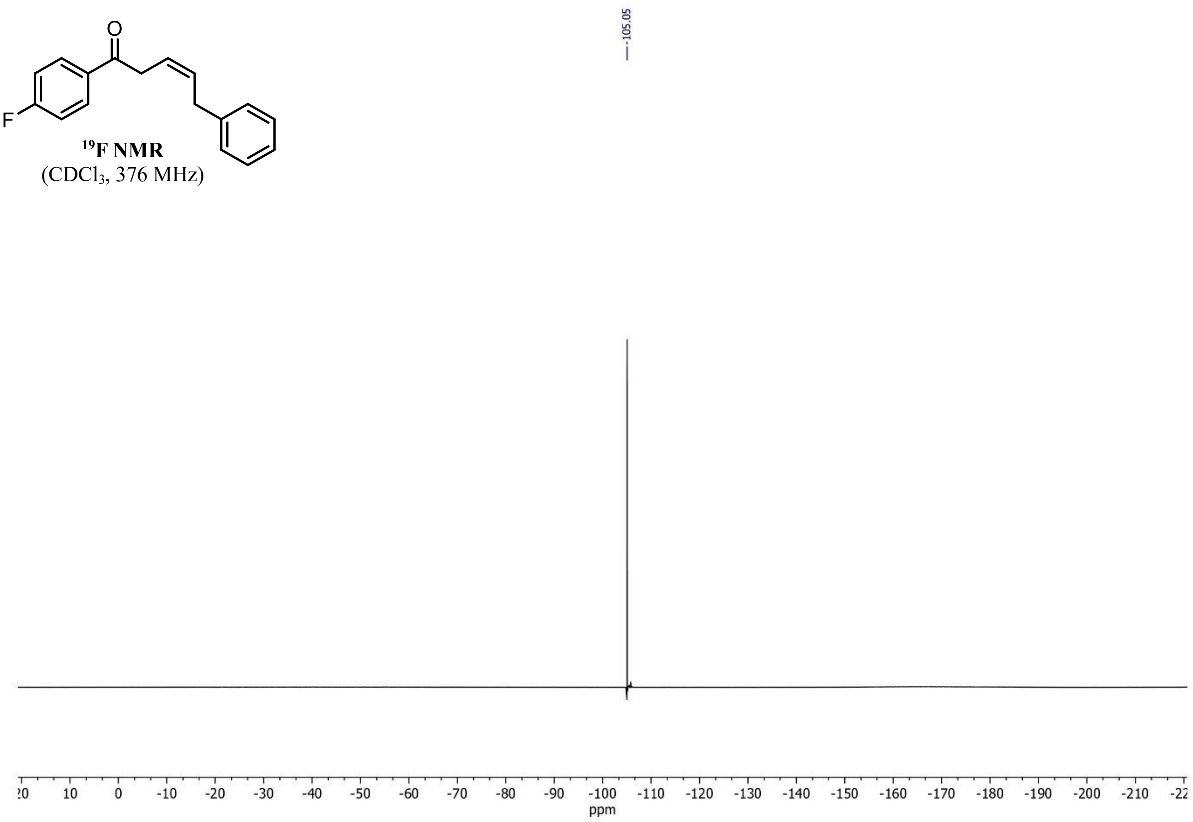
O
F
^{19}F NMR
($CDCl_3$, 376 MHz)
-105.05
20 10 0 -10 -20 -30 -40 -50 -60 -70 -80 -90 -100 -110 -120 -130 -140 -150 -160 -170 -180 -190 -200 -210 -22
ppm

(*Z*)-1-(4-Bromophenyl)-5-phenylpent-3-en-1-one (4c)

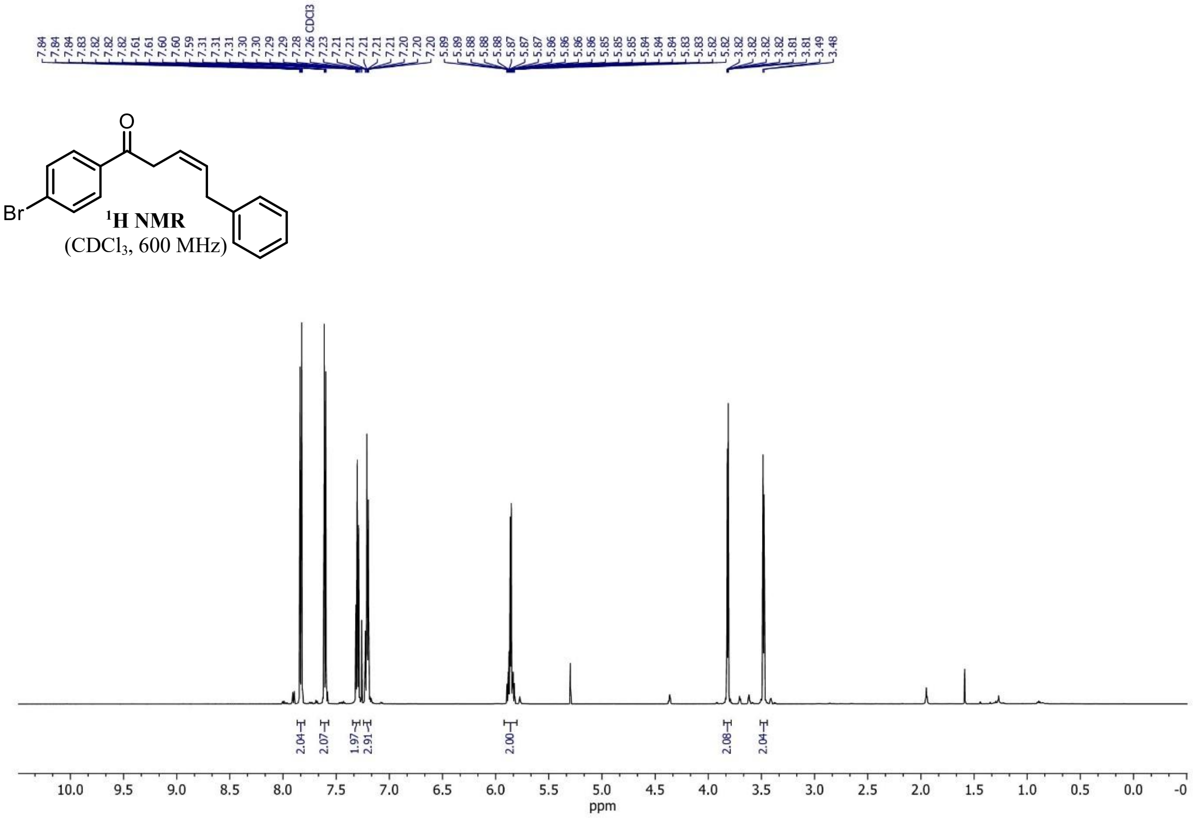

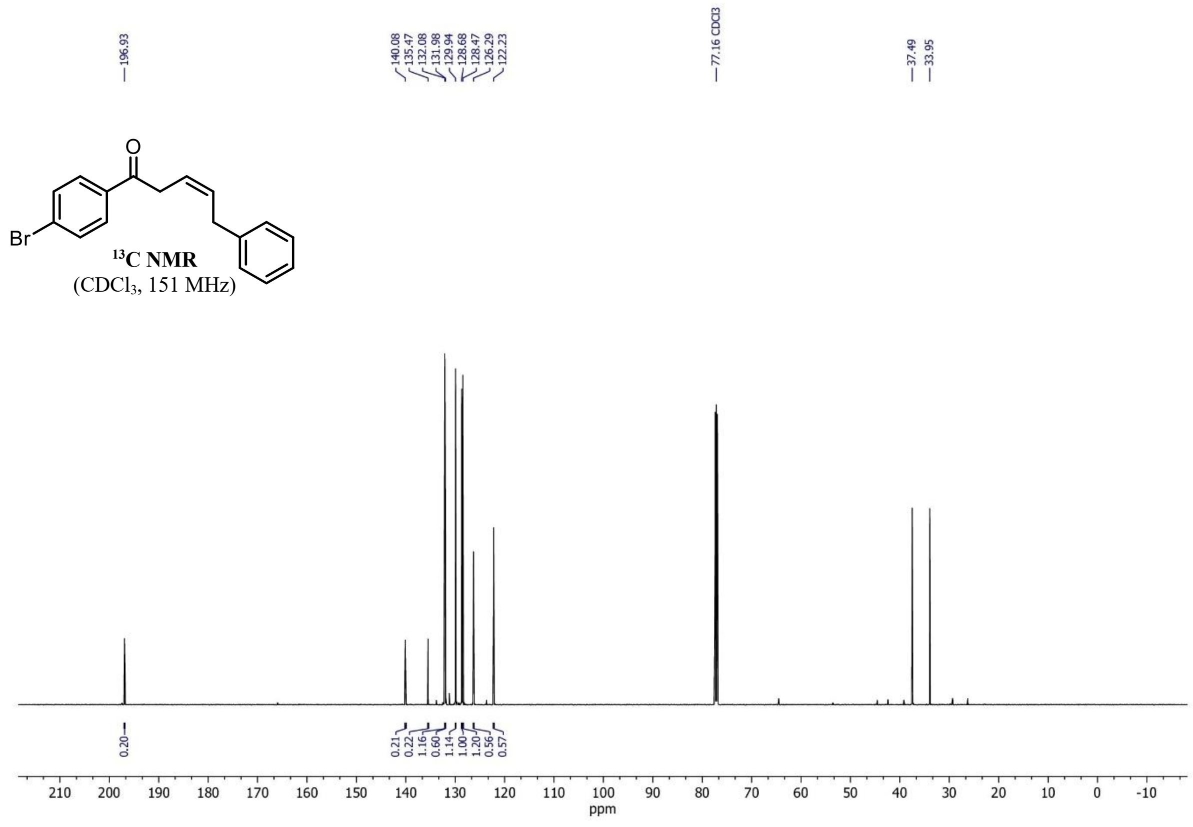

(*Z*)-1-(4-Iodophenyl)-5-phenylpent-3-en-1-one (4d)

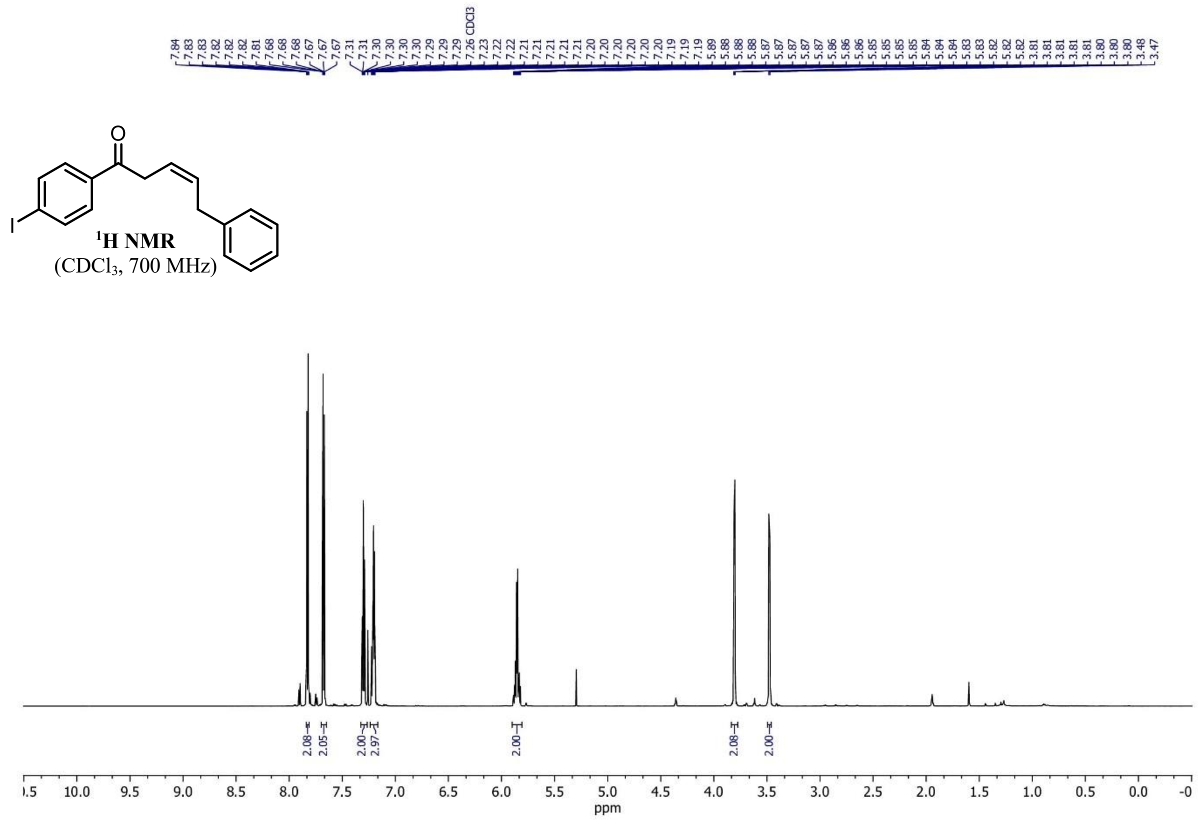

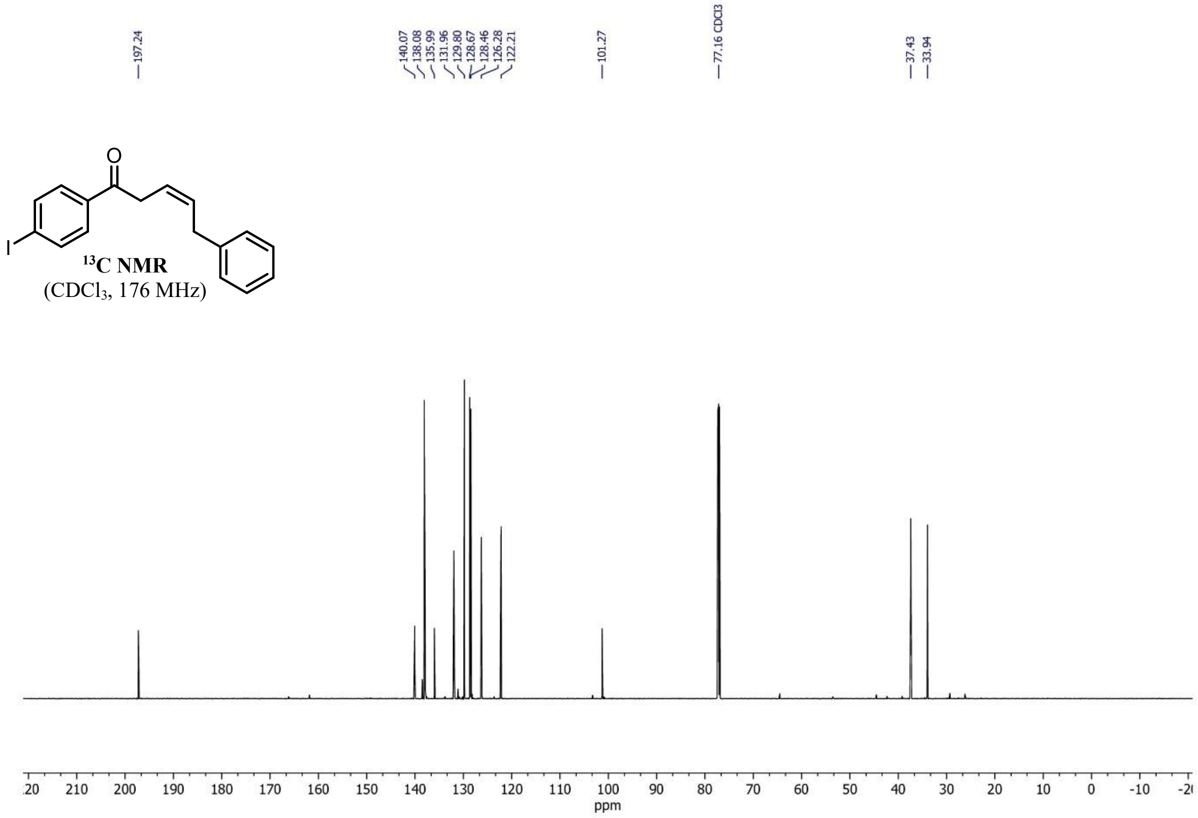

(*Z*)-1-(4-(*tert*-Butyl)phenyl)-5-phenylpent-3-en-1-one (4e)

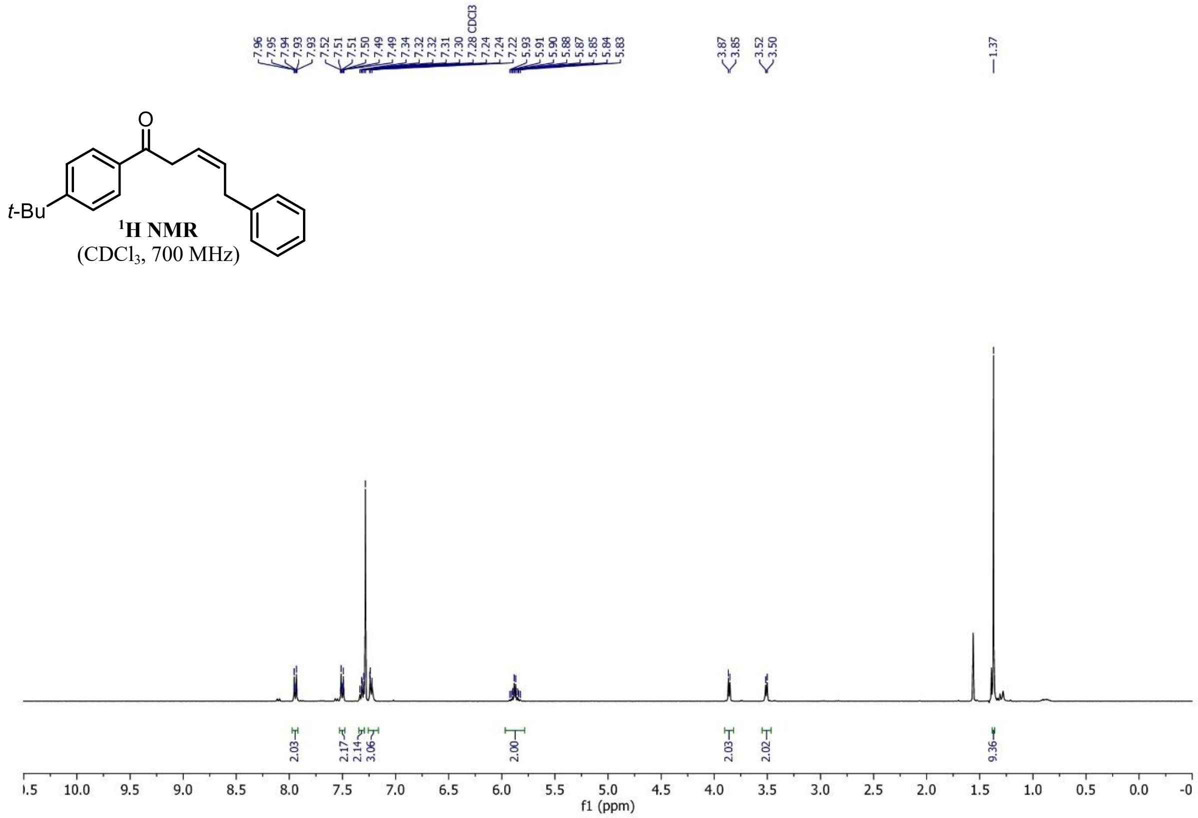

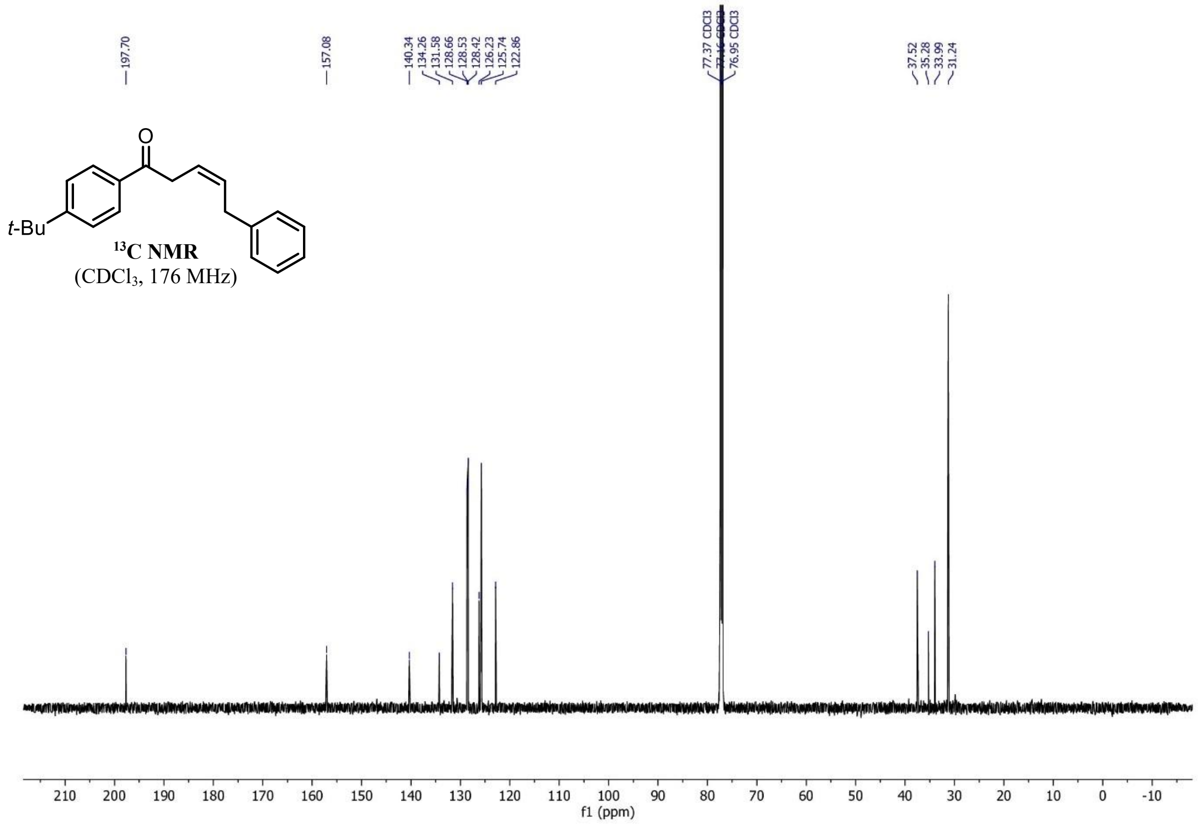

(*Z*)-1-(4-Methoxyphenyl)-5-phenylpent-3-en-1-one (4f)

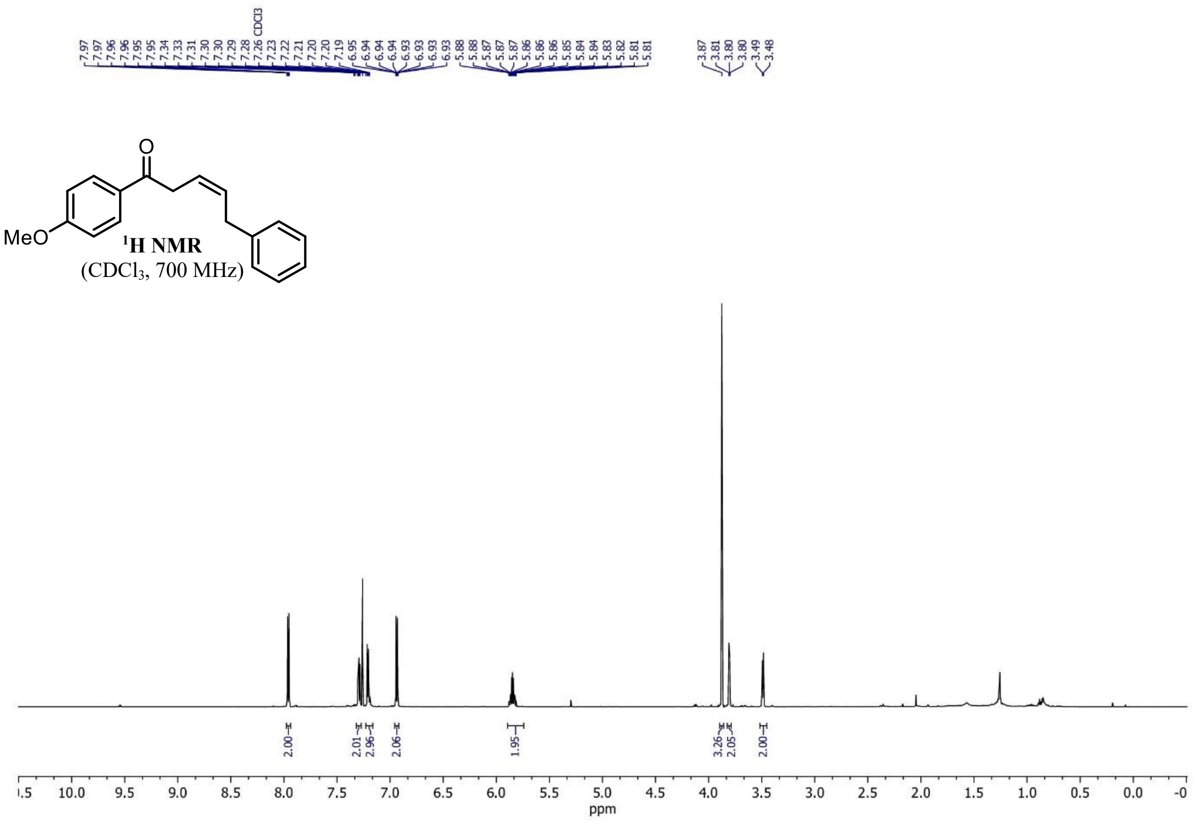

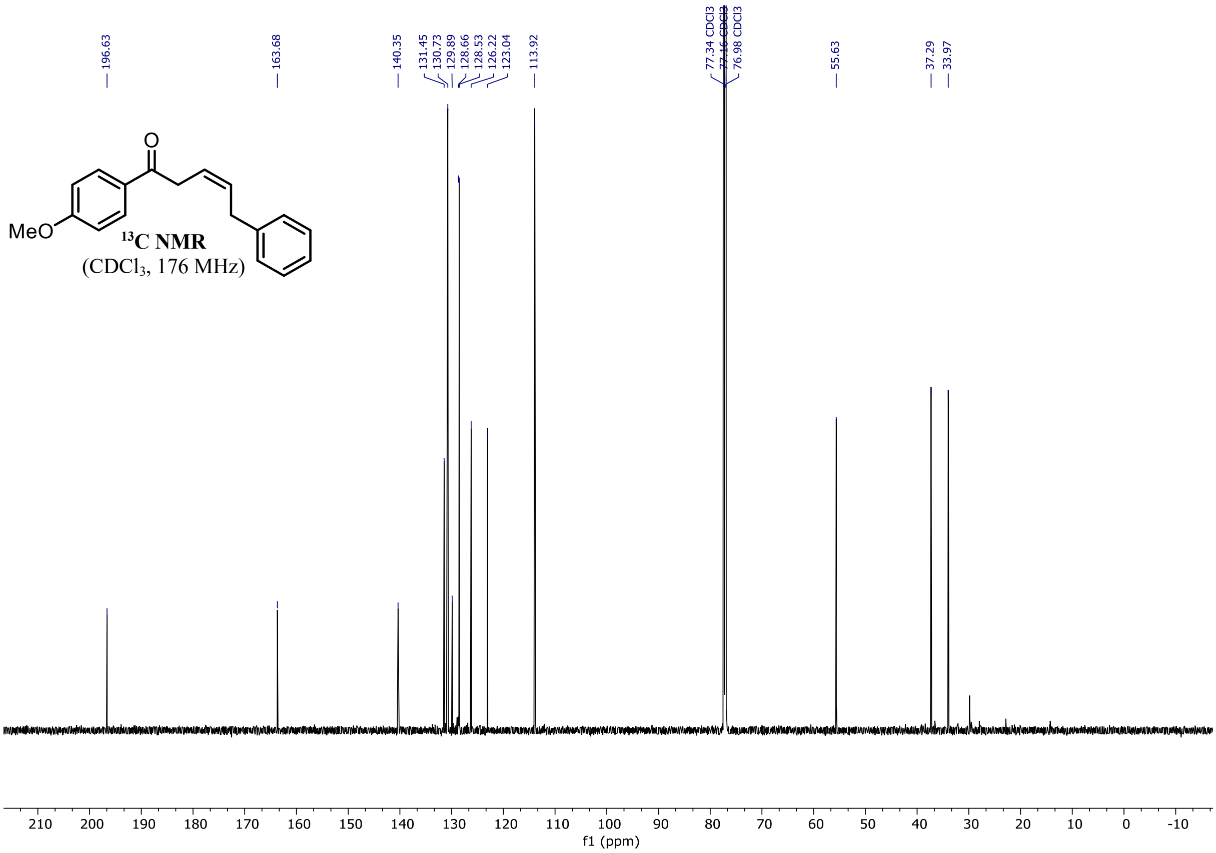

(*Z*)-5-Phenyl-1-(thiophen-2-yl)pent-3-en-1-one (4g)

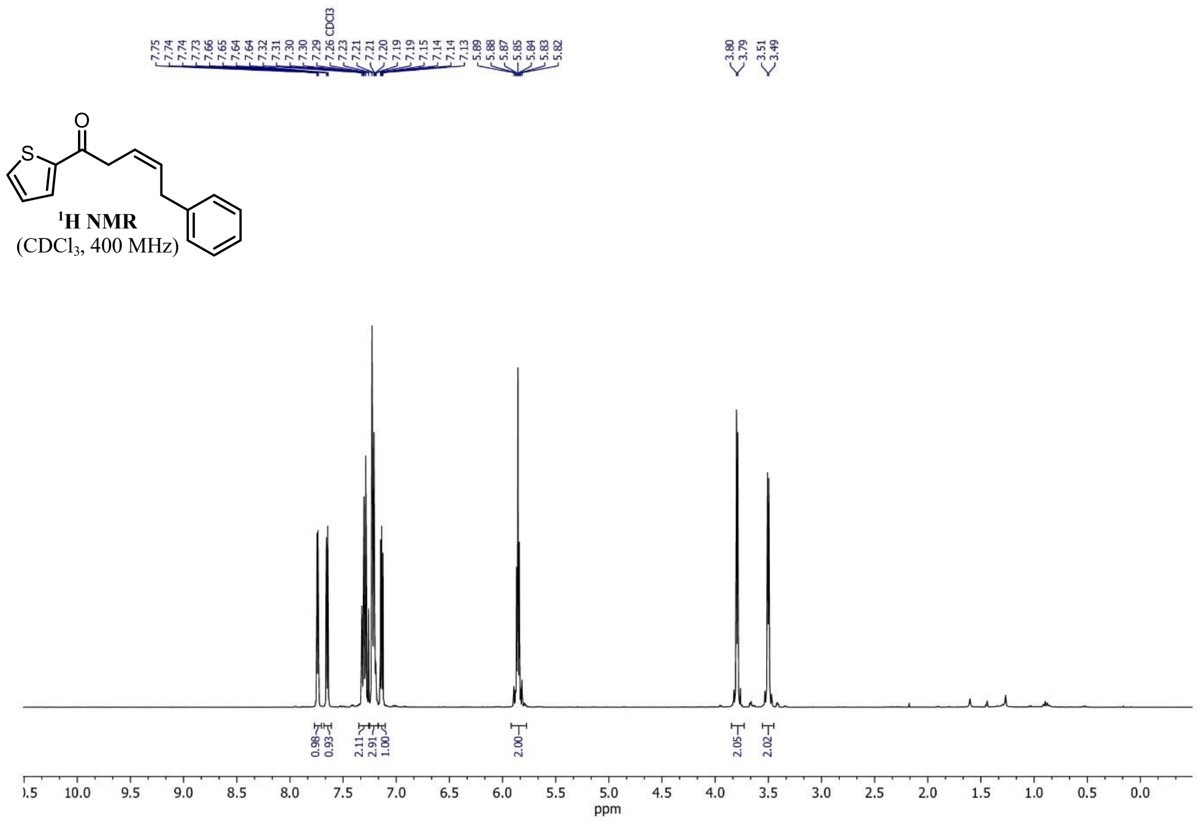

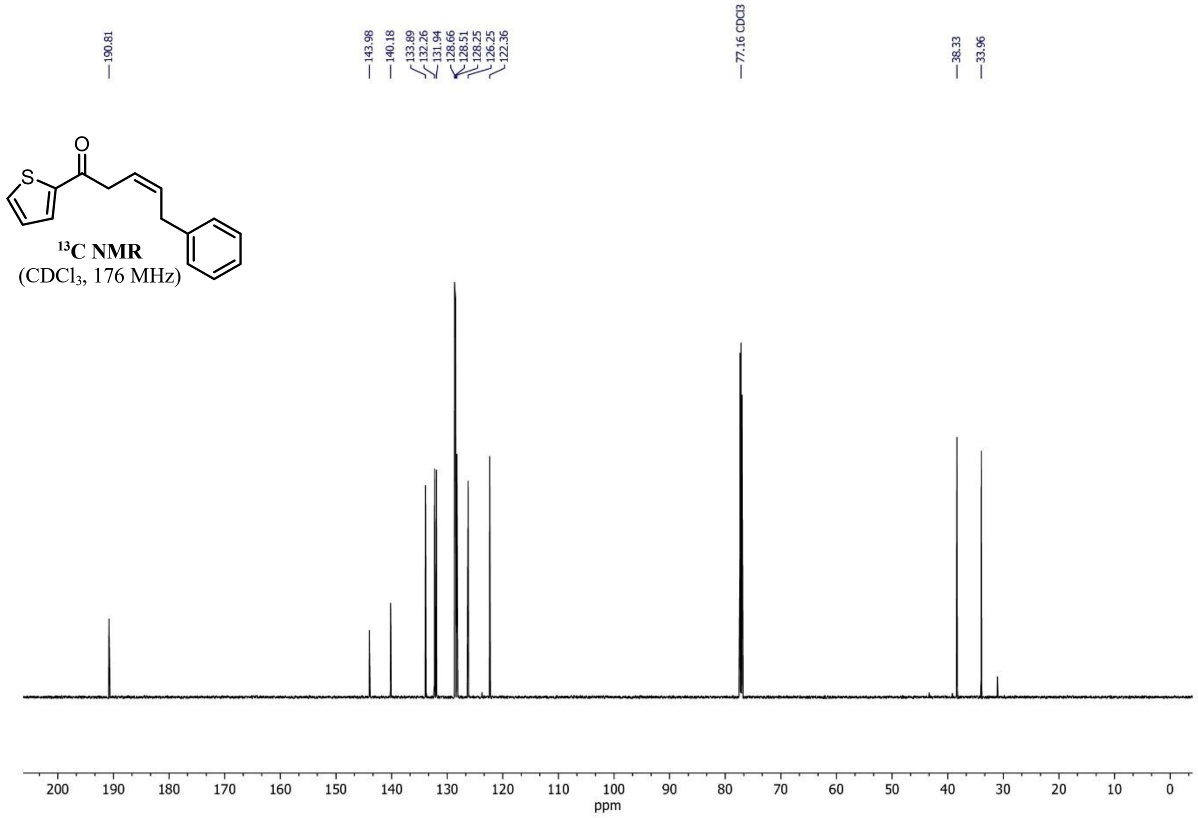

(2*E*,6*Z*)-8-Phenylocta-2,6-dien-4-one (4h)

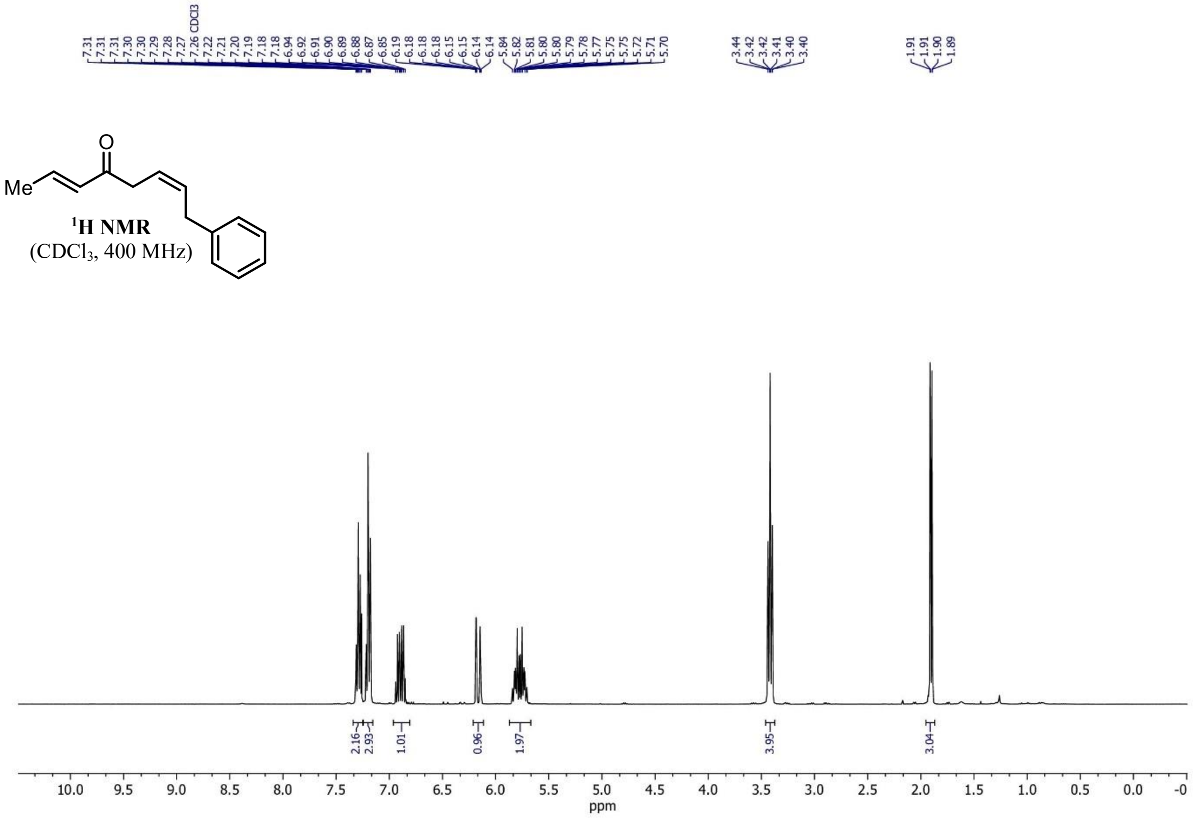

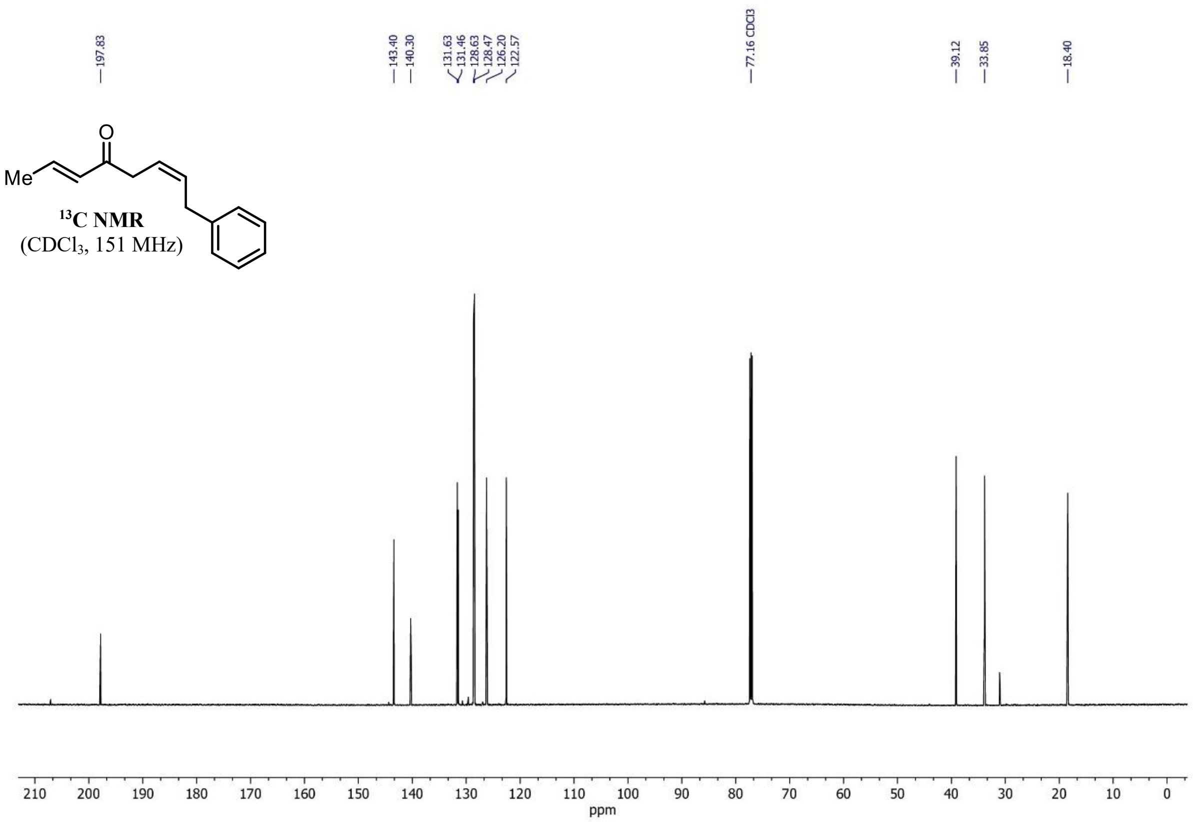

(1*E*,5*Z*)-1,7-Diphenylhepta-1,5-dien-3-one (4i)

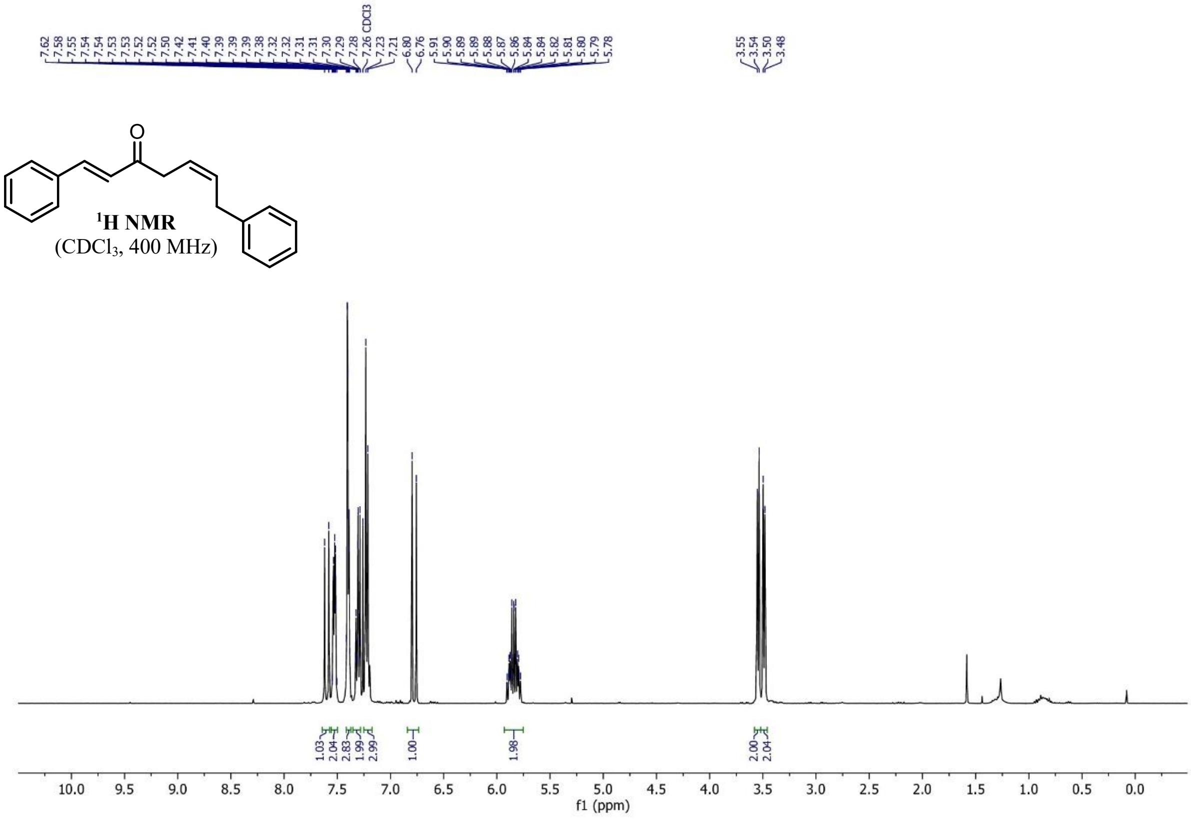

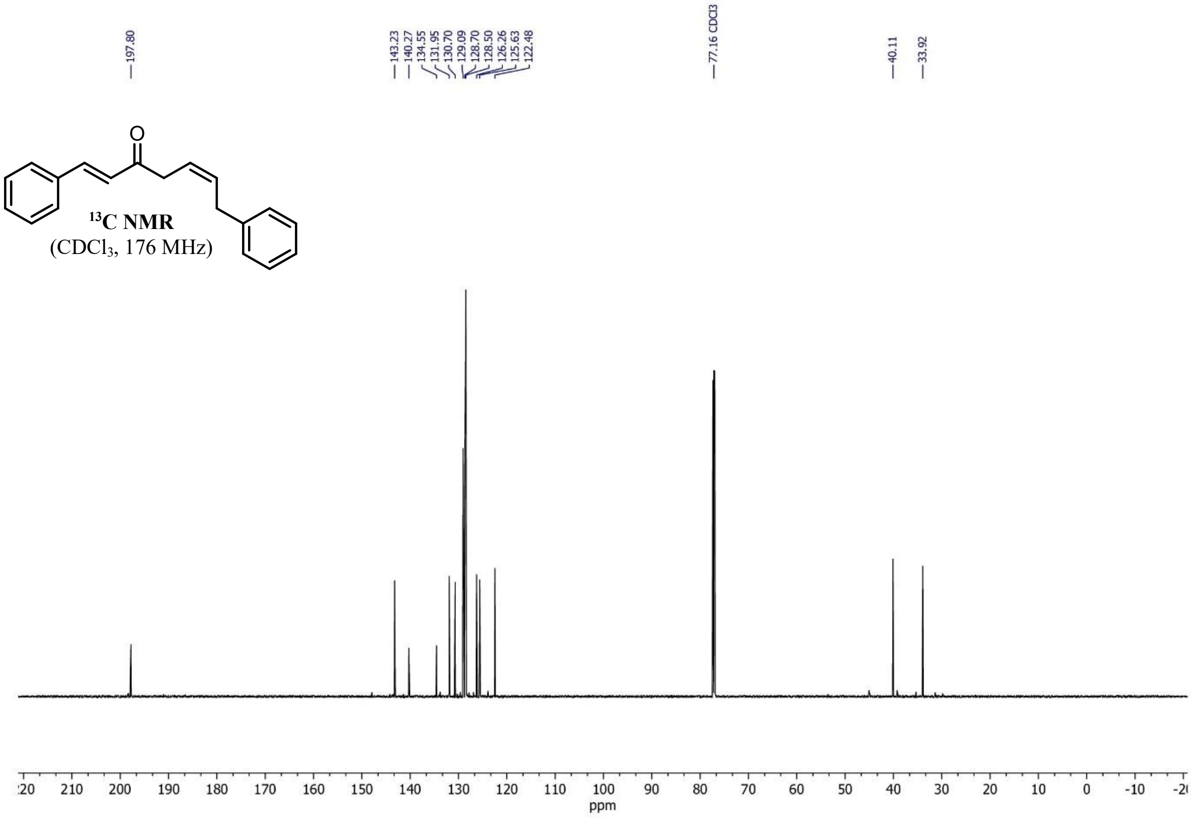

(1*E*,5*Z*)-1-(Benzo[*d*][1,3]dioxol-5-yl)-7-phenylhepta-1,5-dien-3-one (4j)

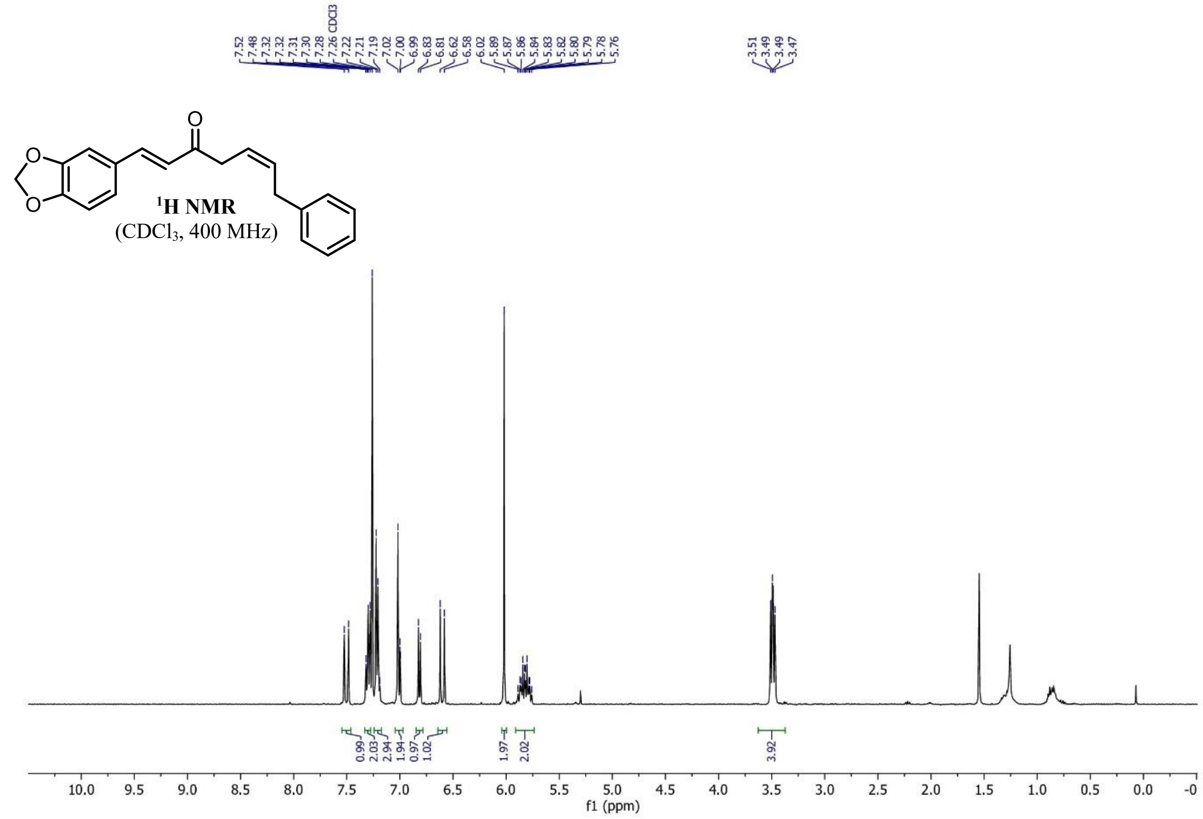

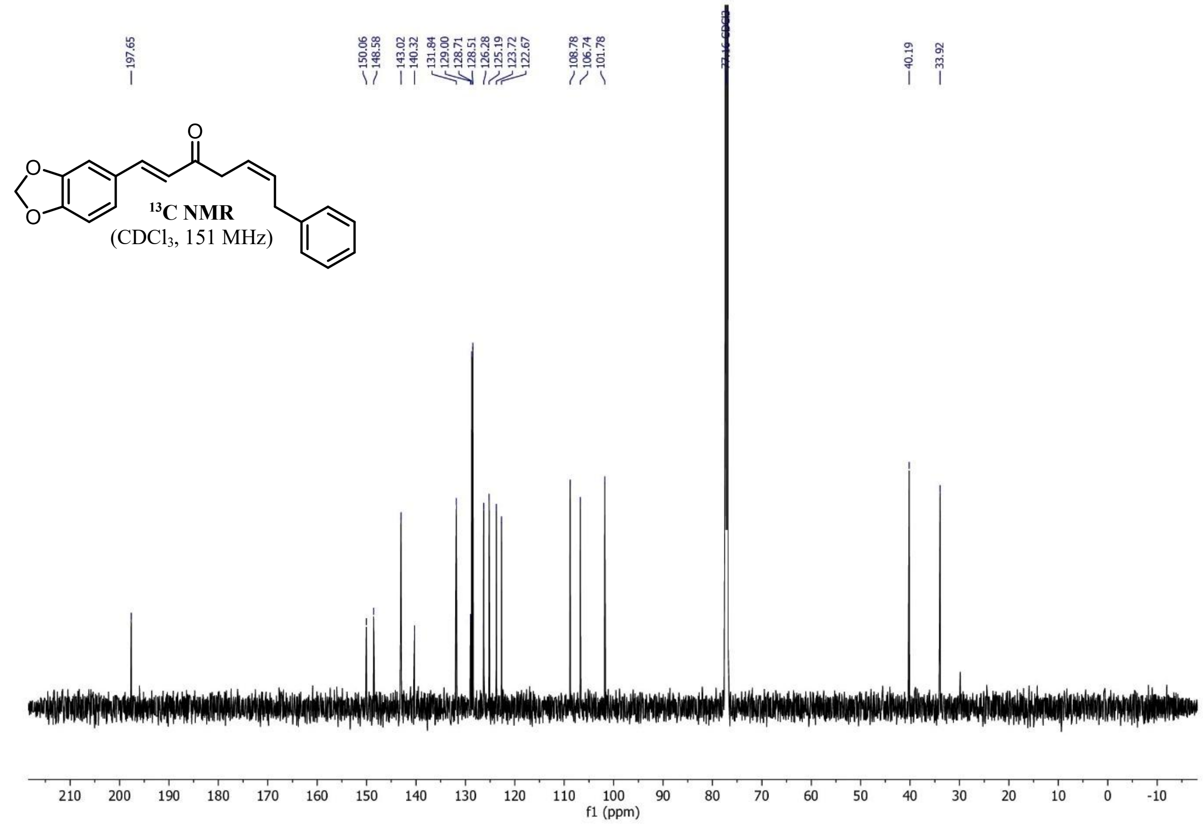

(*Z*)-2,2-Dimethyl-7-phenylhept-5-en-3-one (4k)

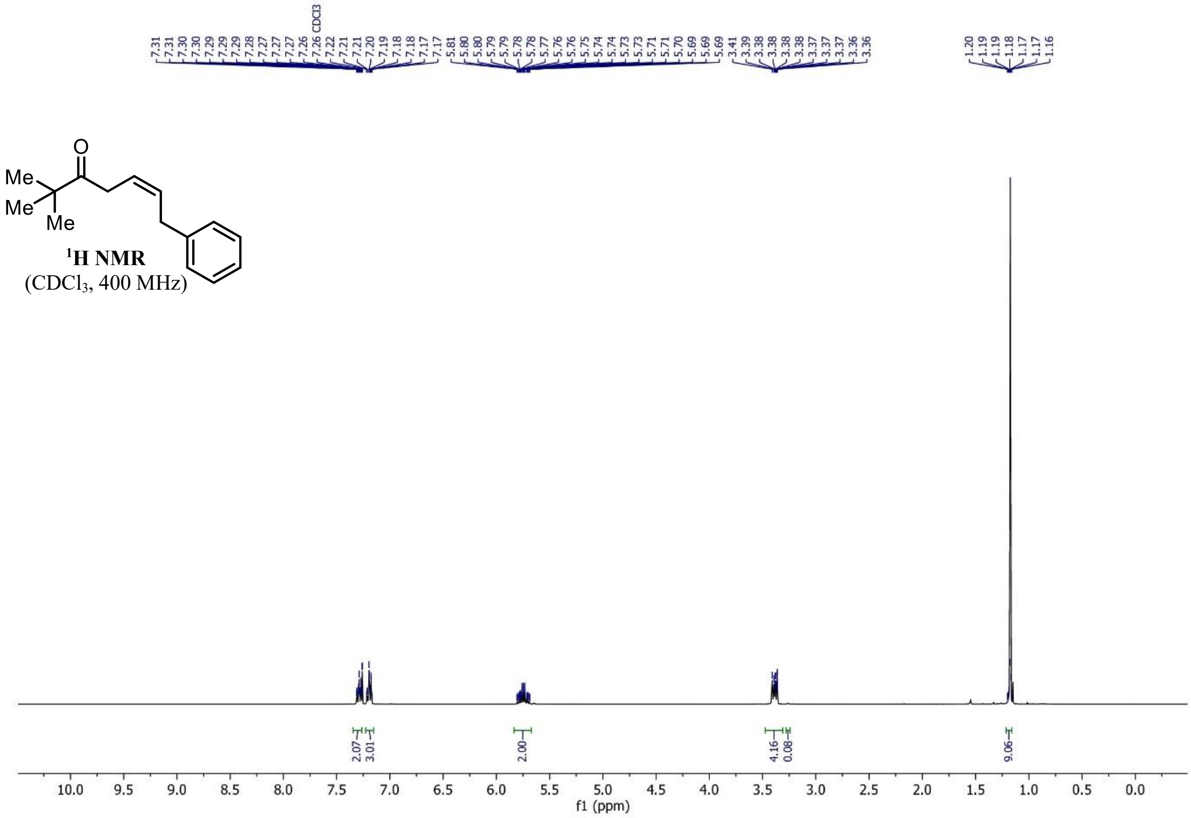

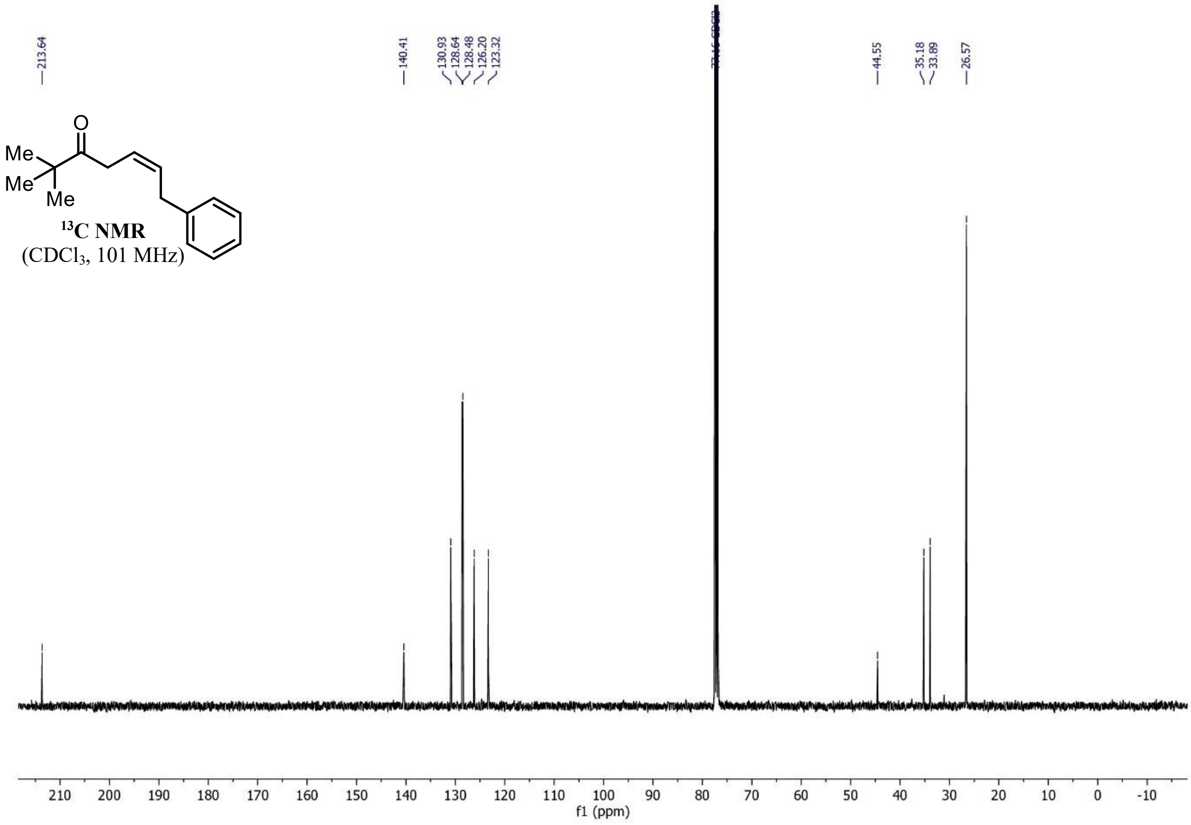

(*Z*)-2-Methyl-7-phenylhept-5-en-3-one (4l)

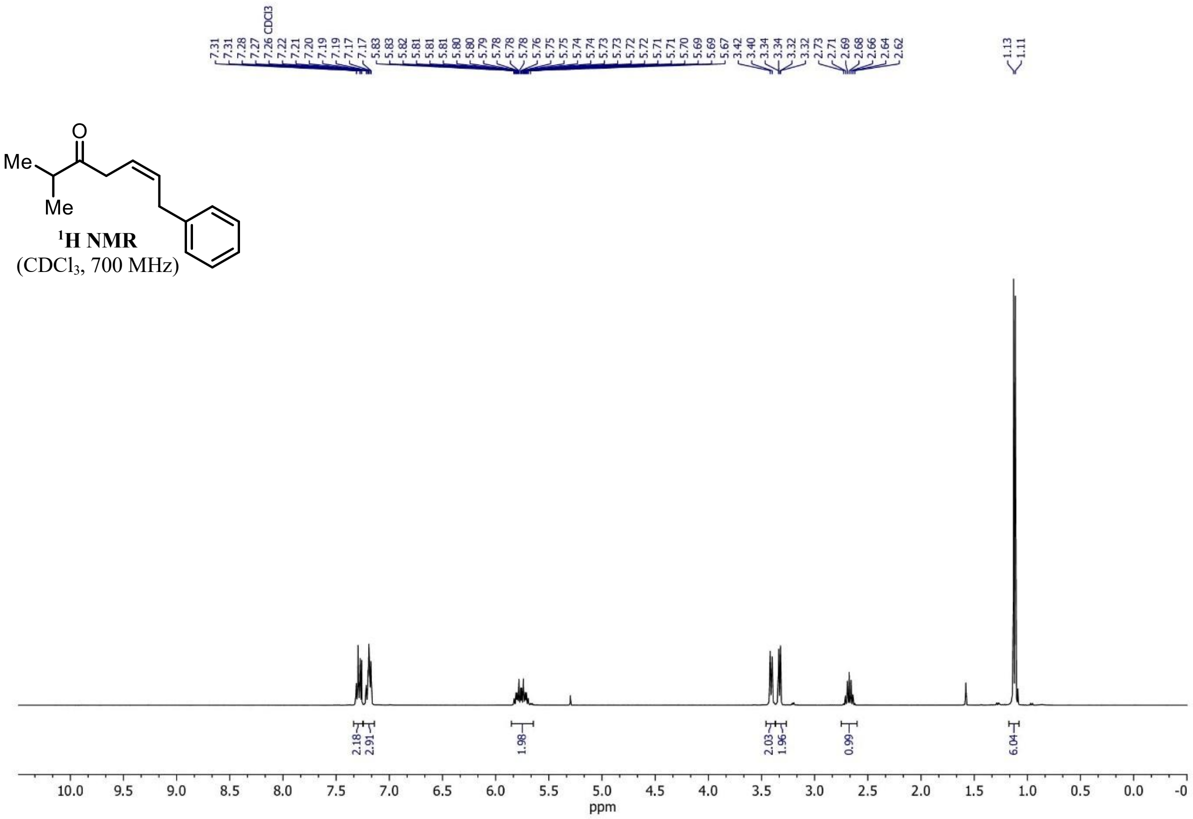

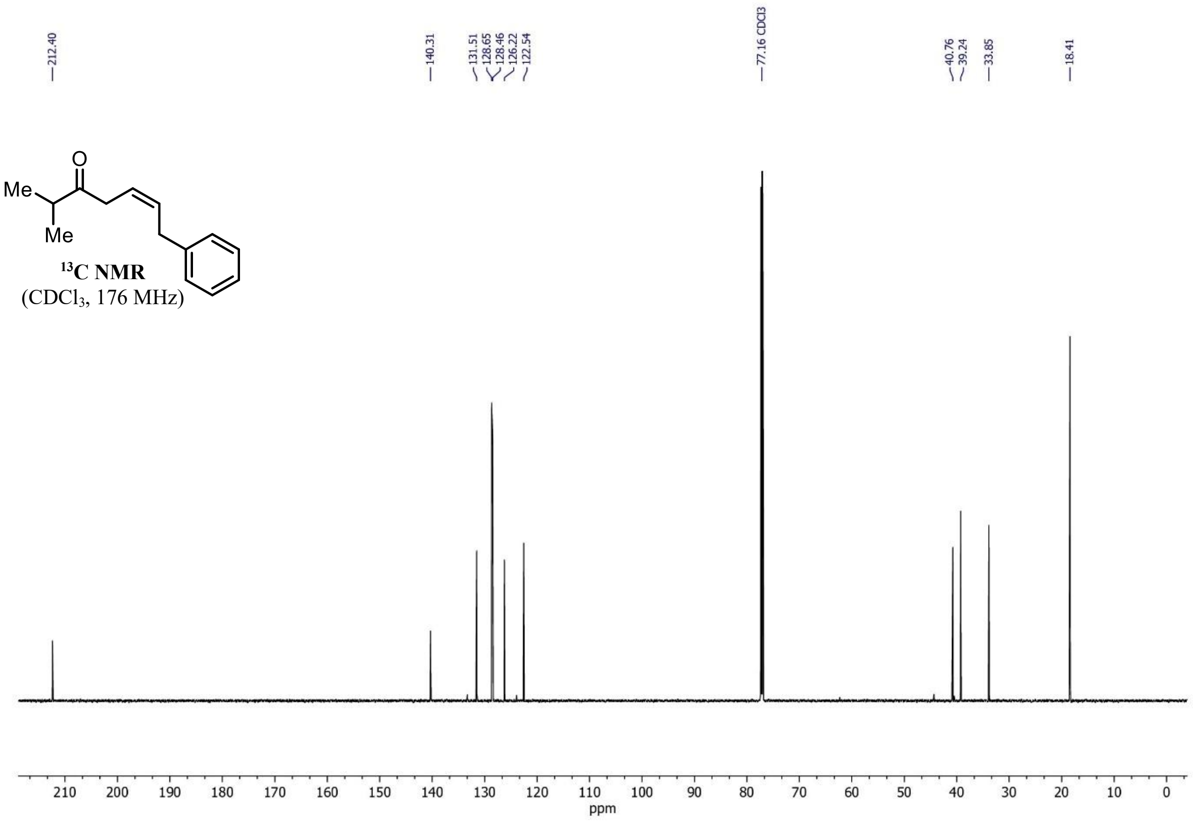

(*Z*)-1-Cyclohexyl-5-phenylpent-3-en-1-one (4m)

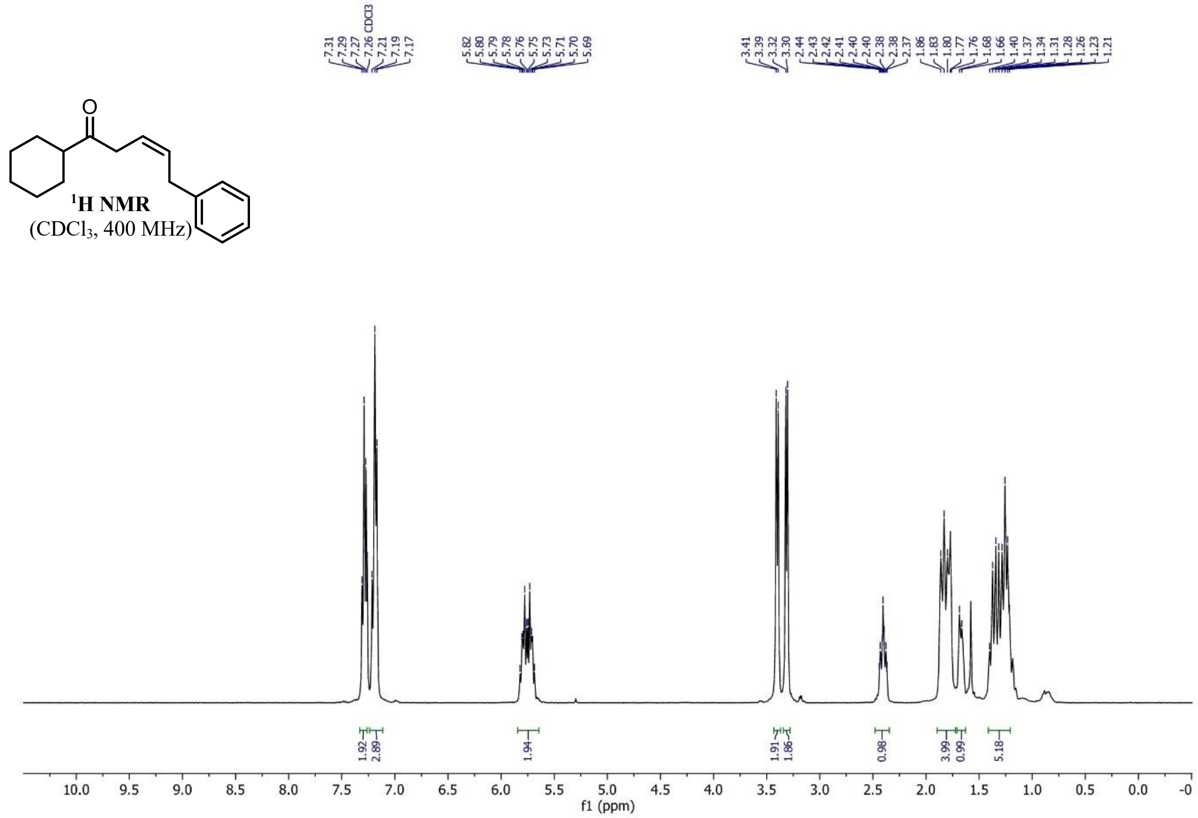

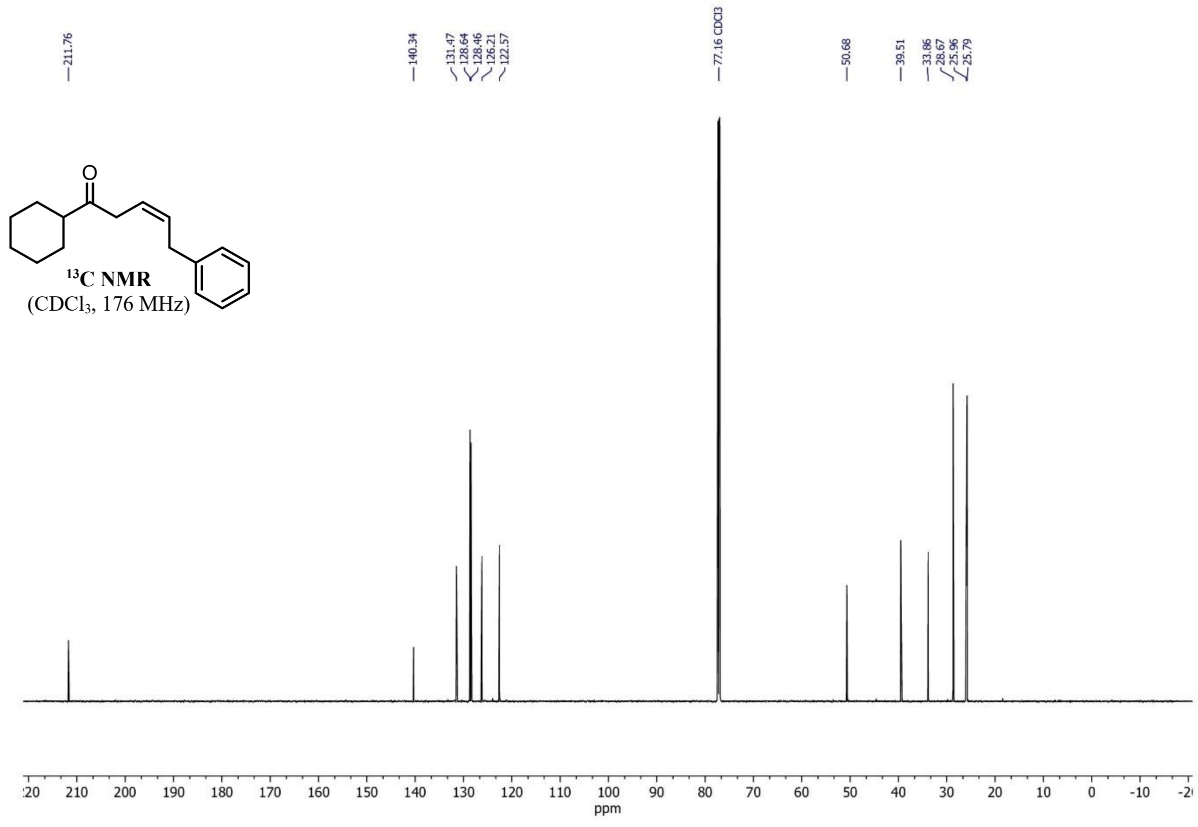

(*Z*)-1-Cyclopropyl-5-phenylpent-3-en-1-one (4n)

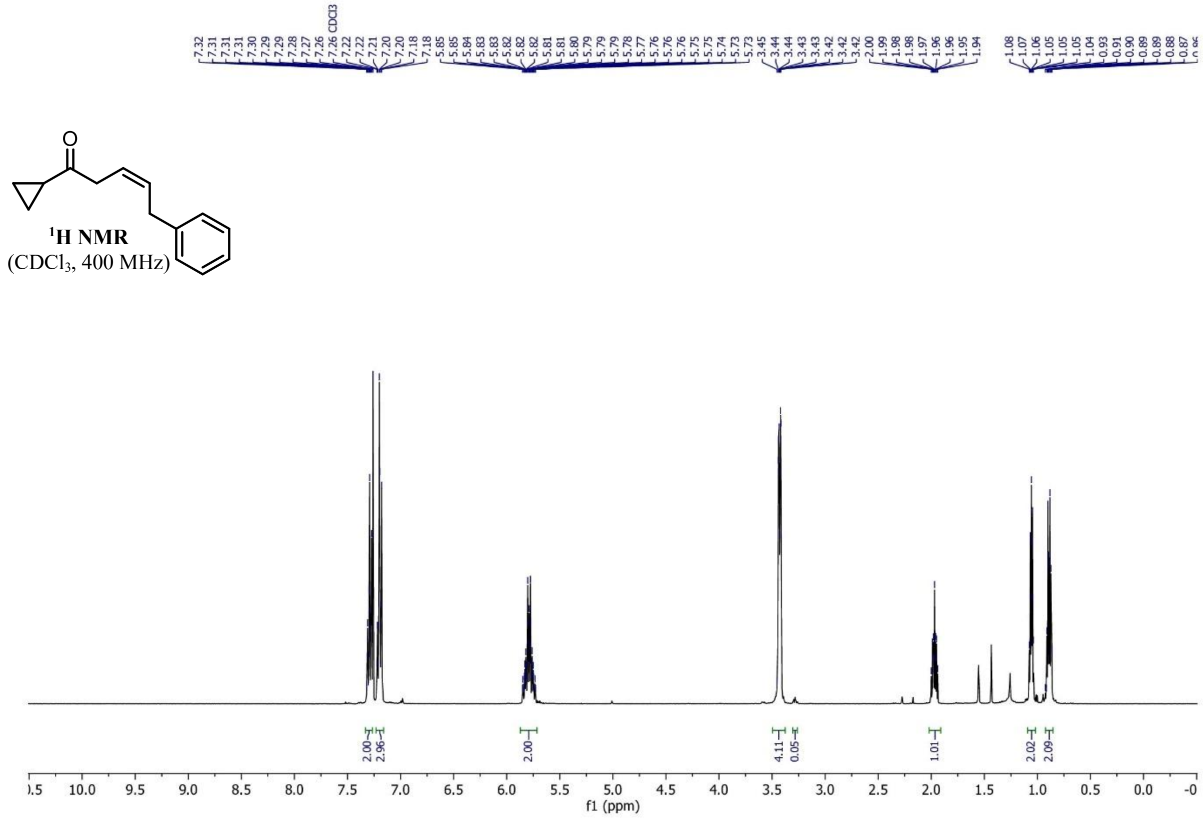

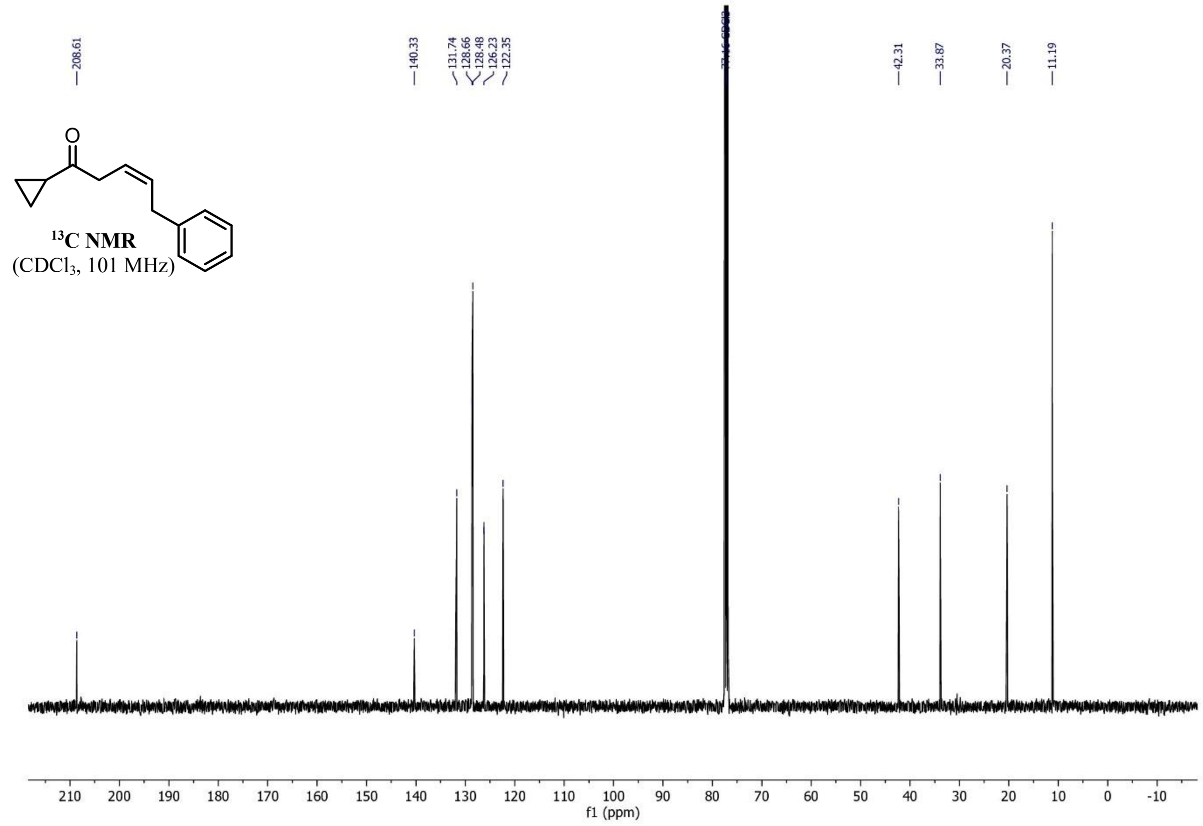

(*Z*)-1-Phenylhexadec-2-en-5-one (4o)

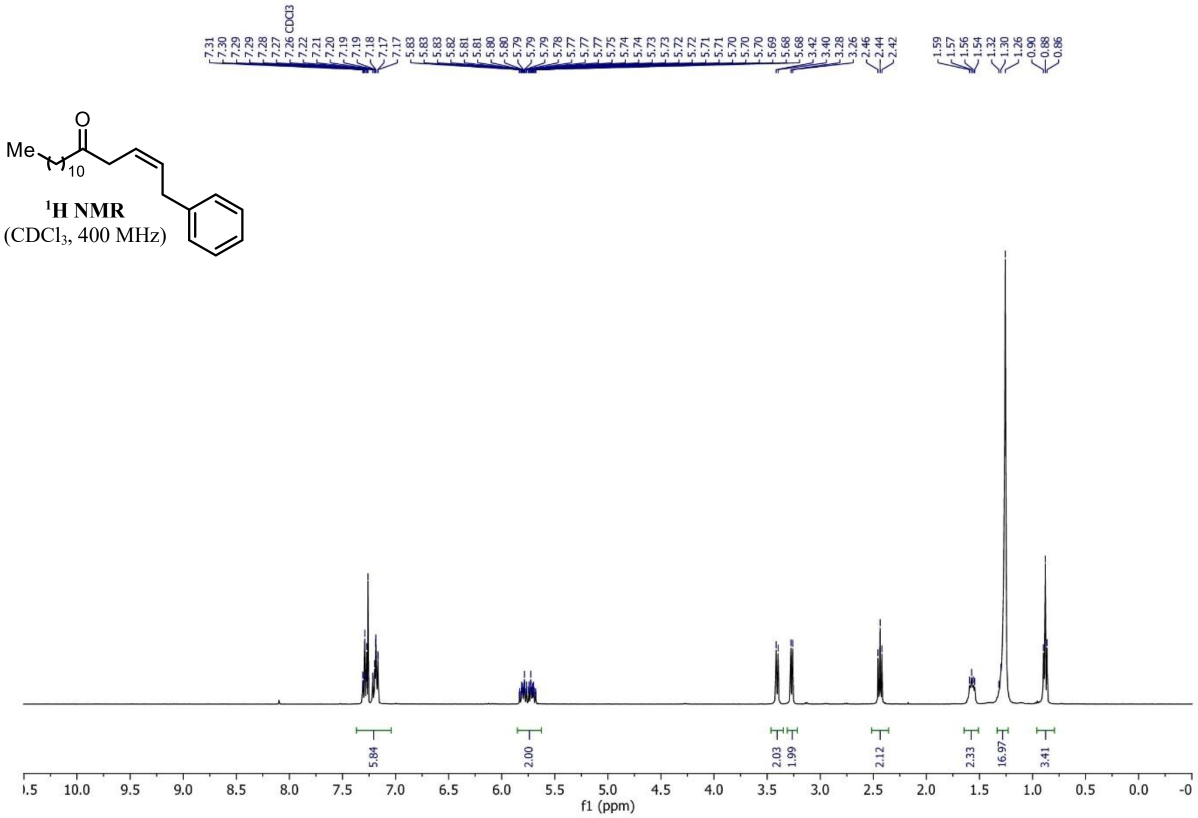

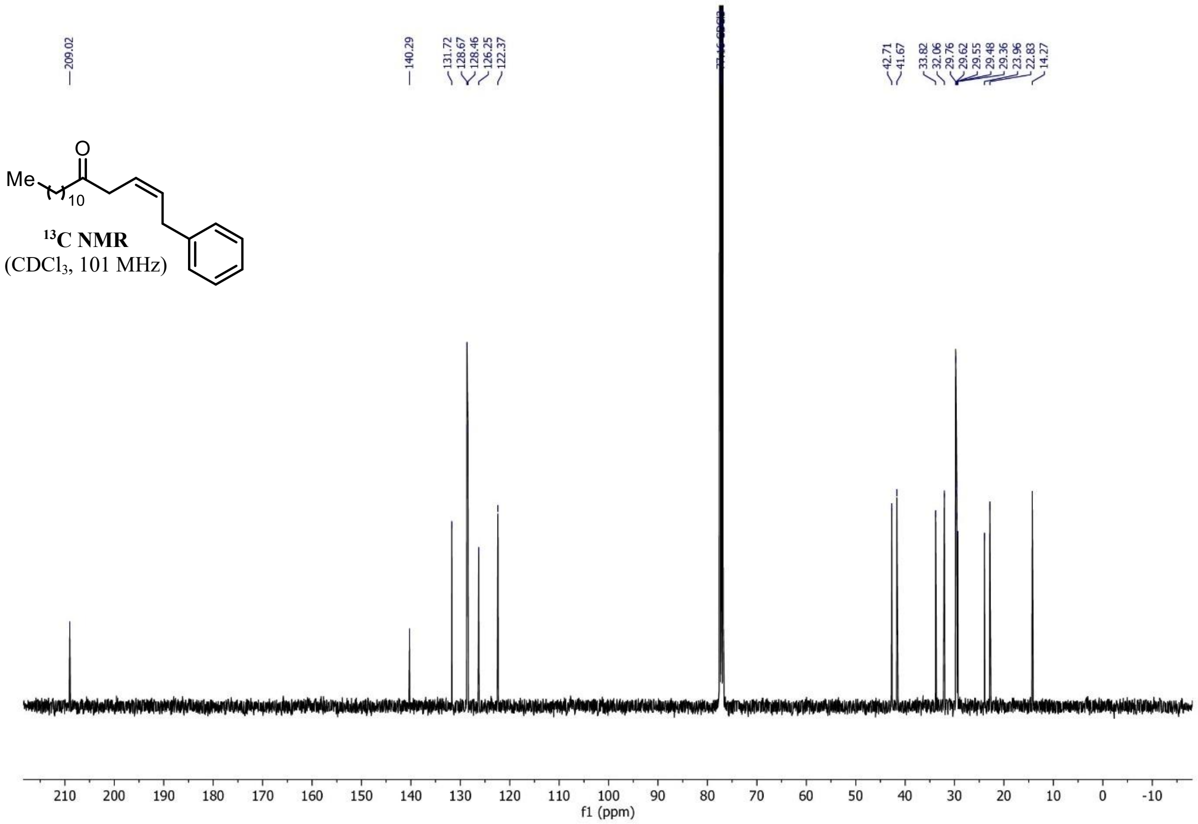

(*Z*)-6-Phenylhex-4-en-2-one **(4p)**

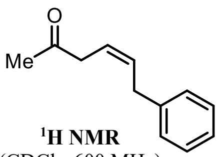

^{1}H NMR
($CDCl_3$, 600 MHz)

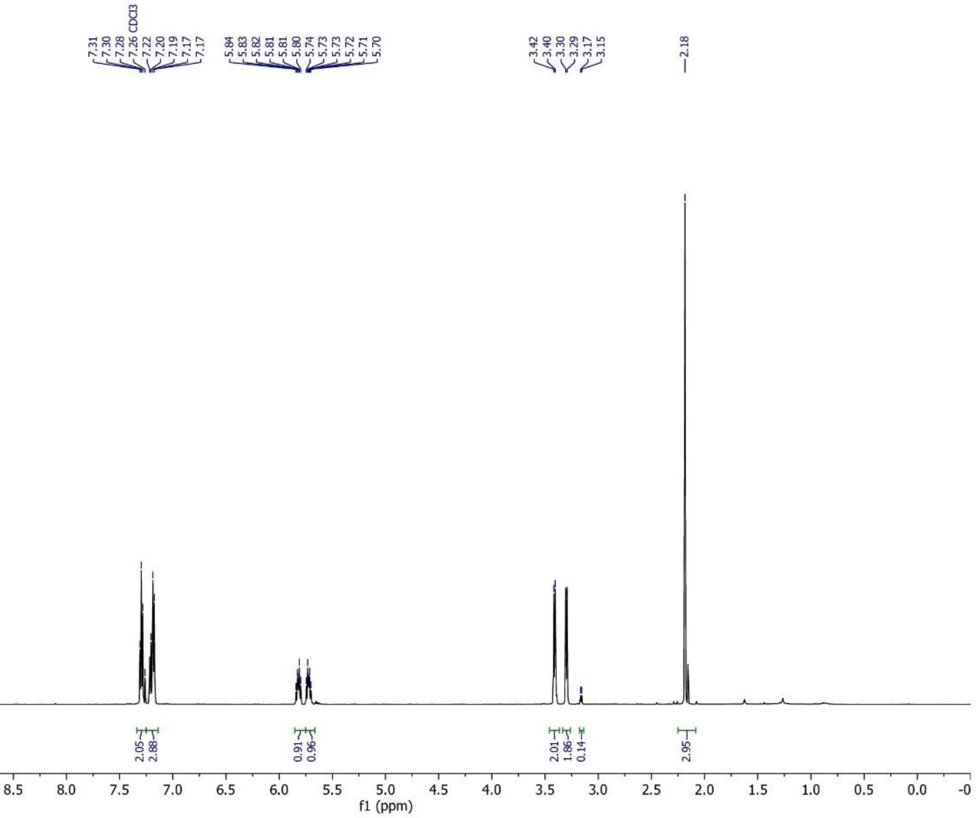

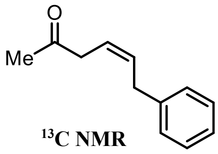

^{13}C NMR
($CDCl_3$, 151 MHz)

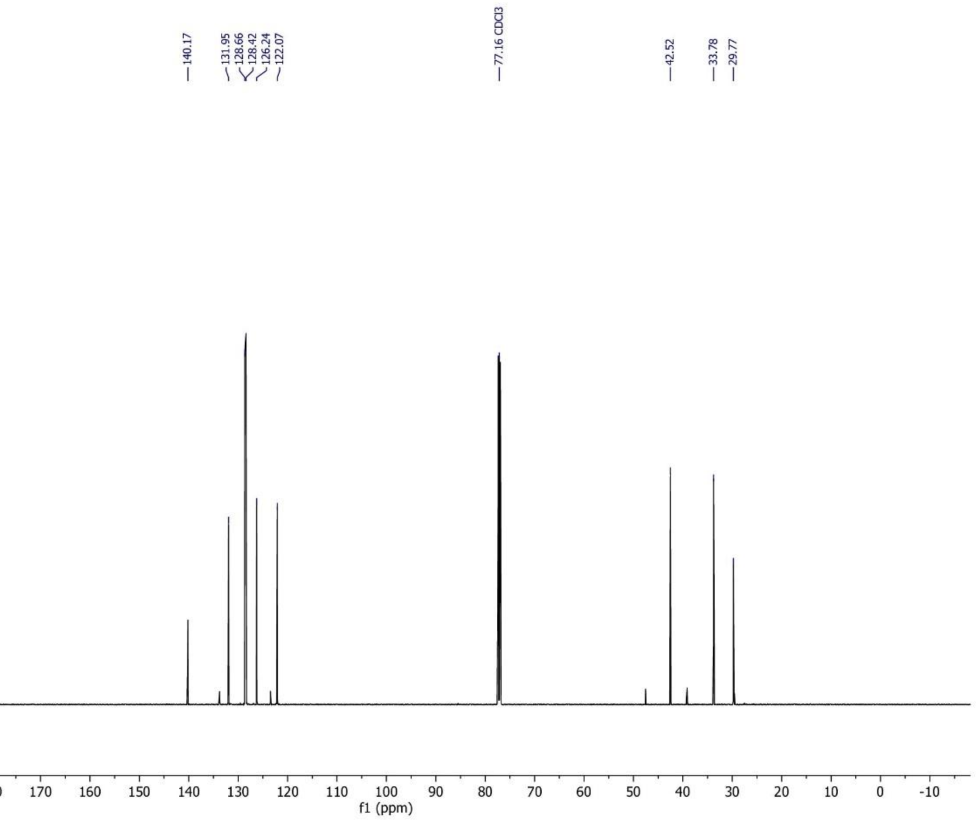

(*Z*)-1,6-Diphenylhex-4-en-2-one (4q)

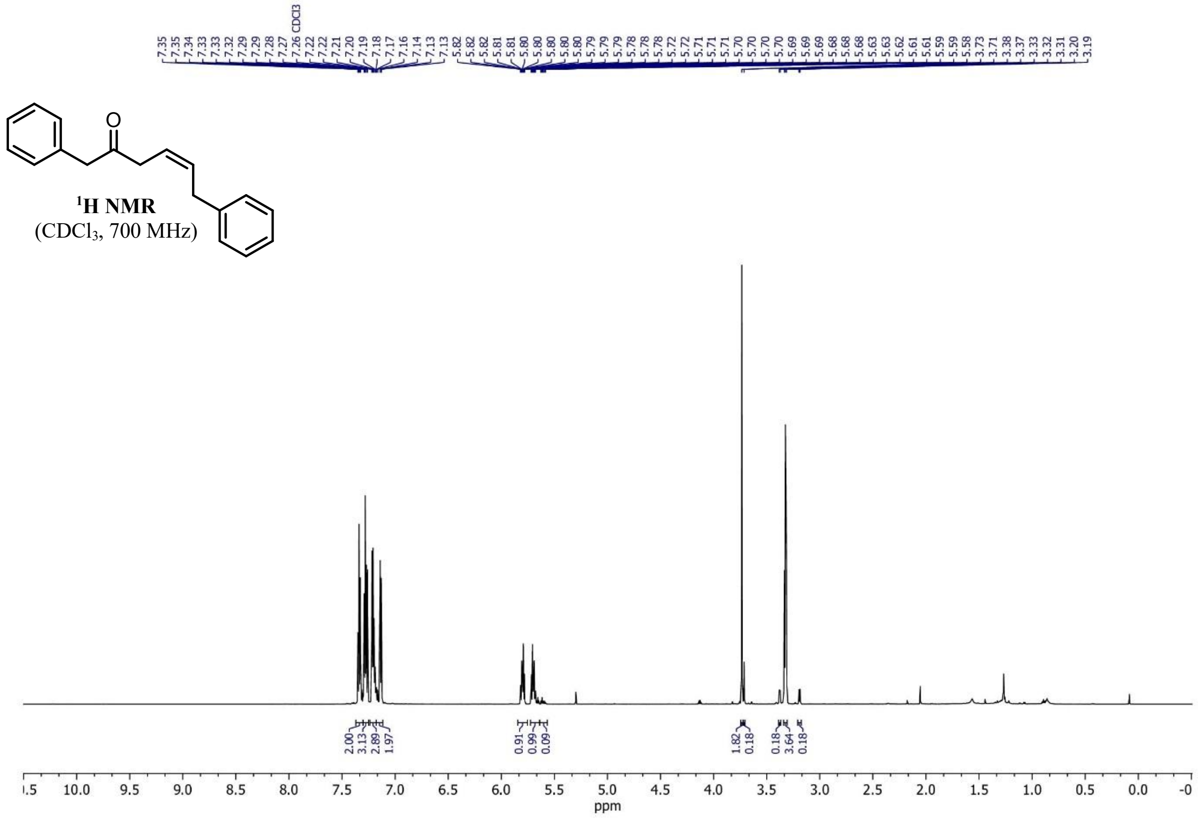

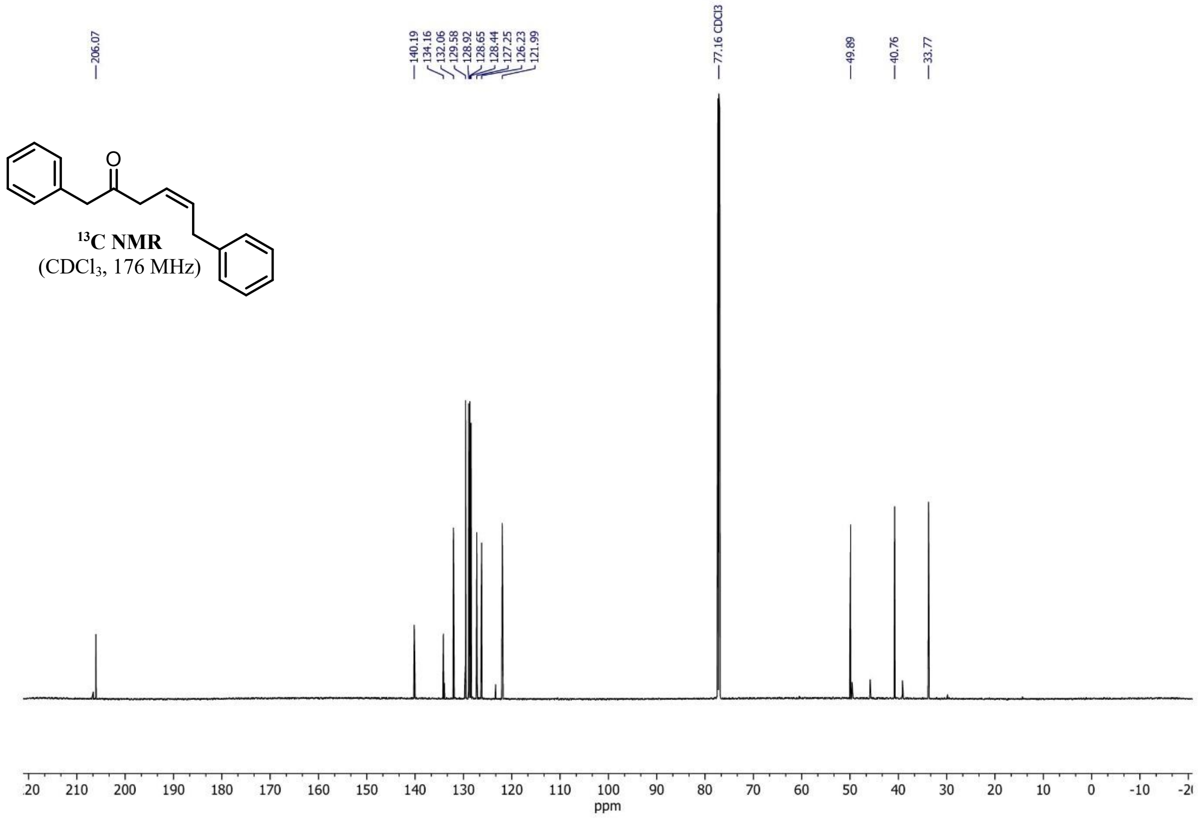

(*Z*)-2-Methyl-8-phenyloct-6-en-4-one (4r)

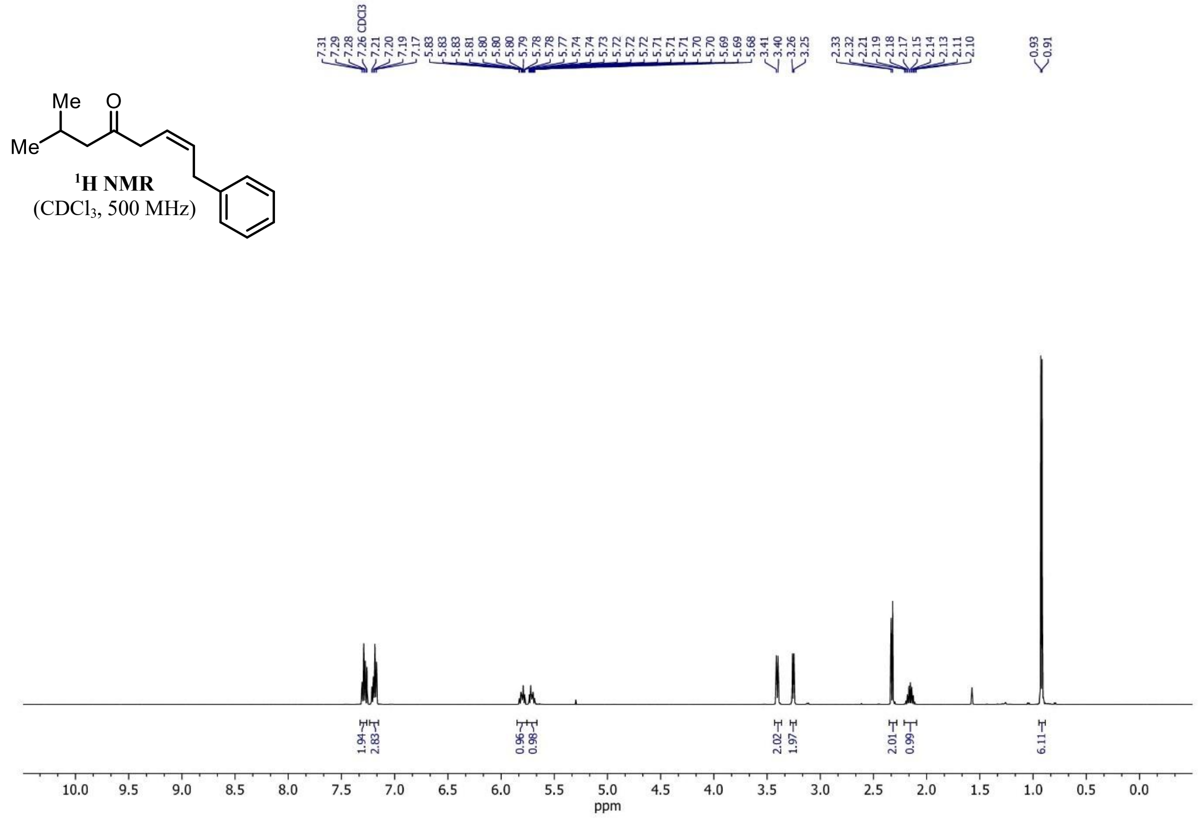

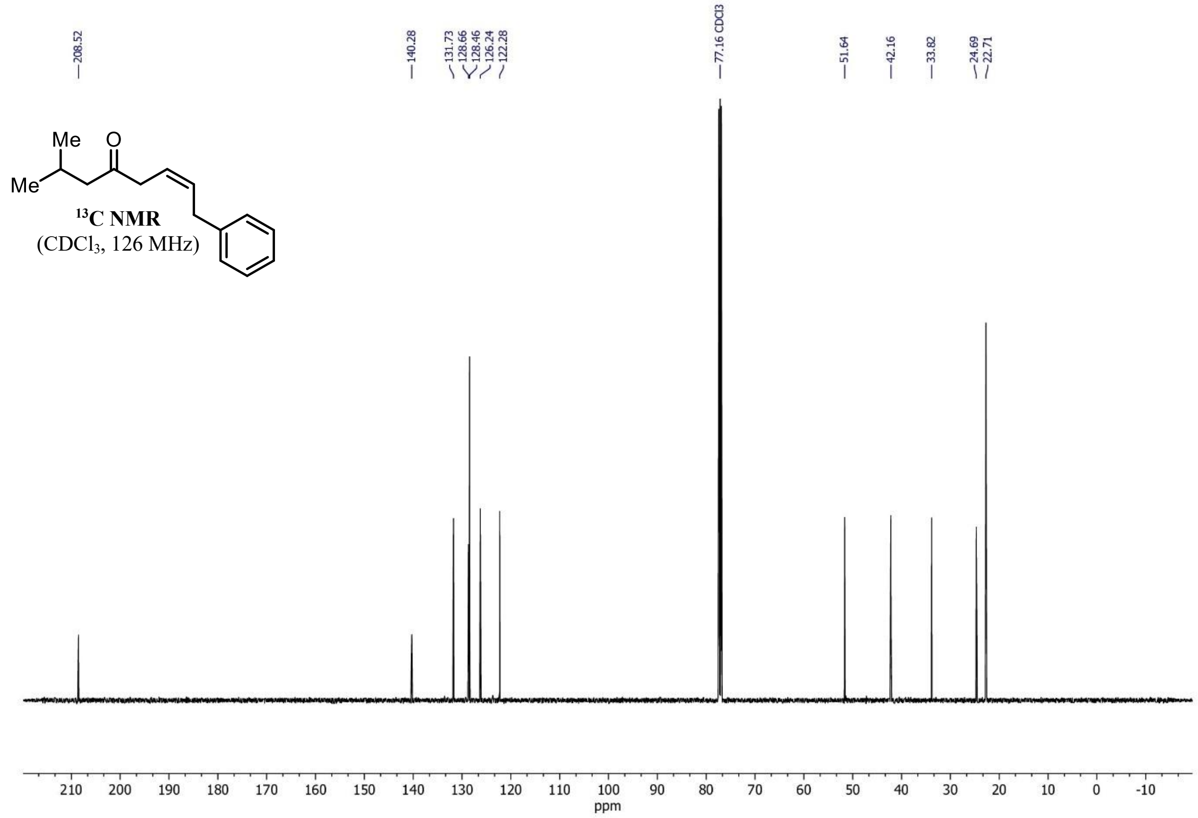

(*Z*)-1-Cyclopentyl-7-phenylhept-5-en-3-one (4s)

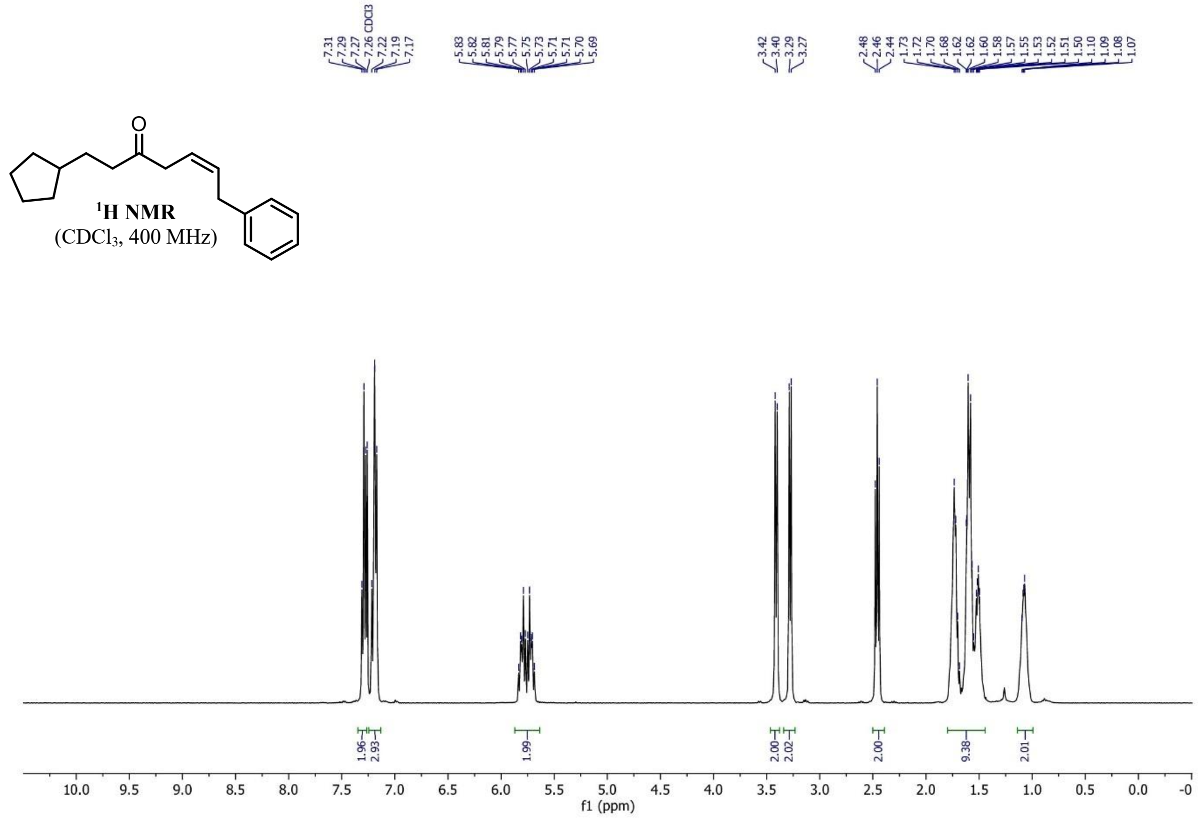

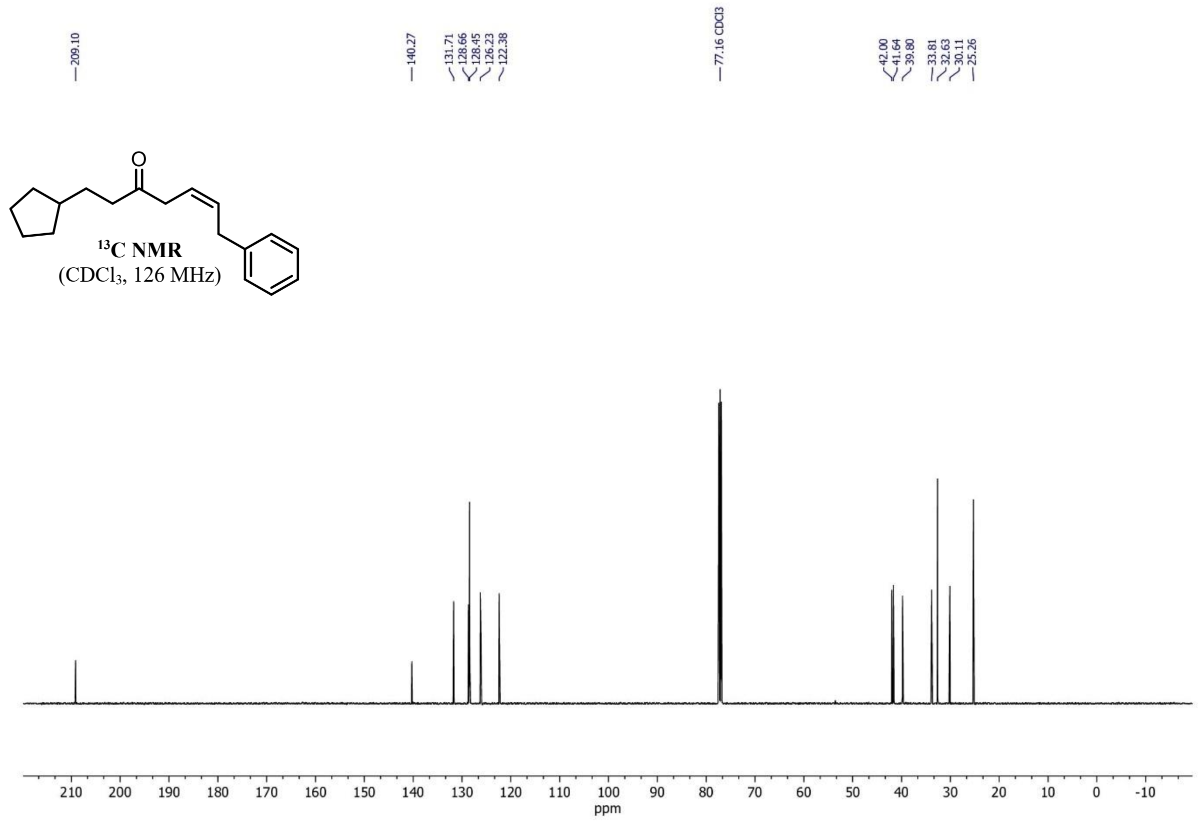

(*Z*)-1,7-Diphenylhept-5-en-3-one (4t)

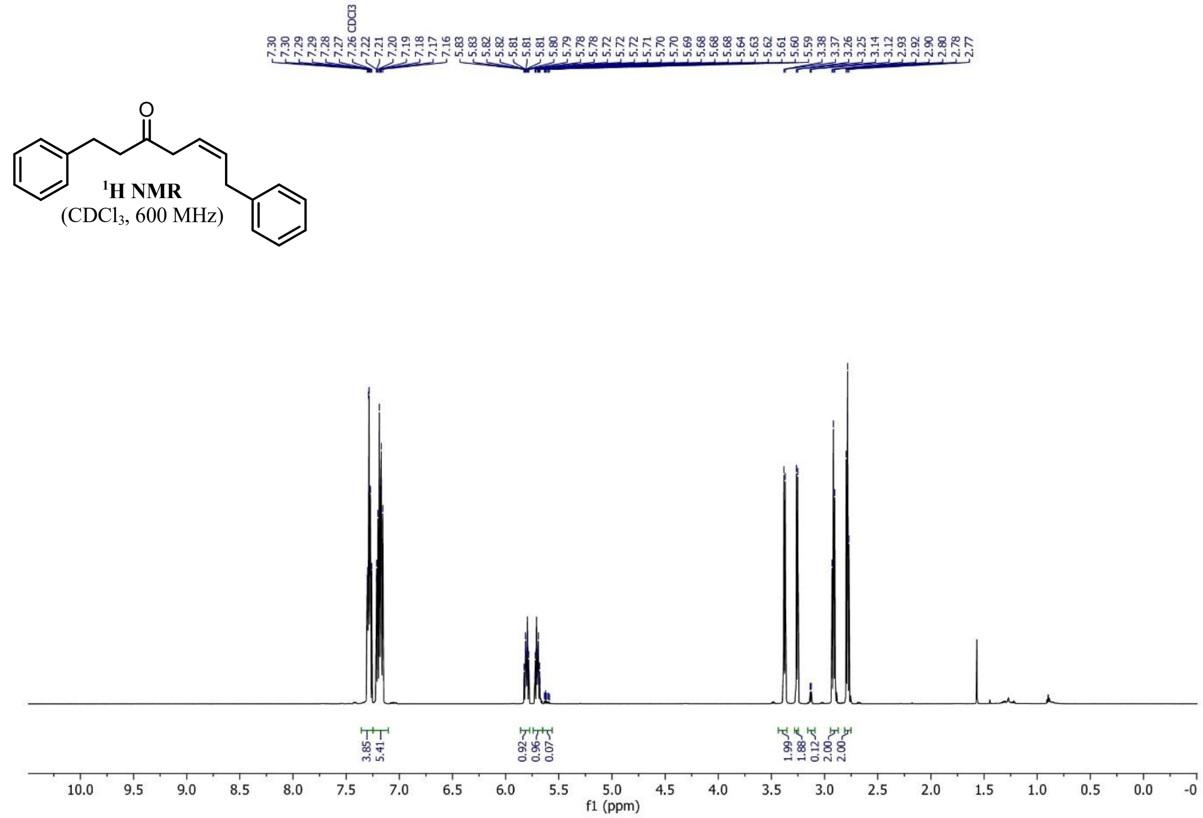

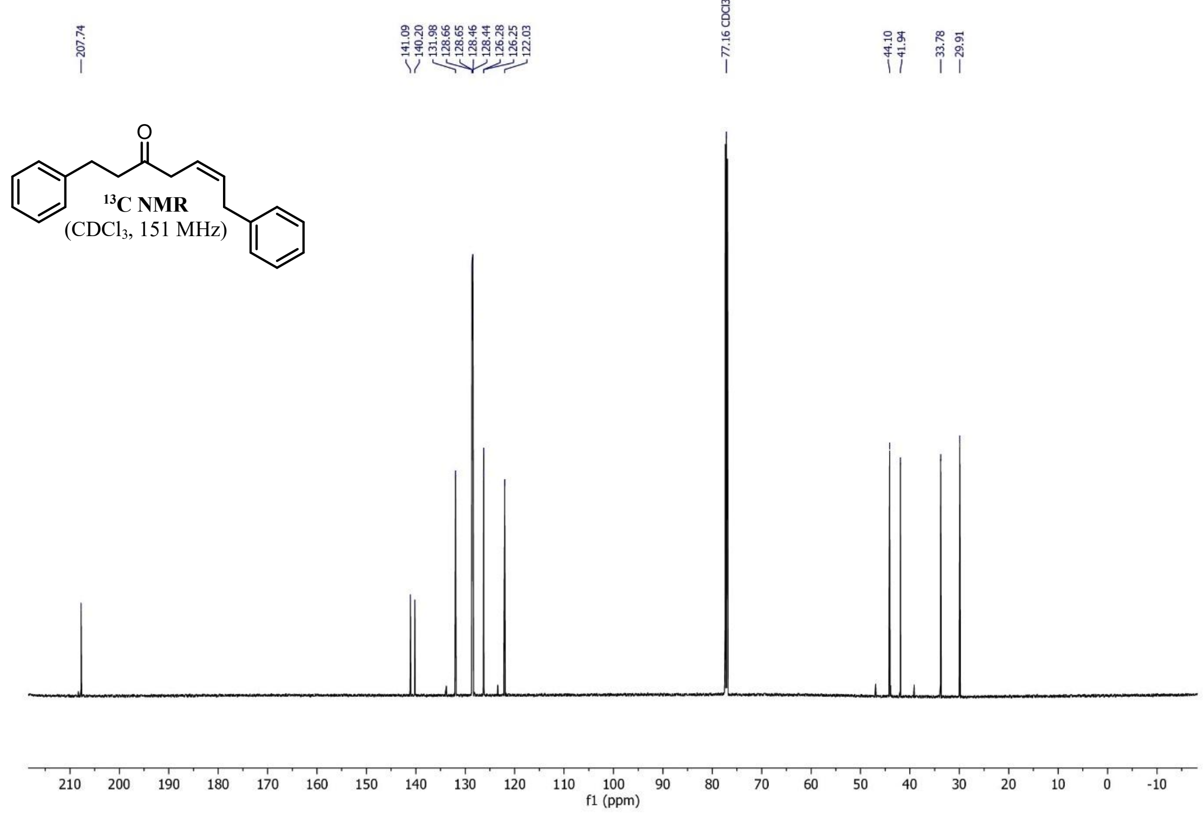

(*Z*)-10-Bromo-1-phenyldec-2-en-5-one (4u)

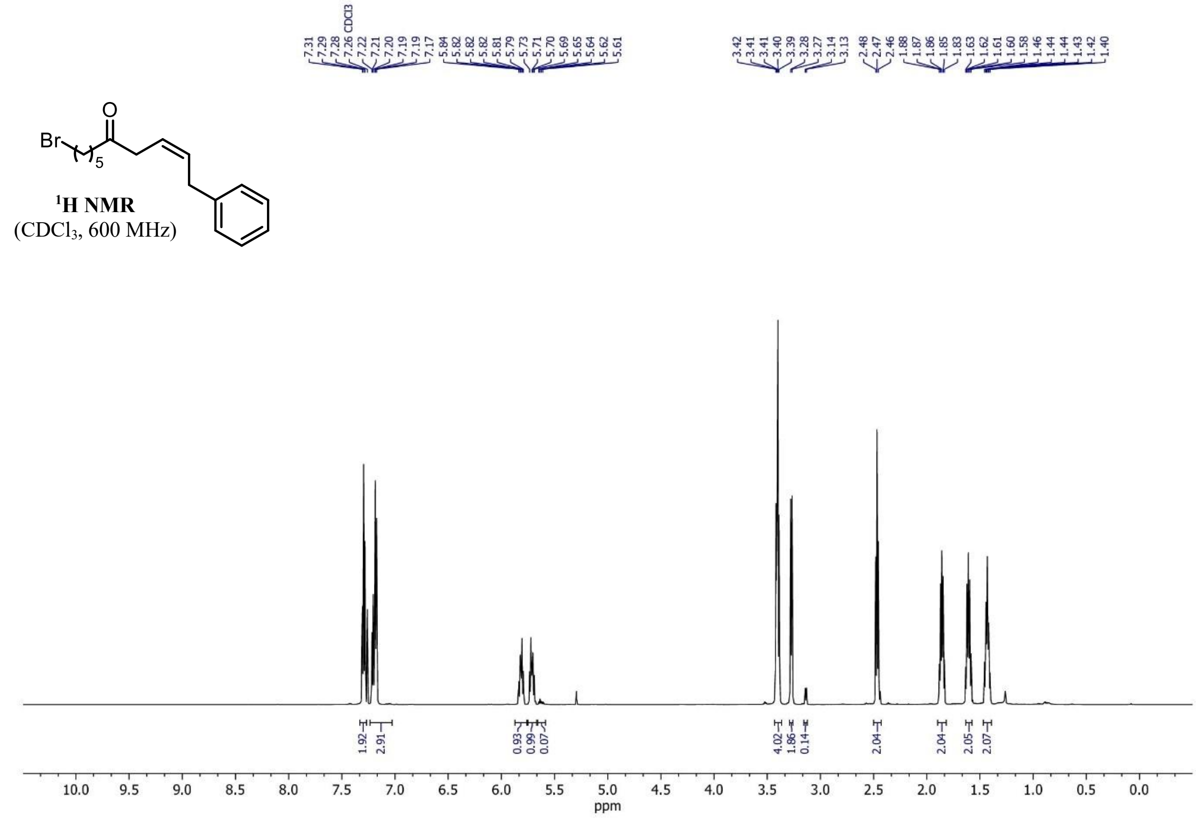

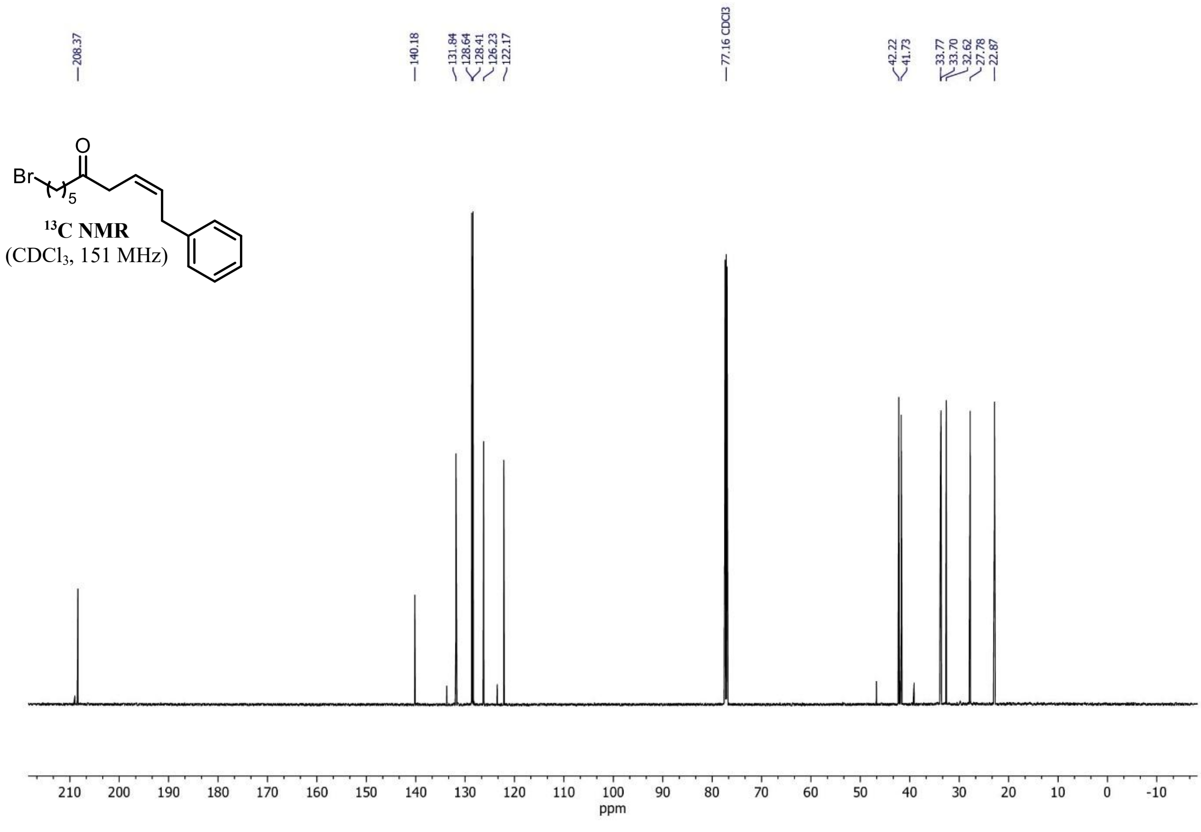

(*Z*)-1-Chloro-8-phenyloct-6-en-4-one (4v)

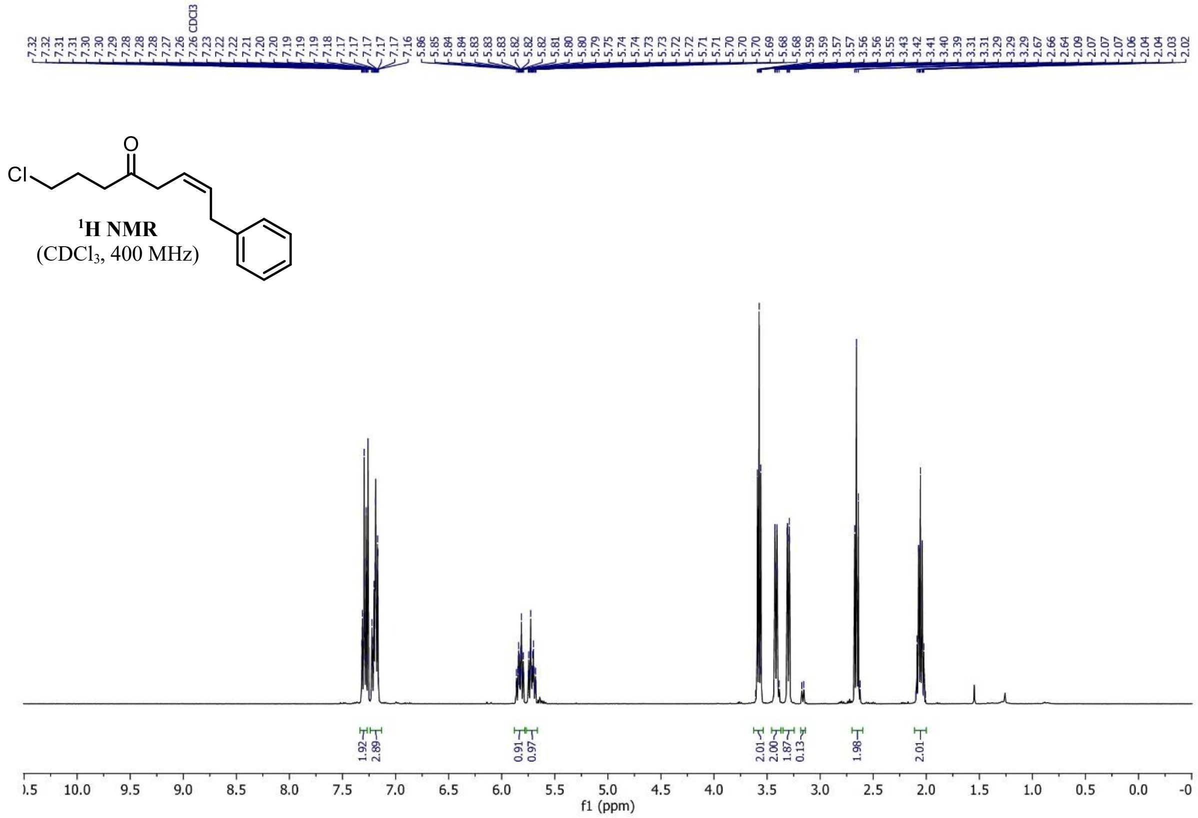

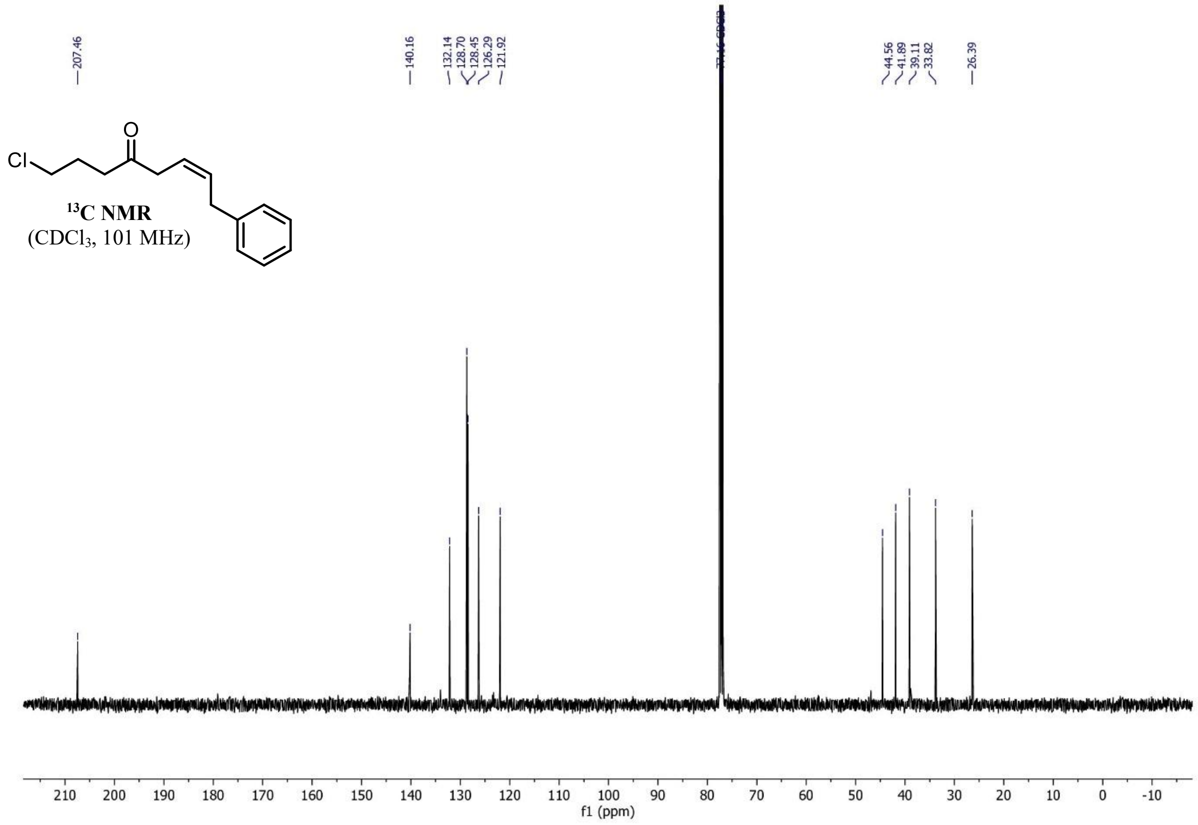

(*Z*)-1-Iodoheptadec-3-en-6-one (5a)

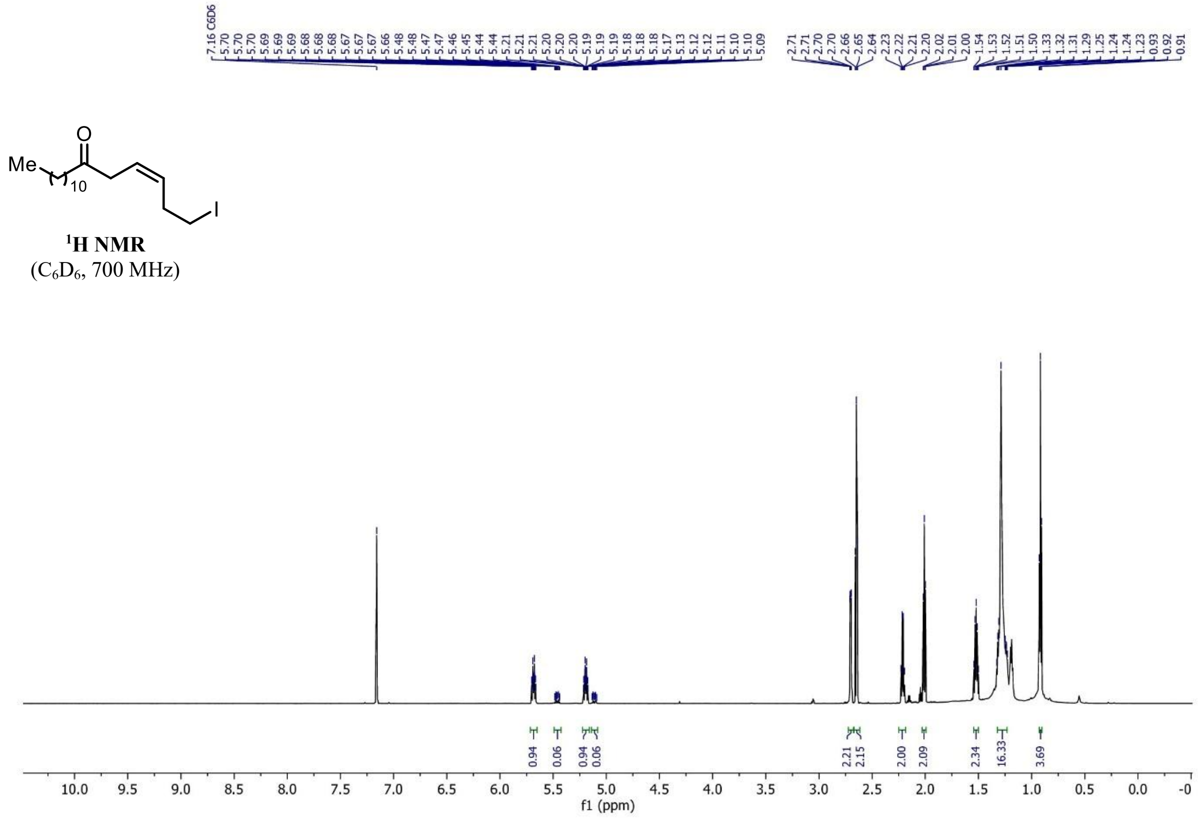

^{1}H NMR
(C_6D_6, 700 MHz)

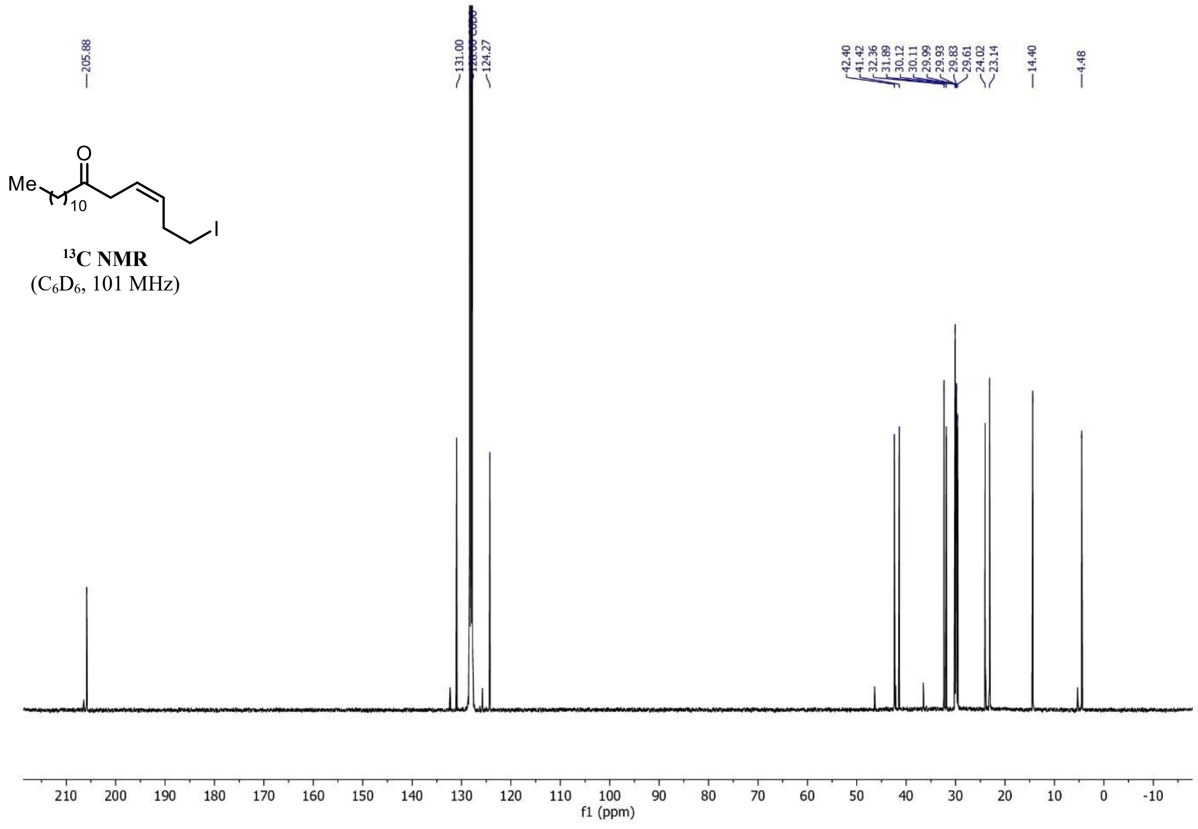

^{13}C NMR
(C_6D_6, 101 MHz)

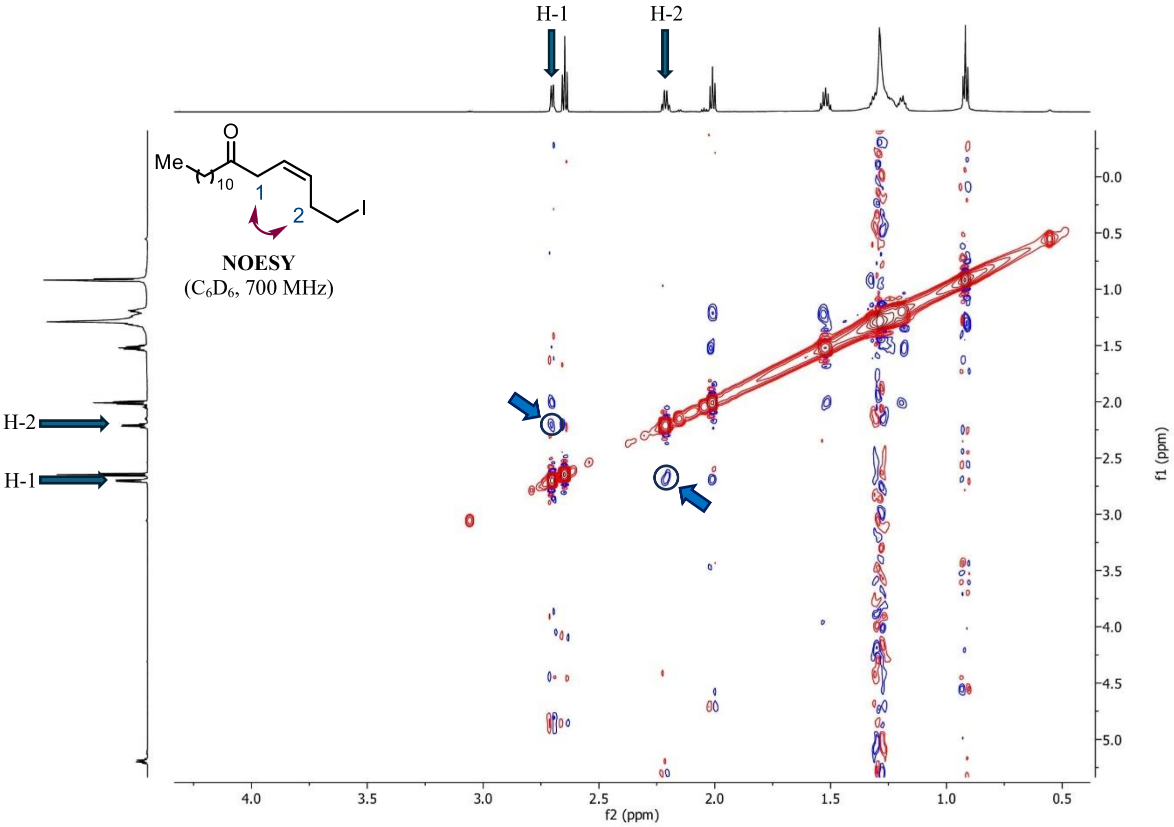

H-1
H-2
Me
O
10
1
2
I
NOESY
(C6D6, 700 MHz)
H-2
H-1
f1 (ppm)
f2 (ppm)

(*Z*)-6-Iodo-1-phenylhex-3-en-1-one (5b)

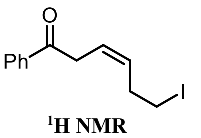

^{1}H NMR
(C_6D_6, 400 MHz)

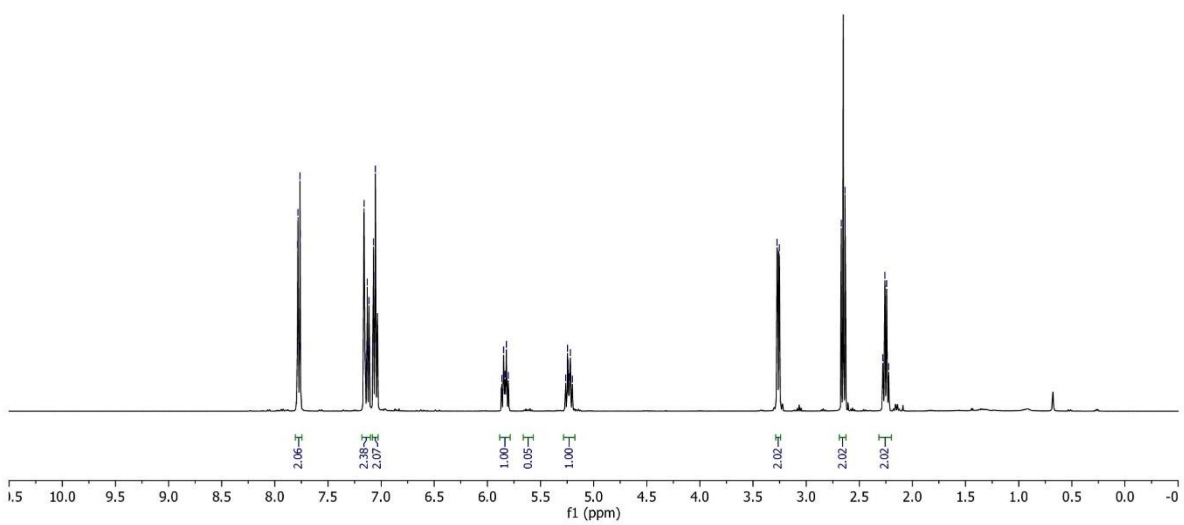

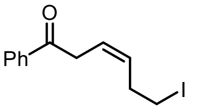

^{13}C NMR
(C_6D_6, 101 MHz)

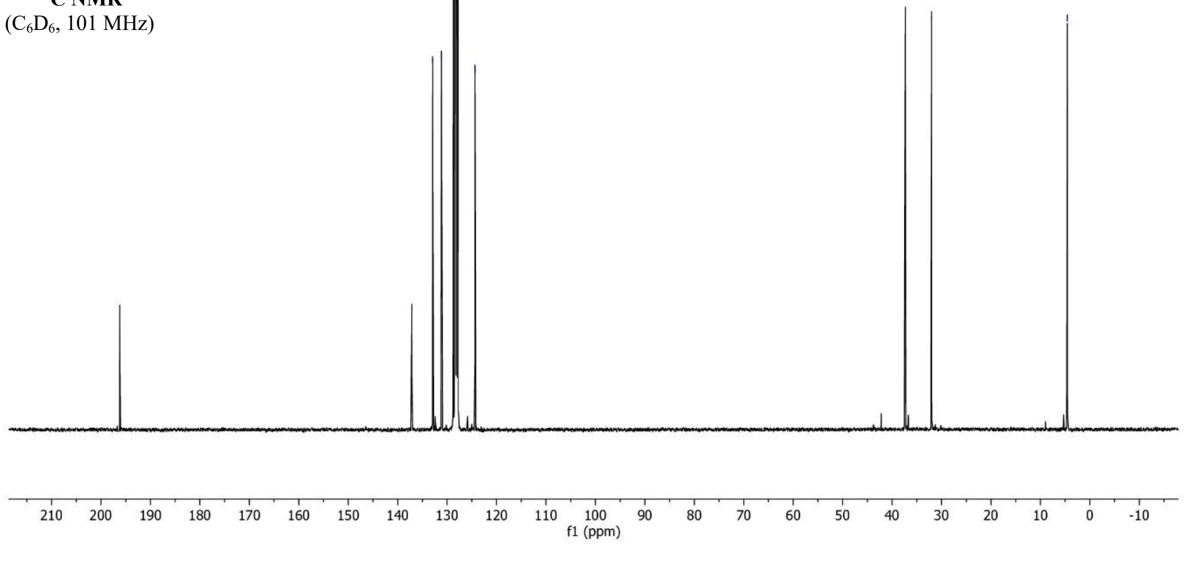

(*Z*)-1,1-Bis(4,4,5,5-tetramethyl-1,3,2-dioxaborolan-2-yl)octadec-4-en-7-one (5c)

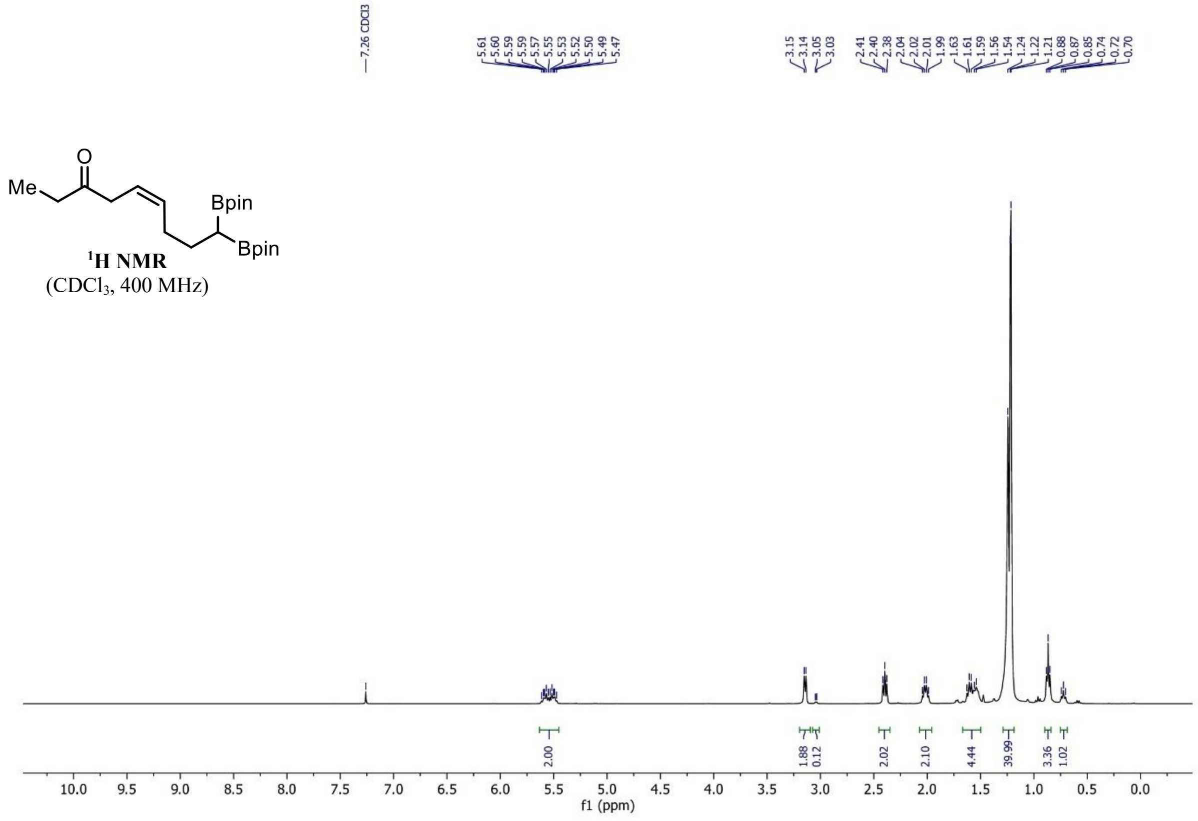

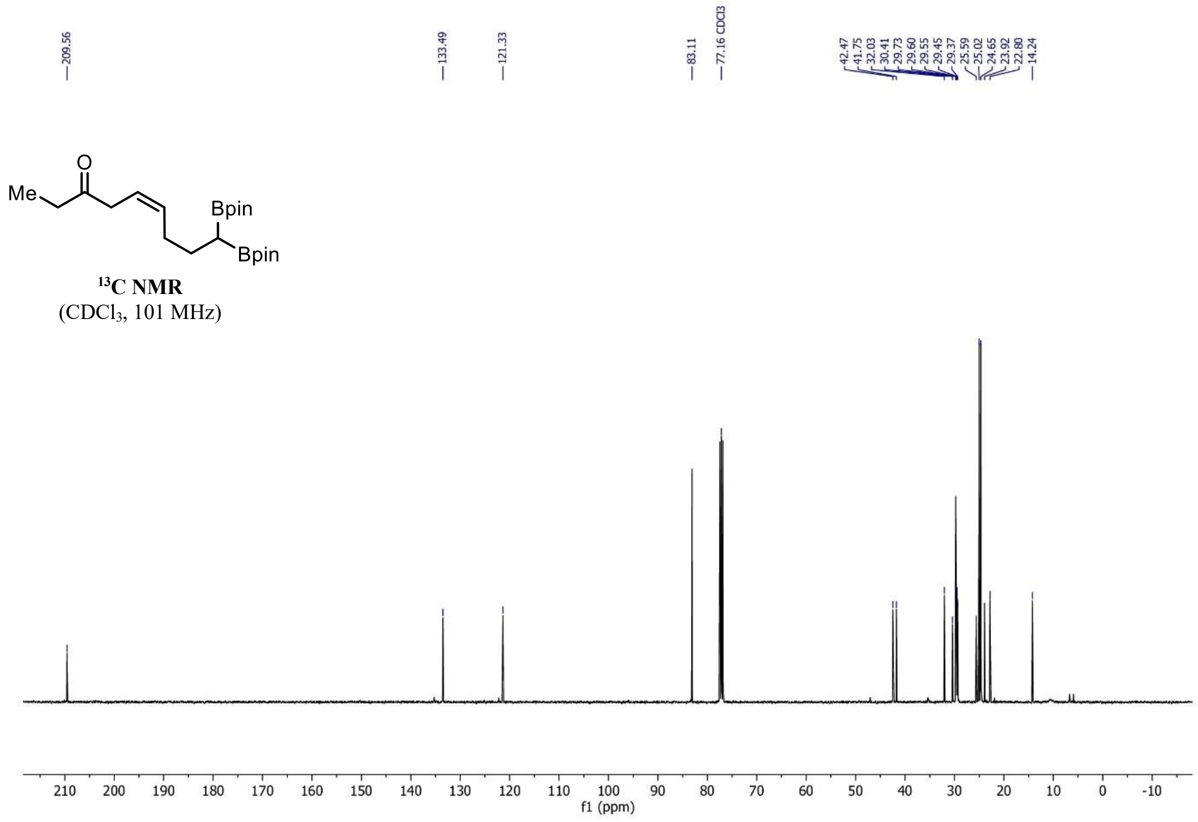

(*Z*)-*N*-Benzyl-*N*-(7-oxooctadec-4-en-1-yl)-2,4,6-tris(trifluoromethyl)benzenesulfonamide (5d)

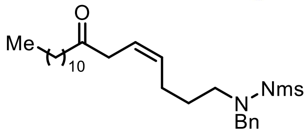

^{1}H NMR
($CDCl_3$, 400 MHz)

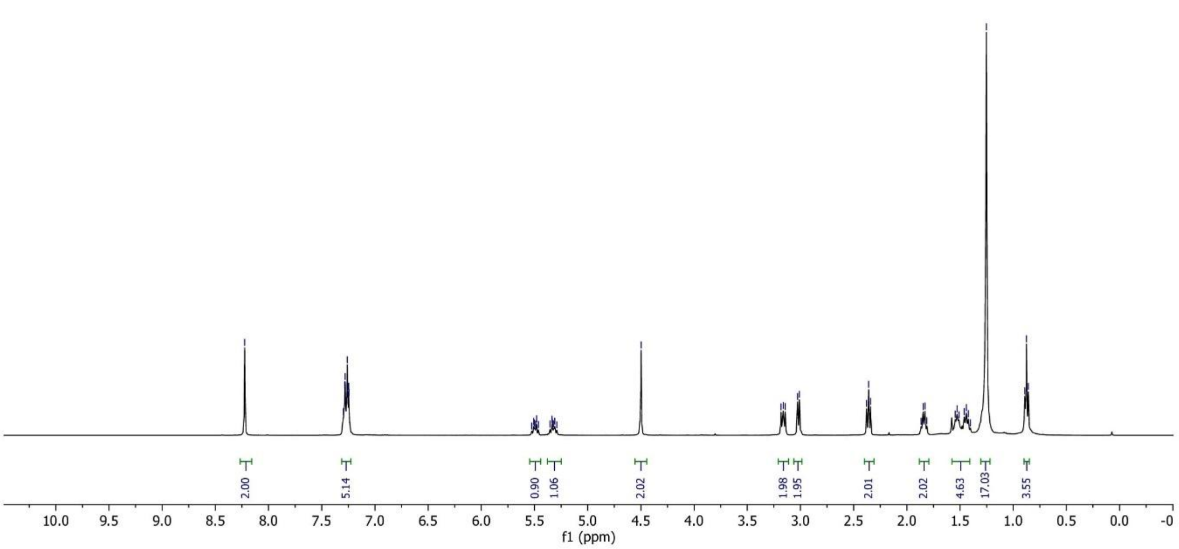

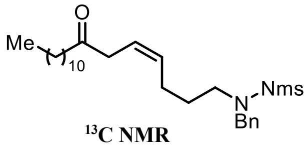

^{13}C NMR
($CDCl_3$, 101 MHz)

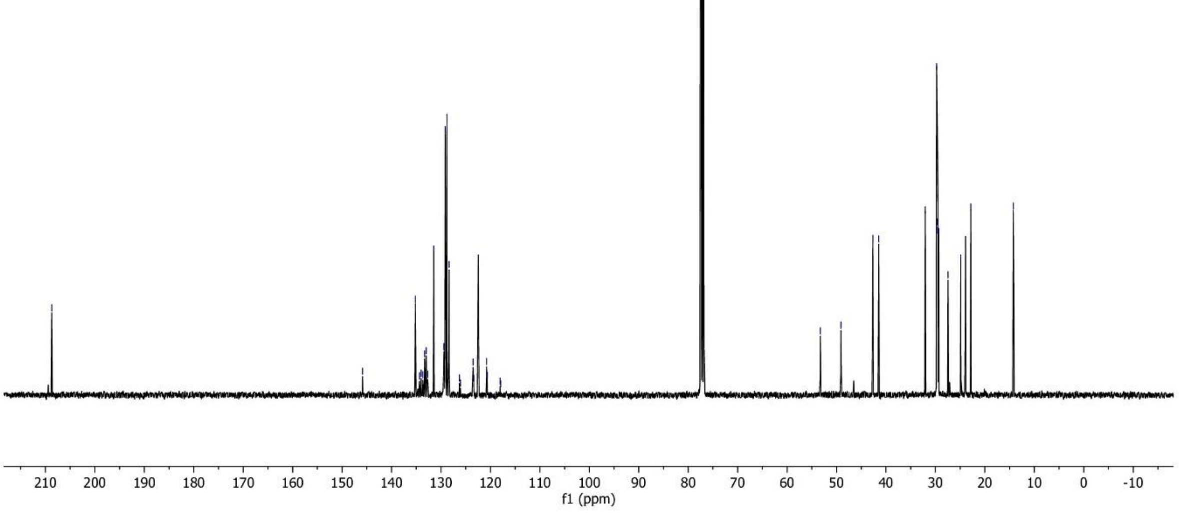

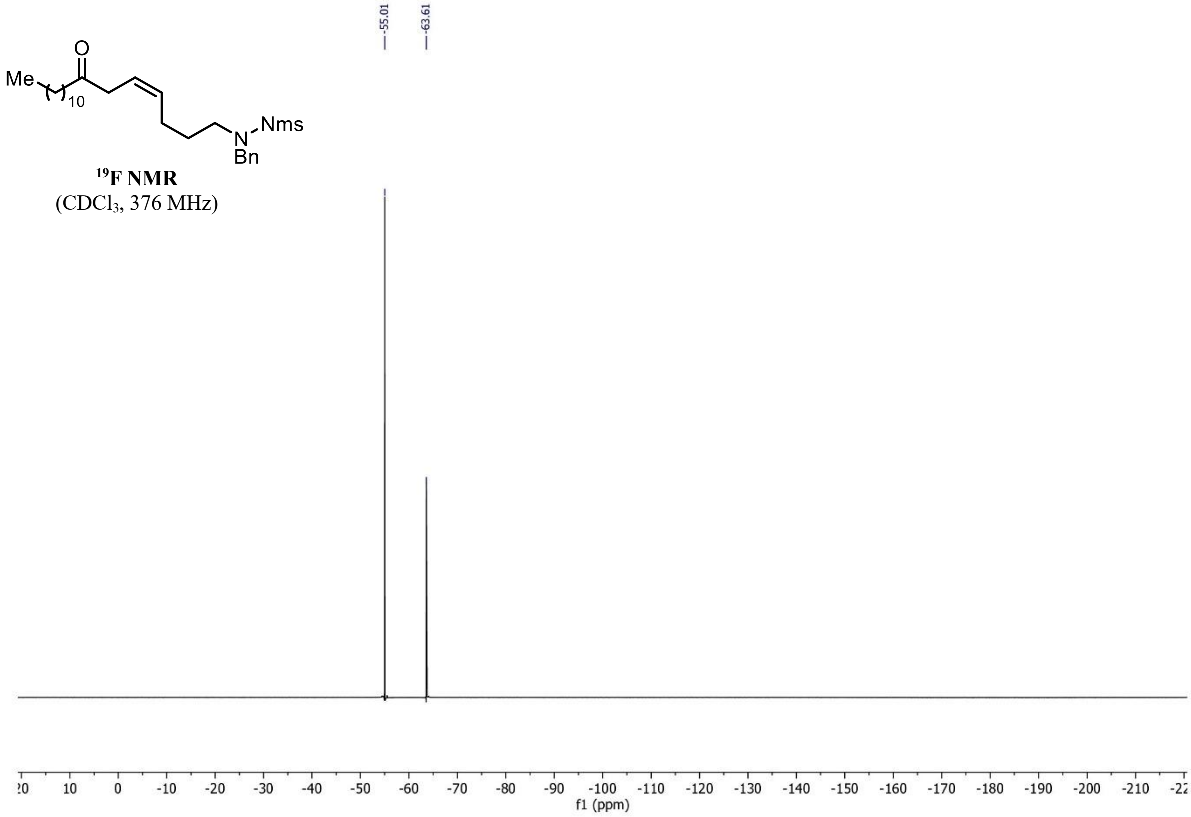

Me
10
O
N
Nms
Bn
¹⁹F NMR
(CDCl₃, 376 MHz)
-55.01
-63.61
f1 (ppm)

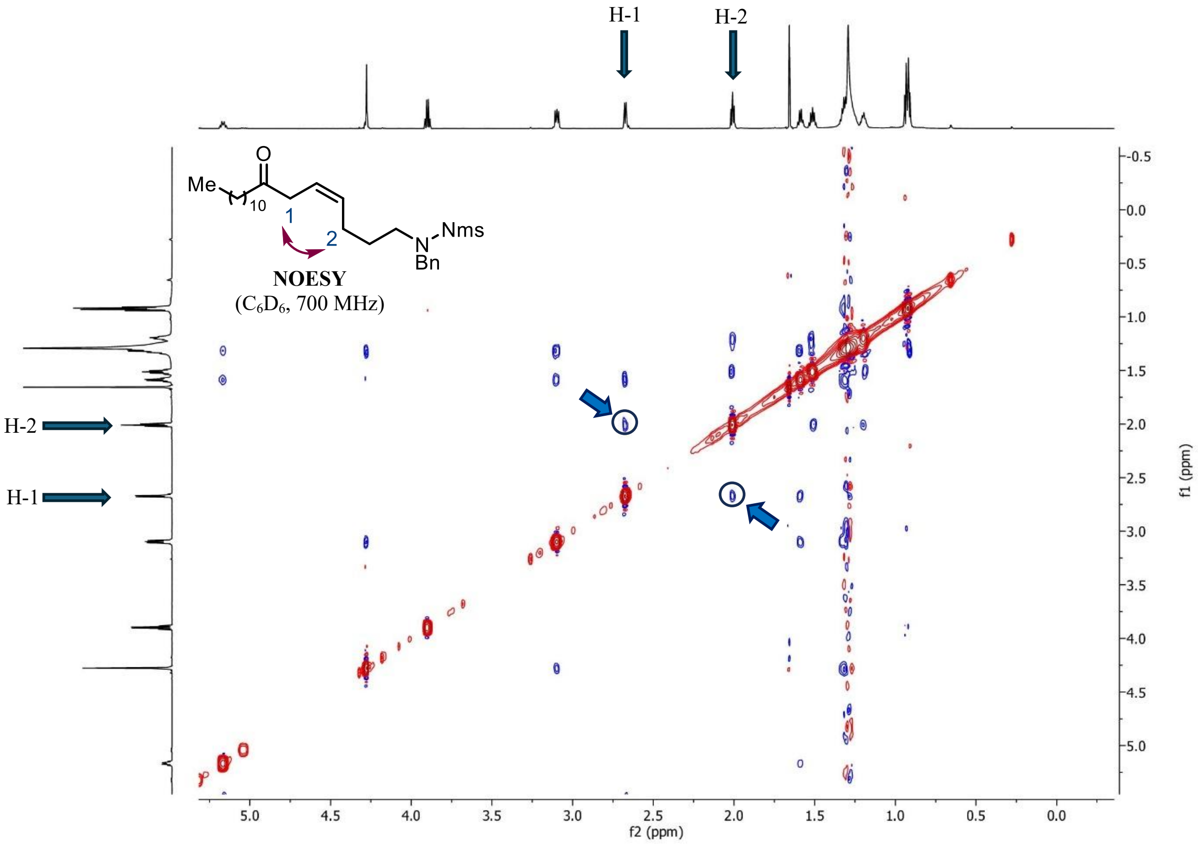

H-1
H-2
Me
10
O
1
2
N
Nms
Bn
NOESY
(C₆D₆, 700 MHz)
H-2
H-1
f2 (ppm)
f1 (ppm)

(*Z*)-2-(7-Oxooctadec-4-en-1-yl)isoindoline-1,3-dione (5e)

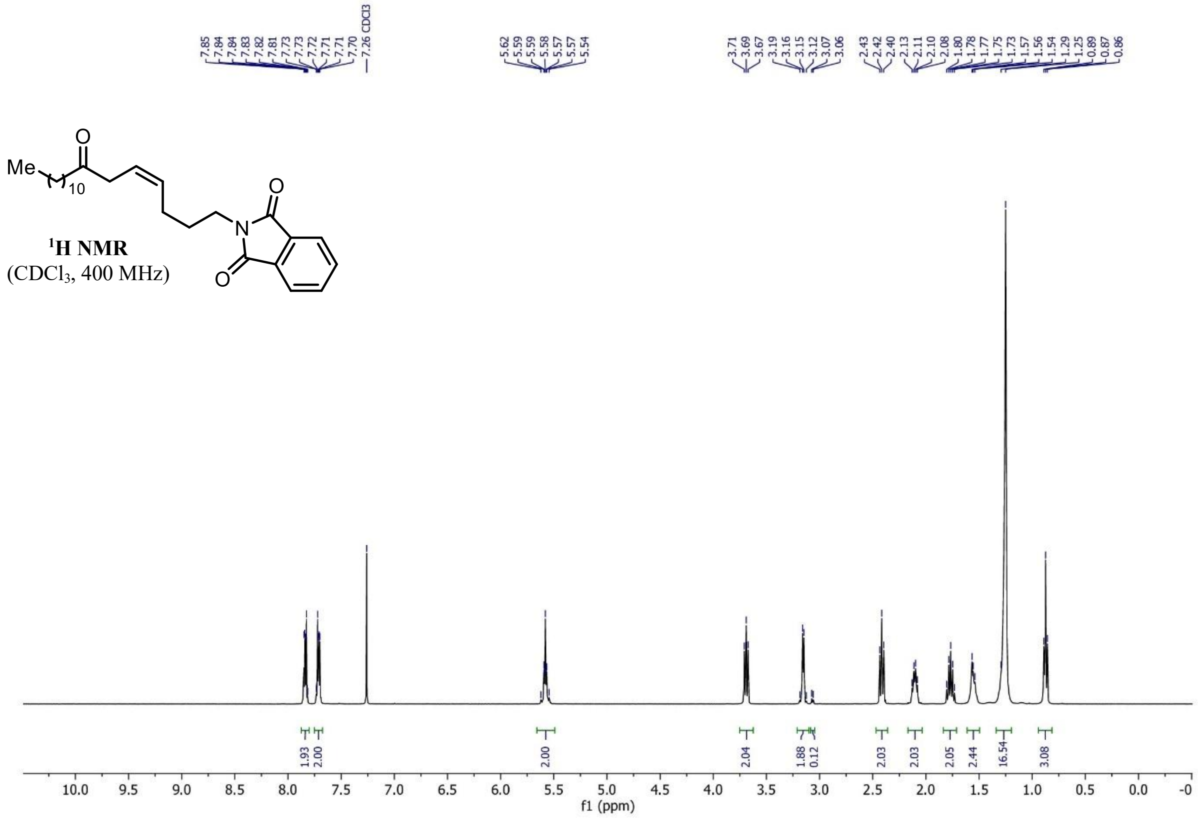

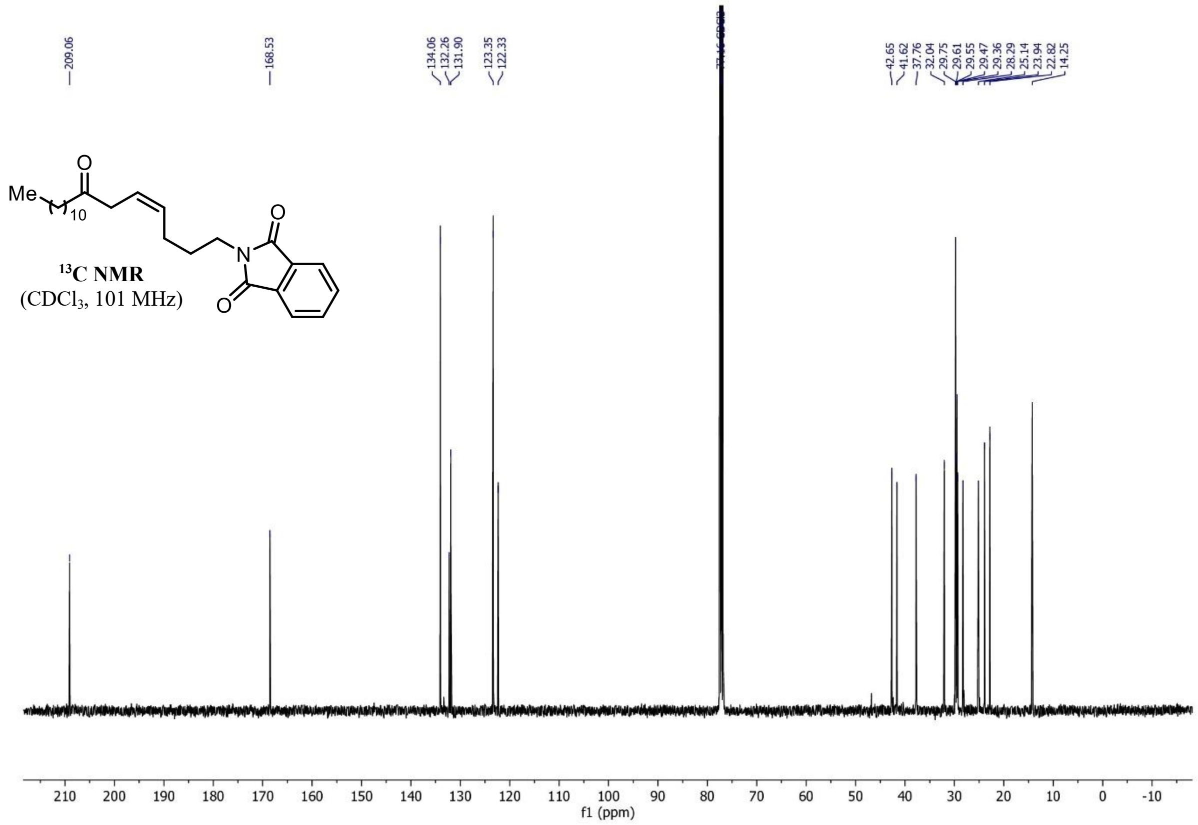

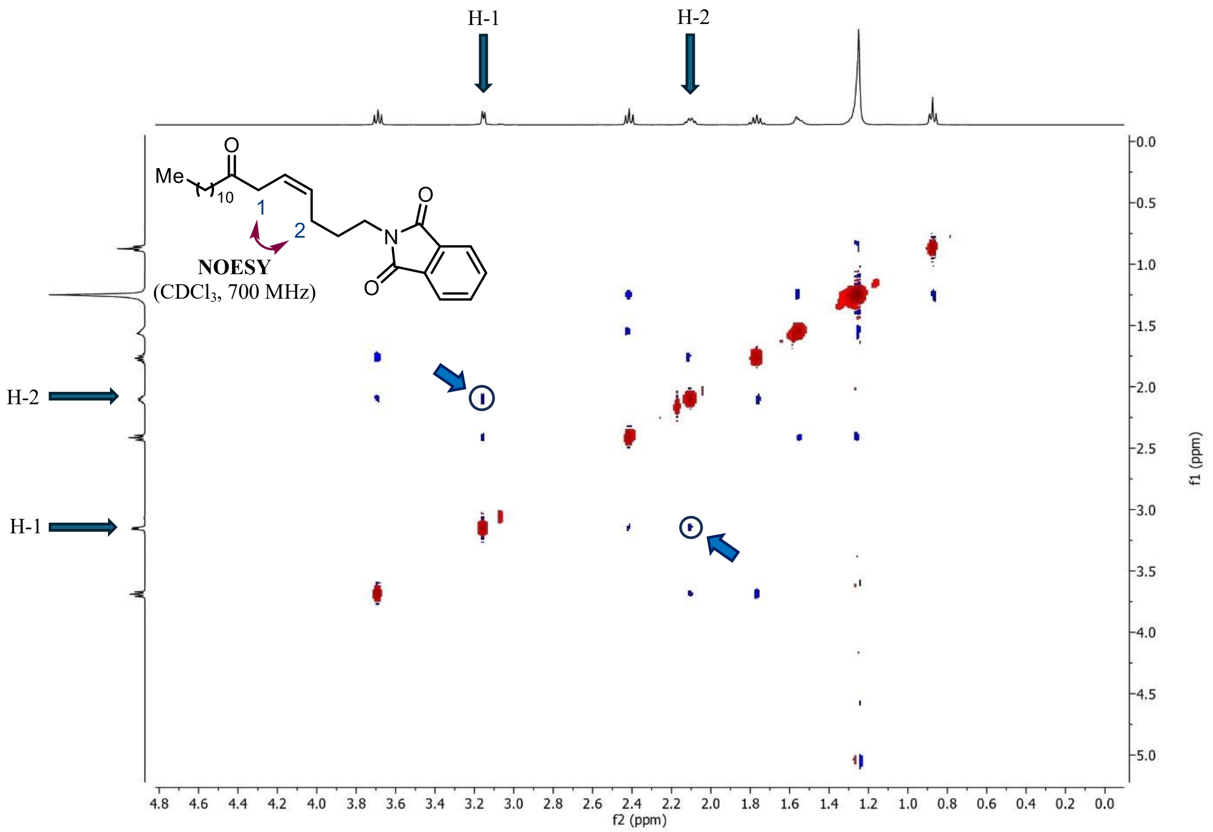

H-1
H-2
Me
10
1
2
O
N
NOESY
(CDCl$_3$, 700 MHz)
f1 (ppm)
f2 (ppm)

(*Z*)-7-Oxooctadec-4-en-1-yl 2,2,2-trifluoroacetate (5f)

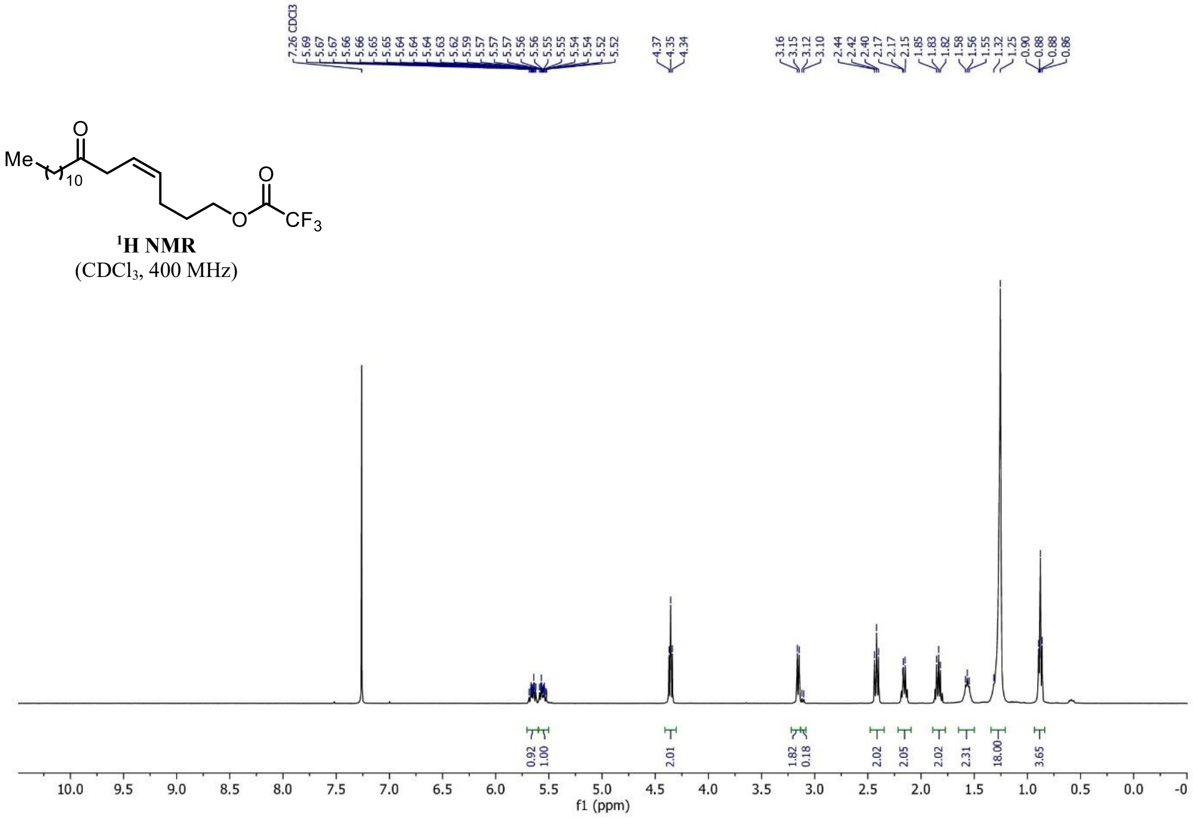

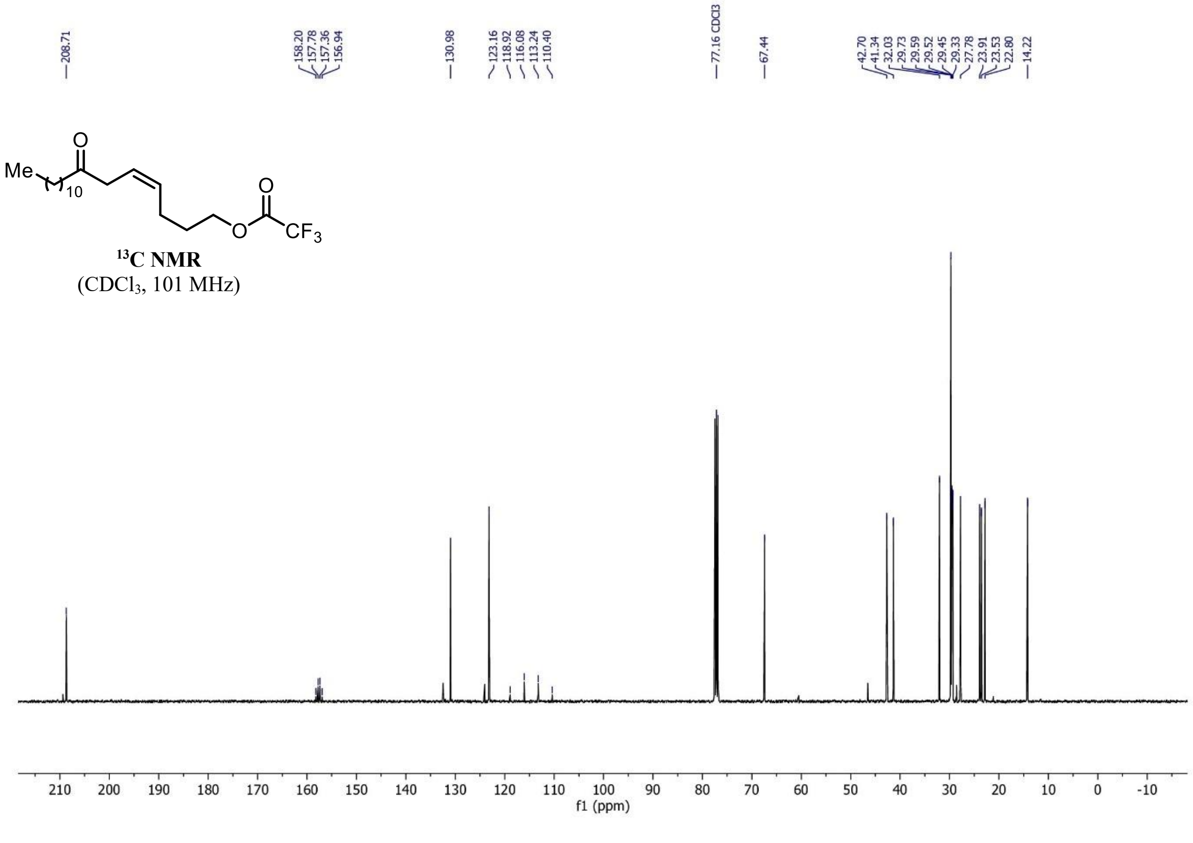

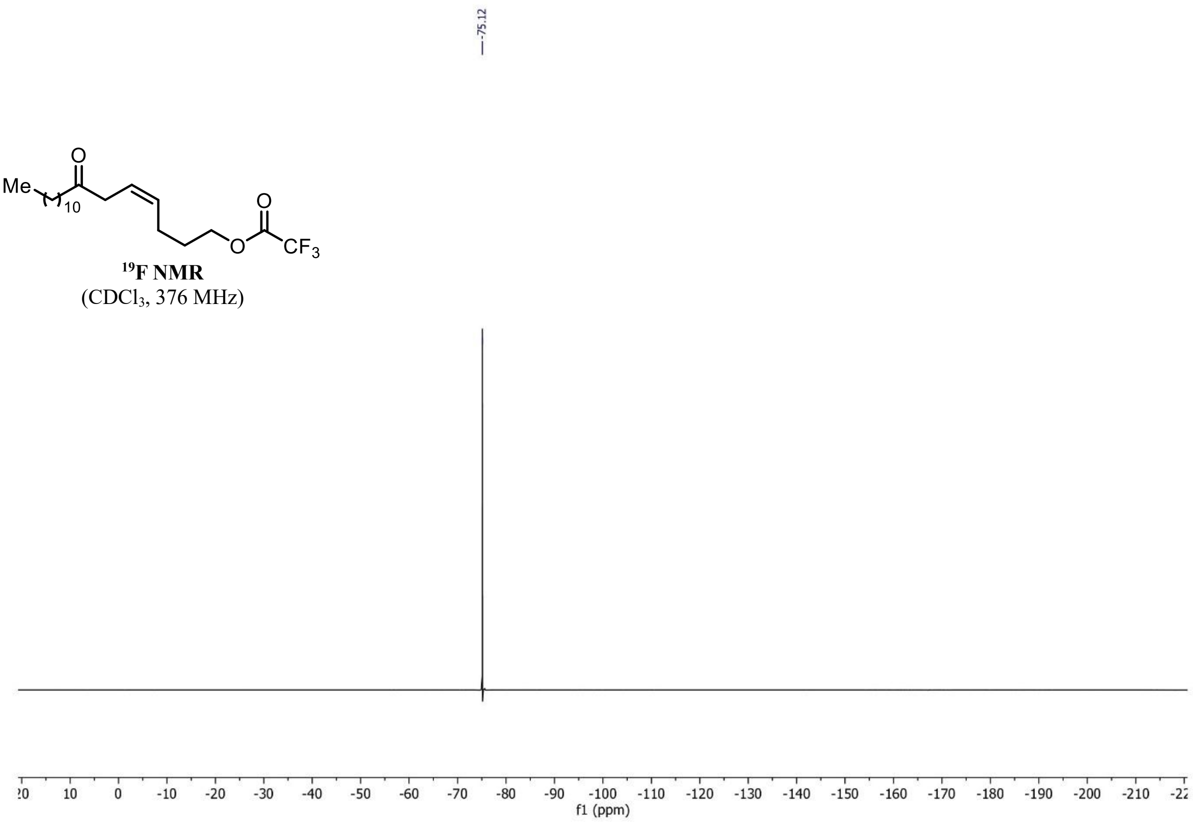

-75.12
Me
10
O
O
O
CF3
19F NMR
(CDCl3, 376 MHz)
f1 (ppm)

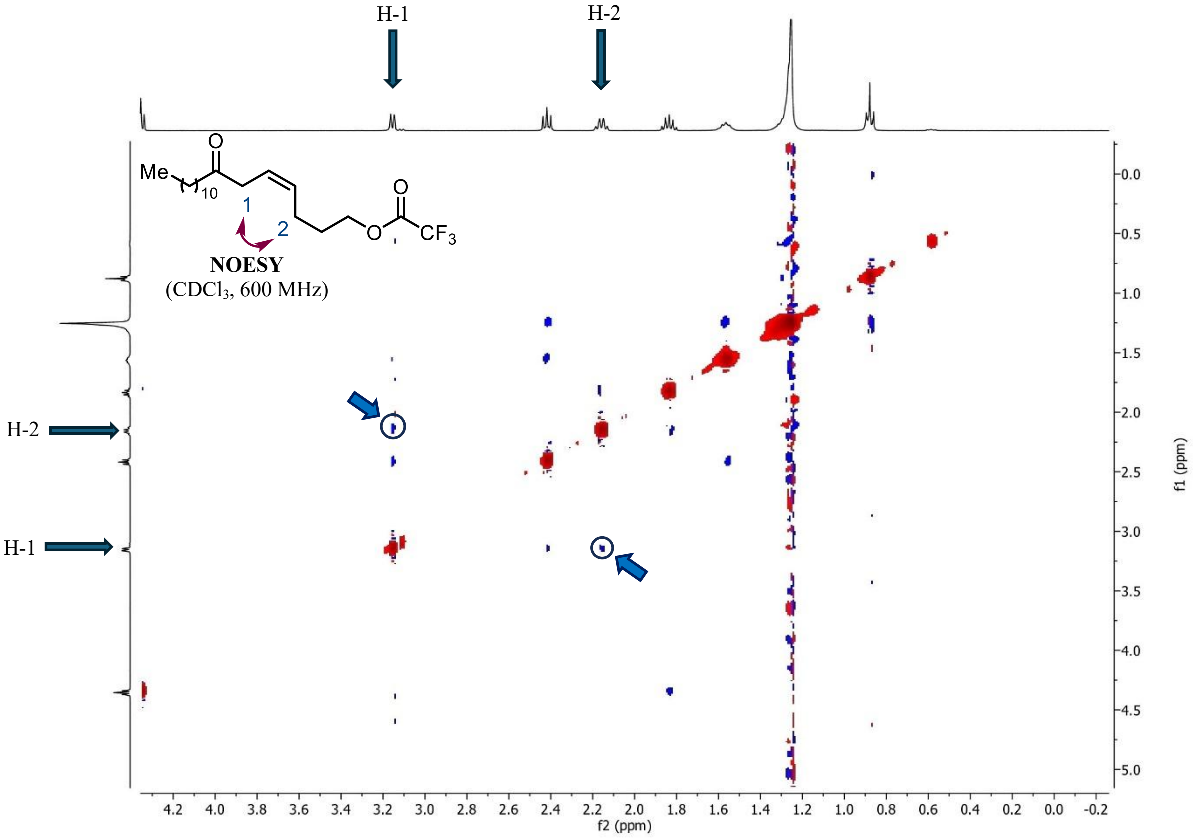

H-1
H-2
Me
10
O
O
O
CF3
1
2
NOESY
(CDCl3, 600 MHz)
f2 (ppm)
f1 (ppm)

(*Z*)-7-Oxooctadec-4-en-1-yl 4-methylbenzenesulfonate (5g)

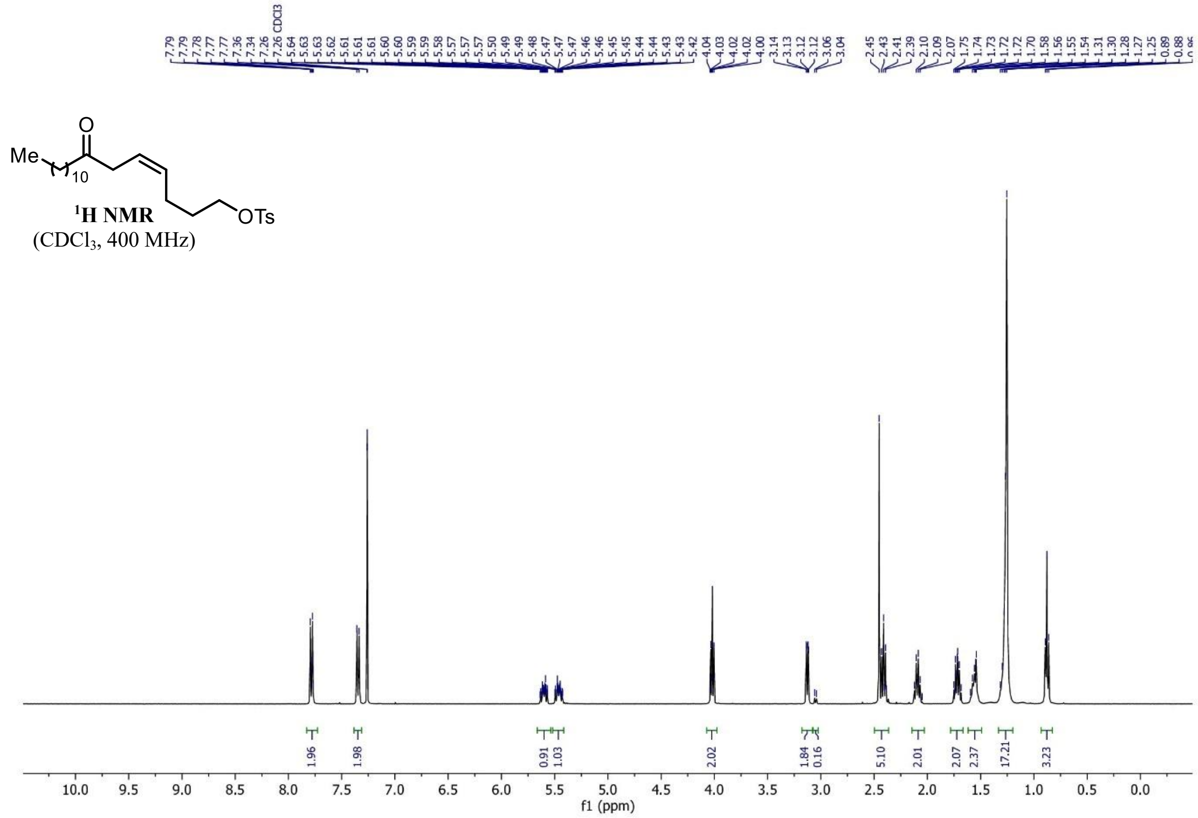

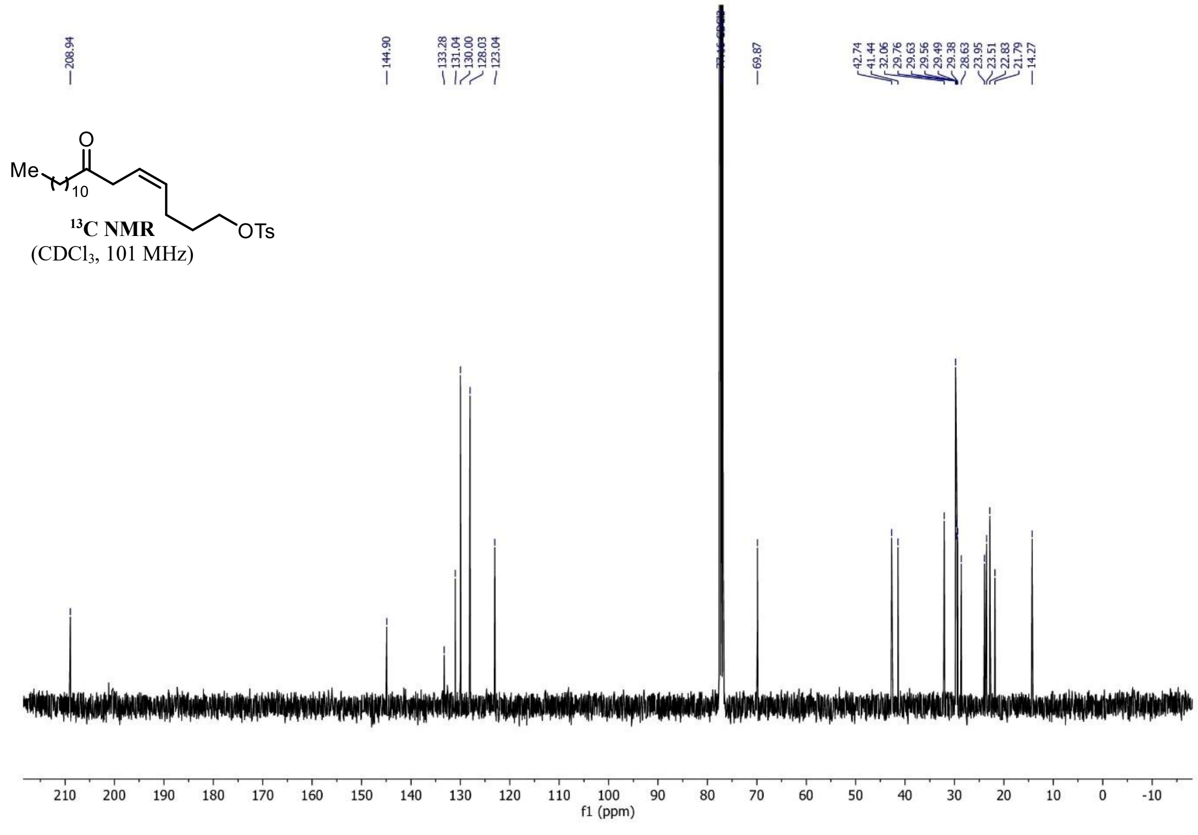

(±)-3-((2*S*,3*R*)-3-(2-oxotridecyl)oxiran-2-yl)propyl 4-methylbenzenesulfonate (6)

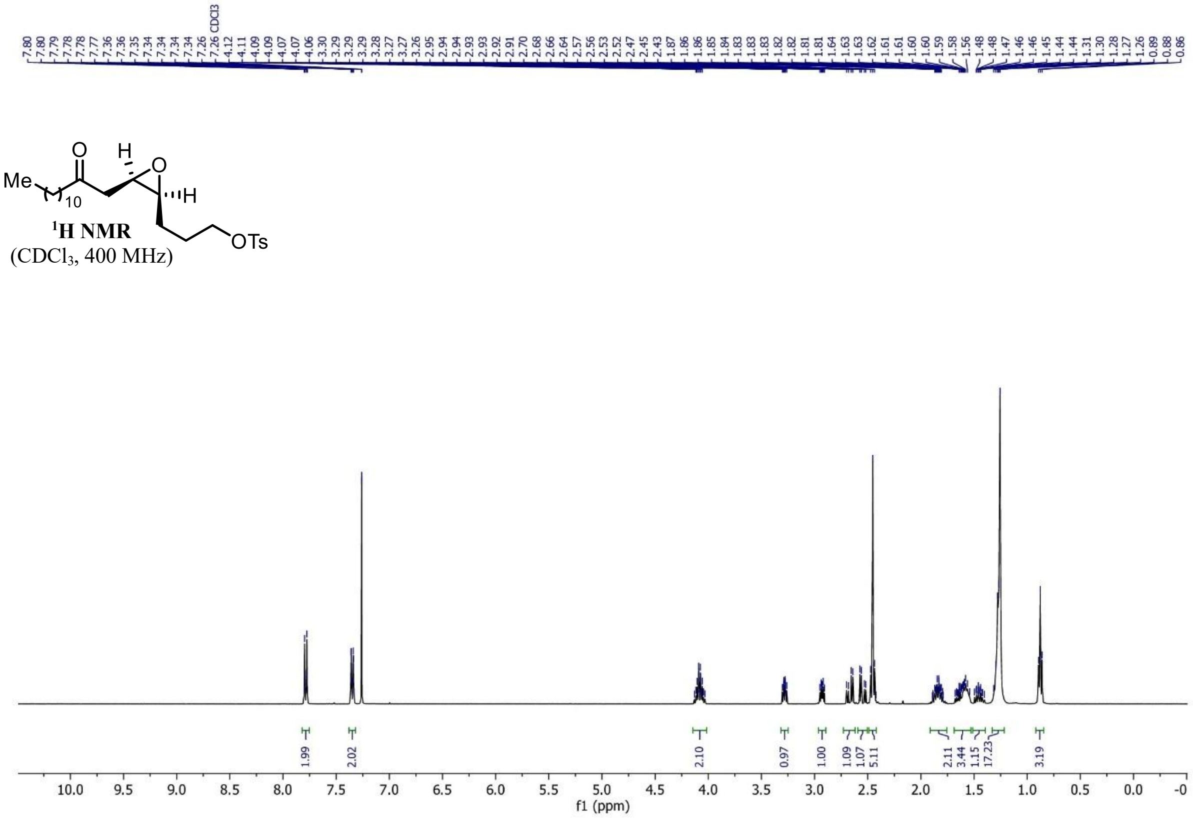

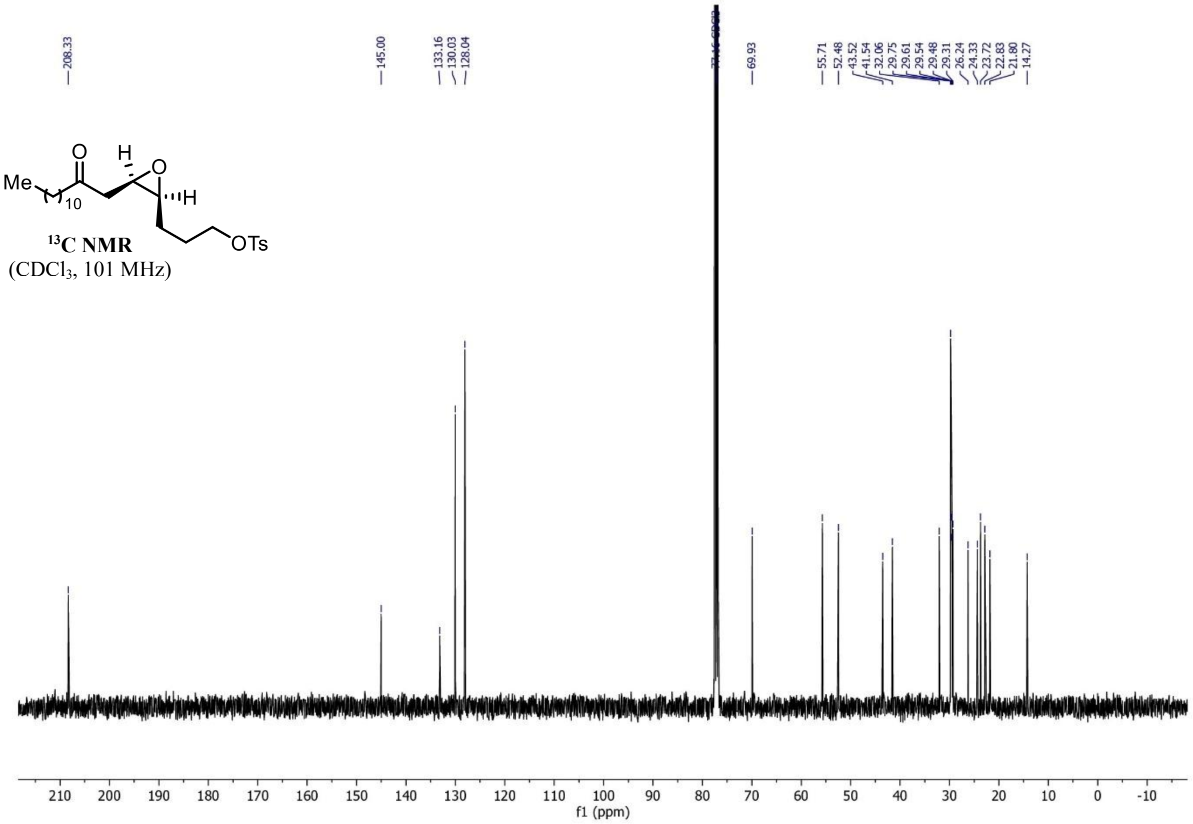

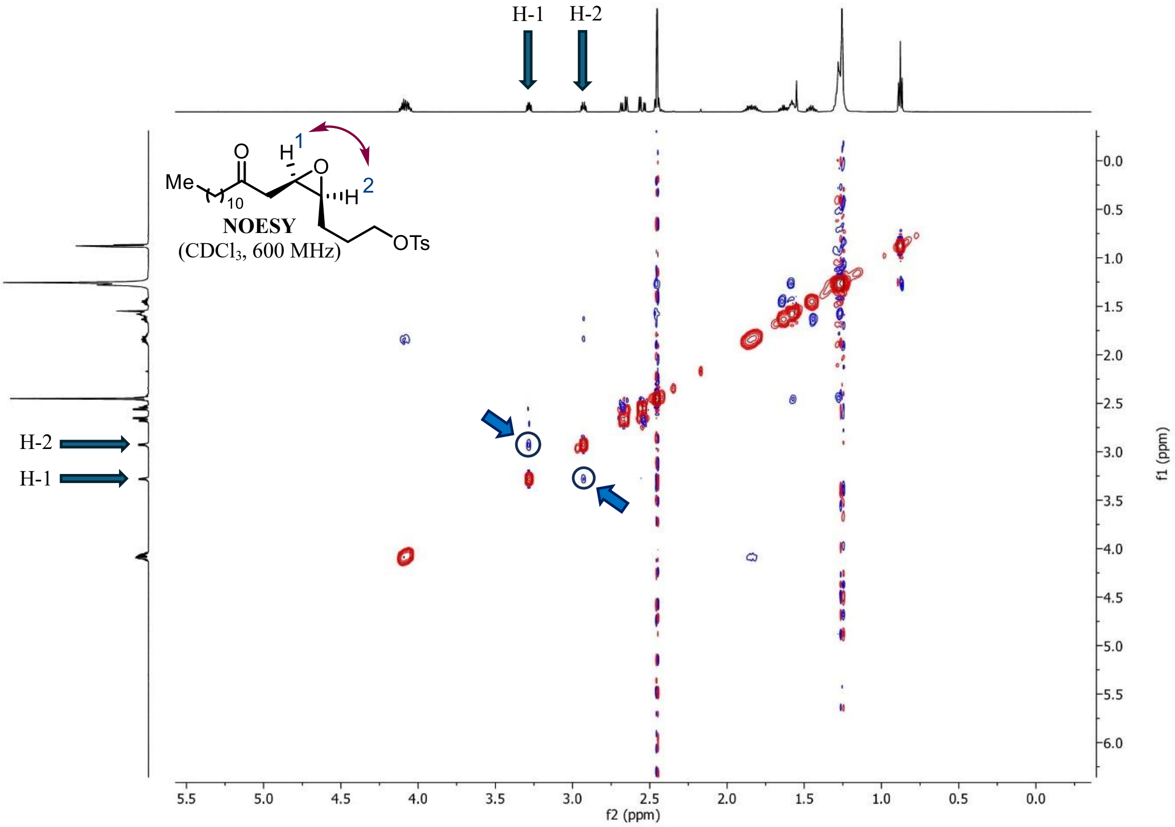

H-1
H-2
Me
10
O
H 1
H 2
OTs
NOESY
(CDCl3, 600 MHz)
f2 (ppm)
f1 (ppm)

(*Z*)-Dimethyl (7-oxooctadec-4-en-1-yl) phosphate (5h)

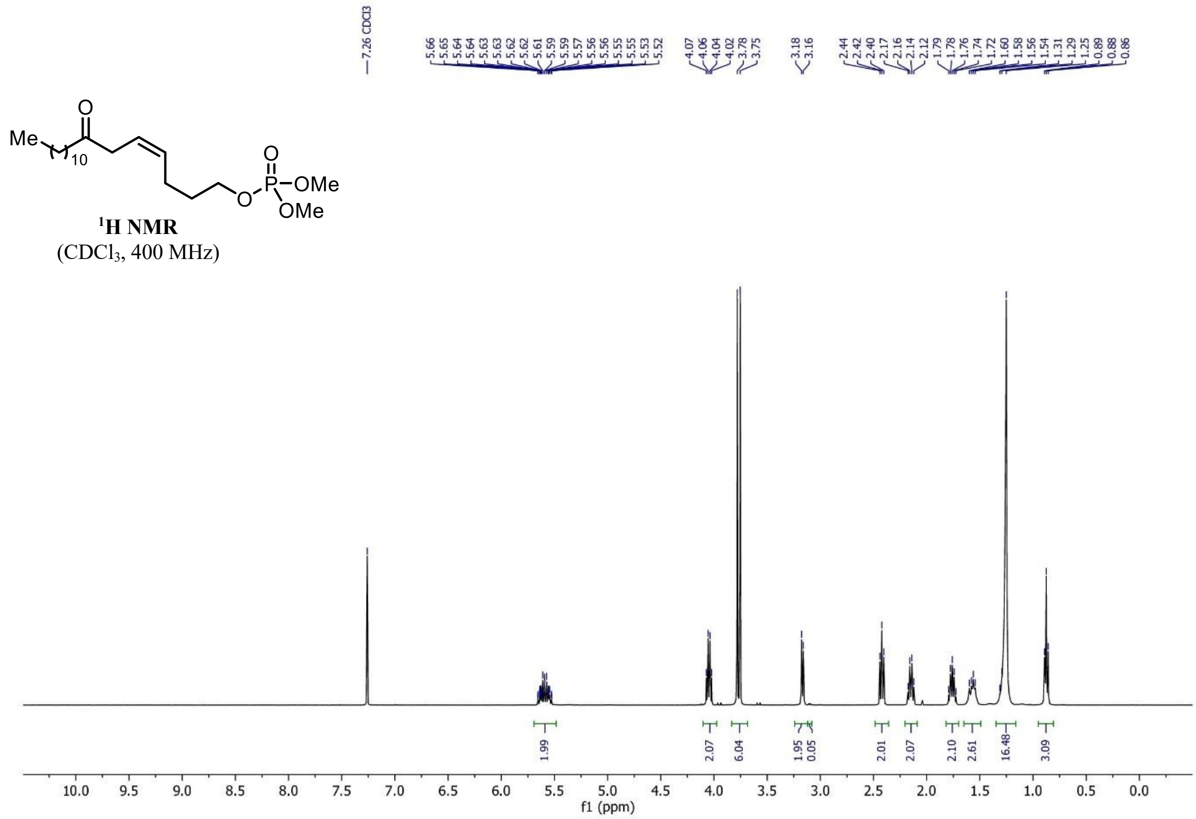

¹H NMR
($CDCl_3$, 400 MHz)

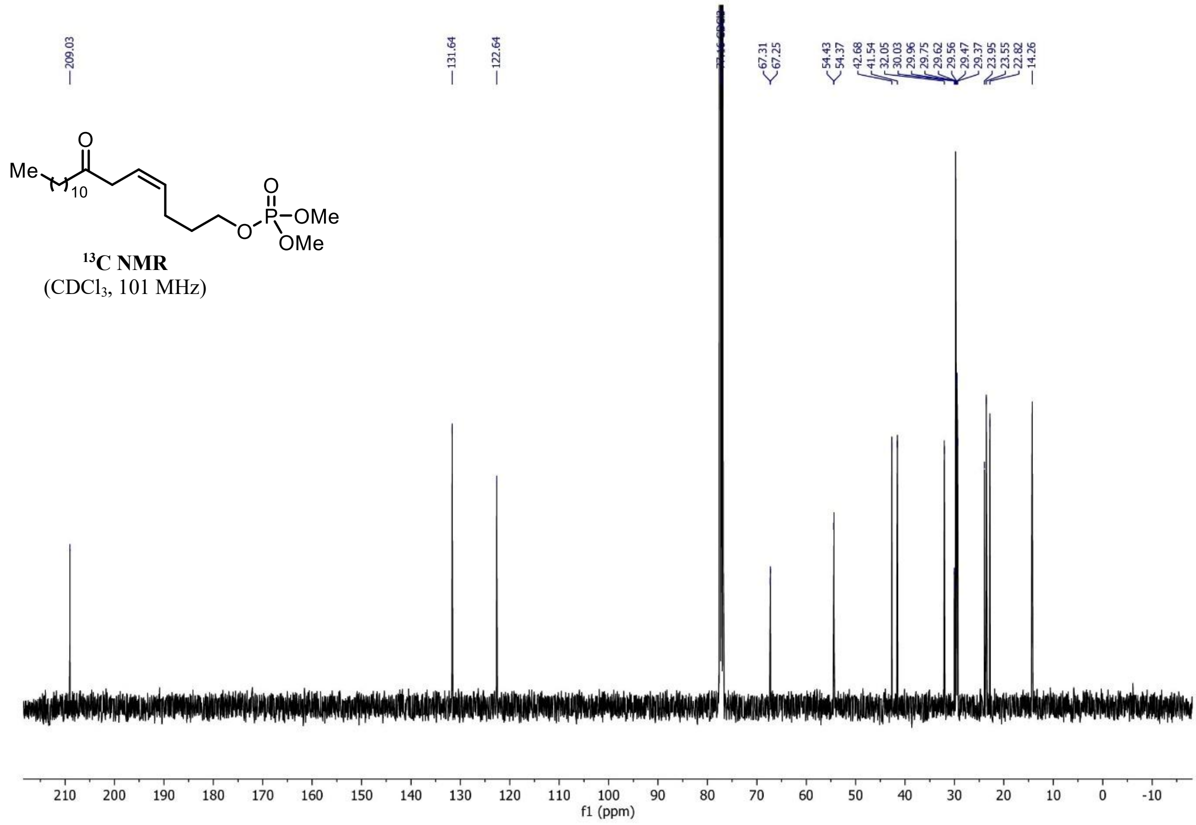

¹³C NMR
($CDCl_3$, 101 MHz)

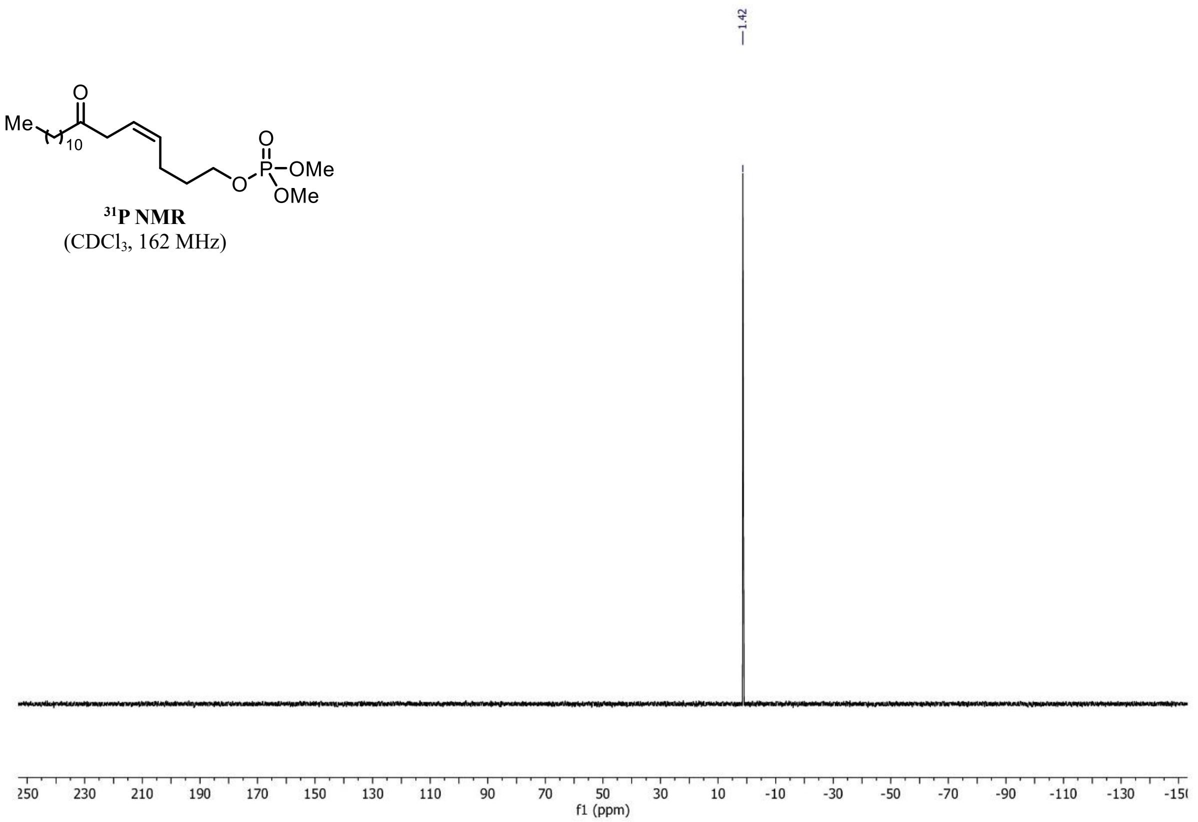

Me
10
O
O
P
OMe
OMe
O
³¹P NMR
(CDCl3, 162 MHz)
1.42
f1 (ppm)

Methyl (*Z*)-4-((7-oxooctadec-4-en-1-yl)oxy)benzoate (5i)

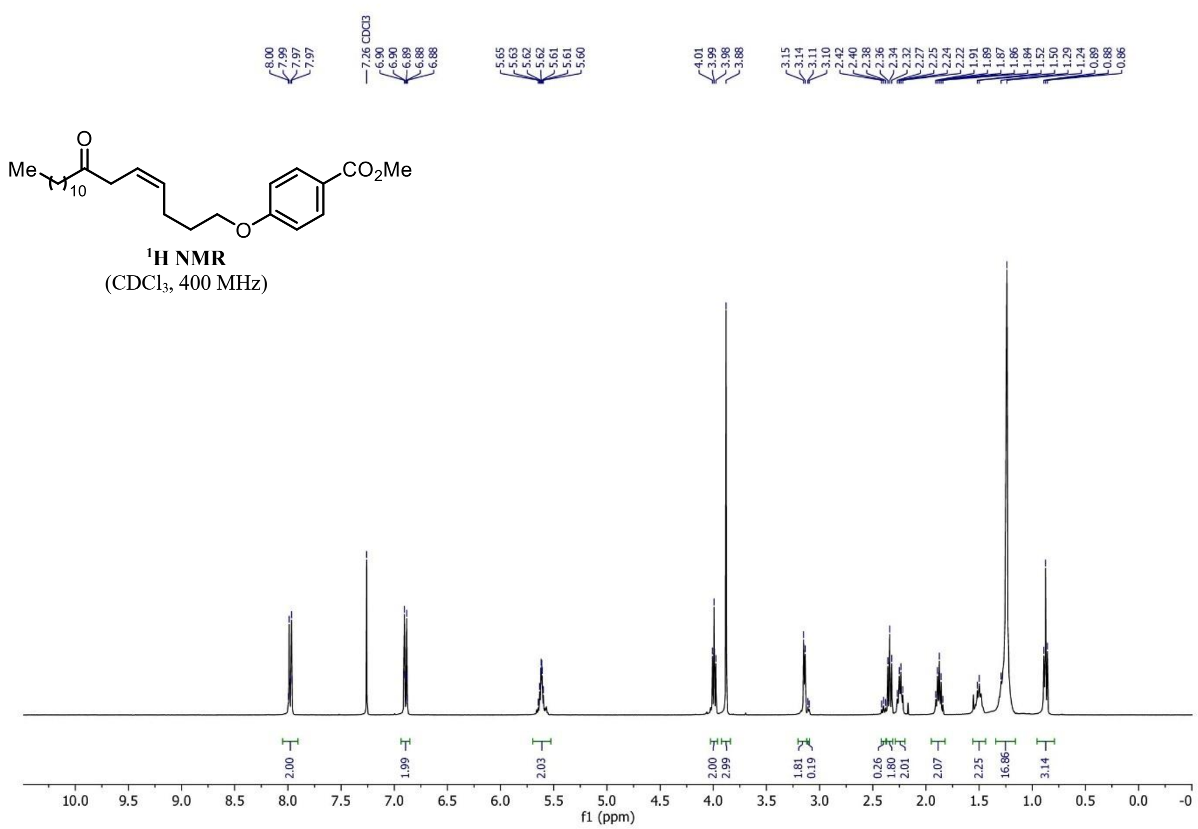

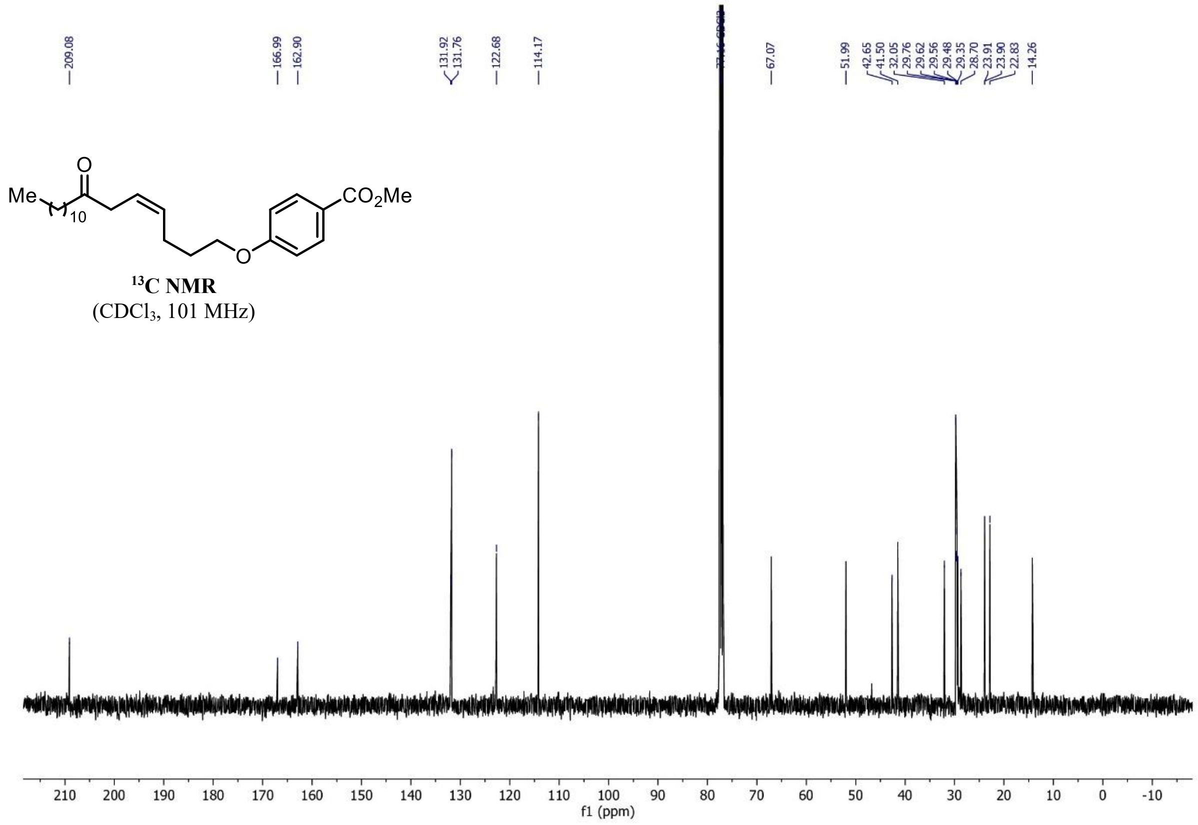

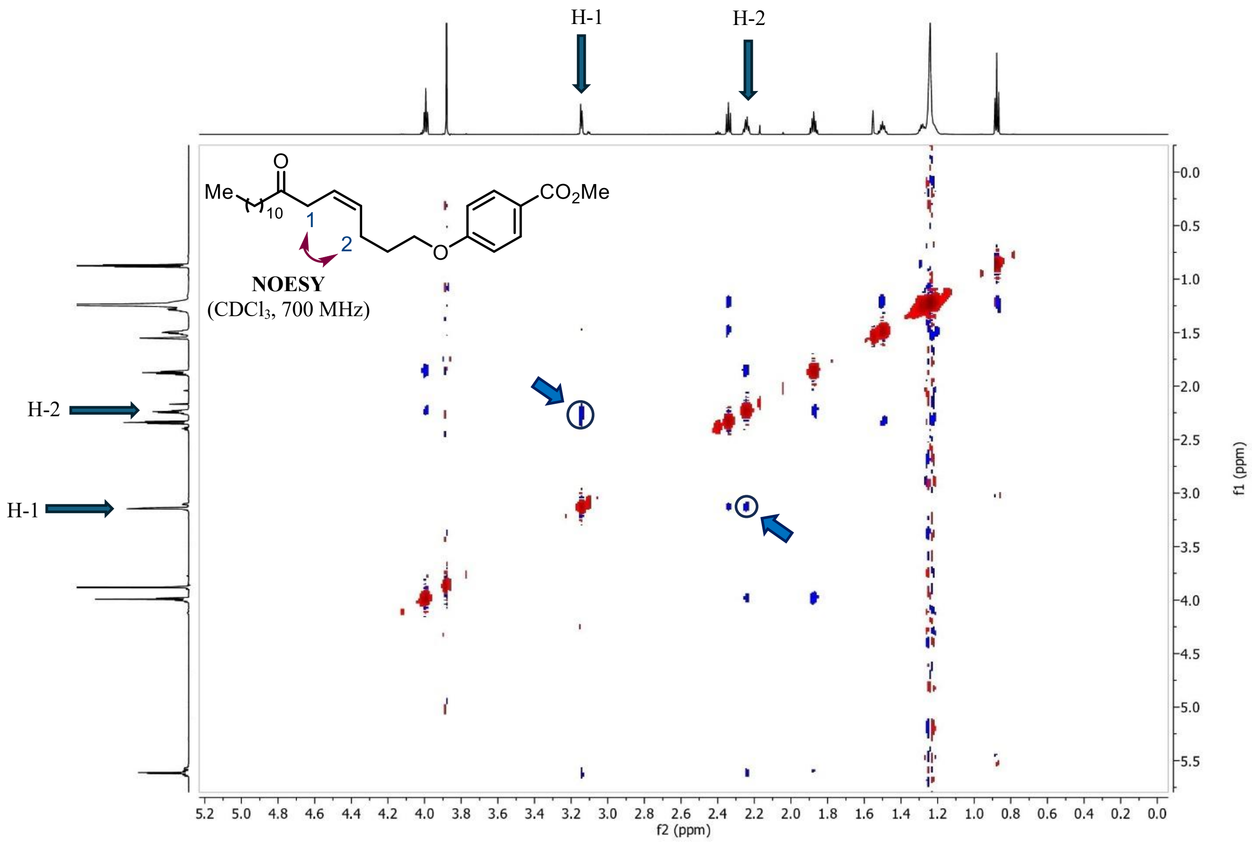
H-1
H-2
Me
10
1
2
CO2Me
O
NOESY
(CDCl3, 700 MHz)
f1 (ppm)
f2 (ppm)

(*Z*)-*N*,*N*-Dimethyl-12-oxotricos-9-enamide (5j)

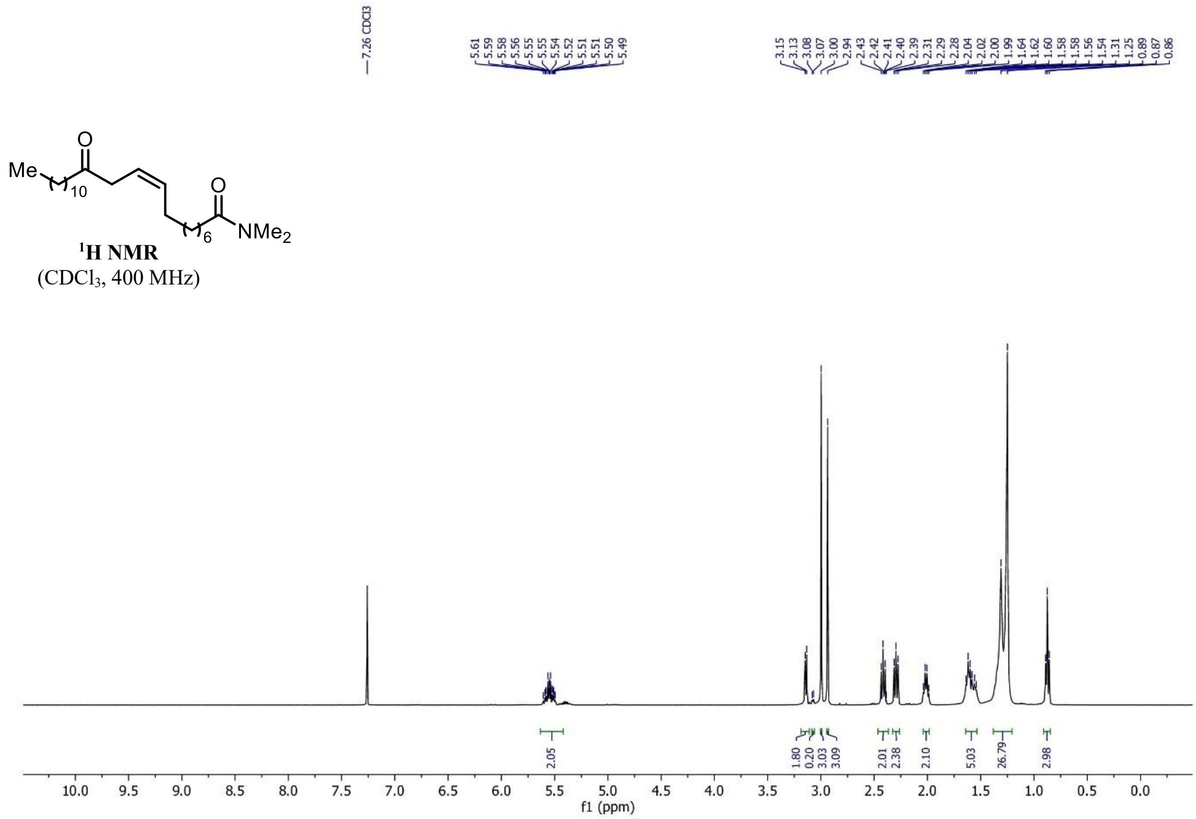

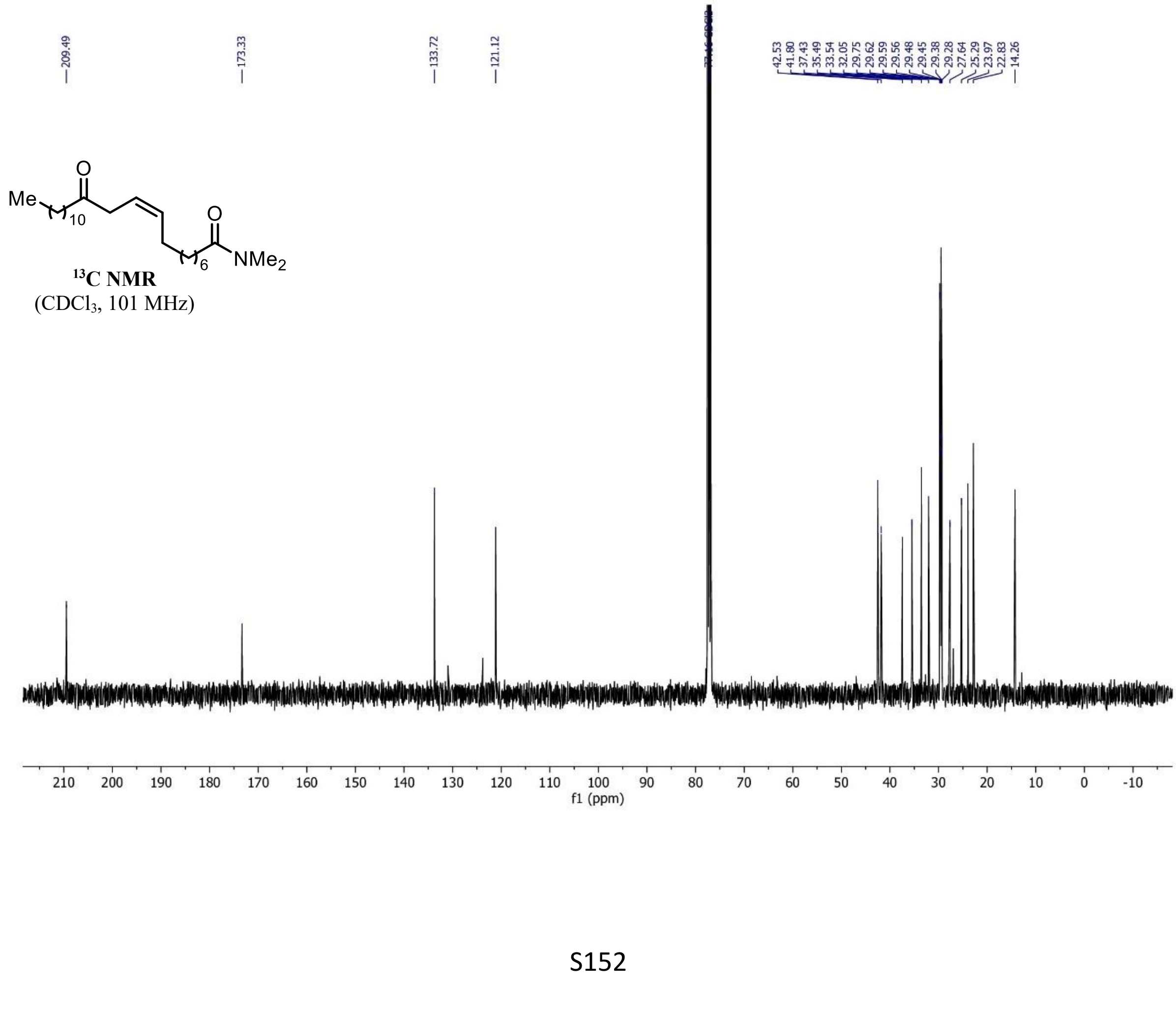

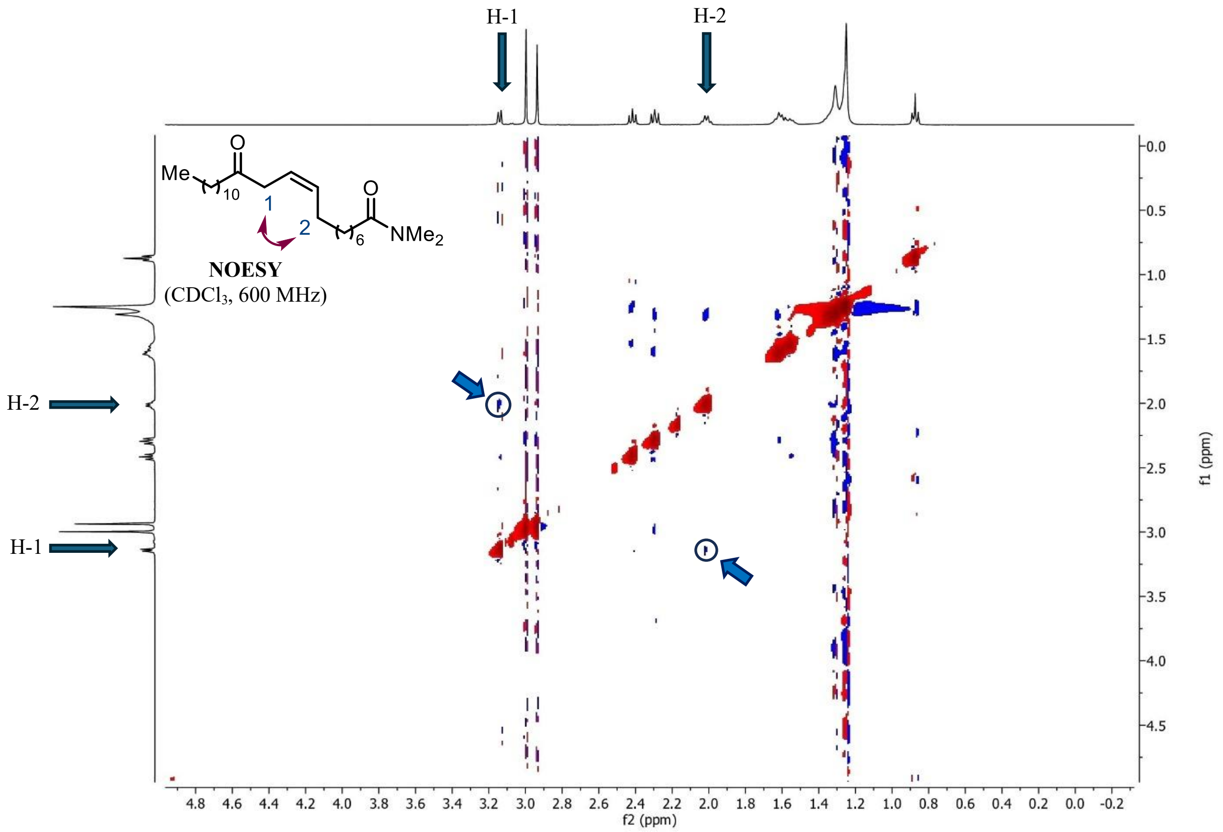
H-1
H-2
Me
O
10
1
2
6
O
NMe2
NOESY
(CDCl3, 600 MHz)
f2 (ppm)
f1 (ppm)

Ethyl (2*E*,11*Z*)-14-oxopentacosa-2,11-dienoate (5k)

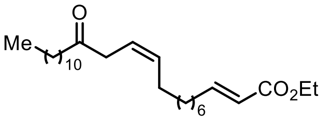

^{1}H NMR
($CDCl_3$, 400 MHz)

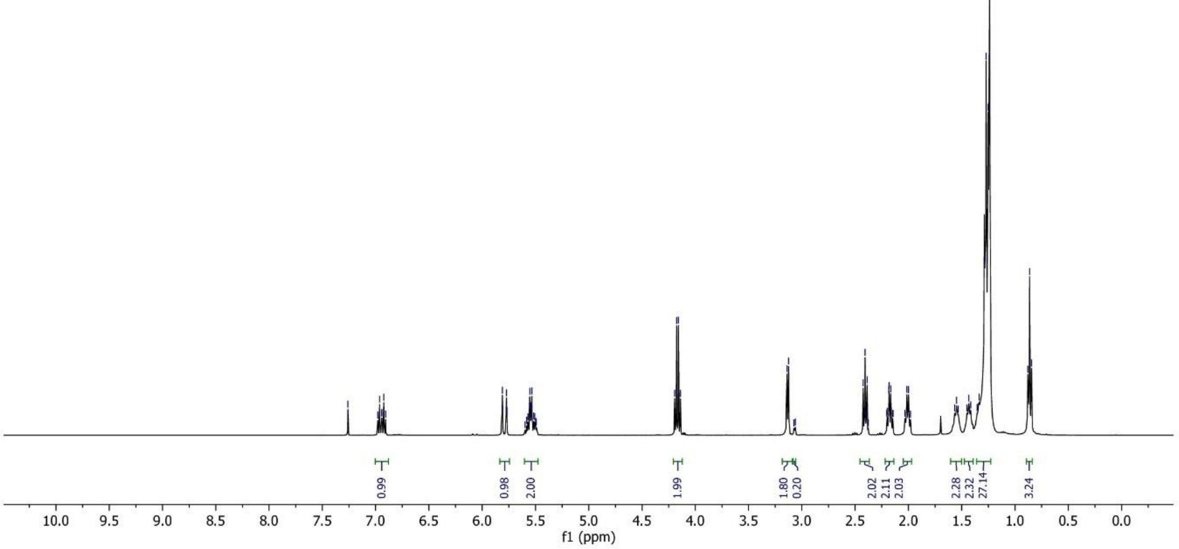

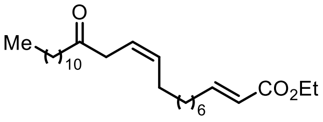

^{13}C NMR
($CDCl_3$, 101 MHz)

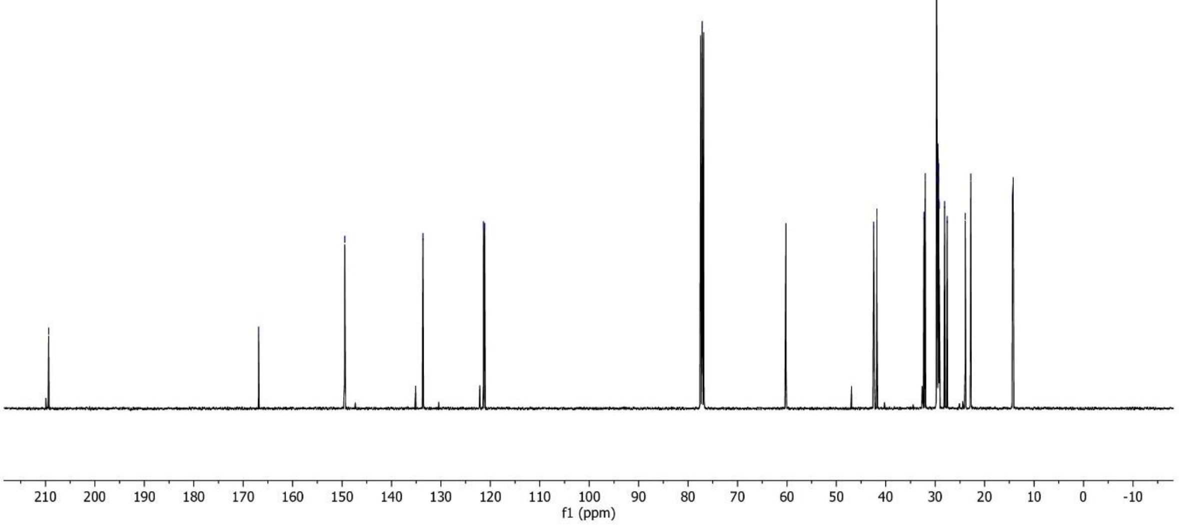

Methyl (*Z*)-8-oxononadec-5-enoate (5l)

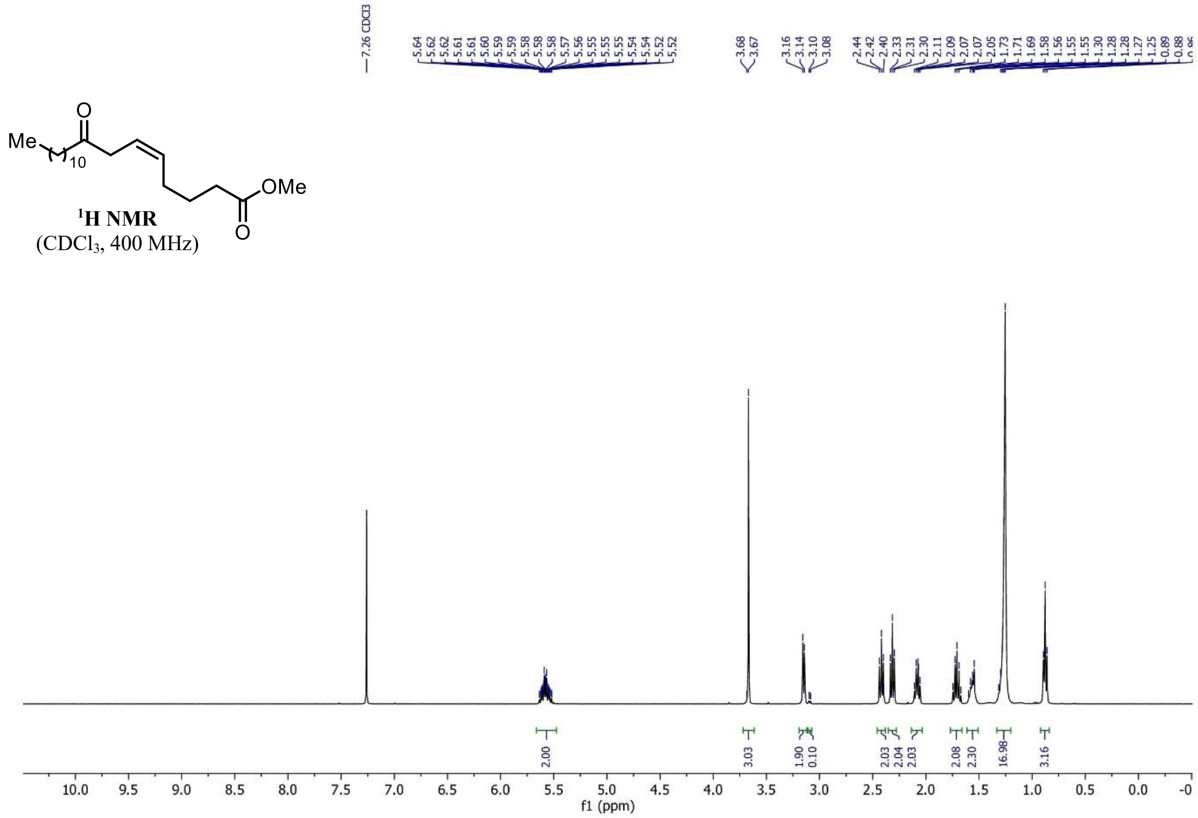

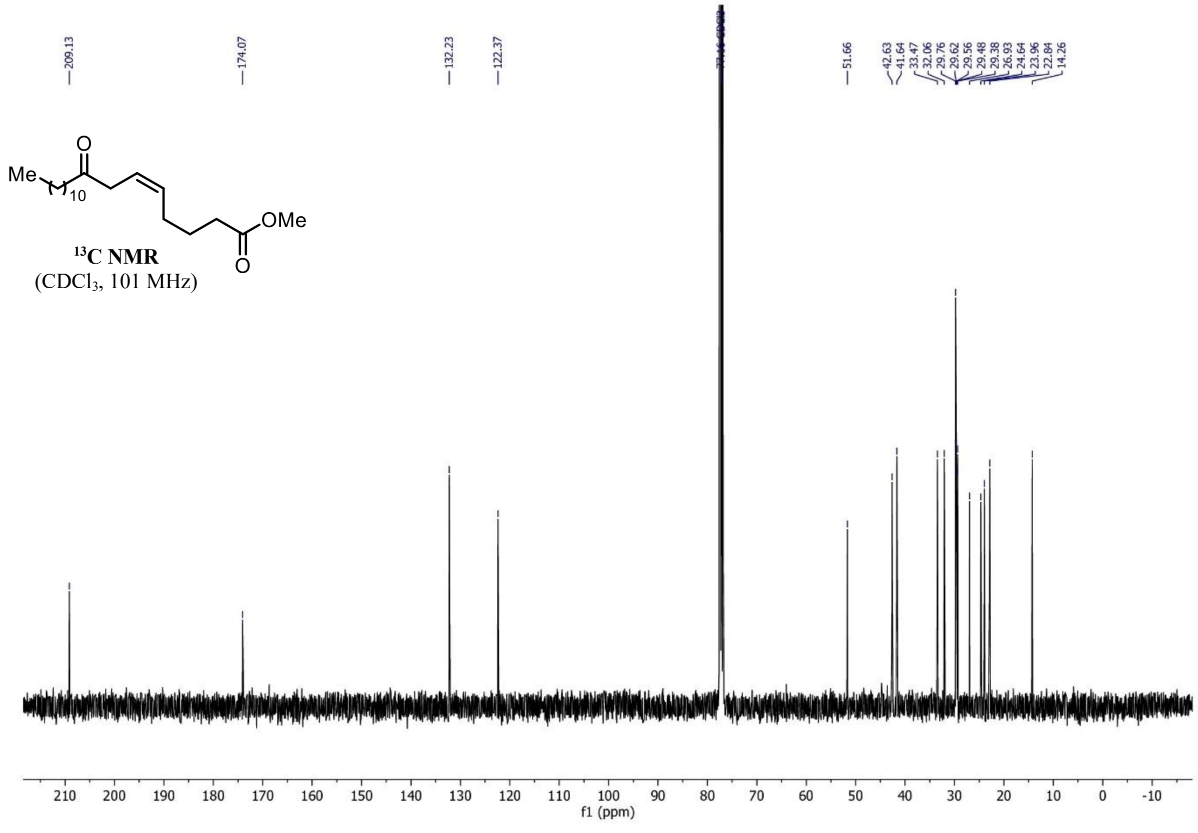

(*S*)-1-(4-Fluorophenyl)-3-((2*S*,3*R*)-1-(4-fluorophenyl)-2-(4-methoxyphenyl)-4-oxoazetidin-3-yl)propyl (*Z*)-12-oxotricos-9-enoate (5m)

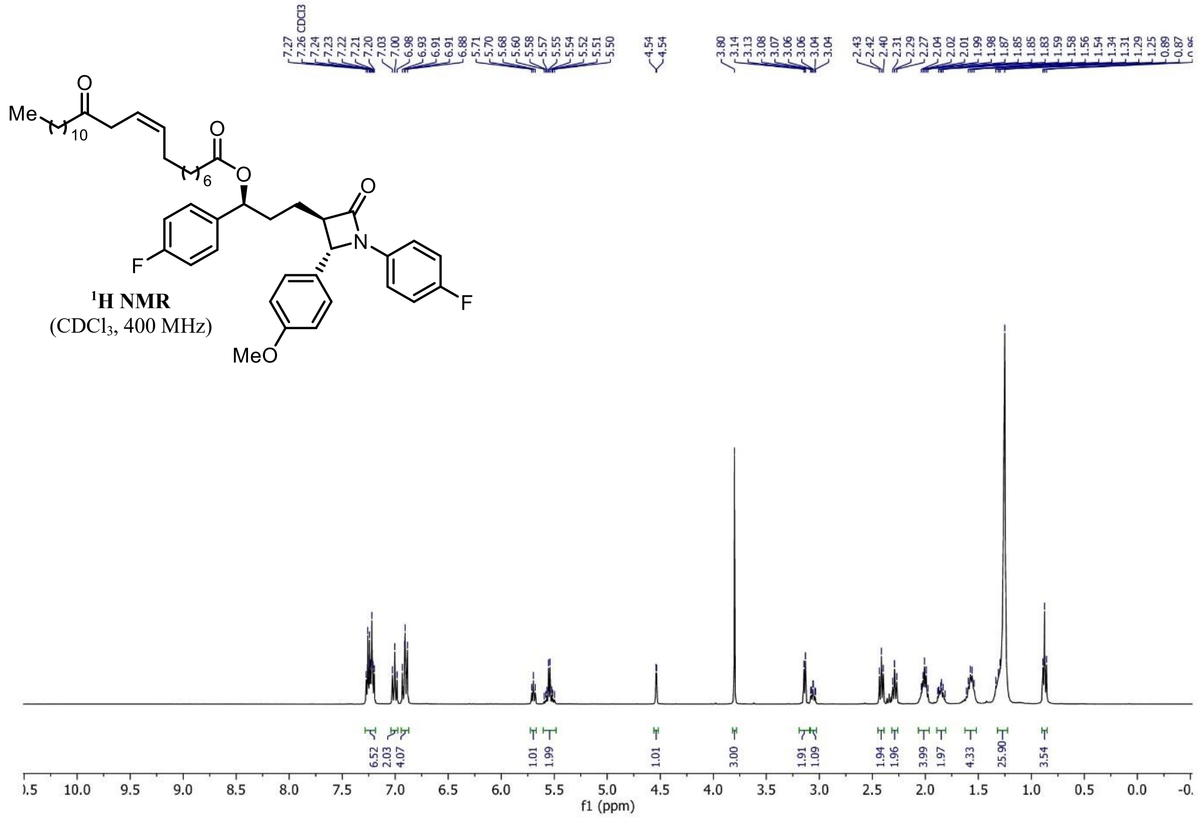

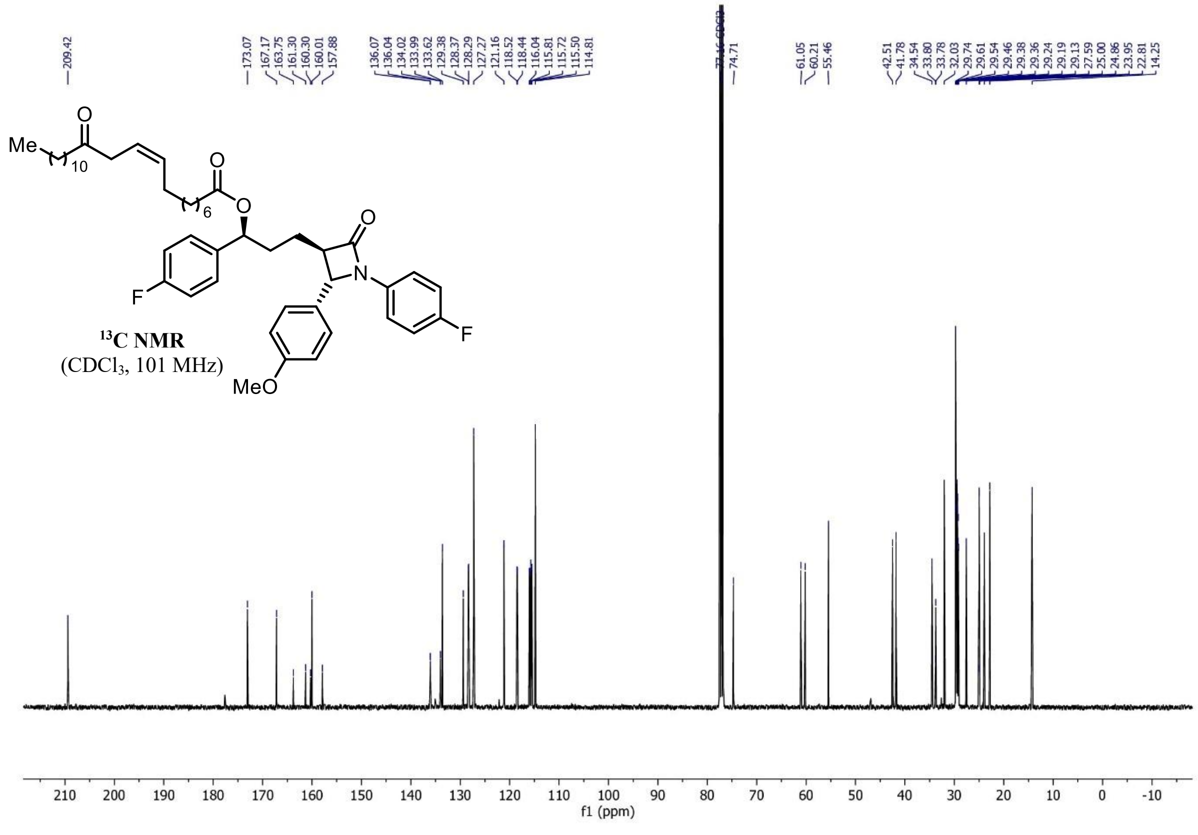

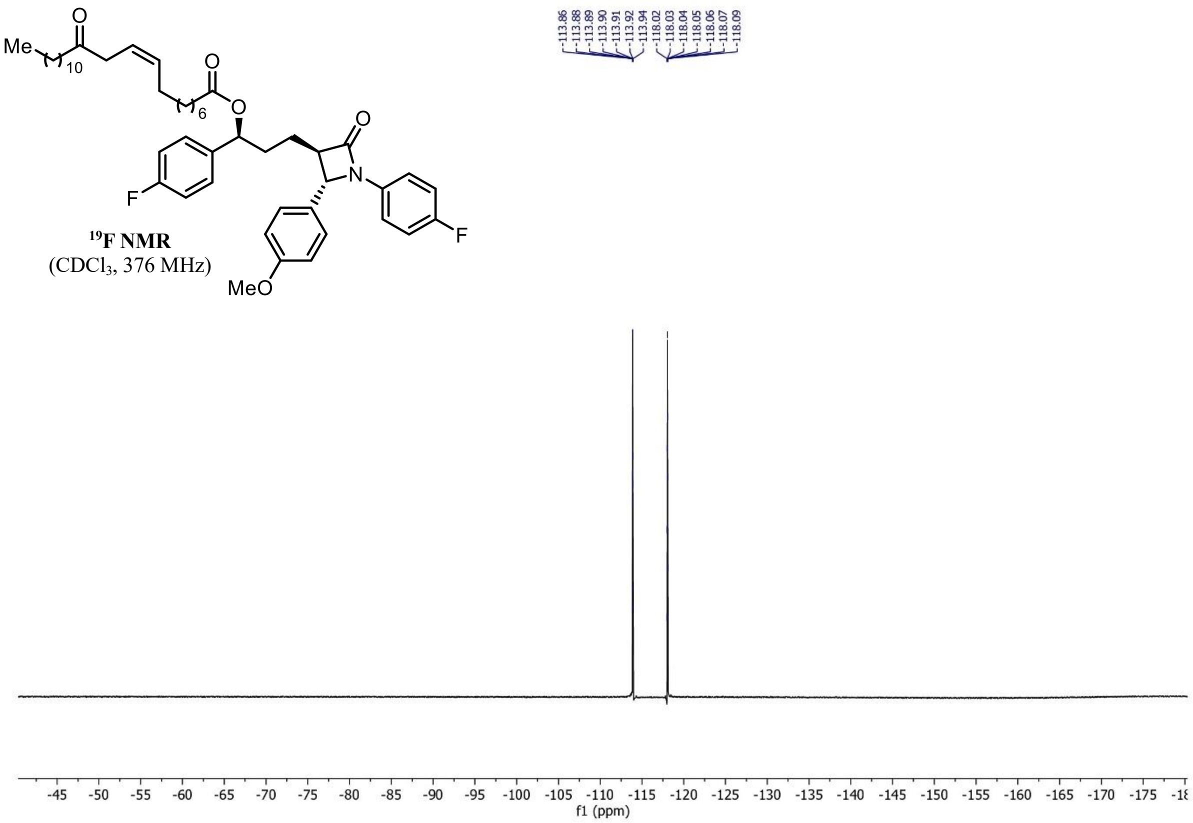

¹⁹F NMR
(CDCl3, 376 MHz)
MeO
F
f1 (ppm)

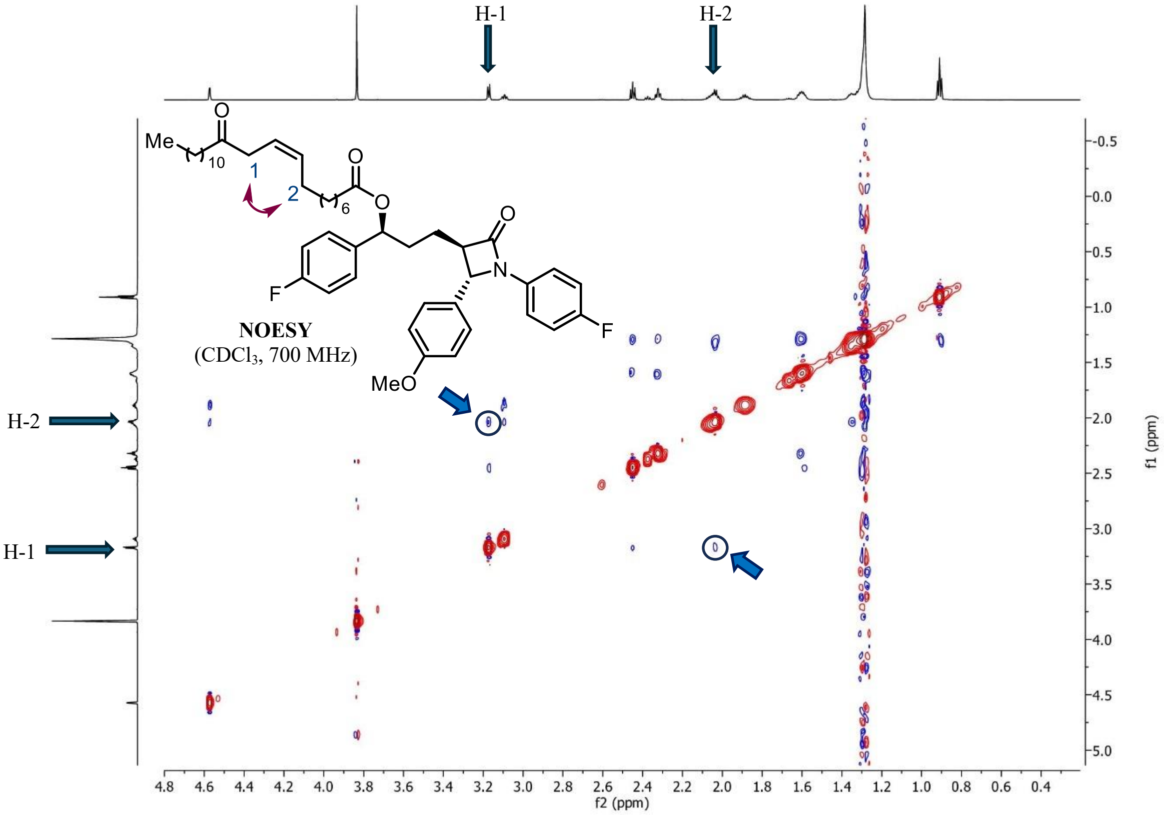

H-1
H-2
NOESY
(CDCl3, 700 MHz)
MeO
F
f2 (ppm)
f1 (ppm)

(*Z*)-6-Butyloctadec-4-en-7-one (5n)

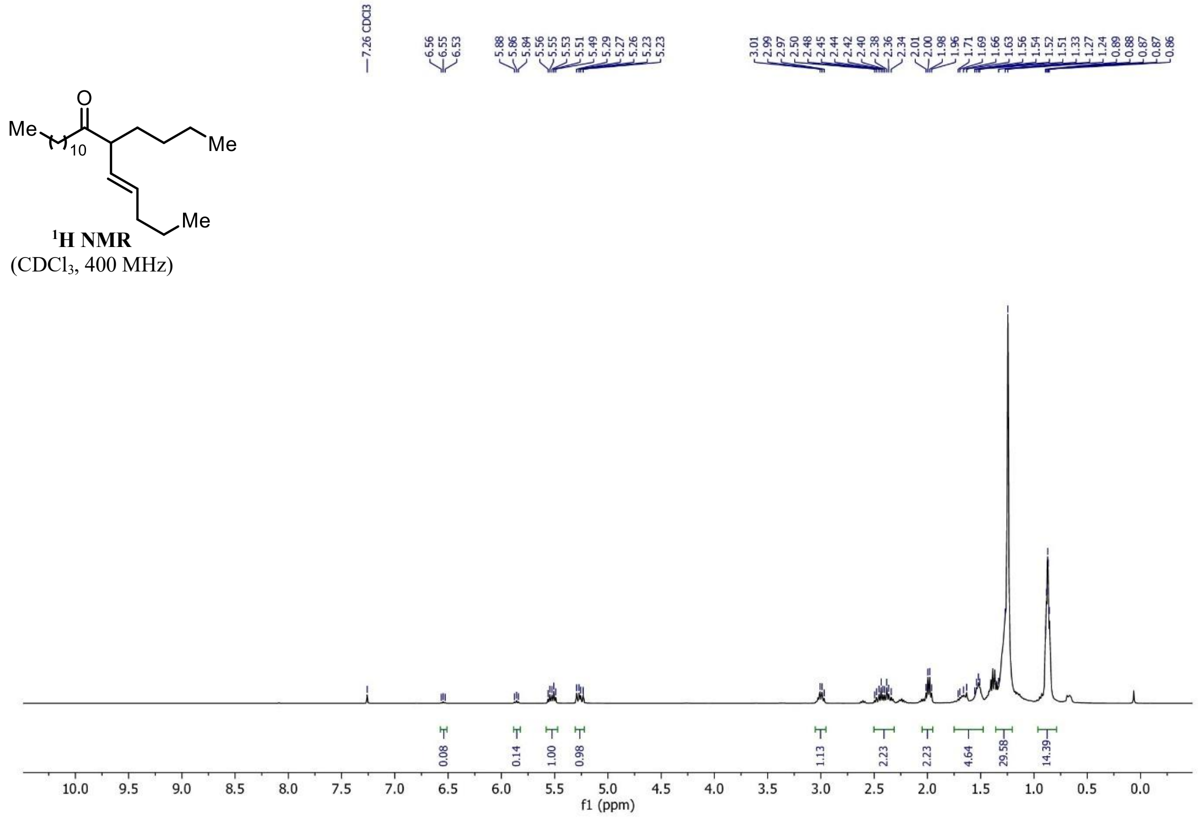

^{1}H NMR
($CDCl_3$, 400 MHz)

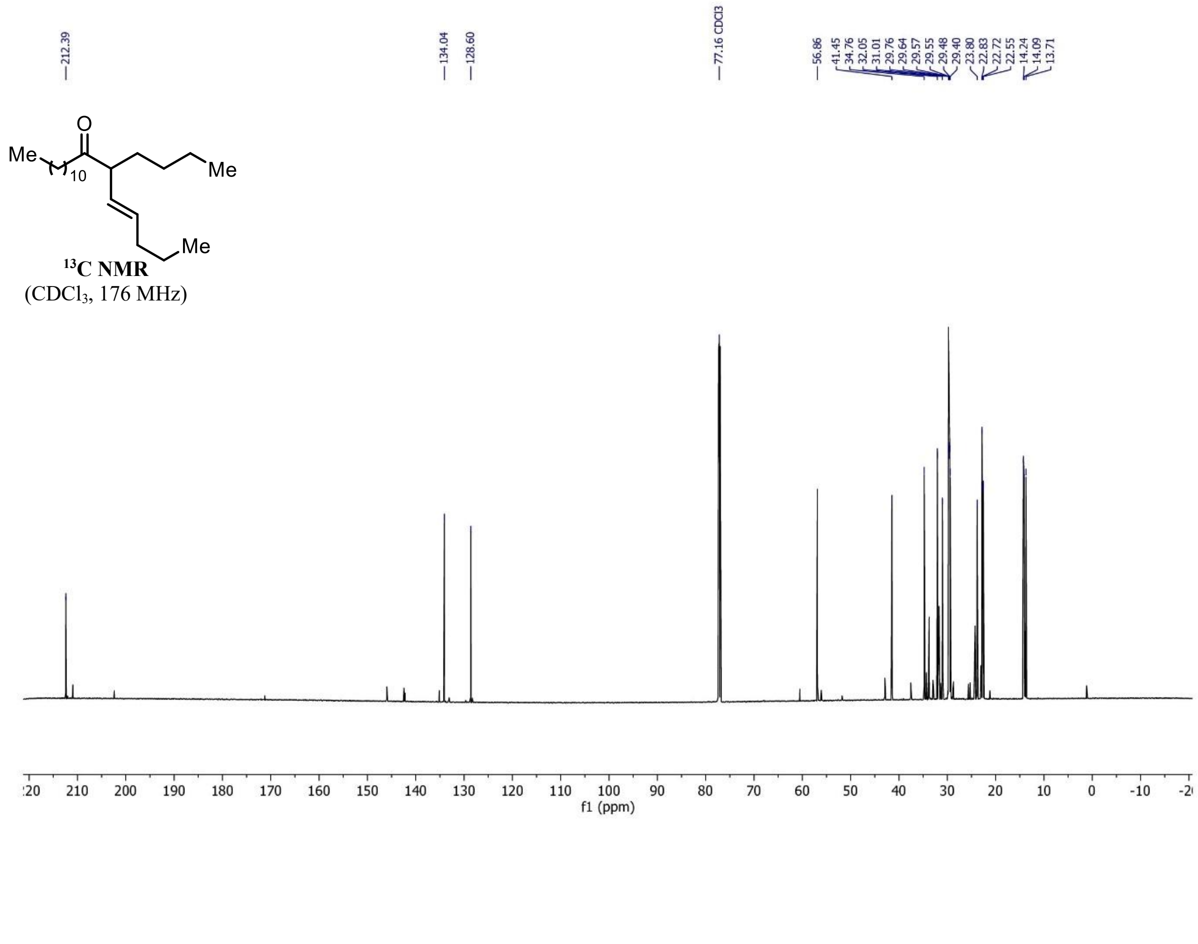

^{13}C NMR
($CDCl_3$, 176 MHz)

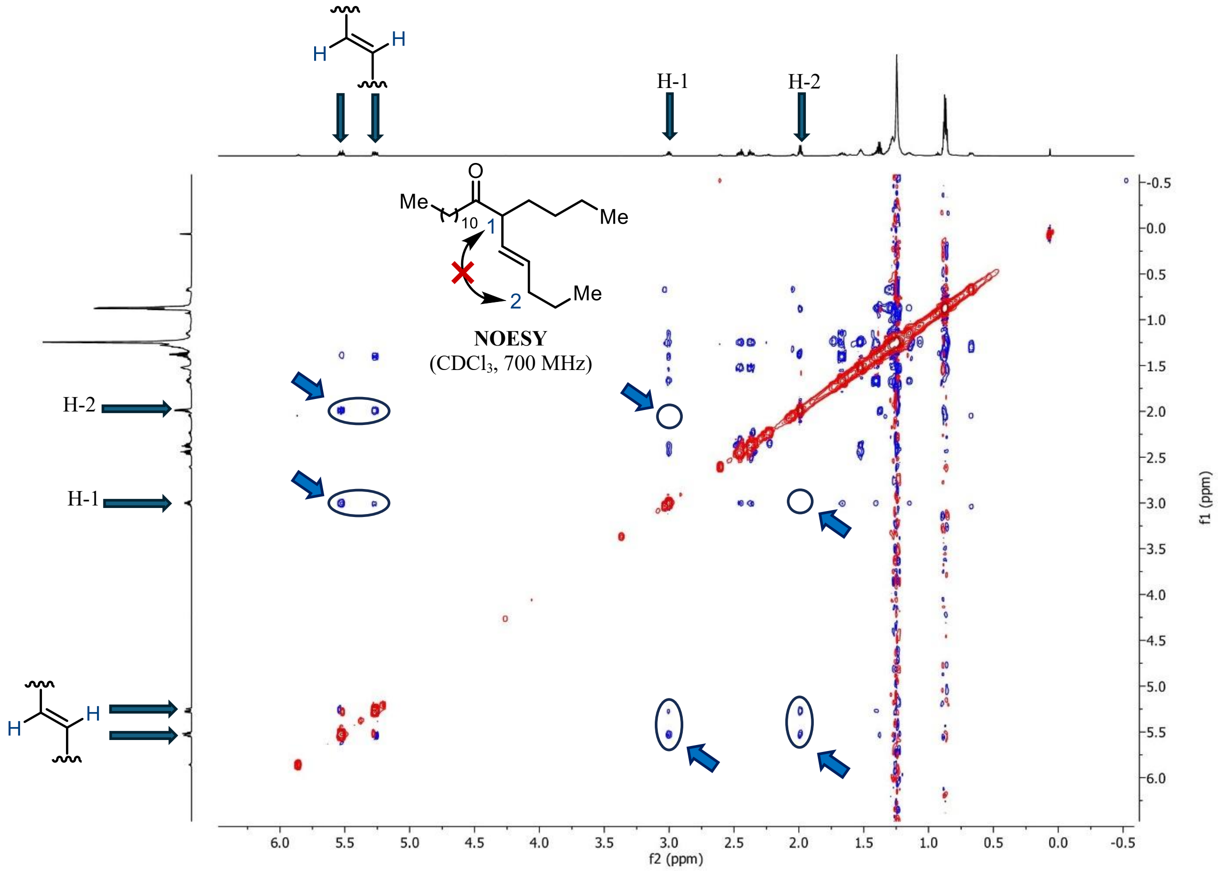

H-1
H-2
H-2
H-1
Me
O
10
1
Me
2
Me
NOESY
(CDCl3, 700 MHz)
f2 (ppm)
f1 (ppm)

Methyl (*Z*)-12-oxooctadec-9-enoate (5o)

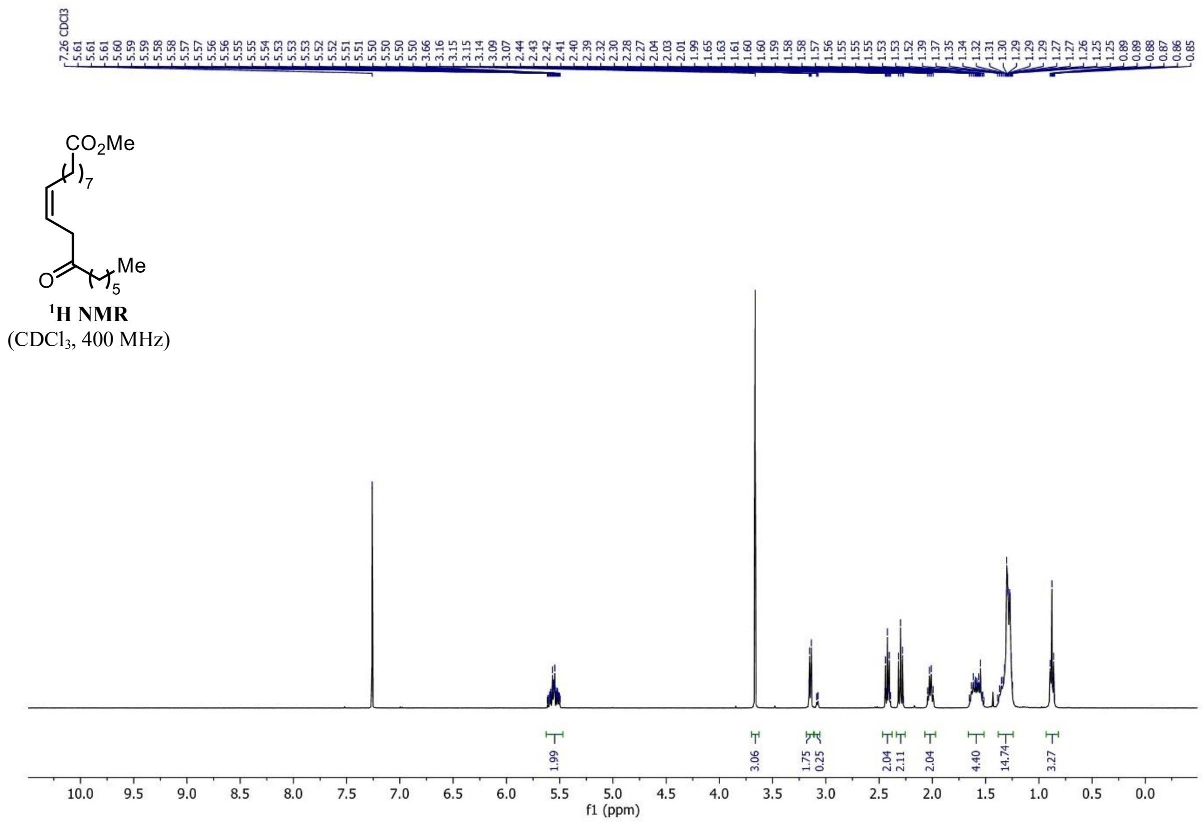

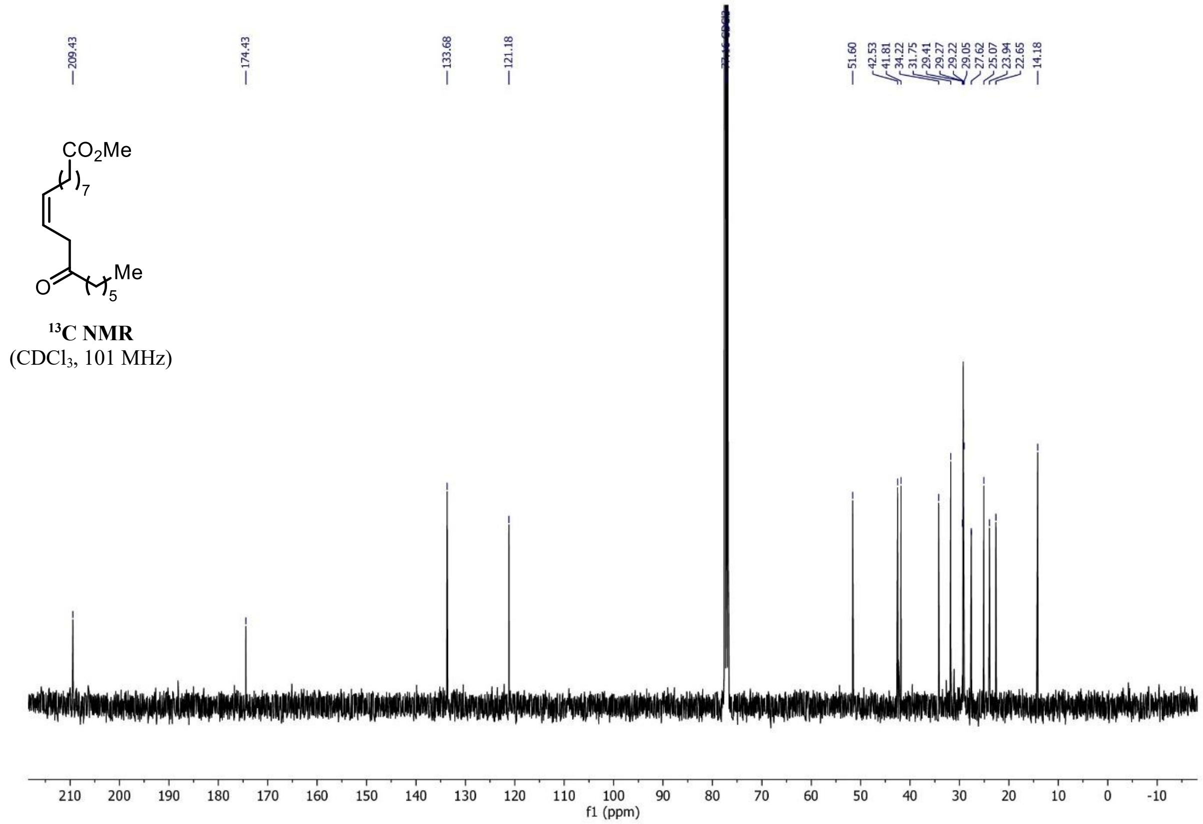

Methyl (*Z*)-14-oxoicos-11-enoate (5p)

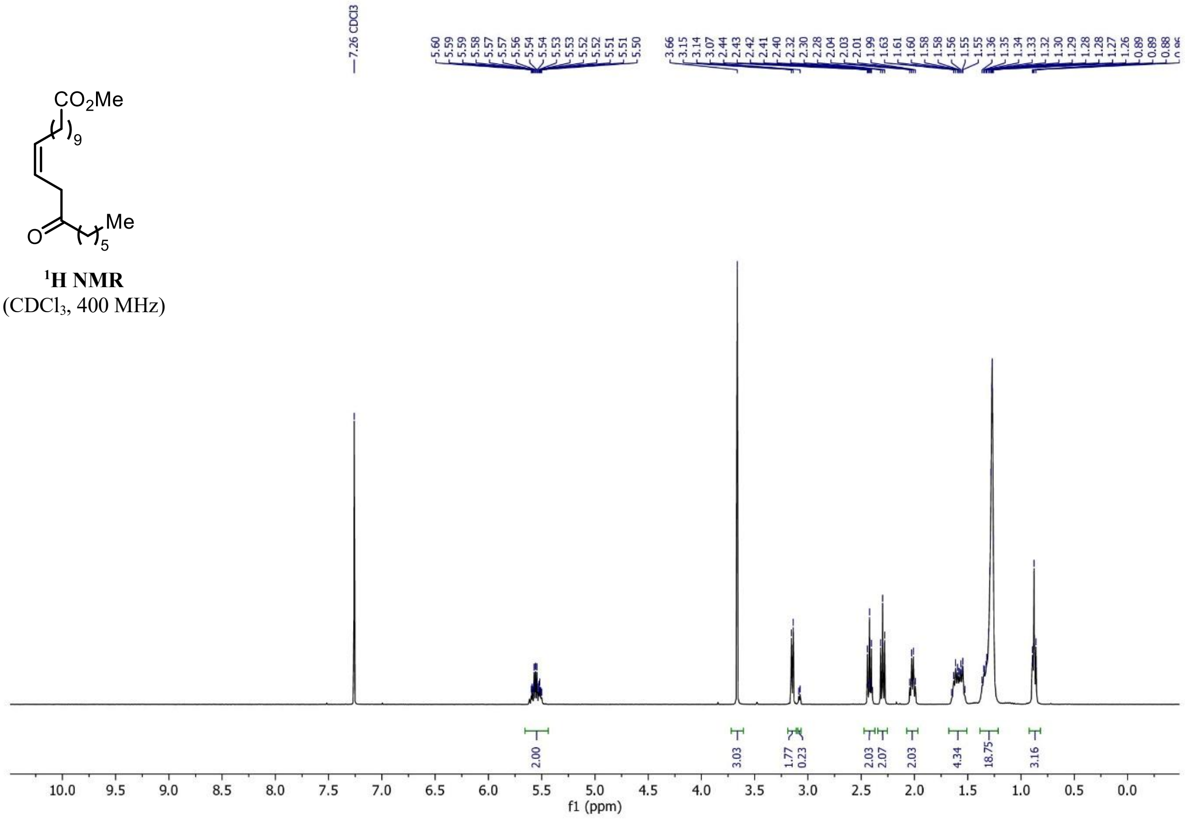

^{1}H NMR
($CDCl_3$, 400 MHz)

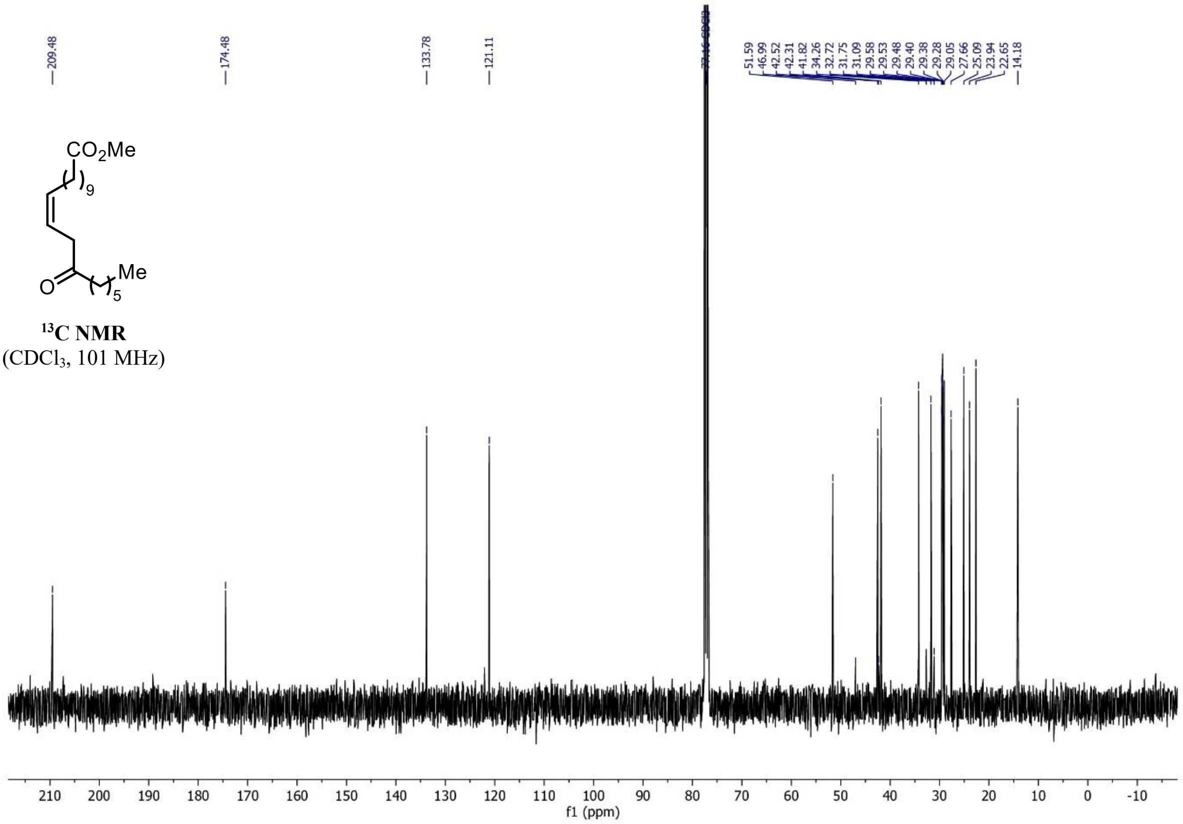

^{13}C NMR
($CDCl_3$, 101 MHz)

Methyl (9*Z*,15*Z*)-12-oxooctadeca-9,15-dienoate (5q)

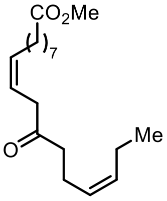

^{1}H NMR
($CDCl_3$, 400 MHz)

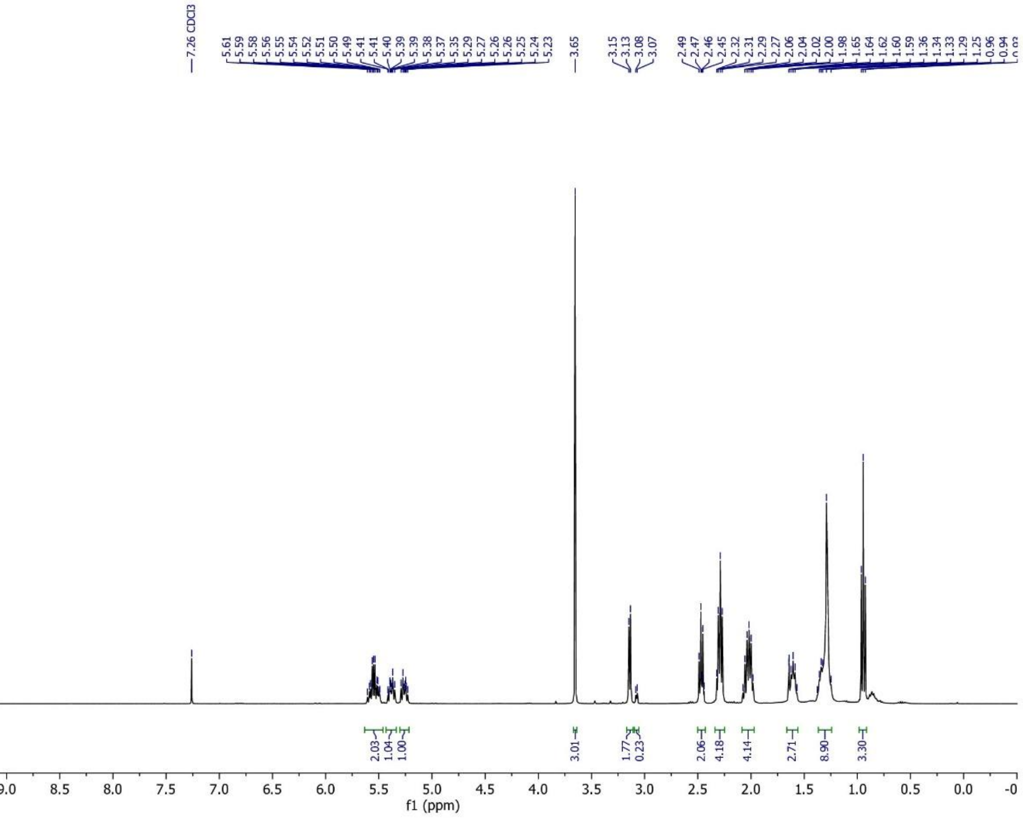

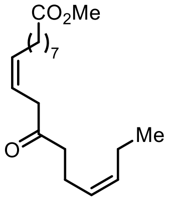

^{13}C NMR
($CDCl_3$, 101 MHz)

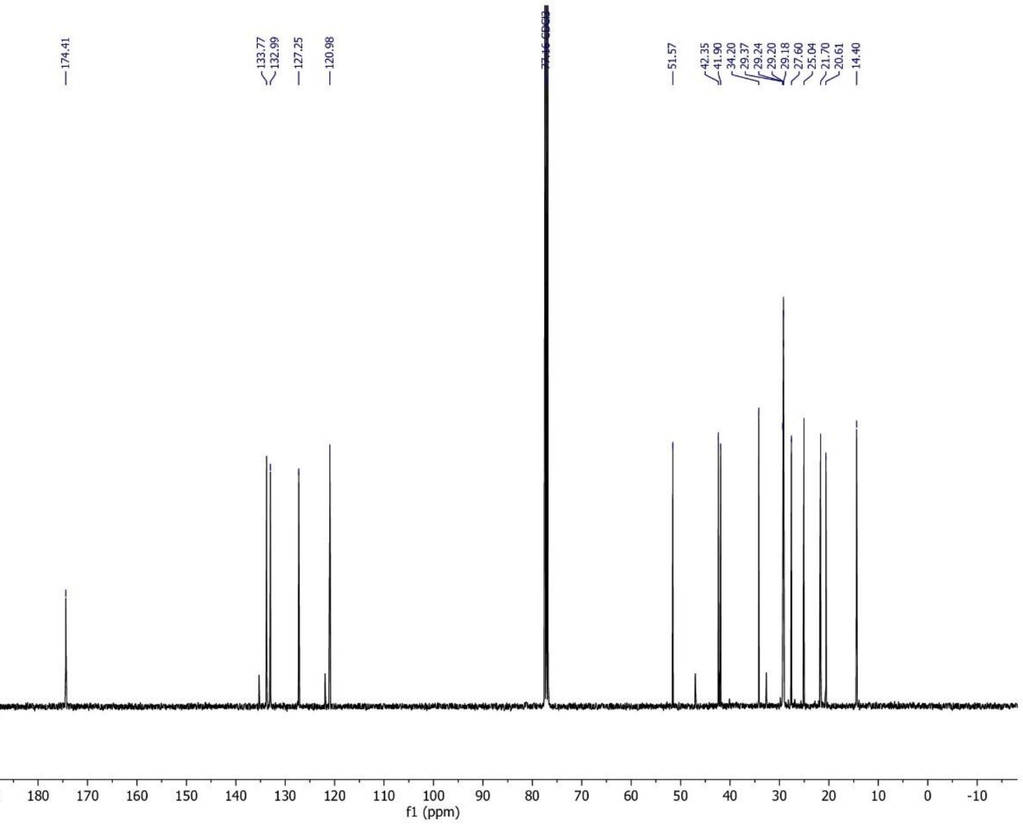

Methyl (11*Z*,17*Z*)-14-oxoicosa-11,17-dienoate (5r)

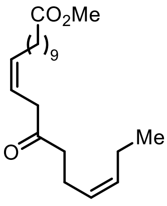

^{1}H NMR
($CDCl_3$, 400 MHz)

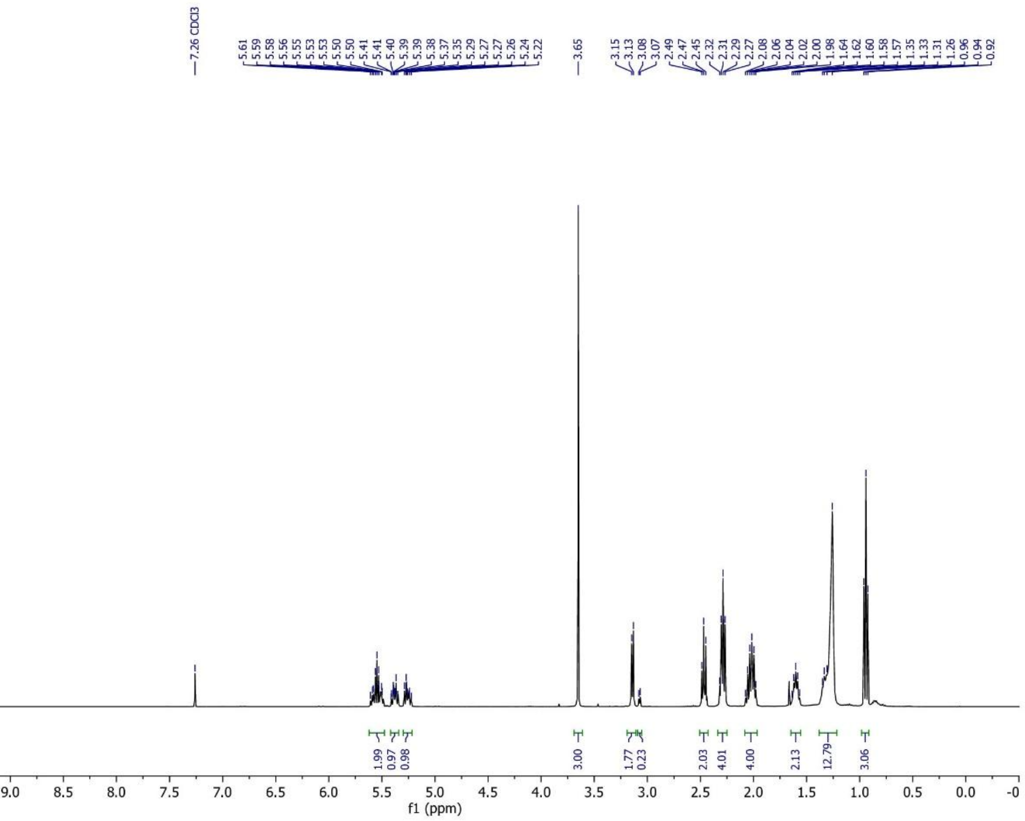

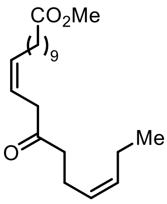

^{13}C NMR
($CDCl_3$, 101 MHz)

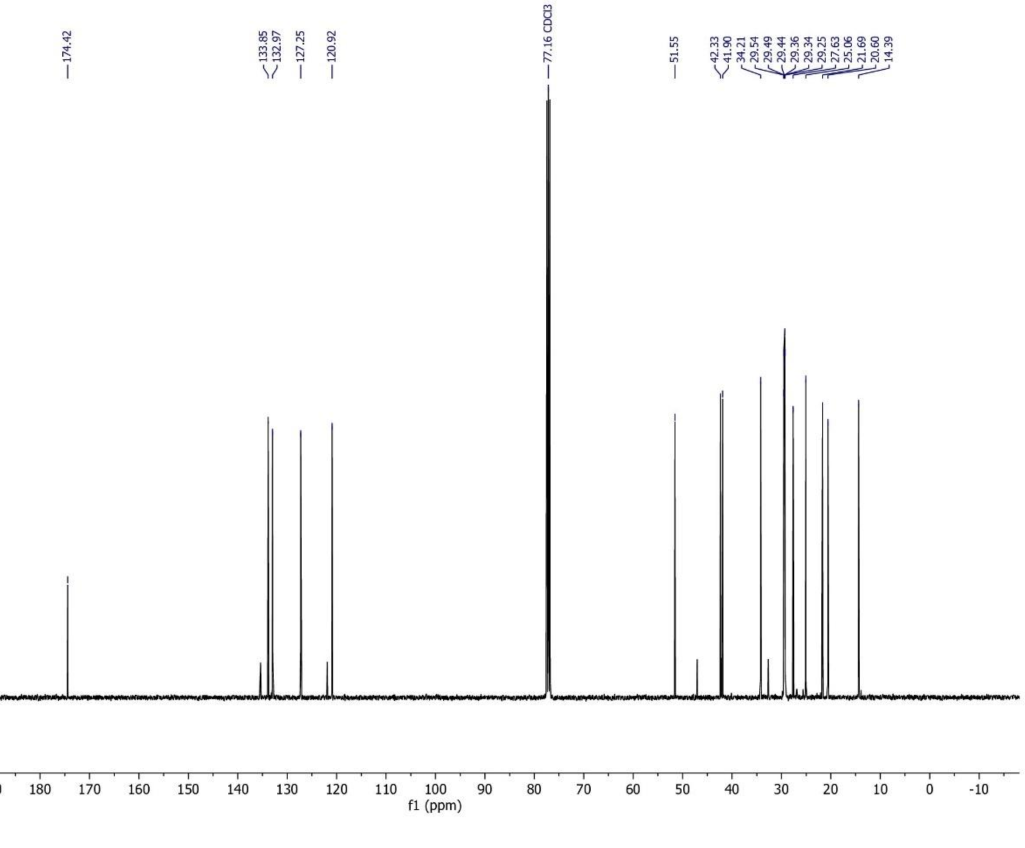

(±)-Methyl (*Z*)-12-hydroxyoctadec-9-enoate (S9)

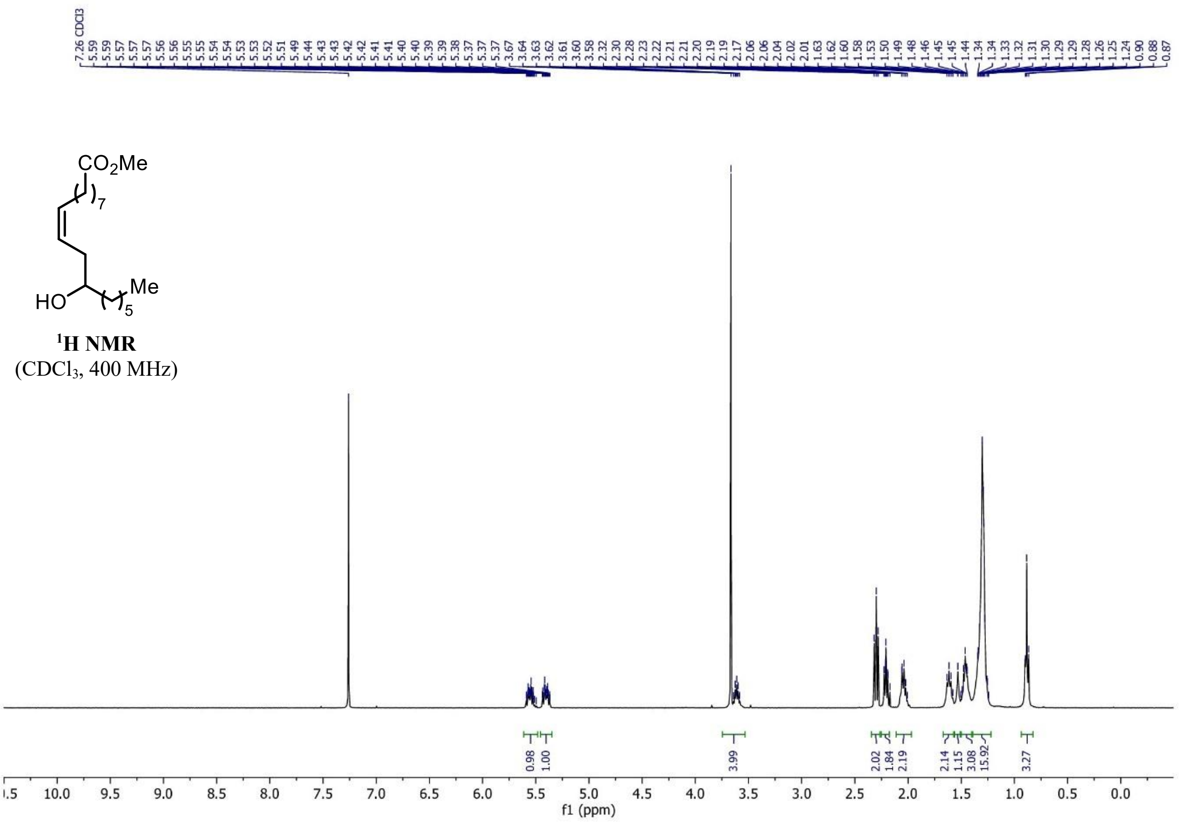

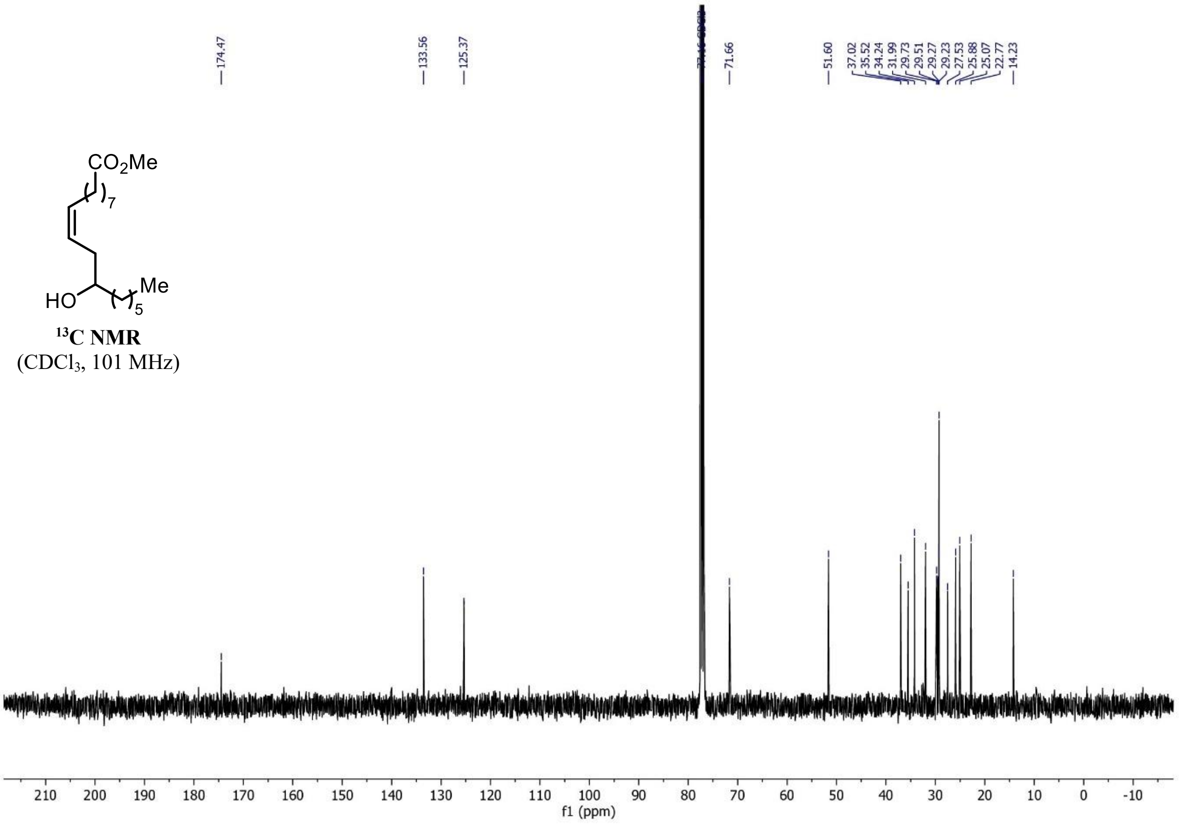

(±)-Methyl (*Z*)-14-hydroxyicos-11-enoate (S10)

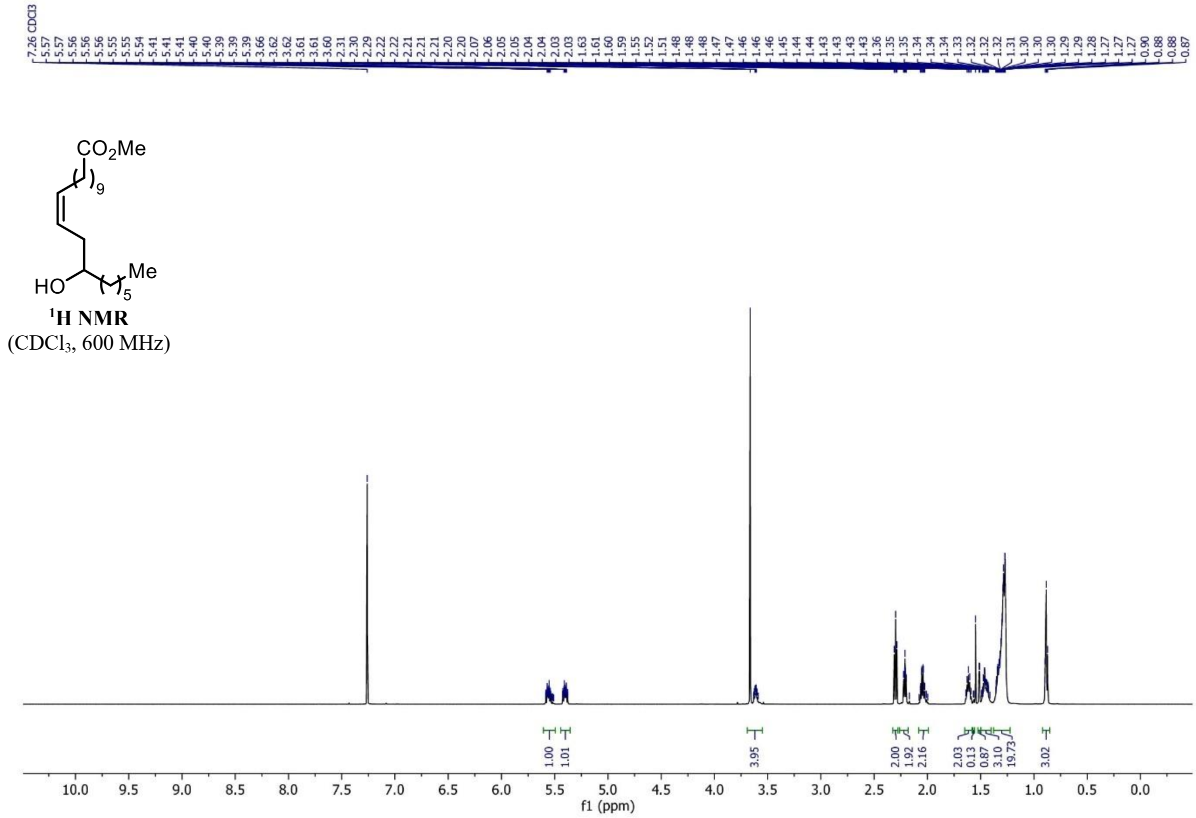

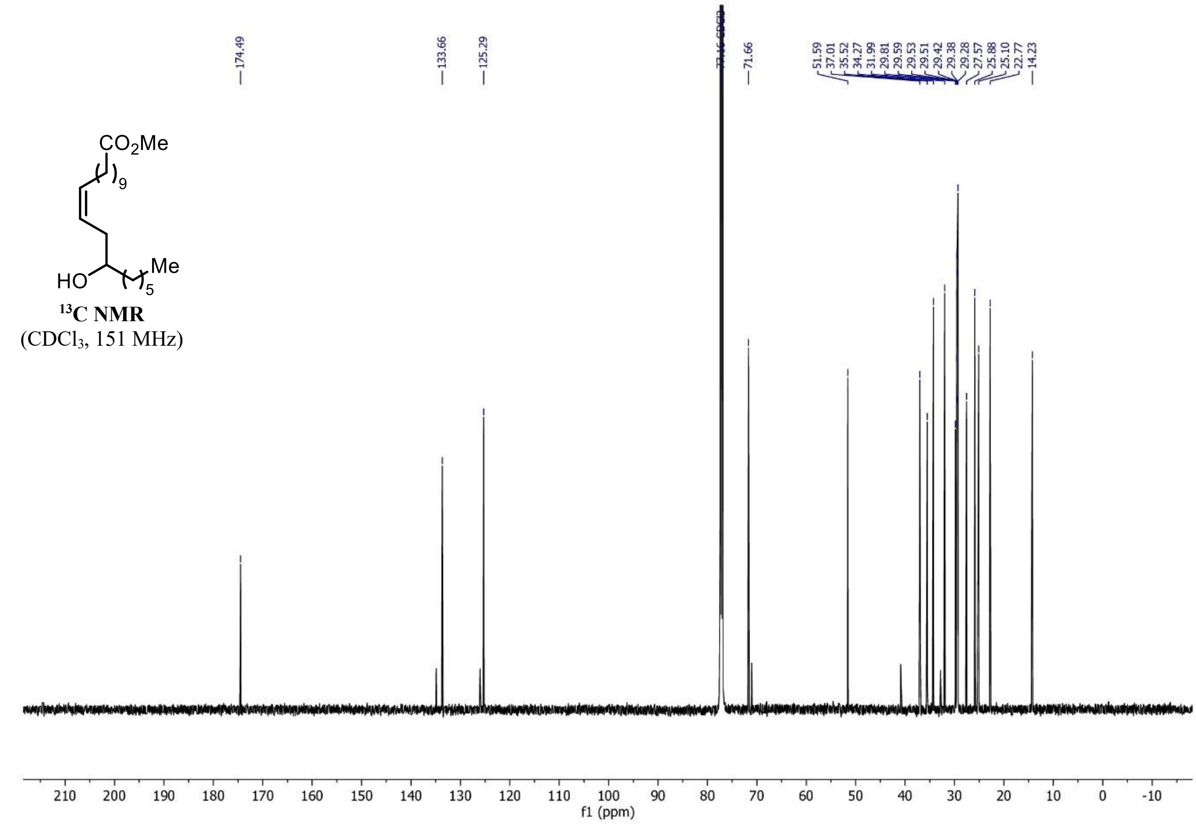

(±)-Methyl (9*Z*,15*Z*)-12-hydroxyoctadeca-9,15-dienoate (S11)

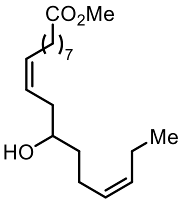

^{1}H NMR
($CDCl_3$, 400 MHz)

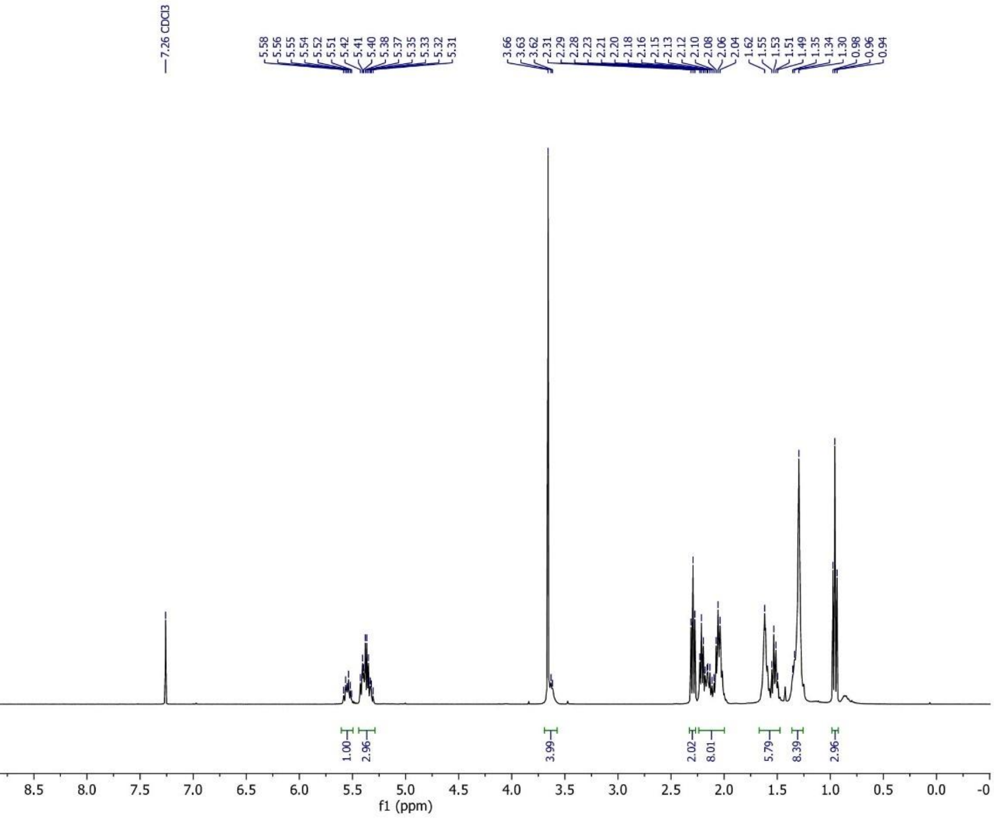

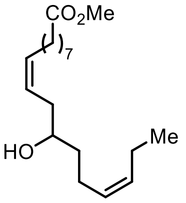

^{13}C NMR
($CDCl_3$, 101 MHz)

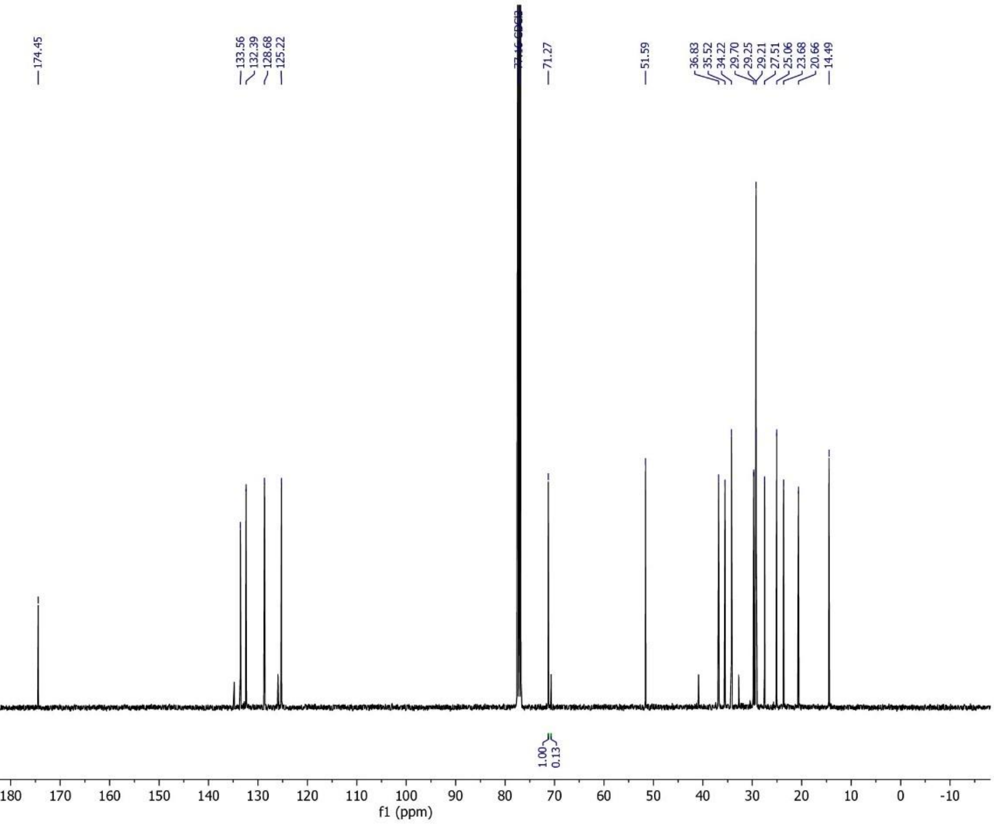

(±)-Methyl (11*Z*,17*Z*)-14-hydroxyicosa-11,17-dienoate (S12)

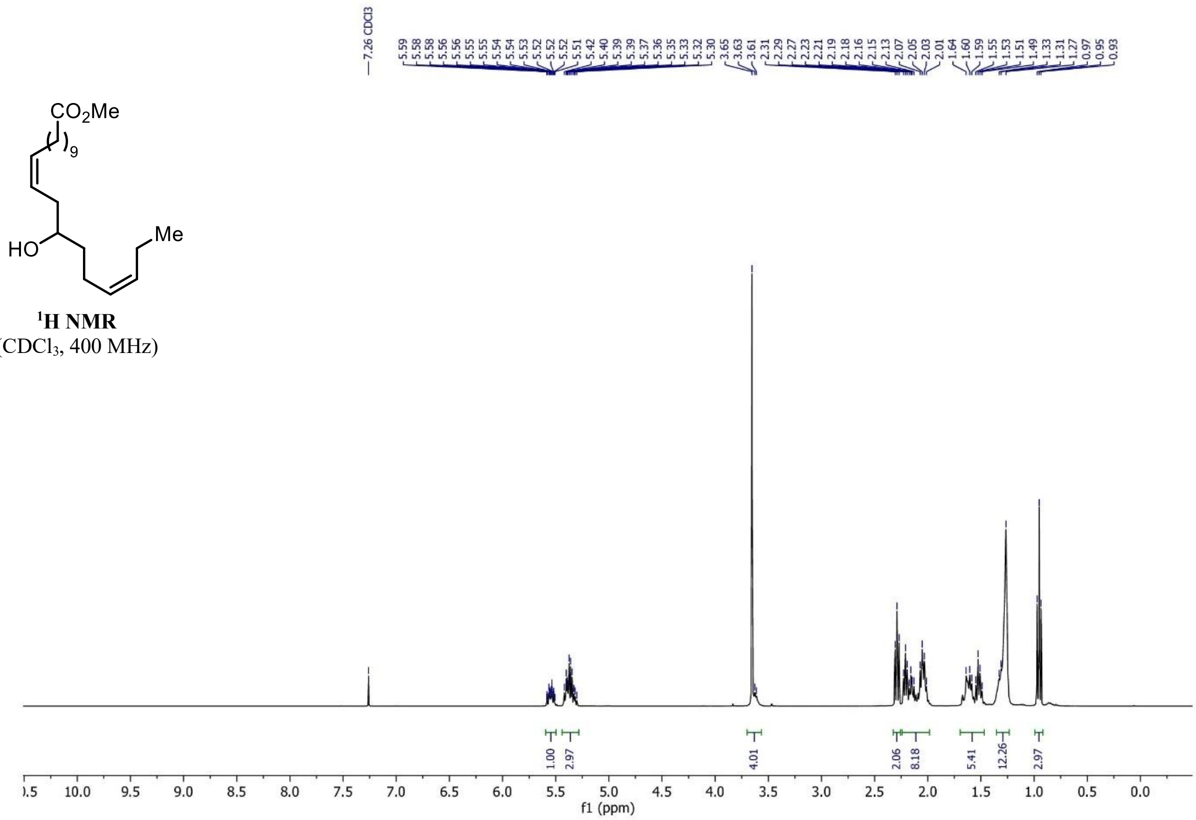

^{1}H NMR
($CDCl_3$, 400 MHz)

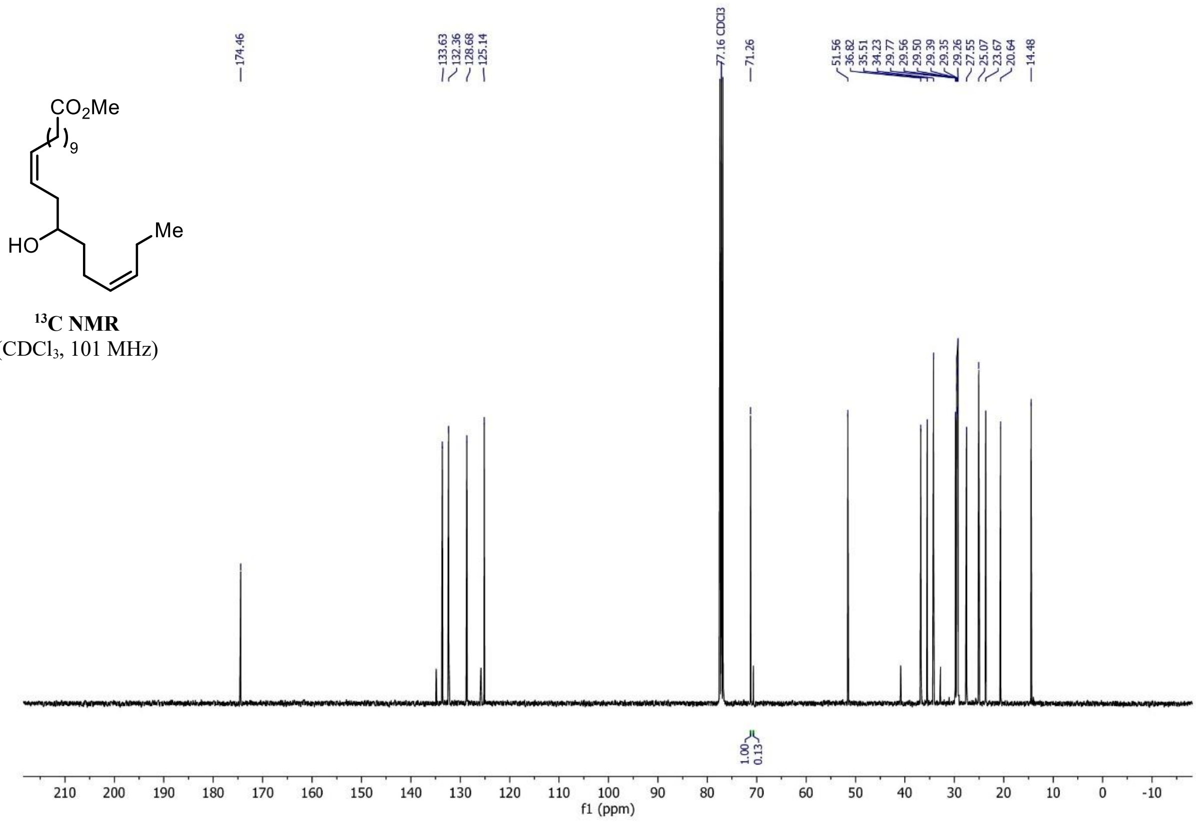

^{13}C NMR
($CDCl_3$, 101 MHz)

(±)-Ricinoleic acid (10a)

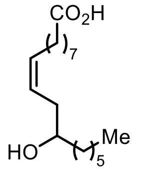

^{1}H NMR
($CDCl_3$, 400 MHz)

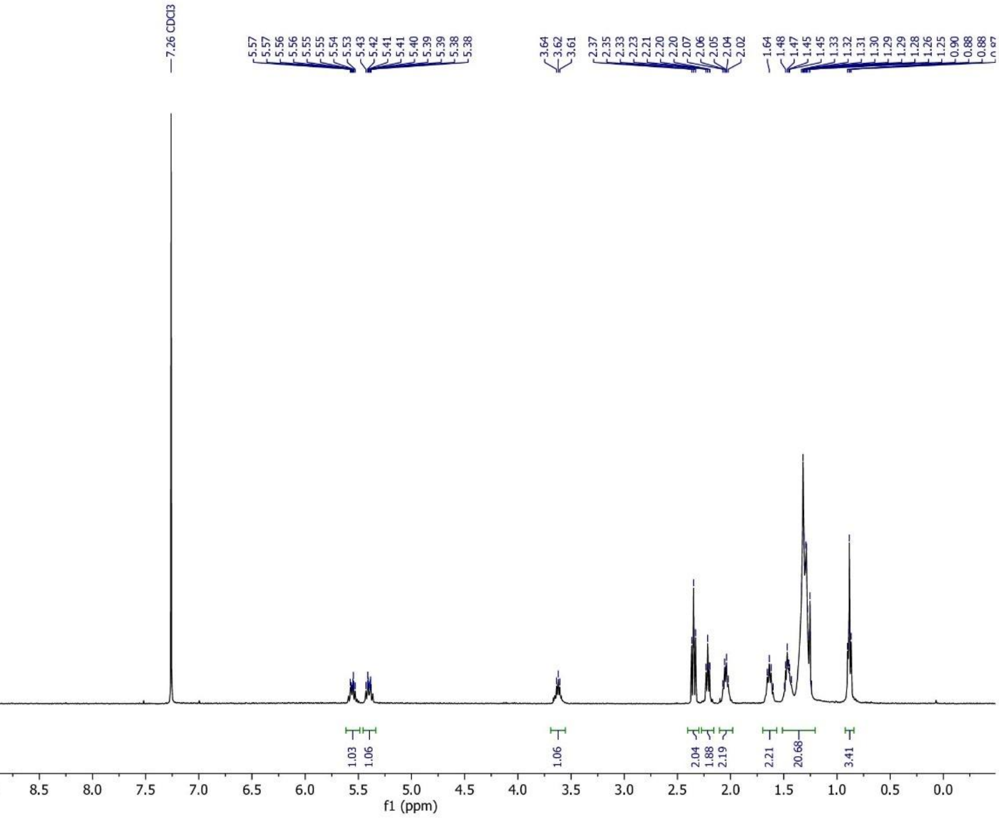

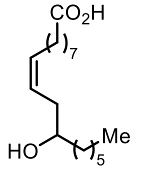

^{13}C NMR
($CDCl_3$, 176 MHz)

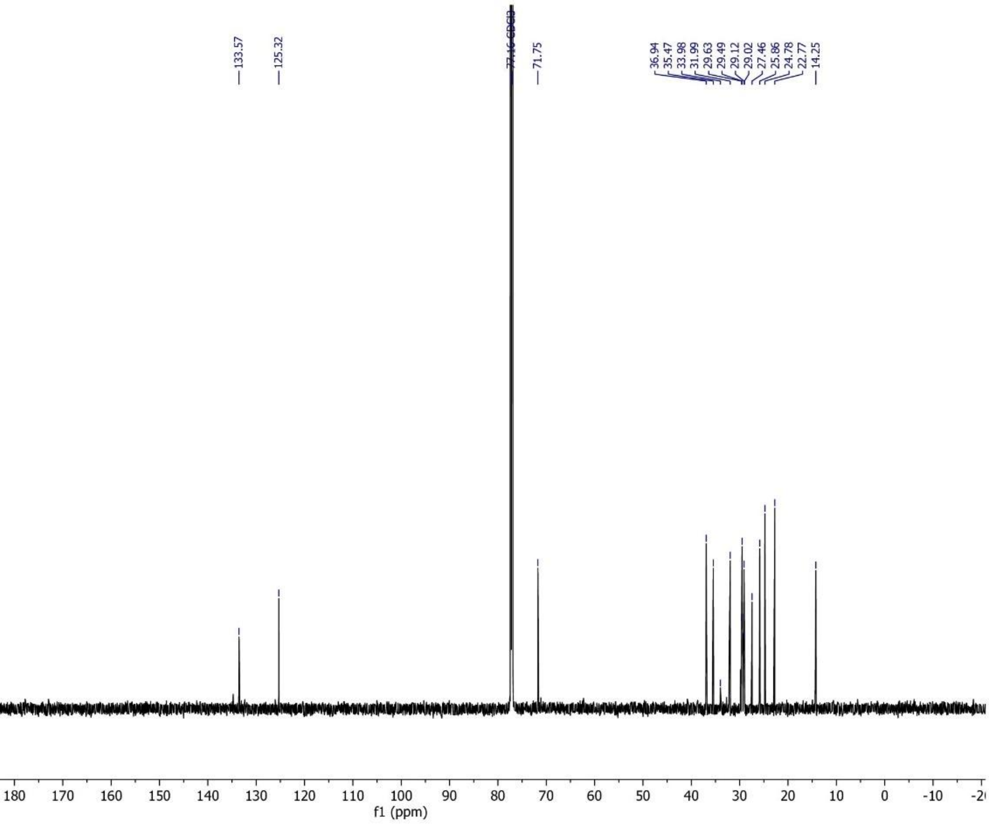

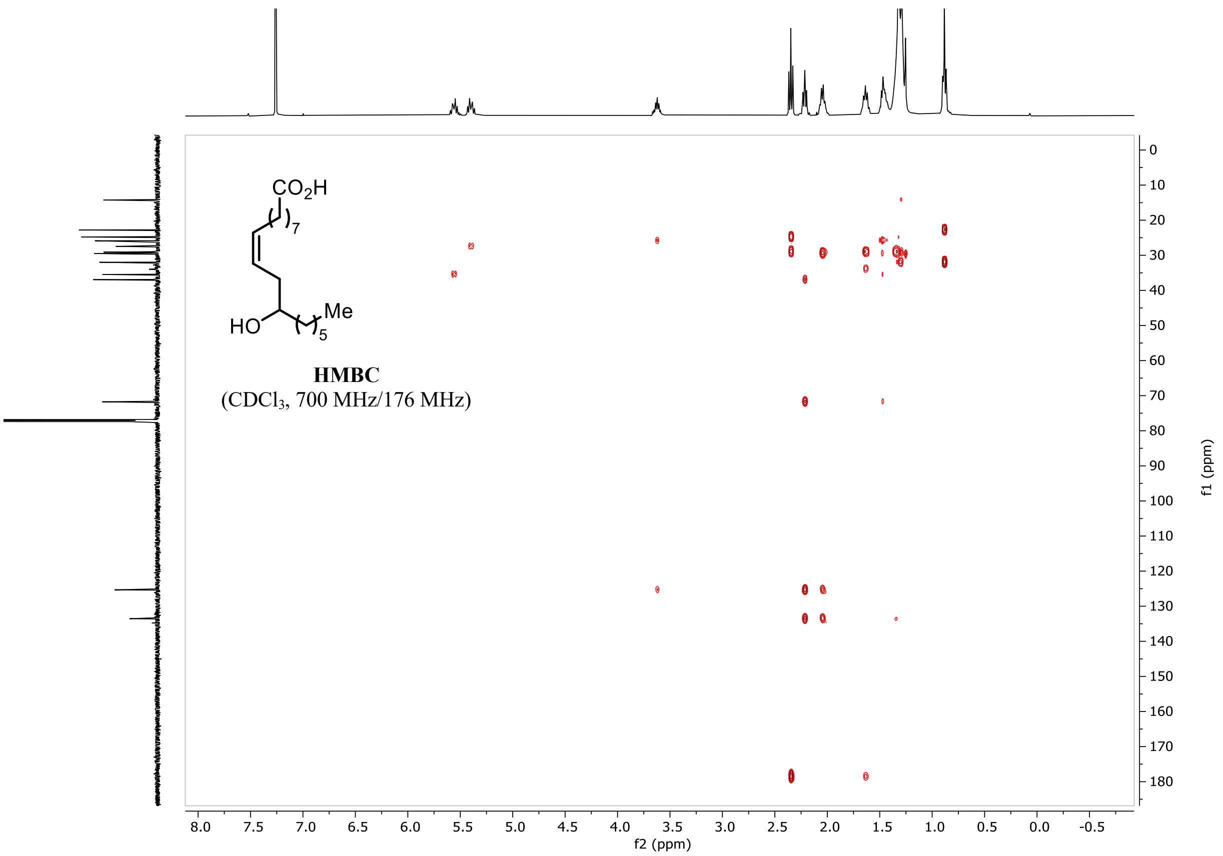

^{13}C NMR comparison between commercial sample of (*R*)-Ricinoleic acid from Sigma Aldrich (top, green) and the racemic synthetic sample (bottom, red)

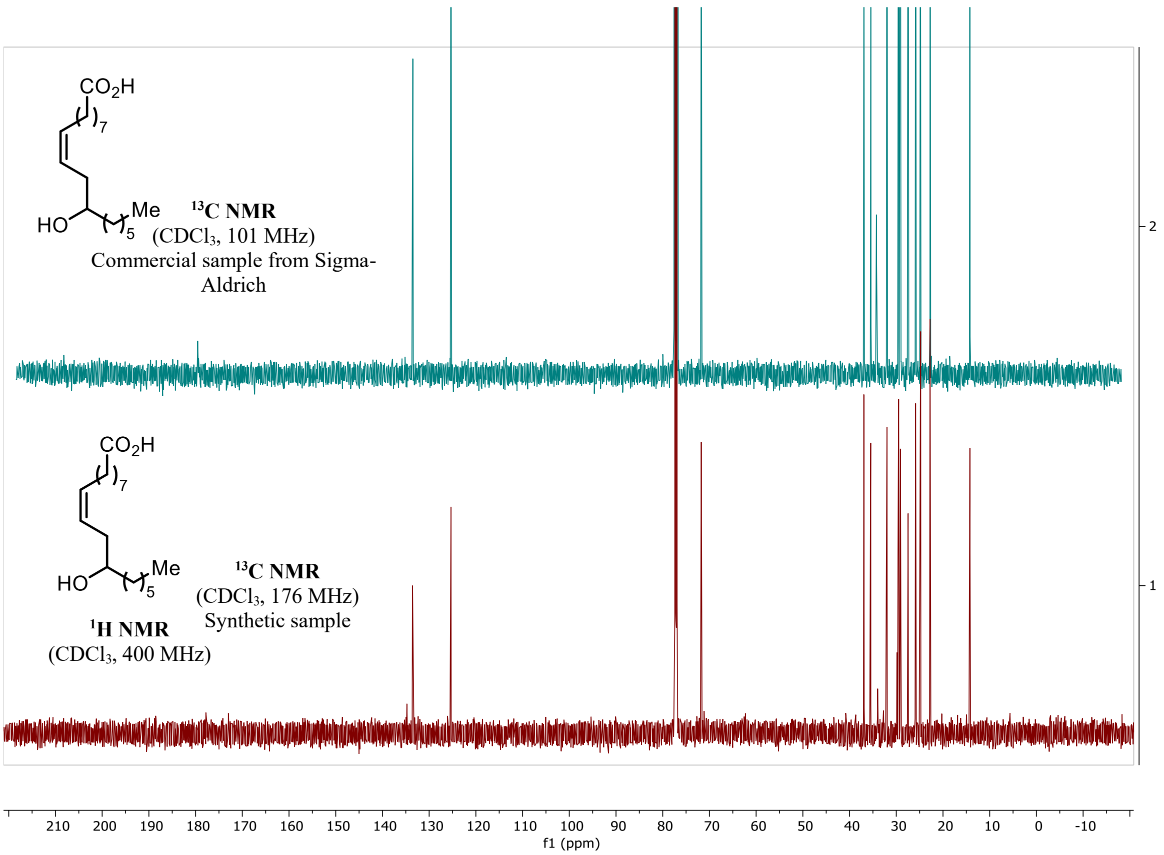

(±)-Lesquerolic acid (10b)

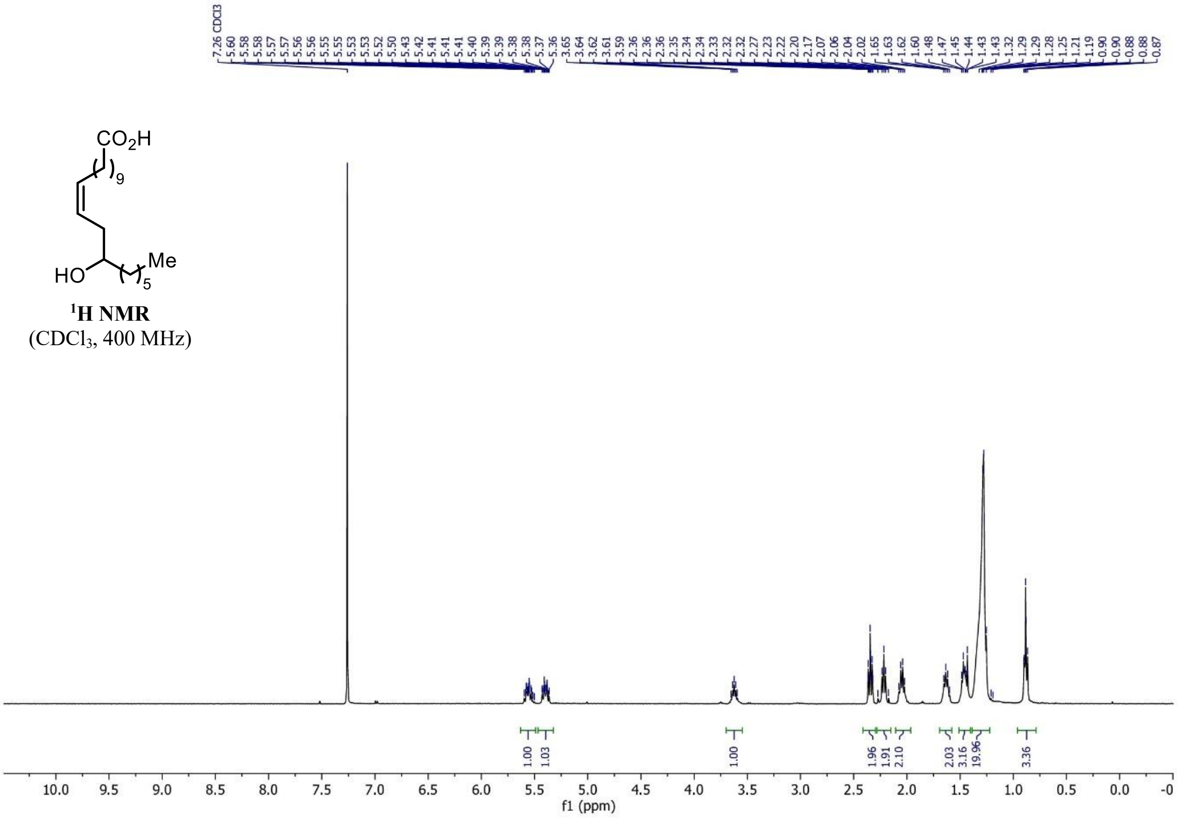

^{1}H NMR
($CDCl_3$, 400 MHz)

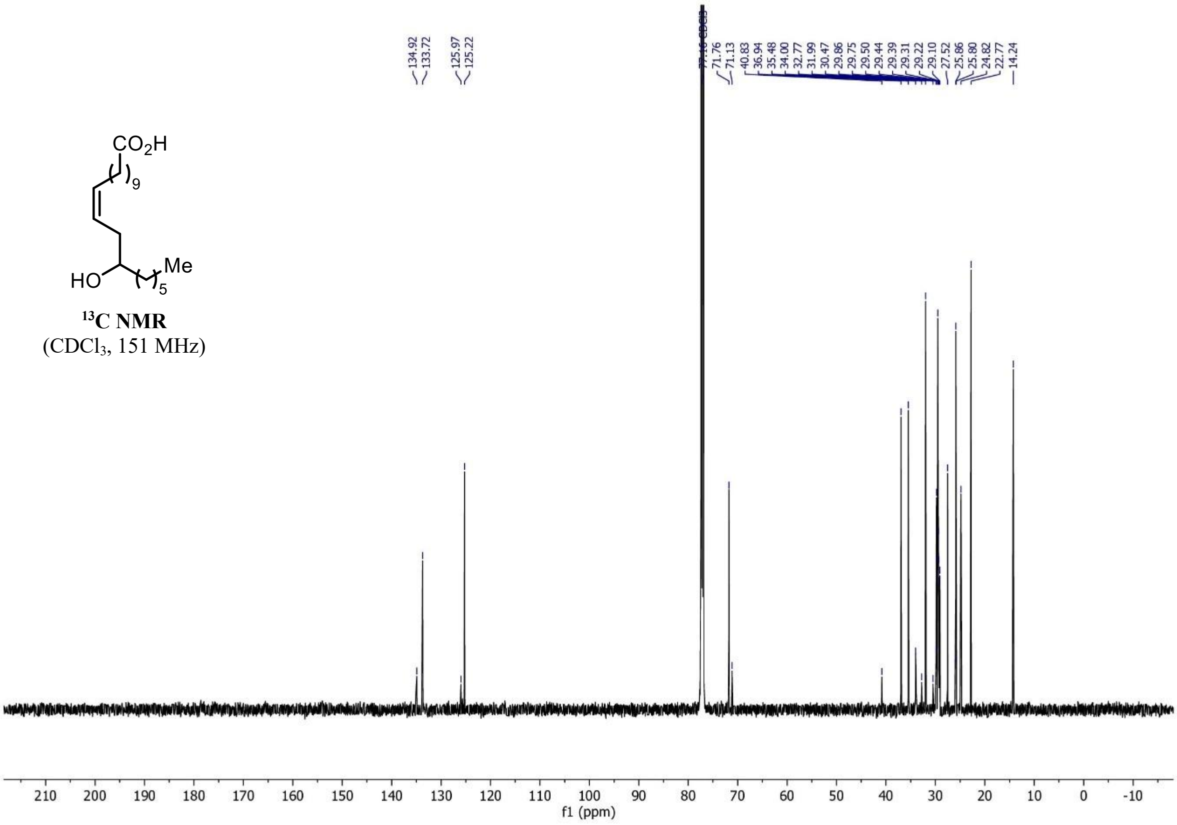

^{13}C NMR
($CDCl_3$, 151 MHz)

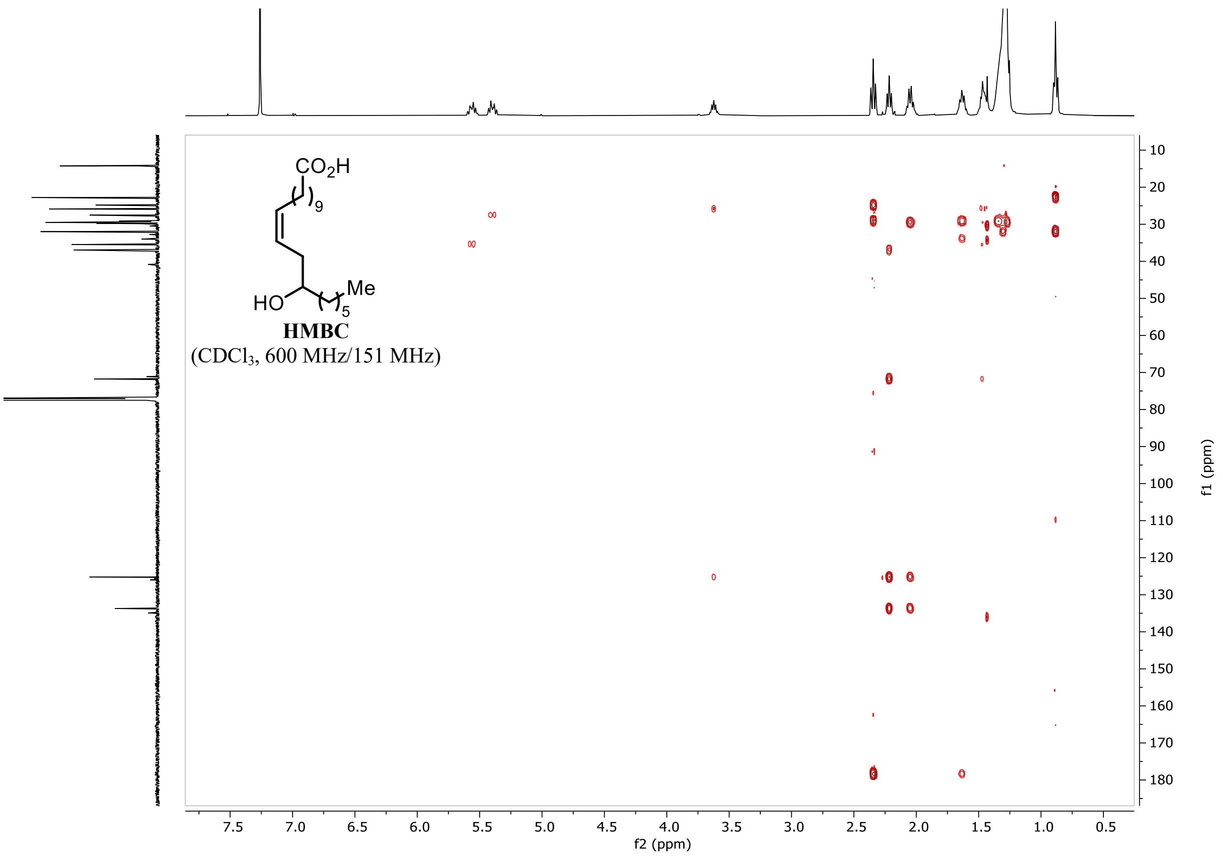
CO2H
9
HO
Me
5
HMBC
(CDCl3, 600 MHz/151 MHz)
f2 (ppm)
f1 (ppm)

(±)-Densipolic acid (10c)

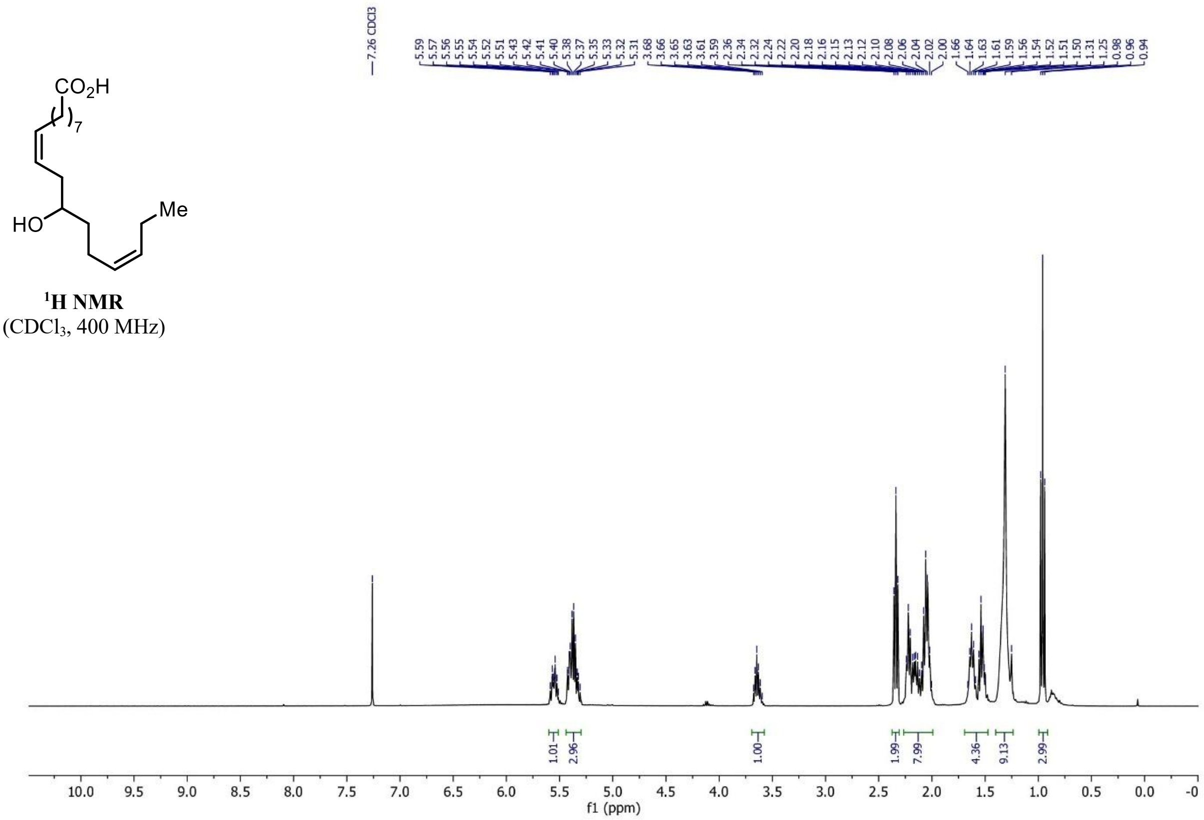

^{1}H NMR
($CDCl_3$, 400 MHz)

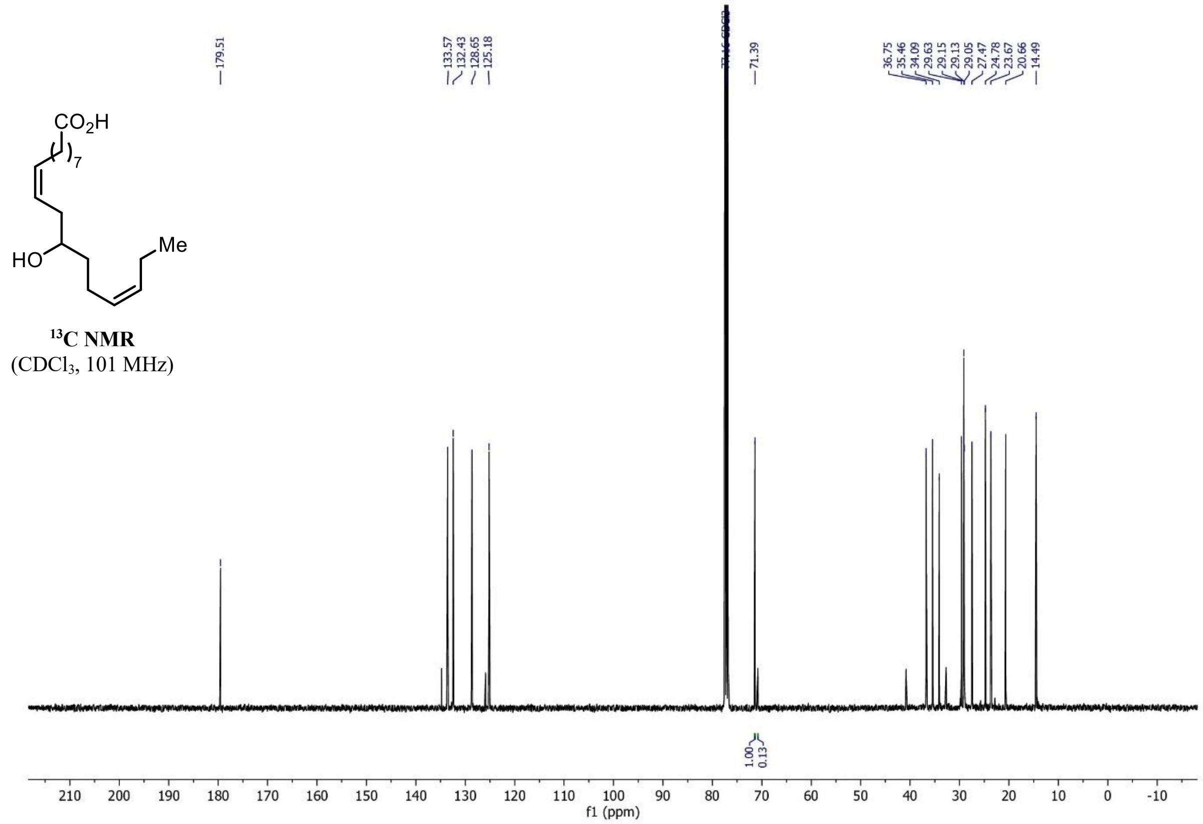

^{13}C NMR
($CDCl_3$, 101 MHz)

(±)-Auricolic acid (10d)

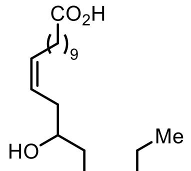

^{1}H NMR
($CDCl_3$, 400 MHz)

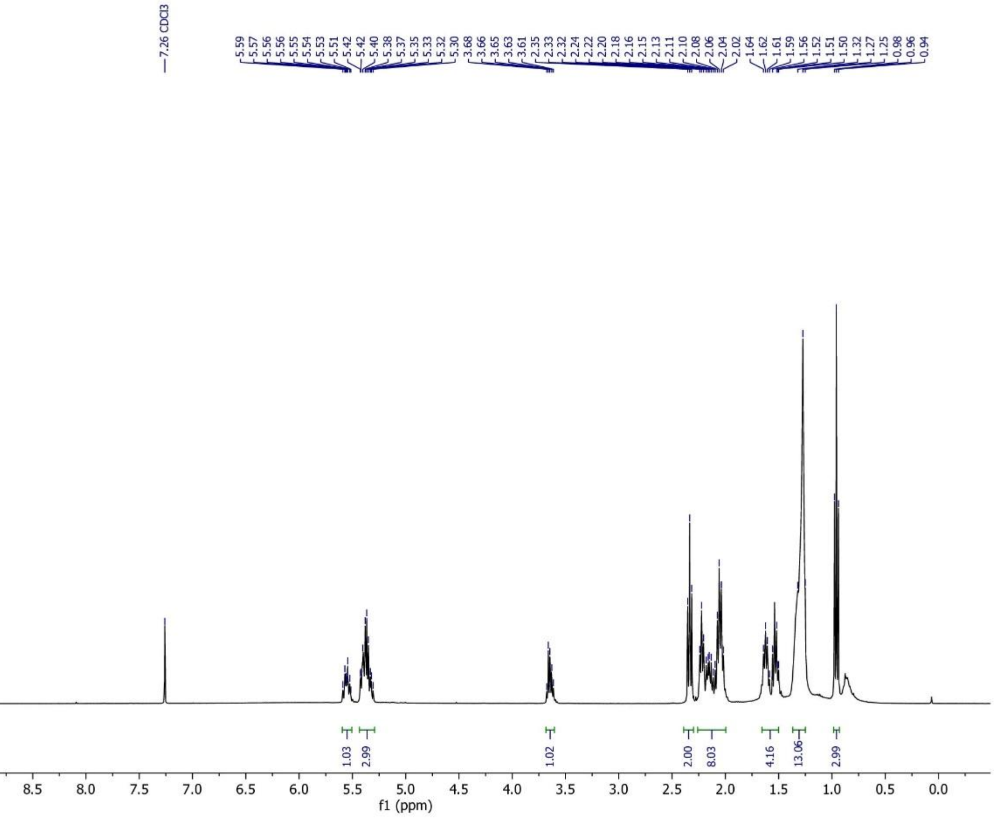

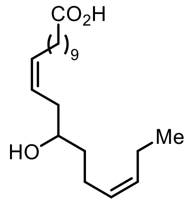

^{13}C NMR
($CDCl_3$, 101 MHz)

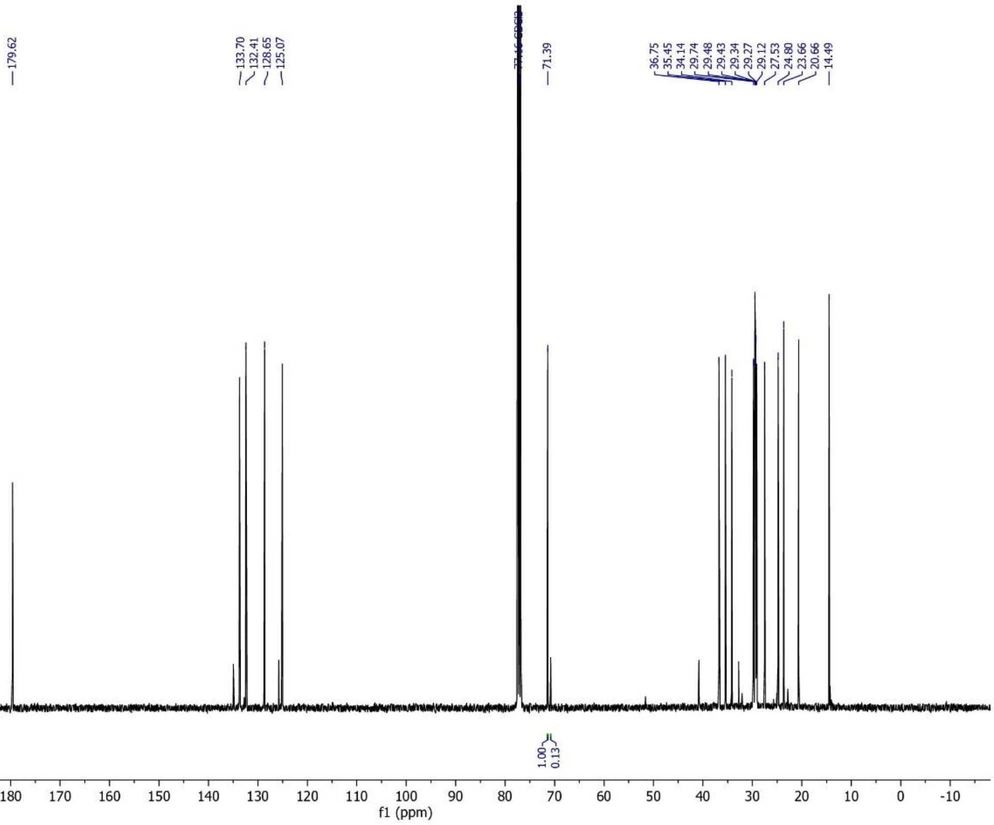

(But-2-yn-1-yloxy)triisopropylsilane (S13)

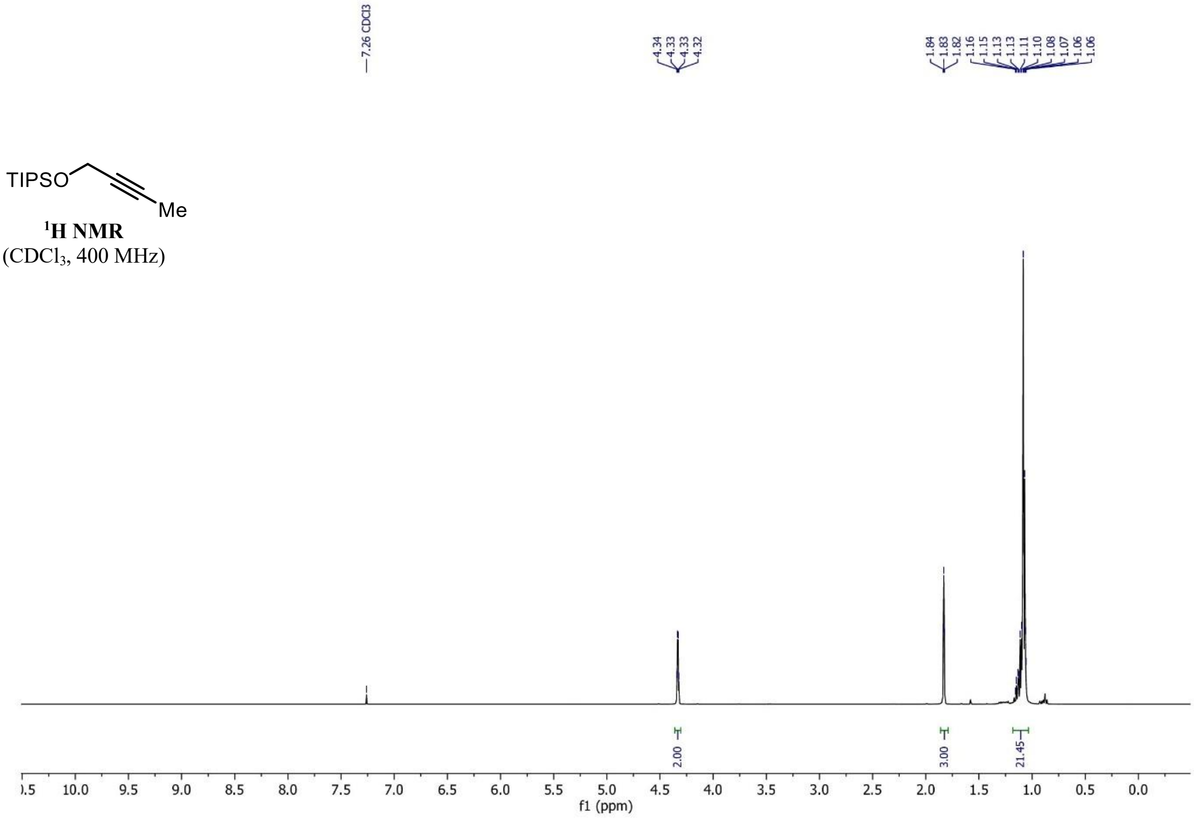

^{1}H NMR
($CDCl_3$, 400 MHz)

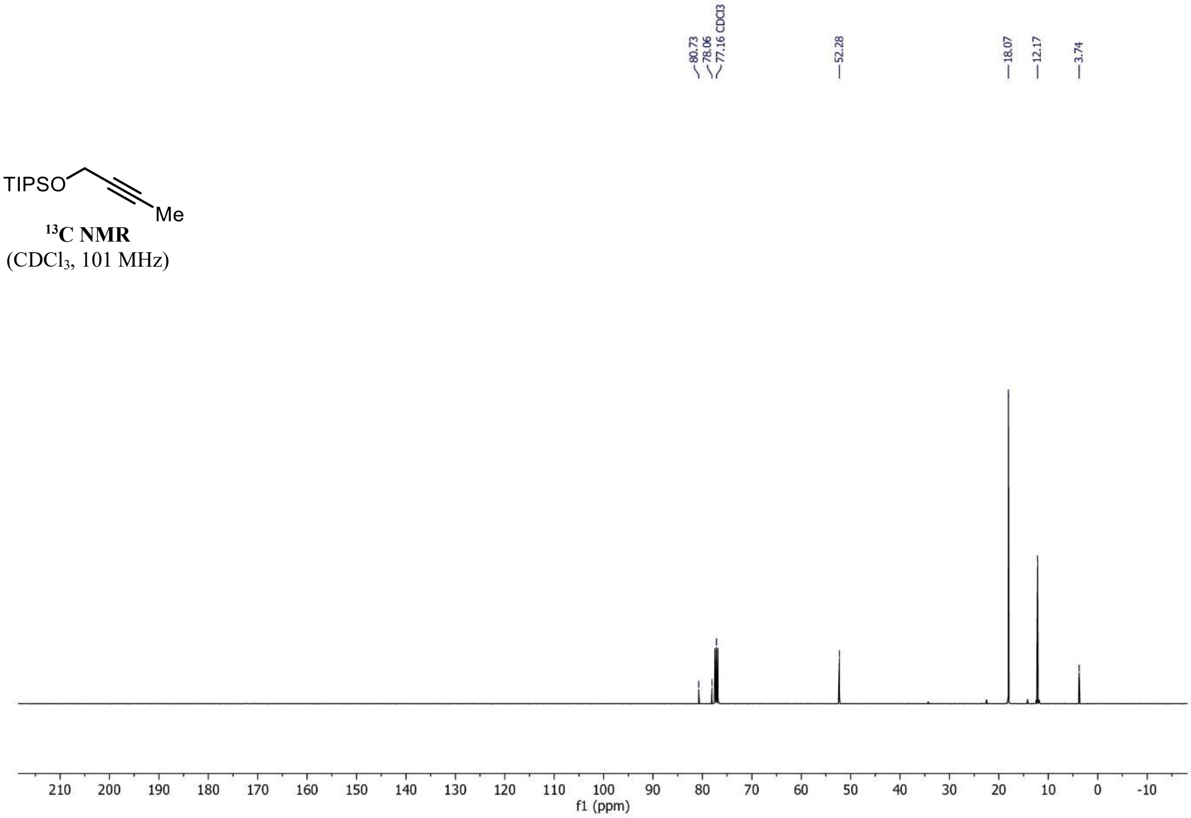

^{13}C NMR
($CDCl_3$, 101 MHz)

4-(Triethylsilyl)pent-4-en-1-ol (S14)

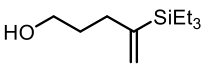

^{1}H NMR
($CDCl_3$, 400 MHz)

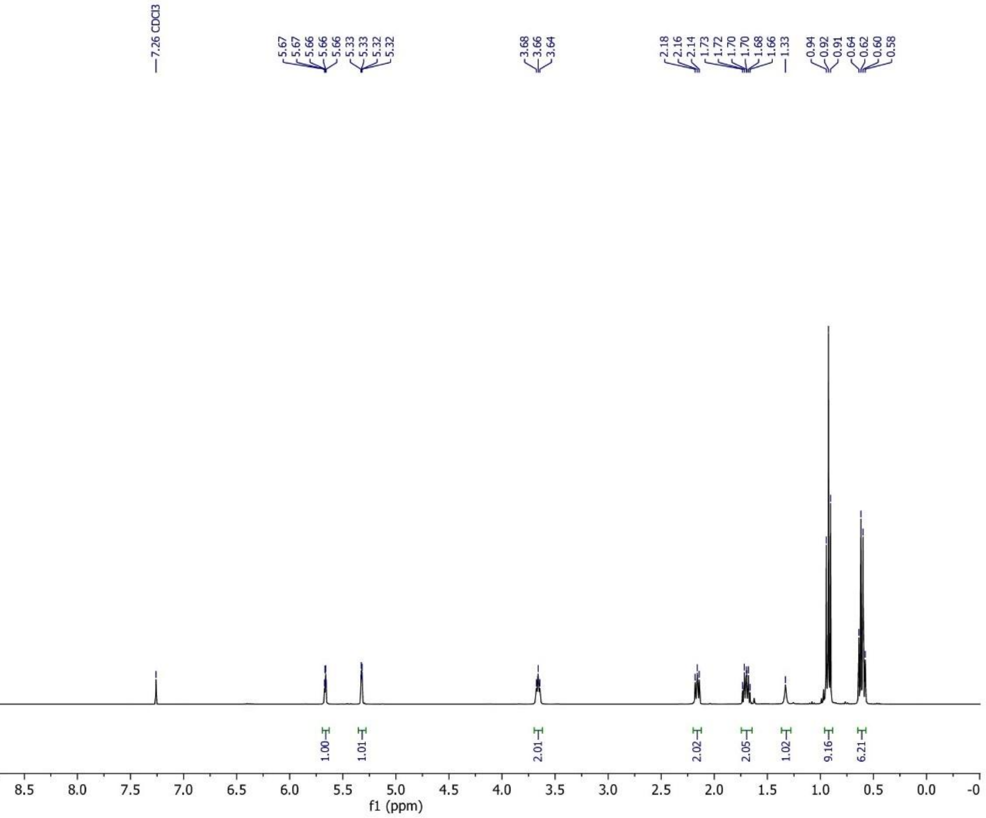

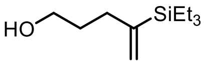

^{13}C NMR
($CDCl_3$, 101 MHz)

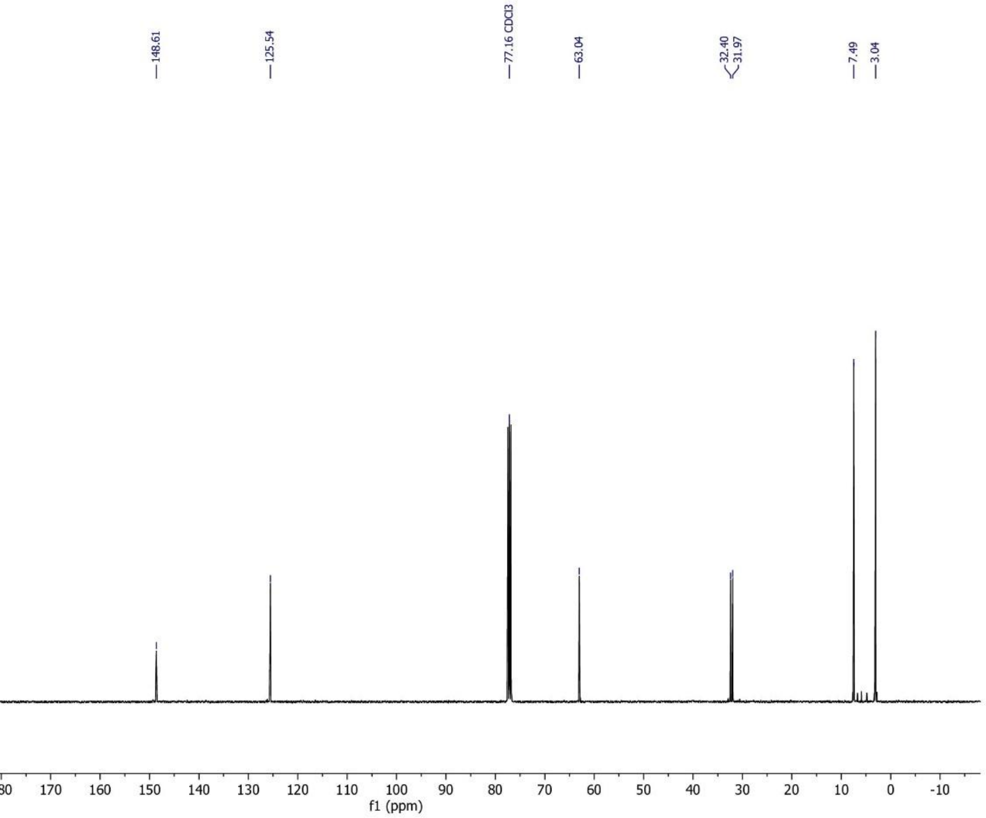

(3*R*,4*S*)-3-Methyl-7-(triethylsilyl)-1-((triisopropylsilyl)oxy)octa-1,7-dien-4-ol (S15)

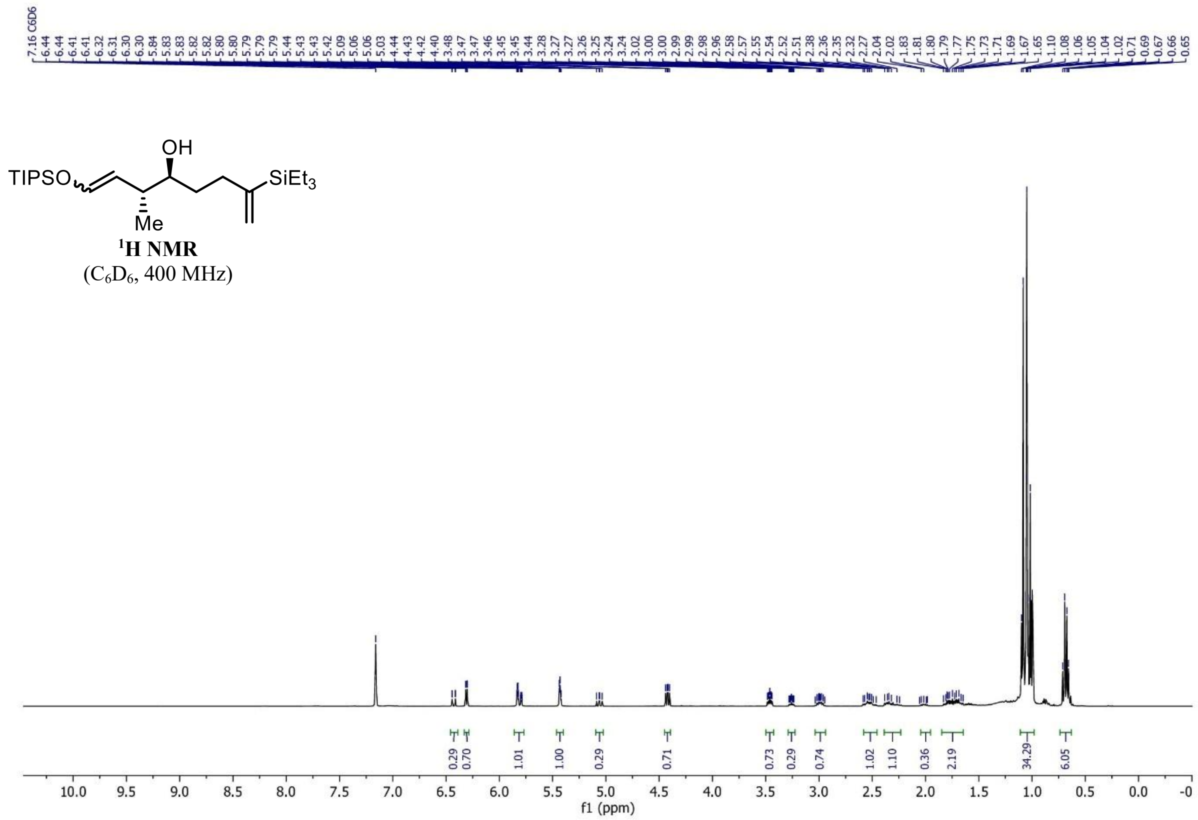

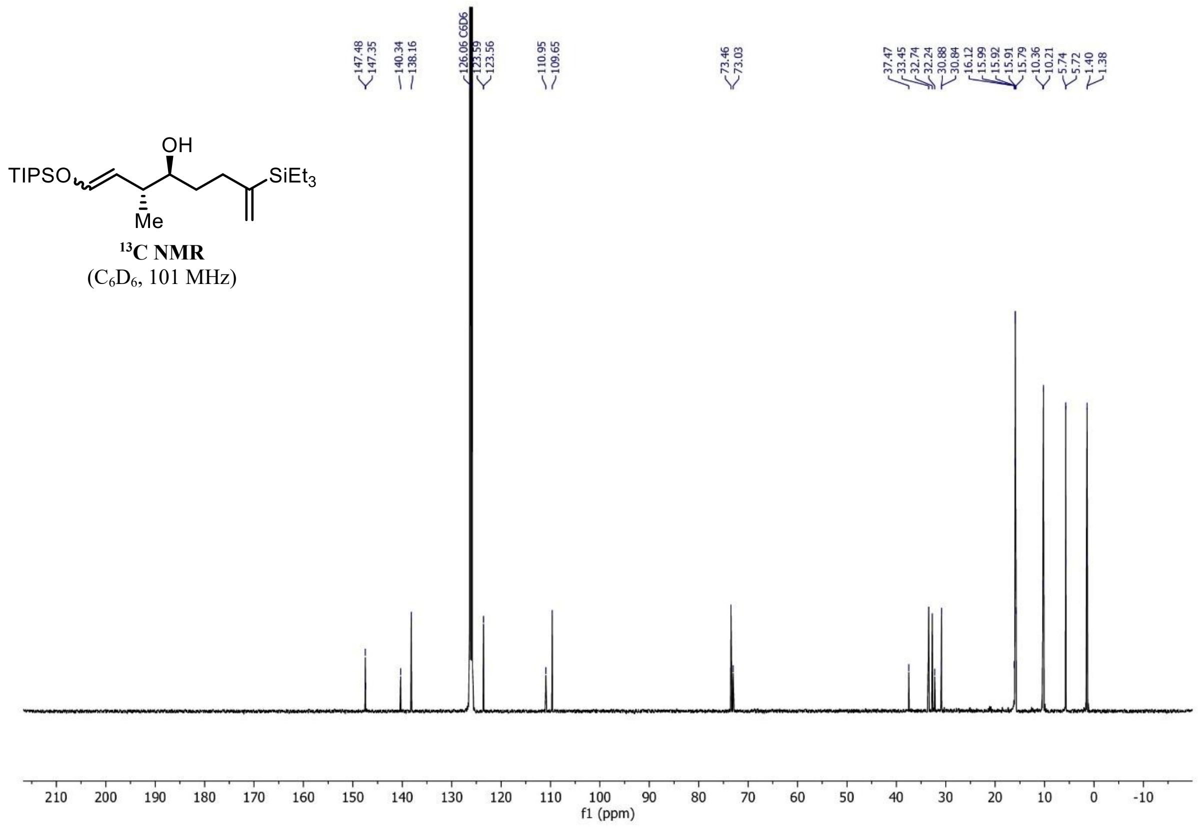

(4*R*,5*S*)-4-Methyl-5-(3-(triethylsilyl)but-3-en-1-yl)dihydrofuran-2(3*H*)-one (7)

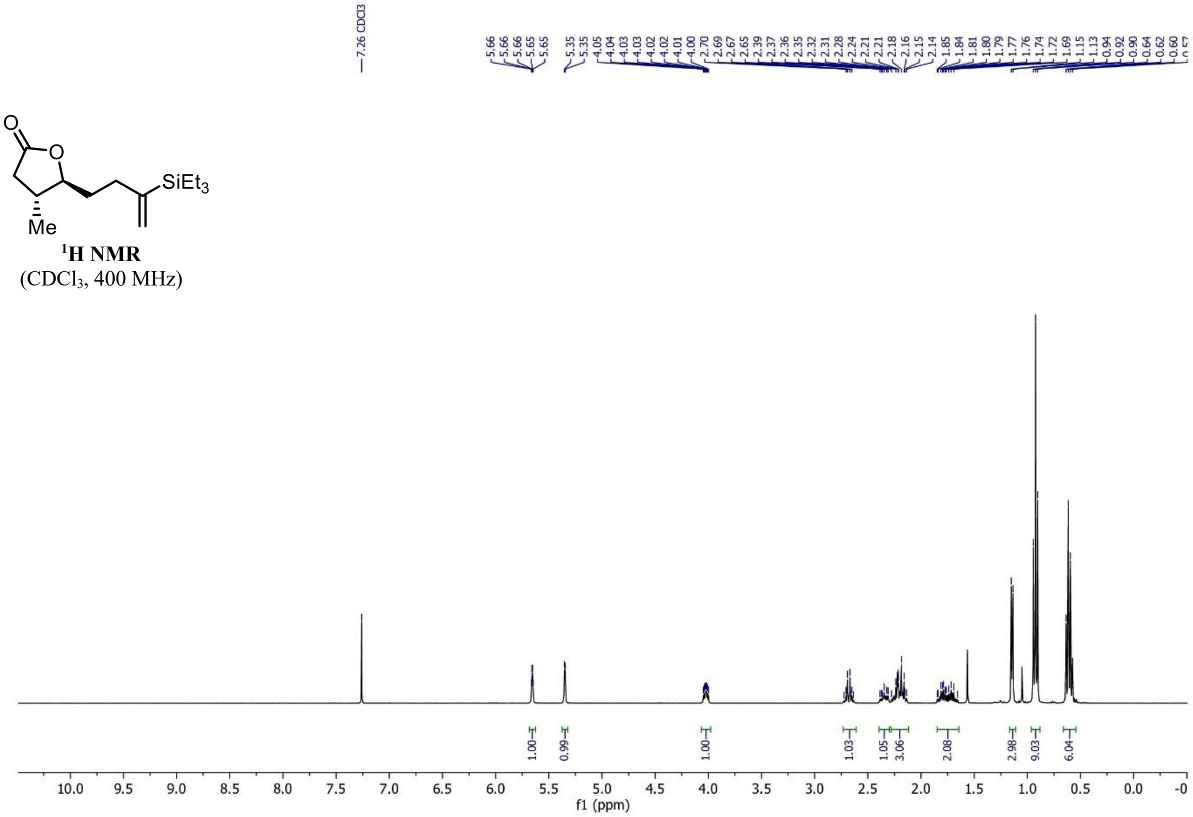

^{1}H NMR
(CDCl$_3$, 400 MHz)

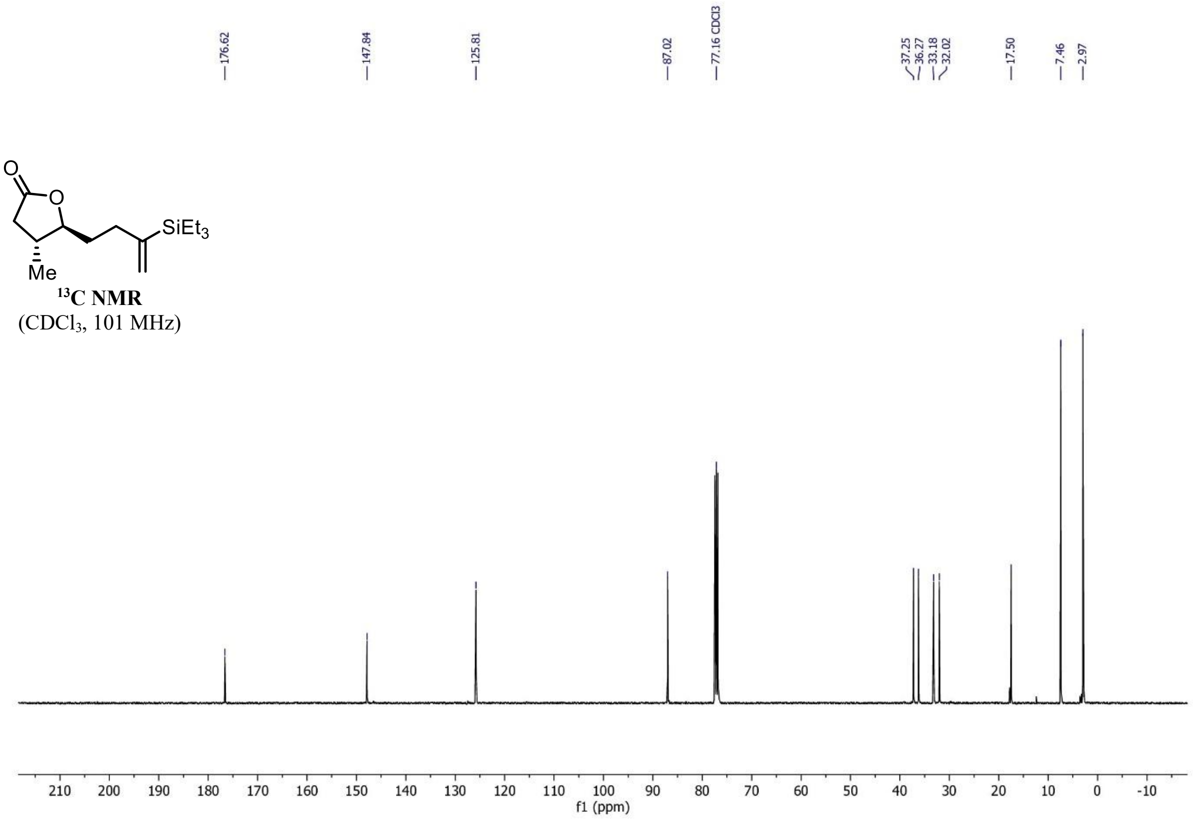

^{13}C NMR
(CDCl$_3$, 101 MHz)

(4*R*,5*S*)-4-Methyl-5-((*Z*)-5-oxooct-2-en-1-yl)dihydrofuran-2(3*H*)-one (8)

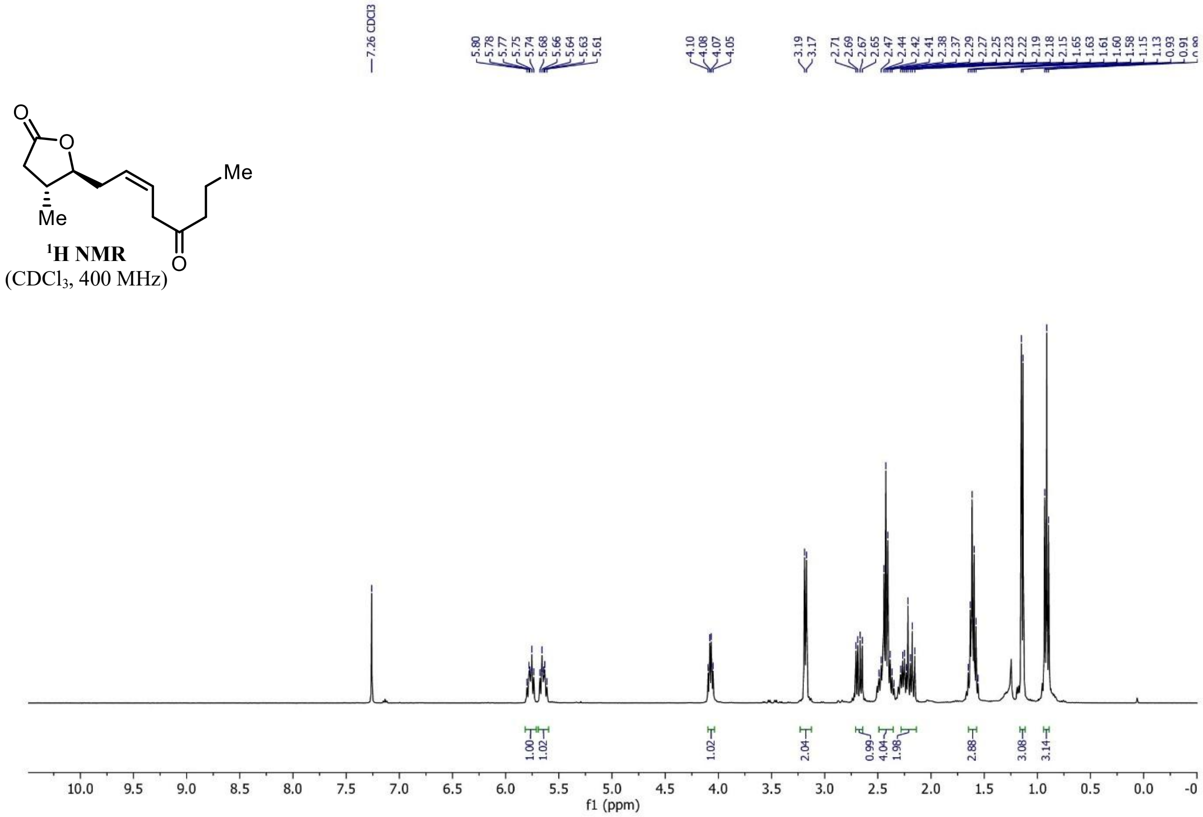

^{1}H NMR
($CDCl_3$, 400 MHz)

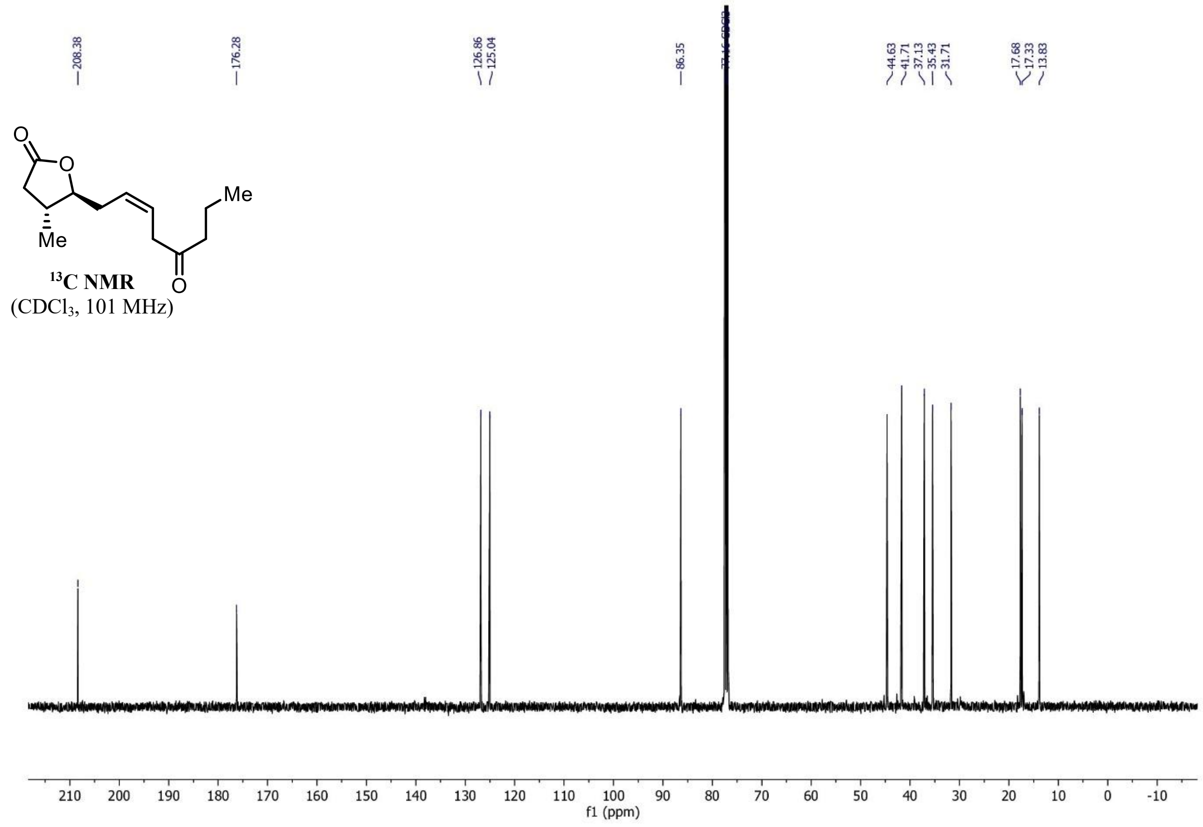

^{13}C NMR
($CDCl_3$, 101 MHz)

(4*R*,5*S*)-4-methyl-5-((*Z*)-5-oxo-5-phenylpent-2-en-1-yl)dihydrofuran-2(3*H*)-one (9)

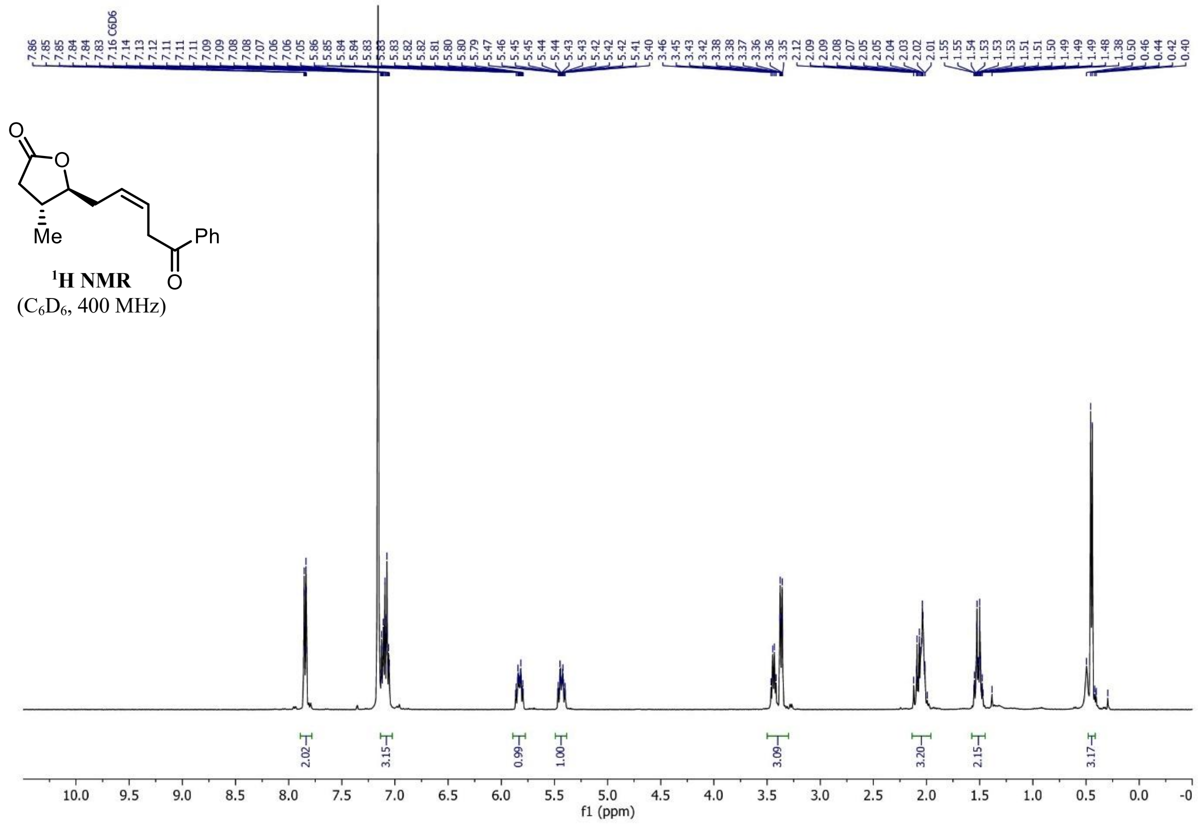

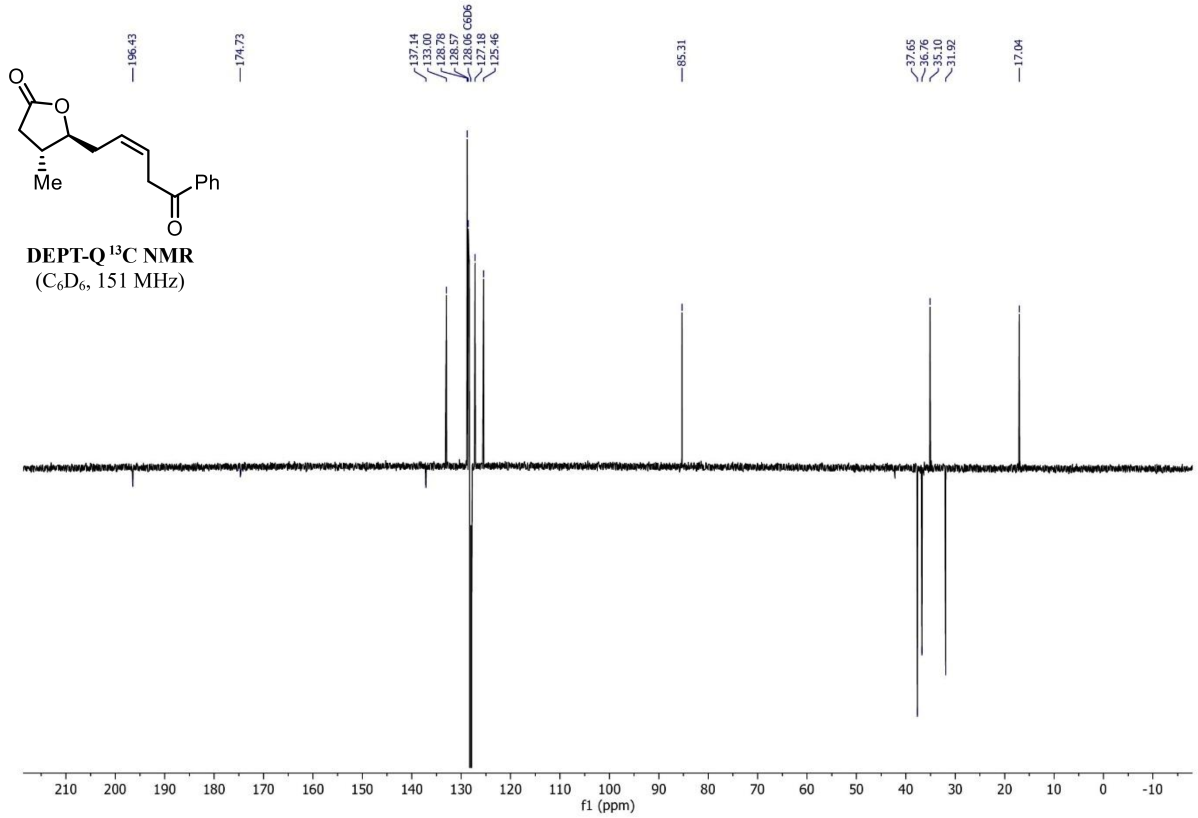

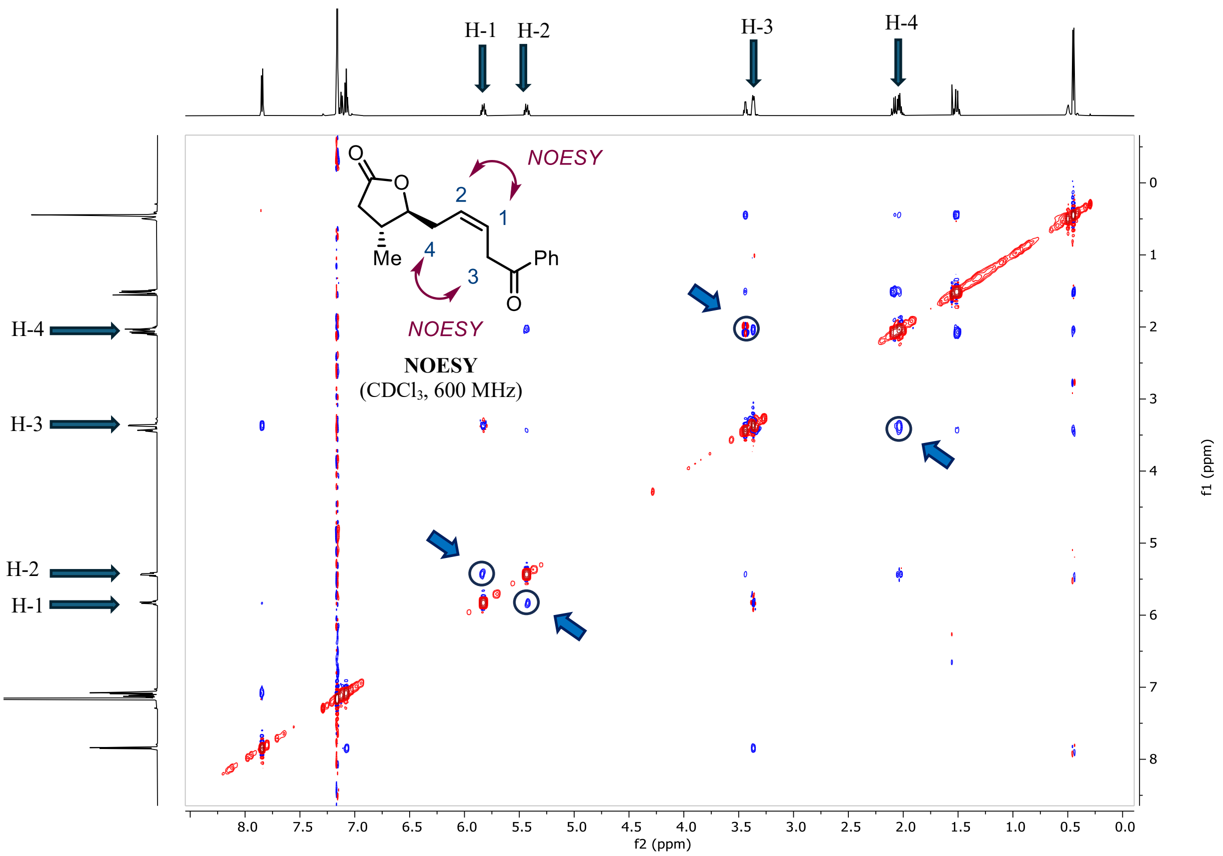
H-1
H-2
H-3
H-4
NOESY
NOESY
NOESY
(CDCl3, 600 MHz)
Me
Ph
f2 (ppm)
f1 (ppm)

(±)-Triethyl((2*S*,3*S*,5*S*)-2-(2-iodoethyl)-5-methyl-5-phenyltetrahydrofuran-3-yl)silane (11)

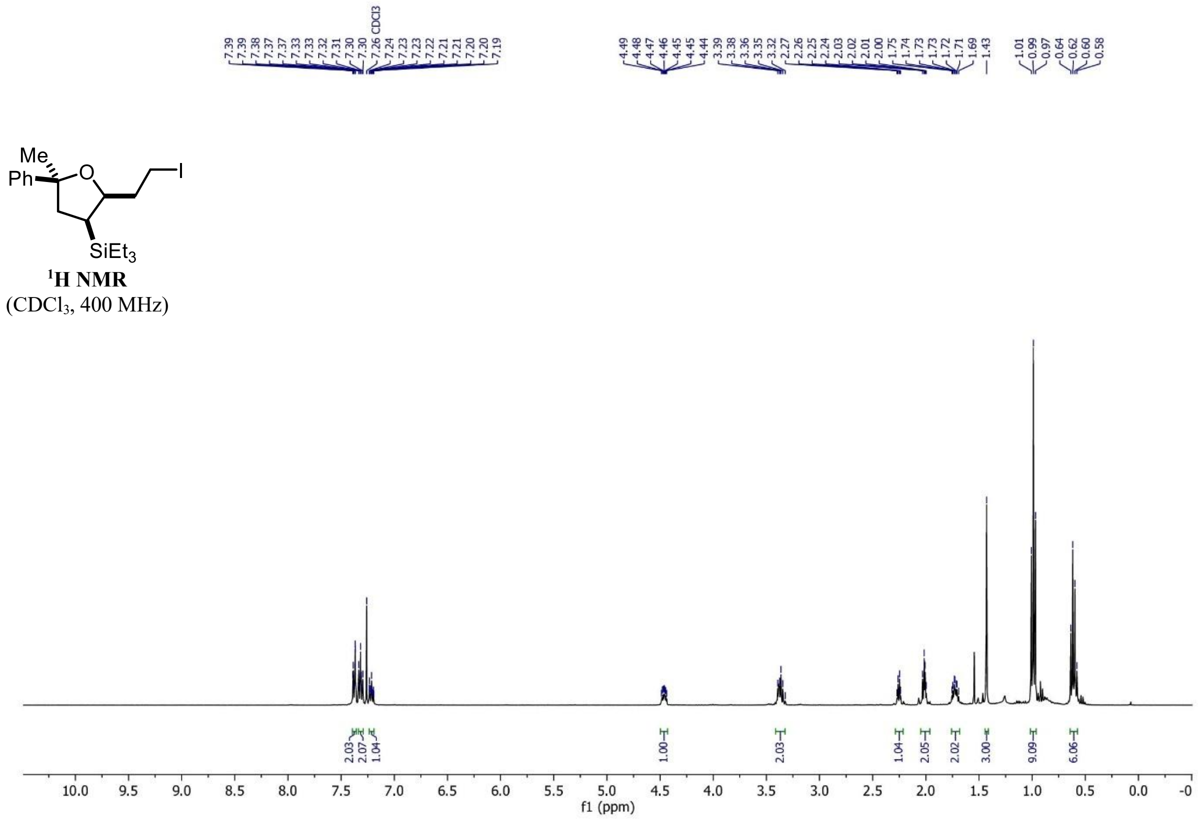

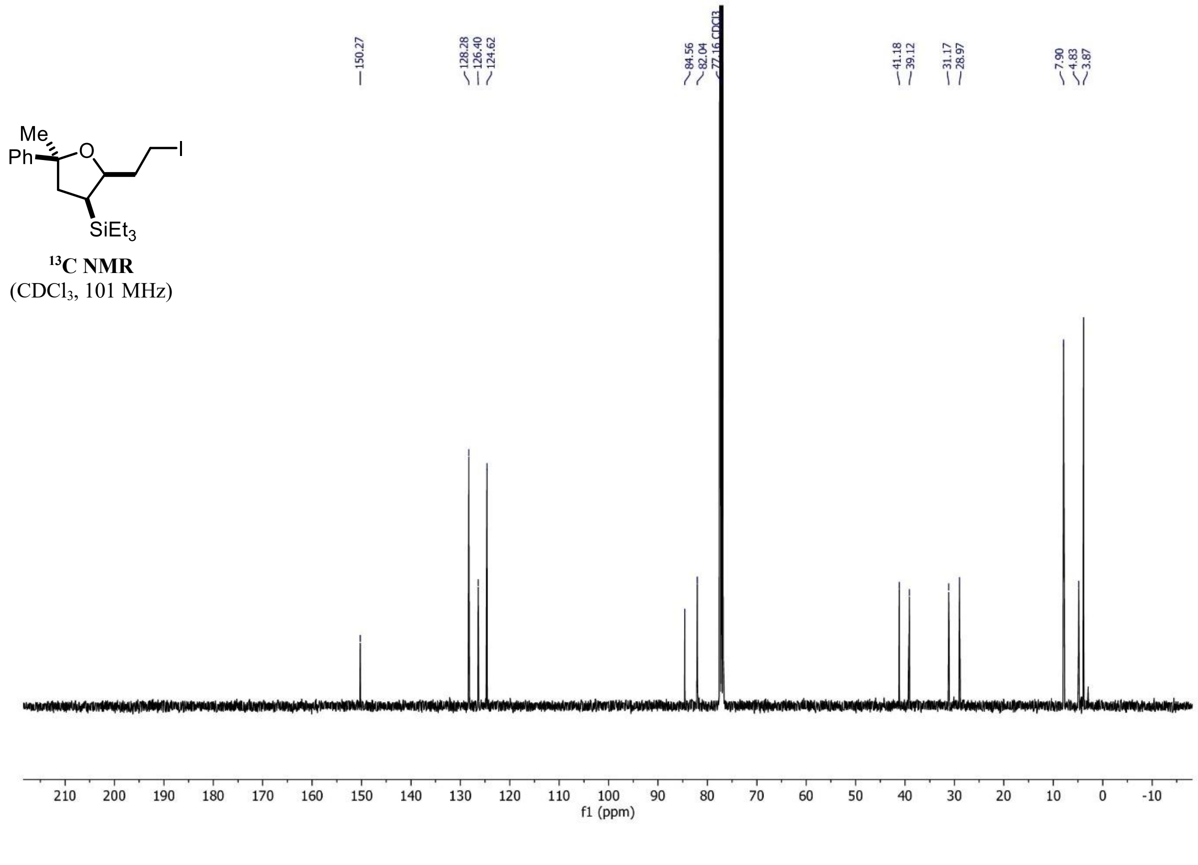

(*E*)-6-Iodo-1-phenyl-3-(triethylsilyl)hex-3-en-1-one (11’)

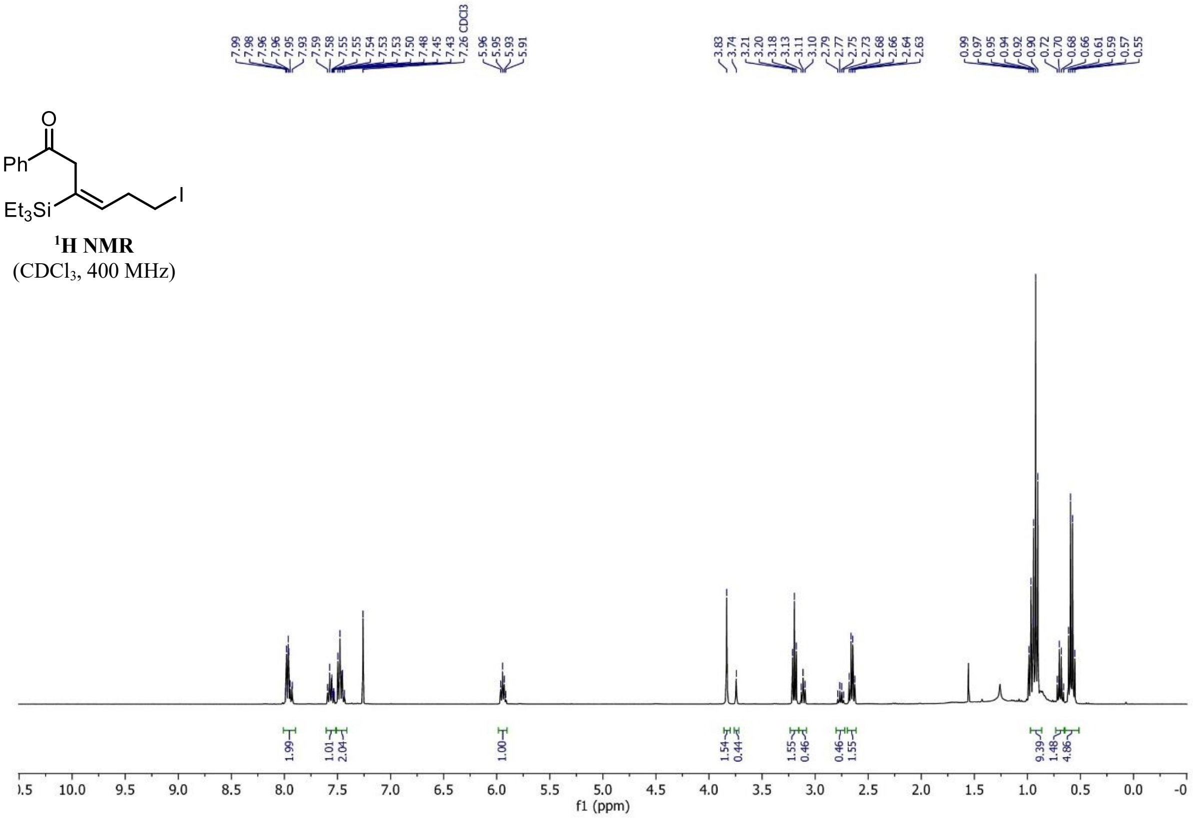

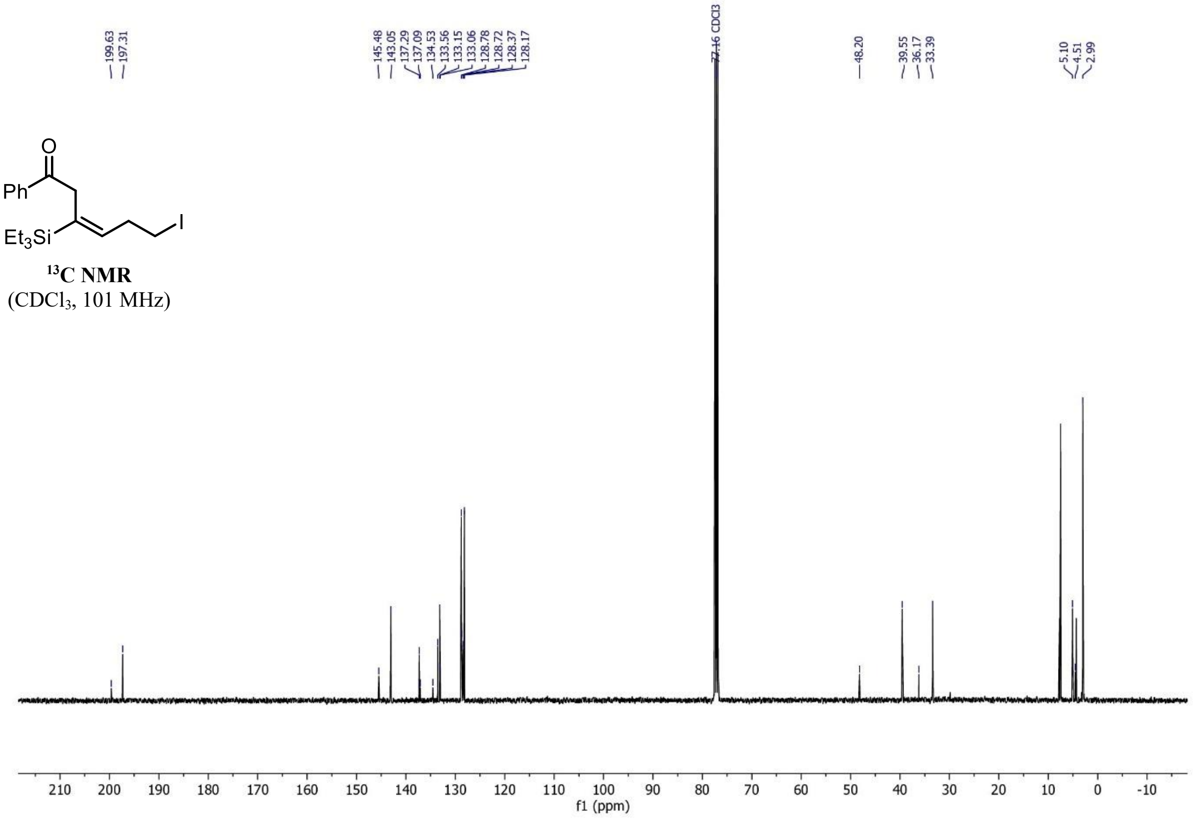

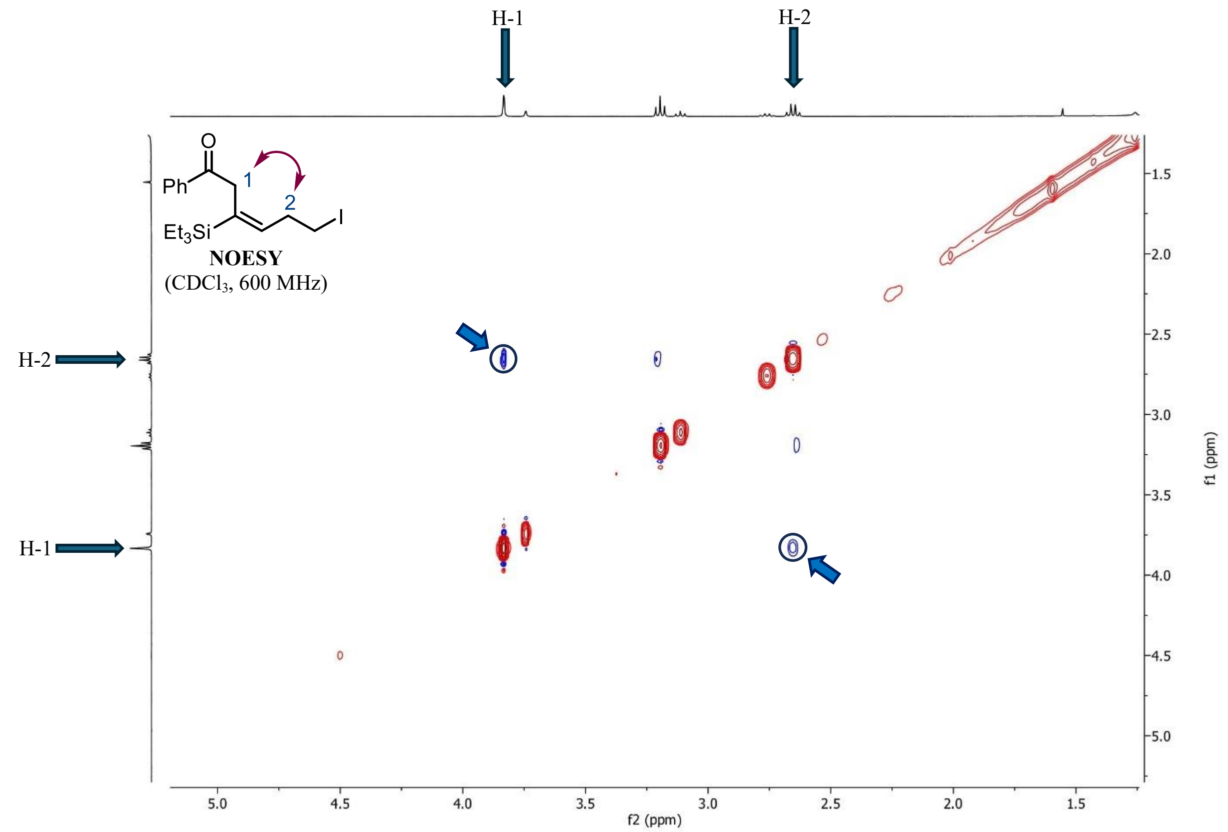

H-1
H-2
Ph
O
1
2
Et3Si
I
NOESY
(CDCl3, 600 MHz)
H-2
H-1
f1 (ppm)
f2 (ppm)

(±)-2-((2*S*,3*S*,5*S*)-5-Methyl-5-phenyl-3-(triethylsilyl)tetrahydrofuran-2-yl)-N-(pyridin-2-ylmethyl)ethan-1-amine (12·2HCl)

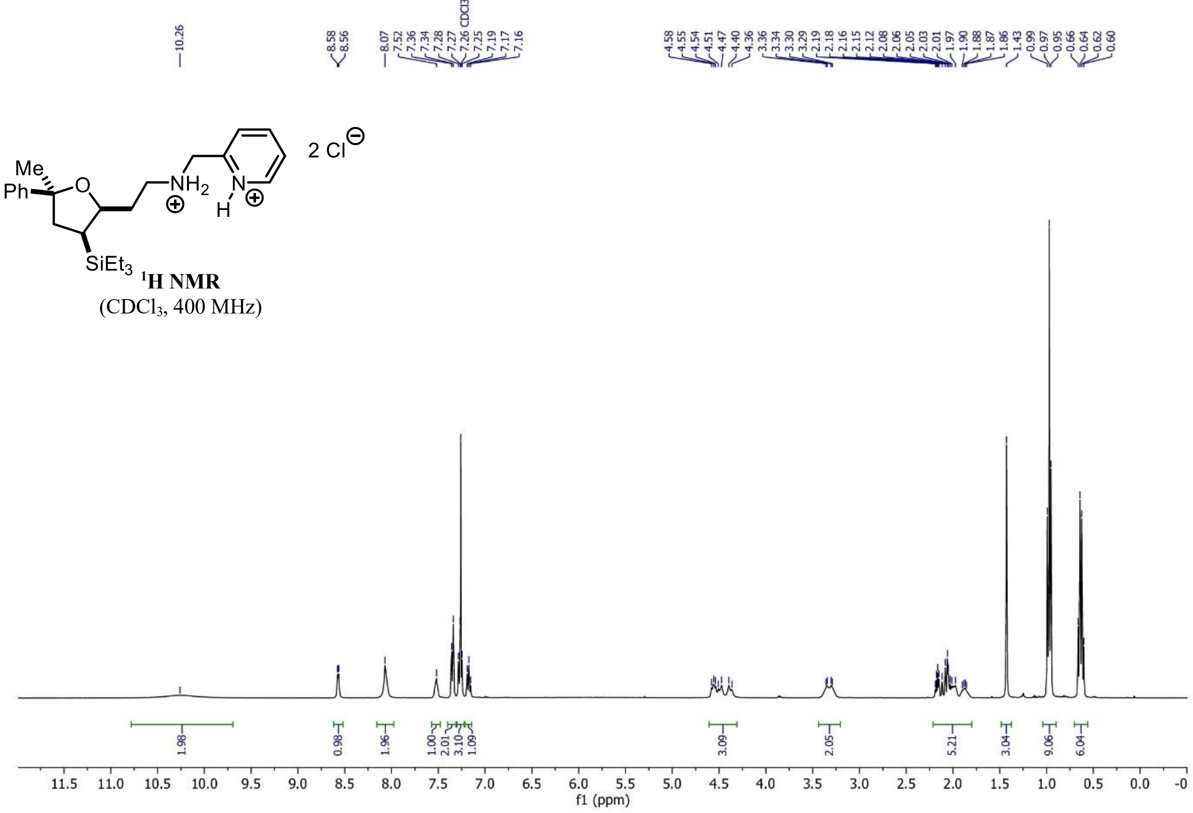

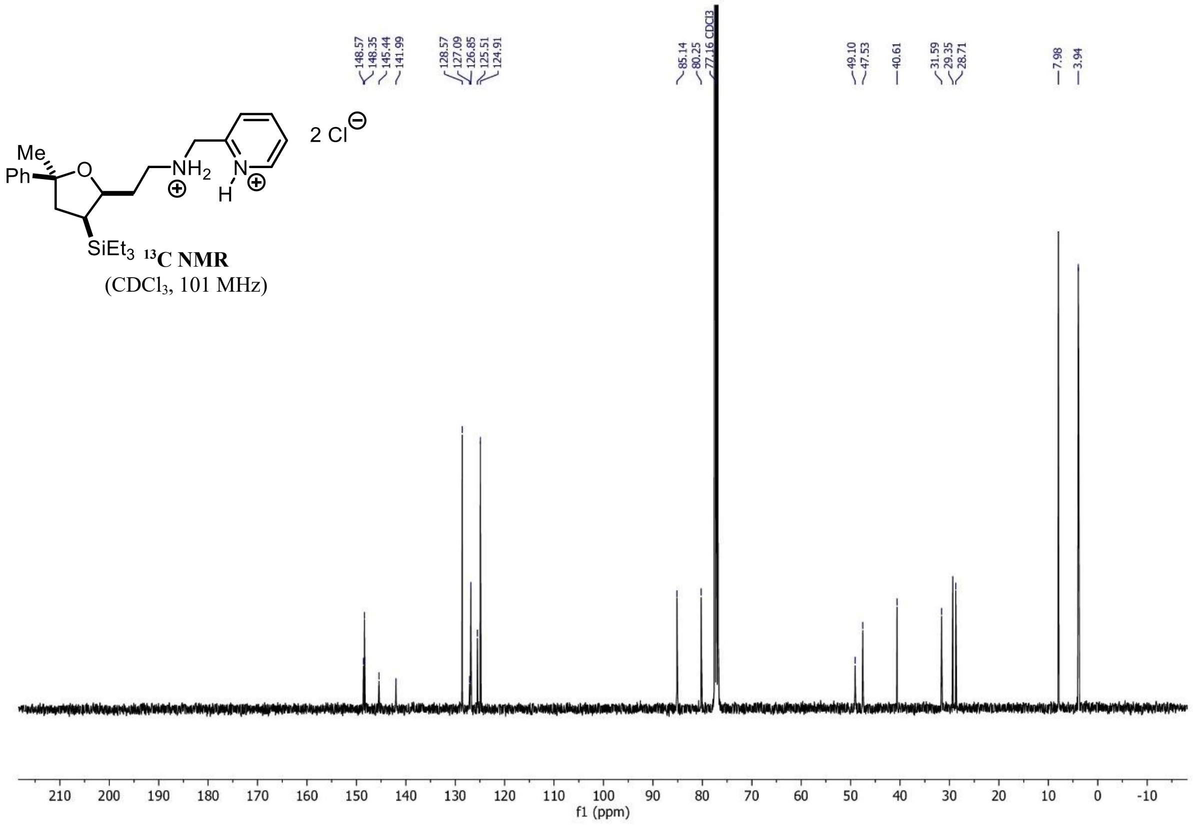

(±)-(2-Benzyl-5-methyl-5-phenyltetrahydrofuran-3-yl)triethylsilane (S16)

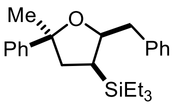

¹H NMR
($CDCl_3$, 400 MHz)

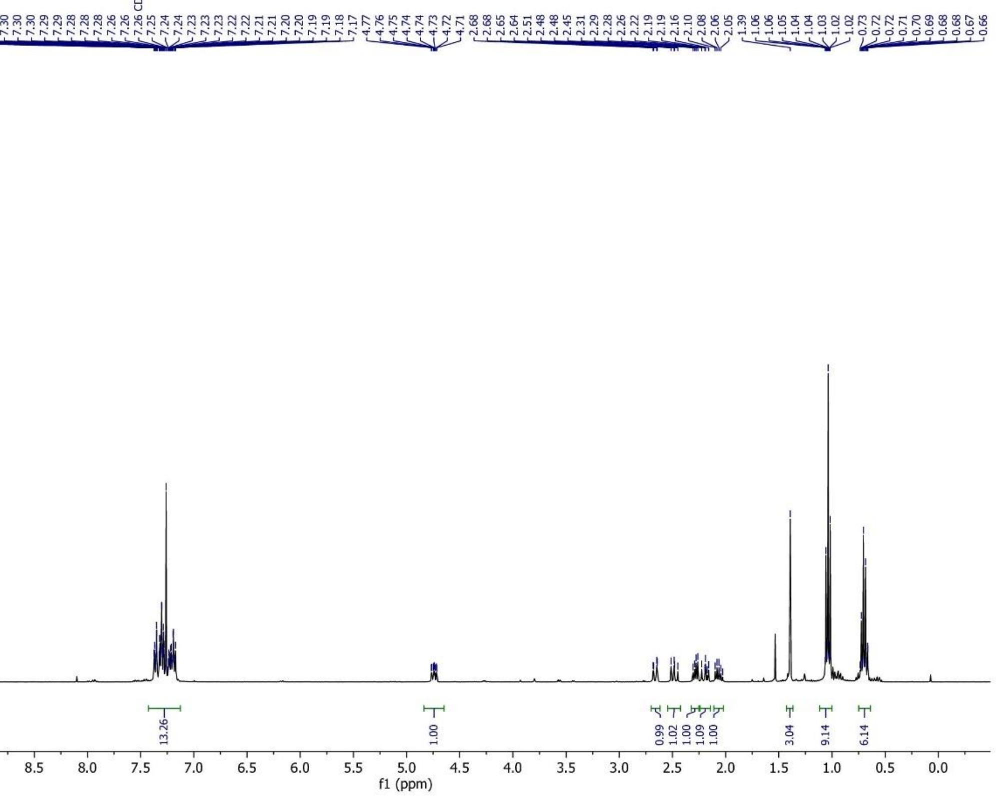

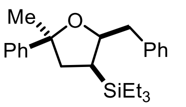

¹³C NMR
($CDCl_3$, 101 MHz)

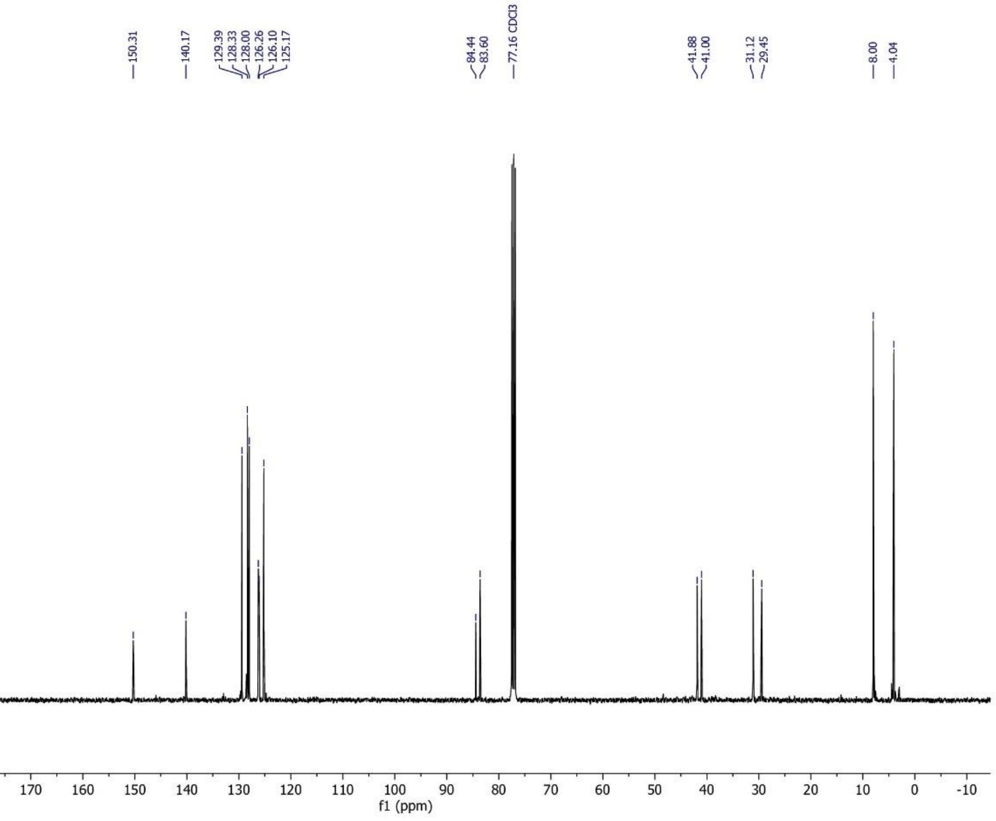

(±)- (2-Benzyl-5-(4-methoxyphenyl)-5-methyltetrahydrofuran-3-yl)triethylsilane (S17)

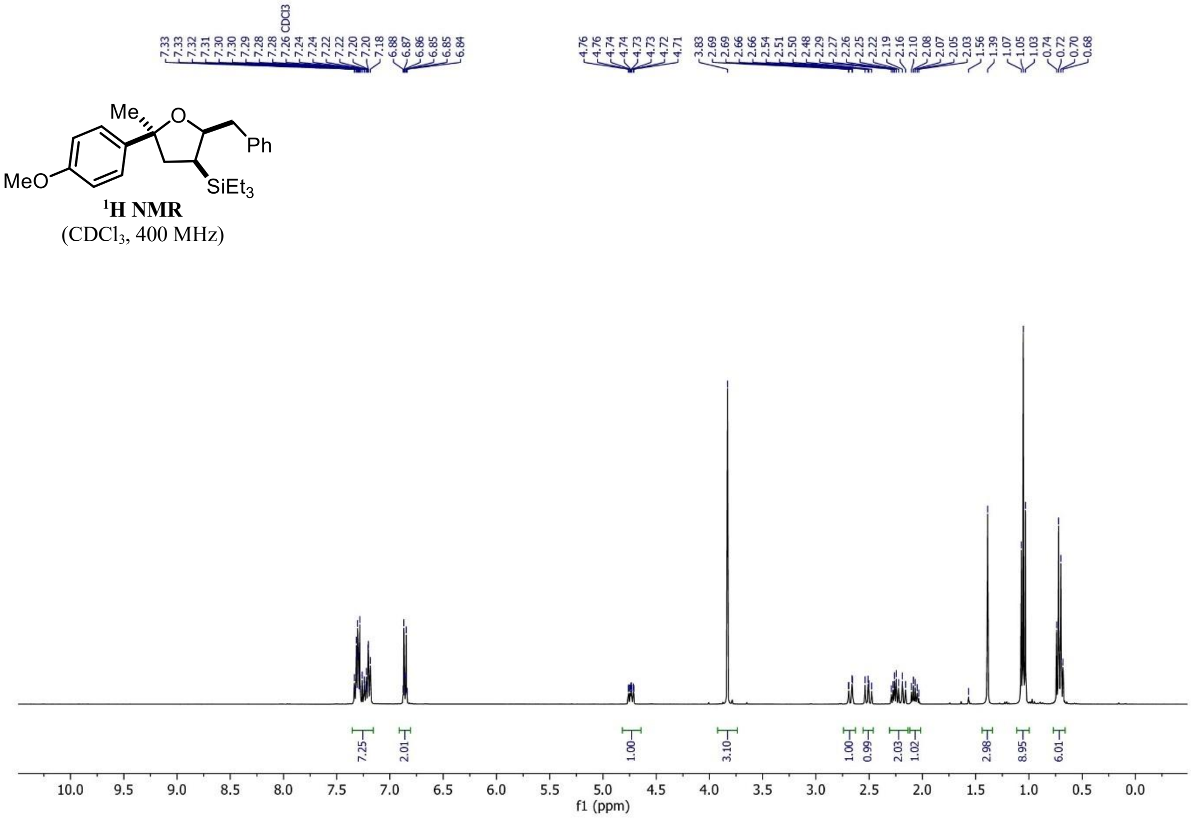

^{1}**H NMR**
($CDCl_3$, 400 MHz)

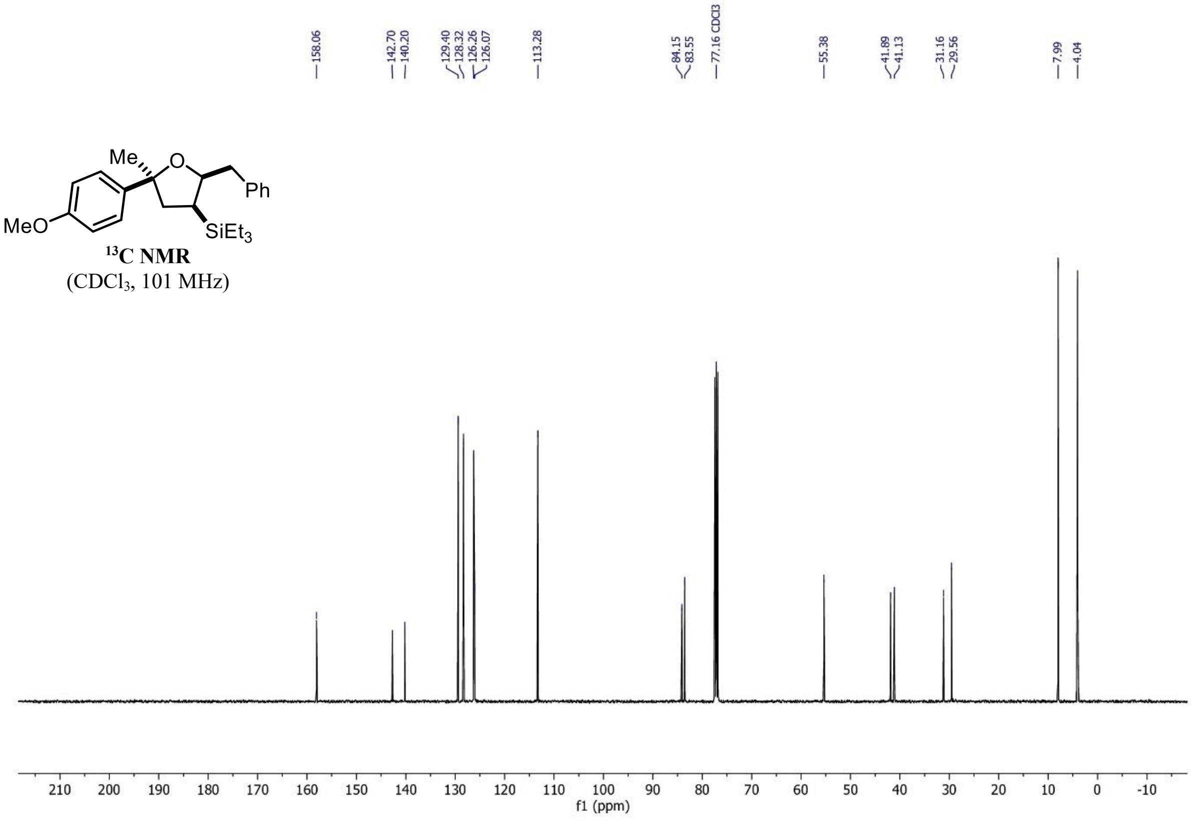

^{13}**C NMR**
($CDCl_3$, 101 MHz)

Analysis of the crude mixture obtained from the reaction of vinyl silane 2c and benzoyl chloride to determine the silicon-containing byproduct

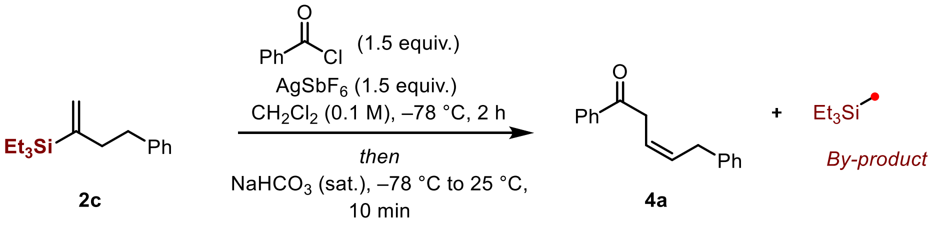

Analysis of the crude NMR mixture

From the crude mixture: ^{1}H NMR (400 MHz, CD_2Cl_2) δ 0.94 (t, *J* = 7.9 Hz), 0.54 (q, *J* = 7.9 Hz) ppm.

<u>Literature data</u> for 1,1,1,3,3,3-hexaethyldisiloxane $(Et_3Si)_2O$ for comparison:[80]

^{1}H NMR (400 MHz, CD_2Cl_2) δ 0.94 (t, *J* = 7.9 Hz, 18H), 0.53 (q, *J* = 7.9 Hz, 12H) ppm.

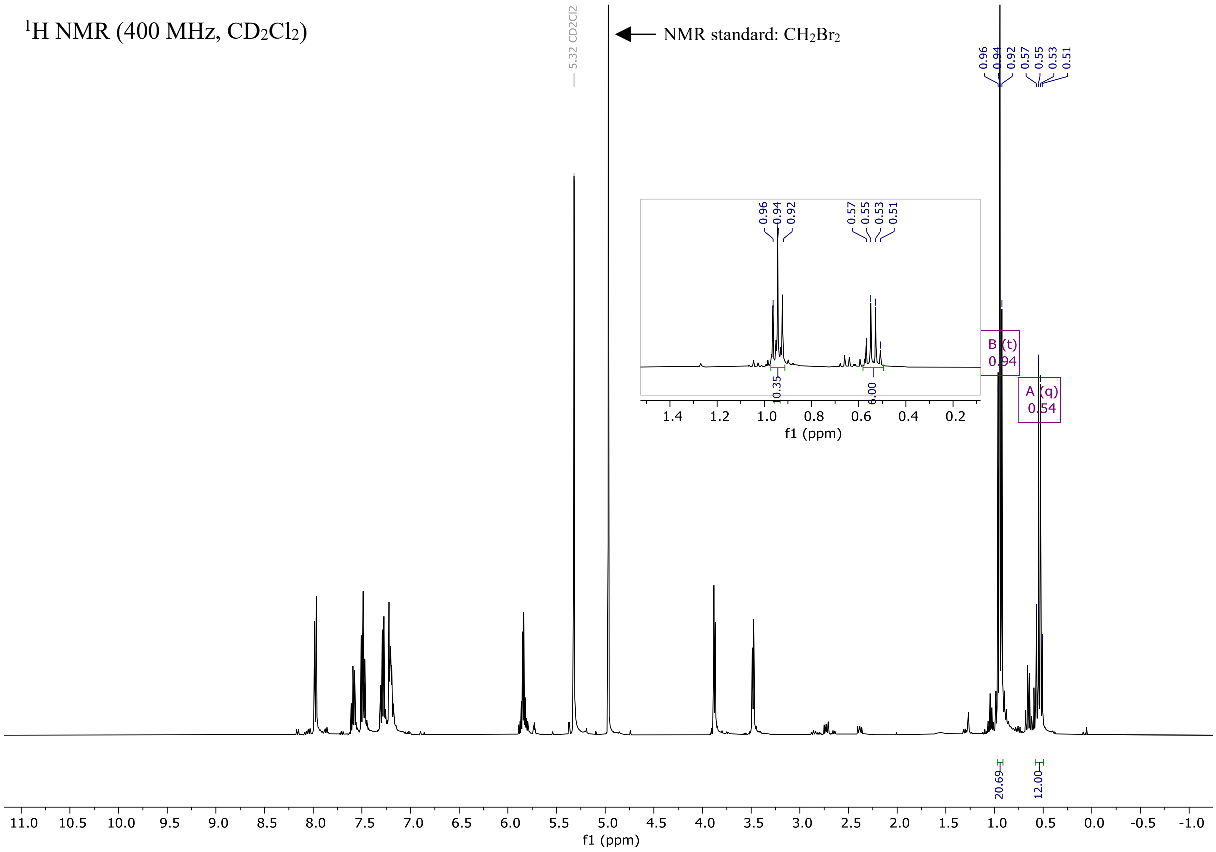

From the crude mixture: ^{29}Si NMR (119 MHz, CD_2Cl_2) δ 8.88 ppm.

<u>Literature data</u> for 1,1,1,3,3,3-hexaethyldisiloxane $(Et_3Si)_2O$ for comparison:[81]

^{29}Si{^{1}H} (79.5 MHz, CD_2Cl_2, 298 K): δ 8.9 ppm.

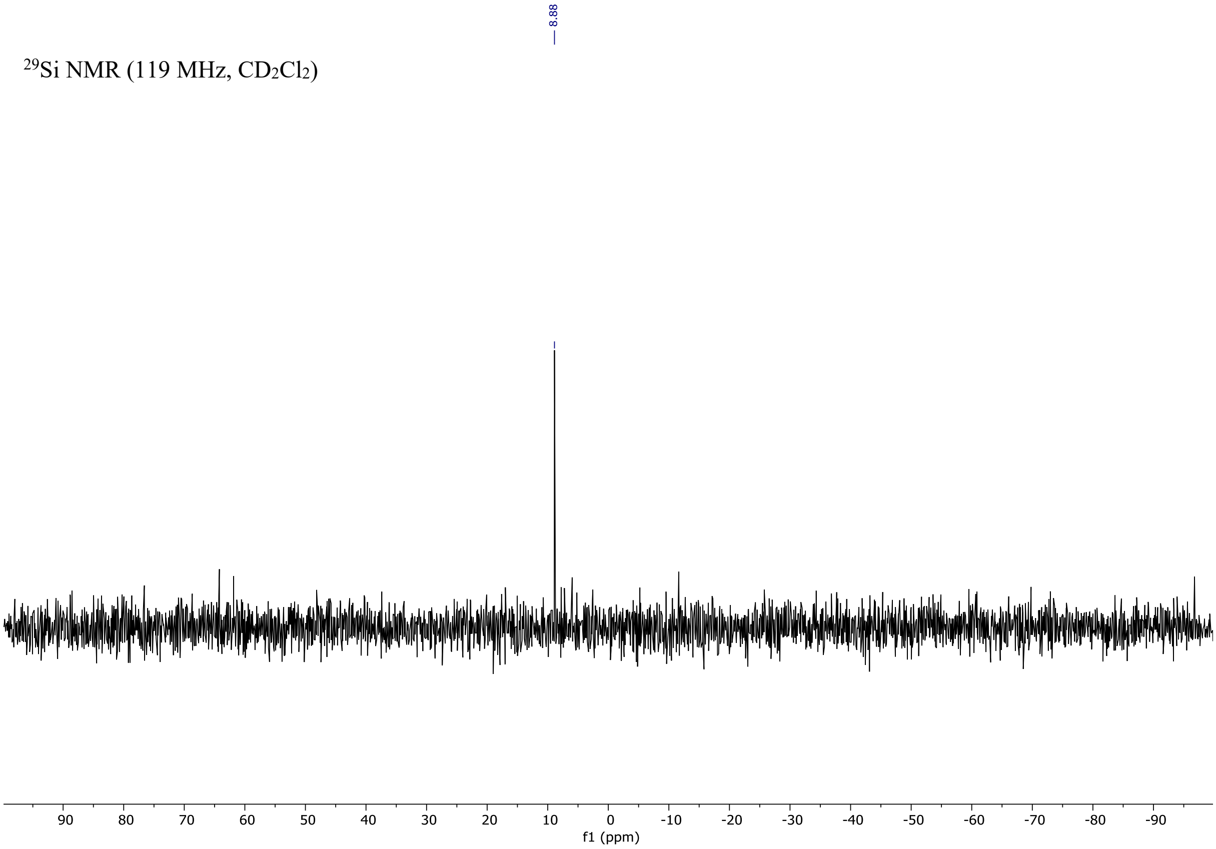

From the crude mixture: ^{13}C NMR (101 MHz, CD_2Cl_2) δ 6.99, 6.76 ppm.

<u>Literature data</u> for 1,1,1,3,3,3-hexaethyldisiloxane $(Et_3Si)_2O$ for comparison:[80]

^{13}C NMR (101 MHz, CD_2Cl_2) δ 7.15, 6.93 ppm.

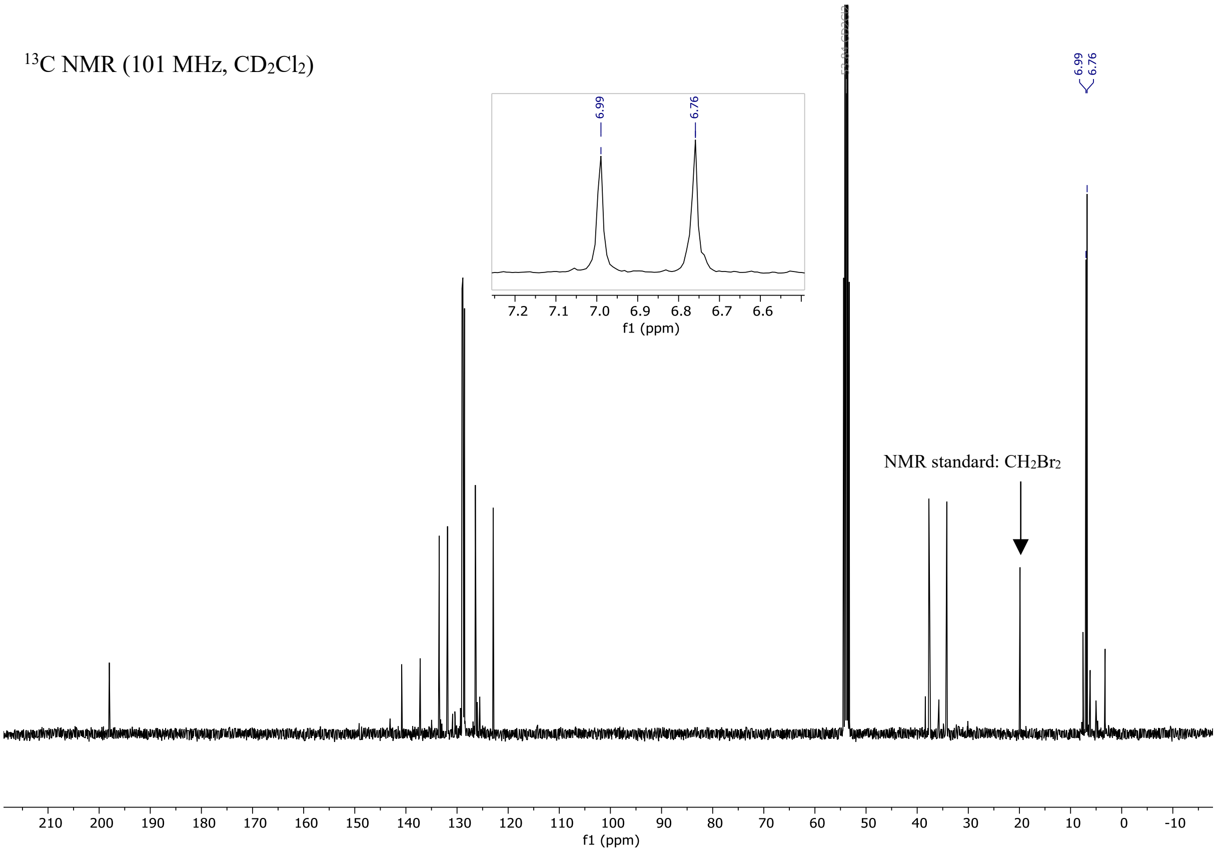

8. X-Ray Analysis

The crystal of **12**·2HCl was grown by slow evaporation from acetonitrile.

Single crystal X-ray diffraction data were collected with a Stadivari Diffractometer (STOE & Cie GmbH, Germany) equipped with an EIGER2 R500 detector (Dectris Ltd, Switzerland). Data were processed and scaled with the STOE software suite X-Area (STOE & Cie GmbH). Structures were solved with SHELXT (DOI: 10.1107/S2053273314026370) and refined with SHELXL[77] or Olex2[78]. Model building was done with Olex2 or ShelXle[79]. Structures were validated with CHECKCIF (https://checkcif.iucr.org/). See the respective CIF files for exact versions and more details.

Experimental data available online: https://www.ccdc.cam.ac.uk/structures/

(±)-2-((2*S*,3*S*,5*S*)-5-Methyl-5-phenyl-3-(triethylsilyl)tetrahydrofuran-2-yl)-N-(pyridin-2-ylmethyl)ethan-1-amine (12·2HCl)

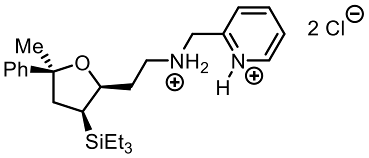

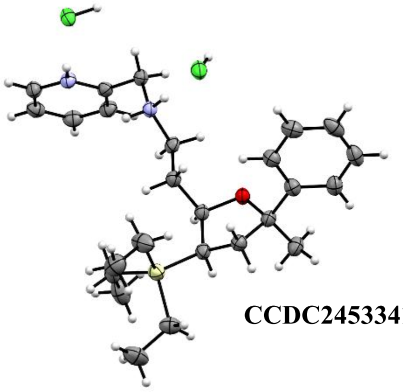

CCDC2453341

Identification code	JABZ514_a
Empirical formula	$C_{25}H_{42}N_2OSiCl_2$
Formula weight	485.59
Temperature/K	103.95
Crystal system	monoclinic
Space group	$P2_1/c$
a/Å	18.029(9)
b/Å	8.518(2)
c/Å	19.797(8)
α/°	90
β/°	115.831(2)
γ/°	90
Volume/Å^3	2736.4(19)
Z	4
ρ_{calc}g/cm^3	1.179
μ/mm^{-1}	2.686
F(000)	1048.0
Crystal size/mm^3	0.195 × 0.09 × 0.05
Radiation	Cu Kα (λ = 1.54178)
2Θ range for data collection/°	5.446 to 146.868
Index ranges	$-22 \leq h \leq 22$, $-8 \leq k \leq 10$, $-23 \leq l \leq 23$
Reflections collected	27407
Independent reflections	5189 [R_{int} = 0.0241, R_{sigma} = 0.0196]
Data/restraints/parameters	5189/4/292
Goodness-of-fit on F^2	1.121
Final R indexes [I>=2σ (I)]	R_1 = 0.0573, wR_2 = 0.1693
Final R indexes [all data]	R_1 = 0.0599, wR_2 = 0.1731
Largest diff. peak/hole / e Å^{-3}	0.99/-0.69

9. References

[59] B. M. Trost, Z. T. Ball, “Alkyne hydrosilylation catalyzed by a cationic ruthenium complex: efficient and general *trans* addition” *J. Am. Chem. Soc*. **2005**, *127*, 17644–17655.

[60] X. -Y. Yang, T. Feng, G. -Q. Wang, J.-H. Ding, Z. -H. Li, Y. Li, S. -H. He, J. -K. Liu, “Chemical constituents from cultures of the basidiomycete *Trichaptum* pargamenum” *Phytochem* **2014***, 104*, 89–94.

[61] G. Raju, R. Nomula, K. V. S. Ramakrishna, P. R. Krishna, “Total synthesis of (6*Z*,9*S*)-3,4-*trans*-9-hydroxy-3-methyldodec-*cis*-6- en-4-olide and (6*Z*)-3,4-*trans*-9-oxo-3-methyldodec-*cis*-6-en-4-olide γ-butyrolactones” *Tetrahedron Lett.* **2014**, *55*, 6655–6657.

[62] T. Liang, W. Zhang, T. -Y. Chen, K. D. Nguyen, M. J. Krische, “Ruthenium Catalyzed Diastereo- and Enantioselective Coupling of Propargyl Ethers with Alcohols: Siloxy-Crotylation *via* Hydride Shift Enabled Conversion of Alkynes to π-Allyls” *J. Am. Chem. Soc*. **2015**, *137*, 13066–13071.

[70] G. R. Fulmer, A. J. M. Miller, N. H. Sherden, H. E. Gottlieb, A. Nudelman, B. M. Stoltz, J. E. Bercaw, K. I. Goldberg, “NMR Chemical Shifts of Trace Impurities: Common Laboratory Solvents, Organics, and Gases in Deuterated Solvents Relevant to the Organometallic Chemist” *Organometallics* **2010**, *29*, 2176–2179.

[71] R. Nagase, N. Matsumoto, K. Hosomi, T. Higashi, S. Funakoshi, T. Misaki, Y. Tanabe, “Ti-direct, powerful, stereoselective aldol-type additions of esters and thioesters to carbonyl compounds: application to the synthesis and evaluation of lactone analogs of jasmone perfumes” *Org. Biomol. Chem.* **2007**, *5*, 151–159.

[72] C. R. Opie, N. Kumagai, M. Shibasaki, “Reversible Stereoselective Folding/Unfolding Fueled by the Interplay of Photoisomerism and Hydrogen Bonding” *Angew. Chem. Int. Ed.* **2017**, *56*, 3349–3353.

[73] P. Spieß, A. Sirvent, I. Tiefenbrunner, J. Sargueil, A. J. Fernandes, A. Arroyo-Bondía, R. Meyrelles, D. Just, A. Prado-Roller, S. Shaaban, D. Kaiser, N. Maulide, “Nms-Amides: An Amine Protecting Group with Unique Stability and Selectivity” *Chem. Eur. J.* **2023**, *29*, e202301312.

[74] E. M. Galathri, L. Di Terlizzi, M. Fagnoni, S. Protti, C. G. Kokotos, “Friedel–Crafts arylation of aldehydes with indoles utilizing arylazo sulfones as the photoacid generator” *Org. Biomol. Chem.* **2023**, *21*, 365–369.

[75] M. R. Chaulagain, G. M. Mahandru, J. Montgomery, “Alkyne hydrosilylation catalyzed by nickel complexes of N-heterocyclic carbenes” *Tetrahedron* **2006**, *62*, 7560–7566.

[76] D.-D. Ma, P. Gu, R. Li, “Asymmetric hydrogenation of 1-silyl-1-substituted alkenes for preparation of optically active silanes” *Tetrahedron Lett*. **2016**, *57*, 5666–5668.

[77] G. M. Sheldrick, “Crystal Structure Refinement with SHELXL” *Acta Cryst.* **2015**, *71*, 3–8.

[78] O. V. Dolomanov, L. J. Bourhis, R. J. Gildea, J. A. K. Howard, H. Puschmann, “OLEX2: a complete structure solution, refinement and analysis program” *J. Appl. Cryst.* **2009**, *42*, 339–341.

[79] C. B. Hübschle, G. M. Sheldrick, B. Dittrich, “ShelXle: a Qt graphical user interface for SHELXL” *J. Appl. Cryst.* **2011**, *44*, 1281–1284.

[80] R. Buhaibeh, N. Thyagarajan, G. Bousrez, L. Monsigny, T. Cantat, E. Nicolas, “Scandium-Catalyzed Highly Selective Deoxygenation of Alcohols by Using Hydrosilanes as Reductants” *Chem. Eur. J.* **2025**, *31*, e202501596.

[81] K. Garcés, F. J. Fernández-Alvarez, V. Polo, R. Lalrempuia, J. J. Pérez-Torrente, L. A. Oro, “Iridium-Catalyzed Hydrogen Production from Hydrosilanes and Water” *ChemCatChem* **2014**, *6*, 1691 – 1697.
